# High-resolution isotopic evidence of specialised cattle herding in the European Neolithic

**DOI:** 10.1371/journal.pone.0180164

**Published:** 2017-07-26

**Authors:** Claudia Gerling, Thomas Doppler, Volker Heyd, Corina Knipper, Thomas Kuhn, Moritz F. Lehmann, Alistair W. G. Pike, Jörg Schibler

**Affiliations:** 1 Institute of Prehistory and Archaeological Science, Department of Environmental Sciences, University of Basel, Basel, Switzerland; 2 Department of Archaeology & Anthropology, University of Bristol, Bristol, United Kingdom; 3 Curt-Engelhorn Centre Archaeometry, Mannheim, Germany; 4 Biogeochemistry, Department of Environmental Sciences, University of Basel, Basel, Switzerland; 5 Department of Archaeology, University of Southampton, Southampton, United Kingdom; New York State Museum, UNITED STATES

## Abstract

Reconstructing stock herding strategies and land use is key to comprehending past human social organization and economy. We present laser-ablation strontium and carbon isotope data from 25 cattle (*Bos taurus*) to reconstruct mobility and infer herding management at the Swiss lakeside settlement of Arbon Bleiche 3, occupied for only 15 years (3384–3370 BC). Our results reveal three distinct isotopic patterns that likely reflect different herding strategies: 1) localized cattle herding, 2) seasonal movement, and 3) herding away from the site year-round. Different strategies of herding are not uniformly represented in various areas of the settlement, which indicates specialist modes of cattle management. The pressure on local fodder capacities and the need for alternative herding regimes must have involved diverse access to grazing resources. Consequently, the increasing importance of cattle in the local landscape was likely to have contributed to the progress of socio-economic differentiation in early agricultural societies in Europe.

## Introduction

Understanding herding practices of cattle (*Bos taurus*) and territorial land use is key for comprehending human social organization and economy, particularly in Europe of the 4^th^ and 3^rd^ millennium BC when the first stratified societies emerged [1] and the use of secondary products gained in importance [2]. In addition to meat production, secondary husbandry products, such as milk, hair, manure and animal power, became the main motivation for keeping cattle [3]. A higher demand and the efficient exploitation of these products necessitated the maintenance of larger herds [4]. In turn, larger herds increased the pressure on local grazing resources and may have been associated with the advent of more complex herding strategies and enhanced mobility. Such mobility, e.g., in the form of transhumance, allowed the most fertile land in close vicinity to the settlements to be utilized exclusively for the production of crops [5] and fodder for overwintering animals [6]. Extensive mobile cattle herding thus permits optimal resource management of different types of grazing grounds in varying altitudes and distances from the permanent settlement. As a consequence, this exploitation triggered the colonization of poor soils, dense woodlands and higher altitudes by human settlers. The enhanced mobility forged new ways of life, which fundamentally altered customs and material cultures [7], and formed the European landscape [8]. Different mobility regimes have been demonstrated for historical times [9], and from ethnographical records [10], but conclusive evidence for prehistoric cattle mobility is rare: The seasonal movement of domesticates away from the settlement to areas of open pasture (e.g., uplands) was a strategy which has been suggested for the European Neolithic [11, 12]. In prehistoric Switzerland, indirect archaeological and archaeobiological evidence for human and animal mobility includes settlement structures at high altitudes (e.g., huts, pens) and the finding of alpine plant and animal remains (e.g., alpine speedwell, alpine ibex) in lowland settlements of the late Neolithic [13].

**Fig 1:**
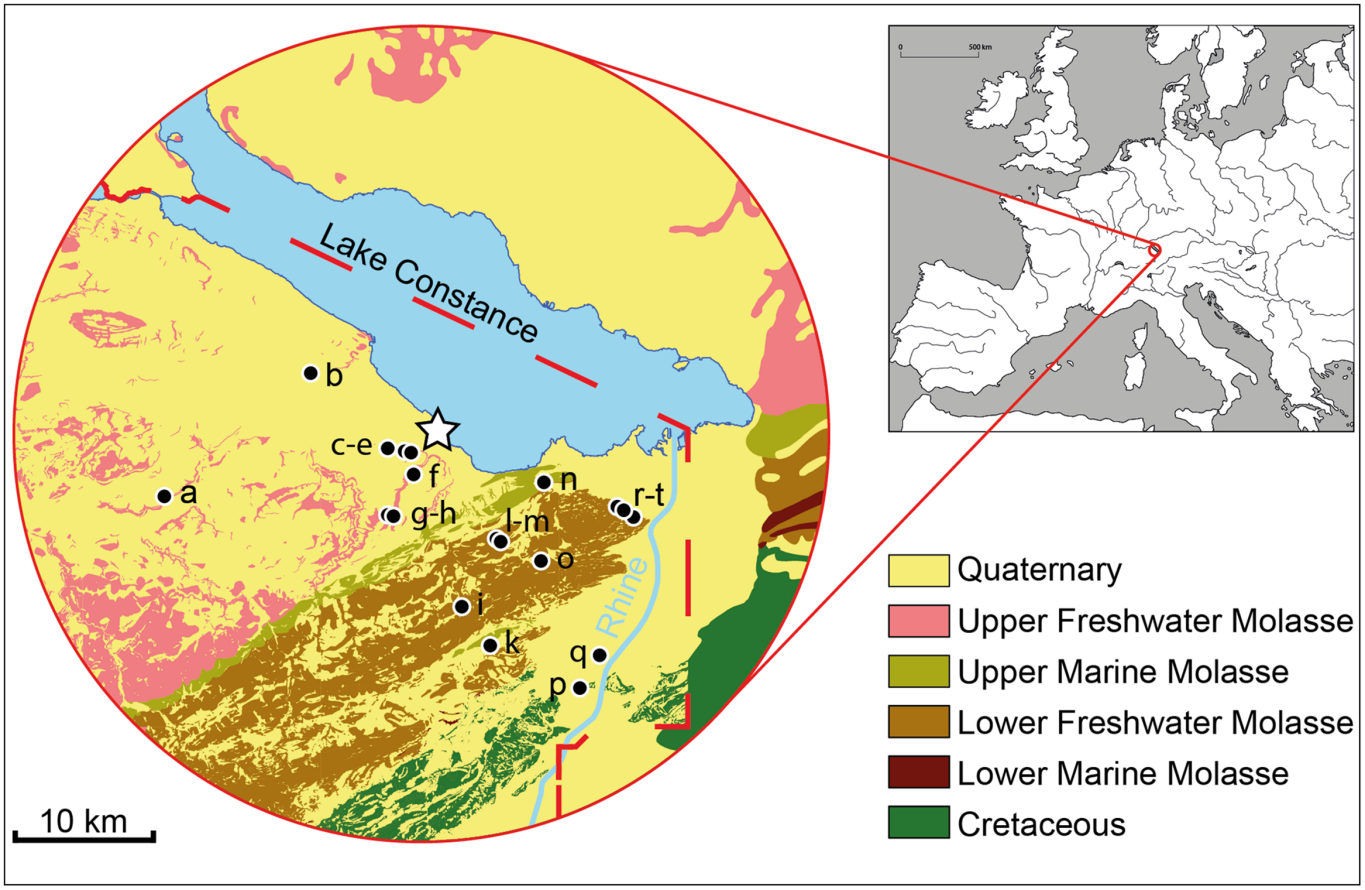
Geology of the study area. The location of Arbon Bleiche 3 (white star) in Central Europe with major bedrock units [17, 18] in a 30 km perimeter. The baseline sampling for bioavailable ^87^Sr/^86^Sr determination (black dots; SI section III) covered all geological units in this area, with some samples also collected outside the mapped circle (cf. S2 Table). The ^87^Sr/^86^Sr ranges are as follows: Quaternary (0.7086–0.7104, n = 8), Upper Freshwater Molasse (0.7082–0.7091, n = 4), Upper Marine Molasse (0.7084–0.7094, n = 2), Lower Freshwater Molasse (0.7084–0.7118, n = 6), Lower Marine Molasse (0.7082, n = 1), Cretaceous (0.7077–0.7085, n = 5).

**Fig 2:**
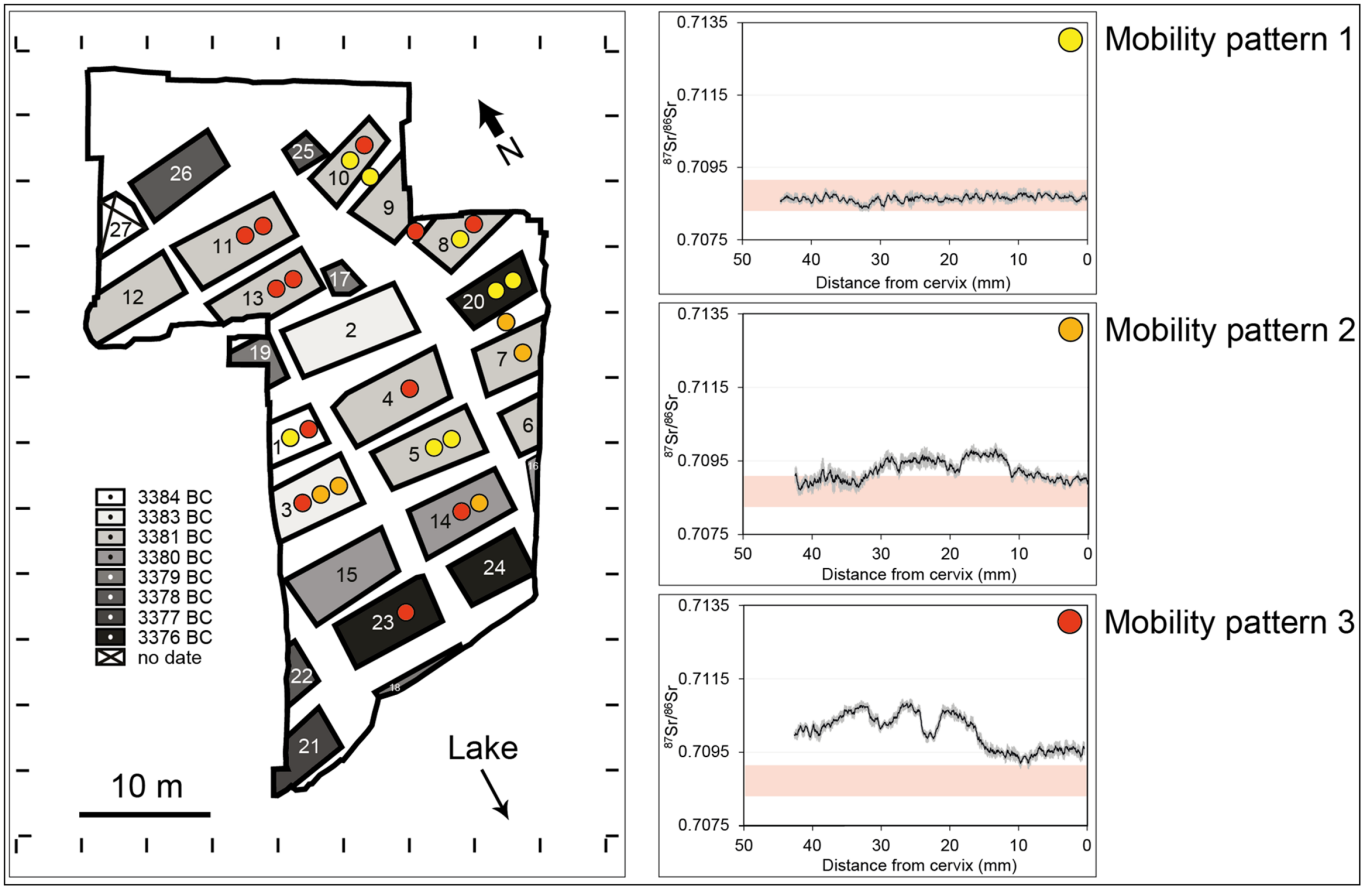
Cattle mobility patterns at Arbon Bleiche 3. Settlement plan with reconstructed houses (modified after [15]) and distribution of cattle mobility patterns (MP) 1–3 (left). The grey-shading represents dates of construction. ^87^Sr/^86^Sr laser ablation profiles (right) illustrate representative patterns of Sr isotope ratio variations along the tooth enamel for each mobility regime (complete data set in S1 Fig).

Arbon Bleiche 3 is an ideal site to explore direct (isotopic) evidence for Neolithic cattle mobility at a time when the systematic utilization and exploitation of secondary husbandry products likely gained in importance. The settlement, located at Lake Constance (NE Switzerland; Fig 1), was occupied for only 15 years (3384–3370 BC) [14–16]. Ground plots for 27 houses were identified. 26 of them could be dated precisely by dendrochronology, providing a unique temporal context for studying Neolithic cattle management down to the level of single houses (Fig 2; Supporting Information (SI), section I).

Strontium isotope (^87^Sr/^86^Sr) analysis is a key method for reconstructing past human and animal mobility [19–21], which has been successfully applied to cattle remains [11, 22, 23]. Sr isotope measurements are traditionally conducted on bulk enamel samples or on micro-drilled sequential samples, usually by thermal-ionization mass spectrometry (TIMS). More recently, laser-ablation multi-collector inductively-coupled plasma mass spectrometry (LA-MC-ICP-MS) has become a highly promising approach for identifying the differences in the ^87^Sr/^86^Sr along tooth crowns, at very high temporal resolution [24, 25]. Often in combination with light stable isotope analyses (δ^13^C, δ^15^N and δ^18^O), recent Sr isotope studies have been used to investigate the links between cattle management, birth seasons and dairying activities [26, 27]. Here, we use LA-MC-ICP-MS strontium isotope ratio measurements along the growth axis of cattle tooth enamel as indicators of mobility to reconstruct patterns of herding. The approach makes use of the fact that the Sr isotopic compositions of geological units, soils, plants, and the hard tissues of the mammals in the same environment are closely related. The Sr isotopic signatures may be highly characteristic and can vary over relatively short geographic distances. The local ^87^Sr/^86^Sr signature is passed along the food chain and is incorporated into the teeth of animals during enamel mineralization, with negligible fractionation [20]. Provided that the ^87^Sr/^86^Sr values in the environment are indeed sufficiently different, enamel Sr isotope measurements may allow the identification of potential pasture areas at and nearby former settlements, and cattle mobility across geological units. The landscape around Arbon Bleiche 3 is dominated by Quaternary moraine deposits and outcrops of Tertiary Upper Freshwater molasse (Fig 1). To the south and east (<15 km), additional geological units of the Tertiary Upper Marine and Lower Freshwater molasse crop out. The sediments of the molasse are, with respect to their Sr-isotopic composition, clearly distinguishable from the closest high-alpine mountain chains even further south, which are dominated by Cretaceous (Alpstein and Churfirsten) and Permian sediments (south of Walensee) (SI, section II). To test also whether potential pasture grounds varied ecologically, e.g., forest pasture vs. open habitats [28, 29], C isotope measurements were conducted on mandibles or maxillae of the sampled cattle. As bone is constantly remodeled throughout the lifetime of mammals [30, 31], carbon isotope ratios measured in bone collagen reflect the protein component of the animal’s diet averaged over several years. Hence, it is important to note that the C isotope data do not correspond directly to the Sr isotope ratio records measured in tooth enamel.

## Materials and methods

At Arbon Bleiche 3, 39 permanent molar teeth (M1, M2, M3) from 25 cattle were selected for high-resolution ^87^Sr/^86^Sr LA-MC-ICP-MS analyses (Table 1). The teeth were associated with twelve of the 27 houses, predominantly in the northern part of the site where most of the cattle remains were excavated (Fig 2; SI, section I) [15, 32]. Although tooth enamel mineralization is a complex process [33, 34], cattle molar crowns form sequentially, progressing from the cusp to the cervix [35–37]. First molars (M1) start mineralizing *in utero*, and mineralization ends at the age of ~2 to 3 months. Second molars (M2) mineralize between the age of 1 and 12–13 months, and third molars (M3) between 9–10 months and 23–24 months [38, 39]. For twelve cattle it was possible to perform analyses on rows of teeth (2 or 3 consecutive molars), yielding expanded time-series data sets of up to two years.

**Table 1:** Contextual information for sampled cattle (*Bos taurus*) specimens. Listed are the length of tooth enamel (as an indicator of tooth wear), age at death (n.a. = not assigned), house attribution, mobility pattern (MP), ^87^Sr/^86^Sr data (tooth enamel) and δ^13^C data (bone collagen).

Ind.	Skeletal element	Age at death (y)	House	Built (y BC)	MP	Lab ID	Tooth/ Bone	Enamel (mm)	^87^Sr/^86^Sr Mean	^87^Sr/^86^Sr Min	^87^Sr/^86^Sr Max	^87^Sr/^86^Sr Range	Coll%	%C	%N	C/N	δ^13^C‰ (vs. VPDB)
ARB 2	Maxilla left	c. 9	1	3384	1	ARB 2.2.1	M2	41.9	0.70836	0.70745	0.70911	0.00166					
						ARB 2.3.1	M3	44.2	0.70831	0.70745	0.70917	0.00172					
						ARB 2.1	Bone						24.3	44.1	15.7	3.3	-23.9
ARB 10	Maxilla right	c. 2–3	3	3383	2	ARB 10.2.1	M2	46.4	0.70925	0.70808	0.71055	0.00247					
						ARB 10.1	Bone						19.7	44.7	15.8	3.3	-23.2
ARB 14	Mandible right	c. 2–3	3	3383	2	ARB 14.2.1	M2	45.5	0.70924	0.70808	0.71052	0.00244					
						ARB 14.3.1	M3	52.6	0.70919	0.70799	0.71067	0.00269					
						ARB 14.1	Bone						11.3	43.7	15.6	3.3	-22.8
ARB 16	Maxilla left	c. 2–3	4	3381	3	ARB 16.3.1	M1	32.7	0.71054	0.70962	0.71203	0.00241					
						ARB 16.2.1	M2	42.5	0.71001	0.70855	0.71264	0.00409					
						ARB 16.1	Bone						19.4	45.1	16.0	3.3	-22.3
ARB 19	Mandible left	c. 9	5	3381	1	ARB 19.2.1	M3	46.7	0.70858	0.70795	0.70933	0.00138					
						ARB 19.1	Bone						19.9	44.5	16.0	3.3	-23.4
ARB 22	Mandible left	c. 6.5	7	3381	2	ARB 22.2.1	M2	38.7	0.70905	0.70790	0.71033	0.00243					
						ARB 22.1	Bone						16.0	44.0	15.9	3.2	-23.9
ARB 23	Maxilla right	c. 6.5	8	3381	3	ARB 23.2.1	M2	46.8	0.70968	0.70874	0.71066	0.00192					
ARB 25	Mandible right	c. 11.5	9/10	-	1	ARB 25.2.1	M3	44.6	0.70864	0.70808	0.70928	0.00120					
ARB 26	Mandible	n.a.	10	3381	1	ARB 26.2.1	M2?	52.3	0.70871	0.70792	0.70951	0.00158					
ARB 27	Mandible right	c. 2–3	11	3381	3	ARB 27.2.1	M2	26.4	0.70984	0.70866	0.71107	0.00240					
ARB 29	Mandible left	c. 1.5	11	3381	3	ARB 29.2.1	M2	44.4	0.71005	0.70873	0.71119	0.00246					
						ARB 29.1	Bone						20.7	43.4	15.5	3.3	-22.6
ARB 33	Maxilla left	c. 9	13	3381	3	ARB 33.4.1	M1	17.9	0.71204	0.71113	0.71283	0.00170					
						ARB 33.2.1	M2	27.7	0.71029	0.70886	0.71204	0.00318					
						ARB 33.3.1	M3	30.2	0.70997	0.70809	0.71192	0.00384					
						ARB 33.1	Bone						21.8	43.9	15.7	3.3	-22.5
ARB 34	Mandible left	c. 2–3	13	3381	3	ARB 34.2.1	M2	46.6	0.71007	0.70903	0.71135	0.00232					
						ARB 34.1	Bone						16.3	44.1	15.7	3.3	-22.4
ARB 43	Mandible right	c. 1.5	20	3376	1	ARB 43.2.1	M2	50.9	0.70866	0.70782	0.70982	0.00199					
						ARB 43.1	Bone						20.5	43.7	15.5	3.3	-24.6
ARB 109	Mandible left	c. 6.5	14	3379	2	ARB 109.2.1	M2	26.3	0.70901	0.70826	0.71023	0.00196					
						ARB 109.1	Bone						18.2	42.9	15.3	3.3	-22.5
ARB 110	Mandible left	c. 9	8/9	-	3	ARB 110.3.1	M1	11.7	0.70956	0.70887	0.71025	0.00138					
						ARB 110.2.1	M2	18.9	0.70964	0.70859	0.71135	0.00276					
						ARB 110.4.1	M3	27.2	0.71028	0.70924	0.71130	0.00207					
						ARB 110.1	Bone						20.3	43.3	15.7	3.2	-22.4
ARB 111	Mandible right	c. 6.5	10	3381	3	ARB 111.2.1	M2	26.8	0.71002	0.70896	0.71121	0.00225					
						ARB 111.1	Bone						19.5	42.9	15.4	3.2	-23.0
ARB 112	Mandible left	c. 6.5	20	3376	1	ARB 112.2.1	M2	40.7	0.70896	0.70825	0.70960	0.00134					
						ARB 112.3.1	M3	47.3	0.70891	0.70822	0.70982	0.00160					
						ARB 112.1	Bone						20.3	42.3	15.1	3.3	-23.3
ARB 113	Mandible right	c. 6.5	5	3381	1	ARB 113.2.1	M2	39.4	0.70855	0.70799	0.70916	0.00116					
						ARB 113.3.1	M3	45.3	0.70868	0.70778	0.70983	0.00205					
						ARB 113.1	Bone						20.3	42.3	15.1	3.3	-22.8
ARB 114	Mandible left	c. 9	23	3376	3	ARB 114.2.1	M2	32.3	0.70984	0.70848	0.71188	0.00340					
						ARB 114.3.1	M3	43.1	0.71004	0.70809	0.71259	0.00450					
						ARB 114.1	Bone						18.3	42.4	15.2	3.3	-22.1
ARB 115	Mandible left	c. 9	3	3383	3	ARB 115.2.1	M2	13.6	0.71028	0.70883	0.71140	0.00258					
						ARB 115.3.1	M3	13.8	0.71002	0.70909	0.71165	0.00256					
						ARB 115.1	Bone						19.2	44.3	15.7	3.3	-22.2
ARB 116	Mandible left	c. 9	14	3379	3	ARB 116.2.1	M2	15.4	0.70982	0.70894	0.71069	0.00176					
						ARB 116.1	Bone						17.6	41.0	14.5	3.3	-22.2
ARB 117	Mandible left	c. 9	1	3384	3	ARB 117.2.1	M2	26.4	0.71159	0.70968	0.71426	0.00458					
						ARB 117.3.1	M3	36.3	0.71198	0.70959	0.71417	0.00458					
						ARB 117.1	Bone						20.2	44.9	15.8	3.3	-21.8
ARB 118	Mandible left	c. 6.5	8	3381	1	ARB 118.2.1	M2	32.7	0.70884	0.70766	0.70996	0.00230					
						ARB 118.3.1	M3	36.3	0.70876	0.70790	0.70988	0.00199					
						ARB 118.1	Bone						19.8	43.4	15.5	3.3	-24.7
ARB 119	Mandible right	c. 6.5	7/20	-	2	ARB 119.2.1	M2	42.0	0.70901	0.70794	0.71038	0.00244					
						ARB 119.3.1	M3	47.8	0.70959	0.70830	0.71098	0.00269					
						ARB 119.1	Bone						19.1	43.4	15.3	3.3	-22.1

### Strontium isotope analysis (LA-MC-ICP-MS) of cattle teeth

Cattle teeth were provided by the Archaeological Service of the Canton Thurgau in Frauenfeld, Switzerland. Sampled skeletal elements were investigated for age at death at the Institute of Prehistory and Archaeological Science (IPAS), University of Basel, and published in 2004 [40]. An archaeozoological MNI-based approach to sampling was undertaken to ensure that the teeth and bones originated from different cattle. For Sr isotope analysis sample preparation in Basel, the surface of each tooth was carefully cleaned using a dental burr and hand drill. An enamel transect (~2 mm thick), covering the complete growth axis of the tooth, was cut out using a diamond-impregnated dental disk. The enamel was fixed in a round PTFE mount and embedded in epoxy resin (Biodur^®^ E12 + Biodur^®^ E1 hardener 100:28). After 12 hours under vacuum and subsequent hardening in an oven at 35°C for 48 hours, the surface of the resin of the sample mount was ground to expose the surface of the enamel, which was then polished with SiC paper (P1200). The prepared sample mount was then transferred to the National Oceanography Centre in Southampton (NOCS) for Sr isotopic analysis, which was performed on a Finnegan Neptune multi-collector ICP-MS with a New Wave 193 nm ArF homogenized excimer laser. Plasma conditions were optimized for low oxide formation by ensuring ^254^(UO)^+^/(^238^U^+^) < 0.1% [24, 25] to reduce the isobaric interference at *m*/*z* 87 primarily due to ^40^Ca^31^P^16^O^+^ [41–43], which is a major constituent of the enamel matrix. Additional isobaric interferences can derive from doubly-charged rare-earth elements (REE), calcium-calcium and calcium-argide dimers, ^87^Rb or ^86^Kr. Contribution from ^86^Kr was corrected for by running gas blanks, and an ^87^Rb correction was applied assuming an ^87^Rb/^85^Rb ratio of 0.385617. Dimer interferences were monitored via the ^84^Sr/^86^Sr and ^89^Y was measured as a proxy for REEs. Sr isotope time-series data were obtained by continuous laser ablation along the tooth’s growth axis (at 20 or 25 μms^-1^, depending on the size of the tooth). The laser pulse repetition was 15 Hz, the spot size was 150 μm, and laser fluence was 8.6 Jcm^-2^. Prior to the actual analysis, the laser track was laser-cleaned using the identical repetition rate and spot size, but at a higher traverse rate (100 μms^-1^). Analysis of an in-house ashed bovine pellet standard (BP), with three repeated measurements after every third sample, revealed a mean offset (Laser ablation-TIMS) in ^87^Sr/^86^Sr of +44±33 ppm (1σ), determined from 279 analyses over 18 months. This is similar to the offset reported by other laboratories using a similar methodology [44]. While this shows that interferences could not be completely eliminated, they have been reduced to the point that the reproducibility was much smaller than the observed inter-tooth variability of 952 ppm, and is therefore considered insignificant to our interpretation of the isotope data. The accuracy of laser-ablation-derived ^87^Sr/^86^Sr ratios was further verified by comparison with discrete analyses of micro-drilled samples using conventional TIMS (S2 Fig).

### Discrete strontium isotope analysis of baseline samples

For baseline ^87^Sr/^86^Sr determination, dentine of cattle and red deer (*Cervus elaphus*) was analysed using a LA-MC-ICP-MS in the laboratory facilities at the National Oceanography Centre Southampton, UK. The laser spot was initially cleaned for 5 seconds with a laser repetition rate of 15 Hz and spot size of 150 μm. Data were collected at the same spot in 60 one-second cycles with a laser repetition rate of 10 Hz and a spot size of 150 μm.

For discrete Sr isotope analysis of vegetation samples (collected in spring 2013 and 2014), leaves were rinsed with demineralized water soon after collection, and dried overnight at 40–50°C. Sample treatment followed established methods [45]. Briefly, dried leaves were ground manually and ashed in acid-washed crucibles at 550°C for 12 h. An aliquot of approximately 20 mg of ashed leaves was transferred into clean PFA (Savillex^™^) vials and dissolved in 15 M HNO_3_. 1 ml of 30% H_2_O_2_ was added to further dissolve the sample at 140–160°C. Samples were evaporated to dryness and dissolved in 2 ml 3M HNO_3_, ultrasonicated and centrifuged, before loading onto ion-exchange columns. Water aliquots (collected in spring 2013 and 2014) were transferred to clean PFA (Savillex^™^) vials. Sample preparation followed established methods [45]. Aliquots were evaporated to dryness, dissolved in 2 ml concentrated HCl and dried down. After adding 2 ml of concentrated HNO_3_, samples were again dried down. Aliquots were then taken up in 1 or 2 ml 3 M HNO_3_, ultrasonicated and centrifuged, prior to loading onto ion-exchange columns. The teeth of pigs (*Sus domesticus*) were sampled by cutting pieces of enamel representing the complete growth axis using a flexible diamond-impregnated dental disk and hand drill. The procedure is described in [46, 47]. Briefly, the inner and outer surfaces of the enamel were treated with a dental burr to remove any adhering contamination. Enamel samples were additionally cleaned with pure water in an ultrasonic bath, dried and then dissolved in 3 ml 7 M HNO_3_. Samples were centrifuged to remove any adhering powder. The supernatant fluid was dried down and dissolved in 3 M HNO_3_. An aliquot of this solution, representing 3 mg of solid enamel, was made up to 0.5 ml 3 M HNO_3_, before loading onto ion-exchange columns. Dissolved Sr (from either plant, water or enamel samples) was separated using standard ion-exchange chromatography using 70 μl of Eichrom Sr spec resin (50–100 μm). Strontium was separated from the sample matrix by washing the column with 2.5 ml 3 M HNO_3_ and eluted using 1.5 ml ultrapure water. After separation, the Sr-containing eluate was dried down. After dissolving in 1 μl 10% HNO_3_, samples were loaded onto rhenium filaments, preconditioned with 1 μl TaCl_5_ solution and 1 μl 10% H_3_PO_4_. Isotope ratios were measured on a Thermo Finnigan TRITON Thermal Ionization Mass Spectrometer (TIMS). Data were calibrated using the standard reference material NIST SRM 987 with a reported ^87^Sr/^86^Sr ratio of 0.710248 [48, 49]. All TIMS Sr-isotope measurements were performed in the laboratory facilities of the Department of Earth Sciences, University of Bristol, UK. The typical precision for ^87^Sr/^86^Sr measurements was ± 0.00001 (in archaeological tooth enamel).

### Stable carbon isotope analysis of cattle bones

For carbon isotope analysis, collagen extraction followed established methods [50], with modifications [51] and omission of the ultrafiltration step [52, 53]. Chunks of bone were cut and subsamples were taken from a clean surface using a dental drill. 200–1000 mg of cleaned sample material was demineralized in 10 ml of 0.5 M HCl at 4°C for two weeks. Samples were then rinsed with ultrapure water before treatment with 10 ml of 0.1 M NaOH at 4°C for about 24 h. After rinsing, 4 ml of ultrapure water and 200 μl of 0.5 M HCl were added, and the samples gelatinized at 70°C for 48 h. The solution containing the dissolved collagen was filtered using Ezee Filter separators (Elkay, UK) with a pore size of 60–90 μm, frozen at -20°C, and lyophilized for 48 h. For carbon isotope analyses, between 0.5 and 1.0 mg of freeze-dried sample were weighed in duplicate into tin capsules, and introduced into an elemental analyser coupled to an isotope ratio mass spectrometer (EA-IRMS) (INTEGRA2, Sercon Ltd., Crewe, UK) in the laboratory facilities of the Department of Environmental Sciences, University of Basel, Switzerland. Carbon isotope data were calibrated using international reference materials (IAEA-CH-6, USGS40) and an in-house standard (EDTA), and reported in the conventional δ-notation as δ^13^C in ‰ relative to Vienna Pee Dee Belemnite (V-PDB). Based on replicate analyses of the isotopic reference materials, our in-house standard and a selected bone collagen sample which were analysed recurrently in each analytical sequence, the analytical reproducibility for δ^13^C was generally ≤ 0.2‰. Collagen preservation and purity was checked following established methods [54–56].

## Results

**Fig 3:**
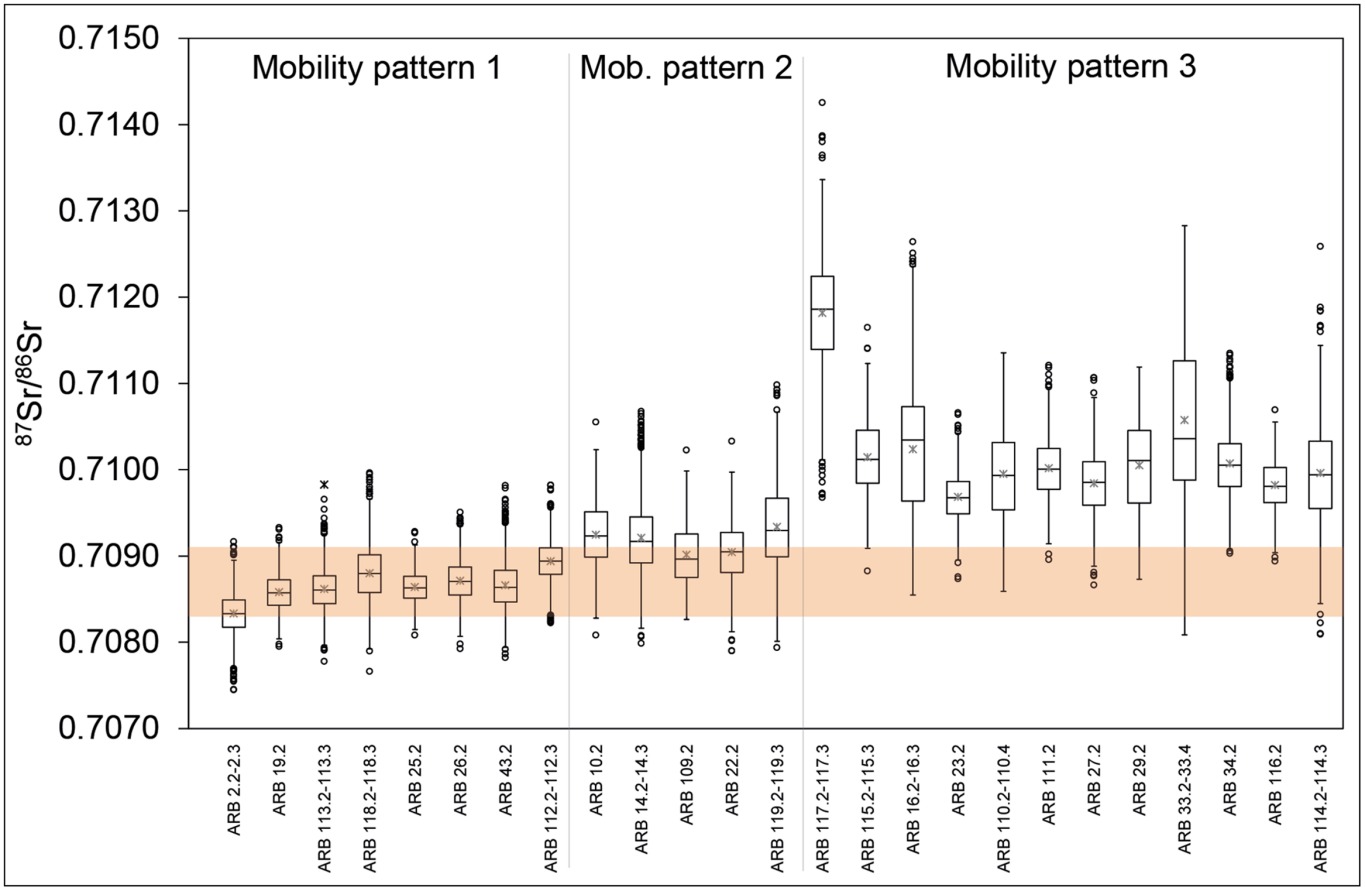
Box plot of ^87^Sr/^86^Sr data for cattle based on individual intra-tooth measurements. Cattle (*Bos taurus*) are grouped by mobility pattern and chronological order (cf. Table 1). The central black line in each box represents the median and the cross represents the mean. The coloured bar represents the local ^87^Sr/^86^Sr range (0.7083–0.7091).

^87^Sr/^86^Sr values for sampled tooth enamel obtained through LA-MC-ICP-MS analysis range from 0.7075 to 0.7143 (based on individual laser measurements) with a mean of 0.7095 ± 0.0019 (2σ) (Fig 3 and S2 Fig). Sr isotope data from the tooth enamel of archaeological pigs, the dentine of cattle and red deer, as well as modern water and vegetation samples suggest that cattle grazing in close vicinity of the settlement site should display ^87^Sr/^86^Sr ratios between 0.7083 and 0.7091 (S3 Fig; SI, section II). A considerable number of samples with ^87^Sr/^86^Sr ratios that are well above this range therefore indicates that some cattle were pastured away from the settlement. Modern reference sampling of the vegetation in the area between Arbon Bleiche 3 and the high mountain ranges located in a distance of >30 to 50 km confirm that nearby and distant pasture grounds can be distinguished based on the isotopic composition, with ^87^Sr/^86^Sr up to 0.7130 in the more distant locations (S1 Table; SI, section II). The observed ^87^Sr/^86^Sr ratios allow us to distinguish between three different patterns of cattle mobility (Figs 2 and 3 and S2 Fig):

**Mobility pattern (MP) 1: Stationary, local (n = 8 individuals):** MP1 is characterized by ^87^Sr/^86^Sr trends that are essentially invariant (≤0.0005) and fully consistent with the local baseline range of 0.7083–0.7091. The second and third quartiles of their boxplots are within the local range. Cattle remains that display MP1 were mainly found in the northern and central part of the settlement (ARB 2, 19, 25, 26, 43, 112, 113, 118).

**Mobility pattern 2: Mobile, partly local (n = 5 individuals):** Cattle assigned to MP2 exhibit mixed signals of local and non-local ^87^Sr/^86^Sr ratios. MP2 includes cattle that show a changeover from local to non-local ^87^Sr/^86^Sr values (ARB 119), from non-local to local ^87^Sr/^86^Sr values (ARB 109), or apparently alternating cycles of local and non-local ^87^Sr/^86^Sr (ARB 10, 14, 22). Samples displaying MP2 were primarily found in the central part of the settlement, and could be linked to houses that were situated close to each other.

**Mobility pattern 3: Mobile, non-local (n = 12 individuals):** MP3 includes ^87^Sr/^86^Sr trends that show exclusively or mostly non-local signatures. The second and third quartiles of the boxplots lie clearly outside the expected local ^87^Sr/^86^Sr range. Samples that exhibit MP3 show ^87^Sr/^86^Sr ratios that are either persistently distinct from the local ^87^Sr/^86^Sr range of the site (ARB 23, 27, 29, 33, 34, 110, 111, 115, 116, 117), or that show dominantly non-local signatures (with some overlap with the local ^87^Sr/^86^Sr range; ARB 16, 114). MP3 specimens were found all over the settlement with a slight predominance in the northern part.

**Fig 4:**
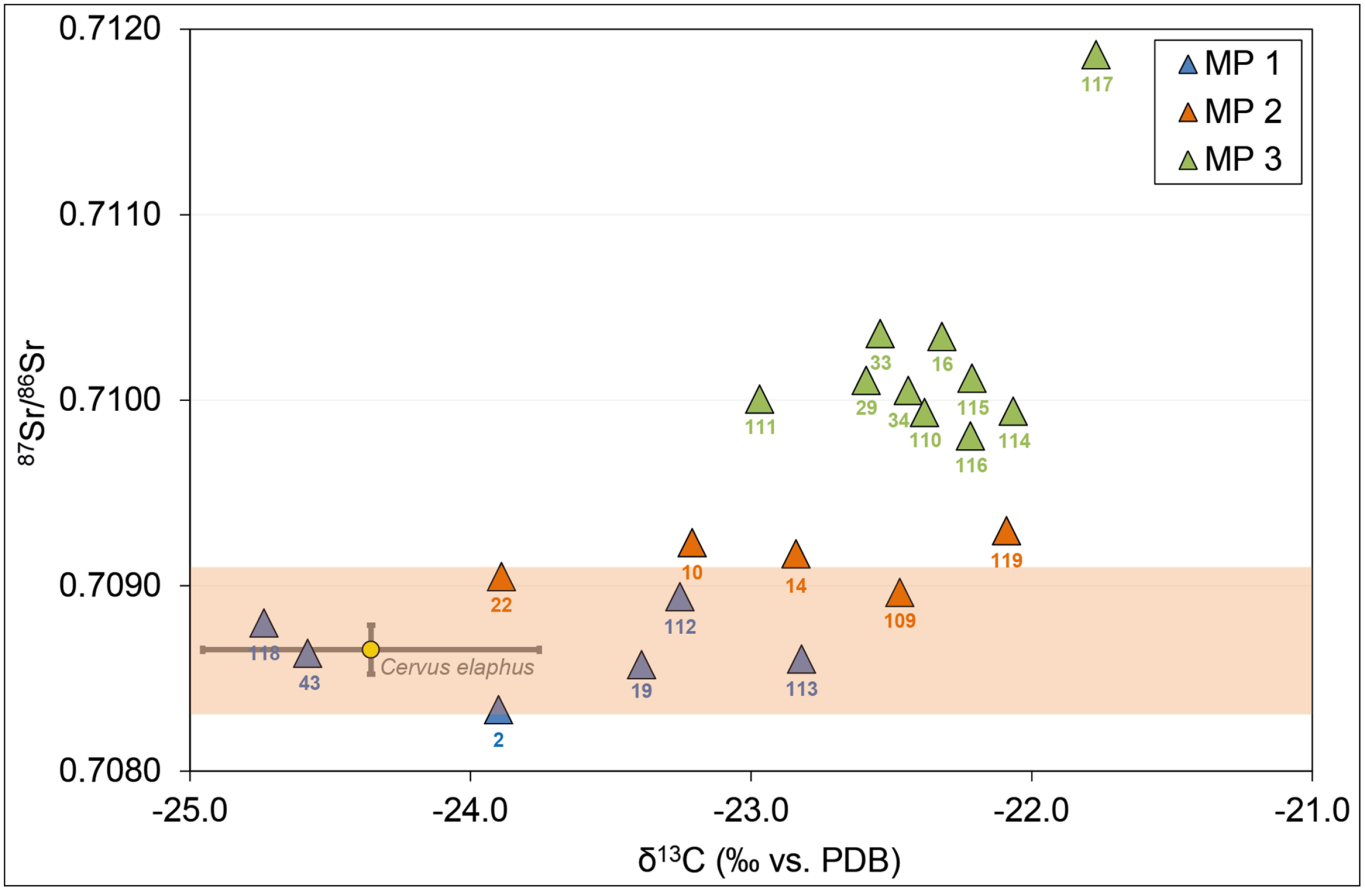
Scatter plot of δ^13^C values of cattle bone collagen and average ^87^Sr/^86^Sr data of tooth enamel. δ^13^C and ^87^Sr/^86^Sr (mean of all intra-tooth measurements for an individual) data for cattle (*Bos taurus*) are given in Table 1. Sample ID’s are indicated (excl. ARB). Red deer (*Cervus elaphus*) isotopic data (n = 6; 1σ standard error) are also provided for comparison. The grey bar represents the local ^87^Sr/^86^Sr range (0.7083–0.7091).

The average ^87^Sr/^86^Sr ratios of the teeth assigned to the three mobility patterns, respectively, differed significantly (Kruskal-Wallis test: *p* < 0.001; XLSTAT, version 2010). An identification and distinction of the three mobility patterns is also supported by the characteristic carbon isotope ratios of bone collagen with significantly (MP1 ≠ MP3; Mann-Whitney test: *p* = 0.002) different mean δ^13^C values of -23.8 ± 0.7‰, -22.8 ± 0.6‰ and -22.4 ± 0.3‰ (1σ) for MP1, 2, and 3 respectively (Fig 4 and Table 1). The difference in the mean δ^13^C for each pool of samples suggests that cattle mobility was associated with an apparent change in pasture grounds or grazing resources.

Cattle from the same house often displayed the same mobility pattern (houses 3, 5, 11, 13, 20). Yet for some houses, different mobility patterns were observed (houses 1, 3, 8, 10, 14). MP1 and MP2 in combination never co-occurred in the same house (Fig 2). Moreover, individual MP’s were not restricted to houses with similar erection dates and they showed no clear relation to the age of the animals (Table 1).

## Discussion

### The landscape and its capacity for cattle husbandry

The interpretation of the cattle movement patterns requires a detailed understanding of the Neolithic landscape and its resources around Lake Constance. The surroundings of Arbon Bleiche 3 were dominated by mixed forests of beech (*Fagus sylvatica*), oak (*Quercus* sp.), silver fir (*Abies alba*), hazel (*Corylus avellana*), ash (*Fraxinus excelsior*), and birch (*Betula* sp.) [16]. As a consequence of long-lasting human activity [14], the landscape resembled a mosaic of densely forested environments and more open settings, both natural (windfall, flood plains, lake shores) and man-made (agricultural fields, fallow ground, orchards) [16]. Human activities, with severe impact on the landscape, created new pasture grounds [57, 58], which aimed at increasing the productivity and resource capacity of the environment [59]. The multifaceted exploitation of the land around settlement sites (e.g., for timber, coppicing, pollarding, hunting, gathering, agriculture, orchards) required sustainable land management practices [58], which implies a certain limitation of land available for animal husbandry in the adjacent forests. Neighbouring settlements with additional resource demands may have created additional pressure on the landscape in the nearby surroundings [60, 61]. Plant remains found in cattle dung demonstrate the importance of wood pasture at Arbon Bleiche 3, and suggest that grazing within and at the edges of forests, and at ruderal places (including lakeshores) was common [62]. Various herding regimes for cattle may have helped to avoid or mitigate overexploitation of the available resources, and diverse (and more distant) grazing grounds were likely targeted. Herd size is an important parameter in this context. A livestock of 30–60 cattle was previously estimated based on excavated remains at Arbon Bleiche 3 [63], which implies an average of one to two co-existing animals per house. Extrapolating the calculated herd size from the excavated area to the whole settlement, we can assume a total number of 60–120 animals. Given individual requirements of approximately 17 ha of land per animal, pasture grounds of 10–20 km^2^ per year were needed to sustain the settlement’s combined herd (SI, section I). Ethnographic studies show that medium-sized cattle herds with more than one animal per house attest to communities that are specialised in animal husbandry or dairying, and which require elaborated herding strategies [64]. Indeed, lipid biomarker evidence on pot sherds from Arbon Bleiche 3 suggests dairying at the settlement [65, 66]. Interestingly, however, not all lactating mother cows seemed to have been kept in the immediate surrounding of Arbon Bleiche 3. Three first molars (ARB 16, 33, 110), representing the time *in utero* and shortly after birth, exhibited ^87^Sr/^86^Sr above the local range (S1 Fig), which point to some dairying in more outlying regions. We argue that the three mobility patterns in the Arbon Bleiche 3 cattle are reflective of different ecological niches utilized to sustain cattle numbers beyond the carrying capacity of the immediate environment of a settlement. This notion is supported by the observed stable carbon isotope ratios, which suggest that the exploited grazing grounds differed in their respective ecological attributes, e.g., with regards to humidity, forest cover or altitude [28, 29]. Although tooth-substance loss primarily relates to the animals’ age, differences in the soil or fodder composition, relating to cattle movement, can also influence distinct tooth-wear patterns (S4 Fig and Table 1) [67, 68]. The observed Sr-isotope pattern variability between the three groups underscores that cattle management in Arbon Bleiche 3 was highly differentiated, emphasizing the complexity of herding strategies.

### Local herding and seasonal movement

The Sr isotope ratios of the individuals representing MP1, accounting for approximately one third of the cattle analysed, are indicative of a static local herd. This group of animals must have been kept in the vicinity of the settlement, not more than a few km away from the site during their enamel mineralization, i.e., the first 24 months. Their C isotope ratios were lowest and most similar to those of red deer (Fig 4), and can best be explained by either leaf and twig foddering in the settlement or feeding in nearby densely forested environments [29]. The observed C isotopic signature would also be consistent with grazing grounds in humid areas [69], e.g., at the shores of Lake Constance or in flood plains. Both ecological niches were readily available in the proximity of the settlement. The absence of any summer-plant remains in cattle dung from the site confirms that there was no year-round husbandry within the actual settlement [62]. Cattle foddering nearby the settlement site implies that local supply of summer browse and winter fodder was sufficient, and that the inevitable disadvantage of losing local fertile land for cattle grazing was compensated for by the obvious benefits of maintaining a local herd (e.g., for milking, slaughtering, and traction purposes). Dairy remains in potsherds [65, 66] as well as the evidence of old animals with traction-related pathologies [40] and a wooden yoke [70] lend clear evidence to the exploitation of these benefits at Arbon Bleiche 3.

In contrast to MP1, the Sr isotope ratios of MP2 individuals suggest periods of both local and non-local grazing. The observed patterns are consistent with a herding management that involves seasonal cattle movement away from the settlement to more Sr-radiogenic catchments, and back. The associated ^13^C/^12^C ratios were higher than for MP1 individuals, consistent with a temporary cattle translocation from the local forested and rather humid environment to less humid landscapes, scattered forests or even open pastures further afield [29]. The co-existence of local cattle (MP1) and cattle displaying isotope patterns of seasonal mobility (MP2) suggests that year-round cattle fodder resources near the site were likely to be fully exploited. That local resources were insufficient to sustain all herded animals is also supported by cattle that carry MP3 (year-round herding away from the site), which represent approximately half of the individuals sampled. MP2 does not necessarily represent long-distance seasonal transhumance, as bio-available Sr with relatively high ^87^Sr/^86^Sr in the same range as observed for cattle teeth occurs within 15 km from the site (S3 Fig). Hence, ^87^Sr/^86^Sr ratios that alternate between local values and values up to 0.7105, e.g., ARB 10, 14, likely reflect periodic mobility in the hilly hinterland of Arbon Bleiche 3. Grazing spots may have varied from one year to the other, but repetitive ^87^Sr/^86^Sr shifts in the composite records suggest that certain pastures were re-visited several times (S1 Fig).

### Non-local herding and alpine transhumance

Alpine mountains, i.e., the Alpstein, are located in a distance of only ~30 km from the site, rendering Arbon Bleiche 3 an ideal environment to conduct alpine transhumance (i.e., the summer exploitation of high-altitude pastures). Botanical remains of stone pine (*Pinus cembra*), alpine speedwell (*Veronica alpina*) and bone finds of the alpine ibex (*Capra ibex*) at the study site indicate that the settlers of Arbon Bleiche 3 visited high-altitude alpine regions between 1500 and 2500 m a.s.l. [16]. Moreover, the remains of Swiss clubmoss (*Selaginella helvetica*), another plant most typical for high-altitude areas (but sporadically found also at lower altitudes and in the region around Lake Constance), were identified in cattle dung [62]. In combination, these findings provide putative evidence for alpine transhumance, exploiting pastures above the timber line (estimated at 1800 m a.s.l. for the mid-4^th^ millennium BC in the Pre-Alps [71]). The Alpstein area itself represents the closest suitable region for transhumant pasturing, but the topography is rather steep and hard to access. Moreover, isotopic values suggest it is unlikely that the Alpstein served as grazing grounds for cattle from Arbon Bleiche 3. The calcareous bedrock is characterized by ^87^Sr/^86^Sr ratios of 0.70812 ± 0.00032 (1σ) (S1 Table), inconsistent with the Sr isotope patterns observed for the MP3 cattle teeth, which are typically more radiogenic, with ^87^Sr/^86^Sr ratios of >0.7120 (ARB 16, 33, 117). Potential grazing spots with ^87^Sr/^86^Sr values of ~0.7120 are located 10–15 km southeast of the site, but they do not exceed an altitude of 1300 m a.s.l. The closest region at higher altitudes and equally high ^87^Sr/^86^Sr ratios (0.71242 ± 0.00086 (1σ)) is located south of Walensee in a distance of ~50 km from Arbon Bleiche 3 (SI, section II). Alternative pasture grounds with similarly old geological units are located east of the Alpine Rhine, slightly further away from the settlement (>60 km). Thus, the Sr isotopic patterns observed for individuals ARB 33 and especially ARB 117 may be best explained by seasonal translocation between different pastures in more Sr-radiogenic catchments, and therefore reflect a mobility pattern that is typical for alpine transhumance [72]. However, we do not see evidence for large-scale alpine cattle migration. The majority of teeth from the MP3 group show Sr isotope ratios that are consistent with grazing grounds away from, but within ~15 km distance of, the settlement site (i.e., ^87^Sr/^86^Sr values of 0.7083–0.7118), with little change in altitude. Moreover, herding grounds and food resources for animals that display MP3 appeared to be distinct from those of the MP2 and MP1 individuals, as indicated by the higher δ^13^C of bone collagen (Fig 4). The higher ^13^C/^12^C ratios may be related to prolonged grazing in open or less humid environments. For comparison, the low δ^13^C values determined for contemporaneous red deer are indicative of feeding grounds with a closed canopy [29]. The observed Sr isotopic fluctuations of MP3 are more complex than for MP2. Some individuals (e.g., ARB 29, 34, 114) suggest periods of stasis followed by periods of enhanced mobility on a timescale that is consistent with differential summer versus winter grazing (SI, section II). In earlier studies, ethnographic records of seasonal mobility provided evidence for complex mobility regimes, likely driven by grazing pressure and climatic constraints. Active cattle management involved multiple (3–8) herd relocations between spring and fall across different altitudes [72, 73]. Additional factors that may have contributed to the unexpectedly high number of MP3 are cattle exchange and the import of animals that had been born before the foundation of Arbon Bleiche 3, e.g., ARB 117 (house 1), ARB 16, 33 (house 3), and ARB 109 (house 14) (S5 Fig). The acquisition of animals from surrounding settlements (cattle exchange), e.g., bulls for breeding, draft animals for labour, or cattle to maintain and increase the herd size, is not unlikely, and social bonds within and beyond settlements are important for minimizing labour costs [64] and forging alliances (e.g., to gain access to land). Ritual activities, feasting, and the symbolic importance of cattle have been documented for the Neolithic and Bronze Age [22, 23, 74], and their relevance may also be indicated by bone middens and bucrania found at Arbon Bleiche 3 [40].

### Specialised herding and differential access to resources

Evidence for specific mobility patterns (MP) appears to be systematically clustered within the settlement (Fig 2). MP1 was primarily observed for individuals found in the northern and central part, evidence for MP2 was focused on the central (and southern) portions of the settlement, and records of MP3 were found all over the settlement area, with a slight predominance in the north. We argue that the MP-clustering attests to differential access to resources by different individuals and groups in the settlement, and to specialised herding strategies to overcome local resource limitations. Local herding (i.e., MP1 and MP2) has likely been organized collectively, with patterns of isotopic variation being sufficiently similar to suggest the use of pastures on similar geological units. Shared herding, certainly the most efficient way of managing cattle, likely required complex interactions, cooperation and networks between the settlers or even between settlements [75–80]. We assume that social alliances were also needed to gain access to specific grazing grounds (e.g., through territorial rights) [81, 82], which apparently differed among the households in Arbon Bleiche 3, possibly depending on the period of occupation or social status. Although patterns of mobility were highly individual among MP3 animals (compared to MP1 and MP2), the range of ^87^Sr/^86^Sr ratios was similar for all cattle during periods of stasis (0.7095–0.7100) (S1 Fig). This suggests perhaps a collective overwintering strategy on the one hand, but the dispersal of animals in summer as small herds with differential access to grazing grounds on the other.

## Conclusions

Our study presents the first large-scale and high-resolution approach using ^87^Sr/^86^Sr LA-MC-ICP-MS measurements to explore Neolithic cattle herding strategies more than 5400 years ago. The data from Arbon Bleiche 3 reveal distinct strontium isotopic patterns that reflect different co-existing herding practices, including localized cattle herding, seasonal mobility and permanent pasturing on distant grounds. Grazing in different environments (either at long- or short-distances) is suggested by the Sr isotope ratios in teeth, dependent on the geology, and supported by the carbon isotope values in bone collagen, dependent on the ecology, and the relation between tooth abrasion and mobility pattern which may relate to the fodder. Exploiting alternative and maybe more distant grazing grounds was necessary to relieve the pressure on local resources (carrying capacity) as a consequence of increased settlement density and larger herd sizes. A limited number of cattle show comparatively high (i.e., radiogenic) ^87^Sr/^86^Sr ratios that are consistent with translocation to, and from, nearby high-altitude areas, and thus reflect vertical (i.e., alpine) transhumance. We cannot exclude, however, that these animals predate the settlement foundation and were brought to Arbon Bleiche 3 by the first generation of settlers. The majority of the transhumant cattle were herded within 15 km of the site. Yet, the Sr isotopic data suggest a high degree of mobility within this perimeter, allowing the exploitation of patchy areas of open pasture. The individual mobility patterns suggest differential access to (presumably) the most favourable grazing grounds, which in turn have contributed to social inequalities between groups or households. But this practise also forged alliances between villages and thus fostered communication and shared values. In consequence, the increasing importance of cattle from the 4^th^ millennium BC onwards, and the emerging complexity in keeping them, may thus be seen as a starting point for further socio-economic differentiation, which was widespread later in the European Bronze Age [83].

## Supporting information

S1 File (PDF)

S1 Fig ^87^Sr/^86^Sr LA-MC-ICP-MS data for individual cattle and analysed teeth (M1–M3), grouped by mobility pattern. Each data point denotes the mean of 10 measurements including a 2σ standard error envelope (given in grey). The coloured bar represents the local ^87^Sr/^86^Sr range (0.7083–0.7091). The complete data set is given in S3 Table.
(PDF)

S2 Fig Comparison of Sr isotope analyses between LA-MC-ICP-MS (solid line) and TIMS (diamonds) of cattle tooth enamel from the Neolithic lakeside settlement of Zurich-Mozartstrasse, Switzerland. LA-MC-ICP-MS data are processed as a 10-point moving mean of the raw signal integrations with the shaded band representing a 2σ standard error of the mean. Micromilled samples (3 mg) were extracted as close as possible to the laser track of the tooth enamel and measured using standard TIMS techniques [48]. Typical errors for TIMS measurements are ± 0.00001. Both methods agree well within the analytical error showing that the potential interferences during LA-MC-ICP-MS measurements have been reduced to insignificant levels.
(TIF)

S3 Fig ^87^Sr/^86^Sr baseline data. We analysed prehistoric fauna and modern local water at Arbon Bleiche 3 (left) and representative environmental ^87^Sr/^86^Sr data for modern vegetation in the nearby and more distant environments (right). Data are also given in S1 and S2 Tables.
(TIF)

S4 Fig Box plot of the tooth enamel length as an indicator of tooth wear. Teeth (M2 = second molar, M3 = third molar) are grouped by mobility pattern. The central black line in each box represents the median, and the cross represents the mean.
(TIF)

S5 Fig Chronological development of houses at Arbon Bleiche 3. Newly built houses are coloured black, existing ones are shown in grey. The last buildings were erected in 3376 BC.
(TIF)

S1 Table Environmental ^87^Sr/^86^Sr baseline data from the surroundings of Arbon Bleiche 3. Calculations on the mean ^87^Sr/^86^Sr and ^87^Sr/^86^Sr ranges for geological units are based on the average values of both plants with shallow (herbs and bushes) and deep (trees) roots from each sampling location. Geological units are colour-coded according to Fig 1.
(PDF)

S2 Table ^87^Sr/^86^Sr baseline data from Arbon Bleiche 3. Prehistoric fauna was analysed by LA-MC-ICP-MS (spot measurements, *Bos taurus* and *Cervus elaphus*) and TIMS (*Sus domesticus*) and modern local water was analysed by TIMS. Water samples were collected in May 2013.
(PDF)

S3 Table ^87^Sr/^86^Sr LA-MC-ICP-MS data for cattle teeth from Arbon Bleiche 3. The length of the laser track (distance from cervix) is shorter than the length of tooth enamel, as given in Table 1. M1 = first molar, M2 = second molar, M3 = third molar.
(PDF)
