# Supplementary material for: High-resolution isotopic evidence of specialised cattle herding in the European Neolithic
Source: PLoS One. 2017 Jul 26;12(7):e0180164. doi: 10.1371/journal.pone.0180164 (PMC5528262; doi:10.1371/journal.pone.0180164)
Supplement: S1 File — (PDF) [file pone.0180164.s001.pdf]

Supporting Information

Section I

Arbon Bleiche 3 – Site characteristics and settlement history

Arbon Bleiche 3, at the southern shores of Lake Constance in northern Switzerland, is one of the most intensively studied wetland sites in the world [1–3], and has provided detailed insights into the socio-economic changes of a Neolithic settlement. The anoxic conditions within the mostly waterlogged archaeological layer were highly conducive to an excellent preservation of wooden remains, enabling the identification of single houses [4]. Between 1993 and 1995, an area of 1100 m^2 was excavated (i.e., 30–50% of the former settlement [1]), exposing the remains of 27 houses. The comparatively large fractional excavation area and the good preservation conditions make Arbon Bleiche 3 an excellent environment in which to study representative patterns and socio-economic strategies at the resolution of both single houses and the whole settlement [5–9].

Dendrochronological dating of the wooden construction elements revealed that the site was occupied for only 15 years, between 3384 and 3370 BC [1]. This corresponds to the early Late Neolithic (according to the Swiss chronology), i.e., to the transition phase from the Pfyn to the Horgen culture, which marks a period of increasing influence from eastern Europe [10] with the introduction of flax, emmer, clay spindle whorls, and the advent of draft-animal use [11]. The first (pioneer) house was built in 3384 BC, followed by the construction of two additional houses in the subsequent year. By 3381 BC the settlement had grown by ten more buildings. One to three additional houses per year were erected until 3376 BC (S5 Fig). In 3370 BC, the settlement was abandoned after a fire event [1]. The excavated houses were rather uniform, with an average size of 4 m x 8 m. Two square buildings of 2 m x 2 m (houses 17 and 25, cf. Fig 2) were exceptions in this regard. Rows of houses were separated by narrow lanes.

Find attribution was performed according to Doppler [12], assuming that archaeological finds of a house reflect the activities and dietary habits of its inhabitants. The analysis of about 70,000 animal bone fragments revealed a bimodal distribution of animal remains within the settlement, suggesting differential dietary habits (mainly beef and littoral fish species in the northern part of the settlement, predominantly pork and limnetic fish in the south [13, 14]. The partitioned animal bone distribution also suggests a certain degree of economic and/or cultural differentiation. Another indication for an economic division of the settlement is given by the uneven distribution of wild and domestic plant species [15, 16]. Different cultural

traditions, or even the presence of culturally distinct groups within the settlement [14], is suggested by the fact that besides local ceramics there is evidence for foreign-style pottery (Baden and Altheim culture), indicating increasing influence from more eastern European regions [17]. It is assumed that people from these regions introduced their technological know-how and integrated with local communities [18].

Estimating land requirements for cattle husbandry

The estimated need for pasture land of 10–20 km^2 for 60–120 cattle, respectively, is based on ethnographic data [19]: mobile cattle husbandry requires grazing areas of 4–14 ha per year for animals in deciduous forests and 8–20 ha in coniferous woodlands depending on the vertical and horizontal structure of the landscape. In the mosaic-like landscape with both deciduous and coniferous woodland as evidenced for Arbon Bleiche 3 [11], it can be assumed that one cattle needs 6–17 ha per year. We consider the mean upper end of estimates for both forest types (17 ha) to be more realistic since the Neolithic landscape was rather densely forested with only small patches of grassland suited for grazing. Although the consideration of these maxima is a hypothetical approximation, it stands in good agreement with observations in subalpine regions [20], where one cattle needed a minimum feeding ground of 5 ha during a season (3–4 months) to avoid damage to trees and allow the animal to select the best locations and plants. This considerable amount of land requirement may explain why people in Arbon Bleiche 3 had to exploit diverse grazing grounds in order to respect the carrying capacity of their environment.

Section II

Local $^{87}Sr/^{86}Sr$ signatures

Since the choice of the most suitable baseline sample for characterizing the local biologically available Sr is a matter of discussion [21–23], we use a mixture of different materials: the enamel of locally living animals (pigs), the water of potential drinking water sources, and (diagenetically altered) dentine. After burial, dentine (in contrast to tooth enamel) is highly susceptible to Sr isotope exchange with water in the soils. It thus reflects a mixture of biogenic and diagenetic strontium due to diagenesis [24, 25] and can therefore be used as a proxy of the local $^{87}Sr/^{86}Sr$ baseline signature. The average $^{87}Sr/^{86}Sr$ of biologically available Sr at Arbon Bleiche 3 based on dentine from cattle (*Bos taurus*) and red deer (*Cervus elaphus*), as well as on tooth enamel from pigs (*Sus domesticus*), were in good agreement with modern Sr isotopic water signatures determined for nearby water sources, such as the

river Aach and Lake Constance (S2 Table). The cattle dentine yielded a mean $^{87}Sr/^{86}Sr$ of 0.70880 ± 0.00037 (2σ, n = 12), while the mean $^{87}Sr/^{86}Sr$ of the red deer dentine was 0.70859 ± 0.00007 (2σ, n = 4). The pig tooth enamel samples averaged at 0.70856 ± 0.00035 (2σ, n = 7) and the water showed $^{87}Sr/^{86}Sr$ values of 0.70842 and 0.70852 (n = 2). Based on the mean $^{87}Sr/^{86}Sr$ of all reference samples (0.70867 ± 0.00040; 2σ, n = 25), a range of 0.70827 to 0.70907 was considered to reflect the local baseline (S3 Fig).

^{87}Sr/^{86}Sr signatures in the region

28 locations were sampled to determine the isotopic signature of biologically available Sr from potential grazing grounds in the surrounding area of Arbon Bleiche 3 (Fig 1 and S1 Table). To this end, plants with shallow roots (herbs or bushes) and plants with deep roots (trees) were sampled in duplicates from each location, and the mean $^{87}Sr/^{86}Sr$ of biologically available Sr was determined.

Around Arbon Bleiche 3, modern vegetation samples (from the Quaternary moraines deposited during the Würm glacial and Upper Freshwater molasses formed in the Tertiary) yielded mean $^{87}Sr/^{86}Sr$ of 0.70945 ± 0.00073 (1σ, n = 5) and 0.70871 ± 0.00039 (1σ, n = 4) respectively and ranged from 0.70828 to 0.71030. This range is in good agreement with published modern environmental $^{87}Sr/^{86}Sr$ reference data (fauna and vegetation) for the equivalent geological formations from the northern shores of Lake Constance, Germany, with average $^{87}Sr/^{86}Sr$ ratios between 0.70861 ± 0.00061 and 0.70982 ± 0.00022, respectively [26]. We assume that observed values within this range are characteristic for cattle browsing and grazing nearby Arbon Bleiche 3 (<5 km; S3 Fig).

A little further away from Arbon Bleiche 3 (within a perimeter of 15–20 km), Quaternary deposits from the Riss glaciation, from Tertiary Upper Marine molasses and from Tertiary Lower Freshwater molasses are outcropping. In agreement with data from a neighbouring region [26], the determined unit-specific $^{87}Sr/^{86}Sr$ values were 0.71045 (n=1) (vegetation from the Riss moraines), 0.70895 ± 0.00070 (1σ, n = 2) (vegetation from the Upper Marine molasse), and 0.71048 ± 0.00118 (1σ, n = 6) (vegetation from Lower Freshwater molasse). We assume $^{87}Sr/^{86}Sr$ between 0.70828 and 0.71181 to be representative for cattle grazing and browsing in the wider environs of Arbon Bleiche 3 (S3 Fig; 5–15 km).

Based on archaeobotanical and archaeozoological data, it was suggested that the inhabitants of Arbon Bleiche 3 occasionally visited the mountain ranges in the Alpstein and Walensee region, at a distance of approximately 30 km [11]. The Alpstein region is predominated by calcareous bedrock, and modern vegetation samples from this area (n = 5) exhibited

characteristically low $^{87}Sr/^{86}Sr$ values between 0.70772 and 0.70849 with an average of 0.70812 ± 0.00032 (1σ). Despite some degree of overlap with more local Sr isotope signatures, these mountainous regions are isotopically distinguishable from the immediate hinterland of the site. Finally, the area south of Walensee, located ~50 km away, is characterized by Permian geological units. Here vegetation samples (n = 2) yielded $^{87}Sr/^{86}Sr$ values that were significantly higher than those observed for vegetation in the vicinity of the settlement, with an average of 0.71242 ± 0.00086 (1σ, S3 Fig).

Identifying seasonal migration based on strontium isotope analysis

Davies recorded movements of livestock from seven Alpine settlements, and while the movement regimes observed in this study are somewhat different for each livestock, they all share a common pattern of ~7 months of stasis during the cold season [27]. These periods of movement and stasis can be identified using strontium isotope analyses by high-resolution LA-MC-ICP-MS and linked to published rates of enamel formation [28, 29]. Seven months represent about 60% of the mineralization time of cattle M2, and about 50% of M3. Although we acknowledge that deviation from this may result from a non-constant rate of enamel growth [30], or from herding practices that significantly deviate from Davies' observations [27], we find that the majority (80%) of cattle that display mobility pattern 2 (ARB 10, 14, 22, 119) and 50% of the cattle representing mobility pattern 3 (ARB 16, 23, 33, 24, 114) show relatively invariant (and low) $^{87}Sr/^{86}Sr$ values over a mineralization time that is consistent with about this length of stasis. While we cannot be completely certain with regards to the exact timing of these periods of stasis, it is reasonable to assume that they relate to foddering practices, which were likely to be highly seasonal in this sub-alpine region.

References

1. Leuzinger U. Die jungsteinzeitliche Seeufersiedlung Arbon-Bleiche 3: Befunde. Frauenfeld: Huber & Co. AG; 2000.
2. De Capitani A, Deschler-Erb S, Leuzinger U, Marti-Grädel E, Schibler J, editors. Die jungsteinzeitliche Seeufersiedlung Arbon Bleiche 3: Funde. Frauenfeld: Huber & Co. AG; 2002.
3. Jacomet S, Leuzinger U, Schibler J, editors. Die jungsteinzeitliche Seeufersiedlung Arbon Bleiche 3: Umwelt und Wirtschaft. Frauenfeld: Huber & Co. AG; 2004.
4. Leuzinger U, Jacomet S. Einleitung. In: Jacomet S, Leuzinger U, Schibler J, editors. Die jungsteinzeitliche Seeufersiedlung Arbon-Bleiche 3: Umwelt und Wirtschaft. Frauenfeld: Huber & Co. AG; 2004. p. 25-39.
5. Brombacher C, Hadorn P. Untersuchungen der Pollen und Makroreste aus den Profilsäulen. In: Jacomet S, Leuzinger U, Schibler J, editors. Die jungsteinzeitliche

Seeufersiedlung Arbon-Bleiche 3: Umwelt und Wirtschaft. Frauenfeld: Huber & Co. AG; 2004. p. 50-65.

6. Deschler-Erb S, Marti-Grädel E. Hinweise zur Schichterhaltung aufgrund der Tierknochen. In: Jacomet S, Leuzinger U, Schibler J, editors. Die jungsteinzeitliche Seeufersiedlung Arbon-Bleiche 3: Umwelt und Wirtschaft. Frauenfeld: Huber & Co. AG; 2004. p. 90-100.
7. Haas JN, Magny M. Schichtgenese und Vegetationsgeschichte. In: Jacomet S, Leuzinger U, Schibler J, editors. Die jungsteinzeitliche Seeufersiedlung Arbon-Bleiche 3: Umwelt und Wirtschaft. Frauenfeld: Huber & Co. AG; 2004. p. 43-49.
8. Ismail-Meyer K, Rentzel P. Mikromorphologische Untersuchung der Schichtabfolge. In: Jacomet S, Leuzinger U, Schibler J, editors. Die jungsteinzeitliche Seeufersiedlung Arbon-Bleiche 3: Umwelt und Wirtschaft. Frauenfeld: Huber & Co. AG; 2004. p. 66-80.
9. Thew N. The Aquatic and terrestrial molluscs from the profile columns. In: Jacomet S, Leuzinger U, Schibler J, editors. Die jungsteinzeitliche Seeufersiedlung Arbon-Bleiche 3: Umwelt und Wirtschaft. Frauenfeld: Huber & Co. AG; 2004. p. 81-89.
10. Köninger J, Kolb M, Schlichtherle H. Elemente von Boleráz und Baden in den Feuchtbodensiedlungen des südwestdeutschen Alpenvorlandes und ihre mögliche Rolle im Transformationsprozess des lokalen Endneolithikums. In: Roman P, Diamandi S, editors. Cernavodă III – Boleráz. Ein vorgeschichtliches Phänomen zwischen dem Oberrhein und der unteren Donau. Bucureşti: Institutul Român de Tracologie; 2001. p. 641-673.
11. Jacomet S, Leuzinger U, Schibler J. Synthesis. In: Jacomet S, Leuzinger U, Schibler J, editors. Die jungsteinzeitliche Seeufersiedlung Arbon-Bleiche 3: Umwelt und Wirtschaft. Frauenfeld: Huber & Co. AG; 2004. p. 379-416.
12. Doppler T. Archäozoologie als Zugang zur Sozialgeschichte in der Feuchtbodenarchäologie. Forschungsperspektiven am Fallbeispiel der neolithischen Seeufersiedlung Arbon Bleiche 3 (Schweiz) [PhD Thesis]. University of Basel; 2013. Available from: doi 10.5451/unibas-006089936.
13. Hüster Plogmann H. Fischfang und Kleintierbeute. Ergebnisse der Untersuchung von Tierresten aus den Schlämmproben. In: Jacomet S, Leuzinger U, Schibler J, editors. Die jungsteinzeitliche Seeufersiedlung Arbon-Bleiche 3: Umwelt und Wirtschaft. Frauenfeld: Huber & Co. AG; 2004. p. 253-276.
14. Marti-Grädel E, Deschler-Erb S, Hüster-Plogmann H, Schibler J. Early evidence of economic specialization or social differentiation: a case study from the Neolithic lake shore settlement 'Arbon-Bleiche 3' (Switzerland). In: Jones O'Day S, van Neer W, Ervynck A, editors. Behaviour Behind Bones. The zooarchaeology of ritual, religion, status and identity. Proceedings of the 9th ICAZ Conference, Durham 2002. Oxford: Oxbow Books; 2004. p. 164-176.
15. Hosch S, Jacomet S. Ackerbau und Sammelwirtschaft. Ergebnisse der Untersuchung von Samen und Früchten. In: Jacomet S, Leuzinger U, Schibler J, editors. Die jungsteinzeitliche Seeufersiedlung Arbon-Bleiche 3: Umwelt und Wirtschaft. Frauenfeld: Huber & Co. AG; 2004. p. 112-157.
16. Doppler T, Pollmann B, Röder B. Considerations about possible household activities in the Neolithic lakeside settlement Arbon Bleiche 3, Switzerland – a preliminary approach. In: Madella M, Kovács G, Kulcsarne-Berzsenyi B, Briz i Godino I, editors. The Archaeology of Household. Oxford: Oxbow Books; 2013. p. 118-130.

17. De Capitani A. Gefässkeramik. In: De Capitani A, Deschler-Erb S, Leuzinger U, Marti-Grädel E, Schibler J, editors. Die jungsteinzeitliche Seeufersiedlung Arbon Bleiche 3: Funde. Frauenfeld: Huber & Co. AG; 2002. p. 135-276.

18. Bonzon J. Archaeometrical study (petrography, mineralogy and chemistry) of the ceramics. In: Jacomet S, Leuzinger U, Schibler J, editors. Die jungsteinzeitliche Seeufersiedlung Arbon-Bleiche 3: Umwelt und Wirtschaft. Frauenfeld: Huber & Co. AG; 2004. p. 294-312.

19. Ebersbach R. Glückliche Milch von glücklichen Kühen? Zur Bedeutung der Rinderhaltung in (neolithischen) Wirtschaftssystemen. In: Herrmann B, editor. Beiträge zum Göttinger Umwelthistorischen Kolloquium 2004-2006. Göttingen: Universitätsverlag Göttingen; 2007. p. 41-58.

20. Mayer AC, Stöckli V, Gotsch N, Konold W, Kreuzer M. Waldweide im Alpenraum. Neubewertung einer traditionellen Mehrfachnutzung. Schweiz Z Forstwesen 2004;155(2): 38-44.

21. Maurer A-F, Galer SJG, Knipper C, Beierlein L, Nunn EV, Peters D, et al. Bioavailable $^{87}Sr/^{86}Sr$ in different environmental samples – Effects of anthropogenic contamination and implications for isoscapes in past migration studies. Sci Total Environ 2012;433: 216-229.

22. Price TD, Burton JH, Bentley RA. The characterization of biologically available strontium isotope ratios for the study of prehistoric migration. Archaeometry 2002;44(1): 117-135.

23. Bentley RA. Strontium Isotopes from the Earth to the Skeleton: A review. J Archaeol Method Th 2006;13(3): 135-187.

24. Hoppe KA, Koch PL, Furutani TT. Assessing the preservation of biogenic strontium in fossil bones and tooth enamel. Int J Osteoarchaeol 2003;13(1/2): 20-28.

25. Trickett MA, Budd P, Montgomery J, Evans J. An assessment of solubility profiling as a decontamination procedure for the Sr-87/Sr-86 analysis of archaeological human skeletal tissue. Appl Geochem 2003;18(5): 653-658.

26. Oelze VM, Nehlich O, Richards MP. ‘There is no place like home’ – no isotopic evidence for mobility at the early Bronze Age cemetery of Singen, Germany. Archaeometry 2012;54(4): 752-778.

27. Davies E. The patterns of transhumance in Europe. Geography 1941;26(4): 155-168.

28. Beasley M, Brown W, Legge A. Incremental banding in dental cementum: methods of preparation for teeth from archaeological sites and for modern comparative specimens. Int J Osteoarchaeol 1992;2(1): 37-50.

29. Brown W, Christofferson P, Massler M, Weiss M. Postnatal tooth development in cattle. Am J Vet Res 1960;21: 7-34.

30. Bendrey R, Vella D, Zazzo A, Balasse M, Lepetz S. Exponentially decreasing tooth growth rate in horse teeth: implications for isotopic analyses. Archaeometry 2015;57(6): 1104-1124.
