# Supplementary material for: High-resolution isotopic evidence of specialised cattle herding in the European Neolithic
Source: PLoS One. 2017 Jul 26;12(7):e0180164. doi: 10.1371/journal.pone.0180164 (PMC5528262; doi:10.1371/journal.pone.0180164)

Mobility pattern 1

ARB 2.2.1 (M2)

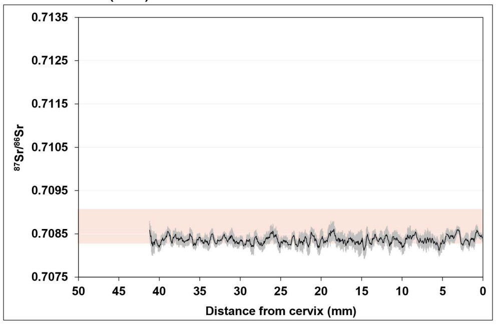

ARB 2.3.1 (M3)

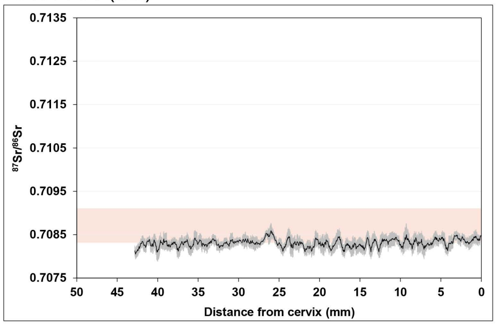

ARB 19.2.1 (M3)

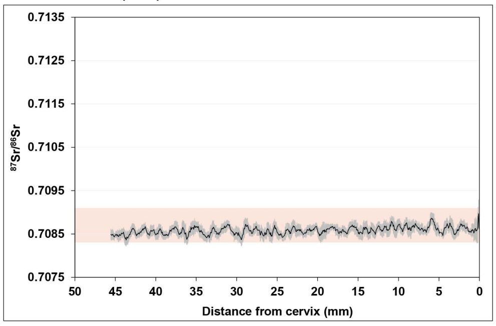

ARB 113.2.1 (M2)

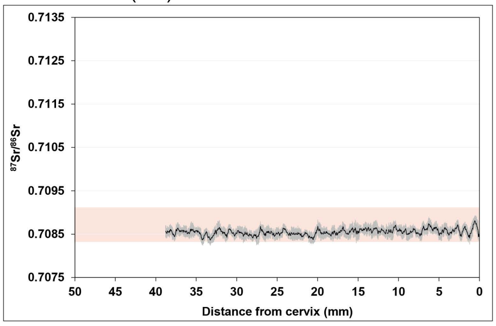

ARB 113.3.1 (M3)

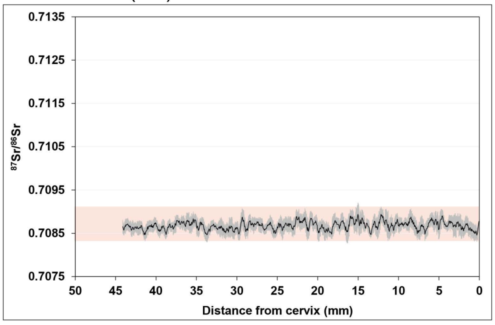

ARB 118.2.1 (M2)

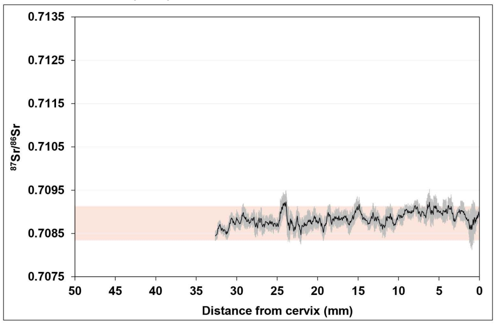

ARB 118.3.1 (M3)

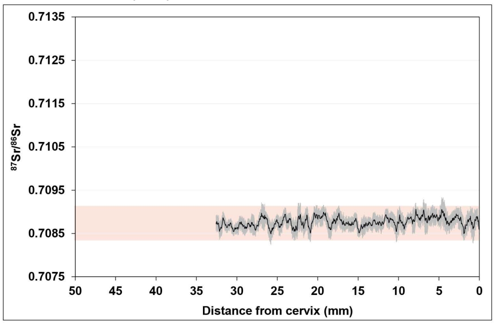

ARB 25.2.1 (M3)

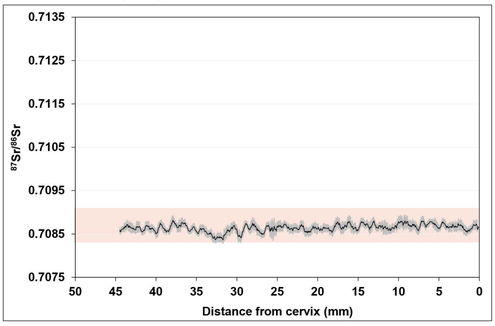

ARB 26.2.1 (M2)

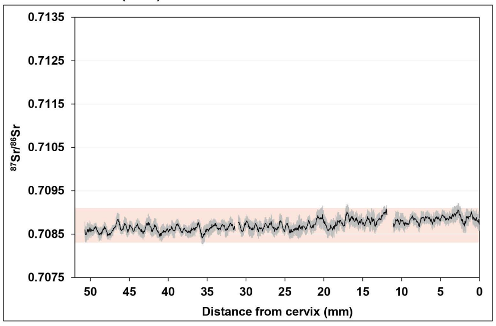

Mobility pattern 1

ARB 43.2.1 (M2)

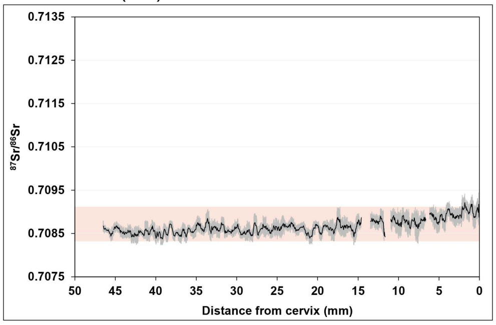

ARB 112.2.1 (M2)

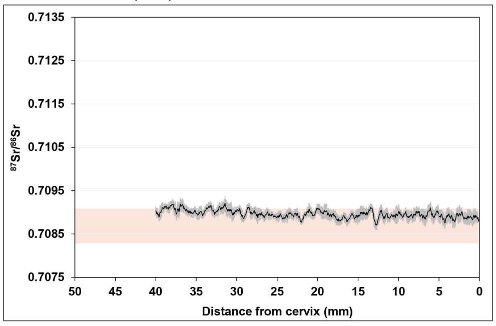

ARB 112.3.1 (M3)

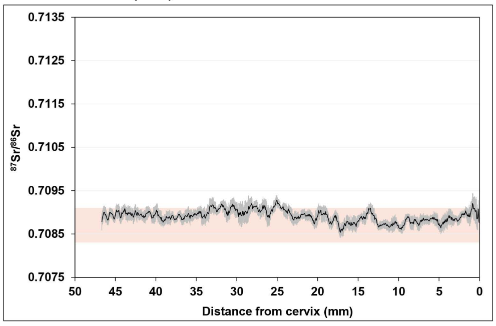

Mobility pattern 2

ARB 10.2.1 (M2)

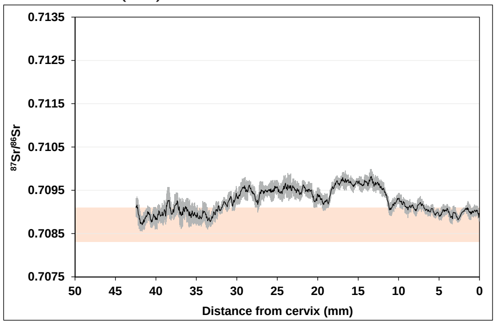

ARB 14.2.1 (M2)

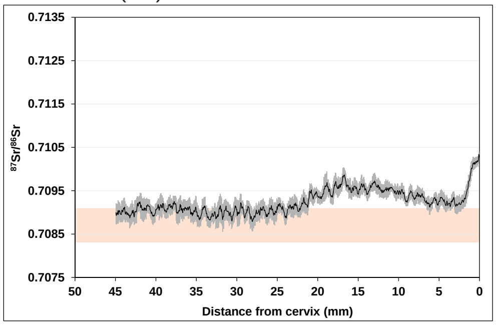

ARB 14.3.1 (M3)

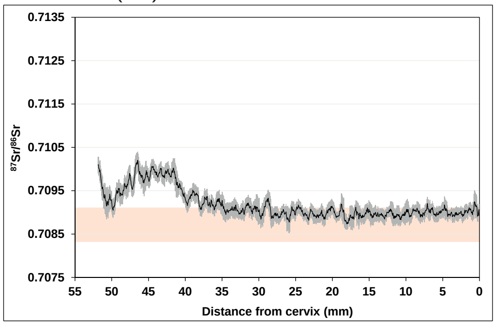

ARB 109.2.1 (M2)

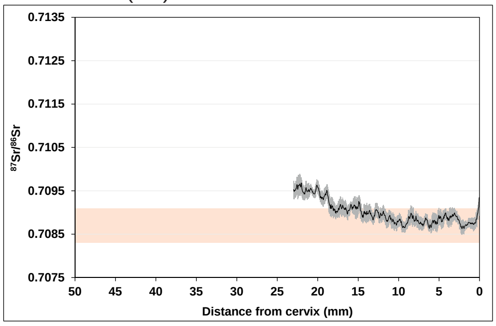

ARB 22.2.1 (M2)

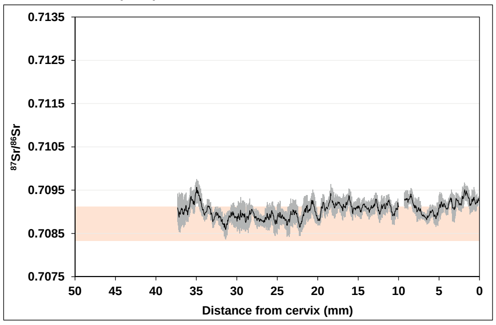

ARB 119.2.1 (M2)

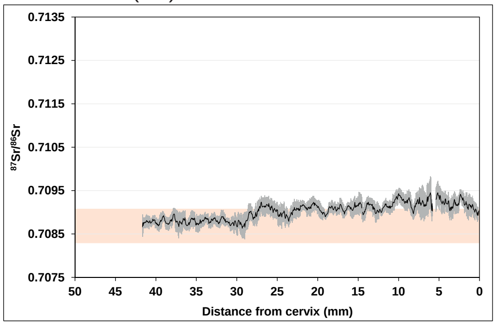

ARB 119.3.1 (M3)

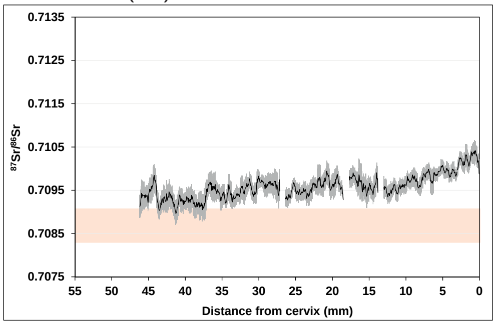

Mobility pattern 3

ARB 117.2.1 (M2)

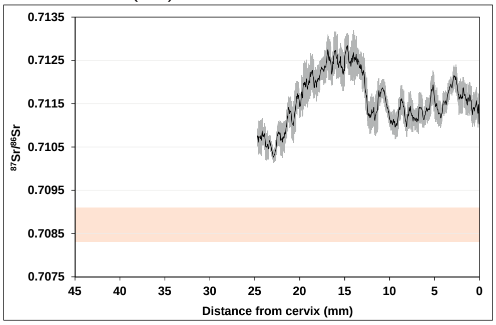

ARB 117.3.1 (M3)

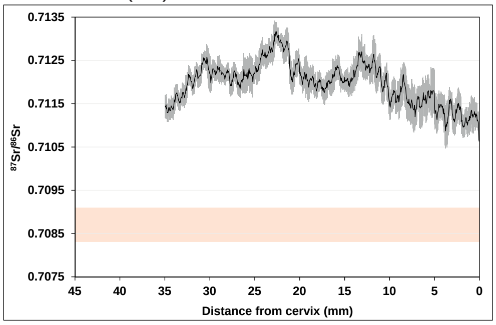

ARB 115.2.1 (M2)

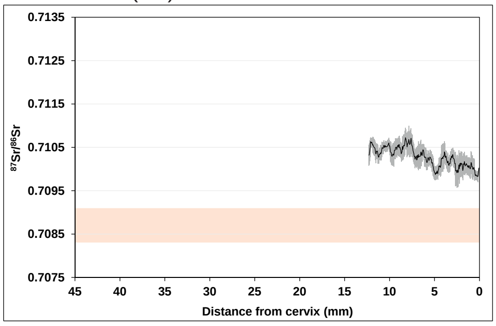

ARB 115.3.1 (M3)

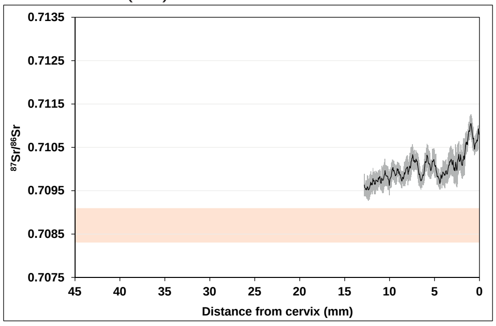

ARB 16.3.1 (M1)

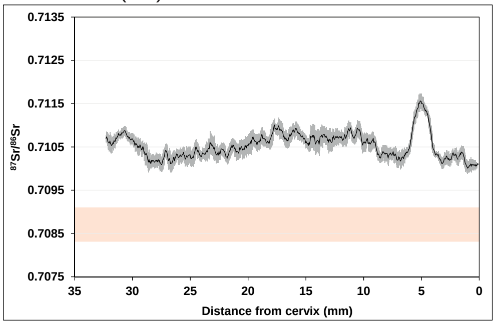

ARB 16.2.1 (M2)

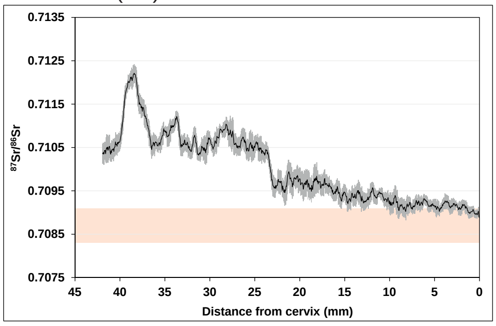

ARB 23.2.1 (M2)

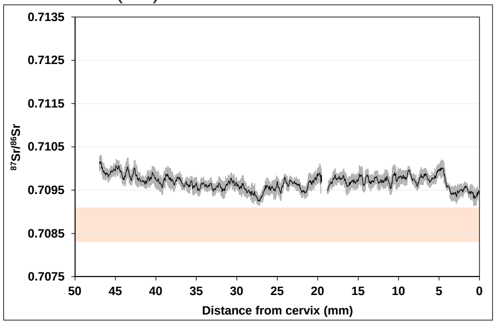

ARB 110.3.1 (M1)

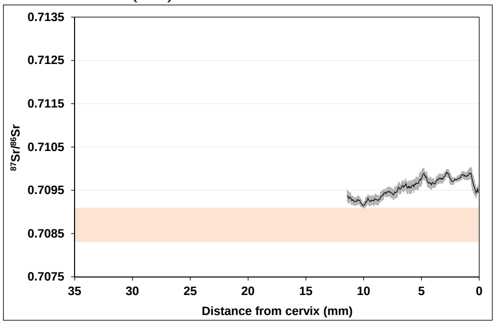

ARB 110.2.1 (M2)

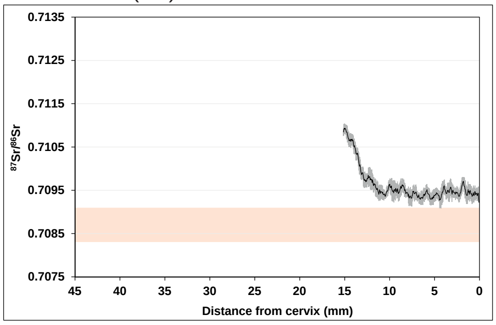

ARB 110.4.1 (M3)

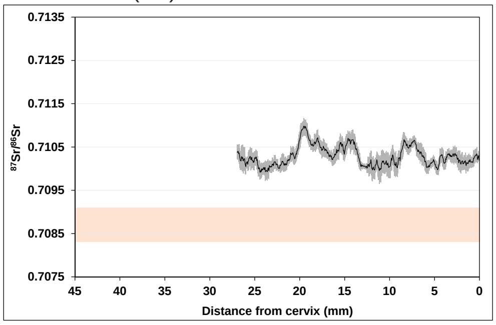

ARB 111.2.1 (M2)

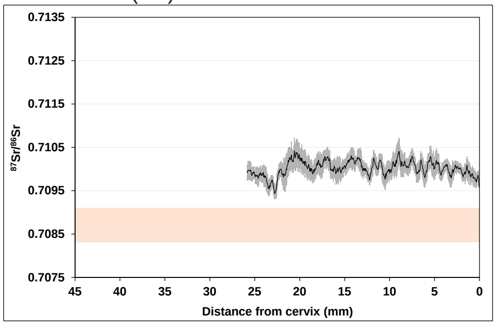

Mobility pattern 3

ARB 27.2.1 (M2)

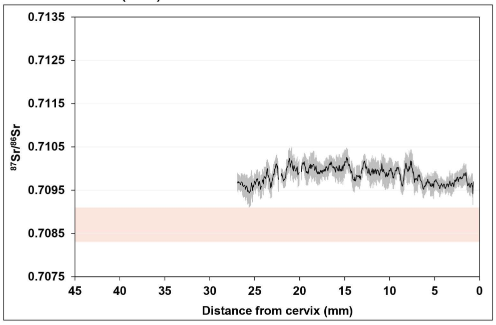

ARB 29.2.1 (M2)

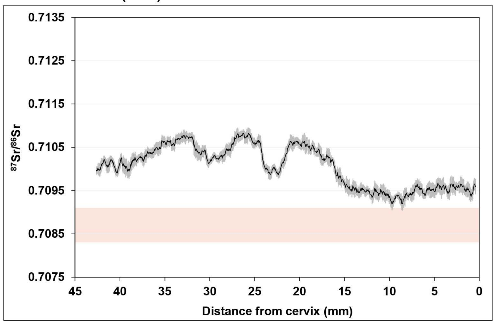

ARB 33.4.1 (M1)

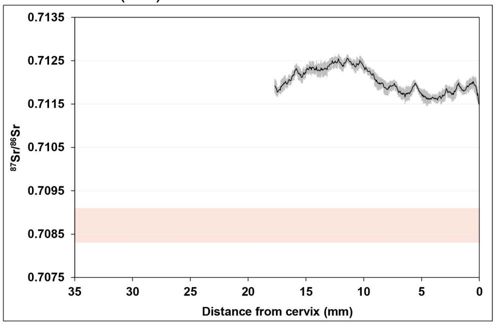

ARB 33.2.1 (M2)

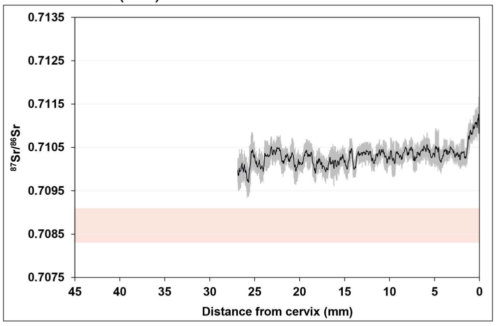

ARB 33.3.1 (M3)

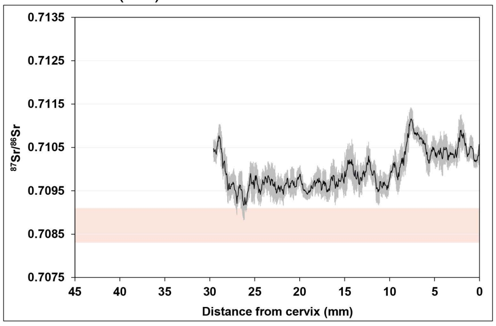

ARB 34.2.1 (M2)

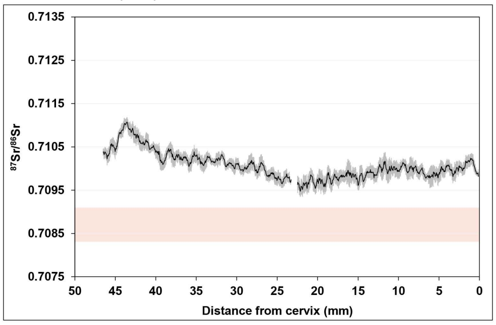

ARB 116.2.1 (M2)

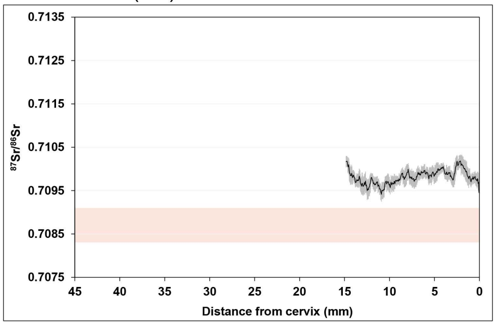

ARB 114.2.1 (M2)

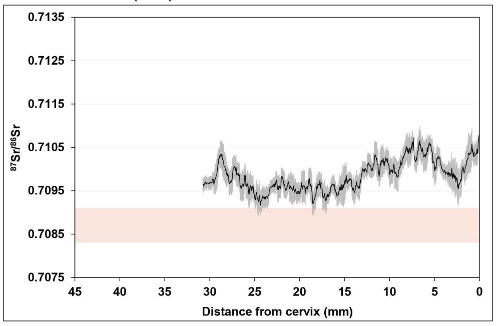

ARB 114.3.1 (M3)

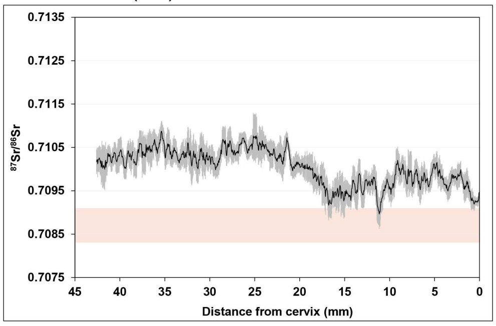

Supplement: S1 Fig — Each data point denotes the mean of 10 measurements including a 2σ standard error envelope (given in grey). The coloured bar represents the local 87Sr/86Sr range (0.7083–0.7091). The complete data set is given in S3 Table. (PDF) [file pone.0180164.s002.pdf]
