# Supplementary figures and images for: High-resolution isotopic evidence of specialised cattle herding in the European Neolithic
Source: PLoS One. 2017 Jul 26;12(7):e0180164. doi: 10.1371/journal.pone.0180164 (PMC5528262; doi:10.1371/journal.pone.0180164)

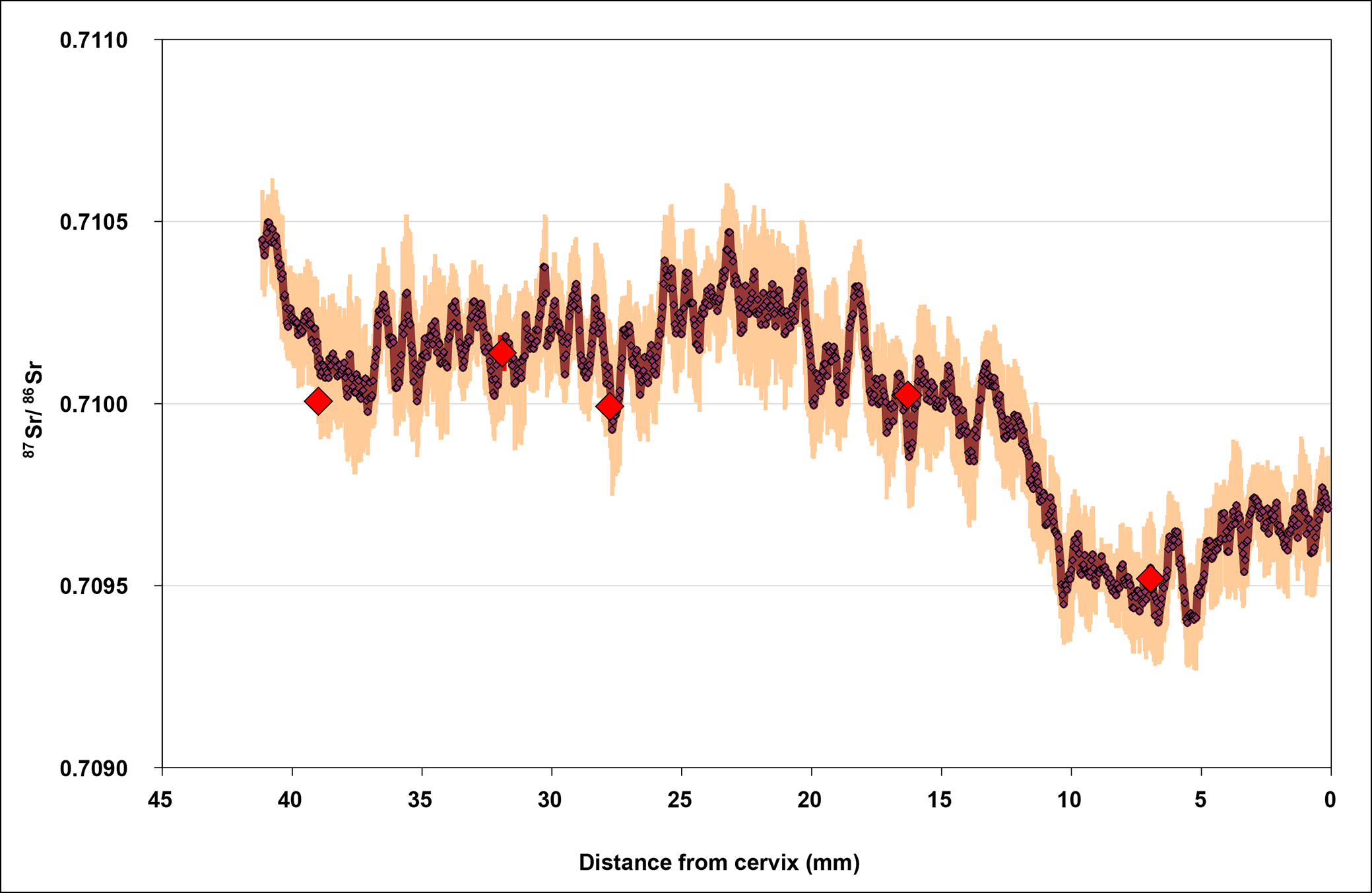

Supplement: S2 Fig — LA-MC-ICP-MS data are processed as a 10-point moving mean of the raw signal integrations with the shaded band representing a 2σ standard error of the mean. Micromilled samples (3 mg) were extracted as close as possible to the laser track of the tooth enamel and measured using standard TIMS techniques [48]. Typical errors for TIMS measurements are ± 0.00001. Both methods agree well within the analytical error showing that the potential interferences during LA-MC-ICP-MS measurements have been reduced to insignificant levels. (TIF) [file pone.0180164.s003.tif]

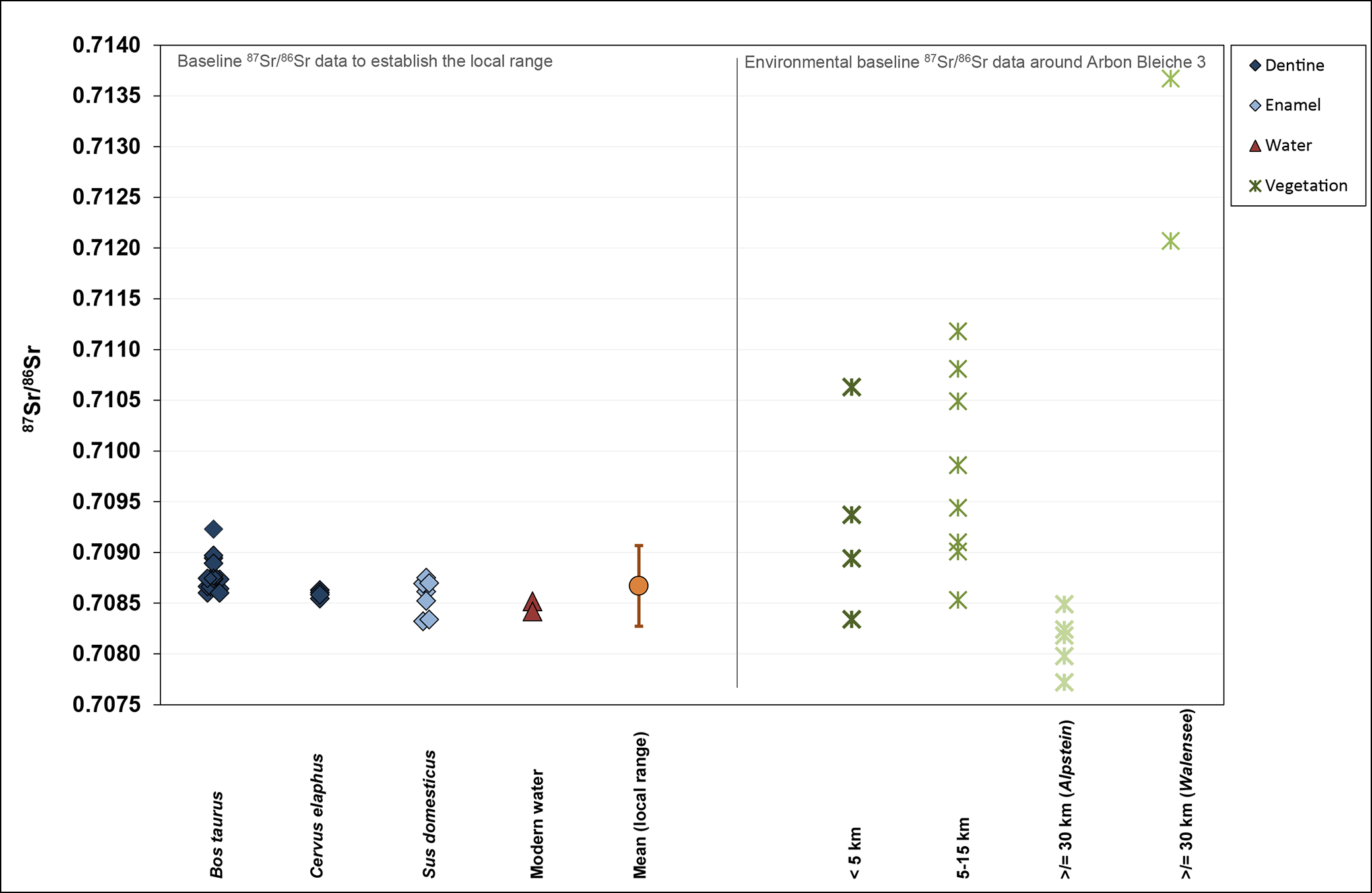

Supplement: S3 Fig — We analysed prehistoric fauna and modern local water at Arbon Bleiche 3 (left) and representative environmental 87Sr/86Sr data for modern vegetation in the nearby and more distant environments (right). Data are also given in S1 and S2 Tables. (TIF) [file pone.0180164.s004.tif]

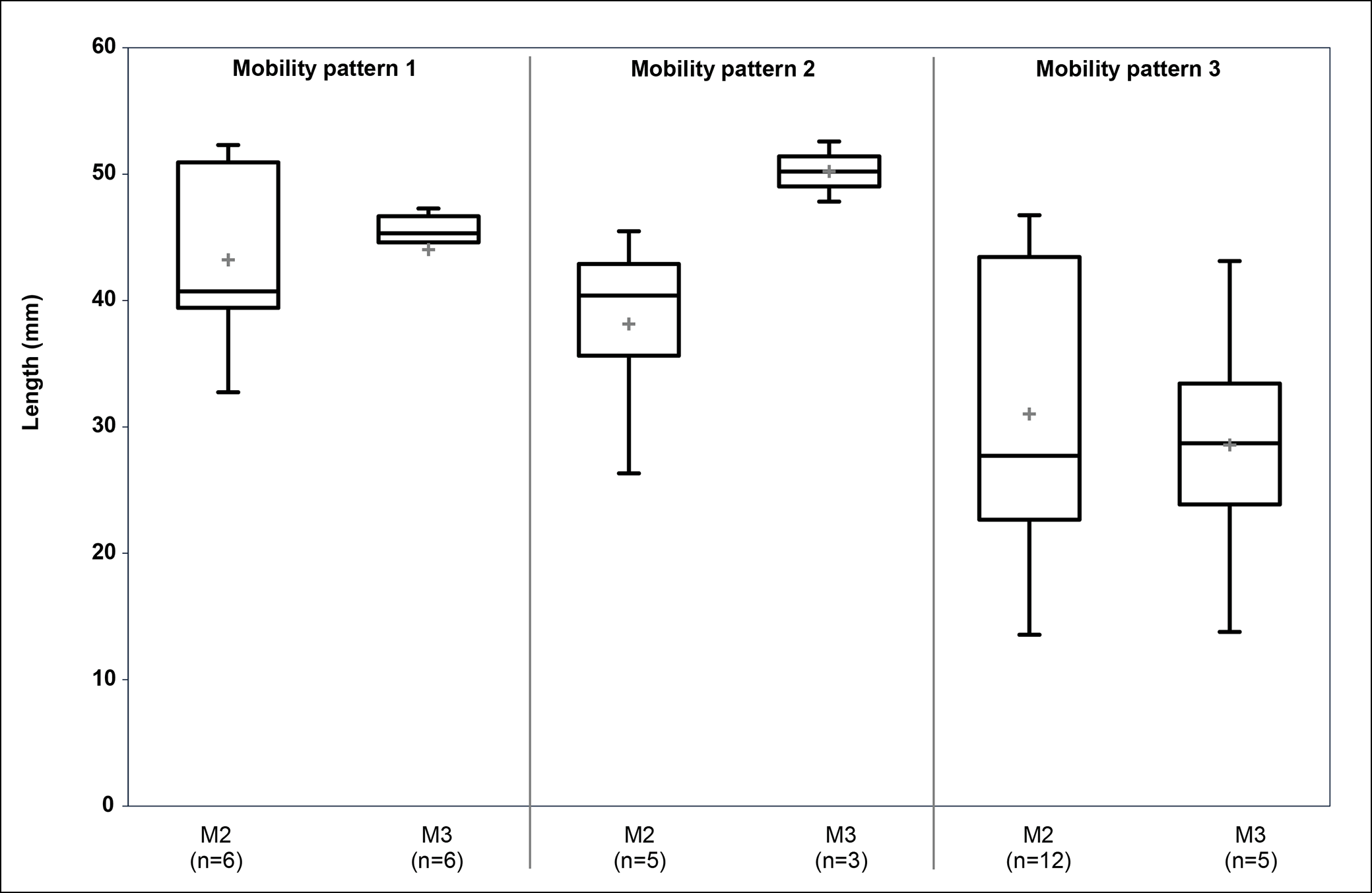

Supplement: S4 Fig — Teeth (M2 = second molar, M3 = third molar) are grouped by mobility pattern. The central black line in each box represents the median, and the cross represents the mean. (TIF) [file pone.0180164.s005.tif]

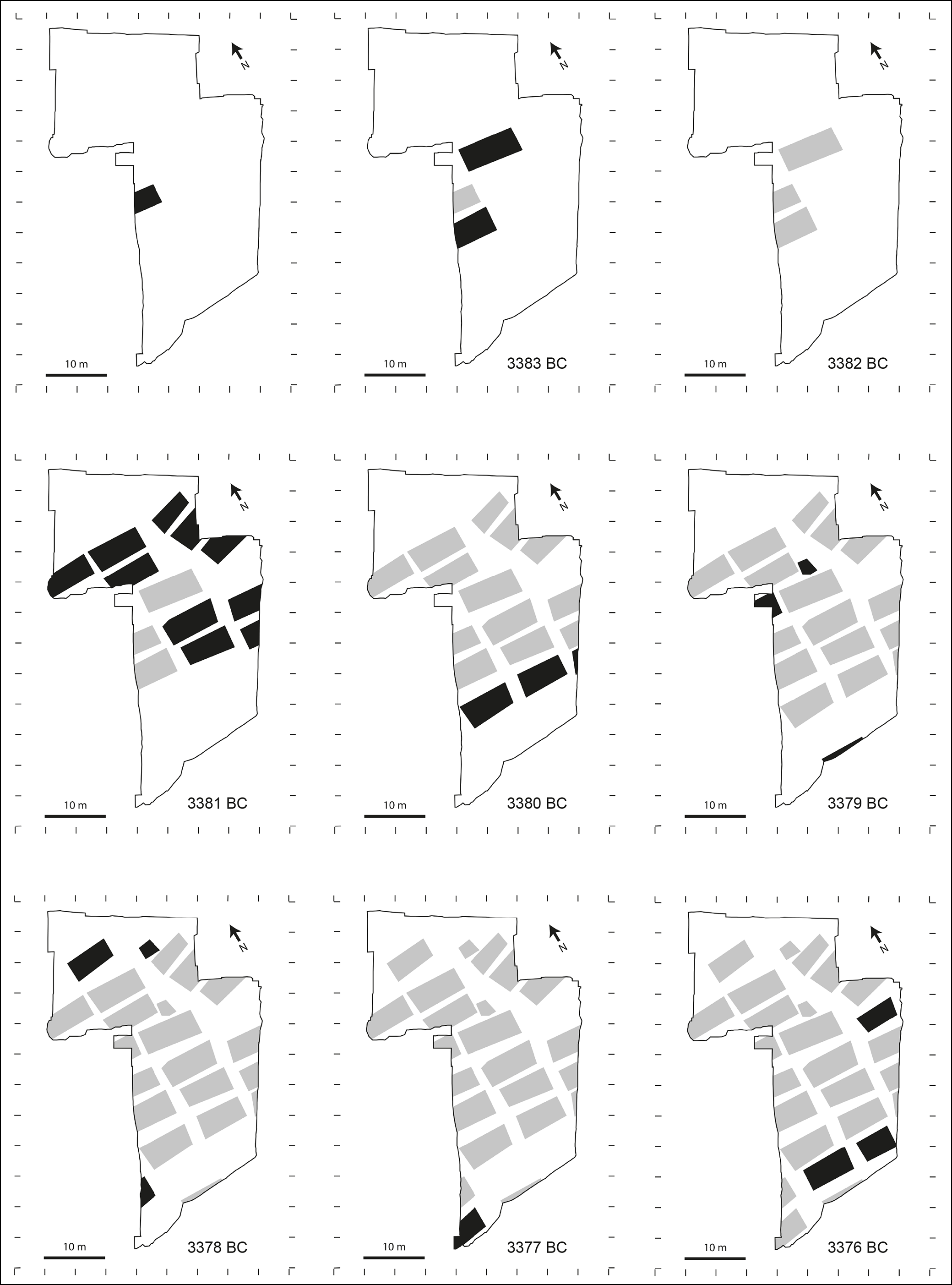

Supplement: S5 Fig — Newly built houses are coloured black, existing ones are shown in grey. The last buildings were erected in 3376 BC. (TIF) [file pone.0180164.s006.tif]
