# Supplementary material for: High-resolution isotopic evidence of specialised cattle herding in the European Neolithic
Source: PLoS One. 2017 Jul 26;12(7):e0180164. doi: 10.1371/journal.pone.0180164 (PMC5528262; doi:10.1371/journal.pone.0180164)
Supplement: S1 Table — Calculations on the mean 87Sr/86Sr and 87Sr/86Sr ranges for geological units are based on the average values of both plants with shallow (herbs and bushes) and deep (trees) roots from each sampling location. Geological units are colour-coded according to Fig 1. (PDF) [file pone.0180164.s007.pdf]

Label	Lab ID	Latitude	Longitude	Geology			Herb/bush	$^{87}Sr/^{86}Sr$	±2σ	Tree	$^{87}Sr/^{86}Sr$	±2σ	$^{87}Sr/^{86}Sr$ Mean	SD	$^{87}Sr/^{86}Sr$ Mean geology	SD	Range geology
q	74	47.35640	9.59135	Quaternary	Alluvial sediments	mainly peat	Fern (*Polypodiopsida sp.*)	0.70878	0.00001	Maple (*Acer pseudoplatanus*)	0.70873	0.00001	0.70876	0.00004	0.70871	0.00007	0.70866-0.70876
p	75	47.33480	9.57139	Quaternary	Alluvial sediments	mainly clay	Impatiens (*Impatiens*)	0.70853	0.00001	Birch (*Betula sp.*)	0.70879	0.00001	0.70866	0.00018			
c	3	47.49678	9.39041	Quaternary	Moraine	Würm glacial	Common wood sorrel (*Oxalis acetosella*)	0.70997	0.00001	Beech (*Fagus sylvatica*)	0.71063	0.00001	0.71030	0.00047	0.70945	0.00073	0.70861-0.71030
b	9	47.54771	9.31660	Quaternary	Moraine	Würm glacial	Raspberry (*Rubus idaeus*)	0.70886	0.00001	Beech (*Fagus sylvatica*)	0.70901	0.00025	0.70894	0.00011			
d	11	47.49454	9.40701	Quaternary	Moraine	Würm glacial	Raspberry (*Rubus idaeus*)	0.70922	0.00001	Beech (*Fagus sylvatica*)	0.70937	0.00001	0.70930	0.00011			
a	69	47.46836	9.17117	Quaternary	Moraine	Würm glacial	Woodruff (*Galium odoratum*)	0.70821	0.00001	Beech (*Fagus sylvatica*)	0.70901	0.00001	0.70861	0.00057			
o	76	47.41943	9.53684	Quaternary	Moraine	Würm glacial	Blackberry (*Rubus fruticosus*)	0.70936	0.00001	Beech (*Fagus sylvatica*)	0.71081	0.00001	0.71009	0.00103			
m	71	47.43396	9.49674	Quaternary	Moraine	Riss glacial	Common wood sorrel (*Oxalis acetosella*)	0.71040	0.00020	Beech (*Fagus sylvatica*)	0.71049	0.00001	0.71045	0.00006			0.71045
f	6	47.47931	9.41532	Tertiary	Upper Freshwater molasse	*Sarmatien*	Impatiens (*Impatiens*)	0.70822	0.00001	Beech (*Fagus sylvatica*)	0.70834	0.00001	0.70828	0.00008	0.70871	0.00039	0.70828-0.70911
e	12	47.49410	9.41022	Tertiary	Upper Freshwater molasse	*Sarmatien*	Ivy (*Hedera helix*)	0.70897	0.00001	Beech (*Fagus sylvatica*)	0.70894	0.00001	0.70896	0.00002			
h	19	47.45229	9.39412	Tertiary	Upper Freshwater molasse	*Tortonien*	Blackberry (*Rubus fruticosus*)	0.70844	0.00001	Beech (*Fagus sylvatica*)	0.70853	0.00001	0.70849	0.00006			
g	20	47.45288	9.38949	Tertiary	Upper Freshwater molasse	*Sarmatien*	Raspberry (*Rubus idaeus*)	0.70911	0.00001	Beech (*Fagus sylvatica*)	0.70910	0.00001	0.70911	0.00001			
n	13	47.47161	9.54180	Tertiary	Upper Seawater molasse	*Burdigalien, marin*	Woodruff (*Galium odoratum*)	0.70944	0.00001	Beech (*Fagus sylvatica*)	0.70944	0.00001	0.70944	0.00000	0.70895	0.00070	0.70845-0.70944
k	77	47.36455	9.48555	Tertiary	Upper Seawater molasse	*Burdigalien, limnisch*	Blackberry (*Rubus fruticosus*)	0.70833	0.00001	Beech (*Fagus sylvatica*)	0.70858	0.00001	0.70845	0.00018			
r	14	47.45421	9.61307	Tertiary	Lower Freshwater molasse	*Älteste Napf-Schüttung*	Blueberry (*Vaccinium myrtillus*)	0.71067	0.00001	Beech (*Fagus sylvatica*)	0.71118	0.00001	0.71093	0.00036	0.71048	0.00118	0.70848-0.71181
s	15	47.45136	9.61907	Tertiary	Lower Freshwater molasse	*Älteste Napf-Schüttung*	Woodruff (*Galium odoratum*)	0.71033	0.00001	Beech (*Fagus sylvatica*)	0.71039	0.00001	0.71036	0.00004			
t	16	47.44740	9.62814	Tertiary	Lower Freshwater molasse	*Höhronen-Schüttung*	Raspberry (*Rubus idaeus*)	0.71138	0.00001	Beech (*Fagus sylvatica*)	0.71224	0.00001	0.71181	0.00061			
i	17	47.39076	9.45848	Tertiary	Lower Freshwater molasse	*Chattien*	Wood anemone (*Anemone nemorosa*)	0.71150	0.00001	Beech (*Fagus sylvatica*)	0.71118	0.00001	0.71134	0.00023			
l	72	47.43501	9.49424	Tertiary	Lower Freshwater molasse	*Aquitanien*	Common wood sorrel (*Oxalis acetosella*)	0.71008	0.00001	Beech (*Fagus sylvatica*)	0.70986	0.00001	0.70997	0.00016			
--	85	47.24761	9.18073	Tertiary	Lower Freshwater molasse	*Kronberg-Gäbris-Schüttung*	Ivy (*Hedera helix*)	0.70854	0.00001	Beech (*Fagus sylvatica*)	0.70842	0.00001	0.70848	0.00008			
--	84	47.21990	9.20837	Tertiary	Lower Seawater molasse	*Grisigen-Mergel*	Dandelion (*Taraxacum officinale*)	0.70819	0.00001	Lime (*Tilia sp.*)	0.70825	0.00001	0.70822	0.00004			0.70822
--	79	47.19943	9.28029	Cretaceous	*Säntis-Decke*	*Seewer Kalk*	Woodruff (*Galium odoratum*)	0.70781	0.00001	Beech (*Fagus sylvatica*)	0.70798	0.00001	0.70790	0.00012	0.70812	0.00032	0.70770-0.70850
--	80	47.19645	9.27102	Cretaceous	*Säntis-Decke*	*Garschella-Formation*	Fern (*Polypodiopsida sp.*)	0.70851	0.00001	Maple (*Acer pseudoplatanus*)	0.70849	0.00001	0.70850	0.00001			
--	81	47.19409	9.25761	Cretaceous	*Säntis-Decke*	*Oberer Schrattenkalk*	Woodruff (*Galium odoratum*)	0.70768	0.00001	Beech (*Fagus sylvatica*)	0.70772	0.00001	0.70770	0.00003			
--	82	47.19472	9.24203	Cretaceous	*Säntis-Decke*	*Helvetischer Kieselkalk*	Wild garlic (*Allium ursinum*)	0.70841	0.00001	Beech (*Fagus sylvatica*)	0.70818	0.00001	0.70830	0.00016			
--	83	47.19721	9.24530	Cretaceous	*Säntis-Decke*	*Drusberg-Formation*	Common wood sorrel (*Oxalis acetosella*)	0.70820	0.00001	Beech (*Fagus sylvatica*)	0.70824	0.00001	0.70822	0.00003			
--	90	47.10240	9.20802	Permian	*Glarner Decke*	*Verrucano (Murgtal Sernifit)*	Blackberry (*Rubus fruticosus*)	0.71155	0.00001	Maple (*Acer pseudoplatanus*)	0.71207	0.00001	0.71181	0.00037	0.71242	0.00086	0.71181-0.71303
--	99	47.00147	9.11616	Permian	*Glarner Decke ?*	*Verrucano (Sernifit)*	Fern (*Polypodiopsida sp.*)	0.71238	0.00001	Maple (*Acer pseudoplatanus*)	0.71367	0.00001	0.71303	0.00091			
