# Supplementary material for: High-resolution isotopic evidence of specialised cattle herding in the European Neolithic
Source: PLoS One. 2017 Jul 26;12(7):e0180164. doi: 10.1371/journal.pone.0180164 (PMC5528262; doi:10.1371/journal.pone.0180164)
Supplement: S2 Table — Prehistoric fauna was analysed by LA-MC-ICP-MS (spot measurements, Bos taurus and Cervus elaphus) and TIMS (Sus domesticus) and modern local water was analysed by TIMS. Water samples were collected in May 2013. (PDF) [file pone.0180164.s008.pdf]

Lab ID	Species/Location	Sample	Latitude	Longitude	$^{87}Sr/^{86}Sr$	2 SE
ARB 23.2.1 dent	*Bos taurus*	dentine			0.70877	0.00004
ARB 25.2.1 dent	*Bos taurus*	dentine			0.70864	0.00005
ARB 27.2.1 dent	*Bos taurus*	dentine			0.70894	0.00006
ARB 33.4.1 dent	*Bos taurus*	dentine			0.70923	0.00005
ARB 10.2.1 dent	*Bos taurus*	dentine			0.70860	0.00005
ARB 13.2.1 dent	*Bos taurus*	dentine			0.70873	0.00004
ARB 14.2.1 dent	*Bos taurus*	dentine			0.70866	0.00005
ARB 16.2.1 dent	*Bos taurus*	dentine			0.70877	0.00006
ARB 29.2.1 dent	*Bos taurus*	dentine			0.70860	0.00005
ARB 114.2.1 dent	*Bos taurus*	dentine			0.70897	0.00003
ARB 34.2.1 dent	*Bos taurus*	dentine			0.70889	0.00006
ARB 110.3.1 dent	*Bos taurus*	dentine			0.70874	0.00005
ARB 50.2.1 dent	*Cervus elaphus*	dentine			0.70863	0.00004
ARB 55.2.1 dent	*Cervus elaphus*	dentine			0.70860	0.00006
ARB 56.2.1 dent	*Cervus elaphus*	dentine			0.70855	0.00006
ARB 57.2.1 dent	*Cervus elaphus*	dentine			0.70858	0.00005
A 58 R	*Sus domesticus*	enamel			0.70832	0.00001
A 61 R	*Sus domesticus*	enamel			0.70861	0.00001
A 62 R	*Sus domesticus*	enamel			0.70869	0.00001
A 63 R	*Sus domesticus*	enamel			0.70875	0.00001
A 64 R	*Sus domesticus*	enamel			0.70870	0.00001
A 68 R	*Sus domesticus*	enamel			0.70852	0.00001
A 69 R	*Sus domesticus*	enamel			0.70834	0.00001
2	Lake Constance	water	47.51551	9.44031	0.70852	0.00001
10	River Aach	water	47.50995	9.42265	0.70842	0.00001
