# Supplementary material for: High-resolution isotopic evidence of specialised cattle herding in the European Neolithic
Source: PLoS One. 2017 Jul 26;12(7):e0180164. doi: 10.1371/journal.pone.0180164 (PMC5528262; doi:10.1371/journal.pone.0180164)
Supplement: S3 Table — The length of the laser track (distance from cervix) is shorter than the length of tooth enamel, as given in Table 1. M1 = first molar, M2 = second molar, M3 = third molar. (PDF) [file pone.0180164.s009.pdf]

ARB 2.2.1 (M2)

Distance from cervix (mm)	$^{87}Sr/^{86}Sr$	10 point mov. average	2 SE on mov. average
41.20	0.70886	0.70859	0.00021
41.16	0.70875	0.70853	0.00021
41.11	0.70865	0.70849	0.00021
41.07	0.70863	0.70843	0.00022
41.03	0.70889	0.70846	0.00023
40.98	0.70858	0.70834	0.00026
40.94	0.70802	0.70827	0.00026
40.89	0.70855	0.70832	0.00026
40.85	0.70894	0.70827	0.00026
40.81	0.70800	0.70821	0.00022
40.76	0.70830	0.70825	0.00022
40.72	0.70832	0.70831	0.00025
40.68	0.70808	0.70830	0.00025
40.63	0.70889	0.70831	0.00025
40.59	0.70767	0.70825	0.00021
40.55	0.70789	0.70833	0.00017
40.50	0.70851	0.70836	0.00015
40.46	0.70805	0.70836	0.00015
40.41	0.70842	0.70838	0.00014
40.37	0.70837	0.70835	0.00014
40.33	0.70888	0.70834	0.00015
40.28	0.70822	0.70828	0.00009
40.24	0.70823	0.70827	0.00010
40.20	0.70824	0.70827	0.00010
40.15	0.70848	0.70822	0.00013
40.11	0.70816	0.70822	0.00013
40.07	0.70856	0.70823	0.00013
40.02	0.70819	0.70819	0.00011
39.98	0.70821	0.70821	0.00012
39.93	0.70818	0.70824	0.00013
39.89	0.70835	0.70825	0.00013
39.85	0.70807	0.70824	0.00013
39.80	0.70824	0.70830	0.00015
39.76	0.70780	0.70833	0.00015
39.72	0.70846	0.70836	0.00011
39.67	0.70824	0.70835	0.00011
39.63	0.70814	0.70836	0.00011
39.59	0.70845	0.70841	0.00011
39.54	0.70850	0.70837	0.00013
39.45	0.70825	0.70834	0.00013
39.41	0.70829	0.70836	0.00013
39.37	0.70867	0.70837	0.00013
39.32	0.70850	0.70839	0.00015
39.28	0.70812	0.70838	0.00015
39.24	0.70833	0.70842	0.00014
39.19	0.70836	0.70843	0.00013
39.15	0.70862	0.70846	0.00014

ARB 2.3.1 (M3)

Distance from cervix (mm)	$^{87}Sr/^{86}Sr$	10 point mov. average	2 SE on mov. average
42.83	0.70828	0.70813	0.00012
42.79	0.70829	0.70809	0.00012
42.74	0.70814	0.70805	0.00012
42.70	0.70794	0.70808	0.00014
42.65	0.70819	0.70809	0.00013
42.61	0.70779	0.70811	0.00014
42.56	0.70820	0.70812	0.00013
42.52	0.70803	0.70813	0.00014
42.48	0.70841	0.70816	0.00014
42.43	0.70800	0.70813	0.00013
42.39	0.70794	0.70816	0.00013
42.34	0.70787	0.70819	0.00012
42.30	0.70840	0.70823	0.00010
42.25	0.70810	0.70818	0.00010
42.21	0.70834	0.70822	0.00012
42.17	0.70789	0.70823	0.00012
42.12	0.70828	0.70829	0.00011
42.08	0.70833	0.70830	0.00011
42.03	0.70816	0.70829	0.00011
41.99	0.70833	0.70830	0.00011
41.94	0.70818	0.70833	0.00014
41.90	0.70826	0.70834	0.00013
41.86	0.70793	0.70835	0.00013
41.81	0.70847	0.70833	0.00016
41.77	0.70846	0.70830	0.00016
41.72	0.70848	0.70827	0.00016
41.68	0.70844	0.70825	0.00015
41.63	0.70821	0.70826	0.00016
41.59	0.70822	0.70826	0.00016
41.55	0.70869	0.70826	0.00016
41.50	0.70828	0.70820	0.00012
41.46	0.70827	0.70822	0.00013
41.41	0.70776	0.70825	0.00015
41.37	0.70814	0.70831	0.00010
41.32	0.70816	0.70833	0.00009
41.28	0.70830	0.70835	0.00009
41.24	0.70855	0.70835	0.00009
41.19	0.70823	0.70835	0.00008
41.15	0.70821	0.70834	0.00010
41.10	0.70813	0.70835	0.00010
41.06	0.70844	0.70838	0.00008
41.01	0.70852	0.70839	0.00009
40.97	0.70839	0.70835	0.00010
40.93	0.70840	0.70830	0.00014
40.88	0.70834	0.70825	0.00015
40.84	0.70832	0.70824	0.00015
40.79	0.70854	0.70824	0.00015

39.11	0.70803	0.70842	0.00014
39.06	0.70821	0.70848	0.00011
39.02	0.70848	0.70852	0.00010
38.97	0.70843	0.70855	0.00012
38.93	0.70881	0.70856	0.00011
38.89	0.70845	0.70854	0.00010
38.84	0.70849	0.70853	0.00011
38.80	0.70843	0.70851	0.00012
38.76	0.70863	0.70849	0.00013
38.71	0.70824	0.70845	0.00014
38.67	0.70859	0.70847	0.00013
38.62	0.70865	0.70841	0.00016
38.58	0.70882	0.70837	0.00015
38.54	0.70847	0.70833	0.00012
38.49	0.70865	0.70832	0.00011
38.45	0.70835	0.70828	0.00009
38.41	0.70829	0.70829	0.00009
38.36	0.70820	0.70835	0.00015
38.32	0.70822	0.70840	0.00017
38.28	0.70849	0.70843	0.00016
38.23	0.70798	0.70840	0.00017
38.19	0.70825	0.70843	0.00014
38.14	0.70835	0.70843	0.00014
38.10	0.70844	0.70844	0.00014
38.06	0.70825	0.70847	0.00015
38.01	0.70838	0.70848	0.00015
37.97	0.70890	0.70849	0.00014
37.93	0.70873	0.70840	0.00014
37.88	0.70850	0.70841	0.00015
37.84	0.70822	0.70838	0.00015
37.80	0.70827	0.70840	0.00014
37.75	0.70829	0.70839	0.00015
37.71	0.70843	0.70839	0.00015
37.66	0.70874	0.70841	0.00015
37.62	0.70839	0.70834	0.00015
37.58	0.70847	0.70838	0.00017
37.53	0.70800	0.70836	0.00017
37.49	0.70876	0.70839	0.00015
37.45	0.70823	0.70838	0.00014
37.40	0.70840	0.70838	0.00014
37.36	0.70820	0.70839	0.00014
37.32	0.70830	0.70843	0.00014
37.27	0.70858	0.70840	0.00017
37.23	0.70806	0.70839	0.00016
37.18	0.70878	0.70840	0.00015
37.14	0.70831	0.70833	0.00014
37.10	0.70830	0.70833	0.00014
37.05	0.70861	0.70836	0.00015
37.01	0.70823	0.70832	0.00013
36.97	0.70854	0.70833	0.00013

40.75	0.70806	0.70823	0.00014
40.70	0.70835	0.70828	0.00015
40.66	0.70842	0.70829	0.00015
40.62	0.70855	0.70824	0.00017
40.57	0.70810	0.70823	0.00016
40.53	0.70790	0.70828	0.00018
40.48	0.70795	0.70834	0.00017
40.44	0.70819	0.70838	0.00014
40.39	0.70835	0.70836	0.00016
40.35	0.70845	0.70831	0.00018
40.31	0.70857	0.70833	0.00019
40.26	0.70843	0.70828	0.00019
40.22	0.70787	0.70823	0.00019
40.17	0.70846	0.70823	0.00019
40.13	0.70868	0.70815	0.00022
40.08	0.70850	0.70809	0.00019
40.04	0.70831	0.70813	0.00024
40.00	0.70794	0.70811	0.00023
39.95	0.70794	0.70815	0.00023
39.91	0.70857	0.70817	0.00023
39.86	0.70807	0.70815	0.00022
39.82	0.70798	0.70821	0.00024
39.77	0.70788	0.70824	0.00023
39.73	0.70761	0.70827	0.00022
39.69	0.70813	0.70832	0.00017
39.64	0.70890	0.70837	0.00018
39.60	0.70809	0.70831	0.00014
39.55	0.70828	0.70833	0.00013
39.51	0.70814	0.70831	0.00013
39.46	0.70844	0.70831	0.00013
39.42	0.70860	0.70830	0.00013
39.38	0.70833	0.70827	0.00011
39.33	0.70819	0.70828	0.00012
39.29	0.70805	0.70836	0.00019
39.24	0.70870	0.70840	0.00018
39.20	0.70832	0.70833	0.00019
39.15	0.70823	0.70832	0.00019
39.11	0.70811	0.70835	0.00019
39.07	0.70815	0.70835	0.00019
39.02	0.70832	0.70835	0.00019
38.98	0.70824	0.70834	0.00019
38.93	0.70845	0.70836	0.00019
38.89	0.70903	0.70836	0.00019
38.84	0.70847	0.70826	0.00013
38.80	0.70792	0.70824	0.00012
38.76	0.70823	0.70825	0.00011
38.71	0.70855	0.70827	0.00011
38.67	0.70809	0.70822	0.00010
38.62	0.70820	0.70826	0.00011
38.58	0.70824	0.70827	0.00011

36.92	0.70861	0.70830	0.00013	38.53	0.70843	0.70825	0.00012
36.88	0.70800	0.70830	0.00013	38.49	0.70843	0.70822	0.00012
36.84	0.70844	0.70833	0.00011	38.45	0.70805	0.70821	0.00011
36.79	0.70814	0.70833	0.00011	38.40	0.70824	0.70825	0.00011
36.75	0.70810	0.70834	0.00011	38.36	0.70806	0.70827	0.00012
36.70	0.70833	0.70833	0.00012	38.31	0.70842	0.70827	0.00012
36.62	0.70858	0.70835	0.00013	38.27	0.70806	0.70827	0.00012
36.57	0.70826	0.70834	0.00012	38.22	0.70849	0.70831	0.00012
36.53	0.70828	0.70837	0.00012	38.18	0.70832	0.70830	0.00012
36.49	0.70824	0.70835	0.00014	38.14	0.70799	0.70827	0.00013
36.44	0.70867	0.70837	0.00014	38.09	0.70814	0.70829	0.00011
36.40	0.70829	0.70835	0.00013	38.05	0.70838	0.70833	0.00012
36.36	0.70844	0.70838	0.00013	38.00	0.70840	0.70832	0.00012
36.31	0.70819	0.70836	0.00013	37.96	0.70848	0.70830	0.00012
36.27	0.70803	0.70844	0.00017	37.91	0.70803	0.70831	0.00013
36.22	0.70855	0.70850	0.00015	37.87	0.70840	0.70834	0.00011
36.18	0.70843	0.70849	0.00015	37.83	0.70851	0.70830	0.00013
36.14	0.70856	0.70846	0.00016	37.78	0.70835	0.70827	0.00012
36.09	0.70807	0.70845	0.00016	37.74	0.70803	0.70828	0.00012
36.05	0.70849	0.70845	0.00016	37.69	0.70819	0.70828	0.00012
36.01	0.70851	0.70844	0.00016	37.65	0.70853	0.70823	0.00017
35.96	0.70854	0.70842	0.00016	37.60	0.70833	0.70815	0.00017
35.92	0.70826	0.70837	0.00017	37.56	0.70814	0.70815	0.00017
35.88	0.70895	0.70837	0.00017	37.52	0.70857	0.70819	0.00018
35.83	0.70869	0.70829	0.00012	37.47	0.70831	0.70812	0.00017
35.79	0.70836	0.70823	0.00008	37.43	0.70803	0.70813	0.00018
35.74	0.70821	0.70824	0.00009	37.38	0.70822	0.70818	0.00019
35.70	0.70837	0.70827	0.00010	37.34	0.70842	0.70822	0.00021
35.66	0.70810	0.70826	0.00010	37.29	0.70804	0.70820	0.00021
35.61	0.70842	0.70829	0.00010	37.25	0.70770	0.70822	0.00021
35.57	0.70829	0.70825	0.00012	37.20	0.70778	0.70826	0.00018
35.53	0.70807	0.70823	0.00012	37.16	0.70831	0.70828	0.00015
35.48	0.70824	0.70827	0.00012	37.12	0.70848	0.70828	0.00015
35.44	0.70813	0.70830	0.00013	37.07	0.70788	0.70831	0.00018
35.40	0.70809	0.70831	0.00012	37.03	0.70839	0.70834	0.00015
35.35	0.70844	0.70837	0.00013	36.98	0.70855	0.70834	0.00015
35.31	0.70851	0.70838	0.00013	36.94	0.70863	0.70830	0.00015
35.26	0.70829	0.70837	0.00013	36.89	0.70821	0.70829	0.00013
35.22	0.70846	0.70836	0.00014	36.85	0.70829	0.70827	0.00014
35.18	0.70796	0.70836	0.00014	36.81	0.70802	0.70826	0.00014
35.13	0.70815	0.70839	0.00011	36.76	0.70805	0.70831	0.00014
35.09	0.70841	0.70843	0.00010	36.72	0.70834	0.70834	0.00013
35.05	0.70852	0.70843	0.00010	36.67	0.70873	0.70833	0.00013
35.00	0.70827	0.70841	0.00010	36.63	0.70821	0.70831	0.00011
34.96	0.70866	0.70842	0.00009	36.58	0.70837	0.70833	0.00011
34.92	0.70855	0.70838	0.00008	36.54	0.70818	0.70835	0.00012
34.87	0.70846	0.70835	0.00008	36.50	0.70847	0.70833	0.00014
34.83	0.70812	0.70832	0.00008	36.45	0.70801	0.70834	0.00015
34.78	0.70852	0.70834	0.00007	36.41	0.70824	0.70834	0.00015
34.74	0.70829	0.70834	0.00007	36.36	0.70850	0.70839	0.00017

34.70	0.70846	0.70836	0.00007	36.32	0.70834	0.70836	0.00017
34.65	0.70841	0.70833	0.00008	36.27	0.70824	0.70832	0.00018
34.61	0.70832	0.70828	0.00010	36.23	0.70858	0.70829	0.00020
34.57	0.70841	0.70831	0.00012	36.19	0.70834	0.70826	0.00019
34.52	0.70823	0.70828	0.00012	36.14	0.70864	0.70824	0.00020
34.48	0.70829	0.70827	0.00012	36.10	0.70796	0.70822	0.00018
34.44	0.70815	0.70831	0.00014	36.05	0.70859	0.70821	0.00019
34.39	0.70835	0.70834	0.00014	36.01	0.70799	0.70822	0.00019
34.35	0.70849	0.70837	0.00016	35.96	0.70871	0.70824	0.00019
34.30	0.70846	0.70834	0.00016	35.92	0.70818	0.70817	0.00016
34.26	0.70816	0.70831	0.00016	35.88	0.70801	0.70818	0.00017
34.22	0.70797	0.70832	0.00016	35.83	0.70788	0.70819	0.00016
34.17	0.70857	0.70834	0.00014	35.79	0.70835	0.70823	0.00015
34.13	0.70819	0.70833	0.00014	35.74	0.70808	0.70823	0.00015
34.09	0.70812	0.70829	0.00016	35.70	0.70843	0.70824	0.00015
34.04	0.70865	0.70833	0.00017	35.65	0.70790	0.70824	0.00015
34.00	0.70842	0.70827	0.00016	35.61	0.70866	0.70828	0.00013
33.96	0.70869	0.70827	0.00016	35.57	0.70823	0.70826	0.00011
33.91	0.70822	0.70823	0.00013	35.52	0.70794	0.70835	0.00021
33.87	0.70809	0.70827	0.00014	35.48	0.70837	0.70840	0.00019
33.82	0.70830	0.70828	0.00014	35.43	0.70806	0.70841	0.00019
33.78	0.70814	0.70834	0.00019	35.39	0.70824	0.70843	0.00018
33.74	0.70843	0.70835	0.00018	35.34	0.70843	0.70840	0.00020
33.69	0.70786	0.70837	0.00019	35.30	0.70813	0.70837	0.00021
33.65	0.70852	0.70843	0.00015	35.26	0.70840	0.70837	0.00021
33.61	0.70804	0.70844	0.00016	35.21	0.70839	0.70836	0.00021
33.56	0.70842	0.70849	0.00013	35.17	0.70841	0.70836	0.00021
33.52	0.70831	0.70849	0.00013	35.12	0.70917	0.70834	0.00021
33.47	0.70856	0.70850	0.00012	35.08	0.70837	0.70828	0.00012
33.43	0.70822	0.70850	0.00012	35.03	0.70847	0.70827	0.00012
33.39	0.70890	0.70849	0.00013	34.99	0.70827	0.70830	0.00014
33.34	0.70826	0.70843	0.00010	34.95	0.70791	0.70833	0.00016
33.30	0.70859	0.70842	0.00011	34.90	0.70822	0.70833	0.00016
33.26	0.70844	0.70837	0.00012	34.86	0.70806	0.70833	0.00016
33.21	0.70866	0.70835	0.00013	34.81	0.70831	0.70838	0.00015
33.17	0.70851	0.70833	0.00011	34.77	0.70839	0.70837	0.00016
33.13	0.70843	0.70830	0.00011	34.72	0.70822	0.70837	0.00016
33.08	0.70845	0.70829	0.00010	34.68	0.70859	0.70837	0.00016
33.04	0.70853	0.70828	0.00010	34.64	0.70829	0.70835	0.00015
32.99	0.70812	0.70826	0.00008	34.59	0.70870	0.70831	0.00017
32.95	0.70832	0.70827	0.00008	34.55	0.70861	0.70825	0.00015
32.91	0.70815	0.70828	0.00008	34.50	0.70789	0.70823	0.00013
32.86	0.70810	0.70828	0.00008	34.46	0.70827	0.70829	0.00012
32.82	0.70818	0.70833	0.00010	34.41	0.70855	0.70830	0.00012
32.78	0.70849	0.70837	0.00010	34.37	0.70818	0.70828	0.00011
32.73	0.70819	0.70834	0.00010	34.33	0.70840	0.70827	0.00012
32.69	0.70838	0.70834	0.00010	34.28	0.70821	0.70829	0.00013
32.65	0.70832	0.70835	0.00010	34.24	0.70837	0.70825	0.00015
32.60	0.70838	0.70834	0.00010	34.19	0.70793	0.70823	0.00015
32.56	0.70823	0.70833	0.00010	34.15	0.70810	0.70827	0.00014

32.51	0.70835	0.70834	0.00010	34.10	0.70841	0.70827	0.00014
32.47	0.70814	0.70836	0.00011	34.06	0.70848	0.70826	0.00013
32.43	0.70865	0.70836	0.00010	34.02	0.70840	0.70828	0.00015
32.38	0.70853	0.70834	0.00008	33.97	0.70834	0.70826	0.00015
32.34	0.70817	0.70835	0.00009	33.93	0.70806	0.70822	0.00016
32.30	0.70824	0.70836	0.00008	33.88	0.70854	0.70825	0.00016
32.25	0.70845	0.70839	0.00009	33.84	0.70784	0.70825	0.00016
32.21	0.70826	0.70839	0.00009	33.79	0.70823	0.70829	0.00013
32.17	0.70829	0.70842	0.00009	33.75	0.70827	0.70829	0.00013
32.12	0.70832	0.70845	0.00009	33.71	0.70812	0.70829	0.00013
32.08	0.70852	0.70845	0.00008	33.66	0.70832	0.70831	0.00013
32.03	0.70819	0.70846	0.00009	33.62	0.70865	0.70833	0.00013
31.99	0.70845	0.70850	0.00007	33.57	0.70823	0.70829	0.00011
31.95	0.70859	0.70847	0.00009	33.53	0.70790	0.70830	0.00011
31.90	0.70829	0.70843	0.00010	33.48	0.70835	0.70835	0.00006
31.86	0.70858	0.70841	0.00012	33.44	0.70853	0.70835	0.00006
31.82	0.70842	0.70840	0.00011	33.40	0.70832	0.70831	0.00007
31.77	0.70852	0.70842	0.00012	33.35	0.70822	0.70832	0.00007
31.73	0.70858	0.70839	0.00012	33.31	0.70822	0.70837	0.00011
31.69	0.70840	0.70837	0.00011	33.26	0.70840	0.70838	0.00010
31.64	0.70855	0.70834	0.00012	33.22	0.70844	0.70840	0.00011
31.60	0.70859	0.70834	0.00012	33.17	0.70833	0.70840	0.00011
31.55	0.70816	0.70831	0.00011	33.13	0.70833	0.70840	0.00011
31.51	0.70820	0.70834	0.00011	33.09	0.70834	0.70839	0.00011
31.47	0.70815	0.70836	0.00010	33.04	0.70840	0.70837	0.00013
31.42	0.70843	0.70837	0.00009	33.00	0.70808	0.70835	0.00013
31.38	0.70860	0.70840	0.00011	32.95	0.70842	0.70837	0.00012
31.34	0.70825	0.70837	0.00010	32.91	0.70874	0.70838	0.00012
31.29	0.70834	0.70843	0.00014	32.86	0.70833	0.70837	0.00011
31.25	0.70814	0.70841	0.00015	32.82	0.70860	0.70837	0.00011
31.21	0.70857	0.70845	0.00014	32.78	0.70843	0.70833	0.00010
31.16	0.70825	0.70840	0.00016	32.73	0.70837	0.70828	0.00013
31.12	0.70846	0.70844	0.00017	32.69	0.70823	0.70826	0.00013
31.07	0.70839	0.70847	0.00018	32.64	0.70814	0.70826	0.00013
31.03	0.70829	0.70845	0.00018	32.60	0.70818	0.70826	0.00013
30.99	0.70869	0.70844	0.00019	32.55	0.70830	0.70826	0.00013
30.94	0.70831	0.70840	0.00018	32.51	0.70846	0.70829	0.00014
30.90	0.70883	0.70838	0.00019	32.47	0.70865	0.70826	0.00013
30.86	0.70814	0.70833	0.00016	32.42	0.70832	0.70824	0.00011
30.81	0.70859	0.70837	0.00016	32.38	0.70822	0.70820	0.00012
30.77	0.70799	0.70833	0.00016	32.33	0.70789	0.70820	0.00012
30.73	0.70872	0.70836	0.00014	32.29	0.70819	0.70824	0.00010
30.68	0.70871	0.70832	0.00012	32.24	0.70821	0.70825	0.00010
30.64	0.70825	0.70827	0.00008	32.20	0.70817	0.70823	0.00010
30.59	0.70814	0.70831	0.00011	32.16	0.70821	0.70824	0.00010
30.55	0.70829	0.70831	0.00012	32.11	0.70853	0.70825	0.00010
30.51	0.70816	0.70831	0.00012	32.07	0.70821	0.70824	0.00010
30.46	0.70837	0.70833	0.00011	32.02	0.70841	0.70826	0.00010
30.42	0.70852	0.70832	0.00011	31.98	0.70797	0.70823	0.00010
30.38	0.70812	0.70827	0.00012	31.93	0.70818	0.70824	0.00009

30.33	0.70832	0.70830	0.00011	31.89	0.70833	0.70826	0.00009
30.29	0.70831	0.70828	0.00012	31.85	0.70824	0.70828	0.00010
30.25	0.70821	0.70830	0.00013	31.80	0.70807	0.70831	0.00012
30.20	0.70868	0.70830	0.00013	31.76	0.70823	0.70830	0.00012
30.16	0.70809	0.70826	0.00010	31.71	0.70831	0.70831	0.00012
30.11	0.70838	0.70830	0.00010	31.67	0.70849	0.70831	0.00012
30.07	0.70836	0.70831	0.00011	31.62	0.70836	0.70832	0.00012
30.03	0.70821	0.70833	0.00012	31.58	0.70807	0.70829	0.00012
29.98	0.70804	0.70835	0.00012	31.54	0.70809	0.70831	0.00011
29.94	0.70839	0.70836	0.00010	31.49	0.70840	0.70834	0.00010
29.90	0.70810	0.70834	0.00011	31.45	0.70851	0.70831	0.00011
29.85	0.70851	0.70836	0.00010	31.40	0.70854	0.70830	0.00011
29.81	0.70826	0.70833	0.00010	31.36	0.70805	0.70827	0.00010
29.77	0.70825	0.70835	0.00010	31.31	0.70828	0.70828	0.00009
29.72	0.70848	0.70834	0.00011	31.27	0.70835	0.70827	0.00009
29.68	0.70851	0.70830	0.00011	31.23	0.70851	0.70826	0.00009
29.63	0.70858	0.70825	0.00011	31.18	0.70814	0.70823	0.00007
29.59	0.70836	0.70822	0.00007	31.14	0.70825	0.70830	0.00014
29.55	0.70818	0.70822	0.00008	31.09	0.70837	0.70829	0.00015
29.50	0.70815	0.70823	0.00008	31.05	0.70808	0.70832	0.00016
29.46	0.70817	0.70825	0.00008	31.00	0.70844	0.70838	0.00017
29.42	0.70843	0.70827	0.00008	30.96	0.70827	0.70836	0.00017
29.37	0.70816	0.70824	0.00008	30.92	0.70808	0.70835	0.00018
29.33	0.70816	0.70824	0.00008	30.87	0.70823	0.70837	0.00017
29.29	0.70806	0.70828	0.00009	30.83	0.70821	0.70837	0.00016
29.24	0.70829	0.70833	0.00010	30.78	0.70824	0.70839	0.00016
29.20	0.70842	0.70837	0.00012	30.74	0.70886	0.70838	0.00016
29.15	0.70825	0.70833	0.00014	30.69	0.70812	0.70832	0.00012
29.11	0.70833	0.70833	0.00014	30.65	0.70861	0.70834	0.00012
29.07	0.70824	0.70835	0.00015	30.61	0.70870	0.70831	0.00010
29.02	0.70841	0.70835	0.00015	30.56	0.70832	0.70830	0.00008
28.98	0.70811	0.70833	0.00015	30.52	0.70808	0.70831	0.00008
28.94	0.70816	0.70833	0.00015	30.47	0.70831	0.70835	0.00007
28.89	0.70850	0.70836	0.00015	30.43	0.70829	0.70833	0.00008
28.85	0.70864	0.70838	0.00016	30.38	0.70834	0.70833	0.00008
28.81	0.70866	0.70834	0.00014	30.34	0.70819	0.70831	0.00009
28.76	0.70797	0.70828	0.00014	30.30	0.70823	0.70834	0.00009
28.72	0.70831	0.70825	0.00017	30.25	0.70831	0.70834	0.00009
28.67	0.70847	0.70827	0.00017	30.21	0.70833	0.70834	0.00009
28.63	0.70823	0.70820	0.00019	30.16	0.70858	0.70836	0.00010
28.59	0.70820	0.70822	0.00019	30.12	0.70844	0.70835	0.00008
28.54	0.70812	0.70821	0.00019	30.07	0.70847	0.70833	0.00009
28.50	0.70853	0.70824	0.00020	30.03	0.70816	0.70833	0.00008
28.46	0.70864	0.70821	0.00019	29.99	0.70825	0.70836	0.00008
28.41	0.70828	0.70823	0.00020	29.94	0.70818	0.70836	0.00007
28.37	0.70807	0.70820	0.00020	29.90	0.70848	0.70838	0.00006
28.32	0.70769	0.70824	0.00021	29.85	0.70826	0.70837	0.00006
28.28	0.70843	0.70835	0.00019	29.81	0.70829	0.70836	0.00008
28.24	0.70784	0.70836	0.00020	29.76	0.70853	0.70837	0.00008
28.19	0.70842	0.70841	0.00016	29.72	0.70840	0.70834	0.00007

28.15	0.70807	0.70842	0.00016
28.11	0.70839	0.70843	0.00015
28.06	0.70832	0.70844	0.00015
28.02	0.70878	0.70845	0.00015
27.98	0.70803	0.70837	0.00015
27.93	0.70846	0.70840	0.00013
27.89	0.70877	0.70836	0.00015
27.84	0.70854	0.70831	0.00011
27.80	0.70831	0.70832	0.00012
27.76	0.70852	0.70831	0.00013
27.71	0.70816	0.70827	0.00012
27.67	0.70850	0.70830	0.00012
27.63	0.70840	0.70826	0.00012
27.58	0.70804	0.70825	0.00012
27.54	0.70830	0.70824	0.00012
27.50	0.70808	0.70824	0.00012
27.45	0.70830	0.70830	0.00014
27.41	0.70864	0.70829	0.00015
27.36	0.70813	0.70824	0.00012
27.32	0.70821	0.70822	0.00014
27.28	0.70839	0.70822	0.00014
27.23	0.70808	0.70824	0.00015
27.19	0.70831	0.70827	0.00015
27.15	0.70799	0.70826	0.00015
27.10	0.70825	0.70828	0.00014
27.06	0.70868	0.70833	0.00017
27.02	0.70817	0.70828	0.00015
26.97	0.70824	0.70832	0.00015
26.93	0.70787	0.70832	0.00016
26.88	0.70826	0.70837	0.00012
26.84	0.70850	0.70838	0.00012
26.80	0.70847	0.70841	0.00014
26.75	0.70819	0.70838	0.00015
26.71	0.70821	0.70840	0.00014
26.67	0.70872	0.70843	0.00013
26.62	0.70821	0.70839	0.00012
26.58	0.70857	0.70838	0.00012
26.54	0.70820	0.70840	0.00015
26.49	0.70838	0.70843	0.00014
26.45	0.70836	0.70841	0.00015
26.40	0.70879	0.70842	0.00015
26.36	0.70820	0.70841	0.00014
26.32	0.70838	0.70849	0.00018
26.27	0.70846	0.70851	0.00018
26.23	0.70834	0.70850	0.00018
26.19	0.70813	0.70853	0.00018
26.14	0.70881	0.70854	0.00017
26.10	0.70845	0.70851	0.00016
26.06	0.70815	0.70850	0.00016
26.01	0.70855	0.70853	0.00015

29.63	0.70839	0.70833	0.00007
29.59	0.70846	0.70831	0.00007
29.54	0.70828	0.70834	0.00012
29.50	0.70833	0.70837	0.00012
29.45	0.70841	0.70839	0.00012
29.41	0.70811	0.70839	0.00012
29.37	0.70844	0.70838	0.00014
29.32	0.70829	0.70833	0.00016
29.28	0.70825	0.70835	0.00016
29.23	0.70831	0.70836	0.00016
29.19	0.70823	0.70838	0.00017
29.14	0.70880	0.70841	0.00017
29.10	0.70854	0.70837	0.00014
29.06	0.70850	0.70835	0.00014
29.01	0.70846	0.70835	0.00014
28.97	0.70795	0.70831	0.00014
28.92	0.70797	0.70835	0.00012
28.88	0.70844	0.70840	0.00008
28.83	0.70836	0.70838	0.00010
28.79	0.70859	0.70835	0.00012
28.75	0.70847	0.70832	0.00010
28.70	0.70840	0.70832	0.00011
28.66	0.70836	0.70830	0.00011
28.61	0.70848	0.70829	0.00011
28.57	0.70808	0.70830	0.00012
28.52	0.70841	0.70836	0.00013
28.48	0.70847	0.70834	0.00013
28.44	0.70815	0.70830	0.00013
28.39	0.70808	0.70831	0.00013
28.35	0.70830	0.70833	0.00012
28.30	0.70851	0.70837	0.00014
28.26	0.70820	0.70832	0.00015
28.21	0.70821	0.70832	0.00015
28.17	0.70859	0.70831	0.00015
28.13	0.70866	0.70830	0.00014
28.08	0.70828	0.70830	0.00015
28.04	0.70807	0.70828	0.00016
27.99	0.70818	0.70829	0.00015
27.95	0.70834	0.70829	0.00015
27.90	0.70865	0.70831	0.00016
27.86	0.70800	0.70827	0.00014
27.82	0.70821	0.70833	0.00013
27.77	0.70815	0.70833	0.00013
27.73	0.70848	0.70833	0.00013
27.68	0.70866	0.70831	0.00013
27.64	0.70801	0.70828	0.00011
27.59	0.70819	0.70830	0.00009
27.55	0.70819	0.70830	0.00009
27.51	0.70854	0.70830	0.00009
27.46	0.70829	0.70829	0.00009

25.97	0.70862	0.70857	0.00017
25.92	0.70905	0.70854	0.00017
25.88	0.70852	0.70846	0.00014
25.84	0.70839	0.70841	0.00016
25.79	0.70867	0.70845	0.00017
25.75	0.70820	0.70845	0.00017
25.71	0.70854	0.70847	0.00016
25.66	0.70830	0.70844	0.00017
25.62	0.70844	0.70846	0.00017
25.58	0.70893	0.70849	0.00018
25.53	0.70836	0.70844	0.00015
25.49	0.70827	0.70843	0.00015
25.44	0.70804	0.70838	0.00019
25.40	0.70872	0.70839	0.00019
25.36	0.70868	0.70832	0.00018
25.31	0.70842	0.70832	0.00017
25.27	0.70819	0.70829	0.00018
25.23	0.70856	0.70830	0.00018
25.18	0.70873	0.70824	0.00018
25.14	0.70841	0.70820	0.00014
25.10	0.70825	0.70819	0.00014
25.05	0.70783	0.70822	0.00016
25.01	0.70805	0.70828	0.00014
24.96	0.70812	0.70831	0.00013
24.92	0.70859	0.70833	0.00012
24.88	0.70812	0.70830	0.00011
24.83	0.70833	0.70831	0.00010
24.79	0.70796	0.70827	0.00012
24.75	0.70834	0.70832	0.00010
24.70	0.70828	0.70833	0.00010
24.66	0.70857	0.70836	0.00011
24.62	0.70848	0.70834	0.00010
24.57	0.70833	0.70832	0.00009
24.53	0.70831	0.70835	0.00011
24.48	0.70828	0.70834	0.00011
24.44	0.70820	0.70831	0.00013
24.40	0.70802	0.70830	0.00014
24.35	0.70843	0.70835	0.00012
24.31	0.70846	0.70833	0.00012
24.27	0.70852	0.70831	0.00012
24.22	0.70843	0.70830	0.00011
24.18	0.70821	0.70830	0.00012
24.14	0.70860	0.70830	0.00012
24.09	0.70825	0.70824	0.00011
24.05	0.70797	0.70825	0.00011
24.00	0.70814	0.70825	0.00010
23.96	0.70843	0.70825	0.00011
23.92	0.70827	0.70826	0.00011
23.87	0.70826	0.70826	0.00011
23.83	0.70841	0.70826	0.00011

27.42	0.70853	0.70829	0.00009
27.37	0.70824	0.70827	0.00007
27.33	0.70813	0.70829	0.00008
27.28	0.70831	0.70832	0.00007
27.24	0.70833	0.70832	0.00008
27.20	0.70821	0.70834	0.00009
27.15	0.70827	0.70835	0.00008
27.11	0.70810	0.70835	0.00008
27.06	0.70848	0.70835	0.00008
27.02	0.70835	0.70834	0.00007
26.97	0.70824	0.70835	0.00008
26.93	0.70845	0.70837	0.00007
26.89	0.70843	0.70839	0.00008
26.84	0.70837	0.70839	0.00009
26.80	0.70853	0.70842	0.00010
26.75	0.70823	0.70843	0.00011
26.71	0.70832	0.70847	0.00011
26.66	0.70815	0.70848	0.00010
26.62	0.70833	0.70853	0.00008
26.57	0.70847	0.70853	0.00008
26.53	0.70845	0.70853	0.00008
26.49	0.70858	0.70852	0.00009
26.44	0.70850	0.70848	0.00010
26.40	0.70868	0.70849	0.00011
26.35	0.70857	0.70852	0.00013
26.31	0.70866	0.70845	0.00018
26.26	0.70839	0.70846	0.00018
26.22	0.70865	0.70848	0.00018
26.18	0.70831	0.70846	0.00018
26.13	0.70848	0.70851	0.00019
26.09	0.70833	0.70853	0.00019
26.04	0.70824	0.70856	0.00019
26.00	0.70861	0.70859	0.00017
25.95	0.70894	0.70858	0.00017
25.91	0.70791	0.70853	0.00016
25.87	0.70869	0.70856	0.00011
25.82	0.70859	0.70852	0.00011
25.78	0.70846	0.70850	0.00012
25.73	0.70880	0.70852	0.00012
25.69	0.70869	0.70849	0.00010
25.64	0.70866	0.70847	0.00009
25.60	0.70855	0.70844	0.00008
25.56	0.70850	0.70842	0.00008
25.51	0.70844	0.70839	0.00009
25.47	0.70820	0.70837	0.00010
25.42	0.70834	0.70837	0.00009
25.38	0.70834	0.70837	0.00009
25.33	0.70866	0.70834	0.00011
25.29	0.70853	0.70831	0.00009
25.25	0.70847	0.70829	0.00008

23.79	0.70847	0.70824	0.00010
23.74	0.70819	0.70824	0.00011
23.70	0.70799	0.70827	0.00012
23.66	0.70833	0.70834	0.00012
23.61	0.70803	0.70838	0.00015
23.57	0.70813	0.70842	0.00012
23.52	0.70848	0.70843	0.00011
23.48	0.70829	0.70843	0.00011
23.44	0.70828	0.70844	0.00011
23.39	0.70822	0.70847	0.00010
23.35	0.70848	0.70845	0.00012
23.31	0.70850	0.70842	0.00014
23.26	0.70867	0.70837	0.00016
23.22	0.70873	0.70830	0.00016
23.17	0.70838	0.70825	0.00013
23.13	0.70828	0.70824	0.00012
23.09	0.70849	0.70825	0.00012
23.04	0.70840	0.70826	0.00014
23.00	0.70855	0.70823	0.00014
22.96	0.70806	0.70820	0.00012
22.91	0.70811	0.70821	0.00012
22.87	0.70800	0.70822	0.00012
22.83	0.70802	0.70825	0.00011
22.78	0.70821	0.70831	0.00012
22.74	0.70832	0.70835	0.00013
22.69	0.70832	0.70839	0.00016
22.65	0.70863	0.70834	0.00020
22.61	0.70806	0.70828	0.00020
22.56	0.70830	0.70829	0.00019
22.52	0.70811	0.70833	0.00021
22.48	0.70817	0.70832	0.00021
22.43	0.70836	0.70833	0.00021
22.39	0.70864	0.70829	0.00022
22.35	0.70858	0.70832	0.00024
22.30	0.70877	0.70830	0.00023
22.26	0.70778	0.70824	0.00021
22.21	0.70803	0.70832	0.00019
22.17	0.70819	0.70835	0.00018
22.13	0.70866	0.70835	0.00018
22.08	0.70804	0.70832	0.00017
22.04	0.70828	0.70839	0.00017
22.00	0.70798	0.70843	0.00017
21.95	0.70887	0.70844	0.00016
21.91	0.70841	0.70841	0.00013
21.87	0.70820	0.70840	0.00013
21.82	0.70854	0.70840	0.00013
21.78	0.70837	0.70830	0.00021
21.73	0.70813	0.70830	0.00021
21.69	0.70841	0.70826	0.00023
21.65	0.70868	0.70823	0.00023

25.20	0.70838	0.70826	0.00007
25.16	0.70833	0.70825	0.00006
25.11	0.70819	0.70828	0.00011
25.07	0.70821	0.70828	0.00011
25.02	0.70828	0.70830	0.00011
24.98	0.70831	0.70832	0.00012
24.94	0.70804	0.70830	0.00012
24.89	0.70836	0.70828	0.00014
24.85	0.70831	0.70828	0.00014
24.80	0.70819	0.70825	0.00015
24.76	0.70824	0.70826	0.00015
24.71	0.70870	0.70823	0.00017
24.67	0.70817	0.70819	0.00014
24.63	0.70837	0.70820	0.00014
24.58	0.70850	0.70817	0.00013
24.54	0.70812	0.70812	0.00011
24.49	0.70788	0.70813	0.00012
24.45	0.70834	0.70819	0.00012
24.40	0.70796	0.70818	0.00011
24.36	0.70832	0.70822	0.00011
24.32	0.70792	0.70820	0.00011
24.27	0.70834	0.70823	0.00009
24.23	0.70821	0.70822	0.00009
24.18	0.70807	0.70827	0.00014
24.14	0.70802	0.70826	0.00015
24.09	0.70824	0.70832	0.00015
24.05	0.70845	0.70835	0.00015
24.01	0.70830	0.70838	0.00017
23.96	0.70835	0.70838	0.00017
23.92	0.70807	0.70843	0.00020
23.87	0.70827	0.70841	0.00021
23.83	0.70822	0.70843	0.00021
23.78	0.70875	0.70846	0.00021
23.74	0.70795	0.70843	0.00020
23.70	0.70856	0.70845	0.00018
23.65	0.70858	0.70834	0.00027
23.61	0.70876	0.70831	0.00026
23.56	0.70832	0.70824	0.00024
23.52	0.70886	0.70825	0.00025
23.47	0.70789	0.70820	0.00021
23.43	0.70843	0.70829	0.00022
23.39	0.70852	0.70827	0.00022
23.34	0.70846	0.70821	0.00022
23.30	0.70809	0.70819	0.00022
23.25	0.70745	0.70821	0.00022
23.21	0.70831	0.70830	0.00014
23.16	0.70810	0.70828	0.00015
23.12	0.70837	0.70831	0.00014
23.08	0.70841	0.70833	0.00015
23.03	0.70875	0.70834	0.00015

21.60	0.70867	0.70822	0.00022	22.99	0.70819	0.70828	0.00013
21.56	0.70816	0.70819	0.00020	22.94	0.70792	0.70827	0.00014
21.52	0.70851	0.70825	0.00022	22.90	0.70832	0.70827	0.00013
21.47	0.70833	0.70826	0.00023	22.85	0.70826	0.70826	0.00013
21.43	0.70819	0.70826	0.00023	22.81	0.70841	0.70831	0.00017
21.39	0.70754	0.70832	0.00025	22.77	0.70809	0.70834	0.00018
21.34	0.70834	0.70840	0.00018	22.72	0.70837	0.70833	0.00018
21.30	0.70778	0.70840	0.00018	22.68	0.70855	0.70832	0.00018
21.25	0.70813	0.70851	0.00015	22.63	0.70856	0.70830	0.00017
21.21	0.70855	0.70854	0.00013	22.59	0.70815	0.70825	0.00017
21.17	0.70835	0.70852	0.00013	22.54	0.70803	0.70825	0.00017
21.12	0.70872	0.70848	0.00017	22.50	0.70799	0.70829	0.00016
21.08	0.70863	0.70847	0.00016	22.46	0.70822	0.70833	0.00015
21.04	0.70840	0.70845	0.00016	22.41	0.70878	0.70834	0.00015
20.99	0.70873	0.70846	0.00016	22.37	0.70863	0.70831	0.00011
20.95	0.70841	0.70845	0.00015	22.32	0.70806	0.70828	0.00009
20.91	0.70831	0.70844	0.00016	22.28	0.70826	0.70832	0.00008
20.86	0.70890	0.70846	0.00016	22.23	0.70833	0.70833	0.00008
20.82	0.70840	0.70837	0.00015	22.19	0.70810	0.70830	0.00010
20.77	0.70831	0.70834	0.00016	22.15	0.70811	0.70830	0.00009
20.73	0.70801	0.70834	0.00016	22.10	0.70841	0.70831	0.00009
20.69	0.70864	0.70839	0.00014	22.06	0.70836	0.70828	0.00009
20.64	0.70840	0.70837	0.00013	22.01	0.70836	0.70826	0.00009
20.60	0.70848	0.70834	0.00013	21.97	0.70847	0.70824	0.00010
20.56	0.70865	0.70834	0.00013	21.92	0.70835	0.70818	0.00011
20.51	0.70825	0.70827	0.00013	21.88	0.70844	0.70818	0.00011
20.47	0.70854	0.70826	0.00013	21.84	0.70834	0.70813	0.00011
20.43	0.70801	0.70819	0.00015	21.79	0.70804	0.70811	0.00010
20.38	0.70809	0.70822	0.00015	21.75	0.70816	0.70815	0.00013
20.34	0.70834	0.70823	0.00015	21.70	0.70817	0.70817	0.00014
20.29	0.70850	0.70817	0.00017	21.66	0.70809	0.70817	0.00014
20.25	0.70839	0.70820	0.00020	21.61	0.70821	0.70815	0.00015
20.21	0.70818	0.70820	0.00020	21.57	0.70810	0.70818	0.00016
20.16	0.70844	0.70826	0.00023	21.53	0.70785	0.70819	0.00016
20.12	0.70793	0.70830	0.00025	21.48	0.70843	0.70823	0.00015
20.08	0.70822	0.70834	0.00024	21.44	0.70790	0.70823	0.00015
20.03	0.70777	0.70836	0.00024	21.39	0.70810	0.70823	0.00015
19.99	0.70838	0.70838	0.00022	21.35	0.70851	0.70822	0.00015
19.95	0.70813	0.70839	0.00022	21.30	0.70837	0.70817	0.00014
19.90	0.70781	0.70841	0.00022	21.26	0.70813	0.70818	0.00015
19.86	0.70878	0.70845	0.00018	21.22	0.70790	0.70822	0.00016
19.81	0.70836	0.70839	0.00017	21.17	0.70851	0.70824	0.00015
19.77	0.70881	0.70839	0.00017	21.13	0.70816	0.70821	0.00013
19.73	0.70879	0.70834	0.00014	21.08	0.70834	0.70821	0.00013
19.68	0.70837	0.70828	0.00010	21.04	0.70842	0.70818	0.00013
19.64	0.70842	0.70830	0.00011	20.99	0.70788	0.70812	0.00014
19.60	0.70792	0.70830	0.00012	20.95	0.70801	0.70813	0.00013
19.55	0.70849	0.70834	0.00008	20.91	0.70802	0.70815	0.00013
19.51	0.70833	0.70832	0.00008	20.86	0.70841	0.70819	0.00014
19.47	0.70818	0.70830	0.00009	20.82	0.70852	0.70820	0.00015

19.42	0.70819	0.70833	0.00009
19.38	0.70839	0.70834	0.00009
19.33	0.70830	0.70834	0.00009
19.29	0.70823	0.70835	0.00009
19.25	0.70854	0.70831	0.00013
19.20	0.70847	0.70836	0.00019
19.16	0.70829	0.70840	0.00021
19.12	0.70827	0.70842	0.00021
19.07	0.70812	0.70845	0.00021
19.03	0.70852	0.70850	0.00020
18.99	0.70831	0.70852	0.00020
18.94	0.70838	0.70851	0.00021
18.90	0.70841	0.70849	0.00021
18.85	0.70785	0.70845	0.00024
18.81	0.70901	0.70857	0.00023
18.77	0.70886	0.70852	0.00021
18.72	0.70849	0.70852	0.00021
18.68	0.70860	0.70854	0.00021
18.64	0.70861	0.70855	0.00021
18.59	0.70866	0.70857	0.00022
18.55	0.70821	0.70855	0.00022
18.51	0.70824	0.70855	0.00022
18.46	0.70795	0.70858	0.00021
18.42	0.70910	0.70861	0.00018
18.37	0.70849	0.70850	0.00018
18.33	0.70885	0.70850	0.00018
18.29	0.70865	0.70845	0.00016
18.24	0.70874	0.70847	0.00018
18.20	0.70884	0.70844	0.00017
18.16	0.70845	0.70836	0.00016
18.11	0.70821	0.70835	0.00016
18.07	0.70854	0.70836	0.00016
18.02	0.70823	0.70833	0.00016
17.98	0.70803	0.70836	0.00016
17.94	0.70849	0.70838	0.00015
17.89	0.70828	0.70839	0.00016
17.85	0.70885	0.70840	0.00016
17.81	0.70852	0.70834	0.00013
17.76	0.70801	0.70830	0.00012
17.72	0.70836	0.70839	0.00015
17.68	0.70835	0.70838	0.00015
17.63	0.70817	0.70832	0.00020
17.59	0.70854	0.70835	0.00020
17.54	0.70821	0.70833	0.00020
17.50	0.70867	0.70835	0.00020
17.46	0.70837	0.70835	0.00020
17.41	0.70819	0.70834	0.00020
17.37	0.70818	0.70838	0.00020
17.33	0.70886	0.70837	0.00021
17.28	0.70826	0.70829	0.00018
20.77	0.70812	0.70823	0.00018
20.73	0.70822	0.70821	0.00019
20.68	0.70812	0.70821	0.00019
20.64	0.70803	0.70823	0.00019
20.60	0.70782	0.70828	0.00020
20.55	0.70805	0.70838	0.00019
20.51	0.70817	0.70839	0.00019
20.46	0.70845	0.70843	0.00018
20.42	0.70852	0.70841	0.00018
20.37	0.70881	0.70839	0.00018
20.33	0.70791	0.70838	0.00017
20.29	0.70824	0.70840	0.00015
20.24	0.70830	0.70839	0.00015
20.20	0.70856	0.70838	0.00016
20.15	0.70883	0.70835	0.00015
20.11	0.70813	0.70828	0.00011
20.06	0.70851	0.70830	0.00011
20.02	0.70834	0.70829	0.00011
19.98	0.70831	0.70828	0.00011
19.93	0.70865	0.70834	0.00015
19.89	0.70811	0.70828	0.00015
19.84	0.70820	0.70825	0.00017
19.80	0.70820	0.70824	0.00017
19.75	0.70820	0.70827	0.00019
19.71	0.70812	0.70827	0.00019
19.67	0.70839	0.70831	0.00020
19.62	0.70843	0.70825	0.00022
19.58	0.70824	0.70828	0.00024
19.53	0.70885	0.70828	0.00024
19.49	0.70804	0.70822	0.00020
19.44	0.70784	0.70821	0.00020
19.40	0.70805	0.70827	0.00019
19.36	0.70858	0.70832	0.00019
19.31	0.70812	0.70830	0.00018
19.27	0.70859	0.70833	0.00018
19.22	0.70777	0.70827	0.00018
19.18	0.70868	0.70838	0.00018
19.13	0.70829	0.70835	0.00017
19.09	0.70821	0.70835	0.00017
19.05	0.70798	0.70835	0.00017
19.00	0.70848	0.70836	0.00016
18.96	0.70848	0.70832	0.00016
18.91	0.70841	0.70828	0.00017
18.87	0.70837	0.70825	0.00017
18.82	0.70800	0.70821	0.00018
18.78	0.70891	0.70824	0.00017
18.74	0.70840	0.70819	0.00010
18.69	0.70823	0.70817	0.00009
18.65	0.70818	0.70818	0.00009
18.60	0.70809	0.70813	0.00012

17.24	0.70771	0.70827	0.00019
17.20	0.70849	0.70833	0.00014
17.15	0.70840	0.70835	0.00016
17.11	0.70832	0.70835	0.00016
17.06	0.70867	0.70836	0.00016
17.02	0.70832	0.70828	0.00016
16.98	0.70860	0.70826	0.00017
16.93	0.70804	0.70822	0.00015
16.89	0.70808	0.70827	0.00015
16.85	0.70806	0.70828	0.00015
16.80	0.70834	0.70830	0.00014
16.76	0.70870	0.70834	0.00017
16.72	0.70838	0.70829	0.00015
16.67	0.70839	0.70832	0.00018
16.63	0.70790	0.70831	0.00017
16.58	0.70811	0.70840	0.00016
16.54	0.70823	0.70840	0.00016
16.50	0.70848	0.70839	0.00017
16.45	0.70823	0.70839	0.00017
16.41	0.70824	0.70838	0.00017
16.37	0.70877	0.70842	0.00017
16.32	0.70813	0.70837	0.00015
16.28	0.70874	0.70838	0.00015
16.24	0.70832	0.70834	0.00012
16.19	0.70873	0.70839	0.00016
16.15	0.70812	0.70830	0.00017
16.10	0.70818	0.70833	0.00017
16.06	0.70843	0.70836	0.00017
16.02	0.70817	0.70835	0.00017
15.97	0.70860	0.70835	0.00017
15.93	0.70827	0.70832	0.00016
15.89	0.70826	0.70830	0.00016
15.84	0.70836	0.70827	0.00018
15.80	0.70882	0.70825	0.00018
15.76	0.70782	0.70826	0.00020
15.71	0.70843	0.70832	0.00018
15.67	0.70841	0.70832	0.00018
15.62	0.70840	0.70830	0.00018
15.58	0.70816	0.70831	0.00019
15.54	0.70823	0.70831	0.00019
15.49	0.70813	0.70833	0.00019
15.45	0.70788	0.70827	0.00024
15.41	0.70821	0.70833	0.00023
15.36	0.70895	0.70826	0.00029
15.32	0.70834	0.70825	0.00028
15.28	0.70847	0.70826	0.00028
15.23	0.70817	0.70826	0.00028
15.19	0.70856	0.70829	0.00028
15.14	0.70813	0.70827	0.00028
15.10	0.70844	0.70830	0.00028

18.56	0.70816	0.70813	0.00013
18.51	0.70806	0.70816	0.00015
18.47	0.70812	0.70816	0.00015
18.43	0.70794	0.70818	0.00015
18.38	0.70832	0.70821	0.00014
18.34	0.70842	0.70825	0.00017
18.29	0.70819	0.70822	0.00016
18.25	0.70827	0.70825	0.00017
18.20	0.70773	0.70825	0.00017
18.16	0.70807	0.70825	0.00017
18.12	0.70853	0.70827	0.00017
18.07	0.70806	0.70828	0.00017
18.03	0.70827	0.70830	0.00017
17.98	0.70826	0.70832	0.00017
17.94	0.70867	0.70836	0.00018
17.89	0.70813	0.70836	0.00018
17.85	0.70848	0.70835	0.00019
17.81	0.70830	0.70840	0.00022
17.76	0.70774	0.70840	0.00022
17.72	0.70832	0.70845	0.00017
17.67	0.70862	0.70845	0.00017
17.63	0.70824	0.70843	0.00017
17.58	0.70846	0.70843	0.00017
17.54	0.70865	0.70846	0.00018
17.50	0.70871	0.70840	0.00020
17.45	0.70802	0.70838	0.00019
17.41	0.70893	0.70839	0.00018
17.36	0.70831	0.70833	0.00013
17.32	0.70822	0.70829	0.00015
17.27	0.70833	0.70826	0.00017
17.23	0.70846	0.70822	0.00018
17.19	0.70821	0.70820	0.00018
17.14	0.70877	0.70821	0.00018
17.10	0.70800	0.70813	0.00014
17.05	0.70851	0.70815	0.00014
17.01	0.70820	0.70811	0.00011
16.96	0.70826	0.70814	0.00014
16.92	0.70796	0.70811	0.00015
16.88	0.70795	0.70816	0.00016
16.83	0.70788	0.70820	0.00016
16.79	0.70827	0.70823	0.00014
16.74	0.70835	0.70827	0.00016
16.70	0.70790	0.70824	0.00016
16.65	0.70820	0.70827	0.00014
16.61	0.70810	0.70829	0.00015
16.57	0.70858	0.70830	0.00014
16.52	0.70793	0.70825	0.00013
16.48	0.70844	0.70829	0.00011
16.43	0.70838	0.70826	0.00011
16.39	0.70818	0.70825	0.00010

15.06	0.70756	0.70832	0.00028
15.01	0.70851	0.70836	0.00024
14.97	0.70745	0.70831	0.00025
14.93	0.70882	0.70840	0.00016
14.88	0.70853	0.70833	0.00015
14.84	0.70846	0.70827	0.00016
14.80	0.70849	0.70820	0.00019
14.75	0.70830	0.70817	0.00018
14.71	0.70849	0.70812	0.00019
14.66	0.70857	0.70815	0.00022
14.62	0.70797	0.70816	0.00023
14.58	0.70798	0.70819	0.00023
14.53	0.70844	0.70822	0.00023
14.49	0.70812	0.70824	0.00024
14.45	0.70786	0.70833	0.00028
14.40	0.70774	0.70837	0.00026
14.36	0.70822	0.70844	0.00022
14.32	0.70779	0.70845	0.00022
14.27	0.70878	0.70850	0.00017
14.23	0.70869	0.70844	0.00017
14.18	0.70829	0.70839	0.00016
14.14	0.70825	0.70838	0.00017
14.10	0.70863	0.70835	0.00018
14.05	0.70902	0.70834	0.00018
14.01	0.70831	0.70829	0.00011
13.97	0.70845	0.70827	0.00011
13.92	0.70829	0.70827	0.00011
13.88	0.70827	0.70829	0.00011
13.84	0.70819	0.70825	0.00014
13.79	0.70822	0.70825	0.00014
13.75	0.70814	0.70826	0.00014
13.70	0.70799	0.70833	0.00017
13.66	0.70856	0.70840	0.00017
13.62	0.70844	0.70836	0.00017
13.57	0.70818	0.70839	0.00018
13.53	0.70845	0.70841	0.00018
13.49	0.70847	0.70840	0.00018
13.44	0.70787	0.70839	0.00018
13.40	0.70817	0.70845	0.00013
13.36	0.70836	0.70849	0.00012
13.31	0.70876	0.70849	0.00012
13.27	0.70870	0.70845	0.00011
13.22	0.70820	0.70842	0.00009
13.18	0.70870	0.70843	0.00009
13.14	0.70839	0.70839	0.00006
13.09	0.70844	0.70836	0.00008
13.05	0.70832	0.70834	0.00008
13.01	0.70845	0.70834	0.00008
12.96	0.70862	0.70832	0.00009
12.92	0.70830	0.70832	0.00008
16.34	0.70861	0.70826	0.00010
16.30	0.70812	0.70819	0.00009
16.26	0.70814	0.70824	0.00012
16.21	0.70845	0.70821	0.00015
16.17	0.70812	0.70821	0.00014
16.12	0.70812	0.70816	0.00018
16.08	0.70830	0.70817	0.00018
16.03	0.70818	0.70818	0.00018
15.99	0.70823	0.70817	0.00018
15.94	0.70831	0.70822	0.00021
15.90	0.70794	0.70824	0.00022
15.86	0.70863	0.70827	0.00021
15.81	0.70785	0.70820	0.00020
15.77	0.70839	0.70824	0.00018
15.72	0.70768	0.70825	0.00019
15.68	0.70819	0.70831	0.00014
15.63	0.70836	0.70830	0.00015
15.59	0.70815	0.70827	0.00015
15.55	0.70875	0.70829	0.00014
15.50	0.70844	0.70818	0.00015
15.46	0.70829	0.70817	0.00015
15.41	0.70795	0.70820	0.00016
15.37	0.70818	0.70823	0.00016
15.32	0.70848	0.70822	0.00016
15.28	0.70828	0.70823	0.00017
15.24	0.70807	0.70822	0.00017
15.19	0.70816	0.70824	0.00016
15.15	0.70830	0.70827	0.00017
15.10	0.70766	0.70827	0.00017
15.06	0.70838	0.70827	0.00016
15.01	0.70852	0.70829	0.00017
14.97	0.70829	0.70824	0.00016
14.93	0.70808	0.70827	0.00017
14.88	0.70859	0.70826	0.00018
14.84	0.70815	0.70822	0.00016
14.79	0.70828	0.70824	0.00016
14.75	0.70844	0.70825	0.00017
14.70	0.70830	0.70820	0.00017
14.66	0.70769	0.70820	0.00017
14.62	0.70853	0.70825	0.00012
14.57	0.70809	0.70818	0.00013
14.53	0.70851	0.70821	0.00014
14.48	0.70800	0.70814	0.00014
14.44	0.70820	0.70819	0.00015
14.39	0.70834	0.70819	0.00015
14.35	0.70839	0.70821	0.00016
14.31	0.70798	0.70824	0.00019
14.26	0.70824	0.70829	0.00018
14.22	0.70817	0.70831	0.00019
14.17	0.70784	0.70835	0.00019

12.87	0.70833	0.70833	0.00009	14.13	0.70841	0.70841	0.00015
12.83	0.70842	0.70837	0.00012	14.08	0.70783	0.70844	0.00016
12.79	0.70828	0.70839	0.00012	14.04	0.70845	0.70843	0.00017
12.74	0.70834	0.70836	0.00014	14.00	0.70825	0.70840	0.00018
12.70	0.70812	0.70841	0.00016	13.95	0.70851	0.70839	0.00018
12.66	0.70825	0.70840	0.00017	13.91	0.70875	0.70835	0.00019
12.61	0.70832	0.70842	0.00017	13.86	0.70845	0.70825	0.00021
12.57	0.70820	0.70840	0.00018	13.82	0.70849	0.70826	0.00021
12.53	0.70861	0.70837	0.00020	13.77	0.70854	0.70822	0.00021
12.48	0.70846	0.70837	0.00019	13.73	0.70844	0.70820	0.00020
12.44	0.70874	0.70836	0.00019	13.69	0.70866	0.70817	0.00019
12.39	0.70855	0.70830	0.00018	13.64	0.70775	0.70810	0.00016
12.35	0.70806	0.70829	0.00017	13.60	0.70813	0.70822	0.00022
12.31	0.70877	0.70831	0.00016	13.55	0.70820	0.70820	0.00022
12.26	0.70801	0.70830	0.00014	13.51	0.70811	0.70825	0.00024
12.22	0.70846	0.70839	0.00018	13.46	0.70772	0.70828	0.00024
12.18	0.70811	0.70839	0.00018	13.42	0.70861	0.70832	0.00021
12.13	0.70796	0.70839	0.00017	13.38	0.70806	0.70830	0.00020
12.09	0.70854	0.70844	0.00015	13.33	0.70829	0.70834	0.00020
12.05	0.70839	0.70845	0.00015	13.29	0.70817	0.70833	0.00020
12.00	0.70815	0.70848	0.00015	13.24	0.70796	0.70838	0.00020
11.96	0.70848	0.70846	0.00018	13.20	0.70894	0.70840	0.00019
11.91	0.70825	0.70848	0.00019	13.15	0.70798	0.70835	0.00015
11.87	0.70860	0.70844	0.00022	13.11	0.70864	0.70840	0.00012
11.83	0.70892	0.70846	0.00023	13.07	0.70847	0.70836	0.00011
11.78	0.70847	0.70841	0.00020	13.02	0.70808	0.70835	0.00011
11.74	0.70814	0.70837	0.00021	12.98	0.70845	0.70835	0.00011
11.65	0.70849	0.70834	0.00024	12.93	0.70837	0.70829	0.00014
11.61	0.70865	0.70830	0.00024	12.89	0.70828	0.70827	0.00014
11.57	0.70861	0.70823	0.00023	12.84	0.70866	0.70822	0.00017
11.52	0.70795	0.70821	0.00022	12.80	0.70812	0.70820	0.00015
11.48	0.70875	0.70828	0.00022	12.76	0.70849	0.70818	0.00015
11.43	0.70785	0.70825	0.00020	12.71	0.70841	0.70815	0.00014
11.39	0.70875	0.70831	0.00018	12.67	0.70826	0.70810	0.00013
11.35	0.70840	0.70828	0.00016	12.62	0.70838	0.70817	0.00021
11.30	0.70807	0.70827	0.00016	12.58	0.70804	0.70818	0.00021
11.26	0.70784	0.70832	0.00016	12.53	0.70789	0.70819	0.00021
11.22	0.70811	0.70839	0.00013	12.49	0.70818	0.70828	0.00022
11.17	0.70800	0.70843	0.00011	12.45	0.70781	0.70826	0.00023
11.13	0.70840	0.70843	0.00010	12.40	0.70838	0.70831	0.00020
11.09	0.70860	0.70844	0.00010	12.36	0.70796	0.70829	0.00020
11.04	0.70848	0.70844	0.00011	12.31	0.70817	0.70834	0.00019
11.00	0.70847	0.70836	0.00018	12.27	0.70796	0.70837	0.00019
10.95	0.70844	0.70839	0.00019	12.22	0.70892	0.70841	0.00017
10.91	0.70833	0.70839	0.00019	12.18	0.70848	0.70833	0.00013
10.87	0.70849	0.70840	0.00019	12.14	0.70818	0.70836	0.00016
10.82	0.70862	0.70840	0.00019	12.09	0.70871	0.70839	0.00015
10.78	0.70845	0.70837	0.00018	12.05	0.70803	0.70835	0.00013
10.74	0.70803	0.70835	0.00018	12.00	0.70830	0.70839	0.00011
10.69	0.70845	0.70839	0.00017	11.96	0.70821	0.70840	0.00011

10.65	0.70863	0.70841	0.00018
10.61	0.70772	0.70837	0.00018
10.56	0.70869	0.70838	0.00017
10.52	0.70847	0.70835	0.00015
10.47	0.70840	0.70834	0.00015
10.43	0.70849	0.70834	0.00015
10.39	0.70833	0.70830	0.00016
10.34	0.70832	0.70831	0.00016
10.30	0.70839	0.70830	0.00016
10.26	0.70871	0.70832	0.00018
10.21	0.70822	0.70828	0.00016
10.17	0.70777	0.70826	0.00017
10.13	0.70839	0.70830	0.00013
10.08	0.70837	0.70829	0.00013
10.04	0.70845	0.70825	0.00015
9.99	0.70802	0.70818	0.00017
9.95	0.70844	0.70821	0.00017
9.91	0.70822	0.70819	0.00016
9.86	0.70865	0.70823	0.00019
9.82	0.70831	0.70821	0.00017
9.78	0.70794	0.70821	0.00017
9.73	0.70824	0.70826	0.00016
9.69	0.70830	0.70828	0.00017
9.65	0.70789	0.70827	0.00017
9.60	0.70780	0.70832	0.00014
9.56	0.70836	0.70840	0.00009
9.51	0.70815	0.70837	0.00011
9.47	0.70865	0.70841	0.00011
9.43	0.70846	0.70839	0.00009
9.38	0.70834	0.70837	0.00009
9.34	0.70839	0.70845	0.00017
9.30	0.70843	0.70848	0.00018
9.25	0.70826	0.70844	0.00020
9.21	0.70841	0.70841	0.00022
9.17	0.70857	0.70842	0.00022
9.12	0.70805	0.70838	0.00023
9.08	0.70852	0.70843	0.00021
9.03	0.70844	0.70846	0.00023
8.99	0.70830	0.70846	0.00023
8.95	0.70911	0.70846	0.00023
8.90	0.70869	0.70841	0.00019
8.86	0.70802	0.70838	0.00018
8.82	0.70796	0.70840	0.00016
8.77	0.70857	0.70844	0.00013
8.73	0.70817	0.70842	0.00013
8.69	0.70849	0.70847	0.00012
8.64	0.70887	0.70847	0.00012
8.60	0.70839	0.70845	0.00010
8.55	0.70830	0.70847	0.00010
8.51	0.70864	0.70847	0.00010

11.91	0.70846	0.70836	0.00015
11.87	0.70847	0.70836	0.00015
11.83	0.70833	0.70836	0.00015
11.78	0.70815	0.70837	0.00015
11.74	0.70880	0.70840	0.00014
11.69	0.70842	0.70837	0.00011
11.65	0.70837	0.70835	0.00012
11.60	0.70838	0.70835	0.00012
11.56	0.70835	0.70832	0.00013
11.52	0.70787	0.70827	0.00016
11.47	0.70846	0.70834	0.00014
11.43	0.70844	0.70835	0.00015
11.38	0.70848	0.70834	0.00015
11.34	0.70840	0.70837	0.00017
11.29	0.70848	0.70838	0.00017
11.25	0.70827	0.70838	0.00017
11.21	0.70839	0.70837	0.00017
11.16	0.70809	0.70836	0.00017
11.12	0.70782	0.70840	0.00016
11.07	0.70857	0.70840	0.00016
11.03	0.70854	0.70841	0.00017
10.98	0.70836	0.70840	0.00017
10.94	0.70875	0.70841	0.00017
10.90	0.70849	0.70839	0.00015
10.85	0.70852	0.70839	0.00015
10.81	0.70821	0.70839	0.00015
10.76	0.70826	0.70840	0.00015
10.72	0.70847	0.70841	0.00015
10.67	0.70782	0.70838	0.00015
10.63	0.70865	0.70843	0.00009
10.59	0.70845	0.70842	0.00008
10.54	0.70854	0.70844	0.00010
10.50	0.70855	0.70842	0.00010
10.45	0.70843	0.70838	0.00011
10.41	0.70854	0.70839	0.00011
10.36	0.70827	0.70838	0.00011
10.32	0.70836	0.70837	0.00011
10.28	0.70819	0.70835	0.00012
10.23	0.70833	0.70835	0.00012
10.19	0.70856	0.70837	0.00012
10.14	0.70868	0.70833	0.00012
10.10	0.70833	0.70827	0.00011
10.05	0.70813	0.70826	0.00011
10.01	0.70848	0.70828	0.00010
9.97	0.70844	0.70827	0.00010
9.92	0.70824	0.70822	0.00011
9.88	0.70809	0.70819	0.00012
9.83	0.70825	0.70823	0.00013
9.79	0.70853	0.70824	0.00013
9.74	0.70815	0.70822	0.00011

8.47	0.70836	0.70847	0.00010
8.42	0.70821	0.70849	0.00010
8.38	0.70839	0.70852	0.00008
8.34	0.70841	0.70854	0.00007
8.29	0.70862	0.70854	0.00008
8.25	0.70855	0.70849	0.00010
8.21	0.70866	0.70843	0.00015
8.16	0.70857	0.70842	0.00015
8.12	0.70828	0.70839	0.00015
8.07	0.70861	0.70837	0.00016
8.03	0.70855	0.70836	0.00015
7.99	0.70859	0.70833	0.00015
7.94	0.70858	0.70834	0.00015
7.90	0.70833	0.70829	0.00014
7.86	0.70817	0.70831	0.00015
7.81	0.70791	0.70833	0.00015
7.77	0.70860	0.70840	0.00012
7.72	0.70825	0.70835	0.00012
7.68	0.70814	0.70834	0.00013
7.64	0.70850	0.70832	0.00015
7.59	0.70826	0.70831	0.00014
7.55	0.70861	0.70830	0.00015
7.51	0.70814	0.70831	0.00015
7.46	0.70852	0.70834	0.00015
7.42	0.70838	0.70833	0.00014
7.38	0.70858	0.70828	0.00017
7.33	0.70809	0.70828	0.00018
7.29	0.70823	0.70830	0.00018
7.24	0.70790	0.70835	0.00019
7.20	0.70838	0.70837	0.00017
7.16	0.70821	0.70840	0.00019
7.11	0.70865	0.70837	0.00021
7.07	0.70849	0.70833	0.00021
7.03	0.70838	0.70828	0.00021
6.98	0.70784	0.70829	0.00022
6.94	0.70866	0.70837	0.00020
6.90	0.70829	0.70836	0.00020
6.85	0.70868	0.70842	0.00022
6.81	0.70808	0.70837	0.00022
6.76	0.70876	0.70838	0.00021
6.72	0.70789	0.70831	0.00020
6.68	0.70824	0.70836	0.00018
6.63	0.70797	0.70837	0.00018
6.59	0.70852	0.70839	0.00016
6.55	0.70857	0.70841	0.00017
6.50	0.70863	0.70837	0.00017
6.46	0.70885	0.70834	0.00016
6.42	0.70815	0.70829	0.00012
6.37	0.70824	0.70832	0.00012
6.33	0.70801	0.70836	0.00013

9.70	0.70805	0.70818	0.00015
9.66	0.70821	0.70826	0.00020
9.61	0.70834	0.70832	0.00023
9.57	0.70837	0.70830	0.00023
9.52	0.70795	0.70833	0.00025
9.48	0.70798	0.70834	0.00024
9.43	0.70849	0.70843	0.00024
9.39	0.70830	0.70843	0.00024
9.35	0.70832	0.70838	0.00027
9.30	0.70774	0.70845	0.00030
9.26	0.70888	0.70850	0.00026
9.21	0.70881	0.70843	0.00025
9.17	0.70810	0.70838	0.00024
9.12	0.70870	0.70841	0.00023
9.08	0.70811	0.70837	0.00022
9.04	0.70887	0.70841	0.00021
8.99	0.70843	0.70837	0.00019
8.95	0.70786	0.70833	0.00019
8.90	0.70903	0.70836	0.00017
8.86	0.70820	0.70829	0.00008
8.81	0.70820	0.70831	0.00009
8.77	0.70833	0.70828	0.00012
8.73	0.70839	0.70827	0.00012
8.68	0.70832	0.70830	0.00015
8.64	0.70847	0.70828	0.00015
8.59	0.70847	0.70823	0.00016
8.55	0.70807	0.70817	0.00016
8.50	0.70815	0.70822	0.00018
8.46	0.70828	0.70828	0.00020
8.42	0.70846	0.70830	0.00021
8.37	0.70790	0.70827	0.00021
8.33	0.70814	0.70834	0.00020
8.28	0.70869	0.70838	0.00020
8.24	0.70816	0.70834	0.00019
8.19	0.70795	0.70833	0.00020
8.15	0.70789	0.70837	0.00018
8.11	0.70859	0.70843	0.00014
8.06	0.70871	0.70842	0.00014
8.02	0.70855	0.70836	0.00013
7.97	0.70814	0.70835	0.00012
7.93	0.70855	0.70830	0.00020
7.88	0.70863	0.70831	0.00021
7.84	0.70828	0.70824	0.00021
7.80	0.70805	0.70827	0.00022
7.75	0.70828	0.70834	0.00023
7.71	0.70855	0.70834	0.00023
7.66	0.70842	0.70830	0.00023
7.62	0.70818	0.70830	0.00023
7.57	0.70845	0.70831	0.00022
7.53	0.70758	0.70831	0.00022

6.28	0.70840	0.70840	0.00011
6.24	0.70833	0.70839	0.00011
6.20	0.70818	0.70835	0.00014
6.15	0.70870	0.70834	0.00015
6.11	0.70821	0.70828	0.00013
6.07	0.70829	0.70829	0.00013
6.02	0.70838	0.70831	0.00014
5.98	0.70847	0.70832	0.00014
5.94	0.70866	0.70829	0.00014
5.89	0.70837	0.70828	0.00014
5.85	0.70828	0.70826	0.00014
5.80	0.70800	0.70824	0.00014
5.76	0.70805	0.70826	0.00013
5.72	0.70805	0.70831	0.00013
5.67	0.70839	0.70828	0.00016
5.63	0.70847	0.70828	0.00016
5.59	0.70849	0.70823	0.00016
5.54	0.70813	0.70821	0.00015
5.50	0.70861	0.70819	0.00017
5.46	0.70816	0.70812	0.00014
5.41	0.70808	0.70811	0.00014
5.37	0.70817	0.70815	0.00016
5.32	0.70852	0.70818	0.00017
5.28	0.70779	0.70821	0.00021
5.24	0.70834	0.70830	0.00020
5.19	0.70803	0.70826	0.00020
5.15	0.70827	0.70827	0.00020
5.11	0.70787	0.70826	0.00020
5.06	0.70801	0.70831	0.00018
5.02	0.70806	0.70832	0.00018
4.98	0.70848	0.70837	0.00017
4.93	0.70844	0.70833	0.00018
4.89	0.70885	0.70832	0.00019
4.84	0.70862	0.70827	0.00014
4.80	0.70801	0.70827	0.00014
4.76	0.70809	0.70830	0.00013
4.71	0.70823	0.70831	0.00012
4.67	0.70834	0.70837	0.00015
4.63	0.70806	0.70838	0.00015
4.58	0.70854	0.70845	0.00013
4.54	0.70813	0.70844	0.00013
4.50	0.70842	0.70850	0.00013
4.45	0.70862	0.70847	0.00015
4.41	0.70827	0.70848	0.00015
4.36	0.70821	0.70849	0.00015
4.32	0.70875	0.70848	0.00015
4.28	0.70844	0.70844	0.00014
4.23	0.70866	0.70846	0.00015
4.19	0.70847	0.70842	0.00014
4.15	0.70882	0.70839	0.00015

7.49	0.70868	0.70833	0.00021
7.44	0.70791	0.70833	0.00021
7.40	0.70861	0.70840	0.00019
7.35	0.70869	0.70830	0.00024
7.31	0.70829	0.70827	0.00022
7.26	0.70818	0.70828	0.00022
7.22	0.70843	0.70826	0.00023
7.18	0.70831	0.70823	0.00023
7.13	0.70847	0.70820	0.00023
7.09	0.70771	0.70816	0.00023
7.04	0.70871	0.70825	0.00021
7.00	0.70858	0.70821	0.00019
6.95	0.70763	0.70819	0.00018
6.91	0.70841	0.70825	0.00013
6.87	0.70837	0.70823	0.00012
6.82	0.70796	0.70823	0.00012
6.78	0.70816	0.70827	0.00011
6.73	0.70802	0.70827	0.00011
6.69	0.70806	0.70828	0.00010
6.64	0.70856	0.70830	0.00009
6.60	0.70832	0.70831	0.00010
6.56	0.70844	0.70829	0.00011
6.51	0.70820	0.70825	0.00011
6.47	0.70818	0.70827	0.00011
6.42	0.70843	0.70828	0.00011
6.38	0.70830	0.70825	0.00010
6.33	0.70813	0.70827	0.00011
6.29	0.70819	0.70827	0.00011
6.25	0.70821	0.70831	0.00012
6.20	0.70867	0.70831	0.00012
6.16	0.70810	0.70826	0.00009
6.11	0.70813	0.70827	0.00009
6.07	0.70832	0.70831	0.00010
6.02	0.70827	0.70829	0.00010
5.98	0.70822	0.70834	0.00014
5.94	0.70843	0.70834	0.00014
5.89	0.70818	0.70836	0.00016
5.85	0.70857	0.70839	0.00015
5.80	0.70827	0.70838	0.00015
5.76	0.70812	0.70842	0.00015
5.71	0.70818	0.70845	0.00014
5.67	0.70854	0.70844	0.00015
5.63	0.70814	0.70839	0.00016
5.58	0.70879	0.70844	0.00016
5.54	0.70820	0.70839	0.00014
5.49	0.70866	0.70842	0.00014
5.45	0.70840	0.70838	0.00013
5.40	0.70851	0.70837	0.00013
5.36	0.70862	0.70836	0.00013
5.31	0.70844	0.70835	0.00012

4.10	0.70839	0.70839	0.00014	5.27	0.70806	0.70835	0.00012
4.06	0.70812	0.70841	0.00015	5.23	0.70807	0.70839	0.00011
4.02	0.70866	0.70846	0.00014	5.18	0.70860	0.70842	0.00008
3.97	0.70835	0.70845	0.00013	5.14	0.70830	0.70842	0.00007
3.93	0.70813	0.70844	0.00014	5.09	0.70855	0.70839	0.00011
3.88	0.70838	0.70845	0.00013	5.05	0.70821	0.70838	0.00010
3.84	0.70859	0.70847	0.00013	5.00	0.70832	0.70839	0.00009
3.80	0.70830	0.70843	0.00014	4.96	0.70845	0.70840	0.00009
3.75	0.70819	0.70843	0.00014	4.92	0.70852	0.70844	0.00013
3.71	0.70877	0.70846	0.00013	4.87	0.70839	0.70841	0.00013
3.67	0.70865	0.70841	0.00012	4.83	0.70849	0.70845	0.00015
3.62	0.70860	0.70839	0.00010	4.78	0.70841	0.70843	0.00015
3.58	0.70857	0.70841	0.00013	4.74	0.70853	0.70839	0.00017
3.54	0.70823	0.70842	0.00014	4.69	0.70802	0.70836	0.00017
3.49	0.70824	0.70844	0.00013	4.65	0.70843	0.70841	0.00016
3.45	0.70856	0.70847	0.00012	4.61	0.70834	0.70839	0.00016
3.40	0.70815	0.70848	0.00013	4.56	0.70843	0.70835	0.00019
3.36	0.70833	0.70850	0.00011	4.52	0.70882	0.70834	0.00019
3.32	0.70852	0.70852	0.00011	4.47	0.70821	0.70825	0.00017
3.27	0.70828	0.70855	0.00012	4.43	0.70877	0.70826	0.00017
3.23	0.70838	0.70857	0.00010	4.38	0.70830	0.70819	0.00013
3.19	0.70881	0.70860	0.00010	4.34	0.70804	0.70817	0.00013
3.14	0.70870	0.70858	0.00009	4.30	0.70819	0.70818	0.00013
3.10	0.70847	0.70858	0.00009	4.25	0.70858	0.70821	0.00014
3.06	0.70847	0.70860	0.00008	4.21	0.70826	0.70816	0.00012
3.01	0.70867	0.70859	0.00008	4.16	0.70790	0.70813	0.00012
2.97	0.70833	0.70856	0.00009	4.12	0.70835	0.70819	0.00013
2.92	0.70855	0.70857	0.00008	4.07	0.70792	0.70820	0.00014
2.88	0.70879	0.70853	0.00013	4.03	0.70831	0.70830	0.00018
2.84	0.70852	0.70846	0.00014	3.99	0.70808	0.70829	0.00018
2.79	0.70866	0.70841	0.00016	3.94	0.70804	0.70831	0.00017
2.75	0.70866	0.70835	0.00017	3.90	0.70820	0.70830	0.00018
2.71	0.70869	0.70829	0.00016	3.85	0.70844	0.70830	0.00018
2.66	0.70862	0.70826	0.00013	3.81	0.70805	0.70829	0.00018
2.62	0.70845	0.70827	0.00015	3.76	0.70798	0.70829	0.00018
2.57	0.70836	0.70827	0.00014	3.72	0.70853	0.70833	0.00016
2.53	0.70843	0.70826	0.00014	3.68	0.70847	0.70834	0.00016
2.49	0.70809	0.70823	0.00014	3.63	0.70887	0.70835	0.00017
2.44	0.70810	0.70825	0.00014	3.59	0.70823	0.70829	0.00013
2.40	0.70801	0.70828	0.00013	3.54	0.70827	0.70831	0.00013
2.36	0.70807	0.70829	0.00012	3.50	0.70793	0.70832	0.00013
2.31	0.70812	0.70833	0.00011	3.45	0.70824	0.70840	0.00012
2.27	0.70837	0.70833	0.00011	3.41	0.70831	0.70840	0.00012
2.23	0.70873	0.70835	0.00012	3.37	0.70808	0.70844	0.00013
2.18	0.70837	0.70836	0.00012	3.32	0.70841	0.70848	0.00010
2.14	0.70834	0.70838	0.00013	3.28	0.70854	0.70847	0.00010
2.09	0.70810	0.70839	0.00014	3.23	0.70860	0.70847	0.00010
2.05	0.70830	0.70836	0.00017	3.19	0.70832	0.70847	0.00010
2.01	0.70834	0.70835	0.00017	3.14	0.70837	0.70846	0.00011
1.96	0.70819	0.70834	0.00017	3.10	0.70839	0.70847	0.00011

1.92	0.70843	0.70836	0.00017
1.88	0.70817	0.70833	0.00018
1.83	0.70857	0.70834	0.00017
1.79	0.70874	0.70832	0.00017
1.75	0.70864	0.70825	0.00014
1.70	0.70788	0.70822	0.00011
1.66	0.70822	0.70826	0.00008
1.61	0.70823	0.70830	0.00010
1.57	0.70840	0.70833	0.00011
1.53	0.70809	0.70837	0.00015
1.48	0.70832	0.70836	0.00016
1.44	0.70839	0.70841	0.00017
1.40	0.70805	0.70837	0.00019
1.35	0.70836	0.70839	0.00018
1.31	0.70822	0.70837	0.00019
1.27	0.70832	0.70839	0.00018
1.22	0.70860	0.70840	0.00018
1.18	0.70857	0.70835	0.00018
1.13	0.70882	0.70838	0.00020
1.09	0.70800	0.70831	0.00018
1.05	0.70873	0.70840	0.00019
1.00	0.70801	0.70840	0.00020
0.96	0.70826	0.70845	0.00018
0.92	0.70818	0.70849	0.00018
0.87	0.70846	0.70853	0.00016
0.83	0.70838	0.70852	0.00017
0.79	0.70813	0.70854	0.00016
0.74	0.70882	0.70859	0.00013
0.70	0.70816	0.70855	0.00013
0.65	0.70884	0.70856	0.00011
0.61	0.70878	0.70853	0.00010
0.57	0.70850	0.70851	0.00008
0.52	0.70868	0.70850	0.00008
0.48	0.70860	0.70847	0.00008
0.44	0.70835	0.70846	0.00007
0.39	0.70857	0.70847	0.00007
0.35	0.70860	0.70845	0.00008
0.31	0.70841	0.70843	0.00007
0.26	0.70827	0.70844	0.00008
0.22	0.70858	0.70847	0.00006
0.17	0.70849	0.70844	0.00006
0.13	0.70848	0.70843	0.00007
0.09	0.70831	0.70840	0.00009
0.04	0.70850		

3.06	0.70873	0.70849	0.00011
3.01	0.70827	0.70847	0.00009
2.97	0.70867	0.70849	0.00008
2.92	0.70847	0.70841	0.00013
2.88	0.70837	0.70842	0.00013
2.83	0.70845	0.70842	0.00013
2.79	0.70867	0.70840	0.00013
2.75	0.70824	0.70839	0.00013
2.70	0.70845	0.70841	0.00012
2.66	0.70859	0.70841	0.00012
2.61	0.70852	0.70837	0.00013
2.57	0.70845	0.70835	0.00012
2.52	0.70794	0.70835	0.00012
2.48	0.70854	0.70840	0.00008
2.44	0.70832	0.70841	0.00009
2.39	0.70825	0.70844	0.00010
2.35	0.70859	0.70845	0.00009
2.30	0.70843	0.70844	0.00009
2.26	0.70846	0.70839	0.00012
2.21	0.70818	0.70839	0.00012
2.17	0.70832	0.70839	0.00011
2.13	0.70847	0.70840	0.00011
2.08	0.70841	0.70837	0.00012
2.04	0.70863	0.70836	0.00012
1.99	0.70865	0.70834	0.00011
1.95	0.70841	0.70831	0.00008
1.90	0.70841	0.70834	0.00011
1.86	0.70801	0.70830	0.00012
1.82	0.70838	0.70832	0.00010
1.77	0.70827	0.70833	0.00010
1.73	0.70840	0.70835	0.00011
1.68	0.70816	0.70832	0.00012
1.64	0.70832	0.70832	0.00012
1.59	0.70839	0.70832	0.00012
1.55	0.70839	0.70828	0.00013
1.51	0.70865	0.70831	0.00016
1.46	0.70806	0.70830	0.00015
1.42	0.70821	0.70831	0.00015
1.37	0.70842	0.70833	0.00015
1.33	0.70847	0.70833	0.00015
1.28	0.70811	0.70833	0.00015
1.24	0.70815	0.70835	0.00014
1.20	0.70834	0.70837	0.00013
1.15	0.70800	0.70836	0.00014
1.11	0.70873	0.70840	0.00011
1.06	0.70856	0.70840	0.00011
1.02	0.70814	0.70836	0.00011
0.97	0.70836	0.70840	0.00011
0.93	0.70846	0.70845	0.00013
0.89	0.70850	0.70843	0.00014

0.84	0.70827	0.70844	0.00014
0.80	0.70837	0.70846	0.00014
0.75	0.70817	0.70843	0.00016
0.71	0.70845	0.70846	0.00015
0.66	0.70873	0.70847	0.00015
0.62	0.70818	0.70843	0.00014
0.58	0.70854	0.70842	0.00015
0.53	0.70881	0.70841	0.00015
0.49	0.70824	0.70837	0.00012
0.44	0.70860	0.70840	0.00012
0.40	0.70851	0.70838	0.00012
0.35	0.70803	0.70836	0.00012
0.31	0.70847	0.70841	0.00009
0.27	0.70856	0.70840	0.00010
0.22	0.70834	0.70837	0.00010
0.18	0.70810	0.70837	0.00011
0.13	0.70847	0.70846	0.00002
0.09	0.70843	0.70846	0.00003
0.04	0.70849		

ARB 10.2.1 (M2)

Distance from cervix (mm)	$^{87}Sr/^{86}Sr$	10 point mov. average	2 SE on mov. average
42.47	0.70869	0.70910	0.00021
42.43	0.70928	0.70913	0.00020
42.38	0.70892	0.70911	0.00019
42.34	0.70938	0.70916	0.00020
42.30	0.70883	0.70909	0.00022
42.26	0.70916	0.70910	0.00021
42.21	0.70939	0.70902	0.00027
42.17	0.70937	0.70895	0.00026
42.13	0.70852	0.70893	0.00024
42.08	0.70947	0.70892	0.00025
42.04	0.70895	0.70883	0.00022
42.00	0.70909	0.70884	0.00023
41.96	0.70946	0.70879	0.00023
41.91	0.70862	0.70873	0.00017
41.87	0.70902	0.70876	0.00018
41.83	0.70830	0.70874	0.00017
41.78	0.70872	0.70874	0.00017
41.74	0.70911	0.70874	0.00017
41.70	0.70845	0.70870	0.00015
41.65	0.70861	0.70872	0.00014
41.61	0.70905	0.70877	0.00015
41.57	0.70853	0.70874	0.00014
41.53	0.70887	0.70882	0.00017
41.48	0.70895	0.70878	0.00018
41.44	0.70880	0.70876	0.00018
41.40	0.70831	0.70880	0.00020
41.35	0.70874	0.70886	0.00016
41.31	0.70873	0.70885	0.00017
41.27	0.70865	0.70888	0.00017
41.23	0.70907	0.70891	0.00016
41.18	0.70876	0.70889	0.00016
41.14	0.70932	0.70891	0.00016
41.10	0.70850	0.70891	0.00016
41.05	0.70876	0.70896	0.00013
41.01	0.70918	0.70901	0.00014
40.97	0.70892	0.70899	0.00013
40.93	0.70858	0.70895	0.00016
40.88	0.70901	0.70899	0.00014
40.84	0.70901	0.70896	0.00015
40.80	0.70885	0.70893	0.00015
40.75	0.70894	0.70895	0.00015
40.71	0.70933	0.70890	0.00019
40.67	0.70901	0.70883	0.00017
40.63	0.70926	0.70881	0.00016
40.58	0.70898	0.70878	0.00014
40.54	0.70853	0.70876	0.00013
40.50	0.70899	0.70882	0.00013

40.45	0.70871	0.70878	0.00014
40.41	0.70873	0.70884	0.00018
40.37	0.70904	0.70886	0.00018
40.33	0.70842	0.70888	0.00019
40.28	0.70863	0.70895	0.00016
40.24	0.70878	0.70892	0.00019
40.20	0.70902	0.70891	0.00020
40.15	0.70880	0.70887	0.00020
40.11	0.70907	0.70890	0.00021
40.07	0.70857	0.70883	0.00024
40.02	0.70935	0.70888	0.00023
39.98	0.70896	0.70882	0.00021
39.94	0.70920	0.70886	0.00023
39.90	0.70911	0.70885	0.00022
39.85	0.70832	0.70882	0.00022
39.81	0.70866	0.70887	0.00019
39.77	0.70869	0.70894	0.00020
39.72	0.70911	0.70895	0.00020
39.68	0.70828	0.70893	0.00020
39.64	0.70909	0.70903	0.00014
39.60	0.70880	0.70900	0.00015
39.55	0.70929	0.70901	0.00015
39.51	0.70911	0.70894	0.00015
39.47	0.70881	0.70892	0.00015
39.42	0.70890	0.70890	0.00017
39.38	0.70936	0.70893	0.00018
39.30	0.70879	0.70892	0.00017
39.25	0.70885	0.70893	0.00017
39.21	0.70930	0.70900	0.00021
39.17	0.70875	0.70893	0.00021
39.12	0.70891	0.70891	0.00022
39.08	0.70862	0.70893	0.00023
39.04	0.70896	0.70895	0.00022
39.00	0.70854	0.70898	0.00023
38.95	0.70921	0.70906	0.00021
38.91	0.70929	0.70904	0.00021
38.87	0.70882	0.70902	0.00020
38.82	0.70958	0.70901	0.00021
38.78	0.70860	0.70890	0.00019
38.74	0.70857	0.70903	0.00026
38.69	0.70912	0.70915	0.00028
38.65	0.70881	0.70913	0.00028
38.61	0.70929	0.70921	0.00029
38.57	0.70929	0.70920	0.00029
38.52	0.70901	0.70926	0.00032
38.48	0.70911	0.70926	0.00032
38.44	0.70869	0.70925	0.00032
38.39	0.70853	0.70926	0.00031
38.35	0.70989	0.70927	0.00031
38.31	0.70978	0.70917	0.00028

38.27	0.70887	0.70908	0.00025
38.22	0.70962	0.70910	0.00024
38.18	0.70926	0.70904	0.00021
38.14	0.70987	0.70901	0.00021
38.09	0.70894	0.70895	0.00010
38.05	0.70903	0.70896	0.00010
38.01	0.70882	0.70895	0.00010
37.97	0.70859	0.70895	0.00010
37.92	0.70889	0.70899	0.00006
37.88	0.70893	0.70906	0.00013
37.84	0.70902	0.70903	0.00015
37.79	0.70901	0.70899	0.00017
37.75	0.70902	0.70904	0.00020
37.71	0.70919	0.70910	0.00023
37.67	0.70906	0.70915	0.00026
37.62	0.70894	0.70915	0.00026
37.58	0.70888	0.70916	0.00025
37.54	0.70896	0.70916	0.00025
37.49	0.70957	0.70919	0.00025
37.45	0.70865	0.70917	0.00024
37.41	0.70862	0.70919	0.00023
37.37	0.70953	0.70926	0.00019
37.32	0.70964	0.70925	0.00018
37.28	0.70964	0.70916	0.00018
37.24	0.70907	0.70908	0.00016
37.19	0.70903	0.70905	0.00017
37.15	0.70890	0.70904	0.00017
37.11	0.70929	0.70912	0.00022
37.06	0.70936	0.70900	0.00030
37.02	0.70883	0.70894	0.00029
36.98	0.70928	0.70900	0.00030
36.94	0.70946	0.70900	0.00030
36.89	0.70877	0.70895	0.00029
36.85	0.70880	0.70891	0.00031
36.81	0.70880	0.70893	0.00031
36.76	0.70887	0.70896	0.00030
36.72	0.70971	0.70900	0.00031
36.68	0.70808	0.70893	0.00027
36.64	0.70875	0.70905	0.00020
36.59	0.70946	0.70911	0.00019
36.55	0.70930	0.70905	0.00018
36.51	0.70894	0.70902	0.00017
36.46	0.70840	0.70900	0.00018
36.42	0.70900	0.70909	0.00013
36.38	0.70906	0.70908	0.00013
36.34	0.70935	0.70913	0.00016
36.29	0.70901	0.70910	0.00015
36.25	0.70928	0.70906	0.00018
36.21	0.70929	0.70910	0.00021
36.16	0.70888	0.70901	0.00025

36.12	0.70901	0.70903	0.00025
36.08	0.70875	0.70906	0.00025
36.04	0.70931	0.70903	0.00027
35.99	0.70890	0.70901	0.00027
35.95	0.70952	0.70896	0.00029
35.91	0.70909	0.70891	0.00027
35.86	0.70864	0.70894	0.00028
35.82	0.70967	0.70900	0.00028
35.78	0.70839	0.70891	0.00024
35.73	0.70901	0.70894	0.00022
35.69	0.70930	0.70893	0.00022
35.65	0.70849	0.70887	0.00021
35.61	0.70910	0.70897	0.00022
35.56	0.70841	0.70894	0.00022
35.52	0.70901	0.70902	0.00020
35.48	0.70933	0.70898	0.00022
35.43	0.70932	0.70896	0.00021
35.39	0.70871	0.70890	0.00020
35.35	0.70871	0.70894	0.00019
35.31	0.70896	0.70892	0.00021
35.26	0.70863	0.70897	0.00024
35.22	0.70950	0.70899	0.00023
35.18	0.70883	0.70893	0.00020
35.13	0.70925	0.70893	0.00020
35.09	0.70853	0.70891	0.00019
35.05	0.70916	0.70893	0.00017
35.01	0.70876	0.70887	0.00018
34.96	0.70907	0.70891	0.00018
34.92	0.70853	0.70891	0.00018
34.88	0.70950	0.70898	0.00018
34.83	0.70879	0.70890	0.00014
34.79	0.70894	0.70887	0.00017
34.75	0.70877	0.70885	0.00017
34.71	0.70902	0.70886	0.00017
34.66	0.70875	0.70884	0.00016
34.62	0.70857	0.70884	0.00016
34.58	0.70918	0.70891	0.00017
34.53	0.70901	0.70887	0.00017
34.49	0.70929	0.70888	0.00017
34.45	0.70867	0.70886	0.00015
34.40	0.70848	0.70884	0.00018
34.36	0.70879	0.70887	0.00016
34.32	0.70884	0.70887	0.00016
34.28	0.70883	0.70891	0.00017
34.23	0.70876	0.70895	0.00019
34.19	0.70930	0.70903	0.00021
34.15	0.70876	0.70897	0.00021
34.10	0.70913	0.70900	0.00021
34.06	0.70908	0.70900	0.00021
34.02	0.70841	0.70901	0.00021

33.98	0.70878	0.70901	0.00022
33.93	0.70880	0.70899	0.00024
33.89	0.70923	0.70897	0.00024
33.85	0.70928	0.70893	0.00024
33.80	0.70954	0.70895	0.00025
33.76	0.70871	0.70886	0.00022
33.72	0.70904	0.70889	0.00021
33.68	0.70910	0.70889	0.00022
33.63	0.70924	0.70884	0.00022
33.59	0.70833	0.70879	0.00020
33.55	0.70857	0.70882	0.00018
33.50	0.70865	0.70883	0.00017
33.46	0.70887	0.70886	0.00017
33.42	0.70944	0.70887	0.00017
33.38	0.70864	0.70880	0.00012
33.33	0.70897	0.70881	0.00011
33.29	0.70910	0.70877	0.00011
33.25	0.70860	0.70879	0.00013
33.20	0.70876	0.70879	0.00013
33.16	0.70857	0.70882	0.00014
33.12	0.70867	0.70886	0.00013
33.08	0.70892	0.70889	0.00013
33.03	0.70899	0.70886	0.00015
32.99	0.70882	0.70887	0.00015
32.95	0.70868	0.70891	0.00017
32.90	0.70863	0.70894	0.00016
32.86	0.70924	0.70900	0.00016
32.82	0.70863	0.70899	0.00015
32.77	0.70904	0.70899	0.00015
32.73	0.70898	0.70894	0.00017
32.69	0.70900	0.70893	0.00017
32.65	0.70854	0.70894	0.00017
32.60	0.70909	0.70904	0.00018
32.56	0.70923	0.70906	0.00019
32.52	0.70898	0.70903	0.00019
32.47	0.70929	0.70905	0.00019
32.43	0.70912	0.70902	0.00018
32.39	0.70862	0.70901	0.00018
32.35	0.70857	0.70906	0.00016
32.30	0.70889	0.70912	0.00012
32.26	0.70907	0.70914	0.00010
32.22	0.70950	0.70914	0.00011
32.17	0.70929	0.70907	0.00009
32.13	0.70900	0.70903	0.00008
32.09	0.70917	0.70906	0.00009
32.05	0.70892	0.70903	0.00009
32.00	0.70910	0.70904	0.00008
31.96	0.70908	0.70905	0.00008
31.92	0.70923	0.70908	0.00011
31.87	0.70909	0.70910	0.00013

31.83	0.70897	0.70910	0.00013
31.79	0.70882	0.70912	0.00013
31.75	0.70896	0.70916	0.00011
31.70	0.70922	0.70917	0.00011
31.66	0.70891	0.70917	0.00011
31.62	0.70905	0.70923	0.00011
31.57	0.70914	0.70924	0.00011
31.53	0.70938	0.70924	0.00011
31.49	0.70949	0.70923	0.00010
31.44	0.70906	0.70919	0.00008
31.40	0.70916	0.70921	0.00008
31.36	0.70925	0.70917	0.00012
31.32	0.70907	0.70917	0.00012
31.27	0.70923	0.70915	0.00014
31.23	0.70951	0.70914	0.00014
31.19	0.70909	0.70912	0.00011
31.14	0.70916	0.70915	0.00013
31.10	0.70926	0.70921	0.00018
31.06	0.70918	0.70923	0.00018
30.97	0.70923	0.70927	0.00019
30.93	0.70875	0.70927	0.00019
30.89	0.70927	0.70931	0.00016
30.84	0.70885	0.70929	0.00016
30.80	0.70915	0.70934	0.00013
30.76	0.70923	0.70936	0.00013
30.72	0.70947	0.70935	0.00013
30.67	0.70975	0.70931	0.00014
30.63	0.70945	0.70925	0.00011
30.59	0.70951	0.70919	0.00011
30.54	0.70924	0.70913	0.00010
30.50	0.70918	0.70914	0.00011
30.46	0.70904	0.70919	0.00016
30.42	0.70934	0.70924	0.00016
30.37	0.70938	0.70927	0.00019
30.33	0.70918	0.70922	0.00020
30.29	0.70905	0.70924	0.00020
30.24	0.70908	0.70927	0.00020
30.20	0.70894	0.70930	0.00019
30.16	0.70887	0.70933	0.00018
30.12	0.70932	0.70937	0.00014
30.07	0.70969	0.70943	0.00017
30.03	0.70953	0.70938	0.00017
29.99	0.70968	0.70935	0.00017
29.94	0.70890	0.70931	0.00015
29.90	0.70935	0.70935	0.00012
29.86	0.70936	0.70933	0.00013
29.81	0.70934	0.70930	0.00013
29.77	0.70925	0.70937	0.00020
29.73	0.70932	0.70938	0.00020
29.69	0.70987	0.70939	0.00020

29.64	0.70922	0.70937	0.00017
29.60	0.70924	0.70940	0.00018
29.56	0.70928	0.70942	0.00017
29.51	0.70925	0.70948	0.00020
29.47	0.70914	0.70948	0.00020
29.43	0.70913	0.70950	0.00019
29.39	0.71004	0.70952	0.00018
29.34	0.70930	0.70953	0.00021
29.30	0.70946	0.70957	0.00020
29.26	0.70960	0.70956	0.00020
29.21	0.70957	0.70955	0.00020
29.17	0.70939	0.70957	0.00021
29.13	0.70994	0.70958	0.00021
29.09	0.70919	0.70954	0.00019
29.04	0.70938	0.70961	0.00018
29.00	0.70927	0.70954	0.00025
28.96	0.71023	0.70956	0.00025
28.91	0.70964	0.70947	0.00020
28.87	0.70940	0.70947	0.00020
28.83	0.70946	0.70950	0.00021
28.79	0.70979	0.70952	0.00021
28.74	0.70945	0.70947	0.00020
28.70	0.70961	0.70946	0.00021
28.66	0.70982	0.70949	0.00022
28.61	0.70870	0.70948	0.00022
28.57	0.70951	0.70957	0.00013
28.53	0.70929	0.70958	0.00013
28.48	0.70964	0.70961	0.00011
28.44	0.70971	0.70962	0.00011
28.40	0.70967	0.70957	0.00013
28.36	0.70933	0.70955	0.00013
28.31	0.70931	0.70953	0.00015
28.27	0.70990	0.70955	0.00014
28.23	0.70971	0.70950	0.00012
28.18	0.70966	0.70947	0.00011
28.14	0.70956	0.70948	0.00011
28.10	0.70964	0.70944	0.00012
28.06	0.70969	0.70947	0.00016
28.01	0.70924	0.70942	0.00016
27.97	0.70943	0.70944	0.00016
27.93	0.70919	0.70942	0.00017
27.88	0.70950	0.70941	0.00017
27.84	0.70939	0.70942	0.00017
27.80	0.70940	0.70938	0.00019
27.76	0.70971	0.70936	0.00020
27.71	0.70920	0.70930	0.00019
27.67	0.70997	0.70929	0.00019
27.63	0.70915	0.70924	0.00012
27.58	0.70950	0.70924	0.00013
27.54	0.70917	0.70922	0.00012

27.50	0.70916	0.70923	0.00012
27.46	0.70956	0.70927	0.00013
27.41	0.70899	0.70917	0.00018
27.37	0.70917	0.70922	0.00019
27.33	0.70910	0.70923	0.00019
27.28	0.70913	0.70927	0.00019
27.24	0.70948	0.70933	0.00021
27.20	0.70910	0.70938	0.00024
27.16	0.70939	0.70945	0.00025
27.11	0.70925	0.70946	0.00025
27.07	0.70956	0.70942	0.00027
27.03	0.70854	0.70943	0.00027
26.98	0.70949	0.70951	0.00019
26.94	0.70922	0.70948	0.00021
26.90	0.70951	0.70949	0.00020
26.85	0.70976	0.70949	0.00020
26.81	0.70994	0.70949	0.00020
26.77	0.70979	0.70947	0.00019
26.73	0.70955	0.70945	0.00017
26.68	0.70888	0.70942	0.00018
26.64	0.70962	0.70948	0.00013
26.60	0.70935	0.70944	0.00013
26.55	0.70915	0.70946	0.00013
26.51	0.70937	0.70949	0.00011
26.47	0.70951	0.70951	0.00011
26.43	0.70976	0.70949	0.00012
26.38	0.70976	0.70949	0.00012
26.34	0.70950	0.70947	0.00010
26.30	0.70927	0.70946	0.00010
26.25	0.70953	0.70950	0.00010
26.21	0.70923	0.70946	0.00012
26.17	0.70955	0.70948	0.00011
26.13	0.70940	0.70950	0.00012
26.08	0.70959	0.70950	0.00012
26.04	0.70933	0.70952	0.00013
26.00	0.70975	0.70951	0.00014
25.95	0.70954	0.70949	0.00013
25.91	0.70944	0.70952	0.00015
25.87	0.70962	0.70949	0.00017
25.83	0.70915	0.70945	0.00018
25.78	0.70942	0.70953	0.00019
25.74	0.70974	0.70948	0.00023
25.70	0.70939	0.70945	0.00022
25.65	0.70980	0.70949	0.00023
25.61	0.70922	0.70947	0.00022
25.57	0.70953	0.70950	0.00022
25.52	0.70989	0.70951	0.00022
25.48	0.70916	0.70946	0.00020
25.44	0.70921	0.70949	0.00019
25.40	0.70997	0.70954	0.00019

25.35	0.70886	0.70949	0.00016
25.31	0.70949	0.70960	0.00012
25.27	0.70974	0.70959	0.00012
25.22	0.70959	0.70955	0.00012
25.18	0.70958	0.70958	0.00015
25.14	0.70965	0.70955	0.00016
25.10	0.70940	0.70955	0.00016
25.05	0.70941	0.70958	0.00016
25.01	0.70974	0.70958	0.00015
24.97	0.70941	0.70957	0.00015
24.92	0.70996	0.70954	0.00017
24.88	0.70939	0.70946	0.00015
24.84	0.70936	0.70950	0.00016
24.80	0.70995	0.70952	0.00016
24.75	0.70927	0.70946	0.00013
24.71	0.70966	0.70944	0.00015
24.67	0.70961	0.70945	0.00015
24.62	0.70948	0.70943	0.00015
24.58	0.70957	0.70944	0.00015
24.54	0.70919	0.70948	0.00018
24.50	0.70916	0.70947	0.00019
24.45	0.70979	0.70945	0.00020
24.41	0.70949	0.70942	0.00019
24.37	0.70937	0.70947	0.00023
24.32	0.70911	0.70950	0.00023
24.28	0.70973	0.70954	0.00021
24.24	0.70941	0.70952	0.00021
24.20	0.70960	0.70960	0.00024
24.15	0.70995	0.70956	0.00024
24.11	0.70908	0.70953	0.00023
24.07	0.70899	0.70961	0.00022
24.02	0.70944	0.70964	0.00019
23.98	0.71002	0.70967	0.00018
23.94	0.70964	0.70960	0.00017
23.89	0.70951	0.70954	0.00021
23.85	0.70957	0.70954	0.00021
23.81	0.71014	0.70957	0.00022
23.77	0.70930	0.70950	0.00018
23.72	0.70961	0.70963	0.00027
23.68	0.70991	0.70961	0.00027
23.64	0.70927	0.70959	0.00026
23.59	0.70969	0.70957	0.00028
23.55	0.70934	0.70954	0.00028
23.51	0.70902	0.70957	0.00028
23.47	0.70959	0.70961	0.00026
23.42	0.70987	0.70959	0.00026
23.38	0.70944	0.70955	0.00025
23.34	0.71055	0.70961	0.00027
23.29	0.70946	0.70950	0.00017
23.25	0.70971	0.70950	0.00017

23.21	0.70903	0.70949	0.00016
23.17	0.70934	0.70956	0.00013
23.12	0.70966	0.70957	0.00013
23.08	0.70941	0.70961	0.00016
23.04	0.70942	0.70957	0.00020
22.99	0.70949	0.70956	0.00020
22.95	0.71004	0.70957	0.00020
22.91	0.70945	0.70954	0.00017
22.87	0.70947	0.70955	0.00017
22.82	0.70960	0.70950	0.00021
22.78	0.70974	0.70952	0.00022
22.74	0.70942	0.70950	0.00021
22.69	0.71005	0.70951	0.00021
22.65	0.70898	0.70944	0.00017
22.61	0.70940	0.70947	0.00014
22.56	0.70959	0.70950	0.00015
22.52	0.70966	0.70954	0.00017
22.48	0.70955	0.70949	0.00018
22.39	0.70898	0.70948	0.00018
22.35	0.70980	0.70952	0.00015
22.31	0.70960	0.70947	0.00014
22.26	0.70945	0.70943	0.00014
22.22	0.70942	0.70940	0.00015
22.18	0.70928	0.70942	0.00016
22.14	0.70970	0.70944	0.00016
22.09	0.70993	0.70944	0.00016
22.05	0.70924	0.70940	0.00012
22.01	0.70939	0.70946	0.00013
21.96	0.70936	0.70946	0.00013
21.92	0.70931	0.70953	0.00017
21.88	0.70920	0.70954	0.00016
21.84	0.70915	0.70959	0.00015
21.79	0.70965	0.70963	0.00011
21.75	0.70949	0.70961	0.00011
21.71	0.70972	0.70960	0.00012
21.66	0.70953	0.70956	0.00013
21.62	0.70976	0.70958	0.00014
21.58	0.70946	0.70955	0.00013
21.54	0.71002	0.70955	0.00013
21.49	0.70947	0.70947	0.00010
21.45	0.70966	0.70950	0.00012
21.41	0.70950	0.70950	0.00012
21.36	0.70954	0.70951	0.00012
21.32	0.70939	0.70949	0.00013
21.28	0.70927	0.70947	0.00014
21.24	0.70975	0.70951	0.00014
21.19	0.70945	0.70946	0.00014
21.15	0.70949	0.70952	0.00018
21.11	0.70918	0.70951	0.00019
21.06	0.70977	0.70953	0.00018

21.02	0.70965	0.70952	0.00017
20.98	0.70965	0.70949	0.00017
20.93	0.70931	0.70947	0.00017
20.89	0.70922	0.70950	0.00016
20.85	0.70965	0.70950	0.00016
20.81	0.70921	0.70950	0.00016
20.76	0.71008	0.70951	0.00015
20.72	0.70934	0.70943	0.00009
20.68	0.70938	0.70940	0.00012
20.63	0.70965	0.70939	0.00012
20.59	0.70936	0.70934	0.00012
20.55	0.70948	0.70936	0.00013
20.51	0.70960	0.70931	0.00015
20.46	0.70925	0.70925	0.00015
20.42	0.70959	0.70926	0.00015
20.38	0.70932	0.70924	0.00013
20.33	0.70931	0.70923	0.00014
20.29	0.70903	0.70926	0.00015
20.25	0.70927	0.70926	0.00015
20.21	0.70914	0.70931	0.00017
20.16	0.70963	0.70930	0.00017
20.12	0.70893	0.70926	0.00016
20.08	0.70904	0.70935	0.00016
20.03	0.70930	0.70941	0.00015
19.99	0.70943	0.70941	0.00016
19.95	0.70943	0.70937	0.00017
19.91	0.70953	0.70935	0.00017
19.86	0.70909	0.70939	0.00016
19.82	0.70967	0.70935	0.00015
19.78	0.70909	0.70939	0.00014
19.73	0.70927	0.70936	0.00016
19.69	0.70975	0.70933	0.00013
19.65	0.70960	0.70928	0.00012
19.60	0.70925	0.70931	0.00013
19.56	0.70951	0.70929	0.00012
19.52	0.70898	0.70930	0.00011
19.48	0.70926	0.70931	0.00011
19.43	0.70954	0.70923	0.00015
19.39	0.70925	0.70923	0.00015
19.35	0.70947	0.70918	0.00015
19.30	0.70902	0.70920	0.00015
19.26	0.70938	0.70917	0.00015
19.22	0.70915	0.70921	0.00017
19.18	0.70952	0.70921	0.00017
19.13	0.70928	0.70924	0.00019
19.09	0.70911	0.70925	0.00019
19.05	0.70935	0.70923	0.00019
19.00	0.70872	0.70926	0.00015
18.96	0.70934	0.70924	0.00016
18.92	0.70895	0.70927	0.00014

18.88	0.70920	0.70928	0.00014
18.83	0.70904	0.70928	0.00014
18.79	0.70961	0.70923	0.00013
18.75	0.70948	0.70921	0.00012
18.70	0.70964	0.70918	0.00008
18.66	0.70916	0.70921	0.00010
18.62	0.70914	0.70924	0.00010
18.58	0.70906	0.70928	0.00011
18.53	0.70910	0.70934	0.00013
18.49	0.70924	0.70937	0.00014
18.45	0.70935	0.70939	0.00014
18.40	0.70904	0.70943	0.00012
18.36	0.70903	0.70947	0.00008
18.32	0.70937	0.70952	0.00010
18.27	0.70932	0.70951	0.00010
18.23	0.70947	0.70956	0.00013
18.19	0.70937	0.70957	0.00012
18.15	0.70947	0.70959	0.00012
18.10	0.70975	0.70954	0.00013
18.06	0.70952	0.70954	0.00013
18.02	0.70951	0.70955	0.00013
17.97	0.70953	0.70956	0.00013
17.93	0.70940	0.70961	0.00014
17.89	0.70981	0.70961	0.00014
17.85	0.70929	0.70966	0.00013
17.80	0.70991	0.70962	0.00011
17.76	0.70953	0.70967	0.00014
17.72	0.70962	0.70968	0.00014
17.67	0.70926	0.70970	0.00012
17.63	0.70952	0.70972	0.00011
17.59	0.70963	0.70972	0.00011
17.55	0.70967	0.70969	0.00013
17.50	0.70992	0.70966	0.00012
17.46	0.70978	0.70964	0.00012
17.42	0.70977	0.70966	0.00013
17.37	0.70956	0.70965	0.00013
17.33	0.71002	0.70961	0.00011
17.29	0.70971	0.70963	0.00012
17.25	0.70940	0.70967	0.00011
17.20	0.70977	0.70966	0.00011
17.16	0.70962	0.70968	0.00011
17.12	0.70937	0.70973	0.00009
17.07	0.70955	0.70975	0.00009
17.03	0.70965	0.70972	0.00012
16.99	0.70993	0.70971	0.00011
16.95	0.70950	0.70978	0.00013
16.90	0.70963	0.70979	0.00012
16.86	0.70992	0.70971	0.00018
16.82	0.70974	0.70973	0.00019
16.77	0.70973	0.70973	0.00019

16.73	0.70979	0.70970	0.00019
16.69	0.70985	0.70969	0.00019
16.64	0.70977	0.70968	0.00019
16.60	0.70935	0.70977	0.00020
16.56	0.70984	0.70975	0.00020
16.52	0.71014	0.70971	0.00018
16.47	0.70981	0.70968	0.00018
16.43	0.70912	0.70973	0.00013
16.39	0.70995	0.70970	0.00012
16.34	0.70966	0.70971	0.00012
16.30	0.70951	0.70974	0.00011
16.26	0.70973	0.70973	0.00011
16.22	0.70971	0.70974	0.00012
16.17	0.71020	0.70968	0.00006
16.13	0.70970	0.70967	0.00006
16.09	0.70970	0.70968	0.00007
16.04	0.70952	0.70969	0.00006
16.00	0.70959	0.70971	0.00007
15.96	0.70974	0.70967	0.00011
15.92	0.70975	0.70970	0.00013
15.87	0.70976	0.70967	0.00014
15.83	0.70964	0.70965	0.00015
15.79	0.70984	0.70964	0.00015
15.74	0.70954	0.70963	0.00015
15.70	0.70965	0.70962	0.00015
15.66	0.70977	0.70960	0.00015
15.62	0.70958	0.70961	0.00015
15.57	0.70988	0.70958	0.00013
15.53	0.70927	0.70962	0.00012
15.49	0.71005	0.70960	0.00008
15.44	0.70948	0.70964	0.00009
15.40	0.70942	0.70965	0.00008
15.36	0.70977	0.70964	0.00008
15.31	0.70947	0.70965	0.00007
15.27	0.70955	0.70968	0.00007
15.23	0.70957	0.70970	0.00007
15.19	0.70966	0.70970	0.00007
15.14	0.70956	0.70971	0.00006
15.10	0.70972	0.70970	0.00007
15.06	0.70980	0.70968	0.00007
15.01	0.70984	0.70965	0.00006
14.97	0.70952	0.70970	0.00008
14.93	0.70969	0.70969	0.00009
14.89	0.70964	0.70972	0.00010
14.84	0.70979	0.70966	0.00015
14.80	0.70982	0.70966	0.00015
14.76	0.70968	0.70963	0.00016
14.71	0.70965	0.70965	0.00016
14.67	0.70959	0.70965	0.00016
14.63	0.70958	0.70963	0.00017

14.59	0.70956	0.70965	0.00017
14.54	0.70999	0.70961	0.00015
14.50	0.70961	0.70961	0.00015
14.46	0.70996	0.70960	0.00015
14.41	0.70915	0.70963	0.00012
14.37	0.70984	0.70961	0.00011
14.33	0.70942	0.70964	0.00011
14.29	0.70982	0.70962	0.00010
14.24	0.70954	0.70968	0.00013
14.20	0.70942	0.70973	0.00013
14.16	0.70974	0.70969	0.00015
14.11	0.70960	0.70971	0.00015
14.07	0.70958	0.70969	0.00017
14.03	0.70991	0.70969	0.00016
13.99	0.70940	0.70970	0.00016
13.94	0.70966	0.70970	0.00016
13.90	0.70975	0.70967	0.00017
13.86	0.70963	0.70967	0.00017
13.81	0.71010	0.70961	0.00014
13.77	0.70997	0.70960	0.00013
13.73	0.70934	0.70967	0.00014
13.68	0.70977	0.70966	0.00014
13.64	0.70938	0.70974	0.00017
13.60	0.70987	0.70971	0.00017
13.56	0.70950	0.70971	0.00017
13.51	0.70969	0.70976	0.00019
13.47	0.70941	0.70981	0.00017
13.43	0.70968	0.70979	0.00018
13.38	0.70947	0.70982	0.00017
13.34	0.70989	0.70979	0.00018
13.30	0.71005	0.70973	0.00018
13.26	0.70963	0.70976	0.00018
13.21	0.71022	0.70966	0.00017
13.17	0.70957	0.70968	0.00017
13.13	0.70949	0.70970	0.00017
13.08	0.71016	0.70965	0.00014
13.04	0.70992	0.70959	0.00013
13.00	0.70952	0.70962	0.00014
12.96	0.70980	0.70965	0.00017
12.91	0.70957	0.70965	0.00017
12.87	0.70947	0.70968	0.00017
12.83	0.70989	0.70962	0.00018
12.78	0.70922	0.70966	0.00016
12.74	0.70978	0.70965	0.00015
12.70	0.70967	0.70966	0.00016
12.66	0.70963	0.70967	0.00016
12.61	0.70935	0.70969	0.00014
12.57	0.70978	0.70969	0.00014
12.53	0.71011	0.70962	0.00012
12.48	0.70955	0.70966	0.00013

12.44	0.70985	0.70962	0.00012
12.40	0.70925	0.70965	0.00009
12.35	0.70961	0.70962	0.00012
12.31	0.70972	0.70957	0.00014
12.27	0.70976	0.70956	0.00013
12.23	0.70969	0.70956	0.00013
12.18	0.70954	0.70960	0.00015
12.14	0.70980	0.70957	0.00015
12.10	0.70944	0.70954	0.00017
12.05	0.70990	0.70952	0.00015
12.01	0.70948	0.70954	0.00016
11.97	0.70960	0.70951	0.00016
11.93	0.70928	0.70953	0.00016
11.88	0.70923	0.70955	0.00014
11.84	0.70961	0.70953	0.00015
11.80	0.70970	0.70951	0.00015
11.75	0.70992	0.70946	0.00011
11.71	0.70957	0.70945	0.00011
11.67	0.70912	0.70946	0.00010
11.63	0.70965	0.70943	0.00009
11.58	0.70971	0.70939	0.00006
11.54	0.70929	0.70941	0.00006
11.50	0.70951	0.70938	0.00006
11.45	0.70942	0.70935	0.00008
11.41	0.70944	0.70927	0.00012
11.37	0.70944	0.70925	0.00012
11.33	0.70939	0.70925	0.00012
11.28	0.70951	0.70922	0.00014
11.24	0.70925	0.70918	0.00014
11.20	0.70938	0.70913	0.00015
11.15	0.70927	0.70907	0.00014
11.11	0.70948	0.70905	0.00013
11.07	0.70931	0.70906	0.00014
11.03	0.70914	0.70909	0.00013
10.98	0.70887	0.70905	0.00015
10.94	0.70914	0.70906	0.00015
10.90	0.70915	0.70907	0.00016
10.85	0.70951	0.70911	0.00016
10.81	0.70891	0.70910	0.00016
10.77	0.70897	0.70914	0.00015
10.72	0.70883	0.70918	0.00014
10.68	0.70891	0.70918	0.00014
10.64	0.70903	0.70917	0.00014
10.60	0.70932	0.70914	0.00015
10.55	0.70917	0.70920	0.00012
10.51	0.70875	0.70921	0.00013
10.47	0.70927	0.70920	0.00011
10.42	0.70956	0.70919	0.00011
10.38	0.70926	0.70920	0.00010
10.34	0.70895	0.70922	0.00010

10.30	0.70915	0.70924	0.00012
10.25	0.70931	0.70925	0.00011
10.21	0.70907	0.70927	0.00011
10.17	0.70916	0.70931	0.00008
10.12	0.70894	0.70931	0.00008
10.08	0.70935	0.70928	0.00009
10.04	0.70939	0.70931	0.00013
10.00	0.70945	0.70931	0.00013
9.95	0.70909	0.70932	0.00012
9.91	0.70915	0.70931	0.00013
9.87	0.70928	0.70924	0.00015
9.82	0.70949	0.70924	0.00015
9.78	0.70922	0.70925	0.00015
9.74	0.70937	0.70925	0.00015
9.70	0.70930	0.70923	0.00015
9.65	0.70940	0.70925	0.00015
9.61	0.70909	0.70918	0.00011
9.57	0.70973	0.70918	0.00011
9.52	0.70908	0.70917	0.00011
9.48	0.70924	0.70917	0.00011
9.44	0.70914	0.70924	0.00011
9.39	0.70884	0.70924	0.00011
9.35	0.70923	0.70921	0.00010
9.31	0.70946	0.70921	0.00010
9.27	0.70928	0.70921	0.00010
9.22	0.70917	0.70914	0.00015
9.18	0.70935	0.70912	0.00016
9.14	0.70904	0.70913	0.00016
9.09	0.70904	0.70913	0.00016
9.05	0.70910	0.70911	0.00016
9.01	0.70918	0.70909	0.00014
8.97	0.70955	0.70911	0.00016
8.92	0.70923	0.70912	0.00016
8.88	0.70917	0.70910	0.00016
8.84	0.70926	0.70904	0.00018
8.79	0.70921	0.70910	0.00014
8.75	0.70857	0.70911	0.00014
8.71	0.70889	0.70911	0.00014
8.67	0.70913	0.70912	0.00014
8.62	0.70908	0.70915	0.00014
8.58	0.70897	0.70913	0.00013
8.54	0.70937	0.70910	0.00011
8.49	0.70945	0.70910	0.00011
8.45	0.70923	0.70911	0.00011
8.41	0.70909	0.70915	0.00005
8.37	0.70866	0.70913	0.00005
8.32	0.70917	0.70914	0.00005
8.28	0.70899	0.70917	0.00006
8.24	0.70910	0.70915	0.00007
8.19	0.70917	0.70910	0.00010

8.15	0.70926	0.70911	0.00011
8.11	0.70920	0.70908	0.00012
8.07	0.70915	0.70907	0.00012
8.02	0.70917	0.70906	0.00011
7.98	0.70920	0.70905	0.00012
7.94	0.70905	0.70903	0.00012
7.89	0.70905	0.70906	0.00014
7.85	0.70907	0.70902	0.00012
7.81	0.70935	0.70904	0.00013
7.76	0.70902	0.70907	0.00011
7.72	0.70876	0.70907	0.00012
7.68	0.70932	0.70914	0.00014
7.64	0.70886	0.70915	0.00014
7.59	0.70905	0.70917	0.00014
7.55	0.70910	0.70916	0.00014
7.51	0.70891	0.70917	0.00014
7.46	0.70888	0.70918	0.00014
7.42	0.70936	0.70919	0.00013
7.38	0.70896	0.70920	0.00014
7.34	0.70920	0.70921	0.00014
7.29	0.70904	0.70922	0.00014
7.25	0.70935	0.70918	0.00013
7.21	0.70953	0.70916	0.00014
7.16	0.70916	0.70914	0.00014
7.12	0.70928	0.70918	0.00012
7.08	0.70884	0.70919	0.00011
7.04	0.70899	0.70916	0.00010
6.99	0.70944	0.70916	0.00010
6.95	0.70909	0.70915	0.00010
6.91	0.70932	0.70914	0.00011
6.86	0.70906	0.70909	0.00008
6.82	0.70948	0.70909	0.00008
6.78	0.70916	0.70909	0.00008
6.74	0.70892	0.70906	0.00010
6.69	0.70908	0.70904	0.00009
6.65	0.70922	0.70903	0.00009
6.61	0.70911	0.70904	0.00010
6.56	0.70910	0.70907	0.00011
6.52	0.70910	0.70905	0.00010
6.48	0.70929	0.70905	0.00010
6.43	0.70889	0.70905	0.00010
6.39	0.70900	0.70901	0.00011
6.35	0.70919	0.70902	0.00010
6.31	0.70890	0.70905	0.00009
6.26	0.70881	0.70904	0.00010
6.22	0.70897	0.70901	0.00011
6.18	0.70910	0.70901	0.00010
6.13	0.70920	0.70900	0.00010
6.09	0.70932	0.70900	0.00009
6.05	0.70910	0.70901	0.00010

6.01	0.70897	0.70905	0.00011
5.96	0.70891	0.70908	0.00009
5.92	0.70881	0.70907	0.00010
5.88	0.70902	0.70908	0.00010
5.83	0.70912	0.70907	0.00010
5.79	0.70882	0.70908	0.00010
5.75	0.70886	0.70904	0.00011
5.71	0.70915	0.70900	0.00011
5.66	0.70925	0.70900	0.00011
5.62	0.70905	0.70898	0.00010
5.58	0.70914	0.70896	0.00008
5.53	0.70929	0.70894	0.00007
5.49	0.70907	0.70895	0.00008
5.45	0.70897	0.70892	0.00007
5.41	0.70915	0.70896	0.00008
5.36	0.70880	0.70896	0.00009
5.32	0.70889	0.70896	0.00008
5.28	0.70877	0.70899	0.00008
5.23	0.70887	0.70899	0.00008
5.19	0.70904	0.70900	0.00008
5.15	0.70894	0.70899	0.00008
5.10	0.70906	0.70897	0.00010
5.06	0.70890	0.70895	0.00010
5.02	0.70907	0.70894	0.00011
4.98	0.70889	0.70889	0.00009
4.93	0.70922	0.70891	0.00009
4.89	0.70880	0.70891	0.00009
4.85	0.70886	0.70892	0.00010
4.80	0.70909	0.70890	0.00009
4.76	0.70911	0.70895	0.00014
4.72	0.70897	0.70894	0.00014
4.68	0.70900	0.70898	0.00014
4.63	0.70872	0.70898	0.00014
4.59	0.70886	0.70900	0.00012
4.55	0.70872	0.70904	0.00012
4.50	0.70881	0.70906	0.00012
4.46	0.70897	0.70905	0.00012
4.42	0.70890	0.70901	0.00013
4.38	0.70917	0.70904	0.00013
4.33	0.70892	0.70901	0.00010
4.29	0.70943	0.70904	0.00011
4.25	0.70887	0.70903	0.00010
4.20	0.70919	0.70905	0.00010
4.16	0.70885	0.70904	0.00010
4.12	0.70893	0.70902	0.00010
4.08	0.70918	0.70902	0.00009
4.03	0.70912	0.70907	0.00011
3.99	0.70887	0.70909	0.00009
3.95	0.70880	0.70906	0.00009
3.90	0.70922	0.70905	0.00009

3.86	0.70904	0.70899	0.00011
3.82	0.70925	0.70899	0.00011
3.78	0.70901	0.70901	0.00012
3.73	0.70905	0.70897	0.00016
3.69	0.70886	0.70894	0.00016
3.65	0.70904	0.70893	0.00016
3.60	0.70910	0.70888	0.00013
3.56	0.70935	0.70888	0.00013
3.52	0.70896	0.70887	0.00014
3.47	0.70891	0.70890	0.00016
3.43	0.70901	0.70890	0.00015
3.39	0.70864	0.70888	0.00015
3.35	0.70903	0.70884	0.00013
3.30	0.70918	0.70895	0.00015
3.26	0.70847	0.70898	0.00015
3.22	0.70879	0.70899	0.00015
3.17	0.70897	0.70899	0.00015
3.13	0.70880	0.70898	0.00014
3.09	0.70896	0.70897	0.00014
3.05	0.70896	0.70898	0.00013
3.00	0.70923	0.70898	0.00013
2.96	0.70869	0.70897	0.00013
2.92	0.70881	0.70894	0.00009
2.87	0.70887	0.70890	0.00009
2.83	0.70942	0.70888	0.00008
2.79	0.70911	0.70889	0.00008
2.75	0.70904	0.70887	0.00009
2.70	0.70876	0.70885	0.00009
2.66	0.70892	0.70881	0.00006
2.62	0.70896	0.70883	0.00007
2.57	0.70912	0.70884	0.00007
2.53	0.70881	0.70885	0.00007
2.49	0.70881	0.70886	0.00008
2.45	0.70876	0.70888	0.00008
2.40	0.70906	0.70890	0.00008
2.36	0.70876	0.70890	0.00008
2.32	0.70882	0.70894	0.00008
2.27	0.70887	0.70895	0.00008
2.23	0.70871	0.70898	0.00008
2.19	0.70875	0.70898	0.00008
2.14	0.70879	0.70899	0.00008
2.10	0.70896	0.70900	0.00007
2.06	0.70897	0.70900	0.00007
2.02	0.70885	0.70901	0.00007
1.97	0.70911	0.70902	0.00008
1.93	0.70902	0.70903	0.00007
1.89	0.70902	0.70902	0.00006
1.84	0.70884	0.70904	0.00004
1.80	0.70914	0.70905	0.00005
1.76	0.70878	0.70907	0.00005

1.72	0.70911	0.70906	0.00006
1.67	0.70898	0.70908	0.00007
1.63	0.70905	0.70908	0.00007
1.59	0.70900	0.70905	0.00010
1.54	0.70909	0.70906	0.00010
1.50	0.70905	0.70910	0.00012
1.46	0.70913	0.70905	0.00016
1.42	0.70895	0.70908	0.00017
1.37	0.70903	0.70906	0.00017
1.33	0.70902	0.70901	0.00018
1.29	0.70920	0.70901	0.00018
1.24	0.70920	0.70899	0.00017
1.20	0.70892	0.70898	0.00017
1.16	0.70922	0.70901	0.00016
1.12	0.70913	0.70898	0.00015
1.07	0.70872	0.70895	0.00013
1.03	0.70920	0.70900	0.00010
0.99	0.70937	0.70896	0.00007
0.94	0.70856	0.70900	0.00010
0.90	0.70932	0.70902	0.00007
0.86	0.70897	0.70902	0.00008
0.82	0.70870	0.70898	0.00010
0.77	0.70892	0.70900	0.00011
0.73	0.70905	0.70899	0.00011
0.69	0.70902	0.70901	0.00012
0.64	0.70898	0.70904	0.00013
0.60	0.70894	0.70904	0.00013
0.56	0.70899	0.70904	0.00013
0.51	0.70908	0.70902	0.00011
0.47	0.70894	0.70900	0.00012
0.43	0.70932	0.70901	0.00012
0.39	0.70897	0.70904	0.00011
0.34	0.70888	0.70902	0.00010
0.30	0.70870	0.70904	0.00010
0.26	0.70923	0.70903	0.00011
0.21	0.70887	0.70898	0.00009
0.17	0.70914	0.70895	0.00010
0.13	0.70925	0.70896	0.00012
0.09	0.70909	0.70887	0.00009
0.04	0.70892		

ARB 14.2.1 (M2)

Distance from cervix (mm)	$^{87}Sr/^{86}Sr$	10 point mov. average	2 SE on mov. average
44.95	0.70935	0.70900	0.00021
44.91	0.70899	0.70893	0.00020
44.86	0.70882	0.70894	0.00020
44.82	0.70874	0.70898	0.00021
44.78	0.70911	0.70899	0.00021
44.73	0.70875	0.70896	0.00021
44.69	0.70961	0.70904	0.00024
44.65	0.70880	0.70895	0.00021
44.60	0.70855	0.70900	0.00022
44.56	0.70926	0.70905	0.00020
44.52	0.70868	0.70902	0.00019
44.47	0.70905	0.70904	0.00018
44.43	0.70929	0.70900	0.00020
44.39	0.70881	0.70896	0.00019
44.35	0.70876	0.70896	0.00018
44.30	0.70959	0.70896	0.00019
44.26	0.70870	0.70895	0.00018
44.22	0.70935	0.70905	0.00022
44.17	0.70899	0.70905	0.00022
44.13	0.70895	0.70902	0.00024
44.09	0.70889	0.70901	0.00024
44.04	0.70867	0.70905	0.00024
44.00	0.70888	0.70910	0.00023
43.96	0.70886	0.70907	0.00024
43.91	0.70869	0.70910	0.00024
43.87	0.70952	0.70912	0.00022
43.83	0.70966	0.70902	0.00024
43.78	0.70942	0.70897	0.00019
43.74	0.70866	0.70896	0.00019
43.70	0.70883	0.70901	0.00018
43.65	0.70926	0.70901	0.00017
43.61	0.70919	0.70899	0.00017
43.57	0.70865	0.70894	0.00017
43.52	0.70908	0.70899	0.00016
43.48	0.70895	0.70896	0.00016
43.44	0.70849	0.70895	0.00016
43.39	0.70914	0.70897	0.00014
43.35	0.70936	0.70892	0.00015
43.31	0.70915	0.70894	0.00017
43.26	0.70885	0.70894	0.00017
43.22	0.70900	0.70887	0.00023
43.18	0.70875	0.70890	0.00025
43.13	0.70913	0.70894	0.00025
43.09	0.70872	0.70893	0.00025
43.05	0.70889	0.70895	0.00024
43.01	0.70868	0.70897	0.00024
42.96	0.70865	0.70898	0.00024

ARB 14.3.1 (M3)

Distance from cervix (mm)	$^{87}Sr/^{86}Sr$	10 point mov. average	2 SE on mov. average
51.86	0.70985	0.71009	0.00019
51.82	0.71045	0.71010	0.00018
51.77	0.71035	0.71003	0.00018
51.73	0.70938	0.71001	0.00016
51.69	0.71011	0.71000	0.00017
51.64	0.71017	0.70994	0.00020
51.60	0.71020	0.70997	0.00023
51.56	0.71014	0.70991	0.00023
51.51	0.71019	0.70984	0.00025
51.47	0.71010	0.70976	0.00024
51.43	0.70993	0.70974	0.00023
51.38	0.70978	0.70965	0.00027
51.34	0.71006	0.70959	0.00028
51.30	0.70934	0.70954	0.00026
51.25	0.70946	0.70958	0.00025
51.21	0.71052	0.70954	0.00027
51.17	0.70960	0.70948	0.00019
51.12	0.70940	0.70940	0.00023
51.08	0.70945	0.70935	0.00025
51.04	0.70985	0.70933	0.00025
50.99	0.70905	0.70933	0.00025
50.95	0.70922	0.70941	0.00026
50.91	0.70957	0.70939	0.00028
50.87	0.70971	0.70929	0.00032
50.82	0.70908	0.70927	0.00031
50.78	0.70989	0.70927	0.00031
50.74	0.70878	0.70918	0.00028
50.69	0.70889	0.70915	0.00031
50.65	0.70927	0.70921	0.00031
50.61	0.70982	0.70925	0.00033
50.56	0.70989	0.70926	0.00033
50.52	0.70899	0.70917	0.00030
50.48	0.70857	0.70919	0.00030
50.43	0.70953	0.70926	0.00027
50.39	0.70906	0.70930	0.00029
50.35	0.70903	0.70930	0.00029
50.30	0.70842	0.70934	0.00029
50.26	0.70949	0.70942	0.00020
50.22	0.70974	0.70935	0.00024
50.17	0.70984	0.70929	0.00022
50.13	0.70901	0.70928	0.00021
50.09	0.70917	0.70929	0.00020
50.04	0.70934	0.70929	0.00020
50.00	0.70989	0.70924	0.00022
49.96	0.70902	0.70913	0.00018
49.91	0.70943	0.70910	0.00019
49.87	0.70929	0.70908	0.00018

42.92	0.70952	0.70904	0.00023	49.83	0.70878	0.70905	0.00018
42.88	0.70918	0.70899	0.00020	49.78	0.70917	0.70912	0.00019
42.83	0.70818	0.70899	0.00020	49.74	0.70966	0.70913	0.00019
42.79	0.70931	0.70905	0.00011	49.70	0.70916	0.70912	0.00017
42.75	0.70913	0.70895	0.00017	49.65	0.70913	0.70915	0.00019
42.70	0.70905	0.70889	0.00018	49.61	0.70882	0.70915	0.00019
42.66	0.70894	0.70893	0.00020	49.57	0.70883	0.70922	0.00019
42.62	0.70905	0.70901	0.00026	49.52	0.70875	0.70924	0.00018
42.57	0.70884	0.70902	0.00026	49.48	0.70921	0.70934	0.00017
42.53	0.70925	0.70902	0.00026	49.44	0.70897	0.70939	0.00017
42.49	0.70893	0.70901	0.00026	49.39	0.70951	0.70943	0.00015
42.44	0.70920	0.70901	0.00026	49.35	0.70926	0.70946	0.00017
42.40	0.70882	0.70901	0.00026	49.31	0.70952	0.70951	0.00018
42.36	0.70832	0.70907	0.00026	49.26	0.70954	0.70946	0.00020
42.31	0.70855	0.70917	0.00020	49.22	0.70910	0.70944	0.00021
42.27	0.70936	0.70921	0.00016	49.18	0.70953	0.70949	0.00019
42.23	0.70976	0.70917	0.00016	49.14	0.70905	0.70946	0.00020
42.18	0.70914	0.70915	0.00013	49.09	0.70976	0.70956	0.00020
42.14	0.70892	0.70919	0.00016	49.05	0.70968	0.70952	0.00020
42.10	0.70905	0.70923	0.00015	49.01	0.70931	0.70954	0.00021
42.05	0.70902	0.70923	0.00015	48.96	0.70987	0.70955	0.00020
42.01	0.70920	0.70920	0.00018	48.92	0.70978	0.70945	0.00024
41.97	0.70937	0.70925	0.00020	48.88	0.70903	0.70937	0.00024
41.92	0.70929	0.70919	0.00021	48.83	0.70926	0.70940	0.00022
41.88	0.70902	0.70911	0.00025	48.79	0.70962	0.70945	0.00023
41.84	0.70889	0.70917	0.00027	48.75	0.70926	0.70943	0.00023
41.79	0.70957	0.70915	0.00028	48.70	0.71000	0.70942	0.00023
41.75	0.70959	0.70911	0.00026	48.66	0.70943	0.70939	0.00020
41.71	0.70931	0.70905	0.00024	48.62	0.70987	0.70941	0.00021
41.67	0.70906	0.70906	0.00024	48.57	0.70941	0.70941	0.00021
41.62	0.70872	0.70908	0.00025	48.53	0.70884	0.70940	0.00021
41.58	0.70964	0.70905	0.00027	48.49	0.70904	0.70947	0.00017
41.54	0.70883	0.70905	0.00027	48.44	0.70927	0.70951	0.00014
41.49	0.70849	0.70905	0.00027	48.40	0.70974	0.70950	0.00015
41.45	0.70957	0.70911	0.00024	48.36	0.70942	0.70952	0.00017
41.41	0.70874	0.70905	0.00022	48.31	0.70921	0.70959	0.00020
41.36	0.70915	0.70910	0.00021	48.27	0.70971	0.70967	0.00020
41.32	0.70901	0.70909	0.00021	48.23	0.70963	0.70965	0.00020
41.28	0.70937	0.70909	0.00021	48.18	0.70986	0.70963	0.00020
41.23	0.70928	0.70905	0.00020	48.14	0.70927	0.70959	0.00020
41.19	0.70844	0.70904	0.00020	48.10	0.70955	0.70960	0.00019
41.15	0.70965	0.70916	0.00018	48.05	0.70945	0.70960	0.00019
41.10	0.70877	0.70913	0.00015	48.01	0.70917	0.70955	0.00024
41.06	0.70909	0.70916	0.00013	47.97	0.70993	0.70961	0.00023
41.02	0.70897	0.70918	0.00013	47.92	0.71009	0.70959	0.00022
40.97	0.70923	0.70917	0.00014	47.88	0.71003	0.70965	0.00030
40.93	0.70909	0.70916	0.00014	47.84	0.70952	0.70963	0.00029
40.89	0.70904	0.70914	0.00016	47.79	0.70941	0.70966	0.00029
40.84	0.70893	0.70914	0.00016	47.75	0.70946	0.70971	0.00029
40.80	0.70917	0.70914	0.00016	47.71	0.70942	0.70975	0.00029

40.76	0.70961	0.70914	0.00016
40.71	0.70942	0.70905	0.00014
40.67	0.70904	0.70905	0.00014
40.63	0.70928	0.70902	0.00015
40.58	0.70885	0.70897	0.00015
40.54	0.70919	0.70901	0.00015
40.50	0.70883	0.70900	0.00015
40.46	0.70912	0.70895	0.00020
40.41	0.70887	0.70892	0.00020
40.37	0.70916	0.70894	0.00020
40.33	0.70872	0.70891	0.00019
40.28	0.70941	0.70894	0.00019
40.24	0.70875	0.70892	0.00017
40.20	0.70882	0.70893	0.00016
40.15	0.70921	0.70894	0.00016
40.11	0.70915	0.70897	0.00020
40.07	0.70832	0.70898	0.00020
40.02	0.70882	0.70907	0.00014
39.98	0.70901	0.70905	0.00015
39.94	0.70887	0.70906	0.00015
39.89	0.70901	0.70908	0.00014
39.85	0.70919	0.70910	0.00015
39.81	0.70886	0.70909	0.00015
39.76	0.70896	0.70914	0.00014
39.72	0.70955	0.70918	0.00014
39.68	0.70922	0.70909	0.00015
39.63	0.70916	0.70913	0.00018
39.59	0.70872	0.70905	0.00024
39.55	0.70910	0.70911	0.00023
39.50	0.70900	0.70910	0.00023
39.46	0.70924	0.70915	0.00025
39.42	0.70912	0.70918	0.00026
39.37	0.70929	0.70918	0.00026
39.33	0.70939	0.70915	0.00026
39.29	0.70869	0.70911	0.00026
39.24	0.70959	0.70915	0.00024
39.20	0.70834	0.70910	0.00022
39.16	0.70937	0.70921	0.00014
39.12	0.70894	0.70918	0.00014
39.07	0.70954	0.70920	0.00013
39.03	0.70952	0.70911	0.00015
38.99	0.70912	0.70908	0.00012
38.94	0.70899	0.70907	0.00012
38.90	0.70899	0.70903	0.00015
38.86	0.70913	0.70905	0.00016
38.81	0.70910	0.70904	0.00016
38.77	0.70936	0.70902	0.00016
38.73	0.70911	0.70900	0.00015
38.68	0.70916	0.70901	0.00015
38.64	0.70863	0.70906	0.00020
47.66	0.70954	0.70976	0.00028
47.62	0.70888	0.70980	0.00028
47.58	0.70985	0.70990	0.00020
47.53	0.70971	0.70986	0.00022
47.49	0.71067	0.70986	0.00022
47.45	0.70978	0.70974	0.00014
47.40	0.70987	0.70974	0.00014
47.36	0.70990	0.70965	0.00019
47.32	0.70991	0.70961	0.00019
47.28	0.70945	0.70956	0.00018
47.23	0.70993	0.70955	0.00018
47.19	0.70993	0.70953	0.00017
47.15	0.70941	0.70957	0.00022
47.10	0.70978	0.70961	0.00022
47.06	0.70946	0.70962	0.00023
47.02	0.70973	0.70965	0.00022
46.97	0.70904	0.70973	0.00029
46.93	0.70951	0.70980	0.00025
46.89	0.70937	0.70981	0.00024
46.84	0.70932	0.70990	0.00024
46.80	0.70975	0.71002	0.00022
46.76	0.71030	0.71008	0.00023
46.71	0.70983	0.71009	0.00023
46.67	0.70989	0.71013	0.00022
46.63	0.70974	0.71011	0.00024
46.58	0.71057	0.71015	0.00022
46.54	0.70974	0.71011	0.00020
46.50	0.70957	0.71020	0.00020
46.45	0.71034	0.71020	0.00019
46.41	0.71043	0.71014	0.00021
46.37	0.71036	0.71006	0.00022
46.32	0.71040	0.70997	0.00024
46.28	0.71025	0.70993	0.00023
46.24	0.70972	0.70988	0.00022
46.19	0.71017	0.70994	0.00024
46.15	0.71017	0.70989	0.00024
46.11	0.71055	0.70990	0.00024
46.06	0.70961	0.70984	0.00020
46.02	0.70974	0.70986	0.00019
45.98	0.70964	0.70980	0.00025
45.93	0.70942	0.70983	0.00025
45.89	0.71007	0.70986	0.00023
45.85	0.70968	0.70984	0.00023
45.80	0.71040	0.70981	0.00024
45.76	0.70966	0.70975	0.00021
45.72	0.71020	0.70969	0.00025
45.67	0.71001	0.70973	0.00030
45.63	0.70983	0.70968	0.00030
45.59	0.70908	0.70971	0.00032
45.55	0.70998	0.70976	0.00029

38.60	0.70919	0.70909	0.00018	45.50	0.70969	0.70975	0.00029
38.55	0.70903	0.70909	0.00018	45.46	0.70988	0.70979	0.00030
38.51	0.70860	0.70907	0.00019	45.42	0.70939	0.70980	0.00030
38.47	0.70925	0.70915	0.00016	45.37	0.70981	0.70989	0.00029
38.42	0.70895	0.70918	0.00018	45.33	0.70902	0.70990	0.00029
38.38	0.70890	0.70917	0.00019	45.29	0.71065	0.70996	0.00023
38.34	0.70920	0.70918	0.00018	45.24	0.70944	0.70987	0.00017
38.29	0.70917	0.70915	0.00019	45.20	0.71020	0.70993	0.00015
38.25	0.70969	0.70919	0.00021	45.16	0.70954	0.70982	0.00020
38.21	0.70893	0.70911	0.00018	45.11	0.70990	0.70983	0.00019
38.16	0.70918	0.70913	0.00018	45.07	0.71009	0.70983	0.00020
38.12	0.70883	0.70912	0.00018	45.03	0.70998	0.70979	0.00019
38.08	0.70938	0.70916	0.00017	44.98	0.71023	0.70975	0.00019
38.03	0.70955	0.70914	0.00016	44.94	0.70998	0.70971	0.00016
37.99	0.70889	0.70908	0.00014	44.90	0.70963	0.70973	0.00017
37.95	0.70897	0.70911	0.00013	44.85	0.70975	0.70978	0.00019
37.91	0.70892	0.70914	0.00013	44.81	0.70996	0.70982	0.00020
37.86	0.70959	0.70922	0.00016	44.77	0.70913	0.70984	0.00021
37.82	0.70888	0.70918	0.00014	44.72	0.70966	0.70996	0.00016
37.78	0.70912	0.70921	0.00012	44.68	0.70993	0.70994	0.00018
37.73	0.70909	0.70925	0.00013	44.64	0.70963	0.70997	0.00019
37.69	0.70919	0.70922	0.00016	44.59	0.70964	0.71003	0.00018
37.65	0.70922	0.70918	0.00018	44.55	0.70982	0.71006	0.00016
37.60	0.70892	0.70919	0.00018	44.51	0.71017	0.71005	0.00017
37.56	0.70922	0.70920	0.00018	44.46	0.71016	0.71004	0.00017
37.52	0.70926	0.70918	0.00018	44.42	0.71006	0.71003	0.00017
37.47	0.70968	0.70908	0.00026	44.38	0.71017	0.71006	0.00018
37.43	0.70922	0.70898	0.00023	44.33	0.71035	0.71000	0.00021
37.39	0.70921	0.70899	0.00024	44.29	0.70950	0.70998	0.00019
37.34	0.70952	0.70898	0.00023	44.25	0.71020	0.71000	0.00018
37.30	0.70880	0.70900	0.00026	44.20	0.71026	0.70997	0.00017
37.26	0.70880	0.70898	0.00027	44.16	0.70996	0.70996	0.00016
37.21	0.70931	0.70903	0.00027	44.12	0.70967	0.70991	0.00018
37.17	0.70903	0.70903	0.00027	44.07	0.71006	0.70995	0.00018
37.13	0.70897	0.70905	0.00027	44.03	0.71010	0.70993	0.00017
37.08	0.70822	0.70903	0.00028	43.99	0.71035	0.70992	0.00017
37.04	0.70875	0.70913	0.00022	43.94	0.70953	0.70987	0.00014
37.00	0.70934	0.70918	0.00020	43.90	0.71017	0.70993	0.00013
36.95	0.70905	0.70912	0.00022	43.86	0.70967	0.70986	0.00014
36.91	0.70974	0.70916	0.00023	43.81	0.70997	0.70985	0.00015
36.87	0.70862	0.70905	0.00021	43.77	0.71008	0.70984	0.00015
36.82	0.70930	0.70909	0.00019	43.73	0.70954	0.70981	0.00014
36.78	0.70929	0.70909	0.00019	43.69	0.70999	0.70983	0.00013
36.74	0.70923	0.70905	0.00019	43.64	0.70987	0.70988	0.00018
36.69	0.70872	0.70899	0.00020	43.60	0.71000	0.70989	0.00018
36.65	0.70927	0.70908	0.00022	43.56	0.70984	0.70991	0.00019
36.61	0.70929	0.70908	0.00022	43.51	0.71017	0.70992	0.00019
36.57	0.70871	0.70907	0.00022	43.47	0.70951	0.70991	0.00019
36.52	0.70945	0.70909	0.00021	43.43	0.70956	0.70998	0.00017
36.48	0.70861	0.70904	0.00020	43.38	0.70980	0.71002	0.00015

36.44	0.70903	0.70910	0.00018	43.34	0.70982	0.71001	0.00015
36.39	0.70925	0.70912	0.00018	43.30	0.70975	0.70999	0.00017
36.35	0.70891	0.70909	0.00018	43.25	0.71048	0.71001	0.00016
36.31	0.70866	0.70909	0.00018	43.21	0.70991	0.70989	0.00019
36.26	0.70965	0.70910	0.00016	43.17	0.71024	0.70986	0.00019
36.22	0.70924	0.70907	0.00013	43.12	0.70995	0.70984	0.00018
36.18	0.70924	0.70905	0.00012	43.08	0.71002	0.70984	0.00018
36.13	0.70884	0.70909	0.00017	43.04	0.71021	0.70986	0.00019
36.09	0.70897	0.70910	0.00017	42.99	0.70998	0.70982	0.00017
36.05	0.70920	0.70912	0.00017	42.95	0.70973	0.70983	0.00018
36.00	0.70924	0.70905	0.00020	42.91	0.70961	0.70981	0.00019
35.96	0.70891	0.70907	0.00022	42.86	0.71000	0.70985	0.00019
35.92	0.70892	0.70911	0.00021	42.82	0.70924	0.70980	0.00020
35.87	0.70880	0.70913	0.00021	42.78	0.70965	0.70987	0.00016
35.83	0.70938	0.70914	0.00020	42.73	0.71001	0.70992	0.00016
35.79	0.70900	0.70908	0.00021	42.69	0.70990	0.70993	0.00016
35.74	0.70964	0.70907	0.00021	42.65	0.71023	0.70996	0.00017
35.70	0.70888	0.70896	0.00019	42.60	0.70983	0.70989	0.00018
35.66	0.70924	0.70900	0.00020	42.56	0.71014	0.70994	0.00020
35.61	0.70852	0.70896	0.00020	42.52	0.70952	0.70990	0.00020
35.57	0.70944	0.70899	0.00018	42.47	0.70995	0.70992	0.00018
35.53	0.70925	0.70895	0.00014	42.43	0.70949	0.70995	0.00019
35.48	0.70910	0.70892	0.00013	42.39	0.70999	0.70998	0.00018
35.44	0.70891	0.70899	0.00021	42.34	0.71014	0.71000	0.00018
35.40	0.70878	0.70895	0.00023	42.30	0.71015	0.70994	0.00020
35.36	0.70890	0.70899	0.00023	42.26	0.71019	0.70990	0.00020
35.31	0.70857	0.70904	0.00024	42.21	0.70952	0.70987	0.00019
35.27	0.70933	0.70904	0.00023	42.17	0.71033	0.70990	0.00017
35.23	0.70880	0.70901	0.00023	42.13	0.70975	0.70987	0.00015
35.18	0.70885	0.70901	0.00022	42.08	0.70974	0.70985	0.00016
35.14	0.70901	0.70907	0.00024	42.04	0.71025	0.70983	0.00018
35.10	0.70896	0.70913	0.00025	42.00	0.70971	0.70982	0.00017
35.05	0.70977	0.70911	0.00026	41.96	0.71022	0.70984	0.00017
35.01	0.70858	0.70900	0.00022	41.91	0.70955	0.70982	0.00015
34.97	0.70915	0.70901	0.00022	41.87	0.70974	0.70992	0.00021
34.92	0.70936	0.70901	0.00021	41.83	0.70995	0.70995	0.00020
34.88	0.70861	0.70891	0.00023	41.78	0.70981	0.70996	0.00020
34.84	0.70903	0.70893	0.00022	41.74	0.71004	0.70992	0.00024
34.79	0.70882	0.70890	0.00022	41.70	0.70950	0.70994	0.00025
34.75	0.70945	0.70893	0.00023	41.65	0.70951	0.71002	0.00023
34.71	0.70953	0.70889	0.00020	41.61	0.71017	0.71002	0.00023
34.66	0.70877	0.70885	0.00015	41.57	0.70990	0.71000	0.00023
34.62	0.70872	0.70883	0.00017	41.52	0.71000	0.70992	0.00029
34.58	0.70870	0.70887	0.00018	41.48	0.71061	0.70995	0.00030
34.53	0.70908	0.70888	0.00018	41.44	0.71001	0.70989	0.00026
34.49	0.70838	0.70885	0.00017	41.39	0.71008	0.70988	0.00026
34.45	0.70886	0.70891	0.00014	41.35	0.70937	0.70978	0.00030
34.40	0.70872	0.70894	0.00015	41.31	0.71029	0.70981	0.00028
34.36	0.70910	0.70896	0.00014	41.26	0.71024	0.70978	0.00027
34.32	0.70901	0.70899	0.00017	41.22	0.70955	0.70965	0.00030

34.27	0.70918	0.70907	0.00024	41.18	0.70992	0.70965	0.00030
34.23	0.70851	0.70906	0.00023	41.13	0.70912	0.70963	0.00029
34.19	0.70917	0.70912	0.00020	41.09	0.71029	0.70970	0.00027
34.14	0.70884	0.70908	0.00021	41.05	0.70999	0.70964	0.00023
34.10	0.70875	0.70912	0.00021	41.00	0.70999	0.70961	0.00022
34.06	0.70891	0.70912	0.00021	40.96	0.70909	0.70957	0.00020
34.02	0.70917	0.70915	0.00021	40.92	0.70966	0.70961	0.00017
33.97	0.70891	0.70911	0.00022	40.87	0.70995	0.70960	0.00018
33.93	0.70947	0.70914	0.00022	40.83	0.70891	0.70957	0.00016
33.89	0.70981	0.70910	0.00020	40.79	0.70958	0.70967	0.00008
33.84	0.70906	0.70904	0.00013	40.74	0.70973	0.70964	0.00010
33.80	0.70906	0.70903	0.00013	40.70	0.70980	0.70963	0.00010
33.76	0.70877	0.70897	0.00017	40.66	0.70970	0.70961	0.00009
33.71	0.70927	0.70898	0.00017	40.61	0.70969	0.70954	0.00016
33.67	0.70873	0.70891	0.00017	40.57	0.70959	0.70956	0.00017
33.63	0.70926	0.70888	0.00020	40.53	0.70955	0.70954	0.00017
33.58	0.70878	0.70888	0.00020	40.48	0.70949	0.70953	0.00017
33.54	0.70923	0.70890	0.00020	40.44	0.70967	0.70951	0.00018
33.50	0.70902	0.70886	0.00019	40.40	0.70988	0.70948	0.00018
33.45	0.70919	0.70884	0.00019	40.35	0.70931	0.70942	0.00016
33.41	0.70899	0.70884	0.00018	40.31	0.70964	0.70942	0.00015
33.37	0.70848	0.70883	0.00018	40.27	0.70961	0.70937	0.00015
33.32	0.70884	0.70883	0.00018	40.23	0.70897	0.70937	0.00015
33.28	0.70862	0.70882	0.00018	40.18	0.70987	0.70945	0.00014
33.24	0.70836	0.70885	0.00018	40.14	0.70943	0.70939	0.00011
33.19	0.70930	0.70890	0.00014	40.10	0.70940	0.70935	0.00013
33.15	0.70899	0.70889	0.00012	40.05	0.70934	0.70936	0.00013
33.11	0.70880	0.70891	0.00013	40.01	0.70936	0.70932	0.00014
33.06	0.70886	0.70897	0.00016	39.97	0.70925	0.70929	0.00016
33.02	0.70915	0.70898	0.00016	39.92	0.70935	0.70927	0.00016
32.98	0.70887	0.70895	0.00016	39.88	0.70917	0.70923	0.00017
32.93	0.70848	0.70890	0.00019	39.84	0.70957	0.70924	0.00017
32.89	0.70881	0.70891	0.00018	39.79	0.70977	0.70921	0.00016
32.85	0.70891	0.70893	0.00017	39.75	0.70923	0.70920	0.00014
32.80	0.70887	0.70896	0.00019	39.71	0.70908	0.70915	0.00016
32.76	0.70915	0.70901	0.00020	39.66	0.70945	0.70922	0.00019
32.72	0.70920	0.70901	0.00020	39.62	0.70902	0.70922	0.00020
32.68	0.70939	0.70900	0.00020	39.58	0.70901	0.70928	0.00020
32.63	0.70897	0.70891	0.00021	39.53	0.70906	0.70930	0.00020
32.59	0.70882	0.70885	0.00023	39.49	0.70898	0.70930	0.00020
32.55	0.70847	0.70886	0.00023	39.45	0.70928	0.70932	0.00018
32.50	0.70858	0.70890	0.00021	39.40	0.70925	0.70935	0.00019
32.46	0.70892	0.70888	0.00023	39.36	0.70965	0.70937	0.00019
32.42	0.70928	0.70891	0.00024	39.32	0.70878	0.70932	0.00018
32.37	0.70938	0.70895	0.00028	39.27	0.70971	0.70940	0.00014
32.33	0.70915	0.70890	0.00027	39.23	0.70951	0.70938	0.00013
32.29	0.70907	0.70891	0.00027	39.19	0.70960	0.70941	0.00015
32.24	0.70845	0.70898	0.00031	39.14	0.70919	0.70939	0.00015
32.20	0.70839	0.70906	0.00030	39.10	0.70906	0.70940	0.00015
32.16	0.70891	0.70910	0.00026	39.06	0.70923	0.70944	0.00012

32.11	0.70889	0.70908	0.00028
32.07	0.70834	0.70914	0.00029
32.03	0.70924	0.70916	0.00027
31.98	0.70970	0.70907	0.00031
31.94	0.70889	0.70902	0.00028
31.90	0.70927	0.70907	0.00029
31.85	0.70969	0.70903	0.00029
31.81	0.70929	0.70895	0.00025
31.77	0.70882	0.70883	0.00028
31.72	0.70865	0.70887	0.00029
31.68	0.70954	0.70891	0.00029
31.64	0.70847	0.70890	0.00028
31.59	0.70843	0.70896	0.00026
31.55	0.70914	0.70904	0.00024
31.51	0.70945	0.70904	0.00024
31.47	0.70879	0.70904	0.00024
31.42	0.70889	0.70906	0.00023
31.38	0.70817	0.70909	0.00023
31.34	0.70917	0.70915	0.00013
31.29	0.70904	0.70906	0.00022
31.25	0.70947	0.70904	0.00022
31.21	0.70908	0.70902	0.00021
31.16	0.70925	0.70903	0.00021
31.12	0.70914	0.70902	0.00021
31.08	0.70941	0.70903	0.00021
31.03	0.70897	0.70902	0.00021
30.99	0.70917	0.70898	0.00022
30.95	0.70879	0.70892	0.00023
30.90	0.70826	0.70893	0.00023
30.86	0.70883	0.70899	0.00018
30.82	0.70930	0.70900	0.00018
30.77	0.70917	0.70896	0.00016
30.73	0.70912	0.70890	0.00017
30.69	0.70923	0.70891	0.00018
30.64	0.70935	0.70882	0.00020
30.60	0.70861	0.70880	0.00018
30.56	0.70856	0.70883	0.00017
30.51	0.70888	0.70891	0.00019
30.47	0.70883	0.70894	0.00020
30.43	0.70896	0.70894	0.00020
30.38	0.70889	0.70900	0.00024
30.34	0.70856	0.70901	0.00023
30.30	0.70922	0.70903	0.00022
30.25	0.70834	0.70908	0.00026
30.21	0.70915	0.70915	0.00020
30.17	0.70888	0.70911	0.00022
30.13	0.70935	0.70914	0.00021
30.08	0.70919	0.70911	0.00021
30.04	0.70881	0.70906	0.00022
30.00	0.70957	0.70907	0.00022

39.01	0.70952	0.70947	0.00012
38.97	0.70942	0.70952	0.00015
38.93	0.70917	0.70946	0.00020
38.88	0.70958	0.70948	0.00019
38.84	0.70955	0.70949	0.00019
38.80	0.70983	0.70947	0.00019
38.75	0.70931	0.70941	0.00018
38.71	0.70932	0.70939	0.00019
38.67	0.70944	0.70938	0.00019
38.62	0.70960	0.70942	0.00021
38.58	0.70994	0.70943	0.00021
38.54	0.70886	0.70941	0.00020
38.49	0.70935	0.70944	0.00016
38.45	0.70966	0.70944	0.00016
38.41	0.70937	0.70940	0.00016
38.37	0.70920	0.70942	0.00016
38.32	0.70913	0.70941	0.00017
38.28	0.70925	0.70938	0.00019
38.24	0.70979	0.70938	0.00019
38.19	0.70970	0.70930	0.00018
38.15	0.70974	0.70923	0.00017
38.11	0.70924	0.70915	0.00013
38.06	0.70926	0.70914	0.00013
38.02	0.70931	0.70915	0.00013
37.98	0.70954	0.70908	0.00016
37.93	0.70910	0.70907	0.00015
37.89	0.70891	0.70908	0.00016
37.85	0.70927	0.70911	0.00015
37.80	0.70899	0.70906	0.00016
37.76	0.70895	0.70913	0.00020
37.72	0.70893	0.70915	0.00019
37.67	0.70916	0.70919	0.00019
37.63	0.70932	0.70919	0.00019
37.59	0.70861	0.70918	0.00019
37.54	0.70944	0.70920	0.00016
37.50	0.70924	0.70922	0.00017
37.46	0.70917	0.70921	0.00017
37.41	0.70883	0.70928	0.00021
37.37	0.70968	0.70936	0.00019
37.33	0.70915	0.70934	0.00018
37.28	0.70930	0.70932	0.00019
37.24	0.70915	0.70931	0.00019
37.20	0.70920	0.70931	0.00019
37.15	0.70885	0.70929	0.00020
37.11	0.70958	0.70936	0.00018
37.07	0.70919	0.70934	0.00018
37.02	0.70981	0.70937	0.00018
36.98	0.70963	0.70928	0.00017
36.94	0.70948	0.70919	0.00018
36.89	0.70904	0.70915	0.00017

29.95	0.70904	0.70893	0.00025	36.85	0.70912	0.70917	0.00017
29.91	0.70879	0.70894	0.00026	36.81	0.70917	0.70917	0.00017
29.87	0.70973	0.70901	0.00027	36.76	0.70898	0.70920	0.00017
29.82	0.70902	0.70900	0.00027	36.72	0.70959	0.70924	0.00017
29.78	0.70872	0.70896	0.00028	36.68	0.70936	0.70916	0.00017
29.74	0.70921	0.70900	0.00027	36.64	0.70950	0.70915	0.00016
29.69	0.70898	0.70898	0.00027	36.59	0.70891	0.70912	0.00014
29.65	0.70873	0.70901	0.00028	36.55	0.70874	0.70917	0.00014
29.61	0.70888	0.70906	0.00028	36.51	0.70909	0.70925	0.00013
29.56	0.70816	0.70913	0.00029	36.46	0.70926	0.70929	0.00013
29.52	0.70922	0.70922	0.00020	36.42	0.70914	0.70925	0.00015
29.48	0.70945	0.70921	0.00020	36.38	0.70939	0.70920	0.00019
29.43	0.70966	0.70918	0.00019	36.33	0.70938	0.70917	0.00019
29.39	0.70861	0.70909	0.00017	36.29	0.70883	0.70914	0.00018
29.35	0.70911	0.70913	0.00014	36.25	0.70927	0.70918	0.00017
29.30	0.70894	0.70910	0.00015	36.20	0.70922	0.70916	0.00017
29.26	0.70932	0.70913	0.00014	36.16	0.70935	0.70914	0.00017
29.22	0.70924	0.70908	0.00014	36.12	0.70956	0.70912	0.00017
29.17	0.70960	0.70903	0.00016	36.07	0.70947	0.70906	0.00014
29.13	0.70908	0.70899	0.00010	36.03	0.70891	0.70908	0.00017
29.09	0.70912	0.70893	0.00014	35.99	0.70864	0.70908	0.00017
29.04	0.70908	0.70887	0.00015	35.94	0.70909	0.70914	0.00014
29.00	0.70883	0.70888	0.00015	35.90	0.70911	0.70914	0.00014
28.96	0.70898	0.70892	0.00016	35.86	0.70916	0.70918	0.00015
28.92	0.70885	0.70894	0.00017	35.81	0.70911	0.70915	0.00016
28.87	0.70918	0.70899	0.00018	35.77	0.70901	0.70921	0.00020
28.83	0.70889	0.70898	0.00018	35.73	0.70915	0.70926	0.00020
28.79	0.70872	0.70899	0.00018	35.68	0.70890	0.70926	0.00020
28.74	0.70913	0.70907	0.00020	35.64	0.70967	0.70929	0.00019
28.70	0.70848	0.70903	0.00021	35.60	0.70896	0.70925	0.00017
28.66	0.70859	0.70908	0.00017	35.55	0.70927	0.70932	0.00017
28.61	0.70913	0.70914	0.00013	35.51	0.70904	0.70930	0.00017
28.57	0.70921	0.70912	0.00014	35.47	0.70948	0.70928	0.00020
28.53	0.70922	0.70912	0.00014	35.42	0.70890	0.70924	0.00019
28.48	0.70930	0.70910	0.00014	35.38	0.70974	0.70932	0.00019
28.44	0.70917	0.70898	0.00024	35.34	0.70950	0.70925	0.00018
28.40	0.70900	0.70897	0.00024	35.29	0.70913	0.70922	0.00017
28.35	0.70950	0.70894	0.00024	35.25	0.70926	0.70924	0.00017
28.31	0.70871	0.70884	0.00022	35.21	0.70919	0.70918	0.00020
28.27	0.70898	0.70887	0.00022	35.16	0.70964	0.70919	0.00021
28.22	0.70921	0.70890	0.00024	35.12	0.70915	0.70914	0.00018
28.18	0.70893	0.70888	0.00023	35.08	0.70880	0.70918	0.00020
28.14	0.70917	0.70884	0.00024	35.03	0.70913	0.70923	0.00018
28.09	0.70905	0.70879	0.00023	34.99	0.70970	0.70928	0.00019
28.05	0.70808	0.70879	0.00023	34.95	0.70905	0.70915	0.00023
28.01	0.70909	0.70883	0.00019	34.90	0.70911	0.70918	0.00023
27.96	0.70868	0.70886	0.00021	34.86	0.70932	0.70914	0.00025
27.92	0.70853	0.70885	0.00022	34.82	0.70866	0.70909	0.00026
27.88	0.70894	0.70891	0.00021	34.78	0.70938	0.70913	0.00024
27.83	0.70936	0.70892	0.00021	34.73	0.70911	0.70908	0.00024

27.79	0.70893	0.70889	0.00019	34.69	0.70957	0.70907	0.00024
27.75	0.70852	0.70889	0.00019	34.65	0.70927	0.70902	0.00021
27.70	0.70872	0.70892	0.00018	34.60	0.70958	0.70906	0.00024
27.66	0.70910	0.70898	0.00019	34.56	0.70844	0.70899	0.00021
27.62	0.70844	0.70901	0.00021	34.52	0.70935	0.70902	0.00019
27.58	0.70936	0.70905	0.00017	34.47	0.70871	0.70895	0.00018
27.53	0.70859	0.70897	0.00018	34.43	0.70882	0.70900	0.00018
27.49	0.70914	0.70897	0.00018	34.39	0.70910	0.70903	0.00017
27.45	0.70907	0.70897	0.00018	34.34	0.70880	0.70904	0.00018
27.40	0.70903	0.70898	0.00019	34.30	0.70905	0.70907	0.00017
27.36	0.70890	0.70900	0.00019	34.26	0.70909	0.70902	0.00020
27.32	0.70882	0.70901	0.00019	34.21	0.70961	0.70903	0.00020
27.27	0.70930	0.70906	0.00019	34.17	0.70894	0.70900	0.00017
27.23	0.70943	0.70901	0.00020	34.13	0.70868	0.70904	0.00018
27.19	0.70890	0.70894	0.00018	34.08	0.70870	0.70903	0.00019
27.14	0.70850	0.70895	0.00018	34.04	0.70924	0.70905	0.00018
27.10	0.70861	0.70905	0.00019	34.00	0.70908	0.70904	0.00018
27.06	0.70912	0.70911	0.00016	33.95	0.70924	0.70904	0.00018
27.01	0.70920	0.70907	0.00018	33.91	0.70905	0.70905	0.00018
26.97	0.70921	0.70904	0.00018	33.87	0.70854	0.70904	0.00018
26.93	0.70907	0.70908	0.00020	33.82	0.70921	0.70908	0.00014
26.88	0.70930	0.70907	0.00021	33.78	0.70928	0.70907	0.00014
26.84	0.70874	0.70905	0.00020	33.74	0.70935	0.70905	0.00013
26.80	0.70873	0.70911	0.00020	33.69	0.70861	0.70908	0.00016
26.75	0.70899	0.70913	0.00018	33.65	0.70887	0.70913	0.00012
26.71	0.70956	0.70914	0.00018	33.61	0.70914	0.70910	0.00017
26.67	0.70922	0.70911	0.00016	33.56	0.70914	0.70905	0.00019
26.62	0.70872	0.70905	0.00018	33.52	0.70928	0.70907	0.00019
26.58	0.70890	0.70904	0.00019	33.48	0.70897	0.70904	0.00018
26.54	0.70956	0.70905	0.00019	33.43	0.70900	0.70907	0.00019
26.49	0.70897	0.70901	0.00015	33.39	0.70905	0.70911	0.00020
26.45	0.70907	0.70899	0.00016	33.35	0.70914	0.70911	0.00020
26.41	0.70937	0.70893	0.00019	33.30	0.70956	0.70913	0.00021
26.36	0.70895	0.70890	0.00016	33.26	0.70920	0.70905	0.00020
26.32	0.70903	0.70892	0.00017	33.22	0.70851	0.70904	0.00020
26.28	0.70927	0.70896	0.00020	33.17	0.70869	0.70911	0.00016
26.24	0.70863	0.70890	0.00019	33.13	0.70927	0.70917	0.00013
26.19	0.70863	0.70893	0.00018	33.09	0.70904	0.70910	0.00017
26.15	0.70906	0.70895	0.00017	33.05	0.70923	0.70908	0.00018
26.11	0.70915	0.70899	0.00020	33.00	0.70945	0.70905	0.00018
26.06	0.70875	0.70898	0.00020	32.96	0.70899	0.70903	0.00016
26.02	0.70849	0.70896	0.00021	32.92	0.70935	0.70906	0.00017
25.98	0.70900	0.70907	0.00022	32.87	0.70873	0.70903	0.00016
25.93	0.70918	0.70911	0.00023	32.83	0.70914	0.70903	0.00016
25.89	0.70943	0.70909	0.00022	32.79	0.70919	0.70903	0.00015
25.85	0.70869	0.70907	0.00021	32.74	0.70933	0.70900	0.00015
25.80	0.70889	0.70908	0.00020	32.70	0.70859	0.70896	0.00013
25.76	0.70883	0.70911	0.00020	32.66	0.70883	0.70897	0.00012
25.72	0.70945	0.70916	0.00019	32.61	0.70893	0.70898	0.00012
25.67	0.70908	0.70905	0.00023	32.57	0.70923	0.70899	0.00012

25.63	0.70855	0.70906	0.00023	32.53	0.70925	0.70896	0.00010
25.59	0.70958	0.70908	0.00022	32.48	0.70907	0.70900	0.00017
25.54	0.70938	0.70904	0.00019	32.44	0.70873	0.70901	0.00017
25.50	0.70904	0.70898	0.00018	32.40	0.70909	0.70905	0.00016
25.46	0.70916	0.70893	0.00020	32.35	0.70891	0.70900	0.00018
25.41	0.70886	0.70898	0.00025	32.31	0.70892	0.70900	0.00018
25.37	0.70914	0.70898	0.00025	32.27	0.70871	0.70902	0.00018
25.33	0.70934	0.70896	0.00025	32.22	0.70899	0.70907	0.00017
25.28	0.70839	0.70895	0.00024	32.18	0.70896	0.70910	0.00017
25.24	0.70917	0.70903	0.00021	32.14	0.70899	0.70906	0.00020
25.20	0.70875	0.70901	0.00021	32.09	0.70966	0.70905	0.00020
25.15	0.70919	0.70901	0.00021	32.05	0.70911	0.70897	0.00016
25.11	0.70876	0.70899	0.00021	32.01	0.70914	0.70895	0.00015
25.07	0.70855	0.70905	0.00021	31.96	0.70858	0.70897	0.00017
25.03	0.70970	0.70913	0.00019	31.92	0.70898	0.70905	0.00016
24.98	0.70880	0.70907	0.00014	31.88	0.70906	0.70906	0.00016
24.94	0.70900	0.70914	0.00015	31.83	0.70925	0.70911	0.00019
24.90	0.70914	0.70918	0.00015	31.79	0.70926	0.70913	0.00020
24.85	0.70928	0.70919	0.00015	31.75	0.70858	0.70912	0.00020
24.81	0.70892	0.70915	0.00016	31.70	0.70893	0.70918	0.00016
24.77	0.70875	0.70914	0.00017	31.66	0.70881	0.70915	0.00018
24.72	0.70899	0.70920	0.00015	31.62	0.70893	0.70917	0.00016
24.68	0.70932	0.70921	0.00014	31.57	0.70937	0.70921	0.00015
24.64	0.70939	0.70918	0.00015	31.53	0.70934	0.70916	0.00016
24.59	0.70907	0.70914	0.00014	31.49	0.70907	0.70917	0.00017
24.55	0.70953	0.70914	0.00014	31.44	0.70957	0.70922	0.00018
24.51	0.70938	0.70911	0.00011	31.40	0.70941	0.70916	0.00017
24.46	0.70923	0.70910	0.00011	31.36	0.70925	0.70918	0.00018
24.42	0.70890	0.70906	0.00012	31.31	0.70909	0.70914	0.00019
24.38	0.70883	0.70909	0.00012	31.27	0.70872	0.70910	0.00021
24.33	0.70936	0.70915	0.00012	31.23	0.70901	0.70912	0.00020
24.29	0.70907	0.70910	0.00012	31.19	0.70927	0.70913	0.00020
24.25	0.70903	0.70905	0.00016	31.14	0.70887	0.70912	0.00020
24.20	0.70898	0.70904	0.00016	31.10	0.70948	0.70914	0.00019
24.16	0.70911	0.70900	0.00018	31.06	0.70952	0.70906	0.00019
24.12	0.70916	0.70899	0.00018	31.01	0.70904	0.70902	0.00016
24.07	0.70936	0.70893	0.00019	30.97	0.70955	0.70904	0.00017
24.03	0.70881	0.70888	0.00018	30.93	0.70892	0.70900	0.00013
23.99	0.70922	0.70891	0.00018	30.88	0.70860	0.70897	0.00015
23.94	0.70938	0.70892	0.00018	30.84	0.70895	0.70904	0.00014
23.90	0.70886	0.70887	0.00014	30.80	0.70911	0.70909	0.00016
23.86	0.70857	0.70890	0.00015	30.75	0.70921	0.70908	0.00016
23.81	0.70894	0.70896	0.00014	30.71	0.70900	0.70908	0.00016
23.77	0.70862	0.70900	0.00016	30.67	0.70874	0.70907	0.00016
23.73	0.70894	0.70907	0.00014	30.62	0.70908	0.70912	0.00015
23.69	0.70862	0.70907	0.00014	30.58	0.70923	0.70914	0.00015
23.64	0.70904	0.70915	0.00013	30.54	0.70912	0.70915	0.00015
23.60	0.70926	0.70911	0.00015	30.49	0.70862	0.70909	0.00020
23.56	0.70896	0.70908	0.00015	30.45	0.70932	0.70914	0.00018
23.51	0.70911	0.70912	0.00015	30.41	0.70948	0.70907	0.00020

23.47	0.70910	0.70915	0.00017
23.43	0.70931	0.70918	0.00017
23.38	0.70926	0.70917	0.00017
23.34	0.70894	0.70910	0.00020
23.30	0.70955	0.70913	0.00020
23.25	0.70892	0.70907	0.00018
23.21	0.70872	0.70912	0.00019
23.17	0.70898	0.70910	0.00022
23.12	0.70927	0.70914	0.00022
23.08	0.70948	0.70914	0.00022
23.04	0.70933	0.70908	0.00021
22.99	0.70922	0.70907	0.00021
22.95	0.70863	0.70909	0.00022
22.91	0.70926	0.70920	0.00022
22.86	0.70890	0.70919	0.00022
22.82	0.70946	0.70918	0.00023
22.78	0.70849	0.70915	0.00022
22.73	0.70935	0.70922	0.00016
22.69	0.70931	0.70917	0.00018
22.65	0.70889	0.70916	0.00018
22.60	0.70919	0.70918	0.00017
22.56	0.70943	0.70912	0.00019
22.52	0.70967	0.70911	0.00019
22.48	0.70925	0.70904	0.00014
22.43	0.70875	0.70902	0.00013
22.39	0.70921	0.70902	0.00013
22.35	0.70915	0.70902	0.00013
22.30	0.70883	0.70902	0.00013
22.26	0.70926	0.70910	0.00016
22.22	0.70903	0.70908	0.00016
22.17	0.70868	0.70905	0.00018
22.13	0.70930	0.70909	0.00016
22.09	0.70900	0.70913	0.00020
22.04	0.70902	0.70915	0.00020
22.00	0.70876	0.70915	0.00020
21.96	0.70920	0.70922	0.00019
21.91	0.70914	0.70922	0.00019
21.87	0.70957	0.70919	0.00020
21.83	0.70914	0.70920	0.00020
21.78	0.70870	0.70926	0.00023
21.74	0.70906	0.70932	0.00020
21.70	0.70972	0.70934	0.00019
21.65	0.70913	0.70923	0.00021
21.61	0.70904	0.70921	0.00022
21.57	0.70951	0.70923	0.00022
21.52	0.70914	0.70919	0.00021
21.48	0.70892	0.70918	0.00021
21.44	0.70963	0.70919	0.00021
21.39	0.70976	0.70918	0.00020
21.31	0.70933	0.70916	0.00018

30.36	0.70899	0.70901	0.00018
30.32	0.70918	0.70913	0.00029
30.28	0.70894	0.70910	0.00029
30.23	0.70927	0.70912	0.00029
30.19	0.70926	0.70912	0.00029
30.15	0.70932	0.70908	0.00029
30.10	0.70849	0.70902	0.00030
30.06	0.70917	0.70903	0.00029
30.02	0.70861	0.70902	0.00029
29.97	0.70893	0.70906	0.00027
29.93	0.71014	0.70905	0.00028
29.89	0.70886	0.70895	0.00014
29.84	0.70911	0.70891	0.00017
29.80	0.70930	0.70892	0.00018
29.76	0.70886	0.70890	0.00016
29.71	0.70870	0.70884	0.00021
29.67	0.70861	0.70887	0.00021
29.63	0.70904	0.70893	0.00021
29.58	0.70905	0.70893	0.00021
29.54	0.70877	0.70897	0.00024
29.50	0.70918	0.70894	0.00025
29.46	0.70847	0.70892	0.00025
29.41	0.70926	0.70899	0.00023
29.37	0.70909	0.70902	0.00026
29.33	0.70825	0.70903	0.00026
29.28	0.70899	0.70909	0.00020
29.24	0.70925	0.70914	0.00021
29.20	0.70902	0.70912	0.00021
29.15	0.70945	0.70914	0.00021
29.11	0.70849	0.70913	0.00020
29.07	0.70895	0.70923	0.00015
29.02	0.70915	0.70926	0.00014
28.98	0.70960	0.70926	0.00014
28.94	0.70910	0.70927	0.00015
28.89	0.70888	0.70926	0.00016
28.85	0.70949	0.70931	0.00013
28.81	0.70909	0.70924	0.00016
28.76	0.70915	0.70931	0.00019
28.72	0.70940	0.70933	0.00019
28.68	0.70943	0.70926	0.00022
28.63	0.70935	0.70925	0.00022
28.59	0.70908	0.70917	0.00026
28.55	0.70969	0.70920	0.00026
28.50	0.70904	0.70914	0.00024
28.46	0.70933	0.70913	0.00024
28.42	0.70883	0.70906	0.00026
28.37	0.70984	0.70900	0.00031
28.33	0.70929	0.70890	0.00024
28.29	0.70873	0.70886	0.00023
28.24	0.70931	0.70890	0.00023

21.26	0.70922	0.70912	0.00018	28.20	0.70853	0.70889	0.00023
21.22	0.70864	0.70912	0.00019	28.16	0.70942	0.70892	0.00022
21.18	0.70890	0.70922	0.00018	28.11	0.70904	0.70887	0.00018
21.14	0.70929	0.70929	0.00018	28.07	0.70897	0.70888	0.00019
21.09	0.70902	0.70930	0.00018	28.03	0.70866	0.70887	0.00019
21.05	0.70913	0.70937	0.00018	27.98	0.70820	0.70892	0.00019
21.01	0.70901	0.70945	0.00020	27.94	0.70881	0.70895	0.00014
20.96	0.70951	0.70949	0.00018	27.90	0.70892	0.70895	0.00014
20.92	0.70958	0.70946	0.00019	27.85	0.70911	0.70897	0.00014
20.88	0.70887	0.70944	0.00019	27.81	0.70928	0.70898	0.00015
20.83	0.70928	0.70947	0.00015	27.77	0.70881	0.70893	0.00015
20.79	0.70967	0.70952	0.00015	27.72	0.70889	0.70897	0.00015
20.75	0.70960	0.70943	0.00020	27.68	0.70913	0.70899	0.00015
20.70	0.70939	0.70941	0.00020	27.64	0.70890	0.70892	0.00018
20.66	0.70964	0.70939	0.00020	27.60	0.70911	0.70895	0.00019
20.62	0.70995	0.70936	0.00020	27.55	0.70850	0.70893	0.00019
20.57	0.70944	0.70931	0.00015	27.51	0.70881	0.70897	0.00016
20.53	0.70920	0.70933	0.00016	27.47	0.70914	0.70897	0.00016
20.49	0.70938	0.70936	0.00017	27.42	0.70926	0.70893	0.00017
20.44	0.70921	0.70938	0.00017	27.38	0.70874	0.70891	0.00015
20.40	0.70973	0.70941	0.00017	27.34	0.70919	0.70890	0.00016
20.36	0.70882	0.70940	0.00016	27.29	0.70907	0.70887	0.00015
20.31	0.70937	0.70945	0.00010	27.25	0.70843	0.70887	0.00015
20.27	0.70922	0.70942	0.00012	27.21	0.70919	0.70891	0.00011
20.23	0.70924	0.70944	0.00011	27.16	0.70898	0.70890	0.00011
20.18	0.70945	0.70948	0.00011	27.12	0.70886	0.70888	0.00011
20.14	0.70966	0.70946	0.00012	27.08	0.70885	0.70889	0.00011
20.10	0.70956	0.70944	0.00011	27.03	0.70872	0.70894	0.00014
20.05	0.70957	0.70937	0.00016	26.99	0.70909	0.70897	0.00013
20.01	0.70945	0.70937	0.00016	26.95	0.70864	0.70895	0.00013
19.97	0.70964	0.70937	0.00016	26.90	0.70885	0.70902	0.00013
19.93	0.70931	0.70935	0.00014	26.86	0.70908	0.70901	0.00013
19.88	0.70911	0.70935	0.00014	26.82	0.70880	0.70899	0.00014
19.84	0.70939	0.70937	0.00013	26.77	0.70918	0.70901	0.00013
19.80	0.70967	0.70935	0.00014	26.73	0.70875	0.70902	0.00014
19.75	0.70926	0.70934	0.00013	26.69	0.70897	0.70907	0.00013
19.71	0.70948	0.70932	0.00014	26.64	0.70929	0.70905	0.00014
19.67	0.70887	0.70932	0.00015	26.60	0.70904	0.70901	0.00013
19.62	0.70955	0.70937	0.00011	26.56	0.70889	0.70901	0.00013
19.58	0.70939	0.70934	0.00010	26.51	0.70934	0.70897	0.00017
19.54	0.70944	0.70931	0.00011	26.47	0.70877	0.70896	0.00016
19.49	0.70935	0.70936	0.00017	26.43	0.70886	0.70898	0.00015
19.45	0.70932	0.70937	0.00017	26.38	0.70903	0.70898	0.00016
19.41	0.70917	0.70937	0.00017	26.34	0.70925	0.70899	0.00016
19.36	0.70958	0.70943	0.00018	26.30	0.70927	0.70901	0.00018
19.32	0.70906	0.70942	0.00017	26.25	0.70882	0.70895	0.00019
19.28	0.70952	0.70942	0.00017	26.21	0.70889	0.70887	0.00027
19.23	0.70930	0.70952	0.00028	26.17	0.70897	0.70884	0.00028
19.19	0.70925	0.70955	0.00027	26.12	0.70847	0.70885	0.00028
19.15	0.70912	0.70954	0.00028	26.08	0.70924	0.70888	0.00027

19.10	0.70994	0.70961	0.00027	26.04	0.70898	0.70884	0.00025
19.06	0.70945	0.70961	0.00027	25.99	0.70883	0.70885	0.00026
19.02	0.70932	0.70959	0.00028	25.95	0.70916	0.70881	0.00028
18.97	0.70975	0.70961	0.00028	25.91	0.70950	0.70881	0.00028
18.93	0.70951	0.70962	0.00028	25.87	0.70865	0.70878	0.00025
18.89	0.70907	0.70964	0.00028	25.82	0.70799	0.70880	0.00025
18.84	0.71052	0.70969	0.00025	25.78	0.70858	0.70891	0.00017
18.80	0.70954	0.70958	0.00018	25.74	0.70908	0.70896	0.00016
18.76	0.70914	0.70958	0.00018	25.69	0.70878	0.70892	0.00016
18.71	0.70982	0.70959	0.00017	25.65	0.70884	0.70899	0.00019
18.67	0.71001	0.70953	0.00017	25.61	0.70912	0.70901	0.00019
18.63	0.70921	0.70948	0.00014	25.56	0.70836	0.70904	0.00020
18.59	0.70950	0.70954	0.00014	25.52	0.70918	0.70913	0.00013
18.54	0.70984	0.70953	0.00014	25.48	0.70920	0.70912	0.00013
18.50	0.70978	0.70951	0.00013	25.43	0.70889	0.70906	0.00016
18.46	0.70952	0.70942	0.00017	25.39	0.70904	0.70904	0.00018
18.41	0.70946	0.70940	0.00017	25.35	0.70909	0.70906	0.00019
18.37	0.70956	0.70937	0.00017	25.30	0.70874	0.70905	0.00019
18.33	0.70923	0.70936	0.00017	25.26	0.70943	0.70906	0.00018
18.28	0.70922	0.70938	0.00017	25.22	0.70911	0.70902	0.00016
18.24	0.70945	0.70939	0.00016	25.17	0.70938	0.70903	0.00016
18.20	0.70982	0.70935	0.00017	25.13	0.70927	0.70896	0.00015
18.15	0.70942	0.70936	0.00019	25.09	0.70901	0.70894	0.00014
18.11	0.70964	0.70942	0.00023	25.04	0.70868	0.70894	0.00014
18.07	0.70883	0.70946	0.00026	25.00	0.70862	0.70899	0.00014
18.02	0.70934	0.70954	0.00022	24.96	0.70927	0.70906	0.00013
17.98	0.70924	0.70958	0.00022	24.91	0.70896	0.70905	0.00012
17.94	0.70941	0.70963	0.00021	24.87	0.70884	0.70910	0.00014
17.89	0.70941	0.70961	0.00022	24.83	0.70908	0.70913	0.00013
17.85	0.70932	0.70968	0.00023	24.78	0.70917	0.70910	0.00015
17.81	0.70907	0.70968	0.00023	24.74	0.70869	0.70908	0.00015
17.76	0.70993	0.70971	0.00020	24.70	0.70911	0.70910	0.00013
17.72	0.71002	0.70964	0.00021	24.65	0.70894	0.70914	0.00015
17.68	0.71005	0.70960	0.00020	24.61	0.70919	0.70917	0.00014
17.63	0.70958	0.70960	0.00019	24.57	0.70934	0.70913	0.00016
17.59	0.70973	0.70956	0.00021	24.52	0.70922	0.70911	0.00015
17.55	0.70981	0.70955	0.00021	24.48	0.70938	0.70912	0.00016
17.50	0.70921	0.70955	0.00020	24.44	0.70916	0.70910	0.00015
17.46	0.71003	0.70962	0.00020	24.39	0.70876	0.70907	0.00015
17.42	0.70935	0.70955	0.00019	24.35	0.70896	0.70911	0.00014
17.37	0.70942	0.70956	0.00018	24.31	0.70892	0.70912	0.00013
17.33	0.70919	0.70957	0.00018	24.26	0.70947	0.70911	0.00014
17.29	0.70965	0.70964	0.00017	24.22	0.70929	0.70904	0.00013
17.25	0.71001	0.70964	0.00017	24.18	0.70882	0.70901	0.00011
17.20	0.70921	0.70958	0.00015	24.14	0.70909	0.70902	0.00011
17.16	0.70964	0.70965	0.00014	24.09	0.70930	0.70905	0.00012
17.12	0.70977	0.70963	0.00015	24.05	0.70923	0.70899	0.00012
17.07	0.70995	0.70965	0.00017	24.01	0.70887	0.70896	0.00012
17.03	0.70934	0.70965	0.00017	23.96	0.70911	0.70896	0.00011
16.99	0.70946	0.70971	0.00016	23.92	0.70907	0.70893	0.00012

16.94	0.70946	0.70974	0.00015	23.88	0.70885	0.70891	0.00011
16.90	0.70991	0.70984	0.00020	23.83	0.70878	0.70894	0.00012
16.86	0.70964	0.70981	0.00020	23.79	0.70902	0.70897	0.00012
16.81	0.70943	0.70983	0.00019	23.75	0.70893	0.70896	0.00012
16.77	0.70992	0.70986	0.00018	23.70	0.70935	0.70899	0.00013
16.73	0.70939	0.70984	0.00018	23.66	0.70874	0.70897	0.00011
16.68	0.71003	0.70987	0.00015	23.62	0.70886	0.70895	0.00013
16.64	0.70989	0.70980	0.00017	23.57	0.70893	0.70893	0.00014
16.60	0.70995	0.70979	0.00017	23.53	0.70874	0.70894	0.00014
16.55	0.70975	0.70975	0.00017	23.49	0.70892	0.70895	0.00014
16.51	0.71045	0.70971	0.00019	23.44	0.70910	0.70893	0.00015
16.47	0.70966	0.70961	0.00010	23.40	0.70914	0.70889	0.00015
16.42	0.70985	0.70962	0.00010	23.36	0.70888	0.70886	0.00014
16.38	0.70971	0.70958	0.00009	23.31	0.70926	0.70888	0.00014
16.34	0.70974	0.70963	0.00015	23.27	0.70909	0.70885	0.00012
16.29	0.70962	0.70963	0.00015	23.23	0.70859	0.70882	0.00011
16.25	0.70940	0.70962	0.00015	23.18	0.70864	0.70889	0.00013
16.21	0.70974	0.70962	0.00016	23.14	0.70901	0.70894	0.00013
16.16	0.70959	0.70955	0.00019	23.10	0.70890	0.70896	0.00014
16.12	0.70933	0.70951	0.00020	23.05	0.70868	0.70898	0.00014
16.08	0.70950	0.70953	0.00020	23.01	0.70874	0.70900	0.00013
16.04	0.70969	0.70958	0.00021	22.97	0.70878	0.70908	0.00014
15.99	0.70951	0.70957	0.00021	22.92	0.70911	0.70907	0.00015
15.95	0.71020	0.70955	0.00022	22.88	0.70897	0.70903	0.00017
15.91	0.70968	0.70948	0.00017	22.84	0.70876	0.70903	0.00017
15.86	0.70959	0.70945	0.00016	22.79	0.70931	0.70903	0.00016
15.82	0.70934	0.70946	0.00016	22.75	0.70911	0.70903	0.00016
15.78	0.70905	0.70946	0.00016	22.71	0.70925	0.70900	0.00016
15.73	0.70923	0.70955	0.00016	22.66	0.70907	0.70896	0.00015
15.69	0.70949	0.70958	0.00015	22.62	0.70892	0.70898	0.00016
15.65	0.70996	0.70954	0.00017	22.58	0.70947	0.70893	0.00019
15.60	0.70962	0.70950	0.00014	22.53	0.70870	0.70891	0.00016
15.56	0.70930	0.70952	0.00016	22.49	0.70872	0.70891	0.00016
15.52	0.70958	0.70961	0.00020	22.45	0.70896	0.70895	0.00016
15.47	0.70937	0.70960	0.00020	22.40	0.70880	0.70891	0.00017
15.43	0.70964	0.70960	0.00020	22.36	0.70925	0.70893	0.00017
15.39	0.70937	0.70960	0.00020	22.32	0.70887	0.70891	0.00016
15.34	0.70997	0.70960	0.00020	22.28	0.70884	0.70891	0.00016
15.30	0.70945	0.70955	0.00018	22.23	0.70926	0.70891	0.00016
15.26	0.70913	0.70955	0.00018	22.19	0.70848	0.70887	0.00014
15.21	0.70956	0.70958	0.00016	22.15	0.70921	0.70892	0.00011
15.17	0.70982	0.70955	0.00018	22.10	0.70877	0.70892	0.00012
15.13	0.71019	0.70951	0.00017	22.06	0.70906	0.70895	0.00012
15.08	0.70956	0.70944	0.00008	22.02	0.70858	0.70890	0.00014
15.04	0.70937	0.70942	0.00007	21.97	0.70897	0.70893	0.00012
15.00	0.70960	0.70944	0.00007	21.93	0.70911	0.70890	0.00013
14.95	0.70935	0.70942	0.00006	21.89	0.70882	0.70887	0.00012
14.91	0.70944	0.70950	0.00017	21.84	0.70886	0.70893	0.00015
14.87	0.70947	0.70954	0.00018	21.80	0.70885	0.70895	0.00015
14.82	0.70948	0.70954	0.00018	21.76	0.70897	0.70893	0.00016

14.78	0.70917	0.70952	0.00019
14.74	0.70942	0.70954	0.00017
14.70	0.70952	0.70960	0.00019
14.65	0.70940	0.70960	0.00019
14.61	0.70951	0.70966	0.00020
14.57	0.70940	0.70963	0.00022
14.52	0.71019	0.70966	0.00021
14.48	0.70984	0.70959	0.00018
14.44	0.70942	0.70959	0.00018
14.39	0.70929	0.70958	0.00018
14.35	0.70940	0.70963	0.00017
14.31	0.71000	0.70966	0.00016
14.26	0.70952	0.70962	0.00014
14.22	0.71003	0.70960	0.00015
14.18	0.70925	0.70951	0.00014
14.13	0.70965	0.70955	0.00013
14.09	0.70948	0.70952	0.00013
14.05	0.70982	0.70954	0.00013
14.00	0.70941	0.70950	0.00012
13.96	0.70975	0.70947	0.00014
13.92	0.70966	0.70941	0.00014
13.87	0.70959	0.70943	0.00016
13.83	0.70938	0.70941	0.00016
13.79	0.70915	0.70943	0.00016
13.74	0.70965	0.70948	0.00015
13.70	0.70937	0.70948	0.00015
13.66	0.70962	0.70950	0.00015
13.61	0.70944	0.70952	0.00016
13.57	0.70907	0.70952	0.00016
13.53	0.70918	0.70957	0.00012
13.49	0.70986	0.70963	0.00009
13.44	0.70941	0.70958	0.00009
13.40	0.70956	0.70958	0.00009
13.36	0.70964	0.70959	0.00009
13.31	0.70962	0.70962	0.00012
13.27	0.70960	0.70967	0.00016
13.23	0.70977	0.70964	0.00017
13.18	0.70945	0.70966	0.00018
13.14	0.70955	0.70967	0.00018
13.10	0.70980	0.70970	0.00018
13.05	0.70934	0.70968	0.00018
13.01	0.70946	0.70968	0.00018
12.97	0.70966	0.70973	0.00018
12.92	0.70994	0.70971	0.00019
12.88	0.71015	0.70968	0.00018
12.84	0.70933	0.70965	0.00016
12.79	0.70998	0.70963	0.00019
12.75	0.70954	0.70959	0.00017
12.71	0.70982	0.70959	0.00017
12.66	0.70964	0.70959	0.00017

21.71	0.70924	0.70893	0.00016
21.67	0.70904	0.70894	0.00017
21.63	0.70857	0.70893	0.00017
21.58	0.70890	0.70899	0.00015
21.54	0.70867	0.70896	0.00017
21.50	0.70882	0.70902	0.00016
21.45	0.70938	0.70905	0.00016
21.41	0.70903	0.70898	0.00016
21.37	0.70867	0.70891	0.00020
21.32	0.70896	0.70893	0.00019
21.28	0.70933	0.70895	0.00020
21.24	0.70899	0.70890	0.00018
21.19	0.70915	0.70888	0.00018
21.15	0.70864	0.70884	0.00017
21.11	0.70921	0.70890	0.00017
21.06	0.70915	0.70887	0.00016
21.02	0.70862	0.70884	0.00015
20.98	0.70837	0.70885	0.00015
20.93	0.70888	0.70892	0.00010
20.89	0.70914	0.70894	0.00010
20.85	0.70889	0.70895	0.00012
20.80	0.70880	0.70898	0.00013
20.76	0.70875	0.70899	0.00012
20.72	0.70917	0.70905	0.00013
20.67	0.70898	0.70899	0.00016
20.63	0.70886	0.70898	0.00016
20.59	0.70870	0.70902	0.00016
20.55	0.70905	0.70907	0.00015
20.50	0.70906	0.70905	0.00015
20.46	0.70929	0.70908	0.00017
20.42	0.70919	0.70906	0.00016
20.37	0.70887	0.70907	0.00016
20.33	0.70935	0.70910	0.00016
20.29	0.70854	0.70903	0.00016
20.24	0.70892	0.70907	0.00013
20.20	0.70918	0.70914	0.00015
20.16	0.70920	0.70910	0.00017
20.11	0.70890	0.70911	0.00017
20.07	0.70936	0.70914	0.00017
20.03	0.70910	0.70912	0.00016
19.98	0.70925	0.70912	0.00016
19.94	0.70916	0.70911	0.00016
19.90	0.70869	0.70908	0.00016
19.85	0.70896	0.70909	0.00015
19.81	0.70956	0.70905	0.00018
19.77	0.70881	0.70899	0.00015
19.72	0.70933	0.70903	0.00014
19.68	0.70922	0.70898	0.00013
19.64	0.70908	0.70897	0.00012
19.59	0.70916	0.70895	0.00012

12.62	0.70927	0.70957	0.00017
12.58	0.70995	0.70964	0.00017
12.53	0.70946	0.70958	0.00016
12.49	0.70966	0.70963	0.00017
12.45	0.70986	0.70960	0.00018
12.40	0.70907	0.70953	0.00019
12.36	0.70964	0.70958	0.00016
12.32	0.70955	0.70958	0.00016
12.27	0.70983	0.70955	0.00016
12.23	0.70945	0.70951	0.00015
12.19	0.70989	0.70951	0.00015
12.15	0.70940	0.70948	0.00013
12.10	0.70993	0.70952	0.00014
12.06	0.70938	0.70945	0.00012
12.02	0.70917	0.70946	0.00012
11.97	0.70955	0.70948	0.00010
11.93	0.70961	0.70951	0.00013
11.89	0.70932	0.70946	0.00015
11.84	0.70938	0.70948	0.00015
11.80	0.70944	0.70950	0.00015
11.76	0.70966	0.70949	0.00015
11.71	0.70976	0.70952	0.00018
11.67	0.70923	0.70953	0.00019
11.63	0.70946	0.70953	0.00018
11.58	0.70939	0.70953	0.00018
11.54	0.70986	0.70958	0.00019
11.50	0.70910	0.70950	0.00020
11.45	0.70951	0.70954	0.00018
11.41	0.70962	0.70953	0.00019
11.37	0.70929	0.70954	0.00019
11.32	0.71002	0.70957	0.00018
11.28	0.70979	0.70951	0.00015
11.24	0.70932	0.70949	0.00014
11.19	0.70941	0.70954	0.00015
11.15	0.70985	0.70956	0.00015
11.11	0.70906	0.70953	0.00013
11.06	0.70953	0.70957	0.00008
11.02	0.70937	0.70956	0.00009
10.98	0.70972	0.70958	0.00008
10.93	0.70958	0.70957	0.00008
10.89	0.70949	0.70956	0.00008
10.85	0.70958	0.70958	0.00008
10.81	0.70984	0.70956	0.00009
10.76	0.70962	0.70955	0.00008
10.72	0.70954	0.70953	0.00008
10.68	0.70946	0.70952	0.00009
10.63	0.70936	0.70951	0.00009
10.59	0.70960	0.70949	0.00011
10.55	0.70963	0.70947	0.00011
10.50	0.70950	0.70944	0.00011

19.55	0.70910	0.70893	0.00011
19.51	0.70892	0.70890	0.00011
19.46	0.70875	0.70888	0.00012
19.42	0.70859	0.70890	0.00011
19.38	0.70898	0.70892	0.00009
19.33	0.70918	0.70891	0.00009
19.29	0.70886	0.70890	0.00008
19.25	0.70910	0.70890	0.00008
19.20	0.70884	0.70892	0.00011
19.16	0.70901	0.70893	0.00011
19.12	0.70873	0.70892	0.00011
19.07	0.70875	0.70892	0.00011
19.03	0.70891	0.70898	0.00012
18.99	0.70881	0.70903	0.00015
18.94	0.70889	0.70903	0.00016
18.90	0.70909	0.70904	0.00015
18.86	0.70883	0.70907	0.00017
18.81	0.70928	0.70919	0.00024
18.77	0.70896	0.70919	0.00024
18.73	0.70896	0.70918	0.00024
18.69	0.70877	0.70910	0.00028
18.64	0.70923	0.70908	0.00029
18.60	0.70944	0.70905	0.00029
18.56	0.70875	0.70900	0.00028
18.51	0.70901	0.70904	0.00028
18.47	0.70934	0.70906	0.00028
18.43	0.70997	0.70900	0.00027
18.38	0.70923	0.70886	0.00018
18.34	0.70893	0.70882	0.00017
18.30	0.70838	0.70881	0.00016
18.25	0.70855	0.70879	0.00018
18.21	0.70895	0.70882	0.00017
18.17	0.70894	0.70880	0.00017
18.12	0.70914	0.70880	0.00017
18.08	0.70916	0.70876	0.00015
18.04	0.70879	0.70873	0.00013
17.99	0.70852	0.70874	0.00013
17.95	0.70889	0.70877	0.00012
17.91	0.70876	0.70875	0.00012
17.86	0.70825	0.70874	0.00012
17.82	0.70881	0.70880	0.00005
17.78	0.70874	0.70883	0.00008
17.73	0.70889	0.70887	0.00010
17.69	0.70882	0.70881	0.00014
17.65	0.70886	0.70887	0.00018
17.60	0.70883	0.70893	0.00021
17.56	0.70886	0.70893	0.00021
17.52	0.70868	0.70894	0.00021
17.47	0.70866	0.70888	0.00027
17.43	0.70880	0.70888	0.00027

10.46	0.70966	0.70950	0.00017
10.42	0.70939	0.70949	0.00016
10.37	0.70976	0.70948	0.00017
10.33	0.70940	0.70942	0.00016
10.29	0.70946	0.70942	0.00016
10.24	0.70934	0.70943	0.00017
10.20	0.70918	0.70946	0.00017
10.16	0.70939	0.70950	0.00016
10.11	0.70933	0.70949	0.00016
10.07	0.71008	0.70949	0.00016
10.03	0.70952	0.70942	0.00010
9.98	0.70932	0.70941	0.00010
9.94	0.70917	0.70946	0.00012
9.90	0.70941	0.70950	0.00010
9.85	0.70960	0.70949	0.00011
9.81	0.70960	0.70946	0.00011
9.77	0.70961	0.70942	0.00012
9.72	0.70927	0.70945	0.00016
9.68	0.70936	0.70948	0.00016
9.64	0.70930	0.70952	0.00016
9.60	0.70948	0.70953	0.00016
9.55	0.70979	0.70952	0.00016
9.51	0.70958	0.70947	0.00015
9.47	0.70930	0.70945	0.00015
9.42	0.70933	0.70943	0.00017
9.38	0.70914	0.70945	0.00017
9.34	0.70994	0.70946	0.00016
9.29	0.70960	0.70940	0.00012
9.25	0.70973	0.70934	0.00013
9.21	0.70942	0.70930	0.00010
9.16	0.70938	0.70929	0.00009
9.12	0.70932	0.70925	0.00010
9.08	0.70935	0.70924	0.00010
9.03	0.70907	0.70926	0.00011
8.99	0.70953	0.70927	0.00011
8.95	0.70929	0.70923	0.00009
8.90	0.70929	0.70926	0.00012
8.86	0.70902	0.70928	0.00012
8.82	0.70936	0.70932	0.00011
8.77	0.70927	0.70934	0.00012
8.73	0.70903	0.70940	0.00016
8.69	0.70924	0.70941	0.00015
8.64	0.70950	0.70944	0.00015
8.60	0.70917	0.70945	0.00015
8.56	0.70917	0.70945	0.00016
8.51	0.70959	0.70950	0.00015
8.47	0.70944	0.70948	0.00015
8.43	0.70947	0.70947	0.00015
8.38	0.70950	0.70947	0.00015
8.34	0.70991	0.70945	0.00016

17.39	0.70913	0.70889	0.00027
17.34	0.70911	0.70888	0.00027
17.30	0.70837	0.70885	0.00026
17.26	0.70942	0.70891	0.00024
17.21	0.70942	0.70889	0.00023
17.17	0.70886	0.70887	0.00021
17.13	0.70894	0.70888	0.00021
17.08	0.70806	0.70885	0.00022
17.04	0.70870	0.70896	0.00015
17.00	0.70886	0.70899	0.00013
16.96	0.70904	0.70903	0.00014
16.91	0.70885	0.70904	0.00014
16.87	0.70891	0.70906	0.00013
16.83	0.70928	0.70912	0.00016
16.78	0.70917	0.70906	0.00018
16.74	0.70902	0.70900	0.00020
16.70	0.70857	0.70893	0.00024
16.65	0.70925	0.70902	0.00025
16.61	0.70900	0.70899	0.00024
16.57	0.70919	0.70899	0.00024
16.52	0.70913	0.70899	0.00024
16.48	0.70906	0.70895	0.00024
16.44	0.70955	0.70896	0.00025
16.39	0.70863	0.70885	0.00023
16.35	0.70865	0.70890	0.00023
16.31	0.70830	0.70892	0.00023
16.26	0.70946	0.70899	0.00018
16.22	0.70899	0.70893	0.00014
16.18	0.70898	0.70894	0.00015
16.13	0.70911	0.70891	0.00015
16.09	0.70875	0.70887	0.00015
16.05	0.70920	0.70890	0.00016
16.00	0.70840	0.70888	0.00014
15.96	0.70913	0.70892	0.00010
15.92	0.70889	0.70888	0.00010
15.87	0.70894	0.70894	0.00015
15.83	0.70889	0.70892	0.00016
15.79	0.70908	0.70889	0.00017
15.74	0.70874	0.70890	0.00017
15.70	0.70866	0.70890	0.00017
15.66	0.70911	0.70891	0.00017
15.61	0.70896	0.70893	0.00019
15.57	0.70882	0.70896	0.00020
15.53	0.70870	0.70898	0.00019
15.48	0.70947	0.70900	0.00019
15.44	0.70877	0.70897	0.00016
15.40	0.70862	0.70899	0.00015
15.35	0.70917	0.70906	0.00014
15.31	0.70873	0.70902	0.00015
15.27	0.70873	0.70906	0.00013

8.30	0.70912	0.70938	0.00013	15.22	0.70935	0.70910	0.00011
8.26	0.70955	0.70938	0.00013	15.18	0.70925	0.70901	0.00016
8.21	0.70958	0.70938	0.00014	15.14	0.70898	0.70899	0.00015
8.17	0.70912	0.70934	0.00014	15.10	0.70890	0.70899	0.00015
8.13	0.70972	0.70933	0.00015	15.05	0.70915	0.70907	0.00020
8.08	0.70939	0.70931	0.00013	15.01	0.70904	0.70905	0.00020
8.04	0.70937	0.70930	0.00013	14.97	0.70929	0.70901	0.00022
8.00	0.70946	0.70934	0.00017	14.92	0.70881	0.70901	0.00021
7.95	0.70931	0.70932	0.00016	14.88	0.70906	0.70903	0.00021
7.91	0.70915	0.70932	0.00016	14.84	0.70922	0.70896	0.00026
7.87	0.70910	0.70936	0.00016	14.79	0.70843	0.70892	0.00025
7.82	0.70963	0.70939	0.00015	14.75	0.70904	0.70896	0.00023
7.78	0.70913	0.70939	0.00015	14.71	0.70901	0.70895	0.00023
7.74	0.70901	0.70937	0.00016	14.66	0.70967	0.70894	0.00023
7.69	0.70958	0.70946	0.00017	14.62	0.70892	0.70888	0.00016
7.65	0.70922	0.70944	0.00017	14.58	0.70870	0.70887	0.00016
7.61	0.70978	0.70944	0.00017	14.53	0.70927	0.70891	0.00016
7.56	0.70927	0.70936	0.00017	14.49	0.70903	0.70892	0.00018
7.52	0.70934	0.70938	0.00017	14.45	0.70828	0.70889	0.00018
7.48	0.70951	0.70941	0.00018	14.40	0.70882	0.70894	0.00013
7.43	0.70946	0.70940	0.00018	14.36	0.70890	0.70898	0.00014
7.39	0.70955	0.70939	0.00018	14.32	0.70893	0.70894	0.00017
7.35	0.70897	0.70935	0.00018	14.27	0.70886	0.70895	0.00017
7.30	0.70987	0.70939	0.00016	14.23	0.70906	0.70897	0.00017
7.26	0.70947	0.70935	0.00012	14.19	0.70888	0.70897	0.00017
7.22	0.70918	0.70938	0.00014	14.14	0.70908	0.70900	0.00017
7.17	0.70903	0.70938	0.00014	14.10	0.70940	0.70898	0.00017
7.13	0.70942	0.70942	0.00012	14.06	0.70866	0.70893	0.00014
7.09	0.70969	0.70944	0.00013	14.01	0.70879	0.70893	0.00014
7.05	0.70939	0.70939	0.00012	13.97	0.70923	0.70894	0.00014
7.00	0.70932	0.70936	0.00014	13.93	0.70851	0.70890	0.00012
6.96	0.70918	0.70935	0.00014	13.88	0.70900	0.70895	0.00009
6.92	0.70938	0.70936	0.00014	13.84	0.70905	0.70900	0.00013
6.87	0.70942	0.70934	0.00014	13.80	0.70912	0.70896	0.00015
6.83	0.70978	0.70933	0.00014	13.75	0.70916	0.70894	0.00015
6.79	0.70918	0.70927	0.00010	13.71	0.70890	0.70891	0.00014
6.74	0.70940	0.70926	0.00010	13.67	0.70884	0.70890	0.00014
6.70	0.70962	0.70924	0.00010	13.62	0.70867	0.70891	0.00014
6.66	0.70927	0.70923	0.00007	13.58	0.70889	0.70896	0.00014
6.61	0.70903	0.70924	0.00008	13.54	0.70890	0.70896	0.00014
6.57	0.70922	0.70927	0.00007	13.49	0.70903	0.70895	0.00014
6.53	0.70930	0.70922	0.00012	13.45	0.70944	0.70894	0.00014
6.48	0.70918	0.70919	0.00013	13.41	0.70866	0.70893	0.00012
6.44	0.70930	0.70923	0.00014	13.37	0.70890	0.70897	0.00011
6.40	0.70918	0.70922	0.00014	13.32	0.70890	0.70898	0.00010
6.35	0.70913	0.70919	0.00016	13.28	0.70879	0.70894	0.00015
6.31	0.70920	0.70922	0.00016	13.24	0.70892	0.70893	0.00015
6.27	0.70946	0.70922	0.00016	13.19	0.70916	0.70895	0.00016
6.22	0.70936	0.70920	0.00015	13.15	0.70887	0.70891	0.00015
6.18	0.70936	0.70913	0.00018	13.11	0.70888	0.70893	0.00016

6.14	0.70874	0.70912	0.00017	13.06	0.70890	0.70890	0.00017
6.09	0.70903	0.70917	0.00015	13.02	0.70933	0.70889	0.00017
6.05	0.70949	0.70919	0.00015	12.98	0.70909	0.70888	0.00016
6.01	0.70926	0.70917	0.00014	12.93	0.70894	0.70886	0.00015
5.96	0.70890	0.70918	0.00014	12.89	0.70847	0.70885	0.00015
5.92	0.70935	0.70923	0.00013	12.85	0.70872	0.70891	0.00013
5.88	0.70920	0.70922	0.00013	12.80	0.70913	0.70894	0.00012
5.83	0.70925	0.70921	0.00013	12.76	0.70873	0.70893	0.00012
5.79	0.70867	0.70920	0.00013	12.72	0.70906	0.70893	0.00012
5.75	0.70932	0.70926	0.00005	12.67	0.70864	0.70890	0.00012
5.71	0.70923	0.70928	0.00007	12.63	0.70876	0.70896	0.00011
5.66	0.70921	0.70932	0.00011	12.59	0.70923	0.70901	0.00012
5.62	0.70932	0.70932	0.00011	12.54	0.70892	0.70899	0.00011
5.58	0.70935	0.70933	0.00011	12.50	0.70882	0.70900	0.00011
5.53	0.70936	0.70935	0.00011	12.46	0.70907	0.70901	0.00010
5.49	0.70929	0.70932	0.00013	12.41	0.70905	0.70900	0.00010
5.45	0.70914	0.70927	0.00016	12.37	0.70903	0.70899	0.00010
5.40	0.70915	0.70931	0.00016	12.33	0.70869	0.70902	0.00012
5.36	0.70923	0.70930	0.00017	12.28	0.70884	0.70905	0.00010
5.32	0.70950	0.70926	0.00019	12.24	0.70916	0.70905	0.00010
5.27	0.70969	0.70921	0.00019	12.20	0.70926	0.70901	0.00011
5.23	0.70919	0.70917	0.00016	12.15	0.70911	0.70903	0.00013
5.19	0.70944	0.70920	0.00017	12.11	0.70896	0.70908	0.00017
5.14	0.70948	0.70916	0.00016	12.07	0.70888	0.70904	0.00019
5.10	0.70907	0.70918	0.00018	12.02	0.70899	0.70906	0.00019
5.06	0.70882	0.70923	0.00019	11.98	0.70896	0.70902	0.00021
5.01	0.70949	0.70926	0.00018	11.94	0.70932	0.70904	0.00021
4.97	0.70911	0.70926	0.00018	11.89	0.70903	0.70897	0.00021
4.93	0.70883	0.70925	0.00018	11.85	0.70887	0.70895	0.00022
4.88	0.70900	0.70928	0.00016	11.81	0.70877	0.70893	0.00023
4.84	0.70930	0.70935	0.00016	11.76	0.70944	0.70899	0.00024
4.80	0.70941	0.70933	0.00017	11.72	0.70957	0.70895	0.00022
4.75	0.70910	0.70935	0.00018	11.68	0.70860	0.70886	0.00018
4.71	0.70969	0.70935	0.00018	11.63	0.70908	0.70890	0.00017
4.67	0.70957	0.70934	0.00017	11.59	0.70859	0.70886	0.00017
4.62	0.70908	0.70933	0.00016	11.55	0.70912	0.70892	0.00017
4.58	0.70952	0.70933	0.00016	11.51	0.70868	0.70889	0.00017
4.54	0.70897	0.70929	0.00016	11.46	0.70882	0.70891	0.00016
4.50	0.70917	0.70929	0.00016	11.42	0.70858	0.70888	0.00018
4.45	0.70965	0.70926	0.00018	11.38	0.70938	0.70892	0.00017
4.41	0.70911	0.70925	0.00017	11.33	0.70905	0.70892	0.00017
4.37	0.70959	0.70930	0.00018	11.29	0.70872	0.70889	0.00017
4.32	0.70914	0.70924	0.00018	11.25	0.70900	0.70896	0.00019
4.28	0.70958	0.70923	0.00018	11.20	0.70866	0.70894	0.00019
4.24	0.70945	0.70918	0.00016	11.16	0.70921	0.70894	0.00019
4.19	0.70909	0.70917	0.00015	11.12	0.70878	0.70895	0.00020
4.15	0.70917	0.70916	0.00016	11.07	0.70887	0.70892	0.00022
4.11	0.70894	0.70919	0.00017	11.03	0.70849	0.70892	0.00022
4.06	0.70890	0.70923	0.00016	10.99	0.70899	0.70895	0.00021
4.02	0.70958	0.70926	0.00015	10.94	0.70942	0.70895	0.00021

3.98	0.70955	0.70922	0.00013
3.93	0.70897	0.70915	0.00012
3.89	0.70909	0.70918	0.00011
3.85	0.70907	0.70920	0.00011
3.80	0.70929	0.70921	0.00011
3.76	0.70898	0.70921	0.00011
3.72	0.70949	0.70921	0.00010
3.67	0.70936	0.70918	0.00009
3.63	0.70922	0.70913	0.00009
3.59	0.70916	0.70914	0.00010
3.54	0.70890	0.70919	0.00014
3.50	0.70926	0.70926	0.00015
3.46	0.70924	0.70926	0.00015
3.41	0.70916	0.70924	0.00015
3.37	0.70931	0.70928	0.00016
3.33	0.70904	0.70926	0.00016
3.28	0.70913	0.70931	0.00016
3.24	0.70889	0.70930	0.00017
3.20	0.70935	0.70935	0.00014
3.16	0.70960	0.70934	0.00014
3.11	0.70965	0.70926	0.00017
3.07	0.70919	0.70922	0.00015
3.03	0.70909	0.70917	0.00018
2.98	0.70954	0.70916	0.00018
2.94	0.70914	0.70913	0.00016
2.90	0.70952	0.70917	0.00018
2.85	0.70901	0.70919	0.00019
2.81	0.70937	0.70920	0.00019
2.77	0.70931	0.70915	0.00020
2.72	0.70877	0.70917	0.00021
2.68	0.70923	0.70918	0.00020
2.64	0.70871	0.70918	0.00020
2.59	0.70904	0.70920	0.00018
2.55	0.70925	0.70920	0.00018
2.51	0.70948	0.70923	0.00019
2.46	0.70969	0.70920	0.00018
2.42	0.70911	0.70919	0.00016
2.38	0.70890	0.70918	0.00017
2.33	0.70952	0.70919	0.00016
2.29	0.70890	0.70919	0.00016
2.25	0.70922	0.70923	0.00015
2.20	0.70887	0.70923	0.00015
2.16	0.70907	0.70928	0.00013
2.12	0.70951	0.70928	0.00013
2.07	0.70924	0.70928	0.00013
2.03	0.70952	0.70924	0.00016
1.99	0.70902	0.70923	0.00015
1.94	0.70901	0.70927	0.00015
1.90	0.70951	0.70930	0.00014
1.86	0.70934	0.70933	0.00017

10.90	0.70880	0.70890	0.00018
10.86	0.70939	0.70892	0.00018
10.81	0.70882	0.70884	0.00015
10.77	0.70864	0.70884	0.00015
10.73	0.70932	0.70887	0.00015
10.68	0.70847	0.70883	0.00011
10.64	0.70883	0.70890	0.00010
10.60	0.70878	0.70888	0.00011
10.55	0.70904	0.70892	0.00013
10.51	0.70888	0.70894	0.00014
10.47	0.70899	0.70893	0.00015
10.42	0.70860	0.70895	0.00016
10.38	0.70889	0.70896	0.00015
10.34	0.70893	0.70892	0.00018
10.29	0.70891	0.70894	0.00018
10.25	0.70917	0.70896	0.00018
10.21	0.70861	0.70892	0.00018
10.16	0.70924	0.70901	0.00020
10.12	0.70920	0.70893	0.00022
10.08	0.70875	0.70896	0.00024
10.03	0.70925	0.70899	0.00023
9.99	0.70871	0.70901	0.00024
9.95	0.70848	0.70905	0.00023
9.90	0.70914	0.70907	0.00021
9.86	0.70905	0.70901	0.00024
9.82	0.70881	0.70902	0.00025
9.78	0.70950	0.70907	0.00025
9.73	0.70844	0.70901	0.00023
9.69	0.70943	0.70907	0.00019
9.65	0.70912	0.70899	0.00019
9.60	0.70939	0.70899	0.00019
9.56	0.70913	0.70895	0.00017
9.52	0.70872	0.70889	0.00018
9.47	0.70847	0.70890	0.00018
9.43	0.70923	0.70893	0.00015
9.39	0.70926	0.70894	0.00016
9.34	0.70893	0.70890	0.00015
9.30	0.70897	0.70892	0.00015
9.26	0.70864	0.70893	0.00015
9.21	0.70911	0.70898	0.00014
9.17	0.70903	0.70898	0.00014
9.13	0.70854	0.70900	0.00015
9.08	0.70880	0.70905	0.00012
9.04	0.70875	0.70908	0.00010
9.00	0.70932	0.70908	0.00010
8.95	0.70893	0.70903	0.00010
8.91	0.70907	0.70905	0.00010
8.87	0.70906	0.70904	0.00010
8.82	0.70913	0.70905	0.00011
8.78	0.70914	0.70906	0.00011

1.82	0.70915	0.70932	0.00017
1.77	0.70940	0.70931	0.00018
1.73	0.70906	0.70932	0.00018
1.69	0.70956	0.70940	0.00020
1.64	0.70886	0.70942	0.00021
1.60	0.70943	0.70947	0.00017
1.56	0.70937	0.70949	0.00017
1.51	0.70932	0.70953	0.00018
1.47	0.70982	0.70954	0.00017
1.43	0.70921	0.70955	0.00018
1.38	0.70905	0.70964	0.00019
1.34	0.70953	0.70972	0.00014
1.30	0.70987	0.70972	0.00014
1.25	0.70971	0.70973	0.00015
1.21	0.70936	0.70977	0.00016
1.17	0.70968	0.70981	0.00013
1.12	0.70975	0.70990	0.00018
1.08	0.70946	0.70991	0.00018
1.04	0.70991	0.70999	0.00016
0.99	0.71010	0.71002	0.00017
0.95	0.70984	0.71001	0.00017
0.91	0.70957	0.71002	0.00017
0.86	0.70996	0.71008	0.00014
0.82	0.71003	0.71013	0.00015
0.78	0.70984	0.71013	0.00015
0.73	0.71051	0.71014	0.00014
0.69	0.70984	0.71011	0.00012
0.65	0.71025	0.71015	0.00011
0.61	0.71028	0.71014	0.00010
0.56	0.70995	0.71011	0.00010
0.52	0.70996	0.71012	0.00010
0.48	0.71022	0.71017	0.00010
0.43	0.71040	0.71017	0.00011
0.39	0.71010	0.71015	0.00010
0.35	0.70991	0.71015	0.00011
0.30	0.71016	0.71019	0.00009
0.26	0.71031	0.71019	0.00010
0.22	0.71013	0.71017	0.00011
0.17	0.70999	0.71018	0.00012
0.13	0.71005	0.71024	0.00011
0.09	0.71040	0.71034	0.00005
0.04	0.71028		

8.74	0.70926	0.70904	0.00011
8.69	0.70903	0.70905	0.00013
8.65	0.70909	0.70902	0.00015
8.61	0.70879	0.70902	0.00015
8.56	0.70878	0.70902	0.00014
8.52	0.70915	0.70908	0.00014
8.48	0.70892	0.70910	0.00016
8.43	0.70923	0.70906	0.00019
8.39	0.70920	0.70905	0.00020
8.35	0.70893	0.70901	0.00019
8.30	0.70941	0.70903	0.00019
8.26	0.70868	0.70897	0.00017
8.22	0.70908	0.70904	0.00017
8.17	0.70887	0.70902	0.00017
8.13	0.70929	0.70898	0.00020
8.09	0.70939	0.70894	0.00018
8.05	0.70856	0.70889	0.00015
8.00	0.70856	0.70894	0.00013
7.96	0.70891	0.70895	0.00012
7.92	0.70909	0.70893	0.00013
7.87	0.70885	0.70892	0.00013
7.83	0.70928	0.70895	0.00014
7.79	0.70890	0.70889	0.00012
7.74	0.70853	0.70892	0.00013
7.70	0.70899	0.70899	0.00011
7.66	0.70891	0.70899	0.00011
7.61	0.70901	0.70897	0.00013
7.57	0.70905	0.70897	0.00013
7.53	0.70867	0.70892	0.00015
7.48	0.70898	0.70897	0.00014
7.44	0.70915	0.70901	0.00016
7.40	0.70875	0.70900	0.00016
7.35	0.70916	0.70903	0.00015
7.31	0.70924	0.70905	0.00016
7.27	0.70898	0.70904	0.00015
7.22	0.70867	0.70905	0.00015
7.18	0.70902	0.70907	0.00014
7.14	0.70863	0.70913	0.00018
7.09	0.70911	0.70920	0.00015
7.05	0.70937	0.70916	0.00019
7.01	0.70905	0.70908	0.00021
6.96	0.70910	0.70909	0.00021
6.92	0.70934	0.70906	0.00021
6.88	0.70915	0.70902	0.00021
6.83	0.70903	0.70900	0.00021
6.79	0.70886	0.70903	0.00022
6.75	0.70969	0.70907	0.00022
6.70	0.70935	0.70900	0.00017
6.66	0.70862	0.70899	0.00016
6.62	0.70862	0.70904	0.00014

6.57	0.70911	0.70908	0.00011
6.53	0.70888	0.70908	0.00011
6.49	0.70889	0.70910	0.00010
6.44	0.70893	0.70907	0.00014
6.40	0.70936	0.70911	0.00015
6.36	0.70926	0.70906	0.00015
6.31	0.70896	0.70901	0.00015
6.27	0.70924	0.70899	0.00016
6.23	0.70915	0.70896	0.00015
6.19	0.70907	0.70895	0.00015
6.14	0.70910	0.70897	0.00016
6.10	0.70903	0.70894	0.00016
6.06	0.70855	0.70894	0.00016
6.01	0.70939	0.70896	0.00014
5.97	0.70887	0.70895	0.00011
5.93	0.70877	0.70891	0.00014
5.88	0.70874	0.70892	0.00014
5.84	0.70893	0.70897	0.00014
5.80	0.70903	0.70900	0.00015
5.75	0.70928	0.70895	0.00017
5.71	0.70882	0.70894	0.00016
5.67	0.70899	0.70895	0.00016
5.62	0.70883	0.70894	0.00016
5.58	0.70921	0.70897	0.00016
5.54	0.70852	0.70895	0.00016
5.49	0.70889	0.70902	0.00013
5.45	0.70920	0.70902	0.00013
5.41	0.70924	0.70898	0.00013
5.36	0.70858	0.70898	0.00013
5.32	0.70913	0.70904	0.00009
5.28	0.70894	0.70898	0.00013
5.23	0.70892	0.70901	0.00014
5.19	0.70912	0.70900	0.00014
5.15	0.70899	0.70902	0.00015
5.10	0.70920	0.70905	0.00016
5.06	0.70886	0.70907	0.00017
5.02	0.70886	0.70907	0.00017
4.97	0.70924	0.70905	0.00019
4.93	0.70913	0.70905	0.00019
4.89	0.70855	0.70905	0.00019
4.84	0.70926	0.70908	0.00016
4.80	0.70883	0.70910	0.00018
4.76	0.70931	0.70917	0.00019
4.71	0.70925	0.70914	0.00019
4.67	0.70940	0.70907	0.00023
4.63	0.70889	0.70904	0.00022
4.58	0.70865	0.70906	0.00021
4.54	0.70927	0.70909	0.00019
4.50	0.70909	0.70906	0.00019
4.46	0.70884	0.70903	0.00020

4.41	0.70949	0.70906	0.00019
4.37	0.70956	0.70898	0.00018
4.33	0.70900	0.70890	0.00013
4.28	0.70849	0.70892	0.00013
4.24	0.70909	0.70896	0.00010
4.20	0.70909	0.70895	0.00009
4.15	0.70899	0.70894	0.00009
4.11	0.70894	0.70896	0.00010
4.07	0.70884	0.70896	0.00010
4.02	0.70909	0.70895	0.00010
3.98	0.70869	0.70892	0.00010
3.94	0.70879	0.70896	0.00010
3.89	0.70916	0.70898	0.00009
3.85	0.70893	0.70899	0.00010
3.81	0.70901	0.70901	0.00010
3.76	0.70894	0.70896	0.00013
3.72	0.70915	0.70895	0.00014
3.68	0.70897	0.70890	0.00014
3.63	0.70878	0.70893	0.00015
3.59	0.70876	0.70892	0.00016
3.55	0.70915	0.70894	0.00015
3.50	0.70898	0.70895	0.00015
3.46	0.70921	0.70893	0.00016
3.42	0.70913	0.70892	0.00015
3.37	0.70855	0.70890	0.00014
3.33	0.70879	0.70896	0.00012
3.29	0.70869	0.70899	0.00012
3.24	0.70923	0.70898	0.00013
3.20	0.70871	0.70894	0.00013
3.16	0.70900	0.70900	0.00014
3.11	0.70918	0.70898	0.00015
3.07	0.70885	0.70895	0.00014
3.03	0.70906	0.70897	0.00014
2.98	0.70896	0.70894	0.00015
2.94	0.70912	0.70895	0.00015
2.90	0.70914	0.70895	0.00016
2.85	0.70860	0.70894	0.00015
2.81	0.70874	0.70898	0.00013
2.77	0.70938	0.70899	0.00012
2.72	0.70881	0.70898	0.00011
2.68	0.70885	0.70898	0.00011
2.64	0.70906	0.70897	0.00011
2.60	0.70871	0.70899	0.00012
2.55	0.70904	0.70901	0.00011
2.51	0.70919	0.70903	0.00012
2.47	0.70905	0.70898	0.00013
2.42	0.70890	0.70894	0.00015
2.38	0.70894	0.70896	0.00015
2.34	0.70926	0.70896	0.00015
2.29	0.70876	0.70890	0.00014

2.25	0.70880	0.70893	0.00014
2.21	0.70921	0.70895	0.00014
2.16	0.70892	0.70892	0.00013
2.12	0.70924	0.70895	0.00014
2.08	0.70874	0.70896	0.00015
2.03	0.70860	0.70901	0.00015
1.99	0.70914	0.70905	0.00012
1.95	0.70894	0.70899	0.00016
1.90	0.70867	0.70902	0.00017
1.86	0.70899	0.70903	0.00016
1.82	0.70908	0.70904	0.00016
1.77	0.70890	0.70904	0.00016
1.73	0.70916	0.70904	0.00015
1.69	0.70933	0.70904	0.00015
1.64	0.70924	0.70899	0.00014
1.60	0.70908	0.70898	0.00014
1.56	0.70849	0.70901	0.00015
1.51	0.70924	0.70906	0.00010
1.47	0.70883	0.70903	0.00010
1.43	0.70906	0.70910	0.00012
1.38	0.70905	0.70911	0.00012
1.34	0.70893	0.70908	0.00014
1.30	0.70909	0.70905	0.00015
1.25	0.70886	0.70907	0.00016
1.21	0.70919	0.70909	0.00015
1.17	0.70932	0.70906	0.00015
1.12	0.70907	0.70901	0.00015
1.08	0.70893	0.70898	0.00015
1.04	0.70947	0.70901	0.00016
0.99	0.70915	0.70899	0.00014
0.95	0.70874	0.70894	0.00015
0.91	0.70872	0.70904	0.00020
0.87	0.70925	0.70904	0.00020
0.82	0.70901	0.70903	0.00020
0.78	0.70891	0.70907	0.00022
0.74	0.70882	0.70917	0.00026
0.69	0.70882	0.70924	0.00026
0.65	0.70919	0.70924	0.00026
0.61	0.70932	0.70922	0.00027
0.56	0.70865	0.70915	0.00029
0.52	0.70969	0.70918	0.00028
0.48	0.70875	0.70913	0.00025
0.43	0.70908	0.70915	0.00024
0.39	0.70950	0.70915	0.00026
0.35	0.70985	0.70911	0.00026
0.30	0.70959	0.70901	0.00019
0.26	0.70881	0.70891	0.00011
0.22	0.70894	0.70893	0.00012
0.17	0.70866	0.70893	0.00014
0.13	0.70891	0.70902	0.00010

0.09	0.70920	0.70907	0.00012
0.04	0.70893		

ARB 16.3.1 (M1)

Distance from cervix (mm)	$^{87}Sr/^{86}Sr$	10 point mov. average	2 SE on mov. average
32.30	0.71070	0.71069	0.00008
32.26	0.71075	0.71073	0.00013
32.21	0.71065	0.71071	0.00013
32.17	0.71079	0.71068	0.00016
32.13	0.71075	0.71062	0.00018
32.08	0.71051	0.71061	0.00018
32.04	0.71078	0.71059	0.00019
32.00	0.71049	0.71059	0.00019
31.95	0.71089	0.71064	0.00020
31.91	0.71057	0.71058	0.00020
31.87	0.71117	0.71056	0.00021
31.82	0.71056	0.71053	0.00017
31.78	0.71028	0.71054	0.00018
31.74	0.71021	0.71056	0.00017
31.69	0.71064	0.71060	0.00015
31.65	0.71032	0.71058	0.00015
31.61	0.71078	0.71065	0.00016
31.56	0.71097	0.71064	0.00016
31.52	0.71026	0.71062	0.00015
31.48	0.71042	0.71066	0.00012
31.43	0.71088	0.71071	0.00013
31.39	0.71067	0.71069	0.00012
31.35	0.71050	0.71071	0.00013
31.30	0.71058	0.71075	0.00012
31.26	0.71039	0.71076	0.00011
31.22	0.71100	0.71081	0.00009
31.17	0.71070	0.71078	0.00008
31.13	0.71078	0.71078	0.00008
31.09	0.71066	0.71076	0.00009
31.04	0.71099	0.71079	0.00009
31.00	0.71067	0.71077	0.00007
30.96	0.71087	0.71082	0.00011
30.91	0.71082	0.71083	0.00011
30.87	0.71070	0.71082	0.00012
30.83	0.71095	0.71083	0.00011
30.78	0.71063	0.71084	0.00012
30.74	0.71075	0.71086	0.00011
30.70	0.71060	0.71088	0.00010
30.65	0.71090	0.71088	0.00011
30.61	0.71078	0.71085	0.00012
30.57	0.71121	0.71082	0.00013
30.48	0.71094	0.71076	0.00011
30.44	0.71072	0.71073	0.00010
30.39	0.71085	0.71073	0.00010
30.35	0.71102	0.71073	0.00010
30.31	0.71089	0.71070	0.00008
30.27	0.71088	0.71072	0.00012

ARB 16.2.1 (M2)

Distance from cervix (mm)	$^{87}Sr/^{86}Sr$	10 point mov. average	2 SE on mov. average
41.94	0.71037	0.71036	0.00023
41.90	0.71015	0.71035	0.00023
41.85	0.71035	0.71039	0.00022
41.81	0.71074	0.71035	0.00024
41.77	0.70962	0.71036	0.00025
41.72	0.71024	0.71043	0.00019
41.68	0.71023	0.71042	0.00020
41.64	0.71084	0.71043	0.00020
41.59	0.71075	0.71035	0.00018
41.55	0.71029	0.71043	0.00030
41.51	0.71032	0.71048	0.00030
41.46	0.71051	0.71050	0.00030
41.42	0.70992	0.71046	0.00031
41.38	0.71087	0.71051	0.00028
41.33	0.71034	0.71043	0.00028
41.29	0.71013	0.71046	0.00028
41.24	0.71032	0.71049	0.00027
41.20	0.71008	0.71052	0.00027
41.16	0.71155	0.71053	0.00027
41.11	0.71072	0.71045	0.00015
41.07	0.71055	0.71037	0.00017
41.03	0.71013	0.71034	0.00017
40.98	0.71041	0.71042	0.00019
40.94	0.71011	0.71038	0.00020
40.90	0.71059	0.71045	0.00021
40.85	0.71041	0.71045	0.00021
40.81	0.71065	0.71044	0.00021
40.77	0.71016	0.71047	0.00023
40.72	0.71075	0.71046	0.00023
40.68	0.70995	0.71041	0.00023
40.64	0.71029	0.71048	0.00021
40.59	0.71087	0.71051	0.00021
40.55	0.71003	0.71051	0.00021
40.51	0.71082	0.71063	0.00022
40.46	0.71055	0.71057	0.00022
40.42	0.71035	0.71056	0.00023
40.38	0.71093	0.71057	0.00022
40.33	0.71009	0.71053	0.00021
40.29	0.71023	0.71060	0.00019
40.25	0.71067	0.71061	0.00018
40.20	0.71056	0.71058	0.00019
40.16	0.71090	0.71063	0.00021
40.11	0.71119	0.71062	0.00021
40.07	0.71027	0.71062	0.00021
40.03	0.71043	0.71062	0.00020
39.98	0.71047	0.71067	0.00020
39.94	0.71046	0.71071	0.00020

30.22	0.71058	0.71068	0.00012	39.90	0.71085	0.71076	0.00020
30.18	0.71060	0.71068	0.00012	39.85	0.71032	0.71081	0.00024
30.14	0.71053	0.71066	0.00013	39.81	0.71032	0.71088	0.00022
30.09	0.71061	0.71068	0.00013	39.77	0.71104	0.71101	0.00023
30.05	0.71066	0.71068	0.00013	39.72	0.71084	0.71105	0.00024
30.01	0.71070	0.71069	0.00013	39.68	0.71117	0.71111	0.00025
29.96	0.71087	0.71066	0.00014	39.64	0.71031	0.71117	0.00029
29.92	0.71063	0.71062	0.00013	39.59	0.71086	0.71133	0.00025
29.88	0.71114	0.71065	0.00014	39.55	0.71089	0.71140	0.00023
29.83	0.71048	0.71058	0.00009	39.51	0.71095	0.71150	0.00022
29.79	0.71054	0.71053	0.00015	39.46	0.71141	0.71162	0.00021
29.75	0.71041	0.71057	0.00017	39.42	0.71097	0.71169	0.00023
29.70	0.71078	0.71059	0.00016	39.38	0.71167	0.71176	0.00016
29.66	0.71061	0.71055	0.00016	39.33	0.71145	0.71180	0.00017
29.62	0.71069	0.71055	0.00016	39.29	0.71138	0.71183	0.00015
29.57	0.71047	0.71050	0.00017	39.25	0.71184	0.71186	0.00011
29.53	0.71048	0.71048	0.00018	39.20	0.71186	0.71188	0.00012
29.49	0.71084	0.71052	0.00020	39.16	0.71159	0.71192	0.00014
29.44	0.71052	0.71049	0.00018	39.12	0.71192	0.71199	0.00015
29.40	0.70999	0.71049	0.00018	39.07	0.71206	0.71203	0.00015
29.36	0.71091	0.71055	0.00015	39.03	0.71218	0.71201	0.00016
29.31	0.71056	0.71052	0.00012	38.98	0.71170	0.71200	0.00016
29.27	0.71046	0.71047	0.00015	38.94	0.71199	0.71200	0.00015
29.23	0.71059	0.71044	0.00016	38.90	0.71179	0.71203	0.00016
29.18	0.71016	0.71043	0.00016	38.85	0.71168	0.71205	0.00016
29.14	0.71027	0.71048	0.00016	38.81	0.71200	0.71212	0.00015
29.10	0.71086	0.71051	0.00015	38.77	0.71225	0.71209	0.00017
29.05	0.71053	0.71047	0.00013	38.72	0.71238	0.71213	0.00020
29.01	0.71060	0.71038	0.00020	38.68	0.71227	0.71207	0.00021
28.97	0.71057	0.71039	0.00021	38.64	0.71183	0.71204	0.00020
28.92	0.71058	0.71031	0.00023	38.59	0.71207	0.71210	0.00021
28.88	0.71010	0.71033	0.00024	38.55	0.71175	0.71211	0.00021
28.84	0.71011	0.71036	0.00024	38.51	0.71232	0.71219	0.00021
28.79	0.71053	0.71033	0.00025	38.46	0.71196	0.71219	0.00021
28.75	0.71065	0.71031	0.00025	38.42	0.71241	0.71221	0.00020
28.71	0.71056	0.71025	0.00024	38.38	0.71166	0.71218	0.00020
28.66	0.71041	0.71019	0.00024	38.33	0.71264	0.71219	0.00019
28.62	0.70966	0.71015	0.00024	38.29	0.71177	0.71217	0.00017
28.58	0.71070	0.71022	0.00021	38.25	0.71200	0.71213	0.00024
28.53	0.70982	0.71015	0.00019	38.20	0.71241	0.71210	0.00025
28.49	0.71073	0.71018	0.00017	38.16	0.71219	0.71203	0.00025
28.45	0.71039	0.71012	0.00012	38.12	0.71251	0.71195	0.00027
28.40	0.70984	0.71012	0.00012	38.07	0.71236	0.71187	0.00025
28.36	0.71030	0.71019	0.00013	38.03	0.71219	0.71174	0.00027
28.32	0.71010	0.71016	0.00013	37.99	0.71212	0.71169	0.00025
28.27	0.70989	0.71019	0.00014	37.94	0.71174	0.71169	0.00025
28.23	0.71007	0.71019	0.00014	37.90	0.71245	0.71162	0.00028
28.19	0.71038	0.71019	0.00014	37.85	0.71129	0.71150	0.00022
28.14	0.70995	0.71016	0.00014	37.81	0.71173	0.71153	0.00022
28.10	0.71011	0.71015	0.00015	37.77	0.71169	0.71150	0.00022

28.06	0.71021	0.71017	0.00015
28.01	0.71034	0.71017	0.00015
27.97	0.71056	0.71020	0.00017
27.93	0.70999	0.71017	0.00015
27.88	0.71040	0.71020	0.00015
27.84	0.70990	0.71015	0.00014
27.80	0.71002	0.71019	0.00013
27.75	0.71010	0.71021	0.00013
27.71	0.70985	0.71016	0.00017
27.67	0.71029	0.71019	0.00016
27.62	0.71027	0.71013	0.00019
27.58	0.71058	0.71014	0.00019
27.54	0.71032	0.71010	0.00017
27.49	0.71023	0.71009	0.00016
27.45	0.70998	0.71011	0.00017
27.41	0.71028	0.71016	0.00019
27.36	0.71015	0.71021	0.00023
27.32	0.70962	0.71021	0.00023
27.28	0.71017	0.71026	0.00019
27.23	0.70965	0.71029	0.00019
27.19	0.71038	0.71038	0.00014
27.15	0.71026	0.71042	0.00016
27.10	0.71018	0.71042	0.00015
27.06	0.71039	0.71039	0.00019
27.02	0.71054	0.71037	0.00019
26.97	0.71079	0.71031	0.00020
26.93	0.71015	0.71023	0.00018
26.89	0.71009	0.71018	0.00021
26.84	0.71047	0.71020	0.00021
26.80	0.71058	0.71023	0.00024
26.76	0.71073	0.71018	0.00023
26.71	0.71027	0.71011	0.00020
26.67	0.70985	0.71012	0.00020
26.63	0.71021	0.71013	0.00020
26.58	0.71000	0.71013	0.00020
26.54	0.71000	0.71017	0.00021
26.50	0.70963	0.71019	0.00020
26.45	0.71025	0.71026	0.00016
26.41	0.71082	0.71026	0.00016
26.37	0.71003	0.71018	0.00012
26.32	0.71005	0.71023	0.00013
26.28	0.71039	0.71027	0.00013
26.24	0.70989	0.71028	0.00014
26.20	0.71019	0.71034	0.00011
26.15	0.71041	0.71033	0.00012
26.11	0.71028	0.71032	0.00012
26.07	0.71024	0.71027	0.00016
26.02	0.71033	0.71025	0.00017
25.98	0.70996	0.71026	0.00017
25.94	0.71055	0.71029	0.00015

37.72	0.71140	0.71149	0.00021
37.68	0.71171	0.71149	0.00021
37.64	0.71106	0.71141	0.00023
37.59	0.71167	0.71144	0.00022
37.55	0.71214	0.71143	0.00021
37.51	0.71108	0.71133	0.00014
37.46	0.71117	0.71143	0.00019
37.42	0.71166	0.71142	0.00020
37.38	0.71138	0.71142	0.00020
37.33	0.71163	0.71135	0.00024
37.29	0.71138	0.71127	0.00025
37.25	0.71096	0.71122	0.00026
37.20	0.71136	0.71122	0.00026
37.16	0.71148	0.71123	0.00026
37.12	0.71121	0.71117	0.00026
37.07	0.71203	0.71118	0.00026
37.03	0.71107	0.71110	0.00018
36.99	0.71166	0.71107	0.00020
36.94	0.71072	0.71100	0.00015
36.90	0.71084	0.71101	0.00014
36.86	0.71084	0.71094	0.00021
36.81	0.71102	0.71093	0.00022
36.77	0.71138	0.71089	0.00023
36.72	0.71096	0.71077	0.00023
36.68	0.71125	0.71077	0.00023
36.64	0.71126	0.71070	0.00021
36.59	0.71073	0.71065	0.00017
36.55	0.71104	0.71057	0.00022
36.51	0.71075	0.71051	0.00020
36.46	0.71020	0.71045	0.00020
36.42	0.71074	0.71053	0.00023
36.38	0.71055	0.71055	0.00023
36.33	0.71024	0.71055	0.00023
36.29	0.71091	0.71062	0.00023
36.25	0.71060	0.71061	0.00023
36.20	0.71077	0.71058	0.00023
36.16	0.70990	0.71053	0.00023
36.12	0.71047	0.71061	0.00019
36.07	0.71013	0.71061	0.00019
36.03	0.71104	0.71063	0.00016
35.99	0.71087	0.71061	0.00015
35.94	0.71061	0.71061	0.00015
35.90	0.71091	0.71061	0.00015
35.86	0.71076	0.71055	0.00014
35.81	0.71038	0.71055	0.00014
35.77	0.71027	0.71053	0.00015
35.73	0.71063	0.71057	0.00014
35.68	0.71048	0.71058	0.00015
35.64	0.71037	0.71061	0.00015
35.59	0.71088	0.71060	0.00016

25.89	0.71041	0.71030	0.00017	35.55	0.71083	0.71055	0.00015
25.85	0.71057	0.71031	0.00017	35.51	0.71056	0.71061	0.00024
25.81	0.71043	0.71028	0.00016	35.46	0.71030	0.71062	0.00024
25.76	0.71013	0.71028	0.00016	35.42	0.71077	0.71069	0.00024
25.72	0.71031	0.71031	0.00016	35.38	0.71022	0.71069	0.00024
25.68	0.70980	0.71033	0.00017	35.33	0.71066	0.71073	0.00021
25.63	0.71002	0.71034	0.00015	35.29	0.71070	0.71077	0.00022
25.59	0.71042	0.71038	0.00013	35.25	0.71081	0.71077	0.00022
25.55	0.71025	0.71031	0.00018	35.20	0.71023	0.71079	0.00023
25.50	0.71068	0.71031	0.00018	35.16	0.71036	0.71088	0.00019
25.46	0.71053	0.71023	0.00018	35.12	0.71146	0.71094	0.00016
25.42	0.71020	0.71022	0.00017	35.07	0.71067	0.71087	0.00011
25.37	0.71048	0.71026	0.00019	35.03	0.71102	0.71089	0.00010
25.33	0.71038	0.71030	0.00022	34.99	0.71077	0.71088	0.00009
25.29	0.71050	0.71027	0.00022	34.94	0.71065	0.71085	0.00012
25.24	0.70993	0.71027	0.00022	34.90	0.71105	0.71088	0.00011
25.20	0.71043	0.71030	0.00021	34.86	0.71071	0.71084	0.00012
25.16	0.70975	0.71030	0.00021	34.81	0.71097	0.71086	0.00012
25.11	0.71021	0.71032	0.00019	34.77	0.71110	0.71075	0.00022
25.07	0.70991	0.71031	0.00019	34.73	0.71095	0.71075	0.00022
25.03	0.71042	0.71032	0.00018	34.68	0.71084	0.71076	0.00023
24.98	0.71059	0.71027	0.00020	34.64	0.71080	0.71075	0.00023
24.94	0.71086	0.71023	0.00019	34.60	0.71094	0.71079	0.00024
24.90	0.71006	0.71023	0.00019	34.55	0.71051	0.71077	0.00024
24.85	0.71051	0.71025	0.00019	34.51	0.71097	0.71083	0.00024
24.81	0.71023	0.71027	0.00020	34.46	0.71058	0.71087	0.00026
24.77	0.71042	0.71024	0.00021	34.42	0.71092	0.71091	0.00025
24.72	0.70996	0.71027	0.00023	34.38	0.70989	0.71089	0.00025
24.68	0.71016	0.71032	0.00022	34.33	0.71109	0.71101	0.00012
24.64	0.71003	0.71037	0.00022	34.29	0.71106	0.71097	0.00013
24.59	0.70984	0.71040	0.00021	34.25	0.71077	0.71095	0.00013
24.55	0.71022	0.71048	0.00018	34.20	0.71117	0.71101	0.00014
24.51	0.71086	0.71051	0.00017	34.16	0.71075	0.71097	0.00014
24.46	0.71031	0.71047	0.00015	34.12	0.71109	0.71098	0.00014
24.42	0.71064	0.71044	0.00017	34.07	0.71135	0.71100	0.00015
24.38	0.70997	0.71039	0.00017	34.03	0.71096	0.71097	0.00013
24.33	0.71073	0.71043	0.00015	33.99	0.71080	0.71101	0.00015
24.29	0.71045	0.71038	0.00014	33.94	0.71103	0.71102	0.00014
24.25	0.71069	0.71037	0.00014	33.90	0.71073	0.71104	0.00015
24.20	0.71028	0.71032	0.00012	33.86	0.71088	0.71109	0.00014
24.16	0.71066	0.71030	0.00013	33.81	0.71133	0.71117	0.00017
24.12	0.71045	0.71030	0.00012	33.77	0.71078	0.71113	0.00017
24.07	0.71052	0.71027	0.00012	33.73	0.71087	0.71117	0.00015
24.03	0.70999	0.71028	0.00012	33.68	0.71131	0.71122	0.00013
23.99	0.71019	0.71033	0.00011	33.64	0.71104	0.71119	0.00014
23.94	0.71029	0.71036	0.00012	33.60	0.71129	0.71113	0.00020
23.90	0.71028	0.71035	0.00013	33.55	0.71089	0.71109	0.00020
23.86	0.71036	0.71034	0.00013	33.51	0.71128	0.71108	0.00020
23.81	0.71014	0.71034	0.00013	33.47	0.71125	0.71098	0.00025
23.77	0.71014	0.71033	0.00013	33.42	0.71163	0.71086	0.00031

23.73	0.71061	0.71032	0.00015	33.38	0.71099	0.71078	0.00026
23.68	0.71018	0.71036	0.00019	33.33	0.71117	0.71074	0.00025
23.64	0.71059	0.71038	0.00019	33.29	0.71131	0.71068	0.00024
23.60	0.71051	0.71041	0.00021	33.25	0.71101	0.71054	0.00023
23.55	0.71053	0.71038	0.00022	33.20	0.71047	0.71050	0.00021
23.51	0.71013	0.71039	0.00022	33.16	0.71092	0.71057	0.00024
23.47	0.71025	0.71046	0.00023	33.12	0.71076	0.71056	0.00023
23.42	0.71036	0.71047	0.00022	33.07	0.71033	0.71053	0.00023
23.38	0.71005	0.71045	0.00023	33.03	0.70999	0.71053	0.00023
23.34	0.71000	0.71048	0.00022	32.99	0.71084	0.71059	0.00019
23.29	0.71101	0.71061	0.00025	32.94	0.71061	0.71057	0.00018
23.25	0.71034	0.71057	0.00024	32.90	0.71055	0.71057	0.00018
23.21	0.71090	0.71058	0.00023	32.86	0.70996	0.71062	0.00021
23.16	0.71025	0.71054	0.00022	32.81	0.71060	0.71067	0.00015
23.12	0.71058	0.71055	0.00022	32.77	0.71111	0.71064	0.00018
23.08	0.71085	0.71052	0.00022	32.73	0.71081	0.71057	0.00015
23.03	0.71034	0.71052	0.00022	32.68	0.71049	0.71058	0.00015
22.99	0.71017	0.71047	0.00026	32.64	0.71039	0.71056	0.00016
22.95	0.71033	0.71045	0.00027	32.60	0.71057	0.71056	0.00016
22.90	0.71136	0.71048	0.00027	32.55	0.71061	0.71059	0.00017
22.86	0.71055	0.71040	0.00019	32.51	0.71056	0.71062	0.00018
22.82	0.71051	0.71038	0.00019	32.47	0.71106	0.71059	0.00020
22.77	0.71041	0.71032	0.00020	32.42	0.71053	0.71054	0.00017
22.73	0.71039	0.71032	0.00020	32.38	0.71022	0.71048	0.00021
22.69	0.71025	0.71033	0.00020	32.34	0.71047	0.71052	0.00020
22.64	0.71085	0.71036	0.00021	32.29	0.71089	0.71051	0.00021
22.60	0.70986	0.71030	0.00017	32.25	0.71028	0.71044	0.00020
22.56	0.70998	0.71033	0.00015	32.20	0.71045	0.71044	0.00020
22.51	0.71067	0.71040	0.00014	32.16	0.71084	0.71045	0.00020
22.47	0.71051	0.71034	0.00014	32.12	0.71093	0.71041	0.00018
22.43	0.71032	0.71034	0.00014	32.07	0.71018	0.71040	0.00017
22.38	0.70999	0.71039	0.00016	32.03	0.71058	0.71042	0.00017
22.34	0.71035	0.71046	0.00015	31.99	0.70995	0.71050	0.00024
22.30	0.71054	0.71045	0.00015	31.94	0.71067	0.71054	0.00021
22.25	0.71051	0.71045	0.00015	31.90	0.71035	0.71056	0.00022
22.21	0.71030	0.71044	0.00015	31.86	0.71018	0.71063	0.00023
22.17	0.71015	0.71044	0.00015	31.81	0.71028	0.71072	0.00023
22.13	0.71063	0.71043	0.00017	31.77	0.71053	0.71074	0.00022
22.08	0.71007	0.71041	0.00016	31.73	0.71044	0.71075	0.00022
22.04	0.71054	0.71040	0.00017	31.68	0.71089	0.71079	0.00021
22.00	0.71077	0.71035	0.00018	31.64	0.71038	0.71073	0.00022
21.95	0.71069	0.71032	0.00015	31.60	0.71132	0.71072	0.00024
21.91	0.71032	0.71027	0.00013	31.55	0.71039	0.71060	0.00022
21.87	0.71047	0.71027	0.00013	31.51	0.71083	0.71060	0.00021
21.82	0.71034	0.71023	0.00012	31.47	0.71104	0.71054	0.00022
21.78	0.71001	0.71028	0.00018	31.42	0.71116	0.71045	0.00021
21.74	0.71045	0.71029	0.00018	31.38	0.71045	0.71036	0.00014
21.69	0.70998	0.71032	0.00019	31.34	0.71064	0.71034	0.00014
21.65	0.71013	0.71038	0.00018	31.29	0.71078	0.71035	0.00015
21.61	0.71053	0.71041	0.00017	31.25	0.71035	0.71032	0.00012

21.56	0.71019	0.71041	0.00017
21.52	0.71030	0.71045	0.00017
21.48	0.71033	0.71050	0.00018
21.43	0.71002	0.71049	0.00019
21.39	0.71090	0.71055	0.00016
21.35	0.71011	0.71049	0.00014
21.30	0.71071	0.71052	0.00012
21.26	0.71054	0.71054	0.00015
21.22	0.71047	0.71050	0.00016
21.17	0.71053	0.71046	0.00019
21.13	0.71064	0.71045	0.00019
21.09	0.71080	0.71046	0.00019
21.04	0.71019	0.71037	0.00020
21.00	0.71061	0.71040	0.00019
20.96	0.71029	0.71039	0.00019
20.91	0.71038	0.71039	0.00019
20.87	0.71096	0.71038	0.00019
20.83	0.71018	0.71036	0.00016
20.78	0.71005	0.71040	0.00016
20.74	0.71044	0.71048	0.00017
20.70	0.71066	0.71044	0.00019
20.65	0.70995	0.71045	0.00020
20.61	0.71050	0.71050	0.00017
20.57	0.71052	0.71051	0.00017
20.52	0.71029	0.71047	0.00019
20.48	0.71028	0.71043	0.00022
20.44	0.71069	0.71046	0.00022
20.39	0.71058	0.71044	0.00021
20.35	0.71091	0.71046	0.00022
20.31	0.71001	0.71044	0.00021
20.26	0.71082	0.71054	0.00021
20.22	0.71038	0.71050	0.00020
20.18	0.71061	0.71053	0.00020
20.13	0.71009	0.71048	0.00022
20.09	0.70994	0.71054	0.00021
20.05	0.71054	0.71054	0.00020
20.00	0.71055	0.71052	0.00021
19.96	0.71076	0.71056	0.00023
19.92	0.71074	0.71058	0.00024
19.87	0.71098	0.71057	0.00023
19.83	0.71042	0.71056	0.00023
19.79	0.71069	0.71062	0.00024
19.74	0.71006	0.71058	0.00025
19.70	0.71073	0.71065	0.00023
19.66	0.70994	0.71067	0.00023
19.61	0.71033	0.71072	0.00018
19.57	0.71100	0.71073	0.00017
19.53	0.71090	0.71068	0.00016
19.48	0.71065	0.71067	0.00016
19.44	0.71092	0.71065	0.00016

31.21	0.71022	0.71036	0.00015
31.16	0.71018	0.71035	0.00015
31.12	0.71042	0.71038	0.00015
31.07	0.71023	0.71035	0.00017
31.03	0.71006	0.71038	0.00017
30.99	0.71027	0.71041	0.00016
30.94	0.71025	0.71045	0.00016
30.90	0.71076	0.71053	0.00020
30.86	0.71049	0.71050	0.00019
30.81	0.71071	0.71051	0.00019
30.77	0.71010	0.71048	0.00019
30.73	0.71056	0.71043	0.00024
30.68	0.71003	0.71048	0.00027
30.64	0.71058	0.71050	0.00026
30.60	0.71033	0.71042	0.00029
30.55	0.71068	0.71047	0.00031
30.51	0.71107	0.71044	0.00030
30.47	0.71043	0.71037	0.00027
30.42	0.71061	0.71045	0.00032
30.38	0.71040	0.71046	0.00032
30.34	0.70966	0.71050	0.00033
30.29	0.71104	0.71061	0.00027
30.25	0.71018	0.71058	0.00026
30.21	0.70979	0.71061	0.00024
30.16	0.71088	0.71073	0.00017
30.12	0.71036	0.71069	0.00017
30.08	0.71038	0.71070	0.00016
30.03	0.71124	0.71072	0.00015
29.99	0.71063	0.71068	0.00010
29.94	0.71085	0.71068	0.00010
29.90	0.71072	0.71063	0.00011
29.86	0.71073	0.71057	0.00015
29.81	0.71052	0.71057	0.00015
29.77	0.71094	0.71057	0.00015
29.73	0.71053	0.71051	0.00012
29.68	0.71044	0.71051	0.00012
29.64	0.71061	0.71046	0.00017
29.60	0.71083	0.71051	0.00021
29.55	0.71059	0.71051	0.00022
29.51	0.71042	0.71053	0.00022
29.47	0.71011	0.71059	0.00024
29.42	0.71068	0.71066	0.00022
29.38	0.71051	0.71063	0.00022
29.34	0.71039	0.71062	0.00023
29.29	0.71052	0.71059	0.00025
29.25	0.70992	0.71064	0.00026
29.21	0.71109	0.71076	0.00022
29.16	0.71091	0.71073	0.00020
29.12	0.71076	0.71073	0.00020
29.08	0.71105	0.71073	0.00020

19.40	0.71101	0.71062	0.00015	29.03	0.71072	0.71071	0.00020
19.35	0.71024	0.71063	0.00016	28.99	0.71040	0.71073	0.00020
19.31	0.71083	0.71062	0.00017	28.95	0.71045	0.71080	0.00020
19.27	0.71085	0.71058	0.00017	28.90	0.71009	0.71086	0.00019
19.22	0.71048	0.71054	0.00016	28.86	0.71104	0.71093	0.00008
19.18	0.71046	0.71061	0.00019	28.81	0.71108	0.71091	0.00008
19.14	0.71047	0.71062	0.00019	28.77	0.71079	0.71091	0.00008
19.09	0.71075	0.71062	0.00019	28.73	0.71091	0.71091	0.00008
19.05	0.71049	0.71061	0.00019	28.68	0.71080	0.71088	0.00009
19.01	0.71061	0.71062	0.00019	28.64	0.71087	0.71086	0.00012
18.96	0.71112	0.71067	0.00021	28.60	0.71083	0.71085	0.00012
18.92	0.71015	0.71064	0.00019	28.55	0.71117	0.71088	0.00013
18.88	0.71043	0.71073	0.00017	28.51	0.71100	0.71091	0.00017
18.83	0.71048	0.71079	0.00017	28.47	0.71080	0.71092	0.00017
18.79	0.71110	0.71078	0.00017	28.42	0.71088	0.71090	0.00018
18.75	0.71059	0.71071	0.00017	28.38	0.71102	0.71091	0.00018
18.70	0.71051	0.71069	0.00018	28.34	0.71080	0.71092	0.00019
18.66	0.71063	0.71070	0.00018	28.29	0.71067	0.71093	0.00019
18.62	0.71055	0.71069	0.00018	28.25	0.71052	0.71096	0.00018
18.57	0.71113	0.71069	0.00018	28.21	0.71075	0.71104	0.00017
18.53	0.71081	0.71067	0.00016	28.16	0.71117	0.71103	0.00018
18.49	0.71108	0.71067	0.00016	28.12	0.71147	0.71102	0.00018
18.44	0.71097	0.71061	0.00014	28.08	0.71106	0.71098	0.00015
18.40	0.71046	0.71059	0.00012	28.03	0.71063	0.71089	0.00022
18.36	0.71035	0.71060	0.00012	27.99	0.71100	0.71092	0.00021
18.31	0.71042	0.71063	0.00010	27.95	0.71116	0.71089	0.00022
18.27	0.71058	0.71062	0.00011	27.90	0.71086	0.71078	0.00026
18.23	0.71058	0.71060	0.00012	27.86	0.71093	0.71082	0.00027
18.18	0.71054	0.71058	0.00013	27.82	0.71137	0.71080	0.00027
18.14	0.71093	0.71062	0.00016	27.77	0.71062	0.71083	0.00030
18.10	0.71081	0.71068	0.00023	27.73	0.71111	0.71091	0.00032
18.06	0.71048	0.71068	0.00023	27.68	0.71107	0.71084	0.00033
18.01	0.71078	0.71073	0.00024	27.64	0.71015	0.71079	0.00033
17.97	0.71053	0.71077	0.00026	27.60	0.71097	0.71079	0.00032
17.93	0.71068	0.71079	0.00025	27.55	0.71065	0.71074	0.00033
17.88	0.71030	0.71081	0.00025	27.51	0.71010	0.71081	0.00035
17.84	0.71037	0.71087	0.00022	27.47	0.71121	0.71088	0.00032
17.80	0.71035	0.71092	0.00019	27.42	0.71076	0.71082	0.00031
17.75	0.71098	0.71099	0.00015	27.38	0.71168	0.71075	0.00035
17.71	0.71152	0.71101	0.00015	27.34	0.71136	0.71062	0.00029
17.67	0.71077	0.71091	0.00012	27.29	0.71046	0.71057	0.00024
17.62	0.71097	0.71093	0.00012	27.25	0.71051	0.71058	0.00024
17.58	0.71122	0.71090	0.00012	27.21	0.71021	0.71057	0.00024
17.54	0.71077	0.71090	0.00012	27.16	0.71042	0.71059	0.00023
17.49	0.71087	0.71094	0.00012	27.12	0.71142	0.71060	0.00023
17.45	0.71085	0.71090	0.00015	27.08	0.71077	0.71057	0.00018
17.41	0.71092	0.71095	0.00017	27.03	0.71066	0.71055	0.00018
17.36	0.71104	0.71099	0.00018	26.99	0.71002	0.71050	0.00019
17.32	0.71114	0.71095	0.00020	26.95	0.71041	0.71054	0.00016
17.28	0.71058	0.71089	0.00021	26.90	0.71080	0.71051	0.00018

17.23	0.71092	0.71091	0.00020
17.19	0.71071	0.71089	0.00020
17.15	0.71119	0.71090	0.00020
17.10	0.71113	0.71088	0.00019
17.06	0.71054	0.71086	0.00018
17.02	0.71136	0.71086	0.00019
16.97	0.71129	0.71079	0.00015
16.93	0.71065	0.71073	0.00011
16.89	0.71051	0.71074	0.00011
16.84	0.71075	0.71070	0.00017
16.80	0.71074	0.71067	0.00017
16.76	0.71085	0.71070	0.00020
16.71	0.71098	0.71069	0.00019
16.67	0.71097	0.71066	0.00018
16.63	0.71046	0.71063	0.00017
16.58	0.71074	0.71066	0.00016
16.54	0.71065	0.71064	0.00016
16.50	0.71078	0.71070	0.00021
16.45	0.71008	0.71073	0.00022
16.41	0.71043	0.71077	0.00018
16.37	0.71110	0.71080	0.00017
16.32	0.71074	0.71075	0.00015
16.28	0.71068	0.71077	0.00016
16.24	0.71068	0.71080	0.00016
16.19	0.71070	0.71083	0.00016
16.15	0.71056	0.71087	0.00017
16.11	0.71130	0.71089	0.00015
16.06	0.71104	0.71089	0.00015
16.02	0.71046	0.71084	0.00016
15.98	0.71075	0.71085	0.00015
15.93	0.71062	0.71089	0.00017
15.89	0.71096	0.71093	0.00016
15.85	0.71099	0.71093	0.00016
15.80	0.71097	0.71091	0.00016
15.76	0.71106	0.71087	0.00017
15.72	0.71079	0.71083	0.00017
15.67	0.71127	0.71084	0.00017
15.63	0.71051	0.71082	0.00015
15.59	0.71056	0.71080	0.00016
15.54	0.71122	0.71082	0.00015
15.50	0.71098	0.71078	0.00013
15.46	0.71094	0.71077	0.00012
15.41	0.71082	0.71073	0.00013
15.37	0.71059	0.71073	0.00013
15.33	0.71065	0.71075	0.00013
15.28	0.71089	0.71076	0.00012
15.24	0.71100	0.71071	0.00013
15.20	0.71036	0.71068	0.00012
15.15	0.71072	0.71069	0.00012
15.11	0.71086	0.71070	0.00012
26.86	0.71056	0.71048	0.00017
26.82	0.71041	0.71053	0.00021
26.77	0.71041	0.71053	0.00021
26.73	0.71057	0.71052	0.00021
26.69	0.71111	0.71053	0.00021
26.64	0.71052	0.71049	0.00018
26.60	0.71015	0.71055	0.00022
26.55	0.71047	0.71060	0.00020
26.51	0.71013	0.71066	0.00022
26.47	0.71048	0.71066	0.00023
26.42	0.71108	0.71070	0.00023
26.38	0.71037	0.71070	0.00023
26.34	0.71032	0.71074	0.00022
26.29	0.71068	0.71074	0.00021
26.25	0.71068	0.71072	0.00022
26.21	0.71111	0.71068	0.00024
26.16	0.71070	0.71062	0.00022
26.12	0.71110	0.71068	0.00025
26.08	0.71008	0.71061	0.00024
26.03	0.71094	0.71064	0.00021
25.99	0.71108	0.71057	0.00021
25.95	0.71072	0.71046	0.00021
25.90	0.71034	0.71042	0.00020
25.86	0.71045	0.71047	0.00022
25.82	0.71028	0.71048	0.00022
25.77	0.71055	0.71049	0.00022
25.73	0.71122	0.71043	0.00024
25.69	0.71042	0.71041	0.00022
25.64	0.71037	0.71043	0.00022
25.60	0.71023	0.71054	0.00030
25.56	0.71000	0.71057	0.00030
25.51	0.71030	0.71058	0.00029
25.47	0.71089	0.71062	0.00028
25.42	0.71054	0.71058	0.00028
25.38	0.71036	0.71052	0.00031
25.34	0.70996	0.71048	0.00032
25.29	0.71106	0.71056	0.00031
25.25	0.71060	0.71048	0.00029
25.21	0.71147	0.71051	0.00030
25.16	0.71058	0.71042	0.00021
25.12	0.71006	0.71044	0.00023
25.08	0.71069	0.71053	0.00023
25.03	0.71049	0.71053	0.00023
24.99	0.70995	0.71050	0.00024
24.95	0.70997	0.71058	0.00021
24.90	0.71073	0.71060	0.00017
24.86	0.71026	0.71061	0.00018
24.82	0.71086	0.71063	0.00016
24.77	0.71059	0.71058	0.00016
24.73	0.71082	0.71058	0.00016

15.07	0.71092	0.71066	0.00012	24.69	0.71095	0.71061	0.00020
15.02	0.71050	0.71060	0.00013	24.64	0.71063	0.71053	0.00021
14.98	0.71086	0.71063	0.00014	24.60	0.71020	0.71045	0.00025
14.94	0.71072	0.71060	0.00013	24.56	0.71075	0.71047	0.00025
14.89	0.71073	0.71058	0.00012	24.51	0.71025	0.71041	0.00025
14.85	0.71045	0.71057	0.00012	24.47	0.71075	0.71049	0.00027
14.81	0.71072	0.71059	0.00012	24.43	0.71054	0.71048	0.00027
14.76	0.71039	0.71053	0.00014	24.38	0.71036	0.71045	0.00027
14.72	0.71082	0.71061	0.00018	24.34	0.71051	0.71049	0.00028
14.68	0.71051	0.71056	0.00018	24.29	0.71122	0.71047	0.00028
14.63	0.71025	0.71064	0.00023	24.25	0.71013	0.71038	0.00022
14.59	0.71085	0.71075	0.00025	24.21	0.70979	0.71037	0.00023
14.55	0.71060	0.71072	0.00025	24.16	0.71045	0.71043	0.00019
14.50	0.71047	0.71074	0.00025	24.12	0.71014	0.71041	0.00020
14.46	0.71062	0.71073	0.00025	24.08	0.71106	0.71040	0.00021
14.42	0.71068	0.71077	0.00026	24.03	0.71061	0.71040	0.00020
14.37	0.71015	0.71071	0.00029	23.99	0.71027	0.71036	0.00020
14.33	0.71111	0.71077	0.00026	23.95	0.71073	0.71038	0.00020
14.29	0.71036	0.71075	0.00025	23.90	0.71033	0.71032	0.00018
14.24	0.71126	0.71071	0.00030	23.86	0.71030	0.71035	0.00019
14.20	0.71138	0.71060	0.00028	23.82	0.70999	0.71037	0.00019
14.16	0.71059	0.71057	0.00025	23.77	0.71044	0.71045	0.00019
14.12	0.71074	0.71060	0.00026	23.73	0.71028	0.71043	0.00019
14.07	0.71042	0.71062	0.00026	23.69	0.71001	0.71043	0.00019
14.03	0.71099	0.71065	0.00026	23.64	0.71101	0.71044	0.00018
13.99	0.71012	0.71065	0.00026	23.60	0.71022	0.71035	0.00014
13.94	0.71073	0.71064	0.00027	23.56	0.71046	0.71039	0.00014
13.90	0.71097	0.71063	0.00027	23.51	0.71020	0.71034	0.00016
13.86	0.70992	0.71056	0.00027	23.47	0.71057	0.71032	0.00018
13.81	0.71020	0.71065	0.00023	23.43	0.71052	0.71025	0.00019
13.77	0.71100	0.71074	0.00022	23.38	0.71078	0.71019	0.00019
13.73	0.71095	0.71074	0.00022	23.34	0.71027	0.71007	0.00017
13.68	0.71087	0.71074	0.00022	23.30	0.71027	0.71005	0.00017
13.64	0.71077	0.71077	0.00025	23.25	0.71009	0.70998	0.00018
13.60	0.71100	0.71075	0.00025	23.21	0.71013	0.70992	0.00020
13.55	0.70999	0.71072	0.00025	23.16	0.71059	0.70991	0.00020
13.51	0.71057	0.71079	0.00019	23.12	0.70998	0.70974	0.00022
13.47	0.71030	0.71079	0.00019	23.08	0.70995	0.70976	0.00023
13.42	0.71079	0.71079	0.00019	23.03	0.70993	0.70971	0.00023
13.38	0.71112	0.71078	0.00019	22.99	0.70986	0.70963	0.00025
13.34	0.71099	0.71072	0.00018	22.95	0.70960	0.70961	0.00025
13.29	0.71095	0.71074	0.00019	22.90	0.71010	0.70961	0.00025
13.25	0.71125	0.71076	0.00020	22.86	0.70961	0.70957	0.00022
13.21	0.71049	0.71072	0.00018	22.82	0.70949	0.70957	0.00022
13.16	0.71073	0.71071	0.00018	22.77	0.70998	0.70959	0.00023
13.12	0.71065	0.71064	0.00022	22.73	0.70895	0.70956	0.00021
13.08	0.71062	0.71066	0.00022	22.69	0.71015	0.70962	0.00016
13.03	0.71029	0.71061	0.00025	22.64	0.70943	0.70956	0.00011
12.99	0.71073	0.71068	0.00026	22.60	0.70913	0.70961	0.00013
12.95	0.71051	0.71065	0.00026	22.56	0.70963	0.70962	0.00011

12.90	0.71119	0.71067	0.00026	22.51	0.70963	0.70962	0.00011
12.86	0.71111	0.71063	0.00023	22.47	0.70967	0.70964	0.00011
12.82	0.71088	0.71061	0.00022	22.43	0.70962	0.70974	0.00024
12.77	0.71036	0.71060	0.00022	22.38	0.70973	0.70976	0.00024
12.73	0.71011	0.71062	0.00021	22.34	0.70963	0.70971	0.00027
12.69	0.71083	0.71072	0.00020	22.30	0.70963	0.70971	0.00027
12.64	0.71006	0.71071	0.00020	22.25	0.70949	0.70971	0.00027
12.60	0.71105	0.71076	0.00015	22.21	0.70994	0.70972	0.00026
12.56	0.71042	0.71067	0.00017	22.17	0.70927	0.70968	0.00026
12.51	0.71070	0.71068	0.00016	22.12	0.70958	0.70971	0.00025
12.47	0.71079	0.71070	0.00017	22.08	0.70981	0.70972	0.00024
12.43	0.71094	0.71074	0.00019	22.03	0.71067	0.70972	0.00024
12.38	0.71076	0.71071	0.00019	21.99	0.70988	0.70959	0.00012
12.34	0.71051	0.71072	0.00019	21.95	0.70915	0.70951	0.00015
12.30	0.71115	0.71075	0.00019	21.90	0.70964	0.70961	0.00018
12.25	0.71068	0.71072	0.00016	21.86	0.70963	0.70956	0.00021
12.21	0.71057	0.71071	0.00017	21.82	0.70962	0.70954	0.00021
12.17	0.71022	0.71070	0.00017	21.77	0.70954	0.70949	0.00023
12.12	0.71047	0.71073	0.00014	21.73	0.70959	0.70946	0.00023
12.08	0.71094	0.71074	0.00014	21.69	0.70968	0.70953	0.00028
12.04	0.71115	0.71071	0.00013	21.64	0.70977	0.70946	0.00030
11.99	0.71068	0.71072	0.00013	21.60	0.70942	0.70952	0.00034
11.95	0.71087	0.71075	0.00015	21.56	0.70904	0.70951	0.00034
11.91	0.71079	0.71071	0.00015	21.51	0.71022	0.70961	0.00034
11.86	0.71083	0.71069	0.00015	21.47	0.70912	0.70960	0.00033
11.82	0.71058	0.71065	0.00016	21.43	0.70944	0.70967	0.00031
11.78	0.71049	0.71068	0.00016	21.38	0.70906	0.70976	0.00033
11.73	0.71054	0.71068	0.00016	21.34	0.70930	0.70985	0.00029
11.69	0.71049	0.71073	0.00017	21.30	0.71024	0.70989	0.00027
11.65	0.71072	0.71079	0.00018	21.25	0.70900	0.70992	0.00030
11.60	0.71115	0.71075	0.00020	21.21	0.71033	0.70994	0.00027
11.56	0.71103	0.71070	0.00018	21.17	0.70940	0.70990	0.00026
11.52	0.71047	0.71072	0.00021	21.12	0.71004	0.70987	0.00029
11.47	0.71055	0.71077	0.00020	21.08	0.71009	0.70977	0.00033
11.43	0.71047	0.71079	0.00020	21.04	0.70979	0.70979	0.00034
11.39	0.71084	0.71085	0.00020	20.99	0.71031	0.70980	0.00034
11.34	0.71047	0.71089	0.00021	20.95	0.70999	0.70973	0.00032
11.30	0.71106	0.71092	0.00018	20.90	0.70969	0.70972	0.00032
11.26	0.71108	0.71089	0.00019	20.86	0.71057	0.70972	0.00032
11.21	0.71032	0.71087	0.00018	20.82	0.70920	0.70959	0.00027
11.17	0.71067	0.71091	0.00014	20.77	0.70995	0.70967	0.00026
11.13	0.71129	0.71094	0.00013	20.73	0.70910	0.70964	0.00026
11.08	0.71094	0.71088	0.00011	20.69	0.70903	0.70969	0.00023
11.04	0.71073	0.71082	0.00015	20.64	0.71031	0.70980	0.00019
11.00	0.71114	0.71079	0.00017	20.60	0.70985	0.70979	0.00018
10.95	0.71117	0.71075	0.00015	20.56	0.70959	0.70981	0.00019
10.91	0.71085	0.71073	0.00013	20.51	0.70994	0.70977	0.00021
10.87	0.71070	0.71071	0.00013	20.47	0.70966	0.70978	0.00022
10.82	0.71088	0.71070	0.00013	20.43	0.70928	0.70976	0.00023
10.78	0.71076	0.71073	0.00017	20.38	0.70998	0.70979	0.00021

10.74	0.71096	0.71077	0.00019	20.34	0.70964	0.70983	0.00024
10.69	0.71069	0.71077	0.00019	20.30	0.70960	0.70981	0.00025
10.65	0.71034	0.71081	0.00019	20.25	0.71012	0.70986	0.00025
10.61	0.71046	0.71084	0.00017	20.21	0.71025	0.70982	0.00024
10.56	0.71067	0.71094	0.00018	20.17	0.71000	0.70971	0.00026
10.52	0.71100	0.71092	0.00020	20.12	0.70927	0.70972	0.00027
10.43	0.71061	0.71089	0.00020	20.08	0.71003	0.70975	0.00025
10.39	0.71061	0.71088	0.00020	20.04	0.70947	0.70977	0.00026
10.35	0.71123	0.71090	0.00020	19.99	0.70951	0.70978	0.00026
10.30	0.71113	0.71084	0.00019	19.95	0.71041	0.70976	0.00027
10.26	0.71096	0.71080	0.00018	19.91	0.70949	0.70964	0.00025
10.22	0.71108	0.71072	0.00022	19.86	0.71003	0.70965	0.00025
10.17	0.71067	0.71065	0.00021	19.82	0.70975	0.70967	0.00027
10.13	0.71142	0.71059	0.00024	19.77	0.70911	0.70969	0.00027
10.09	0.71049	0.71054	0.00018	19.73	0.71017	0.70968	0.00027
10.05	0.71073	0.71058	0.00019	19.69	0.70955	0.70968	0.00027
10.00	0.71052	0.71061	0.00020	19.64	0.71022	0.70962	0.00030
9.96	0.71076	0.71062	0.00020	19.60	0.70957	0.70954	0.00027
9.92	0.71069	0.71059	0.00020	19.56	0.70933	0.70959	0.00030
9.87	0.71071	0.71060	0.00021	19.51	0.70917	0.70966	0.00030
9.83	0.71011	0.71059	0.00021	19.47	0.70962	0.70968	0.00029
9.79	0.71042	0.71064	0.00018	19.43	0.71026	0.70966	0.00029
9.74	0.71006	0.71066	0.00017	19.38	0.70987	0.70964	0.00027
9.70	0.71093	0.71070	0.00011	19.34	0.70907	0.70969	0.00031
9.66	0.71091	0.71067	0.00010	19.30	0.71012	0.70972	0.00028
9.61	0.71094	0.71057	0.00017	19.25	0.70901	0.70964	0.00028
9.57	0.71068	0.71060	0.00019	19.21	0.70937	0.70974	0.00025
9.53	0.71048	0.71058	0.00019	19.17	0.71009	0.70976	0.00024
9.48	0.71079	0.71060	0.00019	19.12	0.71001	0.70971	0.00023
9.44	0.71055	0.71056	0.00019	19.08	0.70942	0.70958	0.00028
9.40	0.71062	0.71059	0.00020	19.04	0.70941	0.70966	0.00031
9.35	0.71058	0.71060	0.00020	18.99	0.71001	0.70967	0.00030
9.31	0.71053	0.71062	0.00020	18.95	0.71038	0.70964	0.00029
9.27	0.71063	0.71065	0.00021	18.91	0.70941	0.70953	0.00025
9.22	0.70994	0.71065	0.00021	18.86	0.70931	0.70956	0.00024
9.18	0.71117	0.71070	0.00014	18.82	0.70997	0.70957	0.00024
9.14	0.71049	0.71060	0.00014	18.78	0.70956	0.70954	0.00022
9.09	0.71072	0.71062	0.00014	18.73	0.70959	0.70955	0.00023
9.05	0.71039	0.71061	0.00013	18.69	0.70877	0.70949	0.00026
9.01	0.71082	0.71061	0.00013	18.64	0.71022	0.70963	0.00023
8.96	0.71073	0.71054	0.00015	18.60	0.70949	0.70954	0.00020
8.92	0.71081	0.71048	0.00017	18.56	0.70967	0.70953	0.00020
8.88	0.71080	0.71043	0.00015	18.51	0.70936	0.70962	0.00028
8.83	0.71064	0.71039	0.00013	18.47	0.70964	0.70973	0.00032
8.79	0.71047	0.71032	0.00014	18.43	0.70947	0.70970	0.00033
8.75	0.71017	0.71034	0.00015	18.38	0.70962	0.70978	0.00034
8.70	0.71069	0.71034	0.00015	18.34	0.70973	0.70973	0.00036
8.66	0.71055	0.71029	0.00013	18.30	0.70894	0.70973	0.00036
8.62	0.71040	0.71025	0.00012	18.25	0.71014	0.70980	0.00032
8.57	0.71014	0.71025	0.00012	18.21	0.70935	0.70975	0.00031

8.53	0.71013	0.71025	0.00012	18.17	0.70941	0.70975	0.00031
8.49	0.71029	0.71031	0.00016	18.12	0.71054	0.70984	0.00032
8.44	0.71037	0.71033	0.00016	18.08	0.71043	0.70975	0.00028
8.40	0.70999	0.71034	0.00016	18.04	0.70937	0.70970	0.00024
8.36	0.71065	0.71039	0.00014	17.99	0.71024	0.70973	0.00023
8.31	0.71015	0.71039	0.00015	17.95	0.70914	0.70966	0.00021
8.27	0.71023	0.71036	0.00018	17.91	0.70979	0.70971	0.00017
8.23	0.71019	0.71036	0.00018	17.86	0.70959	0.70970	0.00017
8.18	0.71036	0.71035	0.00019	17.82	0.70961	0.70970	0.00017
8.14	0.71010	0.71033	0.00019	17.78	0.70935	0.70971	0.00017
8.10	0.71080	0.71036	0.00019	17.73	0.71034	0.70971	0.00016
8.05	0.71051	0.71034	0.00017	17.69	0.70970	0.70958	0.00015
8.01	0.71039	0.71035	0.00018	17.65	0.70992	0.70956	0.00015
7.97	0.71050	0.71035	0.00018	17.60	0.70966	0.70961	0.00023
7.92	0.71071	0.71029	0.00019	17.56	0.70948	0.70963	0.00023
7.88	0.70984	0.71024	0.00017	17.51	0.70970	0.70966	0.00023
7.84	0.71025	0.71030	0.00015	17.47	0.70965	0.70963	0.00023
7.79	0.71001	0.71032	0.00015	17.43	0.70962	0.70962	0.00023
7.75	0.71020	0.71034	0.00014	17.38	0.70964	0.70965	0.00024
7.71	0.71036	0.71035	0.00014	17.34	0.70943	0.70963	0.00025
7.66	0.71064	0.71036	0.00014	17.30	0.70900	0.70968	0.00026
7.62	0.71063	0.71032	0.00013	17.25	0.70948	0.70977	0.00021
7.58	0.71036	0.71031	0.00012	17.21	0.71044	0.70978	0.00020
7.53	0.70993	0.71033	0.00013	17.17	0.70986	0.70964	0.00020
7.49	0.71017	0.71036	0.00010	17.12	0.70975	0.70967	0.00022
7.45	0.71047	0.71039	0.00010	17.08	0.70944	0.70966	0.00022
7.40	0.71044	0.71031	0.00018	17.04	0.70954	0.70966	0.00021
7.36	0.71022	0.71029	0.00017	16.99	0.70996	0.70967	0.00021
7.32	0.71026	0.71032	0.00018	16.95	0.70936	0.70963	0.00020
7.27	0.71050	0.71034	0.00018	16.91	0.70999	0.70963	0.00020
7.23	0.71023	0.71031	0.00018	16.86	0.70992	0.70961	0.00018
7.19	0.71053	0.71028	0.00019	16.82	0.70959	0.70962	0.00019
7.14	0.71057	0.71022	0.00019	16.78	0.70901	0.70959	0.00021
7.10	0.71017	0.71019	0.00018	16.73	0.71011	0.70968	0.00017
7.06	0.71054	0.71020	0.00018	16.69	0.70965	0.70961	0.00014
7.01	0.70966	0.71020	0.00018	16.65	0.70951	0.70961	0.00015
6.97	0.71024	0.71027	0.00014	16.60	0.70958	0.70961	0.00015
6.93	0.71046	0.71024	0.00016	16.56	0.70957	0.70960	0.00015
6.88	0.71051	0.71025	0.00017	16.52	0.70942	0.70955	0.00018
6.84	0.71021	0.71017	0.00019	16.47	0.70974	0.70958	0.00018
6.80	0.70991	0.71018	0.00020	16.43	0.71001	0.70953	0.00019
6.75	0.70996	0.71020	0.00019	16.38	0.70926	0.70947	0.00015
6.71	0.71026	0.71025	0.00019	16.34	0.70990	0.70952	0.00015
6.67	0.71026	0.71026	0.00019	16.30	0.70941	0.70945	0.00014
6.62	0.71055	0.71027	0.00019	16.25	0.70968	0.70942	0.00016
6.58	0.71038	0.71024	0.00018	16.21	0.70948	0.70944	0.00018
6.54	0.70988	0.71023	0.00018	16.17	0.70953	0.70945	0.00018
6.49	0.71063	0.71031	0.00018	16.12	0.70910	0.70945	0.00018
6.45	0.70969	0.71029	0.00016	16.08	0.70967	0.70949	0.00017
6.41	0.71031	0.71035	0.00010	16.04	0.70922	0.70943	0.00018

6.36	0.71008	0.71033	0.00011
6.32	0.71046	0.71039	0.00011
6.28	0.71035	0.71037	0.00011
6.23	0.71038	0.71038	0.00012
6.19	0.71025	0.71037	0.00012
6.15	0.71028	0.71041	0.00012
6.10	0.71066	0.71045	0.00013
6.06	0.71043	0.71045	0.00013
6.02	0.71033	0.71050	0.00016
5.98	0.71007	0.71056	0.00018
5.93	0.71065	0.71066	0.00017
5.89	0.71026	0.71068	0.00017
5.85	0.71050	0.71078	0.00018
5.80	0.71031	0.71087	0.00020
5.76	0.71062	0.71094	0.00016
5.72	0.71065	0.71103	0.00017
5.67	0.71070	0.71105	0.00015
5.63	0.71092	0.71115	0.00017
5.59	0.71095	0.71119	0.00017
5.54	0.71105	0.71120	0.00016
5.50	0.71082	0.71121	0.00016
5.46	0.71127	0.71128	0.00015
5.41	0.71137	0.71134	0.00019
5.37	0.71106	0.71138	0.00020
5.33	0.71145	0.71139	0.00019
5.28	0.71093	0.71141	0.00019
5.24	0.71164	0.71151	0.00020
5.20	0.71138	0.71150	0.00020
5.15	0.71103	0.71151	0.00020
5.11	0.71118	0.71157	0.00016
5.07	0.71152	0.71155	0.00018
5.02	0.71187	0.71155	0.00018
4.98	0.71170	0.71150	0.00017
4.94	0.71124	0.71150	0.00016
4.89	0.71156	0.71150	0.00017
4.85	0.71203	0.71149	0.00016
4.81	0.71154	0.71143	0.00011
4.76	0.71141	0.71140	0.00012
4.72	0.71159	0.71137	0.00013
4.68	0.71106	0.71133	0.00013
4.63	0.71150	0.71135	0.00011
4.59	0.71136	0.71133	0.00011
4.55	0.71167	0.71132	0.00011
4.50	0.71124	0.71125	0.00009
4.46	0.71149	0.71127	0.00010
4.42	0.71146	0.71118	0.00014
4.37	0.71117	0.71110	0.00017
4.33	0.71119	0.71105	0.00019
4.29	0.71113	0.71100	0.00020
4.24	0.71132	0.71096	0.00020

15.99	0.70942	0.70949	0.00018
15.95	0.70976	0.70954	0.00020
15.91	0.70921	0.70948	0.00021
15.86	0.70909	0.70952	0.00020
15.82	0.70993	0.70960	0.00019
15.78	0.70953	0.70956	0.00017
15.73	0.70954	0.70949	0.00023
15.69	0.70951	0.70944	0.00025
15.65	0.70912	0.70946	0.00026
15.60	0.70983	0.70947	0.00025
15.56	0.70993	0.70943	0.00024
15.52	0.70913	0.70935	0.00022
15.47	0.70965	0.70935	0.00022
15.43	0.70982	0.70932	0.00021
15.39	0.70960	0.70927	0.00018
15.34	0.70878	0.70926	0.00018
15.30	0.70901	0.70932	0.00014
15.25	0.70973	0.70941	0.00016
15.21	0.70921	0.70938	0.00014
15.17	0.70944	0.70945	0.00017
15.12	0.70914	0.70942	0.00018
15.08	0.70907	0.70947	0.00018
15.04	0.70941	0.70947	0.00018
14.99	0.70931	0.70946	0.00019
14.95	0.70952	0.70945	0.00019
14.91	0.70940	0.70941	0.00020
14.86	0.70988	0.70939	0.00021
14.82	0.70942	0.70936	0.00019
14.78	0.70991	0.70937	0.00019
14.73	0.70912	0.70924	0.00021
14.69	0.70971	0.70925	0.00021
14.65	0.70904	0.70920	0.00018
14.60	0.70931	0.70925	0.00019
14.56	0.70924	0.70927	0.00020
14.52	0.70913	0.70930	0.00021
14.47	0.70912	0.70932	0.00020
14.43	0.70962	0.70930	0.00022
14.39	0.70952	0.70928	0.00021
14.34	0.70855	0.70925	0.00020
14.30	0.70925	0.70933	0.00012
14.26	0.70922	0.70936	0.00013
14.21	0.70955	0.70944	0.00017
14.17	0.70946	0.70945	0.00018
14.12	0.70959	0.70942	0.00019
14.08	0.70937	0.70935	0.00020
14.04	0.70890	0.70934	0.00020
13.99	0.70941	0.70937	0.00018
13.95	0.70922	0.70934	0.00019
13.91	0.70932	0.70937	0.00019
13.86	0.70961	0.70943	0.00021

4.20	0.71125	0.71087	0.00021	13.82	0.70993	0.70937	0.00022
4.16	0.71130	0.71076	0.00024	13.78	0.70967	0.70933	0.00019
4.11	0.71099	0.71066	0.00022	13.73	0.70914	0.70933	0.00018
4.07	0.71136	0.71061	0.00021	13.69	0.70896	0.70940	0.00021
4.03	0.71067	0.71050	0.00013	13.65	0.70924	0.70945	0.00018
3.98	0.71058	0.71045	0.00014	13.60	0.70921	0.70947	0.00018
3.94	0.71066	0.71043	0.00013	13.56	0.70915	0.70951	0.00017
3.90	0.71070	0.71041	0.00012	13.52	0.70949	0.70946	0.00023
3.85	0.71077	0.71038	0.00011	13.47	0.70987	0.70951	0.00025
3.81	0.71039	0.71033	0.00007	13.43	0.70904	0.70943	0.00025
3.77	0.71014	0.71031	0.00007	13.39	0.70951	0.70946	0.00023
3.72	0.71034	0.71034	0.00006	13.34	0.70965	0.70948	0.00023
3.68	0.71045	0.71033	0.00006	13.30	0.70987	0.70943	0.00024
3.64	0.71027	0.71032	0.00006	13.26	0.70952	0.70936	0.00022
3.59	0.71022	0.71030	0.00008	13.21	0.70940	0.70935	0.00022
3.55	0.71040	0.71033	0.00008	13.17	0.70957	0.70929	0.00025
3.51	0.71045	0.71028	0.00010	13.13	0.70869	0.70926	0.00024
3.46	0.71037	0.71023	0.00012	13.08	0.70995	0.70938	0.00023
3.42	0.71026	0.71023	0.00011	13.04	0.70906	0.70930	0.00020
3.38	0.71023	0.71020	0.00012	12.99	0.70941	0.70930	0.00020
3.33	0.71045	0.71020	0.00012	12.95	0.70964	0.70926	0.00020
3.29	0.71024	0.71015	0.00013	12.91	0.70918	0.70922	0.00018
3.25	0.71034	0.71012	0.00013	12.86	0.70920	0.70923	0.00018
3.20	0.71008	0.71011	0.00012	12.82	0.70942	0.70927	0.00020
3.16	0.71044	0.71013	0.00013	12.78	0.70875	0.70924	0.00019
3.12	0.70996	0.71014	0.00014	12.73	0.70932	0.70925	0.00018
3.07	0.70995	0.71017	0.00013	12.69	0.70986	0.70927	0.00019
3.03	0.71033	0.71023	0.00015	12.65	0.70912	0.70924	0.00016
2.99	0.70997	0.71022	0.00015	12.60	0.70906	0.70928	0.00016
2.94	0.71027	0.71023	0.00014	12.56	0.70904	0.70928	0.00016
2.90	0.70989	0.71022	0.00014	12.52	0.70923	0.70930	0.00015
2.86	0.70996	0.71029	0.00014	12.47	0.70927	0.70929	0.00015
2.81	0.71027	0.71029	0.00013	12.43	0.70958	0.70936	0.00019
2.77	0.71028	0.71025	0.00016	12.39	0.70918	0.70930	0.00020
2.73	0.71054	0.71023	0.00016	12.34	0.70886	0.70929	0.00020
2.68	0.71021	0.71020	0.00014	12.30	0.70949	0.70935	0.00018
2.64	0.71057	0.71024	0.00016	12.26	0.70961	0.70936	0.00018
2.60	0.71027	0.71021	0.00015	12.21	0.70944	0.70935	0.00018
2.55	0.71000	0.71019	0.00015	12.17	0.70907	0.70936	0.00018
2.51	0.71023	0.71022	0.00014	12.13	0.70928	0.70946	0.00022
2.47	0.71055	0.71022	0.00014	12.08	0.70916	0.70945	0.00022
2.42	0.70998	0.71021	0.00013	12.04	0.70991	0.70949	0.00021
2.38	0.70989	0.71027	0.00014	12.00	0.70898	0.70947	0.00019
2.34	0.71007	0.71032	0.00012	11.95	0.70914	0.70953	0.00016
2.29	0.71025	0.71035	0.00010	11.91	0.70947	0.70957	0.00013
2.25	0.71058	0.71036	0.00010	11.86	0.70955	0.70956	0.00013
2.21	0.71025	0.71031	0.00011	11.82	0.70954	0.70953	0.00015
2.16	0.71010	0.71031	0.00012	11.78	0.70947	0.70945	0.00021
2.12	0.71025	0.71030	0.00013	11.73	0.71007	0.70942	0.00022
2.08	0.71024	0.71034	0.00014	11.69	0.70925	0.70938	0.00017

2.03	0.71047	0.71031	0.00016
1.99	0.71062	0.71032	0.00017
1.95	0.71038	0.71027	0.00016
1.91	0.71037	0.71024	0.00016
1.86	0.71038	0.71022	0.00016
1.82	0.70999	0.71022	0.00016
1.78	0.71003	0.71026	0.00015
1.73	0.71058	0.71029	0.00014
1.69	0.70993	0.71031	0.00015
1.65	0.71060	0.71036	0.00013
1.60	0.71014	0.71032	0.00012
1.56	0.71013	0.71038	0.00014
1.52	0.71016	0.71037	0.00015
1.47	0.71044	0.71036	0.00016
1.43	0.71022	0.71037	0.00016
1.39	0.71041	0.71033	0.00018
1.34	0.71030	0.71029	0.00020
1.30	0.71075	0.71025	0.00021
1.26	0.71043	0.71018	0.00018
1.21	0.71025	0.71012	0.00019
1.17	0.71076	0.71011	0.00018
1.13	0.71002	0.71007	0.00014
1.08	0.71005	0.71005	0.00015
1.04	0.71048	0.71003	0.00016
1.00	0.70990	0.71001	0.00013
0.95	0.70994	0.71004	0.00013
0.91	0.70995	0.71003	0.00013
0.87	0.71005	0.71006	0.00014
0.82	0.70984	0.71008	0.00014
0.78	0.71008	0.71008	0.00014
0.74	0.71044	0.71011	0.00015
0.69	0.70976	0.71005	0.00014
0.65	0.70989	0.71010	0.00012
0.61	0.71024	0.71010	0.00012
0.56	0.71020	0.71008	0.00012
0.52	0.70989	0.71008	0.00012
0.48	0.71020	0.71010	0.00011
0.43	0.71025	0.71009	0.00011
0.39	0.70985	0.71007	0.00011
0.35	0.71037	0.71010	0.00010
0.30	0.70987	0.71006	0.00008
0.26	0.71022	0.71009	0.00007
0.22	0.70990	0.71006	0.00006
0.17	0.71007	0.71010	0.00002
0.13	0.71014	0.71011	0.00002
0.09	0.71009	0.71010	0.00001
0.04	0.71011		

11.65	0.70952	0.70940	0.00017
11.60	0.70968	0.70937	0.00017
11.56	0.70956	0.70935	0.00016
11.52	0.70960	0.70936	0.00016
11.47	0.70938	0.70933	0.00015
11.43	0.70922	0.70934	0.00016
11.39	0.70879	0.70935	0.00015
11.34	0.70917	0.70942	0.00009
11.30	0.70963	0.70943	0.00008
11.26	0.70941	0.70942	0.00007
11.21	0.70929	0.70944	0.00008
11.17	0.70946	0.70948	0.00009
11.13	0.70963	0.70946	0.00010
11.08	0.70931	0.70945	0.00009
11.04	0.70944	0.70944	0.00010
11.00	0.70938	0.70946	0.00011
10.95	0.70943	0.70943	0.00013
10.91	0.70929	0.70944	0.00014
10.87	0.70950	0.70944	0.00014
10.82	0.70964	0.70943	0.00014
10.78	0.70969	0.70940	0.00013
10.73	0.70923	0.70936	0.00012
10.69	0.70954	0.70935	0.00012
10.65	0.70922	0.70929	0.00013
10.60	0.70964	0.70927	0.00014
10.56	0.70908	0.70928	0.00016
10.52	0.70956	0.70932	0.00016
10.47	0.70926	0.70932	0.00015
10.43	0.70947	0.70930	0.00016
10.39	0.70926	0.70930	0.00016
10.34	0.70932	0.70930	0.00016
10.30	0.70914	0.70924	0.00019
10.26	0.70897	0.70928	0.00020
10.21	0.70902	0.70932	0.00019
10.17	0.70977	0.70934	0.00018
10.13	0.70944	0.70929	0.00015
10.08	0.70954	0.70924	0.00016
10.04	0.70912	0.70920	0.00015
10.00	0.70946	0.70927	0.00019
9.95	0.70922	0.70919	0.00023
9.91	0.70874	0.70917	0.00023
9.87	0.70955	0.70924	0.00022
9.82	0.70938	0.70920	0.00020
9.78	0.70923	0.70917	0.00020
9.74	0.70925	0.70917	0.00020
9.69	0.70894	0.70922	0.00024
9.65	0.70916	0.70922	0.00024
9.60	0.70980	0.70924	0.00024
9.56	0.70859	0.70919	0.00021
9.52	0.70903	0.70933	0.00022

9.47	0.70948	0.70933	0.00022
9.43	0.70917	0.70935	0.00023
9.39	0.70902	0.70939	0.00023
9.34	0.70921	0.70937	0.00024
9.30	0.70982	0.70934	0.00027
9.26	0.70895	0.70922	0.00027
9.21	0.70933	0.70925	0.00026
9.17	0.70930	0.70923	0.00026
9.13	0.71000	0.70925	0.00027
9.08	0.70904	0.70912	0.00024
9.04	0.70964	0.70913	0.00024
9.00	0.70963	0.70906	0.00021
8.95	0.70883	0.70904	0.00018
8.91	0.70883	0.70911	0.00020
8.87	0.70869	0.70913	0.00019
8.82	0.70918	0.70918	0.00016
8.78	0.70918	0.70915	0.00017
8.74	0.70948	0.70916	0.00018
8.69	0.70864	0.70917	0.00018
8.65	0.70914	0.70919	0.00016
8.61	0.70900	0.70914	0.00019
8.56	0.70943	0.70912	0.00020
8.52	0.70948	0.70911	0.00019
8.47	0.70911	0.70907	0.00017
8.43	0.70916	0.70910	0.00019
8.39	0.70884	0.70912	0.00019
8.34	0.70930	0.70912	0.00019
8.30	0.70956	0.70908	0.00019
8.26	0.70887	0.70905	0.00017
8.21	0.70865	0.70902	0.00020
8.17	0.70883	0.70909	0.00018
8.13	0.70930	0.70913	0.00018
8.08	0.70905	0.70917	0.00021
8.04	0.70940	0.70920	0.00021
8.00	0.70936	0.70916	0.00020
7.95	0.70893	0.70913	0.00020
7.91	0.70880	0.70916	0.00020
7.87	0.70932	0.70918	0.00019
7.82	0.70858	0.70917	0.00018
7.78	0.70928	0.70923	0.00013
7.74	0.70932	0.70923	0.00013
7.69	0.70966	0.70921	0.00013
7.65	0.70935	0.70915	0.00008
7.61	0.70903	0.70910	0.00008
7.56	0.70902	0.70909	0.00009
7.52	0.70926	0.70912	0.00010
7.48	0.70897	0.70907	0.00011
7.43	0.70923	0.70912	0.00013
7.39	0.70922	0.70915	0.00015
7.34	0.70925	0.70915	0.00015

7.30	0.70910	0.70913	0.00015
7.26	0.70906	0.70916	0.00016
7.21	0.70889	0.70914	0.00017
7.17	0.70891	0.70916	0.00016
7.13	0.70928	0.70922	0.00016
7.08	0.70882	0.70923	0.00016
7.04	0.70946	0.70928	0.00014
7.00	0.70952	0.70925	0.00013
6.95	0.70920	0.70921	0.00012
6.91	0.70901	0.70918	0.00014
6.87	0.70940	0.70924	0.00016
6.82	0.70889	0.70921	0.00015
6.78	0.70913	0.70927	0.00014
6.74	0.70944	0.70925	0.00016
6.69	0.70945	0.70921	0.00015
6.65	0.70931	0.70916	0.00015
6.61	0.70913	0.70917	0.00015
6.56	0.70917	0.70919	0.00016
6.52	0.70887	0.70922	0.00017
6.48	0.70961	0.70924	0.00015
6.43	0.70912	0.70921	0.00013
6.39	0.70946	0.70921	0.00013
6.35	0.70892	0.70917	0.00012
6.30	0.70910	0.70924	0.00013
6.26	0.70892	0.70927	0.00013
6.21	0.70936	0.70931	0.00010
6.17	0.70939	0.70932	0.00011
6.13	0.70942	0.70931	0.00011
6.08	0.70913	0.70929	0.00011
6.04	0.70927	0.70930	0.00010
6.00	0.70910	0.70927	0.00012
5.95	0.70908	0.70927	0.00012
5.91	0.70958	0.70927	0.00012
5.87	0.70944	0.70924	0.00010
5.82	0.70928	0.70926	0.00012
5.78	0.70949	0.70921	0.00015
5.74	0.70930	0.70918	0.00014
5.69	0.70917	0.70916	0.00014
5.65	0.70923	0.70914	0.00014
5.61	0.70902	0.70913	0.00014
5.56	0.70913	0.70914	0.00014
5.52	0.70905	0.70915	0.00014
5.48	0.70925	0.70917	0.00014
5.43	0.70964	0.70915	0.00014
5.39	0.70878	0.70913	0.00011
5.35	0.70920	0.70919	0.00008
5.30	0.70914	0.70917	0.00009
5.26	0.70900	0.70916	0.00009
5.22	0.70915	0.70921	0.00010
5.17	0.70911	0.70918	0.00012

5.13	0.70924	0.70915	0.00014
5.08	0.70916	0.70916	0.00015
5.04	0.70911	0.70916	0.00015
5.00	0.70945	0.70915	0.00015
4.95	0.70933	0.70912	0.00013
4.91	0.70898	0.70915	0.00016
4.87	0.70911	0.70915	0.00016
4.82	0.70942	0.70911	0.00019
4.78	0.70883	0.70907	0.00017
4.74	0.70885	0.70909	0.00017
4.69	0.70938	0.70914	0.00017
4.65	0.70914	0.70912	0.00016
4.61	0.70904	0.70911	0.00016
4.56	0.70912	0.70911	0.00016
4.52	0.70962	0.70910	0.00016
4.48	0.70897	0.70903	0.00011
4.43	0.70869	0.70904	0.00012
4.39	0.70904	0.70909	0.00009
4.35	0.70901	0.70908	0.00009
4.30	0.70938	0.70912	0.00011
4.26	0.70920	0.70910	0.00009
4.22	0.70903	0.70911	0.00010
4.17	0.70901	0.70913	0.00011
4.13	0.70906	0.70918	0.00012
4.09	0.70888	0.70916	0.00014
4.04	0.70915	0.70920	0.00012
4.00	0.70914	0.70921	0.00012
3.95	0.70895	0.70922	0.00012
3.91	0.70940	0.70921	0.00013
3.87	0.70916	0.70924	0.00016
3.82	0.70935	0.70927	0.00016
3.78	0.70925	0.70925	0.00017
3.74	0.70947	0.70925	0.00017
3.69	0.70885	0.70923	0.00016
3.65	0.70931	0.70926	0.00014
3.61	0.70922	0.70927	0.00014
3.56	0.70927	0.70925	0.00015
3.52	0.70885	0.70922	0.00016
3.48	0.70964	0.70924	0.00014
3.43	0.70949	0.70923	0.00013
3.39	0.70910	0.70917	0.00013
3.35	0.70927	0.70914	0.00015
3.30	0.70931	0.70915	0.00015
3.26	0.70909	0.70910	0.00015
3.22	0.70942	0.70912	0.00016
3.17	0.70902	0.70912	0.00016
3.13	0.70901	0.70915	0.00016
3.09	0.70900	0.70914	0.00017
3.04	0.70953	0.70916	0.00017
3.00	0.70889	0.70914	0.00016

2.96	0.70884	0.70916	0.00015
2.91	0.70934	0.70921	0.00013
2.87	0.70887	0.70919	0.00013
2.82	0.70930	0.70921	0.00011
2.78	0.70937	0.70919	0.00011
2.74	0.70939	0.70917	0.00010
2.69	0.70886	0.70915	0.00009
2.65	0.70917	0.70917	0.00006
2.61	0.70941	0.70919	0.00008
2.56	0.70909	0.70917	0.00006
2.52	0.70927	0.70918	0.00006
2.48	0.70913	0.70914	0.00007
2.43	0.70911	0.70912	0.00009
2.39	0.70912	0.70911	0.00009
2.35	0.70913	0.70907	0.00013
2.30	0.70917	0.70910	0.00015
2.26	0.70912	0.70910	0.00015
2.22	0.70940	0.70912	0.00015
2.17	0.70919	0.70907	0.00014
2.13	0.70912	0.70909	0.00015
2.09	0.70894	0.70907	0.00015
2.04	0.70888	0.70910	0.00015
2.00	0.70908	0.70913	0.00015
1.96	0.70865	0.70912	0.00015
1.91	0.70942	0.70919	0.00012
1.87	0.70922	0.70916	0.00010
1.83	0.70932	0.70915	0.00010
1.78	0.70894	0.70917	0.00012
1.74	0.70934	0.70919	0.00011
1.69	0.70896	0.70917	0.00010
1.65	0.70919	0.70917	0.00010
1.61	0.70923	0.70913	0.00013
1.56	0.70892	0.70910	0.00014
1.52	0.70934	0.70914	0.00014
1.48	0.70917	0.70912	0.00013
1.43	0.70912	0.70910	0.00013
1.39	0.70945	0.70911	0.00013
1.35	0.70916	0.70905	0.00011
1.30	0.70918	0.70905	0.00011
1.26	0.70894	0.70901	0.00012
1.22	0.70874	0.70900	0.00012
1.17	0.70896	0.70899	0.00013
1.13	0.70935	0.70900	0.00013
1.09	0.70912	0.70898	0.00011
1.04	0.70899	0.70899	0.00011
1.00	0.70917	0.70901	0.00012
0.96	0.70889	0.70902	0.00013
0.91	0.70914	0.70902	0.00013
0.87	0.70877	0.70902	0.00012
0.83	0.70886	0.70902	0.00012

0.78	0.70867	0.70903	0.00012
0.74	0.70909	0.70907	0.00009
0.70	0.70908	0.70907	0.00009
0.65	0.70919	0.70904	0.00011
0.61	0.70923	0.70901	0.00010
0.56	0.70924	0.70897	0.00009
0.52	0.70894	0.70897	0.00009
0.48	0.70909	0.70898	0.00009
0.43	0.70883	0.70896	0.00008
0.39	0.70889	0.70897	0.00008
0.35	0.70909	0.70898	0.00009
0.30	0.70908	0.70896	0.00009
0.26	0.70877	0.70894	0.00009
0.22	0.70892	0.70898	0.00008
0.17	0.70888	0.70899	0.00009
0.13	0.70919	0.70903	0.00010
0.09	0.70902	0.70895	0.00006
0.04	0.70888		

ARB 19.2.1 (M3)

Distance from cervix (mm)	$^{87}Sr/^{86}Sr$	10 point mov. average	2 SE on mov. average
45.55	0.70857	0.70849	0.00012
45.50	0.70861	0.70848	0.00012
45.44	0.70848	0.70847	0.00012
45.39	0.70835	0.70847	0.00012
45.33	0.70861	0.70849	0.00012
45.28	0.70869	0.70849	0.00012
45.23	0.70878	0.70846	0.00011
45.17	0.70844	0.70844	0.00009
45.12	0.70815	0.70843	0.00009
45.07	0.70825	0.70846	0.00006
45.01	0.70846	0.70846	0.00006
44.96	0.70851	0.70849	0.00008
44.90	0.70850	0.70849	0.00008
44.85	0.70856	0.70849	0.00008
44.80	0.70852	0.70846	0.00010
44.74	0.70847	0.70848	0.00012
44.69	0.70858	0.70848	0.00012
44.63	0.70834	0.70844	0.00013
44.58	0.70841	0.70845	0.00013
44.53	0.70829	0.70846	0.00013
44.47	0.70871	0.70849	0.00013
44.42	0.70856	0.70847	0.00012
44.37	0.70849	0.70846	0.00012
44.31	0.70818	0.70848	0.00012
44.26	0.70878	0.70852	0.00010
44.20	0.70846	0.70852	0.00011
44.15	0.70816	0.70850	0.00012
44.10	0.70845	0.70851	0.00011
44.04	0.70849	0.70852	0.00011
43.99	0.70864	0.70854	0.00011
43.93	0.70851	0.70849	0.00014
43.88	0.70849	0.70845	0.00016
43.83	0.70860	0.70842	0.00016
43.77	0.70859	0.70840	0.00016
43.72	0.70882	0.70841	0.00017
43.67	0.70830	0.70836	0.00015
43.61	0.70823	0.70840	0.00016
43.56	0.70853	0.70843	0.00016
43.50	0.70872	0.70841	0.00016
43.45	0.70814	0.70840	0.00015
43.40	0.70810	0.70843	0.00014
43.34	0.70820	0.70845	0.00012
43.29	0.70834	0.70848	0.00011
43.23	0.70875	0.70849	0.00011
43.18	0.70830	0.70848	0.00010
43.13	0.70866	0.70854	0.00012
43.07	0.70859	0.70855	0.00012

43.02	0.70831	0.70856	0.00013
42.97	0.70863	0.70860	0.00012
42.91	0.70843	0.70862	0.00012
42.86	0.70824	0.70861	0.00013
42.80	0.70853	0.70864	0.00011
42.75	0.70849	0.70863	0.00012
42.70	0.70866	0.70861	0.00013
42.64	0.70888	0.70859	0.00013
42.59	0.70873	0.70858	0.00012
42.53	0.70873	0.70854	0.00012
42.48	0.70871	0.70851	0.00011
42.43	0.70881	0.70848	0.00010
42.37	0.70833	0.70846	0.00008
42.32	0.70848	0.70850	0.00008
42.27	0.70843	0.70849	0.00009
42.21	0.70830	0.70851	0.00009
42.16	0.70853	0.70851	0.00010
42.10	0.70870	0.70853	0.00010
42.05	0.70836	0.70850	0.00010
42.00	0.70843	0.70852	0.00009
41.94	0.70846	0.70855	0.00010
41.89	0.70855	0.70855	0.00010
41.83	0.70871	0.70854	0.00010
41.78	0.70841	0.70856	0.00013
41.73	0.70869	0.70858	0.00013
41.67	0.70827	0.70859	0.00013
41.62	0.70869	0.70862	0.00011
41.57	0.70839	0.70859	0.00012
41.51	0.70856	0.70863	0.00011
41.46	0.70872	0.70863	0.00011
41.40	0.70853	0.70862	0.00011
41.35	0.70839	0.70864	0.00011
41.30	0.70898	0.70867	0.00010
41.24	0.70863	0.70863	0.00007
41.19	0.70870	0.70860	0.00009
41.13	0.70862	0.70856	0.00011
41.08	0.70839	0.70853	0.00011
41.03	0.70875	0.70853	0.00011
40.97	0.70859	0.70849	0.00010
40.92	0.70862	0.70851	0.00011
40.87	0.70877	0.70849	0.00011
40.81	0.70839	0.70847	0.00008
40.76	0.70832	0.70847	0.00008
40.70	0.70845	0.70854	0.00013
40.65	0.70831	0.70857	0.00014
40.60	0.70847	0.70858	0.00013
40.54	0.70875	0.70858	0.00013
40.49	0.70842	0.70856	0.00013
40.44	0.70855	0.70857	0.00012
40.38	0.70861	0.70858	0.00012

40.33	0.70841	0.70859	0.00012
40.27	0.70845	0.70860	0.00012
40.22	0.70903	0.70860	0.00012
40.17	0.70875	0.70853	0.00009
40.11	0.70840	0.70853	0.00008
40.06	0.70848	0.70850	0.00011
40.00	0.70847	0.70848	0.00012
39.95	0.70857	0.70849	0.00013
39.90	0.70863	0.70850	0.00013
39.84	0.70867	0.70852	0.00014
39.79	0.70852	0.70851	0.00014
39.74	0.70850	0.70850	0.00014
39.68	0.70829	0.70854	0.00016
39.63	0.70875	0.70858	0.00016
39.57	0.70813	0.70857	0.00015
39.52	0.70827	0.70861	0.00012
39.47	0.70858	0.70862	0.00011
39.41	0.70863	0.70860	0.00012
39.36	0.70886	0.70859	0.00012
39.30	0.70859	0.70854	0.00011
39.25	0.70842	0.70852	0.00012
39.20	0.70891	0.70855	0.00012
39.14	0.70869	0.70848	0.00010
39.09	0.70867	0.70849	0.00011
39.04	0.70849	0.70847	0.00010
38.98	0.70841	0.70846	0.00010
38.93	0.70834	0.70847	0.00010
38.87	0.70853	0.70849	0.00010
38.82	0.70840	0.70847	0.00010
38.77	0.70832	0.70847	0.00010
38.71	0.70873	0.70849	0.00010
38.66	0.70826	0.70848	0.00008
38.60	0.70871	0.70849	0.00007
38.55	0.70852	0.70848	0.00006
38.50	0.70841	0.70846	0.00007
38.44	0.70845	0.70848	0.00007
38.39	0.70856	0.70850	0.00009
38.34	0.70833	0.70852	0.00010
38.28	0.70840	0.70857	0.00011
38.23	0.70857	0.70859	0.00010
38.17	0.70857	0.70858	0.00010
38.12	0.70837	0.70856	0.00011
38.07	0.70859	0.70859	0.00011
38.01	0.70832	0.70860	0.00011
37.96	0.70859	0.70862	0.00009
37.90	0.70873	0.70862	0.00009
37.85	0.70876	0.70862	0.00009
37.80	0.70881	0.70863	0.00010
37.74	0.70860	0.70861	0.00010
37.69	0.70847	0.70866	0.00014

37.64	0.70835	0.70867	0.00013
37.58	0.70871	0.70866	0.00014
37.53	0.70866	0.70867	0.00015
37.47	0.70854	0.70864	0.00016
37.42	0.70859	0.70863	0.00016
37.37	0.70868	0.70861	0.00017
37.31	0.70892	0.70861	0.00017
37.26	0.70852	0.70856	0.00015
37.20	0.70910	0.70854	0.00016
37.15	0.70857	0.70850	0.00011
37.10	0.70826	0.70849	0.00011
37.04	0.70882	0.70851	0.00009
36.99	0.70841	0.70849	0.00007
36.94	0.70843	0.70854	0.00010
36.88	0.70842	0.70852	0.00011
36.83	0.70867	0.70852	0.00011
36.77	0.70844	0.70858	0.00018
36.72	0.70833	0.70862	0.00018
36.67	0.70863	0.70866	0.00017
36.61	0.70846	0.70862	0.00018
36.56	0.70849	0.70861	0.00019
36.50	0.70866	0.70858	0.00021
36.45	0.70883	0.70853	0.00023
36.40	0.70831	0.70850	0.00022
36.34	0.70838	0.70851	0.00021
36.29	0.70922	0.70853	0.00021
36.24	0.70884	0.70844	0.00015
36.18	0.70873	0.70838	0.00013
36.13	0.70832	0.70838	0.00013
36.07	0.70836	0.70842	0.00014
36.02	0.70818	0.70843	0.00014
35.97	0.70810	0.70847	0.00014
35.91	0.70852	0.70853	0.00011
35.86	0.70843	0.70856	0.00013
35.80	0.70861	0.70856	0.00012
35.75	0.70832	0.70854	0.00013
35.70	0.70821	0.70861	0.00015
35.64	0.70878	0.70866	0.00012
35.59	0.70868	0.70860	0.00015
35.54	0.70845	0.70863	0.00016
35.48	0.70863	0.70864	0.00016
35.43	0.70866	0.70865	0.00016
35.37	0.70883	0.70865	0.00016
35.32	0.70848	0.70861	0.00016
35.27	0.70836	0.70864	0.00017
35.21	0.70898	0.70870	0.00016
35.16	0.70878	0.70866	0.00015
35.10	0.70819	0.70865	0.00015
35.05	0.70892	0.70868	0.00012
35.00	0.70856	0.70866	0.00011

34.94	0.70869	0.70866	0.00011
34.89	0.70875	0.70867	0.00012
34.84	0.70836	0.70864	0.00013
34.78	0.70880	0.70866	0.00011
34.73	0.70897	0.70867	0.00012
34.67	0.70853	0.70860	0.00013
34.62	0.70870	0.70858	0.00014
34.57	0.70851	0.70855	0.00014
34.51	0.70879	0.70856	0.00015
34.46	0.70851	0.70855	0.00014
34.40	0.70884	0.70859	0.00015
34.35	0.70844	0.70855	0.00015
34.30	0.70855	0.70854	0.00015
34.24	0.70890	0.70855	0.00015
34.19	0.70823	0.70850	0.00013
34.14	0.70830	0.70849	0.00013
34.08	0.70840	0.70848	0.00014
34.03	0.70868	0.70849	0.00014
33.97	0.70869	0.70847	0.00014
33.92	0.70887	0.70847	0.00014
33.87	0.70842	0.70844	0.00011
33.81	0.70840	0.70844	0.00011
33.76	0.70860	0.70842	0.00011
33.70	0.70839	0.70841	0.00011
33.65	0.70820	0.70844	0.00012
33.60	0.70813	0.70844	0.00011
33.54	0.70851	0.70847	0.00009
33.49	0.70851	0.70848	0.00009
33.44	0.70865	0.70844	0.00012
33.38	0.70858	0.70841	0.00011
33.33	0.70839	0.70847	0.00017
33.27	0.70825	0.70849	0.00018
33.22	0.70849	0.70854	0.00017
33.17	0.70863	0.70853	0.00018
33.11	0.70823	0.70853	0.00018
33.06	0.70845	0.70858	0.00017
33.00	0.70857	0.70863	0.00018
32.95	0.70814	0.70862	0.00018
32.90	0.70839	0.70865	0.00016
32.84	0.70912	0.70866	0.00015
32.79	0.70867	0.70862	0.00012
32.74	0.70872	0.70861	0.00012
32.68	0.70837	0.70856	0.00015
32.63	0.70860	0.70856	0.00014
32.57	0.70880	0.70856	0.00014
32.52	0.70893	0.70856	0.00014
32.47	0.70847	0.70850	0.00012
32.41	0.70842	0.70849	0.00012
32.36	0.70847	0.70852	0.00013
32.30	0.70873	0.70853	0.00013

32.25	0.70863	0.70851	0.00012
32.20	0.70815	0.70848	0.00012
32.14	0.70841	0.70852	0.00010
32.09	0.70855	0.70855	0.00010
32.04	0.70881	0.70854	0.00010
31.98	0.70835	0.70855	0.00011
31.93	0.70842	0.70860	0.00012
31.87	0.70867	0.70863	0.00011
31.82	0.70856	0.70861	0.00011
31.77	0.70860	0.70863	0.00012
31.71	0.70830	0.70862	0.00012
31.66	0.70859	0.70862	0.00012
31.61	0.70863	0.70864	0.00012
31.55	0.70848	0.70865	0.00013
31.50	0.70892	0.70869	0.00012
31.44	0.70883	0.70864	0.00012
31.39	0.70870	0.70866	0.00013
31.34	0.70852	0.70866	0.00013
31.28	0.70877	0.70868	0.00013
31.23	0.70847	0.70869	0.00013
31.17	0.70830	0.70870	0.00012
31.12	0.70875	0.70873	0.00009
31.07	0.70879	0.70872	0.00009
31.01	0.70881	0.70872	0.00009
30.96	0.70847	0.70870	0.00009
30.91	0.70898	0.70872	0.00008
30.85	0.70869	0.70867	0.00007
30.80	0.70876	0.70862	0.00011
30.74	0.70883	0.70861	0.00011
30.69	0.70863	0.70858	0.00010
30.64	0.70855	0.70857	0.00010
30.58	0.70869	0.70856	0.00010
30.53	0.70874	0.70856	0.00010
30.47	0.70861	0.70853	0.00010
30.42	0.70868	0.70854	0.00010
30.37	0.70848	0.70852	0.00010
30.31	0.70819	0.70847	0.00015
30.26	0.70868	0.70851	0.00013
30.21	0.70852	0.70849	0.00013
30.15	0.70858	0.70851	0.00014
30.10	0.70842	0.70854	0.00016
30.04	0.70870	0.70853	0.00016
29.99	0.70844	0.70852	0.00015
29.94	0.70867	0.70852	0.00015
29.88	0.70846	0.70849	0.00015
29.83	0.70798	0.70846	0.00017
29.77	0.70860	0.70854	0.00013
29.72	0.70848	0.70849	0.00016
29.67	0.70874	0.70847	0.00016
29.61	0.70887	0.70844	0.00015

29.56	0.70840	0.70843	0.00014
29.51	0.70854	0.70842	0.00014
29.45	0.70851	0.70837	0.00015
29.40	0.70836	0.70837	0.00015
29.34	0.70813	0.70842	0.00017
29.29	0.70873	0.70847	0.00017
29.24	0.70814	0.70848	0.00018
29.18	0.70829	0.70850	0.00016
29.13	0.70846	0.70859	0.00021
29.07	0.70876	0.70863	0.00021
29.02	0.70826	0.70862	0.00021
28.97	0.70810	0.70867	0.00020
28.91	0.70848	0.70872	0.00015
28.86	0.70879	0.70872	0.00016
28.81	0.70871	0.70867	0.00017
28.75	0.70882	0.70868	0.00017
28.70	0.70832	0.70869	0.00018
28.64	0.70921	0.70870	0.00016
28.59	0.70885	0.70865	0.00012
28.54	0.70865	0.70863	0.00011
28.48	0.70879	0.70860	0.00013
28.43	0.70861	0.70857	0.00012
28.37	0.70840	0.70857	0.00012
28.32	0.70837	0.70857	0.00012
28.27	0.70877	0.70859	0.00012
28.21	0.70893	0.70857	0.00011
28.16	0.70847	0.70851	0.00008
28.11	0.70871	0.70852	0.00008
28.05	0.70861	0.70851	0.00007
28.00	0.70834	0.70855	0.00011
27.94	0.70853	0.70861	0.00013
27.89	0.70859	0.70862	0.00013
27.84	0.70834	0.70860	0.00013
27.78	0.70859	0.70862	0.00012
27.73	0.70858	0.70863	0.00012
27.67	0.70838	0.70863	0.00012
27.62	0.70857	0.70868	0.00011
27.57	0.70863	0.70867	0.00012
27.51	0.70891	0.70862	0.00016
27.46	0.70900	0.70860	0.00015
27.41	0.70859	0.70859	0.00013
27.35	0.70843	0.70858	0.00013
27.30	0.70856	0.70859	0.00013
27.24	0.70861	0.70859	0.00013
27.19	0.70866	0.70858	0.00013
27.14	0.70883	0.70855	0.00014
27.08	0.70847	0.70847	0.00015
27.03	0.70816	0.70849	0.00015
26.97	0.70872	0.70857	0.00015
26.92	0.70885	0.70855	0.00015

26.87	0.70855	0.70852	0.00013
26.81	0.70852	0.70851	0.00013
26.76	0.70853	0.70847	0.00015
26.71	0.70855	0.70846	0.00015
26.65	0.70827	0.70845	0.00015
26.60	0.70812	0.70848	0.00014
26.54	0.70866	0.70853	0.00012
26.49	0.70890	0.70854	0.00013
26.44	0.70853	0.70848	0.00010
26.38	0.70860	0.70851	0.00013
26.33	0.70838	0.70850	0.00013
26.27	0.70821	0.70857	0.00018
26.22	0.70842	0.70861	0.00016
26.17	0.70844	0.70862	0.00016
26.11	0.70850	0.70861	0.00017
26.06	0.70863	0.70860	0.00017
26.01	0.70878	0.70856	0.00019
25.95	0.70832	0.70851	0.00019
25.90	0.70885	0.70852	0.00018
25.84	0.70845	0.70848	0.00017
25.79	0.70916	0.70854	0.00020
25.74	0.70855	0.70845	0.00015
25.68	0.70857	0.70847	0.00016
25.63	0.70824	0.70845	0.00016
25.57	0.70846	0.70851	0.00017
25.52	0.70820	0.70856	0.00019
25.47	0.70831	0.70860	0.00017
25.41	0.70842	0.70864	0.00016
25.36	0.70842	0.70866	0.00015
25.31	0.70903	0.70870	0.00015
25.25	0.70829	0.70862	0.00017
25.20	0.70873	0.70864	0.00015
25.14	0.70838	0.70863	0.00015
25.09	0.70890	0.70861	0.00017
25.04	0.70891	0.70856	0.00016
24.98	0.70865	0.70853	0.00014
24.93	0.70863	0.70853	0.00014
24.87	0.70868	0.70851	0.00014
24.82	0.70885	0.70849	0.00014
24.77	0.70815	0.70847	0.00012
24.71	0.70857	0.70849	0.00010
24.66	0.70859	0.70848	0.00010
24.61	0.70817	0.70847	0.00009
24.55	0.70839	0.70851	0.00007
24.50	0.70862	0.70849	0.00009
24.44	0.70865	0.70847	0.00009
24.39	0.70841	0.70843	0.00009
24.34	0.70848	0.70842	0.00009
24.28	0.70866	0.70843	0.00009
24.23	0.70839	0.70844	0.00011

24.12	0.70844	0.70847	0.00012
24.07	0.70852	0.70847	0.00012
24.01	0.70856	0.70852	0.00015
23.96	0.70819	0.70851	0.00015
23.91	0.70845	0.70859	0.00016
23.85	0.70823	0.70858	0.00017
23.74	0.70831	0.70861	0.00015
23.69	0.70851	0.70862	0.00015
23.64	0.70881	0.70859	0.00016
23.58	0.70864	0.70858	0.00016
23.53	0.70853	0.70855	0.00017
23.47	0.70896	0.70857	0.00017
23.42	0.70852	0.70851	0.00016
23.37	0.70896	0.70854	0.00017
23.31	0.70833	0.70851	0.00015
23.26	0.70856	0.70859	0.00018
23.21	0.70833	0.70859	0.00018
23.15	0.70823	0.70862	0.00017
23.10	0.70878	0.70865	0.00015
23.04	0.70831	0.70867	0.00016
22.99	0.70873	0.70869	0.00015
22.94	0.70831	0.70866	0.00015
22.88	0.70882	0.70867	0.00014
22.83	0.70869	0.70867	0.00014
22.77	0.70910	0.70862	0.00017
22.72	0.70862	0.70858	0.00014
22.67	0.70865	0.70856	0.00014
22.61	0.70845	0.70855	0.00014
22.56	0.70897	0.70861	0.00016
22.51	0.70854	0.70858	0.00014
22.45	0.70849	0.70858	0.00014
22.40	0.70841	0.70860	0.00014
22.34	0.70873	0.70860	0.00014
22.29	0.70820	0.70859	0.00014
22.24	0.70876	0.70861	0.00013
22.18	0.70840	0.70856	0.00013
22.13	0.70859	0.70860	0.00012
22.08	0.70897	0.70860	0.00012
22.02	0.70876	0.70856	0.00009
21.97	0.70848	0.70854	0.00007
21.91	0.70866	0.70855	0.00008
21.86	0.70847	0.70851	0.00008
21.81	0.70836	0.70848	0.00009
21.75	0.70839	0.70849	0.00009
21.70	0.70867	0.70851	0.00008
21.64	0.70865	0.70852	0.00009
21.59	0.70857	0.70853	0.00010
21.54	0.70859	0.70853	0.00010
21.48	0.70836	0.70851	0.00011
21.43	0.70849	0.70854	0.00011

21.38	0.70828	0.70854	0.00011
21.32	0.70845	0.70858	0.00010
21.27	0.70854	0.70861	0.00010
21.21	0.70872	0.70859	0.00011
21.16	0.70876	0.70859	0.00012
21.11	0.70855	0.70859	0.00011
21.05	0.70829	0.70859	0.00011
21.00	0.70864	0.70863	0.00010
20.94	0.70873	0.70859	0.00013
20.89	0.70839	0.70856	0.00013
20.84	0.70873	0.70860	0.00013
20.78	0.70871	0.70857	0.00013
20.73	0.70836	0.70857	0.00012
20.68	0.70878	0.70859	0.00011
20.62	0.70870	0.70859	0.00012
20.57	0.70852	0.70860	0.00012
20.51	0.70877	0.70862	0.00012
20.46	0.70821	0.70860	0.00012
20.41	0.70844	0.70864	0.00008
20.35	0.70876	0.70864	0.00008
20.30	0.70846	0.70866	0.00010
20.24	0.70865	0.70867	0.00009
20.19	0.70860	0.70867	0.00009
20.14	0.70880	0.70868	0.00009
20.08	0.70873	0.70863	0.00010
20.03	0.70874	0.70859	0.00011
19.98	0.70857	0.70855	0.00012
19.92	0.70860	0.70853	0.00013
19.87	0.70845	0.70851	0.00013
19.81	0.70895	0.70849	0.00014
19.76	0.70865	0.70845	0.00011
19.71	0.70865	0.70850	0.00017
19.65	0.70864	0.70847	0.00017
19.60	0.70837	0.70848	0.00018
19.54	0.70833	0.70850	0.00018
19.49	0.70833	0.70854	0.00017
19.44	0.70830	0.70851	0.00020
19.38	0.70847	0.70857	0.00021
19.33	0.70821	0.70856	0.00021
19.28	0.70860	0.70859	0.00020
19.22	0.70914	0.70859	0.00020
19.17	0.70833	0.70854	0.00016
19.11	0.70873	0.70860	0.00017
19.06	0.70860	0.70856	0.00017
19.01	0.70870	0.70857	0.00017
18.95	0.70805	0.70854	0.00017
18.90	0.70892	0.70861	0.00014
18.84	0.70833	0.70860	0.00013
18.79	0.70848	0.70862	0.00011
18.74	0.70859	0.70863	0.00011

18.68	0.70866	0.70862	0.00011
18.63	0.70895	0.70862	0.00011
18.58	0.70836	0.70861	0.00011
18.52	0.70863	0.70863	0.00009
18.47	0.70845	0.70864	0.00009
18.41	0.70878	0.70866	0.00008
18.36	0.70879	0.70865	0.00008
18.31	0.70849	0.70865	0.00008
18.25	0.70861	0.70867	0.00007
18.20	0.70849	0.70866	0.00007
18.14	0.70864	0.70868	0.00006
18.09	0.70888	0.70866	0.00008
18.04	0.70856	0.70862	0.00006
17.98	0.70870	0.70865	0.00007
17.93	0.70866	0.70865	0.00007
17.88	0.70871	0.70866	0.00008
17.82	0.70876	0.70862	0.00011
17.77	0.70873	0.70857	0.00012
17.71	0.70852	0.70859	0.00013
17.66	0.70861	0.70860	0.00013
17.61	0.70845	0.70860	0.00013
17.55	0.70854	0.70860	0.00013
17.50	0.70878	0.70858	0.00014
17.44	0.70870	0.70854	0.00014
17.39	0.70878	0.70851	0.00013
17.34	0.70828	0.70851	0.00013
17.28	0.70832	0.70854	0.00012
17.23	0.70890	0.70856	0.00011
17.18	0.70865	0.70854	0.00008
17.12	0.70861	0.70855	0.00009
17.07	0.70842	0.70852	0.00010
17.01	0.70840	0.70855	0.00010
16.96	0.70836	0.70859	0.00011
16.91	0.70842	0.70860	0.00010
16.85	0.70872	0.70860	0.00010
16.80	0.70859	0.70855	0.00012
16.74	0.70857	0.70857	0.00012
16.69	0.70868	0.70856	0.00012
16.64	0.70875	0.70856	0.00012
16.58	0.70831	0.70854	0.00011
16.53	0.70868	0.70855	0.00011
16.48	0.70879	0.70854	0.00010
16.42	0.70850	0.70850	0.00009
16.37	0.70844	0.70852	0.00010
16.31	0.70822	0.70851	0.00010
16.26	0.70873	0.70856	0.00009
16.21	0.70855	0.70859	0.00012
16.15	0.70861	0.70860	0.00012
16.10	0.70854	0.70863	0.00013
16.04	0.70839	0.70865	0.00013

15.99	0.70863	0.70866	0.00013
15.94	0.70839	0.70866	0.00013
15.88	0.70867	0.70868	0.00012
15.83	0.70836	0.70867	0.00012
15.78	0.70873	0.70868	0.00011
15.72	0.70900	0.70871	0.00013
15.67	0.70871	0.70867	0.00011
15.61	0.70887	0.70868	0.00011
15.56	0.70873	0.70866	0.00011
15.51	0.70852	0.70869	0.00014
15.45	0.70866	0.70866	0.00017
15.40	0.70853	0.70865	0.00017
15.34	0.70860	0.70864	0.00017
15.29	0.70845	0.70866	0.00017
15.24	0.70904	0.70867	0.00017
15.18	0.70861	0.70859	0.00016
15.13	0.70877	0.70861	0.00016
15.08	0.70866	0.70859	0.00016
15.02	0.70908	0.70859	0.00016
14.97	0.70818	0.70851	0.00013
14.91	0.70856	0.70853	0.00011
14.86	0.70849	0.70849	0.00013
14.81	0.70872	0.70853	0.00015
14.75	0.70855	0.70855	0.00017
14.70	0.70829	0.70854	0.00017
14.64	0.70879	0.70854	0.00017
14.59	0.70854	0.70853	0.00017
14.54	0.70874	0.70853	0.00017
14.48	0.70828	0.70850	0.00016
14.43	0.70839	0.70852	0.00015
14.38	0.70816	0.70859	0.00018
14.32	0.70883	0.70865	0.00016
14.27	0.70892	0.70866	0.00016
14.21	0.70849	0.70860	0.00016
14.16	0.70823	0.70860	0.00016
14.11	0.70869	0.70866	0.00014
14.05	0.70853	0.70864	0.00015
14.00	0.70849	0.70866	0.00015
13.94	0.70847	0.70861	0.00019
13.89	0.70907	0.70863	0.00019
13.84	0.70873	0.70857	0.00016
13.78	0.70892	0.70853	0.00016
13.73	0.70841	0.70850	0.00014
13.68	0.70845	0.70851	0.00014
13.62	0.70882	0.70855	0.00015
13.57	0.70848	0.70852	0.00014
13.51	0.70872	0.70854	0.00014
13.46	0.70804	0.70855	0.00015
13.41	0.70861	0.70865	0.00013
13.35	0.70855	0.70863	0.00014

13.30	0.70831	0.70862	0.00014
13.25	0.70864	0.70861	0.00016
13.19	0.70853	0.70858	0.00016
13.14	0.70881	0.70859	0.00016
13.08	0.70852	0.70857	0.00015
13.03	0.70866	0.70856	0.00016
12.98	0.70880	0.70854	0.00015
12.92	0.70907	0.70853	0.00015
12.87	0.70839	0.70849	0.00009
12.81	0.70844	0.70854	0.00012
12.76	0.70821	0.70859	0.00014
12.71	0.70839	0.70861	0.00012
12.65	0.70862	0.70863	0.00011
12.60	0.70856	0.70865	0.00012
12.55	0.70843	0.70867	0.00012
12.49	0.70854	0.70869	0.00010
12.44	0.70865	0.70868	0.00011
12.38	0.70867	0.70868	0.00011
12.33	0.70893	0.70866	0.00012
12.28	0.70891	0.70860	0.00013
12.22	0.70845	0.70860	0.00013
12.17	0.70851	0.70860	0.00013
12.11	0.70881	0.70859	0.00014
12.06	0.70877	0.70856	0.00013
12.01	0.70866	0.70859	0.00016
11.95	0.70840	0.70857	0.00016
11.90	0.70867	0.70860	0.00015
11.85	0.70852	0.70863	0.00017
11.79	0.70824	0.70863	0.00017
11.74	0.70895	0.70869	0.00015
11.68	0.70845	0.70866	0.00014
11.63	0.70844	0.70867	0.00014
11.58	0.70848	0.70866	0.00015
11.52	0.70903	0.70869	0.00015
11.47	0.70849	0.70859	0.00017
11.41	0.70869	0.70859	0.00017
11.36	0.70898	0.70861	0.00018
11.31	0.70851	0.70858	0.00016
11.25	0.70884	0.70859	0.00016
11.20	0.70870	0.70860	0.00016
11.15	0.70851	0.70861	0.00017
11.09	0.70831	0.70863	0.00017
11.04	0.70879	0.70872	0.00018
10.98	0.70810	0.70868	0.00019
10.93	0.70845	0.70873	0.00014
10.88	0.70889	0.70879	0.00014
10.82	0.70870	0.70878	0.00013
10.77	0.70862	0.70877	0.00014
10.71	0.70894	0.70874	0.00016
10.66	0.70876	0.70868	0.00018

10.61	0.70876	0.70871	0.00020
10.55	0.70914	0.70867	0.00021
10.50	0.70845	0.70860	0.00018
10.45	0.70860	0.70861	0.00018
10.39	0.70905	0.70859	0.00018
10.34	0.70875	0.70858	0.00017
10.28	0.70863	0.70857	0.00017
10.23	0.70830	0.70861	0.00019
10.18	0.70832	0.70865	0.00018
10.12	0.70909	0.70869	0.00016
10.07	0.70834	0.70868	0.00016
10.01	0.70848	0.70871	0.00014
9.96	0.70856	0.70872	0.00013
9.91	0.70841	0.70873	0.00013
9.85	0.70893	0.70873	0.00013
9.80	0.70867	0.70866	0.00014
9.75	0.70899	0.70865	0.00015
9.69	0.70868	0.70863	0.00013
9.64	0.70869	0.70862	0.00013
9.58	0.70905	0.70862	0.00013
9.53	0.70868	0.70856	0.00010
9.48	0.70852	0.70856	0.00010
9.42	0.70862	0.70860	0.00013
9.37	0.70842	0.70863	0.00014
9.31	0.70830	0.70864	0.00014
9.26	0.70854	0.70871	0.00012
9.21	0.70879	0.70871	0.00012
9.15	0.70856	0.70869	0.00012
9.10	0.70875	0.70874	0.00014
9.05	0.70839	0.70874	0.00014
8.99	0.70869	0.70876	0.00013
8.94	0.70896	0.70870	0.00019
8.88	0.70891	0.70865	0.00018
8.83	0.70854	0.70860	0.00018
8.78	0.70894	0.70864	0.00019
8.72	0.70857	0.70860	0.00018
8.67	0.70859	0.70861	0.00018
8.61	0.70910	0.70865	0.00019
8.56	0.70871	0.70863	0.00018
8.51	0.70859	0.70858	0.00019
8.45	0.70806	0.70858	0.00019
8.40	0.70852	0.70864	0.00016
8.35	0.70838	0.70867	0.00016
8.29	0.70893	0.70868	0.00015
8.24	0.70854	0.70866	0.00014
8.18	0.70873	0.70870	0.00015
8.13	0.70898	0.70870	0.00015
8.08	0.70889	0.70866	0.00013
8.02	0.70821	0.70865	0.00013
7.97	0.70856	0.70871	0.00008

7.91	0.70871	0.70874	0.00008
7.86	0.70875	0.70872	0.00010
7.81	0.70852	0.70869	0.00011
7.75	0.70871	0.70869	0.00012
7.70	0.70897	0.70867	0.00012
7.65	0.70870	0.70865	0.00010
7.59	0.70858	0.70865	0.00010
7.54	0.70878	0.70862	0.00011
7.48	0.70883	0.70865	0.00014
7.43	0.70888	0.70862	0.00013
7.38	0.70845	0.70859	0.00012
7.32	0.70852	0.70859	0.00012
7.27	0.70844	0.70860	0.00012
7.21	0.70857	0.70861	0.00012
7.16	0.70877	0.70864	0.00013
7.11	0.70864	0.70863	0.00012
7.05	0.70836	0.70861	0.00013
7.00	0.70903	0.70862	0.00012
6.95	0.70855	0.70856	0.00009
6.89	0.70861	0.70857	0.00009
6.84	0.70841	0.70858	0.00010
6.78	0.70861	0.70860	0.00009
6.73	0.70856	0.70858	0.00009
6.68	0.70883	0.70860	0.00010
6.62	0.70868	0.70857	0.00008
6.57	0.70848	0.70854	0.00008
6.51	0.70842	0.70859	0.00013
6.46	0.70842	0.70860	0.00012
6.41	0.70867	0.70864	0.00012
6.35	0.70876	0.70867	0.00013
6.30	0.70858	0.70864	0.00013
6.25	0.70843	0.70866	0.00013
6.19	0.70869	0.70868	0.00012
6.14	0.70852	0.70871	0.00014
6.08	0.70839	0.70879	0.00018
6.03	0.70901	0.70884	0.00015
5.98	0.70853	0.70884	0.00015
5.92	0.70884	0.70887	0.00014
5.87	0.70890	0.70886	0.00014
5.81	0.70852	0.70884	0.00014
5.76	0.70871	0.70885	0.00013
5.71	0.70869	0.70879	0.00019
5.65	0.70897	0.70878	0.00020
5.60	0.70931	0.70877	0.00019
5.55	0.70895	0.70871	0.00015
5.49	0.70900	0.70871	0.00016
5.44	0.70876	0.70865	0.00015
5.38	0.70877	0.70863	0.00015
5.33	0.70869	0.70860	0.00015
5.28	0.70864	0.70859	0.00015

5.22	0.70816	0.70861	0.00016
5.17	0.70854	0.70864	0.00013
5.11	0.70891	0.70862	0.00014
5.06	0.70864	0.70858	0.00012
5.01	0.70898	0.70857	0.00013
4.95	0.70838	0.70851	0.00009
4.90	0.70858	0.70854	0.00009
4.74	0.70847	0.70853	0.00009
4.68	0.70859	0.70852	0.00009
4.63	0.70884	0.70853	0.00009
4.58	0.70849	0.70849	0.00006
4.52	0.70835	0.70846	0.00008
4.47	0.70852	0.70852	0.00012
4.42	0.70850	0.70857	0.00015
4.36	0.70842	0.70861	0.00017
4.31	0.70867	0.70865	0.00016
4.25	0.70841	0.70864	0.00016
4.20	0.70844	0.70865	0.00016
4.15	0.70864	0.70865	0.00016
4.09	0.70845	0.70868	0.00017
4.04	0.70824	0.70870	0.00017
3.98	0.70896	0.70874	0.00013
3.93	0.70898	0.70870	0.00013
3.88	0.70890	0.70868	0.00012
3.82	0.70880	0.70863	0.00012
3.77	0.70855	0.70866	0.00014
3.72	0.70857	0.70869	0.00015
3.66	0.70838	0.70875	0.00017
3.61	0.70897	0.70875	0.00018
3.55	0.70861	0.70870	0.00018
3.50	0.70869	0.70867	0.00019
3.45	0.70856	0.70868	0.00019
3.39	0.70880	0.70865	0.00021
3.34	0.70841	0.70862	0.00021
3.28	0.70907	0.70862	0.00021
3.23	0.70884	0.70857	0.00019
3.18	0.70918	0.70853	0.00018
3.12	0.70831	0.70849	0.00012
3.07	0.70849	0.70850	0.00011
3.02	0.70833	0.70848	0.00012
2.96	0.70877	0.70851	0.00011
2.91	0.70826	0.70851	0.00011
2.85	0.70856	0.70853	0.00010
2.80	0.70834	0.70855	0.00011
2.75	0.70864	0.70860	0.00012
2.69	0.70847	0.70857	0.00013
2.64	0.70873	0.70860	0.00013
2.58	0.70840	0.70858	0.00013
2.53	0.70832	0.70862	0.00013
2.48	0.70863	0.70867	0.00012

2.42	0.70877	0.70867	0.00012
2.37	0.70844	0.70868	0.00012
2.32	0.70872	0.70873	0.00012
2.26	0.70892	0.70873	0.00012
2.21	0.70834	0.70870	0.00011
2.15	0.70875	0.70872	0.00009
2.10	0.70851	0.70868	0.00012
2.05	0.70882	0.70868	0.00012
1.99	0.70881	0.70869	0.00012
1.94	0.70865	0.70870	0.00013
1.88	0.70888	0.70870	0.00013
1.83	0.70894	0.70867	0.00013
1.78	0.70869	0.70865	0.00011
1.72	0.70863	0.70863	0.00012
1.67	0.70851	0.70863	0.00012
1.62	0.70833	0.70863	0.00012
1.56	0.70860	0.70866	0.00010
1.51	0.70883	0.70866	0.00010
1.45	0.70898	0.70864	0.00009
1.40	0.70866	0.70858	0.00007
1.35	0.70855	0.70856	0.00007
1.29	0.70876	0.70857	0.00008
1.24	0.70844	0.70854	0.00006
1.18	0.70863	0.70855	0.00006
1.13	0.70850	0.70856	0.00008
1.08	0.70867	0.70855	0.00009
1.02	0.70861	0.70854	0.00009
0.97	0.70858	0.70859	0.00015
0.92	0.70836	0.70857	0.00015
0.86	0.70849	0.70860	0.00015
0.81	0.70864	0.70860	0.00015
0.75	0.70853	0.70858	0.00015
0.70	0.70847	0.70852	0.00020
0.65	0.70879	0.70854	0.00020
0.59	0.70832	0.70860	0.00025
0.54	0.70861	0.70863	0.00024
0.48	0.70913	0.70863	0.00026
0.43	0.70840	0.70857	0.00024
0.38	0.70865	0.70859	0.00026
0.32	0.70843	0.70858	0.00028
0.27	0.70846	0.70861	0.00031
0.22	0.70795	0.70865	0.00036
0.16	0.70869	0.70888	0.00025
0.11	0.70933	0.70897	0.00032
0.05	0.70861		

ARB 22.2.1 (M2)

Distance from cervix (mm)	$^{87}Sr/^{86}Sr$	10 point mov. average	2 SE on mov. average
37.31	0.70928	0.70909	0.00033
37.27	0.70916	0.70904	0.00033
37.23	0.70893	0.70901	0.00033
37.19	0.70959	0.70894	0.00036
37.14	0.70906	0.70887	0.00033
37.10	0.70982	0.70900	0.00044
37.06	0.70932	0.70897	0.00043
37.01	0.70927	0.70895	0.00042
36.97	0.70837	0.70894	0.00042
36.93	0.70812	0.70901	0.00040
36.89	0.70877	0.70905	0.00037
36.84	0.70888	0.70909	0.00036
36.80	0.70823	0.70905	0.00038
36.76	0.70888	0.70907	0.00036
36.72	0.71033	0.70906	0.00036
36.67	0.70955	0.70895	0.00024
36.63	0.70909	0.70899	0.00029
36.59	0.70919	0.70894	0.00030
36.54	0.70910	0.70894	0.00030
36.50	0.70847	0.70901	0.00034
36.46	0.70914	0.70907	0.00032
36.42	0.70848	0.70903	0.00033
36.37	0.70849	0.70903	0.00033
36.33	0.70876	0.70911	0.00031
36.29	0.70921	0.70915	0.00030
36.25	0.70995	0.70913	0.00030
36.20	0.70858	0.70902	0.00024
36.16	0.70920	0.70904	0.00023
36.12	0.70978	0.70902	0.00023
36.08	0.70908	0.70893	0.00015
36.03	0.70873	0.70900	0.00024
35.99	0.70849	0.70904	0.00023
35.95	0.70930	0.70909	0.00019
35.90	0.70919	0.70907	0.00019
35.86	0.70900	0.70911	0.00022
35.82	0.70880	0.70916	0.00023
35.78	0.70878	0.70919	0.00021
35.73	0.70904	0.70931	0.00024
35.69	0.70886	0.70934	0.00023
35.65	0.70983	0.70936	0.00022
35.61	0.70912	0.70929	0.00019
35.56	0.70899	0.70926	0.00021
35.52	0.70905	0.70928	0.00021
35.48	0.70961	0.70928	0.00020
35.43	0.70947	0.70926	0.00019
35.39	0.70918	0.70918	0.00022
35.35	0.70994	0.70925	0.00026

35.31	0.70932	0.70919	0.00021
35.26	0.70906	0.70916	0.00022
35.22	0.70917	0.70923	0.00024
35.18	0.70882	0.70926	0.00025
35.14	0.70911	0.70937	0.00025
35.09	0.70914	0.70943	0.00025
35.05	0.70938	0.70945	0.00025
35.01	0.70865	0.70945	0.00025
34.97	0.70990	0.70957	0.00018
34.92	0.70938	0.70947	0.00021
34.88	0.70901	0.70943	0.00023
34.84	0.70972	0.70950	0.00022
34.79	0.70951	0.70943	0.00024
34.75	0.70987	0.70946	0.00025
34.71	0.70970	0.70937	0.00024
34.67	0.70941	0.70938	0.00025
34.62	0.70931	0.70937	0.00025
34.58	0.70987	0.70932	0.00027
34.54	0.70890	0.70925	0.00024
34.50	0.70896	0.70931	0.00023
34.45	0.70977	0.70929	0.00024
34.41	0.70902	0.70921	0.00023
34.37	0.70979	0.70918	0.00024
34.32	0.70899	0.70912	0.00020
34.28	0.70977	0.70910	0.00020
34.24	0.70927	0.70904	0.00014
34.20	0.70886	0.70905	0.00015
34.15	0.70919	0.70906	0.00014
34.11	0.70944	0.70901	0.00016
34.07	0.70880	0.70895	0.00012
34.03	0.70893	0.70894	0.00013
33.98	0.70877	0.70892	0.00014
33.94	0.70914	0.70896	0.00015
33.90	0.70889	0.70898	0.00016
33.86	0.70912	0.70899	0.00016
33.81	0.70932	0.70899	0.00016
33.77	0.70900	0.70900	0.00017
33.73	0.70868	0.70900	0.00017
33.68	0.70887	0.70904	0.00015
33.64	0.70871	0.70911	0.00018
33.60	0.70868	0.70913	0.00017
33.56	0.70923	0.70914	0.00016
33.51	0.70927	0.70912	0.00015
33.47	0.70904	0.70911	0.00015
33.43	0.70908	0.70913	0.00015
33.39	0.70945	0.70912	0.00016
33.34	0.70900	0.70907	0.00014
33.30	0.70907	0.70907	0.00014
33.26	0.70959	0.70907	0.00015
33.21	0.70885	0.70894	0.00014

33.17	0.70883	0.70895	0.00014
33.13	0.70908	0.70898	0.00014
33.09	0.70909	0.70895	0.00014
33.04	0.70927	0.70888	0.00017
33.00	0.70897	0.70881	0.00014
32.96	0.70893	0.70877	0.00014
32.92	0.70899	0.70881	0.00018
32.83	0.70846	0.70881	0.00017
32.79	0.70893	0.70885	0.00015
32.75	0.70907	0.70889	0.00017
32.70	0.70881	0.70889	0.00017
32.66	0.70847	0.70892	0.00018
32.62	0.70865	0.70896	0.00015
32.57	0.70861	0.70899	0.00014
32.53	0.70933	0.70899	0.00013
32.49	0.70894	0.70892	0.00012
32.45	0.70882	0.70891	0.00012
32.40	0.70891	0.70893	0.00013
32.36	0.70925	0.70898	0.00015
32.32	0.70908	0.70894	0.00014
32.28	0.70915	0.70893	0.00013
32.23	0.70889	0.70886	0.00016
32.19	0.70889	0.70891	0.00019
32.15	0.70865	0.70889	0.00019
32.10	0.70866	0.70888	0.00020
32.06	0.70882	0.70890	0.00020
32.02	0.70903	0.70888	0.00020
31.98	0.70933	0.70887	0.00020
31.93	0.70886	0.70878	0.00018
31.89	0.70899	0.70878	0.00018
31.85	0.70843	0.70880	0.00020
31.81	0.70940	0.70883	0.00018
31.76	0.70873	0.70872	0.00016
31.72	0.70851	0.70872	0.00016
31.68	0.70886	0.70872	0.00016
31.64	0.70864	0.70870	0.00016
31.59	0.70890	0.70873	0.00016
31.55	0.70849	0.70864	0.00021
31.51	0.70884	0.70873	0.00024
31.46	0.70919	0.70867	0.00026
31.42	0.70871	0.70861	0.00023
31.38	0.70832	0.70859	0.00023
31.34	0.70877	0.70863	0.00022
31.29	0.70848	0.70868	0.00025
31.25	0.70871	0.70873	0.00025
31.21	0.70889	0.70872	0.00025
31.17	0.70803	0.70870	0.00025
31.12	0.70932	0.70881	0.00021
31.08	0.70829	0.70878	0.00019
31.04	0.70861	0.70885	0.00015

30.99	0.70848	0.70886	0.00015
30.95	0.70873	0.70891	0.00012
30.91	0.70923	0.70890	0.00013
30.87	0.70897	0.70889	0.00013
30.82	0.70871	0.70887	0.00012
30.78	0.70867	0.70889	0.00012
30.74	0.70908	0.70896	0.00013
30.70	0.70907	0.70900	0.00017
30.65	0.70898	0.70895	0.00018
30.61	0.70872	0.70890	0.00021
30.57	0.70895	0.70894	0.00021
30.53	0.70858	0.70897	0.00021
30.48	0.70913	0.70901	0.00019
30.44	0.70881	0.70893	0.00023
30.40	0.70894	0.70897	0.00024
30.35	0.70930	0.70892	0.00027
30.31	0.70949	0.70890	0.00026
30.27	0.70864	0.70886	0.00023
30.23	0.70848	0.70886	0.00023
30.18	0.70912	0.70889	0.00022
30.14	0.70922	0.70888	0.00021
30.10	0.70894	0.70887	0.00021
30.06	0.70836	0.70888	0.00021
30.01	0.70925	0.70887	0.00023
29.97	0.70838	0.70878	0.00023
29.93	0.70910	0.70890	0.00025
29.88	0.70917	0.70887	0.00025
29.84	0.70858	0.70885	0.00024
29.80	0.70884	0.70896	0.00029
29.76	0.70892	0.70891	0.00032
29.71	0.70916	0.70888	0.00032
29.67	0.70905	0.70884	0.00031
29.63	0.70823	0.70881	0.00031
29.59	0.70837	0.70892	0.00029
29.54	0.70954	0.70904	0.00029
29.50	0.70887	0.70893	0.00029
29.46	0.70888	0.70888	0.00032
29.42	0.70972	0.70888	0.00032
29.37	0.70831	0.70888	0.00031
29.33	0.70866	0.70897	0.00029
29.29	0.70881	0.70895	0.00030
29.24	0.70873	0.70892	0.00031
29.20	0.70928	0.70894	0.00031
29.16	0.70960	0.70895	0.00031
29.12	0.70843	0.70888	0.00029
29.07	0.70837	0.70888	0.00029
29.03	0.70893	0.70896	0.00026
28.99	0.70965	0.70891	0.00028
28.95	0.70923	0.70878	0.00023
28.90	0.70850	0.70875	0.00021

28.86	0.70848	0.70878	0.00020
28.82	0.70898	0.70880	0.00019
28.77	0.70932	0.70890	0.00030
28.69	0.70847	0.70887	0.00029
28.65	0.70903	0.70889	0.00028
28.60	0.70848	0.70882	0.00030
28.56	0.70851	0.70886	0.00029
28.52	0.70900	0.70891	0.00028
28.48	0.70873	0.70892	0.00028
28.43	0.70864	0.70898	0.00029
28.39	0.70991	0.70903	0.00028
28.35	0.70862	0.70894	0.00020
28.31	0.70929	0.70896	0.00019
28.26	0.70873	0.70896	0.00019
28.22	0.70829	0.70899	0.00018
28.18	0.70884	0.70905	0.00010
28.13	0.70907	0.70906	0.00010
28.09	0.70911	0.70904	0.00010
28.05	0.70924	0.70902	0.00011
28.01	0.70919	0.70901	0.00010
27.96	0.70899	0.70898	0.00009
27.92	0.70882	0.70897	0.00009
27.88	0.70930	0.70896	0.00011
27.84	0.70902	0.70889	0.00010
27.79	0.70896	0.70886	0.00010
27.75	0.70889	0.70889	0.00013
27.71	0.70889	0.70885	0.00015
27.66	0.70890	0.70889	0.00018
27.62	0.70913	0.70890	0.00018
27.58	0.70890	0.70886	0.00017
27.54	0.70894	0.70883	0.00018
27.49	0.70867	0.70880	0.00019
27.45	0.70861	0.70884	0.00019
27.41	0.70868	0.70885	0.00019
27.37	0.70928	0.70886	0.00019
27.32	0.70850	0.70883	0.00017
27.28	0.70931	0.70885	0.00016
27.24	0.70893	0.70881	0.00012
27.20	0.70883	0.70878	0.00012
27.15	0.70851	0.70878	0.00012
27.11	0.70864	0.70884	0.00012
27.07	0.70911	0.70882	0.00013
27.02	0.70873	0.70881	0.00012
26.98	0.70873	0.70882	0.00012
26.94	0.70904	0.70882	0.00012
26.90	0.70865	0.70878	0.00011
26.85	0.70892	0.70879	0.00011
26.81	0.70861	0.70879	0.00011
26.77	0.70890	0.70878	0.00011
26.73	0.70904	0.70879	0.00012

26.68	0.70847	0.70875	0.00011
26.64	0.70897	0.70879	0.00010
26.60	0.70882	0.70881	0.00011
26.55	0.70877	0.70877	0.00014
26.51	0.70864	0.70884	0.00021
26.47	0.70879	0.70888	0.00020
26.43	0.70886	0.70887	0.00021
26.38	0.70855	0.70882	0.00023
26.34	0.70903	0.70885	0.00022
26.30	0.70862	0.70889	0.00025
26.26	0.70885	0.70897	0.00026
26.21	0.70914	0.70894	0.00027
26.17	0.70842	0.70886	0.00029
26.13	0.70953	0.70889	0.00028
26.09	0.70898	0.70884	0.00024
26.04	0.70870	0.70882	0.00024
26.00	0.70833	0.70882	0.00024
25.96	0.70894	0.70889	0.00022
25.91	0.70943	0.70895	0.00026
25.87	0.70935	0.70891	0.00024
25.83	0.70853	0.70886	0.00022
25.79	0.70836	0.70889	0.00021
25.74	0.70871	0.70895	0.00017
25.70	0.70910	0.70902	0.00018
25.66	0.70871	0.70903	0.00018
25.62	0.70869	0.70896	0.00027
25.57	0.70905	0.70898	0.00026
25.53	0.70956	0.70895	0.00027
25.49	0.70907	0.70890	0.00023
25.44	0.70878	0.70891	0.00023
25.40	0.70890	0.70882	0.00031
25.36	0.70895	0.70881	0.00031
25.32	0.70939	0.70878	0.00031
25.27	0.70918	0.70874	0.00029
25.23	0.70802	0.70872	0.00027
25.19	0.70885	0.70881	0.00023
25.15	0.70878	0.70880	0.00023
25.10	0.70911	0.70877	0.00024
25.06	0.70913	0.70873	0.00023
25.02	0.70790	0.70881	0.00033
24.98	0.70874	0.70891	0.00026
24.93	0.70866	0.70892	0.00026
24.89	0.70906	0.70897	0.00026
24.85	0.70891	0.70890	0.00028
24.80	0.70901	0.70889	0.00028
24.76	0.70870	0.70887	0.00028
24.72	0.70846	0.70891	0.00028
24.68	0.70869	0.70899	0.00027
24.63	0.70997	0.70898	0.00028
24.59	0.70889	0.70886	0.00017

24.55	0.70883	0.70888	0.00018
24.51	0.70918	0.70889	0.00018
24.46	0.70838	0.70885	0.00017
24.42	0.70879	0.70893	0.00014
24.38	0.70885	0.70893	0.00014
24.33	0.70904	0.70890	0.00017
24.29	0.70926	0.70888	0.00016
24.25	0.70855	0.70884	0.00014
24.21	0.70879	0.70885	0.00013
24.16	0.70914	0.70888	0.00014
24.12	0.70893	0.70884	0.00013
24.08	0.70872	0.70885	0.00014
24.04	0.70918	0.70880	0.00019
23.99	0.70883	0.70881	0.00020
23.95	0.70851	0.70880	0.00020
23.91	0.70893	0.70883	0.00019
23.87	0.70883	0.70878	0.00020
23.82	0.70863	0.70869	0.00027
23.78	0.70911	0.70871	0.00027
23.74	0.70870	0.70869	0.00026
23.69	0.70908	0.70879	0.00032
23.65	0.70821	0.70878	0.00032
23.61	0.70930	0.70883	0.00030
23.57	0.70874	0.70882	0.00029
23.52	0.70874	0.70882	0.00031
23.48	0.70849	0.70876	0.00034
23.44	0.70790	0.70884	0.00034
23.40	0.70885	0.70893	0.00027
23.35	0.70890	0.70893	0.00026
23.31	0.70967	0.70901	0.00030
23.27	0.70899	0.70895	0.00026
23.22	0.70867	0.70894	0.00026
23.18	0.70921	0.70893	0.00026
23.10	0.70816	0.70893	0.00029
23.05	0.70920	0.70905	0.00025
23.01	0.70871	0.70900	0.00025
22.97	0.70889	0.70902	0.00024
22.93	0.70960	0.70903	0.00024
22.88	0.70908	0.70893	0.00022
22.84	0.70894	0.70899	0.00027
22.80	0.70859	0.70898	0.00027
22.76	0.70854	0.70901	0.00026
22.71	0.70962	0.70903	0.00025
22.67	0.70933	0.70895	0.00021
22.63	0.70871	0.70895	0.00021
22.58	0.70893	0.70897	0.00020
22.54	0.70901	0.70889	0.00025
22.50	0.70858	0.70888	0.00025
22.46	0.70969	0.70887	0.00025
22.41	0.70876	0.70878	0.00017

22.37	0.70894	0.70877	0.00018
22.33	0.70873	0.70873	0.00017
22.29	0.70884	0.70870	0.00019
22.24	0.70929	0.70865	0.00020
22.20	0.70888	0.70865	0.00020
22.16	0.70822	0.70865	0.00020
22.11	0.70885	0.70873	0.00019
22.07	0.70854	0.70872	0.00019
22.03	0.70873	0.70872	0.00019
21.99	0.70865	0.70878	0.00022
21.94	0.70861	0.70876	0.00023
21.90	0.70840	0.70886	0.00028
21.86	0.70831	0.70887	0.00027
21.82	0.70928	0.70892	0.00024
21.77	0.70896	0.70894	0.00026
21.73	0.70900	0.70891	0.00026
21.69	0.70875	0.70899	0.00032
21.65	0.70855	0.70903	0.00032
21.60	0.70934	0.70906	0.00030
21.56	0.70846	0.70901	0.00030
21.52	0.70957	0.70908	0.00028
21.47	0.70852	0.70899	0.00027
21.43	0.70873	0.70905	0.00025
21.39	0.70949	0.70913	0.00026
21.35	0.70867	0.70909	0.00024
21.30	0.70984	0.70915	0.00023
21.26	0.70910	0.70904	0.00019
21.22	0.70883	0.70898	0.00021
21.18	0.70884	0.70900	0.00021
21.13	0.70925	0.70904	0.00021
21.09	0.70858	0.70902	0.00021
21.05	0.70919	0.70906	0.00018
21.00	0.70952	0.70902	0.00019
20.96	0.70911	0.70903	0.00019
20.92	0.70928	0.70907	0.00022
20.88	0.70870	0.70907	0.00022
20.83	0.70850	0.70912	0.00020
20.79	0.70902	0.70921	0.00015
20.75	0.70922	0.70927	0.00017
20.71	0.70909	0.70920	0.00022
20.66	0.70899	0.70918	0.00023
20.62	0.70879	0.70926	0.00026
20.58	0.70956	0.70926	0.00026
20.54	0.70952	0.70918	0.00027
20.49	0.70926	0.70918	0.00027
20.45	0.70923	0.70912	0.00028
20.41	0.70940	0.70911	0.00028
20.36	0.70962	0.70906	0.00028
20.32	0.70855	0.70900	0.00025
20.28	0.70887	0.70900	0.00025

20.24	0.70980	0.70900	0.00025
20.19	0.70876	0.70892	0.00018
20.15	0.70876	0.70891	0.00018
20.11	0.70952	0.70892	0.00018
20.07	0.70873	0.70885	0.00012
20.02	0.70912	0.70886	0.00011
19.98	0.70882	0.70880	0.00011
19.94	0.70908	0.70881	0.00012
19.89	0.70853	0.70880	0.00011
19.85	0.70885	0.70884	0.00009
19.81	0.70899	0.70881	0.00011
19.77	0.70865	0.70879	0.00010
19.72	0.70887	0.70884	0.00012
19.68	0.70884	0.70888	0.00014
19.64	0.70882	0.70894	0.00018
19.60	0.70854	0.70900	0.00020
19.55	0.70893	0.70911	0.00021
19.51	0.70900	0.70912	0.00020
19.47	0.70890	0.70909	0.00022
19.43	0.70855	0.70908	0.00022
19.38	0.70883	0.70918	0.00020
19.34	0.70915	0.70922	0.00019
19.30	0.70922	0.70921	0.00019
19.25	0.70942	0.70921	0.00019
19.21	0.70946	0.70915	0.00020
19.17	0.70960	0.70910	0.00019
19.13	0.70907	0.70904	0.00016
19.08	0.70872	0.70901	0.00017
19.04	0.70880	0.70905	0.00015
19.00	0.70950	0.70909	0.00014
18.96	0.70930	0.70909	0.00015
18.91	0.70903	0.70911	0.00016
18.87	0.70920	0.70911	0.00016
18.83	0.70883	0.70907	0.00017
18.78	0.70892	0.70909	0.00016
18.70	0.70883	0.70912	0.00016
18.66	0.70903	0.70916	0.00015
18.61	0.70913	0.70920	0.00015
18.57	0.70958	0.70921	0.00015
18.53	0.70941	0.70923	0.00018
18.49	0.70907	0.70927	0.00021
18.44	0.70881	0.70928	0.00020
18.40	0.70909	0.70943	0.00021
18.36	0.70915	0.70937	0.00027
18.23	0.70936	0.70927	0.00029
18.19	0.70922	0.70922	0.00030
18.14	0.70976	0.70924	0.00030
18.10	0.70970	0.70921	0.00028
18.06	0.70915	0.70916	0.00026
18.02	0.70997	0.70918	0.00027

17.97	0.70866	0.70912	0.00021
17.93	0.70929	0.70909	0.00024
17.89	0.70898	0.70909	0.00024
17.85	0.70860	0.70913	0.00025
17.80	0.70882	0.70917	0.00022
17.76	0.70949	0.70922	0.00020
17.72	0.70941	0.70917	0.00020
17.67	0.70928	0.70914	0.00019
17.63	0.70934	0.70916	0.00019
17.59	0.70932	0.70918	0.00021
17.55	0.70836	0.70918	0.00021
17.50	0.70932	0.70923	0.00013
17.46	0.70934	0.70922	0.00013
17.42	0.70905	0.70919	0.00013
17.38	0.70924	0.70921	0.00013
17.33	0.70907	0.70922	0.00013
17.29	0.70913	0.70920	0.00015
17.25	0.70942	0.70916	0.00017
17.21	0.70958	0.70915	0.00017
17.16	0.70932	0.70915	0.00016
17.12	0.70885	0.70913	0.00016
17.08	0.70918	0.70911	0.00018
17.03	0.70909	0.70909	0.00018
16.99	0.70926	0.70912	0.00019
16.95	0.70932	0.70905	0.00023
16.91	0.70883	0.70902	0.00022
16.86	0.70875	0.70910	0.00024
16.82	0.70935	0.70918	0.00024
16.78	0.70951	0.70914	0.00024
16.74	0.70917	0.70911	0.00023
16.69	0.70859	0.70910	0.00023
16.65	0.70902	0.70912	0.00021
16.61	0.70940	0.70912	0.00021
16.56	0.70852	0.70909	0.00020
16.52	0.70904	0.70919	0.00018
16.48	0.70965	0.70922	0.00018
16.44	0.70950	0.70917	0.00015
16.39	0.70903	0.70916	0.00015
16.35	0.70919	0.70919	0.00014
16.31	0.70911	0.70923	0.00017
16.27	0.70877	0.70925	0.00017
16.22	0.70903	0.70928	0.00014
16.18	0.70904	0.70929	0.00013
16.14	0.70955	0.70938	0.00017
16.10	0.70939	0.70936	0.00016
16.05	0.70909	0.70927	0.00024
16.01	0.70944	0.70926	0.00025
15.97	0.70925	0.70922	0.00025
15.92	0.70965	0.70922	0.00025
15.88	0.70930	0.70915	0.00023

15.84	0.70905	0.70912	0.00023
15.80	0.70916	0.70912	0.00023
15.75	0.70989	0.70912	0.00023
15.71	0.70943	0.70899	0.00018
15.67	0.70845	0.70896	0.00016
15.63	0.70897	0.70903	0.00012
15.58	0.70905	0.70907	0.00014
15.54	0.70922	0.70909	0.00014
15.50	0.70900	0.70909	0.00014
15.45	0.70895	0.70908	0.00014
15.41	0.70904	0.70907	0.00015
15.37	0.70919	0.70909	0.00016
15.33	0.70856	0.70904	0.00017
15.28	0.70914	0.70909	0.00014
15.24	0.70920	0.70908	0.00014
15.20	0.70936	0.70909	0.00014
15.16	0.70927	0.70912	0.00017
15.11	0.70915	0.70908	0.00017
15.07	0.70895	0.70910	0.00018
15.03	0.70880	0.70914	0.00018
14.99	0.70927	0.70911	0.00021
14.94	0.70870	0.70908	0.00021
14.90	0.70906	0.70914	0.00019
14.86	0.70905	0.70910	0.00021
14.81	0.70932	0.70909	0.00021
14.77	0.70961	0.70906	0.00021
14.73	0.70887	0.70900	0.00017
14.69	0.70934	0.70903	0.00017
14.64	0.70935	0.70899	0.00015
14.60	0.70855	0.70903	0.00020
14.56	0.70898	0.70908	0.00017
14.52	0.70924	0.70910	0.00017
14.47	0.70868	0.70910	0.00018
14.43	0.70899	0.70916	0.00014
14.39	0.70898	0.70915	0.00015
14.34	0.70905	0.70918	0.00014
14.30	0.70913	0.70916	0.00015
14.26	0.70898	0.70918	0.00015
14.22	0.70970	0.70918	0.00015
14.17	0.70913	0.70911	0.00009
14.09	0.70928	0.70911	0.00010
14.05	0.70920	0.70913	0.00013
14.00	0.70894	0.70910	0.00014
13.96	0.70923	0.70909	0.00014
13.92	0.70890	0.70907	0.00014
13.88	0.70927	0.70906	0.00015
13.83	0.70900	0.70907	0.00016
13.79	0.70905	0.70906	0.00016
13.75	0.70894	0.70909	0.00018
13.70	0.70927	0.70907	0.00019

13.66	0.70955	0.70902	0.00019
13.62	0.70882	0.70898	0.00016
13.58	0.70893	0.70905	0.00018
13.53	0.70901	0.70904	0.00018
13.49	0.70876	0.70906	0.00018
13.45	0.70940	0.70910	0.00017
13.41	0.70890	0.70911	0.00017
13.36	0.70937	0.70914	0.00017
13.32	0.70869	0.70908	0.00018
13.28	0.70880	0.70908	0.00018
13.23	0.70916	0.70910	0.00017
13.19	0.70945	0.70912	0.00017
13.15	0.70890	0.70909	0.00016
13.11	0.70921	0.70915	0.00016
13.06	0.70913	0.70912	0.00017
13.02	0.70947	0.70918	0.00021
12.98	0.70925	0.70914	0.00020
12.94	0.70874	0.70916	0.00021
12.89	0.70872	0.70922	0.00019
12.85	0.70900	0.70927	0.00016
12.81	0.70930	0.70930	0.00015
12.77	0.70923	0.70929	0.00015
12.72	0.70944	0.70927	0.00015
12.68	0.70892	0.70920	0.00019
12.64	0.70974	0.70918	0.00020
12.59	0.70902	0.70909	0.00017
12.55	0.70947	0.70911	0.00017
12.51	0.70936	0.70902	0.00018
12.47	0.70926	0.70904	0.00020
12.42	0.70921	0.70902	0.00019
12.38	0.70922	0.70896	0.00020
12.34	0.70907	0.70894	0.00020
12.30	0.70869	0.70895	0.00020
12.25	0.70879	0.70901	0.00020
12.21	0.70879	0.70902	0.00020
12.17	0.70924	0.70906	0.00020
12.12	0.70858	0.70911	0.00024
12.08	0.70957	0.70917	0.00021
12.04	0.70909	0.70911	0.00020
12.00	0.70862	0.70907	0.00021
11.95	0.70901	0.70913	0.00018
11.91	0.70913	0.70914	0.00018
11.87	0.70934	0.70915	0.00018
11.83	0.70880	0.70916	0.00019
11.78	0.70923	0.70919	0.00018
11.74	0.70976	0.70913	0.00020
11.70	0.70916	0.70907	0.00015
11.66	0.70894	0.70907	0.00015
11.61	0.70876	0.70907	0.00015
11.57	0.70916	0.70915	0.00017

11.53	0.70908	0.70919	0.00019
11.48	0.70923	0.70920	0.00018
11.44	0.70952	0.70917	0.00019
11.40	0.70904	0.70914	0.00018
11.36	0.70869	0.70918	0.00018
11.31	0.70913	0.70925	0.00015
11.27	0.70917	0.70925	0.00015
11.23	0.70889	0.70924	0.00015
11.19	0.70958	0.70928	0.00013
11.14	0.70957	0.70922	0.00012
11.10	0.70918	0.70920	0.00009
11.06	0.70896	0.70914	0.00014
11.01	0.70922	0.70914	0.00014
10.97	0.70938	0.70913	0.00014
10.93	0.70937	0.70910	0.00013
10.89	0.70916	0.70900	0.00018
10.84	0.70903	0.70899	0.00018
10.80	0.70930	0.70900	0.00018
10.76	0.70906	0.70891	0.00020
10.72	0.70930	0.70891	0.00020
10.67	0.70866	0.70891	0.00020
10.63	0.70896	0.70895	0.00019
10.59	0.70906	0.70895	0.00019
10.55	0.70906	0.70896	0.00020
10.50	0.70837	0.70891	0.00021
10.46	0.70909	0.70901	0.00019
10.42	0.70913	0.70902	0.00019
10.37	0.70843	0.70901	0.00019
10.33	0.70901	0.70908	0.00015
10.29	0.70934	0.70909	0.00016
10.25	0.70903	0.70905	0.00016
10.20	0.70896	0.70905	0.00017
10.16	0.70916	0.70907	0.00019
10.12	0.70857	0.70905	0.00022
10.08	0.70938	0.70921	0.00009
10.03	0.70915	0.70913	0.00001
9.99	0.70911	0.70911	0.00001
9.26	0.70900	0.70930	0.00016
9.22	0.70923	0.70927	0.00019
9.18	0.70928	0.70927	0.00019
9.14	0.70931	0.70929	0.00019
9.09	0.70943	0.70930	0.00019
9.05	0.70903	0.70928	0.00019
9.01	0.70915	0.70928	0.00019
8.97	0.70920	0.70931	0.00019
8.92	0.70946	0.70929	0.00020
8.88	0.70985	0.70929	0.00020
8.84	0.70871	0.70922	0.00016
8.79	0.70931	0.70928	0.00011
8.75	0.70944	0.70930	0.00012

8.71	0.70943	0.70929	0.00012
8.67	0.70917	0.70929	0.00012
8.62	0.70910	0.70930	0.00011
8.58	0.70945	0.70936	0.00013
8.54	0.70899	0.70933	0.00013
8.50	0.70946	0.70941	0.00013
8.45	0.70911	0.70935	0.00018
8.41	0.70930	0.70936	0.00017
8.37	0.70951	0.70932	0.00019
8.33	0.70935	0.70928	0.00019
8.28	0.70947	0.70926	0.00020
8.24	0.70929	0.70921	0.00020
8.20	0.70968	0.70919	0.00020
8.15	0.70919	0.70915	0.00017
8.11	0.70978	0.70916	0.00017
8.07	0.70881	0.70910	0.00010
8.03	0.70919	0.70915	0.00008
7.98	0.70895	0.70909	0.00013
7.94	0.70905	0.70909	0.00014
7.90	0.70915	0.70915	0.00018
7.86	0.70896	0.70910	0.00019
7.81	0.70913	0.70911	0.00019
7.77	0.70929	0.70905	0.00022
7.73	0.70929	0.70900	0.00023
7.68	0.70917	0.70902	0.00024
7.64	0.70929	0.70902	0.00025
7.60	0.70860	0.70904	0.00026
7.56	0.70893	0.70909	0.00024
7.51	0.70965	0.70910	0.00024
7.47	0.70873	0.70904	0.00021
7.43	0.70904	0.70904	0.00021
7.39	0.70852	0.70902	0.00021
7.34	0.70874	0.70907	0.00018
7.30	0.70953	0.70907	0.00018
7.26	0.70920	0.70901	0.00015
7.22	0.70950	0.70899	0.00014
7.17	0.70911	0.70894	0.00009
7.13	0.70896	0.70893	0.00008
7.09	0.70907	0.70892	0.00008
7.04	0.70872	0.70890	0.00008
7.00	0.70881	0.70892	0.00006
6.96	0.70906	0.70893	0.00006
6.92	0.70875	0.70889	0.00008
6.87	0.70892	0.70890	0.00007
6.83	0.70898	0.70891	0.00008
6.79	0.70901	0.70890	0.00008
6.75	0.70903	0.70887	0.00008
6.70	0.70884	0.70888	0.00008
6.66	0.70891	0.70887	0.00008
6.62	0.70892	0.70885	0.00009

6.57	0.70892	0.70885	0.00009
6.53	0.70865	0.70881	0.00010
6.49	0.70878	0.70886	0.00011
6.45	0.70908	0.70891	0.00014
6.40	0.70883	0.70887	0.00015
6.36	0.70878	0.70887	0.00014
6.32	0.70907	0.70889	0.00014
6.28	0.70878	0.70891	0.00016
6.23	0.70870	0.70891	0.00016
6.19	0.70889	0.70894	0.00016
6.15	0.70859	0.70895	0.00016
6.11	0.70911	0.70901	0.00014
6.06	0.70931	0.70902	0.00014
6.02	0.70863	0.70898	0.00013
5.98	0.70887	0.70899	0.00012
5.93	0.70895	0.70899	0.00012
5.89	0.70928	0.70902	0.00013
5.85	0.70874	0.70903	0.00014
5.81	0.70899	0.70905	0.00012
5.76	0.70905	0.70902	0.00014
5.72	0.70920	0.70900	0.00015
5.68	0.70916	0.70896	0.00014
5.64	0.70889	0.70889	0.00016
5.59	0.70872	0.70892	0.00017
5.55	0.70896	0.70889	0.00020
5.51	0.70923	0.70893	0.00021
5.46	0.70937	0.70891	0.00020
5.42	0.70890	0.70887	0.00018
5.38	0.70870	0.70884	0.00019
5.34	0.70883	0.70889	0.00020
5.29	0.70882	0.70889	0.00020
5.25	0.70850	0.70897	0.00024
5.21	0.70921	0.70898	0.00023
5.17	0.70843	0.70892	0.00024
5.12	0.70930	0.70899	0.00022
5.08	0.70906	0.70900	0.00022
5.04	0.70899	0.70909	0.00029
5.00	0.70856	0.70908	0.00029
4.95	0.70922	0.70914	0.00027
4.91	0.70884	0.70917	0.00028
4.87	0.70959	0.70917	0.00028
4.82	0.70862	0.70910	0.00027
4.78	0.70859	0.70917	0.00025
4.74	0.70913	0.70923	0.00021
4.70	0.70941	0.70923	0.00021
4.65	0.70992	0.70918	0.00022
4.61	0.70893	0.70911	0.00014
4.57	0.70916	0.70915	0.00014
4.53	0.70953	0.70917	0.00015
4.48	0.70878	0.70911	0.00012

4.44	0.70893	0.70916	0.00010
4.40	0.70928	0.70919	0.00009
4.35	0.70919	0.70918	0.00009
4.31	0.70918	0.70918	0.00009
4.27	0.70890	0.70912	0.00015
4.23	0.70926	0.70914	0.00014
4.18	0.70926	0.70909	0.00015
4.14	0.70936	0.70909	0.00015
4.10	0.70900	0.70906	0.00014
4.06	0.70928	0.70907	0.00014
4.01	0.70922	0.70906	0.00013
3.97	0.70913	0.70906	0.00013
3.93	0.70921	0.70909	0.00016
3.89	0.70858	0.70909	0.00015
3.84	0.70910	0.70918	0.00013
3.80	0.70878	0.70918	0.00013
3.76	0.70925	0.70923	0.00009
3.71	0.70910	0.70923	0.00009
3.67	0.70909	0.70927	0.00010
3.63	0.70918	0.70928	0.00010
3.59	0.70919	0.70931	0.00010
3.54	0.70946	0.70932	0.00009
3.50	0.70912	0.70923	0.00018
3.46	0.70951	0.70930	0.00021
3.42	0.70913	0.70921	0.00025
3.37	0.70928	0.70916	0.00027
3.33	0.70921	0.70908	0.00030
3.29	0.70949	0.70910	0.00031
3.24	0.70923	0.70910	0.00031
3.20	0.70944	0.70908	0.00031
3.16	0.70936	0.70906	0.00030
3.12	0.70853	0.70905	0.00029
3.07	0.70983	0.70911	0.00027
3.03	0.70861	0.70908	0.00023
2.99	0.70865	0.70912	0.00021
2.95	0.70848	0.70923	0.00021
2.90	0.70935	0.70931	0.00014
2.86	0.70950	0.70930	0.00014
2.82	0.70909	0.70928	0.00013
2.78	0.70923	0.70926	0.00015
2.73	0.70922	0.70926	0.00015
2.69	0.70918	0.70925	0.00015
2.65	0.70946	0.70927	0.00015
2.60	0.70900	0.70921	0.00016
2.56	0.70974	0.70921	0.00016
2.52	0.70929	0.70915	0.00012
2.48	0.70933	0.70919	0.00015
2.43	0.70930	0.70917	0.00015
2.39	0.70887	0.70918	0.00015
2.35	0.70916	0.70919	0.00014

2.31	0.70916	0.70915	0.00016
2.26	0.70937	0.70916	0.00016
2.22	0.70888	0.70919	0.00019
2.18	0.70899	0.70927	0.00020
2.13	0.70920	0.70936	0.00022
2.09	0.70963	0.70936	0.00022
2.05	0.70916	0.70930	0.00022
2.01	0.70935	0.70931	0.00022
1.96	0.70901	0.70933	0.00023
1.92	0.70880	0.70939	0.00022
1.88	0.70918	0.70946	0.00018
1.84	0.70967	0.70951	0.00017
1.79	0.70969	0.70946	0.00017
1.75	0.70992	0.70942	0.00017
1.71	0.70920	0.70938	0.00013
1.67	0.70904	0.70943	0.00014
1.62	0.70926	0.70949	0.00011
1.58	0.70957	0.70944	0.00018
1.54	0.70958	0.70942	0.00018
1.49	0.70952	0.70942	0.00017
1.45	0.70964	0.70937	0.00018
1.41	0.70922	0.70934	0.00017
1.37	0.70927	0.70932	0.00019
1.32	0.70948	0.70932	0.00019
1.28	0.70972	0.70932	0.00019
1.24	0.70960	0.70924	0.00018
1.20	0.70877	0.70915	0.00020
1.15	0.70940	0.70919	0.00018
1.11	0.70954	0.70915	0.00018
1.07	0.70910	0.70916	0.00018
1.02	0.70935	0.70918	0.00019
0.98	0.70892	0.70919	0.00019
0.94	0.70932	0.70929	0.00023
0.90	0.70945	0.70929	0.00023
0.85	0.70897	0.70924	0.00024
0.81	0.70863	0.70926	0.00023
0.77	0.70922	0.70932	0.00018
0.73	0.70901	0.70927	0.00022
0.68	0.70959	0.70934	0.00023
0.64	0.70940	0.70929	0.00023
0.60	0.70938	0.70927	0.00023
0.56	0.70993	0.70924	0.00023
0.51	0.70934	0.70918	0.00017
0.47	0.70890	0.70918	0.00017
0.43	0.70924	0.70921	0.00016
0.38	0.70917	0.70921	0.00017
0.34	0.70872	0.70921	0.00018
0.30	0.70972	0.70928	0.00014
0.26	0.70906	0.70921	0.00008
0.21	0.70924	0.70924	0.00008

0.17	0.70904	0.70924	0.00009
0.13	0.70938	0.70931	0.00004
0.09	0.70929	0.70927	0.00002
0.04	0.70925		

ARB 23.2.1 (M2)

Distance from cervix (mm)	$^{87}Sr/^{86}Sr$	10 point mov. average	2 SE on mov. average
46.96	0.70965	0.71010	0.00018
46.91	0.71023	0.71016	0.00015
46.87	0.71001	0.71013	0.00015
46.82	0.71028	0.71013	0.00015
46.78	0.71030	0.71015	0.00017
46.74	0.71066	0.71010	0.00017
46.69	0.70990	0.71000	0.00014
46.65	0.71010	0.71003	0.00014
46.61	0.70993	0.70999	0.00016
46.56	0.70998	0.70995	0.00018
46.52	0.71024	0.70994	0.00018
46.47	0.70989	0.70991	0.00017
46.43	0.71003	0.70994	0.00018
46.39	0.71048	0.70992	0.00018
46.34	0.70983	0.70988	0.00014
46.30	0.70969	0.70990	0.00014
46.26	0.71018	0.70990	0.00014
46.21	0.70966	0.70985	0.00013
46.17	0.70957	0.70986	0.00013
46.13	0.70986	0.70990	0.00011
46.08	0.70990	0.70990	0.00011
46.04	0.71021	0.70988	0.00012
45.99	0.70986	0.70988	0.00012
45.95	0.71009	0.70988	0.00012
45.91	0.70998	0.70982	0.00014
45.86	0.70965	0.70981	0.00014
45.82	0.70971	0.70983	0.00013
45.78	0.70973	0.70986	0.00013
45.73	0.71001	0.70986	0.00013
45.69	0.70986	0.70986	0.00013
45.64	0.70969	0.70987	0.00013
45.60	0.71021	0.70993	0.00014
45.56	0.70990	0.70991	0.00014
45.51	0.70944	0.70992	0.00014
45.47	0.70996	0.70996	0.00009
45.43	0.70980	0.70993	0.00011
45.38	0.70996	0.70992	0.00011
45.34	0.70980	0.70992	0.00011
45.29	0.71002	0.70995	0.00011
45.25	0.70993	0.70996	0.00012
45.21	0.71026	0.70999	0.00013
45.16	0.71009	0.70998	0.00012
45.12	0.71000	0.70998	0.00012
45.08	0.70976	0.70995	0.00013
45.03	0.70972	0.70994	0.00015
44.99	0.70968	0.70999	0.00015
44.94	0.70998	0.71007	0.00015

44.90	0.71003	0.71005	0.00016
44.86	0.71019	0.71004	0.00016
44.81	0.71023	0.71003	0.00016
44.77	0.71008	0.70999	0.00016
44.73	0.71014	0.70998	0.00016
44.68	0.70972	0.70999	0.00017
44.64	0.70962	0.71002	0.00015
44.59	0.71022	0.71007	0.00013
44.55	0.71044	0.71005	0.00012
44.51	0.70980	0.70998	0.00010
44.46	0.70997	0.70997	0.00010
44.42	0.71008	0.70992	0.00015
44.38	0.70986	0.70994	0.00016
44.33	0.70994	0.70994	0.00016
44.29	0.71028	0.70994	0.00016
44.25	0.71001	0.70989	0.00015
44.20	0.71009	0.70986	0.00015
44.16	0.71002	0.70981	0.00015
44.11	0.70973	0.70979	0.00014
44.07	0.70976	0.70977	0.00015
44.03	0.70946	0.70975	0.00015
43.98	0.71028	0.70979	0.00014
43.94	0.70978	0.70974	0.00009
43.90	0.70998	0.70977	0.00011
43.85	0.70980	0.70983	0.00020
43.81	0.70974	0.70981	0.00021
43.76	0.70957	0.70983	0.00022
43.72	0.70976	0.70989	0.00022
43.68	0.70955	0.70992	0.00022
43.63	0.70957	0.70997	0.00020
43.59	0.70981	0.70996	0.00021
43.55	0.70983	0.71001	0.00022
43.50	0.71007	0.71004	0.00022
43.46	0.71064	0.71003	0.00022
43.41	0.70951	0.70994	0.00017
43.37	0.70995	0.70995	0.00016
43.33	0.71018	0.70990	0.00019
43.28	0.71012	0.70985	0.00019
43.24	0.71001	0.70982	0.00018
43.20	0.70948	0.70980	0.00017
43.15	0.71030	0.70982	0.00016
43.11	0.71010	0.70974	0.00013
43.06	0.70997	0.70972	0.00011
43.02	0.70978	0.70972	0.00011
42.98	0.70959	0.70978	0.00018
42.93	0.70944	0.70981	0.00017
42.89	0.70971	0.70985	0.00015
42.85	0.70980	0.70986	0.00015
42.80	0.70985	0.70988	0.00015
42.76	0.70961	0.70991	0.00015

42.71	0.70954	0.71000	0.00018
42.67	0.70993	0.70999	0.00018
42.63	0.70994	0.71001	0.00018
42.58	0.71041	0.70997	0.00021
42.54	0.70984	0.70987	0.00021
42.50	0.70983	0.70985	0.00022
42.45	0.70984	0.70983	0.00022
42.41	0.71001	0.70984	0.00022
42.37	0.71010	0.70985	0.00023
42.32	0.71052	0.70981	0.00023
42.28	0.70952	0.70975	0.00017
42.23	0.71012	0.70978	0.00017
42.19	0.70953	0.70973	0.00015
42.15	0.70938	0.70972	0.00016
42.10	0.70970	0.70976	0.00014
42.06	0.70958	0.70978	0.00014
42.02	0.70990	0.70977	0.00014
41.97	0.71019	0.70976	0.00014
41.93	0.70962	0.70971	0.00011
41.88	0.70995	0.70970	0.00011
41.84	0.70978	0.70969	0.00010
41.80	0.70968	0.70970	0.00011
41.75	0.70939	0.70971	0.00011
41.71	0.70979	0.70971	0.00011
41.67	0.70988	0.70973	0.00012
41.62	0.70954	0.70971	0.00011
41.58	0.70982	0.70972	0.00011
41.53	0.70964	0.70969	0.00011
41.49	0.70951	0.70969	0.00011
41.45	0.70984	0.70971	0.00010
41.40	0.70994	0.70967	0.00011
41.36	0.70974	0.70965	0.00009
41.32	0.70941	0.70964	0.00009
41.27	0.70992	0.70972	0.00013
41.23	0.70971	0.70969	0.00013
41.18	0.70967	0.70968	0.00013
41.14	0.70952	0.70966	0.00013
41.10	0.70965	0.70970	0.00014
41.05	0.70969	0.70975	0.00016
41.01	0.70948	0.70978	0.00017
40.97	0.70967	0.70981	0.00015
40.92	0.70964	0.70981	0.00015
40.88	0.71022	0.70977	0.00019
40.83	0.70965	0.70971	0.00016
40.79	0.70958	0.70973	0.00016
40.75	0.70950	0.70974	0.00016
40.70	0.70997	0.70980	0.00017
40.66	0.71013	0.70979	0.00016
40.62	0.70996	0.70975	0.00014
40.57	0.70978	0.70976	0.00015

40.53	0.70968	0.70980	0.00017
40.49	0.70928	0.70983	0.00017
40.44	0.70961	0.70991	0.00013
40.40	0.70985	0.70989	0.00017
40.35	0.70966	0.70989	0.00017
40.31	0.71012	0.70986	0.00020
40.27	0.70984	0.70985	0.00019
40.22	0.70972	0.70984	0.00019
40.18	0.71009	0.70982	0.00020
40.14	0.71014	0.70979	0.00019
40.09	0.71002	0.70976	0.00018
40.05	0.71010	0.70979	0.00021
40.00	0.70932	0.70975	0.00019
39.96	0.70988	0.70974	0.00020
39.92	0.70933	0.70971	0.00020
39.87	0.71004	0.70972	0.00020
39.83	0.70979	0.70970	0.00019
39.79	0.70952	0.70970	0.00019
39.74	0.70973	0.70974	0.00019
39.70	0.70984	0.70977	0.00020
39.65	0.71031	0.70973	0.00020
39.61	0.70972	0.70967	0.00016
39.57	0.70924	0.70966	0.00016
39.52	0.70961	0.70965	0.00018
39.48	0.70937	0.70967	0.00018
39.44	0.70991	0.70967	0.00017
39.39	0.70976	0.70963	0.00017
39.35	0.70995	0.70962	0.00017
39.30	0.70997	0.70963	0.00017
39.26	0.70946	0.70957	0.00016
39.22	0.70970	0.70956	0.00017
39.17	0.70969	0.70958	0.00018
39.13	0.70910	0.70961	0.00020
39.09	0.70977	0.70969	0.00017
39.04	0.70942	0.70972	0.00019
39.00	0.70947	0.70975	0.00017
38.95	0.70970	0.70978	0.00016
38.91	0.71000	0.70974	0.00019
38.87	0.70944	0.70972	0.00018
38.82	0.70929	0.70978	0.00018
38.78	0.70991	0.70987	0.00016
38.74	0.71001	0.70985	0.00016
38.69	0.70987	0.70985	0.00016
38.65	0.71010	0.70985	0.00016
38.61	0.70971	0.70982	0.00016
38.56	0.70974	0.70988	0.00018
38.52	0.70932	0.70983	0.00022
38.47	0.70980	0.70983	0.00021
38.43	0.71004	0.70980	0.00022
38.39	0.71019	0.70976	0.00022

38.34	0.70969	0.70975	0.00021
38.30	0.71009	0.70979	0.00022
38.26	0.70986	0.70975	0.00021
38.21	0.70978	0.70974	0.00021
38.17	0.71029	0.70978	0.00023
38.12	0.70924	0.70970	0.00021
38.08	0.70936	0.70972	0.00019
38.04	0.70949	0.70974	0.00018
37.99	0.70964	0.70975	0.00018
37.95	0.71008	0.70973	0.00018
37.91	0.71005	0.70973	0.00018
37.86	0.70965	0.70969	0.00017
37.82	0.70980	0.70966	0.00018
37.77	0.71022	0.70964	0.00018
37.73	0.70947	0.70961	0.00014
37.69	0.70942	0.70963	0.00014
37.64	0.70954	0.70968	0.00014
37.60	0.70964	0.70970	0.00014
37.56	0.70944	0.70971	0.00014
37.51	0.71004	0.70974	0.00012
37.47	0.70965	0.70971	0.00010
37.42	0.70932	0.70972	0.00010
37.38	0.70967	0.70980	0.00008
37.34	0.70994	0.70979	0.00009
37.29	0.70966	0.70980	0.00009
37.25	0.70985	0.70980	0.00009
37.21	0.70977	0.70977	0.00011
37.16	0.70977	0.70977	0.00011
37.12	0.70969	0.70975	0.00012
37.07	0.70979	0.70980	0.00014
37.03	0.70978	0.70978	0.00014
36.99	0.71007	0.70975	0.00015
36.94	0.70958	0.70971	0.00014
36.90	0.71000	0.70973	0.00013
36.86	0.70972	0.70970	0.00012
36.81	0.70949	0.70966	0.00014
36.77	0.70981	0.70966	0.00014
36.73	0.70956	0.70964	0.00013
36.68	0.71015	0.70964	0.00013
36.64	0.70965	0.70960	0.00008
36.59	0.70950	0.70959	0.00008
36.55	0.70968	0.70959	0.00008
36.51	0.70978	0.70957	0.00007
36.46	0.70964	0.70959	0.00010
36.42	0.70937	0.70961	0.00011
36.38	0.70949	0.70965	0.00010
36.33	0.70962	0.70966	0.00009
36.29	0.70955	0.70969	0.00010
36.24	0.70971	0.70970	0.00010
36.20	0.70952	0.70964	0.00015

36.16	0.70951	0.70962	0.00016
36.11	0.70956	0.70964	0.00016
36.07	0.70997	0.70963	0.00016
36.03	0.70979	0.70960	0.00014
35.98	0.70975	0.70958	0.00014
35.94	0.70962	0.70960	0.00016
35.89	0.70989	0.70962	0.00016
35.85	0.70966	0.70960	0.00015
35.81	0.70912	0.70958	0.00016
35.76	0.70935	0.70963	0.00012
35.72	0.70969	0.70968	0.00011
35.68	0.70949	0.70970	0.00012
35.63	0.70960	0.70972	0.00011
35.59	0.70958	0.70971	0.00011
35.54	0.70999	0.70969	0.00013
35.50	0.70984	0.70962	0.00013
35.46	0.70965	0.70955	0.00015
35.41	0.70946	0.70953	0.00015
35.37	0.70962	0.70958	0.00017
35.33	0.70985	0.70960	0.00018
35.28	0.70993	0.70961	0.00018
35.24	0.70963	0.70957	0.00017
35.19	0.70954	0.70958	0.00017
35.15	0.70942	0.70961	0.00018
35.11	0.70930	0.70961	0.00018
35.06	0.70911	0.70963	0.00017
35.02	0.70948	0.70967	0.00013
34.98	0.70991	0.70964	0.00015
34.93	0.70982	0.70959	0.00014
34.89	0.70993	0.70957	0.00014
34.85	0.70952	0.70950	0.00012
34.80	0.70973	0.70951	0.00012
34.76	0.70986	0.70954	0.00015
34.71	0.70946	0.70949	0.00014
34.67	0.70946	0.70949	0.00014
34.63	0.70948	0.70949	0.00014
34.58	0.70922	0.70951	0.00014
34.54	0.70945	0.70954	0.00013
34.50	0.70954	0.70956	0.00013
34.45	0.70927	0.70961	0.00015
34.41	0.70964	0.70964	0.00013
34.36	0.71000	0.70966	0.00014
34.32	0.70939	0.70964	0.00012
34.28	0.70943	0.70963	0.00012
34.23	0.70948	0.70966	0.00011
34.19	0.70965	0.70966	0.00011
34.15	0.70954	0.70968	0.00012
34.10	0.70968	0.70967	0.00012
34.06	0.70999	0.70965	0.00013
34.01	0.70965	0.70962	0.00011

33.97	0.70979	0.70960	0.00011
33.93	0.70977	0.70959	0.00011
33.88	0.70935	0.70958	0.00011
33.84	0.70968	0.70963	0.00010
33.80	0.70944	0.70961	0.00011
33.75	0.70985	0.70962	0.00010
33.71	0.70950	0.70957	0.00011
33.66	0.70945	0.70957	0.00011
33.62	0.70971	0.70957	0.00011
33.58	0.70944	0.70957	0.00011
33.53	0.70967	0.70961	0.00012
33.49	0.70974	0.70962	0.00012
33.45	0.70985	0.70962	0.00012
33.40	0.70946	0.70958	0.00011
33.36	0.70956	0.70960	0.00011
33.32	0.70929	0.70965	0.00013
33.27	0.70952	0.70965	0.00014
33.23	0.70946	0.70965	0.00014
33.18	0.70968	0.70966	0.00013
33.14	0.70990	0.70964	0.00014
33.10	0.70970	0.70960	0.00013
33.05	0.70973	0.70956	0.00014
33.01	0.70952	0.70952	0.00014
32.97	0.70967	0.70950	0.00015
32.92	0.71002	0.70951	0.00015
32.88	0.70926	0.70948	0.00011
32.83	0.70954	0.70952	0.00010
32.79	0.70952	0.70953	0.00011
32.75	0.70950	0.70954	0.00011
32.70	0.70954	0.70952	0.00012
32.66	0.70925	0.70950	0.00012
32.62	0.70937	0.70953	0.00011
32.57	0.70932	0.70956	0.00011
32.53	0.70978	0.70958	0.00010
32.48	0.70966	0.70959	0.00010
32.44	0.70967	0.70960	0.00011
32.40	0.70973	0.70960	0.00011
32.35	0.70957	0.70959	0.00011
32.31	0.70933	0.70956	0.00012
32.27	0.70938	0.70965	0.00016
32.22	0.70946	0.70965	0.00016
32.18	0.70967	0.70966	0.00015
32.13	0.70955	0.70964	0.00016
32.09	0.70985	0.70963	0.00016
32.05	0.70979	0.70962	0.00016
32.00	0.70972	0.70956	0.00017
31.96	0.70957	0.70957	0.00018
31.92	0.70930	0.70956	0.00019
31.87	0.71018	0.70954	0.00020
31.83	0.70944	0.70947	0.00014

31.78	0.70957	0.70949	0.00014
31.74	0.70943	0.70953	0.00017
31.70	0.70946	0.70950	0.00019
31.65	0.70969	0.70949	0.00019
31.61	0.70921	0.70950	0.00020
31.57	0.70989	0.70953	0.00019
31.52	0.70939	0.70949	0.00017
31.48	0.70915	0.70950	0.00017
31.44	0.70942	0.70955	0.00015
31.39	0.70969	0.70959	0.00016
31.35	0.71000	0.70962	0.00018
31.30	0.70915	0.70961	0.00017
31.26	0.70935	0.70963	0.00014
31.22	0.70979	0.70964	0.00014
31.17	0.70952	0.70962	0.00013
31.13	0.70941	0.70962	0.00013
31.09	0.70953	0.70966	0.00013
31.04	0.70964	0.70970	0.00014
31.00	0.70980	0.70972	0.00014
30.95	0.71005	0.70970	0.00014
30.91	0.70984	0.70968	0.00013
30.87	0.70939	0.70964	0.00013
30.82	0.70946	0.70968	0.00012
30.78	0.70952	0.70971	0.00011
30.74	0.70954	0.70978	0.00013
30.69	0.70985	0.70978	0.00013
30.65	0.70994	0.70975	0.00013
30.60	0.70977	0.70972	0.00013
30.56	0.70961	0.70968	0.00015
30.52	0.70990	0.70967	0.00015
30.47	0.70945	0.70967	0.00015
30.43	0.70975	0.70971	0.00014
30.39	0.70981	0.70972	0.00015
30.34	0.71014	0.70969	0.00015
30.30	0.70955	0.70961	0.00014
30.25	0.70958	0.70961	0.00014
30.21	0.70968	0.70961	0.00014
30.17	0.70930	0.70960	0.00013
30.12	0.70958	0.70966	0.00013
30.08	0.70981	0.70968	0.00013
30.04	0.70992	0.70967	0.00012
29.99	0.70986	0.70963	0.00011
29.95	0.70952	0.70960	0.00010
29.90	0.70929	0.70960	0.00010
29.86	0.70959	0.70964	0.00007
29.82	0.70955	0.70959	0.00012
29.77	0.70963	0.70958	0.00013
29.73	0.70986	0.70959	0.00013
29.69	0.70979	0.70952	0.00013
29.64	0.70970	0.70953	0.00014

29.60	0.70949	0.70956	0.00017
29.56	0.70955	0.70957	0.00017
29.51	0.70957	0.70959	0.00018
29.47	0.70965	0.70957	0.00018
29.42	0.70916	0.70953	0.00019
29.38	0.70941	0.70961	0.00018
29.34	0.70971	0.70965	0.00019
29.29	0.70920	0.70961	0.00020
29.25	0.70986	0.70965	0.00018
29.21	0.71004	0.70965	0.00018
29.16	0.70953	0.70960	0.00016
29.12	0.70981	0.70959	0.00016
29.07	0.70934	0.70953	0.00018
29.03	0.70928	0.70954	0.00017
28.99	0.70987	0.70953	0.00018
28.94	0.70991	0.70953	0.00017
28.90	0.70929	0.70950	0.00015
28.86	0.70956	0.70952	0.00015
28.81	0.70992	0.70950	0.00015
28.77	0.70950	0.70944	0.00012
28.72	0.70945	0.70944	0.00012
28.68	0.70918	0.70946	0.00013
28.64	0.70946	0.70948	0.00012
28.59	0.70920	0.70948	0.00012
28.55	0.70983	0.70950	0.00010
28.51	0.70960	0.70949	0.00009
28.46	0.70953	0.70946	0.00010
28.42	0.70932	0.70944	0.00010
28.37	0.70932	0.70946	0.00010
28.33	0.70948	0.70946	0.00009
28.29	0.70965	0.70941	0.00014
28.24	0.70940	0.70938	0.00012
28.20	0.70945	0.70944	0.00019
28.16	0.70941	0.70948	0.00020
28.11	0.70977	0.70946	0.00020
28.07	0.70926	0.70940	0.00020
28.02	0.70935	0.70941	0.00019
27.98	0.70947	0.70941	0.00019
27.94	0.70937	0.70939	0.00020
27.89	0.70897	0.70938	0.00020
27.85	0.70932	0.70943	0.00018
27.81	0.71006	0.70947	0.00019
27.76	0.70978	0.70942	0.00014
27.72	0.70925	0.70937	0.00011
27.68	0.70921	0.70937	0.00011
27.63	0.70930	0.70937	0.00011
27.59	0.70941	0.70937	0.00011
27.54	0.70922	0.70932	0.00014
27.50	0.70926	0.70932	0.00014
27.46	0.70948	0.70932	0.00014

27.41	0.70977	0.70929	0.00014
27.37	0.70949	0.70925	0.00009
27.33	0.70929	0.70925	0.00009
27.28	0.70928	0.70928	0.00011
27.24	0.70919	0.70927	0.00011
27.19	0.70928	0.70926	0.00011
27.15	0.70895	0.70925	0.00011
27.11	0.70926	0.70930	0.00010
27.06	0.70925	0.70932	0.00010
27.02	0.70915	0.70931	0.00011
26.98	0.70939	0.70935	0.00011
26.93	0.70945	0.70936	0.00012
26.89	0.70955	0.70934	0.00012
26.84	0.70925	0.70937	0.00015
26.80	0.70906	0.70937	0.00015
26.76	0.70920	0.70942	0.00013
26.71	0.70944	0.70946	0.00012
26.67	0.70945	0.70947	0.00012
26.63	0.70914	0.70948	0.00013
26.58	0.70958	0.70952	0.00010
26.54	0.70949	0.70950	0.00010
26.49	0.70927	0.70956	0.00015
26.45	0.70983	0.70961	0.00014
26.41	0.70928	0.70954	0.00017
26.36	0.70947	0.70955	0.00016
26.32	0.70962	0.70958	0.00017
26.28	0.70953	0.70961	0.00018
26.23	0.70961	0.70960	0.00018
26.19	0.70951	0.70959	0.00018
26.14	0.70942	0.70958	0.00019
26.10	0.71007	0.70958	0.00019
26.06	0.70977	0.70953	0.00015
26.01	0.70911	0.70952	0.00015
25.97	0.70937	0.70958	0.00012
25.93	0.70980	0.70959	0.00011
25.88	0.70991	0.70953	0.00013
25.84	0.70939	0.70949	0.00010
25.80	0.70958	0.70952	0.00011
25.75	0.70937	0.70953	0.00011
25.71	0.70943	0.70958	0.00012
25.66	0.70957	0.70956	0.00013
25.62	0.70963	0.70959	0.00014
25.58	0.70974	0.70956	0.00015
25.53	0.70952	0.70955	0.00014
25.49	0.70919	0.70954	0.00014
25.45	0.70947	0.70949	0.00021
25.40	0.70971	0.70952	0.00021
25.36	0.70971	0.70952	0.00021
25.31	0.70979	0.70951	0.00021
25.27	0.70928	0.70947	0.00020

25.23	0.70986	0.70953	0.00021
25.18	0.70938	0.70955	0.00022
25.14	0.70962	0.70956	0.00022
25.10	0.70938	0.70958	0.00022
25.05	0.70874	0.70962	0.00022
25.01	0.70977	0.70970	0.00010
24.96	0.70965	0.70965	0.00014
24.92	0.70963	0.70960	0.00017
24.88	0.70944	0.70956	0.00019
24.83	0.70988	0.70957	0.00018
24.79	0.70998	0.70954	0.00017
24.75	0.70955	0.70954	0.00017
24.70	0.70978	0.70950	0.00019
24.66	0.70977	0.70947	0.00018
24.61	0.70957	0.70946	0.00017
24.57	0.70925	0.70943	0.00017
24.53	0.70913	0.70945	0.00017
24.48	0.70923	0.70951	0.00017
24.44	0.70959	0.70957	0.00016
24.40	0.70960	0.70959	0.00017
24.35	0.70997	0.70960	0.00017
24.31	0.70914	0.70958	0.00015
24.26	0.70945	0.70964	0.00012
24.22	0.70967	0.70965	0.00011
24.18	0.70926	0.70967	0.00012
24.13	0.70941	0.70975	0.00011
24.09	0.70979	0.70980	0.00008
24.05	0.70982	0.70979	0.00009
24.00	0.70978	0.70975	0.00010
23.96	0.70968	0.70974	0.00010
23.92	0.70977	0.70969	0.00015
23.87	0.70973	0.70966	0.00016
23.83	0.70963	0.70965	0.00016
23.78	0.70985	0.70967	0.00016
23.74	0.71007	0.70963	0.00016
23.70	0.70990	0.70959	0.00012
23.65	0.70962	0.70959	0.00012
23.61	0.70949	0.70959	0.00012
23.57	0.70969	0.70962	0.00013
23.52	0.70919	0.70965	0.00015
23.48	0.70947	0.70971	0.00011
23.43	0.70963	0.70974	0.00009
23.39	0.70978	0.70974	0.00009
23.35	0.70949	0.70970	0.00011
23.30	0.70966	0.70971	0.00010
23.26	0.70988	0.70971	0.00010
23.22	0.70961	0.70969	0.00009
23.17	0.70980	0.70969	0.00009
23.13	0.71000	0.70967	0.00009
23.08	0.70978	0.70964	0.00006

23.04	0.70975	0.70965	0.00007
23.00	0.70961	0.70965	0.00007
22.95	0.70947	0.70966	0.00007
22.91	0.70959	0.70971	0.00007
22.87	0.70962	0.70968	0.00010
22.82	0.70971	0.70968	0.00010
22.78	0.70959	0.70965	0.00011
22.73	0.70960	0.70967	0.00011
22.69	0.70972	0.70964	0.00013
22.65	0.70988	0.70966	0.00015
22.60	0.70969	0.70963	0.00014
22.56	0.70975	0.70962	0.00014
22.52	0.70991	0.70963	0.00014
22.47	0.70933	0.70960	0.00013
22.43	0.70959	0.70965	0.00011
22.38	0.70948	0.70963	0.00012
22.34	0.70971	0.70962	0.00013
22.30	0.70933	0.70961	0.00013
22.25	0.70998	0.70964	0.00011
22.21	0.70952	0.70955	0.00013
22.17	0.70964	0.70956	0.00013
22.12	0.70976	0.70953	0.00013
22.08	0.70970	0.70950	0.00012
22.04	0.70974	0.70950	0.00012
21.99	0.70942	0.70944	0.00012
21.95	0.70936	0.70948	0.00013
21.90	0.70969	0.70948	0.00013
21.86	0.70960	0.70947	0.00013
21.82	0.70911	0.70945	0.00013
21.77	0.70956	0.70950	0.00010
21.73	0.70937	0.70949	0.00010
21.69	0.70950	0.70947	0.00012
21.64	0.70964	0.70948	0.00012
21.60	0.70920	0.70943	0.00013
21.55	0.70975	0.70946	0.00012
21.51	0.70937	0.70946	0.00012
21.47	0.70964	0.70944	0.00012
21.42	0.70941	0.70945	0.00013
21.38	0.70953	0.70948	0.00014
21.34	0.70944	0.70951	0.00015
21.29	0.70920	0.70951	0.00015
21.25	0.70958	0.70958	0.00016
21.20	0.70915	0.70956	0.00017
21.16	0.70950	0.70967	0.00019
21.12	0.70973	0.70969	0.00019
21.07	0.70926	0.70969	0.00019
21.03	0.70973	0.70970	0.00018
20.99	0.70969	0.70972	0.00018
20.94	0.70981	0.70972	0.00018
20.90	0.70940	0.70968	0.00019

20.85	0.70996	0.70971	0.00018
20.81	0.70933	0.70965	0.00018
20.77	0.71023	0.70968	0.00016
20.72	0.70974	0.70962	0.00011
20.68	0.70972	0.70967	0.00016
20.64	0.70938	0.70968	0.00017
20.59	0.70992	0.70973	0.00015
20.55	0.70967	0.70970	0.00015
20.50	0.70945	0.70967	0.00016
20.46	0.70970	0.70972	0.00017
20.42	0.70937	0.70974	0.00017
20.37	0.70965	0.70977	0.00015
20.33	0.70964	0.70978	0.00015
20.29	0.71022	0.70980	0.00015
20.24	0.70984	0.70975	0.00012
20.20	0.70979	0.70973	0.00012
20.16	0.70966	0.70974	0.00013
20.11	0.70932	0.70979	0.00014
20.07	0.71001	0.70987	0.00012
20.02	0.70988	0.70988	0.00013
19.98	0.70963	0.70982	0.00018
19.94	0.70975	0.70986	0.00017
19.89	0.70991	0.70986	0.00017
19.85	0.70967	0.70985	0.00018
19.81	0.70967	0.70987	0.00019
19.76	0.70995	0.70990	0.00020
19.72	0.71008	0.70989	0.00022
19.67	0.71014	0.70986	0.00024
19.63	0.71016	0.70978	0.00025
19.59	0.70928	0.70966	0.00023
19.54	0.71000	0.70985	0.00013
19.50	0.70970	0.70970	0.00010
18.80	0.70959	0.70949	0.00008
18.76	0.70944	0.70950	0.00009
18.71	0.70953	0.70952	0.00009
18.67	0.70930	0.70957	0.00014
18.62	0.70969	0.70959	0.00013
18.58	0.70947	0.70960	0.00013
18.54	0.70950	0.70962	0.00013
18.49	0.70931	0.70963	0.00013
18.45	0.70948	0.70966	0.00010
18.41	0.70955	0.70968	0.00010
18.36	0.70971	0.70969	0.00009
18.32	0.70966	0.70967	0.00010
18.28	0.71006	0.70964	0.00011
18.23	0.70950	0.70966	0.00013
18.19	0.70975	0.70970	0.00014
18.14	0.70964	0.70972	0.00015
18.10	0.70968	0.70975	0.00016
18.06	0.70961	0.70978	0.00016

18.01	0.70968	0.70976	0.00017
17.97	0.70963	0.70977	0.00017
17.93	0.70948	0.70979	0.00017
17.88	0.70941	0.70983	0.00016
17.84	0.71018	0.70986	0.00014
17.79	0.70996	0.70979	0.00014
17.75	0.70992	0.70976	0.00013
17.71	0.70999	0.70976	0.00014
17.66	0.70996	0.70974	0.00013
17.62	0.70939	0.70973	0.00012
17.58	0.70975	0.70978	0.00010
17.53	0.70984	0.70977	0.00010
17.49	0.70995	0.70978	0.00010
17.44	0.70962	0.70977	0.00010
17.40	0.70948	0.70977	0.00009
17.36	0.70966	0.70982	0.00007
17.31	0.70999	0.70980	0.00010
17.27	0.70976	0.70976	0.00009
17.23	0.70988	0.70973	0.00011
17.18	0.70982	0.70977	0.00015
17.14	0.70971	0.70978	0.00015
17.09	0.70990	0.70977	0.00015
17.05	0.70984	0.70975	0.00015
17.01	0.70966	0.70976	0.00015
16.96	0.70994	0.70979	0.00015
16.92	0.70945	0.70978	0.00015
16.88	0.70967	0.70980	0.00014
16.83	0.70946	0.70984	0.00015
16.79	0.71022	0.70983	0.00016
16.74	0.70992	0.70978	0.00014
16.70	0.70965	0.70972	0.00017
16.66	0.70971	0.70973	0.00016
16.61	0.70994	0.70976	0.00017
16.57	0.70990	0.70973	0.00017
16.53	0.70987	0.70966	0.00020
16.48	0.70962	0.70967	0.00021
16.44	0.71012	0.70964	0.00022
16.40	0.70934	0.70960	0.00020
16.35	0.70977	0.70960	0.00020
16.31	0.70928	0.70958	0.00019
16.26	0.70977	0.70961	0.00018
16.22	0.70999	0.70961	0.00018
16.18	0.70966	0.70961	0.00018
16.13	0.70915	0.70962	0.00018
16.09	0.71003	0.70968	0.00015
16.05	0.70927	0.70962	0.00014
16.00	0.70969	0.70969	0.00013
15.96	0.70935	0.70964	0.00015
15.91	0.70956	0.70969	0.00014
15.87	0.70964	0.70968	0.00015

15.83	0.70975	0.70970	0.00015
15.78	0.71001	0.70972	0.00016
15.74	0.70970	0.70971	0.00015
15.70	0.70975	0.70970	0.00015
15.65	0.70946	0.70971	0.00016
15.61	0.70995	0.70974	0.00015
15.56	0.70926	0.70967	0.00018
15.52	0.70981	0.70972	0.00015
15.48	0.70946	0.70971	0.00015
15.43	0.70980	0.70972	0.00014
15.39	0.71001	0.70968	0.00016
15.35	0.70988	0.70965	0.00014
15.30	0.70965	0.70968	0.00017
15.26	0.70986	0.70968	0.00017
15.21	0.70977	0.70969	0.00017
15.17	0.70917	0.70971	0.00018
15.13	0.70978	0.70975	0.00014
15.08	0.70972	0.70971	0.00016
15.04	0.70960	0.70971	0.00016
15.00	0.70937	0.70972	0.00016
14.95	0.70966	0.70976	0.00014
14.91	0.71018	0.70978	0.00014
14.86	0.70970	0.70980	0.00016
14.82	0.70990	0.70987	0.00021
14.78	0.70999	0.70984	0.00021
14.73	0.70959	0.70981	0.00021
14.69	0.70935	0.70983	0.00021
14.65	0.70971	0.70984	0.00020
14.60	0.70979	0.70984	0.00020
14.56	0.70971	0.70986	0.00020
14.52	0.70991	0.70980	0.00025
14.47	0.71033	0.70976	0.00025
14.43	0.71044	0.70970	0.00022
14.38	0.70959	0.70962	0.00014
14.34	0.70972	0.70961	0.00015
14.30	0.70979	0.70963	0.00016
14.25	0.70947	0.70961	0.00016
14.21	0.70967	0.70963	0.00015
14.17	0.70997	0.70966	0.00016
14.12	0.70913	0.70969	0.00020
14.08	0.70949	0.70978	0.00017
14.03	0.70975	0.70980	0.00016
13.99	0.70966	0.70982	0.00016
13.95	0.70944	0.70983	0.00016
13.90	0.70993	0.70985	0.00014
13.86	0.70962	0.70984	0.00014
13.82	0.70965	0.70981	0.00017
13.77	0.70993	0.70983	0.00016
13.73	0.71028	0.70979	0.00018
13.68	0.71011	0.70971	0.00015

13.64	0.70969	0.70969	0.00013
13.60	0.70990	0.70971	0.00014
13.55	0.70976	0.70966	0.00014
13.51	0.70968	0.70966	0.00014
13.47	0.70978	0.70969	0.00016
13.42	0.70932	0.70965	0.00017
13.38	0.70990	0.70971	0.00016
13.33	0.70945	0.70969	0.00016
13.29	0.70947	0.70971	0.00015
13.25	0.70992	0.70973	0.00014
13.20	0.70989	0.70972	0.00014
13.16	0.70946	0.70968	0.00014
13.12	0.70974	0.70970	0.00013
13.07	0.71001	0.70970	0.00013
13.03	0.70932	0.70969	0.00011
12.98	0.70993	0.70979	0.00014
12.94	0.70969	0.70978	0.00014
12.90	0.70968	0.70980	0.00014
12.85	0.70962	0.70978	0.00016
12.81	0.70986	0.70980	0.00015
12.77	0.70954	0.70980	0.00015
12.72	0.70961	0.70981	0.00014
12.68	0.70978	0.70981	0.00015
12.64	0.70983	0.70973	0.00021
12.59	0.71032	0.70974	0.00021
12.55	0.70986	0.70971	0.00018
12.50	0.70994	0.70969	0.00018
12.46	0.70946	0.70965	0.00017
12.42	0.70979	0.70969	0.00017
12.37	0.70986	0.70969	0.00017
12.33	0.70970	0.70967	0.00016
12.29	0.70953	0.70969	0.00017
12.24	0.70906	0.70969	0.00017
12.20	0.70989	0.70974	0.00010
12.15	0.70999	0.70973	0.00010
12.11	0.70966	0.70968	0.00008
12.07	0.70956	0.70967	0.00009
12.02	0.70987	0.70971	0.00010
11.98	0.70978	0.70974	0.00013
11.94	0.70971	0.70970	0.00015
11.89	0.70986	0.70968	0.00015
11.85	0.70958	0.70968	0.00015
11.80	0.70952	0.70973	0.00017
11.76	0.70973	0.70973	0.00017
11.72	0.70957	0.70971	0.00017
11.67	0.70949	0.70978	0.00019
11.63	0.70996	0.70979	0.00019
11.59	0.71016	0.70976	0.00019
11.54	0.70942	0.70971	0.00017
11.50	0.70954	0.70977	0.00016

11.45	0.70979	0.70982	0.00016
11.41	0.71009	0.70980	0.00017
11.37	0.70955	0.70975	0.00016
11.32	0.70957	0.70974	0.00016
11.28	0.71023	0.70973	0.00017
11.24	0.70956	0.70970	0.00014
11.19	0.70966	0.70967	0.00016
11.15	0.70970	0.70965	0.00016
11.11	0.71003	0.70965	0.00016
11.06	0.71002	0.70957	0.00016
11.02	0.70956	0.70954	0.00013
10.97	0.70964	0.70956	0.00014
10.93	0.70948	0.70954	0.00015
10.89	0.70946	0.70961	0.00020
10.84	0.70991	0.70967	0.00021
10.80	0.70930	0.70966	0.00020
10.76	0.70943	0.70978	0.00025
10.71	0.70969	0.70982	0.00023
10.67	0.70921	0.70984	0.00023
10.62	0.70971	0.70987	0.00020
10.58	0.70983	0.70985	0.00021
10.54	0.70942	0.70985	0.00021
10.49	0.71020	0.70991	0.00019
10.45	0.71000	0.70985	0.00019
10.41	0.70978	0.70984	0.00018
10.36	0.71051	0.70988	0.00020
10.32	0.70987	0.70974	0.00019
10.27	0.70986	0.70971	0.00019
10.23	0.70950	0.70970	0.00019
10.19	0.70957	0.70973	0.00018
10.14	0.70976	0.70973	0.00018
10.10	0.71004	0.70977	0.00021
10.06	0.70957	0.70977	0.00021
10.01	0.70992	0.70981	0.00021
9.97	0.71016	0.70979	0.00021
9.92	0.70913	0.70978	0.00020
9.88	0.70963	0.70980	0.00017
9.84	0.70971	0.70982	0.00016
9.79	0.70982	0.70981	0.00017
9.75	0.70951	0.70982	0.00017
9.71	0.71019	0.70983	0.00017
9.66	0.71005	0.70979	0.00015
9.62	0.71002	0.70977	0.00014
9.57	0.70972	0.70976	0.00013
9.53	0.71003	0.70981	0.00016
9.49	0.70935	0.70979	0.00015
9.44	0.70981	0.70983	0.00011
9.40	0.70961	0.70984	0.00011
9.36	0.70997	0.70982	0.00014
9.31	0.70956	0.70976	0.00016

9.27	0.70982	0.70980	0.00015
9.23	0.70984	0.70977	0.00016
9.18	0.70988	0.70978	0.00016
9.14	0.71020	0.70980	0.00017
9.09	0.70981	0.70974	0.00015
9.05	0.70983	0.70977	0.00016
9.01	0.70991	0.70979	0.00017
8.96	0.70940	0.70980	0.00017
8.92	0.70939	0.70984	0.00015
8.88	0.70991	0.70992	0.00013
8.83	0.70956	0.70992	0.00013
8.79	0.70994	0.70996	0.00010
8.74	0.71004	0.70996	0.00010
8.70	0.70963	0.70995	0.00010
8.66	0.71006	0.70996	0.00008
8.61	0.71006	0.70992	0.00010
8.57	0.71000	0.70987	0.00011
8.53	0.70986	0.70986	0.00011
8.48	0.71018	0.70986	0.00011
8.44	0.70989	0.70981	0.00008
8.39	0.70990	0.70980	0.00008
8.35	0.71002	0.70977	0.00009
8.31	0.70989	0.70972	0.00008
8.26	0.70976	0.70975	0.00013
8.22	0.70959	0.70975	0.00013
8.18	0.70965	0.70976	0.00012
8.09	0.70984	0.70976	0.00012
8.04	0.70975	0.70971	0.00014
8.00	0.70982	0.70971	0.00014
7.96	0.70957	0.70971	0.00014
7.91	0.70956	0.70969	0.00015
7.87	0.71021	0.70970	0.00015
7.83	0.70979	0.70966	0.00010
7.78	0.70966	0.70963	0.00010
7.74	0.70979	0.70961	0.00010
7.69	0.70963	0.70958	0.00010
7.65	0.70937	0.70960	0.00011
7.61	0.70974	0.70967	0.00012
7.56	0.70976	0.70966	0.00012
7.52	0.70939	0.70969	0.00015
7.48	0.70964	0.70972	0.00013
7.43	0.70980	0.70975	0.00014
7.39	0.70950	0.70979	0.00016
7.35	0.70951	0.70978	0.00017
7.30	0.70950	0.70980	0.00016
7.26	0.70982	0.70983	0.00014
7.21	0.71002	0.70987	0.00016
7.17	0.70964	0.70980	0.00018
7.13	0.71008	0.70981	0.00018
7.08	0.70968	0.70980	0.00017

7.04	0.70996	0.70981	0.00017
7.00	0.71017	0.70980	0.00017
6.95	0.70946	0.70978	0.00015
6.91	0.70964	0.70982	0.00014
6.86	0.70985	0.70986	0.00014
6.82	0.71019	0.70987	0.00014
6.78	0.70936	0.70982	0.00012
6.73	0.70973	0.70984	0.00010
6.69	0.70993	0.70988	0.00012
6.65	0.70979	0.70986	0.00013
6.60	0.70992	0.70988	0.00013
6.56	0.70990	0.70986	0.00013
6.51	0.70985	0.70982	0.00014
6.47	0.71006	0.70979	0.00016
6.43	0.70999	0.70977	0.00015
6.38	0.70970	0.70974	0.00014
6.34	0.70953	0.70974	0.00014
6.30	0.71019	0.70975	0.00013
6.25	0.70965	0.70973	0.00011
6.21	0.70997	0.70974	0.00011
6.16	0.70976	0.70968	0.00012
6.12	0.70954	0.70969	0.00012
6.08	0.70951	0.70970	0.00012
6.03	0.70987	0.70973	0.00011
5.99	0.70965	0.70973	0.00011
5.95	0.70967	0.70972	0.00012
5.90	0.70967	0.70976	0.00014
5.86	0.71005	0.70979	0.00014
5.81	0.70965	0.70976	0.00012
5.77	0.70938	0.70976	0.00013
5.73	0.70987	0.70977	0.00011
5.68	0.70966	0.70978	0.00012
5.64	0.70983	0.70977	0.00012
5.60	0.70986	0.70979	0.00013
5.55	0.70958	0.70976	0.00013
5.51	0.71008	0.70984	0.00016
5.47	0.70993	0.70981	0.00015
5.42	0.70978	0.70978	0.00015
5.38	0.70962	0.70980	0.00016
5.33	0.70949	0.70986	0.00017
5.29	0.70994	0.70991	0.00015
5.25	0.70962	0.70990	0.00015
5.20	0.70998	0.70995	0.00014
5.16	0.70963	0.70992	0.00014
5.12	0.71032	0.70997	0.00013
5.07	0.70979	0.70995	0.00012
5.03	0.70965	0.70997	0.00011
4.98	0.71000	0.70997	0.00011
4.94	0.71018	0.70999	0.00012
4.90	0.70996	0.70994	0.00012

4.85	0.70991	0.70996	0.00013
4.81	0.71004	0.71002	0.00016
4.77	0.70971	0.70996	0.00019
4.72	0.71014	0.70999	0.00018
4.68	0.71012	0.71001	0.00019
4.63	0.70996	0.70999	0.00019
4.59	0.70963	0.71000	0.00019
4.55	0.71022	0.71001	0.00018
4.50	0.70976	0.71000	0.00018
4.46	0.71016	0.70999	0.00019
4.42	0.71042	0.70991	0.00022
4.37	0.70948	0.70985	0.00019
4.33	0.71006	0.70986	0.00018
4.28	0.71033	0.70981	0.00019
4.24	0.70992	0.70972	0.00015
4.20	0.70999	0.70971	0.00015
4.15	0.70981	0.70967	0.00014
4.11	0.71012	0.70964	0.00014
4.07	0.70964	0.70959	0.00009
4.02	0.70934	0.70957	0.00009
3.98	0.70982	0.70960	0.00008
3.93	0.70958	0.70958	0.00006
3.89	0.70953	0.70959	0.00006
3.85	0.70950	0.70959	0.00006
3.80	0.70979	0.70958	0.00007
3.76	0.70962	0.70957	0.00006
3.72	0.70947	0.70954	0.00008
3.67	0.70958	0.70953	0.00008
3.63	0.70949	0.70946	0.00015
3.59	0.70962	0.70945	0.00015
3.54	0.70963	0.70942	0.00014
3.50	0.70965	0.70943	0.00015
3.45	0.70955	0.70939	0.00015
3.41	0.70939	0.70941	0.00016
3.37	0.70969	0.70943	0.00016
3.32	0.70931	0.70942	0.00016
3.28	0.70939	0.70944	0.00016
3.24	0.70892	0.70941	0.00017
3.19	0.70935	0.70944	0.00014
3.15	0.70932	0.70940	0.00017
3.10	0.70975	0.70942	0.00016
3.06	0.70924	0.70941	0.00016
3.02	0.70973	0.70945	0.00016
2.97	0.70959	0.70945	0.00016
2.93	0.70961	0.70941	0.00017
2.89	0.70946	0.70938	0.00016
2.84	0.70917	0.70935	0.00017
2.80	0.70917	0.70940	0.00018
2.75	0.70899	0.70942	0.00017
2.71	0.70946	0.70949	0.00015

2.67	0.70968	0.70947	0.00016
2.62	0.70966	0.70946	0.00016
2.58	0.70975	0.70944	0.00015
2.54	0.70921	0.70945	0.00016
2.49	0.70922	0.70948	0.00015
2.45	0.70918	0.70948	0.00016
2.40	0.70971	0.70951	0.00014
2.36	0.70931	0.70951	0.00014
2.32	0.70973	0.70951	0.00014
2.27	0.70924	0.70950	0.00013
2.23	0.70960	0.70951	0.00012
2.19	0.70945	0.70952	0.00013
2.14	0.70983	0.70950	0.00015
2.10	0.70956	0.70947	0.00013
2.05	0.70915	0.70946	0.00013
2.01	0.70949	0.70951	0.00011
1.97	0.70970	0.70953	0.00012
1.92	0.70935	0.70951	0.00011
1.88	0.70960	0.70955	0.00011
1.84	0.70935	0.70956	0.00012
1.79	0.70976	0.70959	0.00011
1.75	0.70921	0.70956	0.00010
1.71	0.70953	0.70960	0.00007
1.66	0.70942	0.70957	0.00011
1.62	0.70965	0.70957	0.00010
1.57	0.70970	0.70956	0.00010
1.53	0.70955	0.70956	0.00010
1.49	0.70970	0.70953	0.00011
1.44	0.70975	0.70950	0.00011
1.40	0.70959	0.70946	0.00010
1.36	0.70943	0.70945	0.00009
1.31	0.70964	0.70943	0.00010
1.27	0.70921	0.70943	0.00010
1.22	0.70948	0.70943	0.00010
1.18	0.70958	0.70947	0.00015
1.14	0.70964	0.70945	0.00015
1.09	0.70930	0.70941	0.00015
1.05	0.70942	0.70946	0.00017
1.01	0.70931	0.70946	0.00017
0.96	0.70945	0.70947	0.00017
0.92	0.70923	0.70945	0.00017
0.87	0.70965	0.70945	0.00017
0.83	0.70921	0.70941	0.00017
0.79	0.70995	0.70944	0.00016
0.74	0.70930	0.70932	0.00017
0.70	0.70926	0.70933	0.00017
0.66	0.70985	0.70938	0.00019
0.61	0.70935	0.70930	0.00017
0.57	0.70940	0.70932	0.00017
0.52	0.70930	0.70932	0.00017

0.48	0.70924	0.70934	0.00018
0.44	0.70926	0.70936	0.00018
0.39	0.70953	0.70937	0.00018
0.35	0.70876	0.70935	0.00019
0.31	0.70939	0.70944	0.00013
0.26	0.70975	0.70944	0.00014
0.22	0.70907	0.70938	0.00012
0.17	0.70947	0.70946	0.00005
0.13	0.70939	0.70946	0.00006
0.09	0.70956	0.70949	0.00006
0.04	0.70942		

ARB 25.2.1 (M3)

Distance from cervix (mm)	$^{87}Sr/^{86}Sr$	10 point mov. average	2 SE on mov. average
44.50	0.70852	0.70856	0.00007
44.45	0.70878	0.70859	0.00008
44.39	0.70844	0.70855	0.00007
44.34	0.70846	0.70856	0.00007
44.29	0.70846	0.70860	0.00008
44.23	0.70847	0.70861	0.00007
44.18	0.70863	0.70861	0.00007
44.12	0.70869	0.70861	0.00007
44.07	0.70863	0.70862	0.00007
44.02	0.70858	0.70862	0.00007
43.96	0.70874	0.70866	0.00011
43.91	0.70843	0.70864	0.00011
43.86	0.70856	0.70866	0.00010
43.80	0.70878	0.70869	0.00010
43.75	0.70863	0.70866	0.00010
43.69	0.70845	0.70865	0.00010
43.64	0.70865	0.70869	0.00010
43.59	0.70872	0.70872	0.00011
43.53	0.70861	0.70871	0.00011
43.48	0.70900	0.70871	0.00011
43.43	0.70853	0.70866	0.00010
43.37	0.70865	0.70866	0.00010
43.32	0.70885	0.70869	0.00012
43.27	0.70853	0.70865	0.00012
43.21	0.70858	0.70867	0.00012
43.16	0.70884	0.70865	0.00013
43.10	0.70891	0.70865	0.00013
43.05	0.70857	0.70863	0.00012
43.00	0.70868	0.70864	0.00012
42.94	0.70842	0.70862	0.00012
42.89	0.70854	0.70864	0.00011
42.84	0.70899	0.70863	0.00011
42.78	0.70847	0.70861	0.00008
42.73	0.70868	0.70862	0.00008
42.67	0.70842	0.70860	0.00008
42.62	0.70878	0.70859	0.00009
42.57	0.70877	0.70859	0.00009
42.51	0.70865	0.70859	0.00009
42.46	0.70850	0.70864	0.00013
42.41	0.70858	0.70867	0.00013
42.35	0.70848	0.70866	0.00014
42.30	0.70874	0.70867	0.00013
42.25	0.70862	0.70866	0.00013
42.19	0.70850	0.70865	0.00014
42.14	0.70834	0.70866	0.00013
42.08	0.70875	0.70868	0.00012
42.03	0.70880	0.70867	0.00012

41.98	0.70906	0.70865	0.00012
41.92	0.70884	0.70861	0.00007
41.87	0.70846	0.70859	0.00006
41.82	0.70862	0.70856	0.00008
41.76	0.70863	0.70856	0.00008
41.71	0.70850	0.70854	0.00008
41.66	0.70865	0.70856	0.00008
41.60	0.70845	0.70856	0.00009
41.55	0.70865	0.70858	0.00008
41.49	0.70866	0.70859	0.00009
41.44	0.70867	0.70858	0.00009
41.39	0.70856	0.70860	0.00011
41.33	0.70825	0.70865	0.00014
41.28	0.70856	0.70868	0.00010
41.23	0.70850	0.70868	0.00011
41.17	0.70862	0.70870	0.00010
41.12	0.70870	0.70869	0.00011
41.06	0.70862	0.70869	0.00011
41.01	0.70874	0.70867	0.00012
40.96	0.70857	0.70867	0.00012
40.90	0.70889	0.70868	0.00012
40.85	0.70903	0.70867	0.00011
40.80	0.70860	0.70861	0.00009
40.74	0.70853	0.70861	0.00009
40.69	0.70868	0.70863	0.00009
40.64	0.70850	0.70860	0.00010
40.58	0.70874	0.70863	0.00010
40.53	0.70839	0.70862	0.00010
40.47	0.70878	0.70864	0.00009
40.42	0.70868	0.70862	0.00008
40.37	0.70879	0.70863	0.00008
40.31	0.70840	0.70860	0.00008
40.26	0.70866	0.70860	0.00008
40.21	0.70870	0.70857	0.00009
40.15	0.70841	0.70857	0.00009
40.10	0.70872	0.70856	0.00010
40.04	0.70869	0.70851	0.00011
39.99	0.70858	0.70850	0.00010
39.94	0.70861	0.70852	0.00011
39.88	0.70872	0.70853	0.00012
39.83	0.70848	0.70853	0.00012
39.78	0.70843	0.70858	0.00015
39.72	0.70837	0.70861	0.00015
39.67	0.70867	0.70864	0.00014
39.62	0.70830	0.70865	0.00014
39.56	0.70825	0.70868	0.00012
39.51	0.70864	0.70873	0.00007
39.45	0.70871	0.70875	0.00007
39.40	0.70879	0.70871	0.00011
39.35	0.70864	0.70871	0.00011

39.29	0.70900	0.70872	0.00011
39.24	0.70872	0.70869	0.00009
39.19	0.70872	0.70865	0.00012
39.13	0.70870	0.70864	0.00012
39.08	0.70862	0.70863	0.00012
39.02	0.70871	0.70863	0.00012
38.97	0.70890	0.70862	0.00012
38.92	0.70833	0.70858	0.00010
38.86	0.70879	0.70860	0.00008
38.81	0.70866	0.70858	0.00007
38.76	0.70873	0.70856	0.00008
38.70	0.70832	0.70854	0.00007
38.65	0.70861	0.70858	0.00005
38.60	0.70866	0.70857	0.00005
38.54	0.70857	0.70858	0.00006
38.49	0.70863	0.70856	0.00007
38.43	0.70850	0.70855	0.00007
38.38	0.70855	0.70860	0.00011
38.33	0.70861	0.70859	0.00011
38.27	0.70842	0.70863	0.00013
38.22	0.70853	0.70869	0.00014
38.17	0.70869	0.70868	0.00014
38.11	0.70856	0.70870	0.00015
38.06	0.70875	0.70875	0.00016
38.00	0.70841	0.70875	0.00016
37.95	0.70851	0.70879	0.00014
37.90	0.70900	0.70881	0.00012
37.84	0.70849	0.70877	0.00012
37.79	0.70894	0.70879	0.00011
37.74	0.70900	0.70877	0.00011
37.68	0.70850	0.70873	0.00010
37.63	0.70890	0.70874	0.00009
37.58	0.70903	0.70874	0.00008
37.52	0.70875	0.70870	0.00005
37.47	0.70879	0.70871	0.00006
37.41	0.70876	0.70865	0.00011
37.36	0.70859	0.70865	0.00011
37.31	0.70867	0.70864	0.00011
37.25	0.70874	0.70865	0.00011
37.20	0.70858	0.70868	0.00014
37.15	0.70862	0.70869	0.00014
37.09	0.70883	0.70871	0.00014
37.04	0.70865	0.70868	0.00014
36.98	0.70885	0.70869	0.00014
36.93	0.70824	0.70867	0.00014
36.88	0.70869	0.70875	0.00012
36.82	0.70853	0.70876	0.00012
36.77	0.70873	0.70876	0.00012
36.72	0.70909	0.70876	0.00012
36.66	0.70862	0.70873	0.00009

36.61	0.70883	0.70873	0.00009
36.56	0.70860	0.70875	0.00010
36.50	0.70875	0.70875	0.00009
36.45	0.70861	0.70874	0.00010
36.39	0.70905	0.70876	0.00010
36.34	0.70878	0.70871	0.00007
36.29	0.70858	0.70869	0.00008
36.23	0.70875	0.70870	0.00007
36.18	0.70876	0.70868	0.00007
36.13	0.70865	0.70864	0.00010
36.07	0.70893	0.70863	0.00010
36.02	0.70868	0.70858	0.00008
35.97	0.70856	0.70858	0.00008
35.91	0.70881	0.70857	0.00008
35.86	0.70859	0.70856	0.00007
35.80	0.70858	0.70856	0.00007
35.75	0.70864	0.70857	0.00007
35.70	0.70863	0.70860	0.00010
35.64	0.70834	0.70860	0.00010
35.59	0.70851	0.70860	0.00010
35.54	0.70847	0.70862	0.00009
35.48	0.70862	0.70864	0.00009
35.43	0.70851	0.70866	0.00009
35.37	0.70871	0.70867	0.00009
35.32	0.70857	0.70866	0.00009
35.27	0.70872	0.70866	0.00009
35.21	0.70888	0.70864	0.00010
35.16	0.70867	0.70859	0.00009
35.11	0.70835	0.70858	0.00008
35.05	0.70866	0.70858	0.00008
35.00	0.70870	0.70856	0.00009
34.95	0.70880	0.70855	0.00009
34.89	0.70860	0.70853	0.00007
34.84	0.70864	0.70851	0.00007
34.78	0.70858	0.70850	0.00006
34.73	0.70848	0.70853	0.00009
34.68	0.70845	0.70857	0.00011
34.62	0.70852	0.70858	0.00011
34.57	0.70835	0.70860	0.00011
34.52	0.70842	0.70863	0.00009
34.46	0.70867	0.70863	0.00009
34.41	0.70858	0.70860	0.00011
34.35	0.70842	0.70859	0.00011
34.30	0.70856	0.70862	0.00010
34.25	0.70885	0.70863	0.00010
34.19	0.70884	0.70862	0.00010
34.14	0.70859	0.70862	0.00009
34.09	0.70872	0.70862	0.00009
34.03	0.70863	0.70859	0.00010
33.98	0.70842	0.70856	0.00011

33.93	0.70834	0.70855	0.00012
33.87	0.70854	0.70857	0.00011
33.82	0.70867	0.70855	0.00012
33.76	0.70868	0.70854	0.00011
33.71	0.70877	0.70852	0.00011
33.66	0.70878	0.70851	0.00010
33.60	0.70869	0.70849	0.00008
33.55	0.70835	0.70849	0.00009
33.50	0.70837	0.70850	0.00008
33.44	0.70835	0.70851	0.00008
33.39	0.70848	0.70852	0.00007
33.33	0.70837	0.70849	0.00010
33.28	0.70854	0.70851	0.00009
33.23	0.70854	0.70853	0.00011
33.17	0.70860	0.70850	0.00012
33.12	0.70858	0.70848	0.00012
33.07	0.70877	0.70847	0.00012
33.01	0.70844	0.70841	0.00011
32.96	0.70843	0.70841	0.00011
32.91	0.70847	0.70840	0.00011
32.85	0.70820	0.70841	0.00011
32.80	0.70853	0.70845	0.00011
32.74	0.70876	0.70843	0.00011
32.69	0.70821	0.70837	0.00008
32.64	0.70843	0.70837	0.00009
32.58	0.70841	0.70839	0.00010
32.53	0.70825	0.70840	0.00010
32.48	0.70839	0.70842	0.00010
32.42	0.70832	0.70841	0.00011
32.37	0.70853	0.70844	0.00012
32.31	0.70861	0.70843	0.00011
32.26	0.70833	0.70841	0.00011
32.21	0.70820	0.70842	0.00011
32.15	0.70820	0.70841	0.00012
32.10	0.70862	0.70844	0.00011
32.05	0.70850	0.70842	0.00010
31.99	0.70852	0.70843	0.00011
31.94	0.70823	0.70840	0.00011
31.89	0.70867	0.70842	0.00010
31.83	0.70847	0.70840	0.00009
31.78	0.70841	0.70839	0.00009
31.72	0.70838	0.70837	0.00010
31.67	0.70808	0.70838	0.00010
31.62	0.70852	0.70848	0.00014
31.56	0.70838	0.70846	0.00014
31.51	0.70861	0.70848	0.00014
31.46	0.70829	0.70848	0.00014
31.40	0.70841	0.70850	0.00014
31.35	0.70846	0.70852	0.00014
31.29	0.70834	0.70854	0.00014

31.24	0.70819	0.70854	0.00014
31.19	0.70855	0.70860	0.00012
31.13	0.70900	0.70859	0.00013
31.08	0.70840	0.70857	0.00011
31.03	0.70858	0.70859	0.00010
30.97	0.70861	0.70857	0.00011
30.92	0.70841	0.70855	0.00012
30.87	0.70865	0.70858	0.00012
30.81	0.70862	0.70858	0.00012
30.76	0.70835	0.70860	0.00014
30.70	0.70880	0.70864	0.00013
30.65	0.70842	0.70863	0.00012
30.60	0.70885	0.70866	0.00011
30.54	0.70863	0.70861	0.00011
30.49	0.70835	0.70864	0.00013
30.44	0.70838	0.70868	0.00011
30.38	0.70875	0.70870	0.00010
30.33	0.70861	0.70870	0.00010
30.28	0.70891	0.70869	0.00010
30.22	0.70873	0.70863	0.00011
30.17	0.70870	0.70863	0.00011
30.11	0.70869	0.70860	0.00012
30.06	0.70841	0.70861	0.00012
30.01	0.70893	0.70861	0.00011
29.95	0.70867	0.70853	0.00012
29.90	0.70856	0.70849	0.00013
29.85	0.70875	0.70845	0.00014
29.79	0.70854	0.70844	0.00013
29.74	0.70833	0.70846	0.00015
29.68	0.70872	0.70847	0.00014
29.63	0.70845	0.70844	0.00013
29.58	0.70872	0.70845	0.00013
29.52	0.70843	0.70842	0.00012
29.47	0.70815	0.70841	0.00012
29.42	0.70825	0.70844	0.00011
29.36	0.70819	0.70849	0.00012
29.31	0.70863	0.70856	0.00012
29.26	0.70874	0.70855	0.00012
29.20	0.70844	0.70856	0.00013
29.15	0.70839	0.70860	0.00014
29.09	0.70855	0.70864	0.00013
29.04	0.70841	0.70867	0.00013
28.99	0.70832	0.70870	0.00012
28.93	0.70843	0.70872	0.00009
28.88	0.70884	0.70874	0.00007
28.83	0.70882	0.70874	0.00007
28.77	0.70857	0.70871	0.00008
28.72	0.70887	0.70871	0.00008
28.66	0.70882	0.70868	0.00008
28.61	0.70882	0.70864	0.00009

28.56	0.70877	0.70861	0.00008
28.50	0.70873	0.70863	0.00010
28.45	0.70855	0.70860	0.00011
28.40	0.70862	0.70860	0.00011
28.34	0.70879	0.70862	0.00012
28.29	0.70854	0.70865	0.00015
28.24	0.70863	0.70866	0.00015
28.18	0.70849	0.70868	0.00015
28.13	0.70842	0.70870	0.00015
28.07	0.70857	0.70874	0.00014
28.02	0.70895	0.70873	0.00015
27.97	0.70839	0.70868	0.00015
27.91	0.70857	0.70873	0.00014
27.86	0.70884	0.70873	0.00014
27.81	0.70912	0.70870	0.00014
27.75	0.70860	0.70866	0.00011
27.70	0.70883	0.70869	0.00011
27.64	0.70871	0.70867	0.00011
27.59	0.70886	0.70865	0.00012
27.54	0.70844	0.70859	0.00012
27.48	0.70841	0.70858	0.00013
27.43	0.70889	0.70862	0.00013
27.38	0.70859	0.70857	0.00012
27.32	0.70856	0.70857	0.00012
27.27	0.70867	0.70858	0.00012
27.22	0.70889	0.70857	0.00012
27.16	0.70863	0.70854	0.00010
27.11	0.70849	0.70852	0.00009
27.05	0.70830	0.70852	0.00009
27.00	0.70838	0.70851	0.00011
26.95	0.70879	0.70852	0.00010
26.89	0.70840	0.70848	0.00009
26.84	0.70857	0.70850	0.00009
26.79	0.70871	0.70851	0.00009
26.73	0.70853	0.70849	0.00008
26.68	0.70857	0.70850	0.00008
26.62	0.70846	0.70851	0.00009
26.57	0.70850	0.70854	0.00010
26.52	0.70819	0.70856	0.00011
26.46	0.70850	0.70861	0.00007
26.41	0.70841	0.70865	0.00009
26.36	0.70853	0.70867	0.00008
26.30	0.70871	0.70870	0.00008
26.25	0.70852	0.70868	0.00008
26.20	0.70857	0.70867	0.00009
26.14	0.70870	0.70868	0.00009
26.09	0.70872	0.70870	0.00010
26.03	0.70872	0.70867	0.00011
25.98	0.70870	0.70864	0.00012
25.93	0.70894	0.70859	0.00016

25.87	0.70856	0.70856	0.00014
25.82	0.70881	0.70863	0.00020
25.77	0.70859	0.70858	0.00020
25.71	0.70841	0.70860	0.00021
25.66	0.70867	0.70866	0.00021
25.60	0.70888	0.70862	0.00022
25.55	0.70842	0.70856	0.00023
25.50	0.70843	0.70858	0.00022
25.44	0.70816	0.70862	0.00022
25.39	0.70865	0.70868	0.00020
25.34	0.70928	0.70866	0.00021
25.28	0.70831	0.70860	0.00015
25.23	0.70880	0.70862	0.00014
25.18	0.70896	0.70860	0.00013
25.12	0.70832	0.70856	0.00011
25.07	0.70828	0.70863	0.00014
25.01	0.70859	0.70869	0.00011
24.96	0.70880	0.70868	0.00012
24.91	0.70876	0.70866	0.00012
24.85	0.70847	0.70867	0.00012
24.80	0.70867	0.70870	0.00012
24.75	0.70850	0.70868	0.00013
24.69	0.70868	0.70868	0.00013
24.64	0.70852	0.70867	0.00013
24.59	0.70907	0.70870	0.00013
24.53	0.70880	0.70867	0.00010
24.48	0.70854	0.70864	0.00010
24.42	0.70857	0.70866	0.00010
24.37	0.70887	0.70867	0.00010
24.32	0.70882	0.70865	0.00010
24.26	0.70840	0.70866	0.00010
24.21	0.70853	0.70871	0.00009
24.16	0.70858	0.70871	0.00009
24.10	0.70882	0.70873	0.00008
24.05	0.70878	0.70872	0.00008
23.99	0.70851	0.70871	0.00008
23.94	0.70876	0.70871	0.00008
23.89	0.70858	0.70870	0.00008
23.83	0.70876	0.70868	0.00009
23.78	0.70891	0.70866	0.00010
23.73	0.70884	0.70864	0.00009
23.67	0.70858	0.70864	0.00009
23.62	0.70878	0.70867	0.00010
23.57	0.70872	0.70864	0.00010
23.51	0.70871	0.70861	0.00010
23.46	0.70852	0.70860	0.00010
23.40	0.70858	0.70861	0.00010
23.35	0.70845	0.70862	0.00010
23.30	0.70847	0.70863	0.00010
23.24	0.70873	0.70862	0.00010

23.19	0.70884	0.70863	0.00011
23.14	0.70888	0.70860	0.00010
23.08	0.70851	0.70857	0.00009
23.03	0.70842	0.70861	0.00010
22.97	0.70859	0.70865	0.00010
22.92	0.70863	0.70863	0.00011
22.87	0.70869	0.70864	0.00011
22.81	0.70853	0.70864	0.00011
22.76	0.70840	0.70864	0.00011
22.71	0.70884	0.70864	0.00011
22.65	0.70848	0.70863	0.00010
22.60	0.70863	0.70864	0.00009
22.55	0.70889	0.70862	0.00010
22.49	0.70878	0.70861	0.00009
22.44	0.70846	0.70859	0.00008
22.38	0.70873	0.70861	0.00008
22.33	0.70863	0.70859	0.00007
22.28	0.70855	0.70862	0.00010
22.22	0.70841	0.70863	0.00010
22.17	0.70873	0.70869	0.00012
22.12	0.70859	0.70868	0.00012
22.06	0.70844	0.70869	0.00012
22.01	0.70878	0.70872	0.00010
21.95	0.70862	0.70869	0.00012
21.90	0.70863	0.70868	0.00012
21.85	0.70856	0.70866	0.00013
21.79	0.70893	0.70869	0.00013
21.74	0.70860	0.70863	0.00014
21.69	0.70905	0.70862	0.00014
21.63	0.70855	0.70856	0.00011
21.58	0.70872	0.70855	0.00011
21.53	0.70877	0.70856	0.00012
21.47	0.70843	0.70854	0.00011
21.42	0.70861	0.70856	0.00011
21.36	0.70842	0.70855	0.00011
21.31	0.70884	0.70855	0.00011
21.26	0.70835	0.70853	0.00008
21.20	0.70848	0.70857	0.00009
21.15	0.70846	0.70854	0.00011
21.10	0.70842	0.70854	0.00011
21.04	0.70883	0.70857	0.00011
20.99	0.70855	0.70854	0.00010
20.93	0.70864	0.70855	0.00010
20.88	0.70846	0.70855	0.00010
20.83	0.70848	0.70859	0.00012
20.77	0.70858	0.70860	0.00012
20.72	0.70877	0.70862	0.00012
20.67	0.70822	0.70862	0.00012
20.61	0.70844	0.70865	0.00008
20.56	0.70872	0.70868	0.00007

20.51	0.70858	0.70865	0.00009
20.45	0.70866	0.70862	0.00012
20.40	0.70857	0.70865	0.00013
20.34	0.70888	0.70866	0.00013
20.29	0.70856	0.70864	0.00012
20.24	0.70877	0.70865	0.00012
20.18	0.70876	0.70862	0.00013
20.13	0.70857	0.70861	0.00012
20.08	0.70877	0.70862	0.00012
20.02	0.70838	0.70861	0.00012
19.97	0.70829	0.70863	0.00011
19.91	0.70893	0.70866	0.00008
19.86	0.70870	0.70862	0.00006
19.81	0.70871	0.70859	0.00006
19.75	0.70866	0.70859	0.00006
19.70	0.70845	0.70858	0.00005
19.65	0.70864	0.70862	0.00008
19.59	0.70872	0.70862	0.00008
19.54	0.70866	0.70860	0.00008
19.49	0.70854	0.70858	0.00008
19.43	0.70856	0.70859	0.00008
19.38	0.70854	0.70862	0.00010
19.32	0.70846	0.70864	0.00010
19.27	0.70864	0.70866	0.00010
19.22	0.70856	0.70863	0.00011
19.16	0.70892	0.70865	0.00011
19.11	0.70857	0.70863	0.00009
19.06	0.70851	0.70863	0.00009
19.00	0.70851	0.70864	0.00009
18.95	0.70858	0.70863	0.00009
18.90	0.70889	0.70863	0.00009
18.84	0.70880	0.70863	0.00009
18.79	0.70858	0.70862	0.00009
18.73	0.70842	0.70864	0.00009
18.68	0.70872	0.70864	0.00008
18.63	0.70871	0.70862	0.00009
18.57	0.70863	0.70859	0.00010
18.52	0.70856	0.70858	0.00010
18.47	0.70846	0.70857	0.00010
18.41	0.70852	0.70859	0.00010
18.36	0.70886	0.70858	0.00011
18.30	0.70876	0.70858	0.00010
18.25	0.70872	0.70853	0.00010
18.20	0.70850	0.70856	0.00013
18.14	0.70848	0.70858	0.00013
18.09	0.70837	0.70858	0.00013
18.04	0.70857	0.70861	0.00012
17.98	0.70842	0.70863	0.00013
17.93	0.70871	0.70869	0.00013
17.88	0.70844	0.70867	0.00014

17.82	0.70880	0.70868	0.00013
17.77	0.70833	0.70872	0.00016
17.71	0.70895	0.70875	0.00014
17.66	0.70870	0.70873	0.00013
17.61	0.70850	0.70875	0.00013
17.55	0.70869	0.70878	0.00012
17.50	0.70880	0.70877	0.00013
17.45	0.70895	0.70874	0.00014
17.39	0.70850	0.70871	0.00013
17.34	0.70859	0.70875	0.00013
17.28	0.70916	0.70873	0.00015
17.23	0.70864	0.70867	0.00011
17.18	0.70877	0.70864	0.00013
17.12	0.70888	0.70864	0.00013
17.07	0.70883	0.70861	0.00012
17.02	0.70858	0.70861	0.00012
16.96	0.70849	0.70865	0.00015
16.91	0.70863	0.70866	0.00014
16.86	0.70891	0.70866	0.00014
16.80	0.70837	0.70862	0.00013
16.75	0.70862	0.70865	0.00012
16.69	0.70829	0.70866	0.00012
16.64	0.70877	0.70871	0.00009
16.59	0.70865	0.70868	0.00010
16.53	0.70875	0.70869	0.00010
16.48	0.70904	0.70868	0.00010
16.43	0.70856	0.70865	0.00007
16.37	0.70862	0.70871	0.00011
16.32	0.70851	0.70876	0.00014
16.26	0.70873	0.70877	0.00013
16.21	0.70868	0.70875	0.00014
16.16	0.70877	0.70879	0.00015
16.10	0.70846	0.70878	0.00015
16.05	0.70877	0.70878	0.00014
16.00	0.70871	0.70875	0.00016
15.94	0.70872	0.70872	0.00017
15.89	0.70913	0.70873	0.00017
15.84	0.70910	0.70866	0.00015
15.78	0.70864	0.70865	0.00014
15.73	0.70853	0.70867	0.00014
15.67	0.70905	0.70868	0.00014
15.62	0.70867	0.70861	0.00013
15.57	0.70850	0.70862	0.00013
15.51	0.70846	0.70862	0.00013
15.46	0.70843	0.70863	0.00012
15.41	0.70873	0.70864	0.00012
15.35	0.70851	0.70865	0.00012
15.30	0.70901	0.70866	0.00012
15.24	0.70879	0.70860	0.00010
15.19	0.70864	0.70857	0.00009

15.14	0.70834	0.70857	0.00009
15.08	0.70873	0.70860	0.00008
15.03	0.70860	0.70858	0.00007
14.98	0.70852	0.70860	0.00008
14.92	0.70856	0.70862	0.00008
14.87	0.70884	0.70866	0.00011
14.82	0.70855	0.70867	0.00012
14.76	0.70846	0.70870	0.00012
14.71	0.70845	0.70875	0.00012
14.65	0.70863	0.70876	0.00010
14.60	0.70867	0.70878	0.00010
14.55	0.70851	0.70877	0.00011
14.49	0.70879	0.70878	0.00009
14.44	0.70870	0.70876	0.00010
14.39	0.70896	0.70876	0.00010
14.33	0.70895	0.70877	0.00011
14.28	0.70890	0.70874	0.00010
14.22	0.70890	0.70872	0.00010
14.17	0.70862	0.70871	0.00009
14.12	0.70881	0.70873	0.00009
14.06	0.70851	0.70869	0.00010
14.01	0.70869	0.70871	0.00009
13.96	0.70856	0.70873	0.00010
13.90	0.70873	0.70874	0.00010
13.85	0.70901	0.70873	0.00010
13.80	0.70867	0.70871	0.00008
13.74	0.70875	0.70867	0.00012
13.69	0.70875	0.70865	0.00012
13.63	0.70880	0.70865	0.00012
13.58	0.70845	0.70860	0.00013
13.53	0.70868	0.70865	0.00014
13.47	0.70893	0.70867	0.00015
13.42	0.70860	0.70865	0.00014
13.37	0.70867	0.70867	0.00014
13.31	0.70879	0.70870	0.00015
13.26	0.70829	0.70871	0.00016
13.21	0.70855	0.70874	0.00013
13.15	0.70876	0.70877	0.00012
13.10	0.70830	0.70877	0.00012
13.04	0.70892	0.70880	0.00008
12.99	0.70889	0.70876	0.00009
12.94	0.70871	0.70872	0.00011
12.88	0.70885	0.70871	0.00011
12.83	0.70894	0.70869	0.00010
12.78	0.70889	0.70864	0.00009
12.72	0.70861	0.70864	0.00009
12.67	0.70888	0.70866	0.00009
12.61	0.70869	0.70865	0.00008
12.56	0.70862	0.70864	0.00008
12.51	0.70856	0.70863	0.00008

12.45	0.70843	0.70867	0.00010
12.40	0.70862	0.70869	0.00009
12.35	0.70863	0.70869	0.00009
12.29	0.70852	0.70868	0.00010
12.24	0.70887	0.70869	0.00009
12.19	0.70875	0.70865	0.00010
12.13	0.70879	0.70866	0.00011
12.08	0.70858	0.70868	0.00012
12.02	0.70856	0.70870	0.00012
11.97	0.70893	0.70868	0.00013
11.92	0.70869	0.70862	0.00014
11.86	0.70859	0.70861	0.00014
11.81	0.70850	0.70866	0.00016
11.76	0.70868	0.70865	0.00016
11.70	0.70838	0.70865	0.00016
11.65	0.70887	0.70868	0.00015
11.59	0.70897	0.70864	0.00015
11.54	0.70877	0.70863	0.00015
11.49	0.70843	0.70861	0.00015
11.43	0.70830	0.70862	0.00014
11.38	0.70861	0.70865	0.00012
11.33	0.70903	0.70863	0.00013
11.27	0.70844	0.70859	0.00009
11.22	0.70870	0.70859	0.00009
11.17	0.70868	0.70860	0.00010
11.11	0.70845	0.70861	0.00011
11.06	0.70892	0.70863	0.00010
11.00	0.70854	0.70857	0.00009
10.95	0.70855	0.70859	0.00010
10.90	0.70855	0.70860	0.00010
10.84	0.70848	0.70863	0.00011
10.79	0.70856	0.70865	0.00010
10.74	0.70846	0.70864	0.00011
10.68	0.70878	0.70865	0.00011
10.63	0.70886	0.70864	0.00010
10.57	0.70857	0.70861	0.00009
10.52	0.70839	0.70863	0.00009
10.47	0.70874	0.70867	0.00008
10.41	0.70863	0.70868	0.00009
10.36	0.70884	0.70870	0.00009
10.31	0.70870	0.70866	0.00010
10.25	0.70841	0.70863	0.00011
10.20	0.70853	0.70870	0.00013
10.15	0.70873	0.70873	0.00013
10.09	0.70861	0.70873	0.00013
10.04	0.70872	0.70876	0.00013
9.98	0.70879	0.70879	0.00014
9.93	0.70886	0.70875	0.00015
9.88	0.70879	0.70875	0.00015
9.82	0.70847	0.70877	0.00016

9.77	0.70838	0.70877	0.00016
9.72	0.70908	0.70880	0.00013
9.66	0.70886	0.70878	0.00012
9.61	0.70874	0.70879	0.00013
9.55	0.70887	0.70878	0.00013
9.50	0.70903	0.70876	0.00014
9.45	0.70845	0.70870	0.00014
9.39	0.70885	0.70873	0.00013
9.34	0.70894	0.70877	0.00017
9.29	0.70848	0.70875	0.00016
9.23	0.70868	0.70879	0.00015
9.18	0.70885	0.70880	0.00015
9.13	0.70903	0.70878	0.00015
9.07	0.70866	0.70873	0.00015
9.02	0.70859	0.70876	0.00015
8.96	0.70842	0.70878	0.00014
8.91	0.70875	0.70879	0.00014
8.86	0.70927	0.70877	0.00014
8.80	0.70876	0.70870	0.00010
8.75	0.70894	0.70871	0.00010
8.70	0.70872	0.70872	0.00011
8.64	0.70864	0.70868	0.00013
8.59	0.70860	0.70868	0.00013
8.53	0.70888	0.70870	0.00013
8.48	0.70888	0.70869	0.00013
8.43	0.70850	0.70866	0.00013
8.37	0.70858	0.70868	0.00012
8.32	0.70852	0.70868	0.00012
8.27	0.70883	0.70873	0.00013
8.21	0.70903	0.70872	0.00013
8.16	0.70838	0.70868	0.00010
8.11	0.70857	0.70872	0.00008
8.05	0.70879	0.70872	0.00009
8.00	0.70886	0.70869	0.00009
7.94	0.70858	0.70866	0.00009
7.89	0.70866	0.70870	0.00011
7.84	0.70862	0.70867	0.00013
7.78	0.70897	0.70866	0.00013
7.73	0.70872	0.70861	0.00012
7.68	0.70870	0.70861	0.00012
7.62	0.70878	0.70860	0.00011
7.57	0.70851	0.70857	0.00011
7.52	0.70852	0.70859	0.00011
7.46	0.70850	0.70861	0.00011
7.41	0.70900	0.70865	0.00012
7.35	0.70836	0.70866	0.00013
7.30	0.70852	0.70871	0.00012
7.25	0.70853	0.70874	0.00011
7.19	0.70869	0.70878	0.00011
7.14	0.70859	0.70880	0.00011

7.09	0.70848	0.70878	0.00013
7.03	0.70873	0.70879	0.00012
6.98	0.70870	0.70880	0.00012
6.92	0.70892	0.70880	0.00012
6.87	0.70906	0.70878	0.00012
6.82	0.70885	0.70874	0.00010
6.76	0.70886	0.70873	0.00010
6.71	0.70893	0.70872	0.00009
6.66	0.70886	0.70867	0.00009
6.60	0.70839	0.70865	0.00008
6.55	0.70862	0.70868	0.00005
6.50	0.70878	0.70872	0.00007
6.44	0.70875	0.70871	0.00007
6.39	0.70867	0.70872	0.00008
6.33	0.70872	0.70873	0.00008
6.28	0.70874	0.70875	0.00009
6.23	0.70871	0.70875	0.00009
6.17	0.70850	0.70876	0.00009
6.12	0.70865	0.70878	0.00007
6.07	0.70868	0.70879	0.00006
6.01	0.70896	0.70878	0.00007
5.96	0.70870	0.70878	0.00006
5.90	0.70890	0.70878	0.00006
5.85	0.70874	0.70878	0.00006
5.80	0.70891	0.70875	0.00010
5.74	0.70876	0.70874	0.00009
5.69	0.70878	0.70873	0.00009
5.64	0.70872	0.70876	0.00011
5.58	0.70875	0.70872	0.00013
5.53	0.70862	0.70871	0.00013
5.48	0.70891	0.70874	0.00014
5.42	0.70874	0.70871	0.00013
5.37	0.70889	0.70871	0.00013
5.31	0.70839	0.70868	0.00013
5.26	0.70883	0.70868	0.00012
5.21	0.70870	0.70866	0.00012
5.15	0.70901	0.70865	0.00012
5.10	0.70840	0.70863	0.00009
5.05	0.70860	0.70864	0.00008
4.99	0.70890	0.70864	0.00008
4.94	0.70868	0.70863	0.00006
4.88	0.70868	0.70864	0.00008
4.83	0.70856	0.70865	0.00008
4.78	0.70843	0.70867	0.00008
4.72	0.70866	0.70872	0.00007
4.67	0.70862	0.70873	0.00008
4.62	0.70873	0.70877	0.00009
4.56	0.70849	0.70875	0.00011
4.51	0.70869	0.70878	0.00009
4.46	0.70871	0.70877	0.00010

4.40	0.70887	0.70875	0.00011
4.35	0.70874	0.70874	0.00011
4.29	0.70874	0.70874	0.00011
4.24	0.70891	0.70872	0.00011
4.19	0.70881	0.70870	0.00010
4.13	0.70905	0.70869	0.00010
4.08	0.70851	0.70864	0.00007
4.03	0.70877	0.70867	0.00007
3.97	0.70857	0.70866	0.00007
3.92	0.70853	0.70868	0.00007
3.86	0.70878	0.70865	0.00011
3.81	0.70872	0.70864	0.00010
3.76	0.70858	0.70865	0.00011
3.70	0.70870	0.70865	0.00011
3.65	0.70874	0.70867	0.00012
3.60	0.70856	0.70866	0.00012
3.54	0.70881	0.70867	0.00011
3.49	0.70859	0.70865	0.00011
3.44	0.70882	0.70867	0.00011
3.38	0.70826	0.70865	0.00011
3.33	0.70860	0.70871	0.00008
3.27	0.70880	0.70872	0.00008
3.22	0.70865	0.70868	0.00010
3.17	0.70890	0.70866	0.00011
3.11	0.70861	0.70865	0.00010
3.06	0.70862	0.70865	0.00010
3.01	0.70865	0.70869	0.00012
2.95	0.70881	0.70868	0.00012
2.90	0.70856	0.70866	0.00012
2.84	0.70890	0.70867	0.00012
2.79	0.70875	0.70867	0.00012
2.74	0.70841	0.70866	0.00011
2.68	0.70843	0.70867	0.00010
2.63	0.70881	0.70874	0.00012
2.58	0.70862	0.70871	0.00014
2.52	0.70895	0.70871	0.00013
2.47	0.70861	0.70870	0.00013
2.42	0.70853	0.70870	0.00012
2.36	0.70869	0.70870	0.00013
2.31	0.70888	0.70872	0.00013
2.25	0.70866	0.70869	0.00013
2.20	0.70855	0.70870	0.00013
2.15	0.70912	0.70869	0.00013
2.09	0.70844	0.70865	0.00009
2.04	0.70865	0.70868	0.00009
1.99	0.70882	0.70864	0.00012
1.93	0.70867	0.70861	0.00012
1.88	0.70849	0.70859	0.00012
1.83	0.70887	0.70860	0.00012
1.77	0.70858	0.70860	0.00011

1.72	0.70876	0.70858	0.00011
1.66	0.70847	0.70855	0.00011
1.61	0.70869	0.70857	0.00011
1.56	0.70877	0.70856	0.00010
1.50	0.70823	0.70854	0.00009
1.45	0.70853	0.70858	0.00007
1.40	0.70852	0.70857	0.00008
1.34	0.70860	0.70859	0.00009
1.29	0.70878	0.70859	0.00009
1.23	0.70842	0.70857	0.00008
1.18	0.70852	0.70858	0.00007
1.13	0.70866	0.70861	0.00009
1.07	0.70857	0.70861	0.00009
1.02	0.70852	0.70863	0.00009
0.97	0.70869	0.70863	0.00009
0.91	0.70837	0.70861	0.00009
0.86	0.70874	0.70866	0.00008
0.81	0.70867	0.70867	0.00009
0.75	0.70850	0.70871	0.00011
0.70	0.70853	0.70871	0.00011
0.64	0.70886	0.70871	0.00011
0.59	0.70867	0.70869	0.00011
0.54	0.70870	0.70869	0.00011
0.48	0.70854	0.70869	0.00011
0.43	0.70858	0.70871	0.00011
0.38	0.70883	0.70873	0.00012
0.32	0.70887	0.70872	0.00012
0.27	0.70902	0.70868	0.00013
0.21	0.70851	0.70860	0.00005
0.16	0.70857	0.70863	0.00003
0.11	0.70867	0.70866	0.00000
0.05	0.70866		

ARB 26.2.1 (M2?)

Distance from cervix (mm)	$^{87}Sr/^{86}Sr$	10 point mov. average	2 SE on mov. average
50.72	0.70899	0.70860	0.00018
50.67	0.70869	0.70851	0.00019
50.63	0.70881	0.70849	0.00018
50.59	0.70854	0.70849	0.00018
50.54	0.70817	0.70849	0.00018
50.50	0.70848	0.70851	0.00017
50.46	0.70888	0.70855	0.00018
50.41	0.70811	0.70856	0.00019
50.37	0.70853	0.70861	0.00017
50.33	0.70881	0.70860	0.00017
50.28	0.70811	0.70854	0.00018
50.24	0.70849	0.70860	0.00015
50.20	0.70877	0.70860	0.00015
50.16	0.70855	0.70856	0.00015
50.11	0.70834	0.70859	0.00016
50.07	0.70886	0.70865	0.00016
50.03	0.70899	0.70862	0.00015
49.98	0.70864	0.70858	0.00013
49.94	0.70848	0.70855	0.00014
49.90	0.70823	0.70858	0.00014
49.85	0.70867	0.70861	0.00012
49.81	0.70849	0.70860	0.00012
49.77	0.70838	0.70859	0.00013
49.72	0.70882	0.70860	0.00012
49.68	0.70894	0.70858	0.00011
49.64	0.70858	0.70857	0.00011
49.59	0.70855	0.70859	0.00011
49.55	0.70833	0.70862	0.00012
49.51	0.70877	0.70865	0.00011
49.46	0.70851	0.70862	0.00011
49.42	0.70858	0.70863	0.00010
49.38	0.70839	0.70865	0.00011
49.33	0.70846	0.70867	0.00009
49.29	0.70865	0.70867	0.00009
49.25	0.70890	0.70864	0.00012
49.20	0.70876	0.70861	0.00010
49.16	0.70884	0.70859	0.00009
49.12	0.70864	0.70856	0.00008
49.08	0.70847	0.70858	0.00009
49.03	0.70864	0.70855	0.00013
48.99	0.70873	0.70853	0.00013
48.95	0.70865	0.70853	0.00013
48.90	0.70842	0.70848	0.00015
48.86	0.70834	0.70849	0.00015
48.82	0.70861	0.70851	0.00014
48.77	0.70858	0.70851	0.00014
48.73	0.70855	0.70852	0.00015

48.69	0.70883	0.70852	0.00015
48.64	0.70813	0.70848	0.00013
48.60	0.70849	0.70852	0.00011
48.56	0.70872	0.70855	0.00012
48.51	0.70812	0.70852	0.00012
48.47	0.70851	0.70856	0.00008
48.43	0.70857	0.70859	0.00009
48.38	0.70857	0.70862	0.00010
48.34	0.70873	0.70862	0.00010
48.30	0.70856	0.70859	0.00011
48.25	0.70837	0.70862	0.00012
48.21	0.70861	0.70867	0.00011
48.17	0.70877	0.70865	0.00012
48.12	0.70838	0.70862	0.00013
48.08	0.70858	0.70864	0.00012
48.04	0.70880	0.70864	0.00012
48.00	0.70882	0.70859	0.00013
47.95	0.70862	0.70857	0.00012
47.91	0.70841	0.70856	0.00012
47.87	0.70888	0.70854	0.00013
47.82	0.70886	0.70851	0.00011
47.78	0.70843	0.70845	0.00008
47.74	0.70841	0.70849	0.00011
47.69	0.70862	0.70850	0.00010
47.65	0.70858	0.70848	0.00010
47.61	0.70829	0.70848	0.00010
47.56	0.70858	0.70851	0.00010
47.52	0.70853	0.70851	0.00010
47.48	0.70826	0.70847	0.00012
47.43	0.70850	0.70851	0.00011
47.39	0.70834	0.70852	0.00011
47.35	0.70879	0.70853	0.00010
47.30	0.70846	0.70853	0.00011
47.26	0.70845	0.70854	0.00011
47.22	0.70855	0.70858	0.00012
47.17	0.70867	0.70859	0.00012
47.13	0.70852	0.70858	0.00012
47.09	0.70817	0.70859	0.00012
47.04	0.70864	0.70863	0.00008
47.00	0.70855	0.70862	0.00008
46.96	0.70845	0.70864	0.00008
46.92	0.70885	0.70865	0.00007
46.87	0.70855	0.70864	0.00005
46.83	0.70880	0.70867	0.00007
46.79	0.70868	0.70866	0.00006
46.74	0.70857	0.70867	0.00006
46.70	0.70859	0.70875	0.00015
46.66	0.70866	0.70875	0.00015
46.61	0.70855	0.70878	0.00016
46.57	0.70867	0.70883	0.00016

46.53	0.70862	0.70882	0.00016
46.48	0.70873	0.70884	0.00015
46.44	0.70888	0.70884	0.00016
46.40	0.70870	0.70879	0.00018
46.35	0.70870	0.70879	0.00018
46.31	0.70938	0.70879	0.00018
46.27	0.70858	0.70871	0.00012
46.22	0.70899	0.70873	0.00012
46.18	0.70903	0.70867	0.00012
46.14	0.70860	0.70861	0.00010
46.09	0.70887	0.70862	0.00011
46.05	0.70867	0.70860	0.00009
46.01	0.70841	0.70858	0.00009
45.96	0.70867	0.70859	0.00009
45.92	0.70870	0.70860	0.00009
45.88	0.70859	0.70862	0.00011
45.84	0.70877	0.70862	0.00011
45.79	0.70837	0.70861	0.00011
45.75	0.70846	0.70868	0.00013
45.71	0.70874	0.70869	0.00013
45.66	0.70860	0.70870	0.00013
45.62	0.70850	0.70875	0.00015
45.58	0.70847	0.70873	0.00016
45.53	0.70878	0.70873	0.00016
45.49	0.70892	0.70872	0.00016
45.45	0.70856	0.70871	0.00016
45.40	0.70868	0.70868	0.00019
45.36	0.70911	0.70866	0.00019
45.32	0.70851	0.70861	0.00017
45.27	0.70886	0.70861	0.00017
45.23	0.70909	0.70859	0.00016
45.19	0.70838	0.70854	0.00011
45.14	0.70847	0.70857	0.00011
45.10	0.70863	0.70861	0.00012
45.06	0.70886	0.70861	0.00012
45.01	0.70818	0.70862	0.00013
44.97	0.70857	0.70869	0.00010
44.93	0.70860	0.70871	0.00010
44.88	0.70850	0.70870	0.00011
44.84	0.70863	0.70869	0.00012
44.80	0.70853	0.70867	0.00013
44.76	0.70869	0.70867	0.00013
44.71	0.70888	0.70866	0.00013
44.67	0.70866	0.70864	0.00013
44.63	0.70892	0.70864	0.00013
44.58	0.70893	0.70860	0.00011
44.54	0.70878	0.70858	0.00008
44.50	0.70845	0.70856	0.00007
44.45	0.70837	0.70856	0.00007
44.41	0.70853	0.70857	0.00007

44.37	0.70844	0.70859	0.00008
44.32	0.70862	0.70861	0.00007
44.28	0.70869	0.70865	0.00010
44.24	0.70868	0.70866	0.00011
44.19	0.70856	0.70866	0.00011
44.15	0.70865	0.70865	0.00011
44.11	0.70867	0.70869	0.00013
44.06	0.70844	0.70870	0.00013
44.02	0.70842	0.70869	0.00014
43.98	0.70874	0.70872	0.00012
43.93	0.70869	0.70874	0.00013
43.89	0.70900	0.70869	0.00017
43.85	0.70882	0.70867	0.00016
43.80	0.70859	0.70863	0.00016
43.76	0.70854	0.70863	0.00016
43.72	0.70902	0.70864	0.00016
43.68	0.70873	0.70863	0.00015
43.63	0.70839	0.70860	0.00016
43.59	0.70866	0.70860	0.00016
43.55	0.70898	0.70860	0.00016
43.50	0.70818	0.70855	0.00013
43.46	0.70877	0.70862	0.00011
43.42	0.70848	0.70856	0.00014
43.37	0.70855	0.70858	0.00014
43.33	0.70867	0.70856	0.00015
43.29	0.70890	0.70855	0.00015
43.24	0.70841	0.70850	0.00013
43.20	0.70837	0.70853	0.00014
43.16	0.70869	0.70856	0.00013
43.11	0.70849	0.70856	0.00013
43.07	0.70882	0.70858	0.00013
43.03	0.70818	0.70856	0.00012
42.98	0.70876	0.70861	0.00009
42.94	0.70829	0.70865	0.00014
42.90	0.70859	0.70866	0.00013
42.85	0.70844	0.70870	0.00014
42.81	0.70870	0.70873	0.00013
42.77	0.70870	0.70875	0.00013
42.72	0.70861	0.70874	0.00014
42.68	0.70868	0.70874	0.00014
42.64	0.70861	0.70870	0.00016
42.60	0.70871	0.70877	0.00019
42.55	0.70916	0.70877	0.00019
42.51	0.70839	0.70870	0.00018
42.47	0.70895	0.70874	0.00017
42.42	0.70876	0.70874	0.00017
42.38	0.70888	0.70871	0.00018
42.34	0.70863	0.70868	0.00018
42.29	0.70859	0.70866	0.00018
42.25	0.70835	0.70867	0.00018

42.21	0.70928	0.70866	0.00019
42.16	0.70873	0.70860	0.00013
42.12	0.70847	0.70856	0.00014
42.08	0.70874	0.70858	0.00014
42.03	0.70901	0.70860	0.00015
41.99	0.70844	0.70855	0.00012
41.95	0.70853	0.70862	0.00016
41.90	0.70850	0.70864	0.00016
41.86	0.70862	0.70863	0.00017
41.82	0.70823	0.70864	0.00017
41.77	0.70868	0.70872	0.00016
41.73	0.70837	0.70868	0.00018
41.69	0.70868	0.70871	0.00016
41.64	0.70891	0.70867	0.00018
41.60	0.70858	0.70868	0.00018
41.56	0.70915	0.70867	0.00018
41.52	0.70868	0.70862	0.00015
41.47	0.70838	0.70859	0.00016
41.43	0.70878	0.70858	0.00016
41.39	0.70896	0.70858	0.00016
41.34	0.70828	0.70855	0.00014
41.30	0.70868	0.70856	0.00013
41.26	0.70834	0.70854	0.00013
41.21	0.70893	0.70854	0.00013
41.17	0.70855	0.70850	0.00009
41.13	0.70866	0.70849	0.00009
41.08	0.70834	0.70845	0.00010
41.04	0.70832	0.70845	0.00010
41.00	0.70875	0.70852	0.00014
40.95	0.70862	0.70850	0.00013
40.91	0.70845	0.70846	0.00013
40.87	0.70842	0.70852	0.00018
40.82	0.70836	0.70852	0.00018
40.78	0.70850	0.70855	0.00017
40.74	0.70851	0.70855	0.00017
40.69	0.70821	0.70854	0.00018
40.65	0.70838	0.70856	0.00016
40.61	0.70898	0.70859	0.00016
40.56	0.70852	0.70854	0.00013
40.52	0.70826	0.70855	0.00013
40.48	0.70903	0.70857	0.00012
40.44	0.70845	0.70855	0.00008
40.39	0.70867	0.70857	0.00009
40.35	0.70849	0.70854	0.00009
40.31	0.70838	0.70857	0.00011
40.26	0.70848	0.70857	0.00011
40.22	0.70862	0.70860	0.00012
40.18	0.70848	0.70860	0.00012
40.13	0.70868	0.70863	0.00012
40.09	0.70843	0.70860	0.00013

40.05	0.70878	0.70860	0.00013
40.00	0.70872	0.70858	0.00012
39.96	0.70837	0.70855	0.00012
39.92	0.70880	0.70857	0.00011
39.87	0.70831	0.70857	0.00012
39.83	0.70882	0.70862	0.00011
39.79	0.70864	0.70859	0.00010
39.74	0.70872	0.70860	0.00010
39.70	0.70840	0.70861	0.00011
39.66	0.70839	0.70861	0.00010
39.61	0.70860	0.70863	0.00010
39.57	0.70842	0.70868	0.00014
39.53	0.70860	0.70865	0.00017
39.48	0.70882	0.70864	0.00017
39.44	0.70876	0.70863	0.00017
39.40	0.70856	0.70859	0.00018
39.36	0.70874	0.70859	0.00018
39.31	0.70881	0.70858	0.00017
39.27	0.70844	0.70857	0.00017
39.23	0.70850	0.70857	0.00017
39.18	0.70912	0.70859	0.00017
39.14	0.70811	0.70858	0.00015
39.10	0.70859	0.70862	0.00011
39.05	0.70868	0.70866	0.00013
39.01	0.70832	0.70868	0.00014
38.97	0.70864	0.70871	0.00011
38.92	0.70862	0.70873	0.00011
38.88	0.70873	0.70870	0.00013
38.84	0.70842	0.70870	0.00013
38.79	0.70868	0.70873	0.00012
38.75	0.70899	0.70874	0.00012
38.71	0.70858	0.70869	0.00012
38.66	0.70896	0.70870	0.00011
38.62	0.70885	0.70868	0.00010
38.58	0.70862	0.70864	0.00011
38.53	0.70883	0.70864	0.00011
38.49	0.70837	0.70862	0.00010
38.45	0.70875	0.70863	0.00009
38.40	0.70872	0.70858	0.00011
38.36	0.70874	0.70856	0.00011
38.32	0.70844	0.70854	0.00010
38.28	0.70875	0.70856	0.00010
38.23	0.70878	0.70855	0.00009
38.19	0.70838	0.70853	0.00008
38.15	0.70863	0.70855	0.00007
38.10	0.70861	0.70859	0.00011
38.06	0.70851	0.70860	0.00011
38.02	0.70825	0.70862	0.00011
37.97	0.70851	0.70864	0.00008
37.93	0.70857	0.70864	0.00008

37.89	0.70861	0.70862	0.00010
37.84	0.70862	0.70860	0.00010
37.80	0.70864	0.70858	0.00012
37.76	0.70859	0.70858	0.00012
37.71	0.70894	0.70859	0.00012
37.67	0.70874	0.70856	0.00010
37.63	0.70869	0.70858	0.00011
37.58	0.70849	0.70855	0.00011
37.54	0.70852	0.70854	0.00012
37.50	0.70837	0.70854	0.00012
37.45	0.70843	0.70857	0.00011
37.41	0.70832	0.70854	0.00014
37.37	0.70874	0.70858	0.00013
37.32	0.70865	0.70857	0.00013
37.28	0.70867	0.70857	0.00013
37.24	0.70887	0.70855	0.00013
37.20	0.70842	0.70852	0.00011
37.15	0.70840	0.70853	0.00011
37.11	0.70850	0.70860	0.00014
37.07	0.70869	0.70858	0.00015
37.02	0.70815	0.70857	0.00015
36.98	0.70869	0.70864	0.00012
36.94	0.70866	0.70859	0.00015
36.89	0.70865	0.70860	0.00016
36.85	0.70846	0.70861	0.00016
36.81	0.70858	0.70862	0.00016
36.76	0.70856	0.70863	0.00016
36.72	0.70902	0.70865	0.00016
36.68	0.70833	0.70858	0.00015
36.63	0.70862	0.70861	0.00014
36.59	0.70888	0.70864	0.00015
36.55	0.70817	0.70859	0.00014
36.50	0.70879	0.70865	0.00011
36.46	0.70874	0.70865	0.00011
36.42	0.70848	0.70864	0.00011
36.37	0.70875	0.70866	0.00010
36.33	0.70870	0.70866	0.00010
36.29	0.70835	0.70867	0.00010
36.24	0.70868	0.70873	0.00010
36.20	0.70886	0.70874	0.00010
36.16	0.70842	0.70872	0.00010
36.12	0.70872	0.70877	0.00008
36.07	0.70883	0.70876	0.00009
36.03	0.70862	0.70871	0.00011
35.99	0.70870	0.70869	0.00013
35.94	0.70869	0.70866	0.00014
35.90	0.70878	0.70861	0.00017
35.86	0.70904	0.70859	0.00016
35.81	0.70874	0.70853	0.00013
35.77	0.70863	0.70850	0.00012

35.73	0.70890	0.70852	0.00014
35.68	0.70862	0.70843	0.00015
35.64	0.70836	0.70842	0.00015
35.60	0.70842	0.70841	0.00015
35.55	0.70838	0.70845	0.00017
35.51	0.70823	0.70844	0.00017
35.47	0.70855	0.70850	0.00018
35.42	0.70845	0.70848	0.00018
35.38	0.70843	0.70851	0.00019
35.34	0.70890	0.70852	0.00019
35.29	0.70798	0.70848	0.00017
35.25	0.70847	0.70854	0.00013
35.21	0.70833	0.70857	0.00013
35.16	0.70880	0.70860	0.00012
35.12	0.70826	0.70856	0.00012
35.08	0.70881	0.70861	0.00011
35.04	0.70839	0.70862	0.00012
34.99	0.70877	0.70862	0.00012
34.95	0.70852	0.70859	0.00012
34.91	0.70846	0.70862	0.00012
34.86	0.70862	0.70861	0.00012
34.82	0.70878	0.70857	0.00015
34.78	0.70863	0.70859	0.00017
34.73	0.70834	0.70860	0.00017
34.69	0.70883	0.70864	0.00016
34.65	0.70886	0.70862	0.00016
34.60	0.70839	0.70863	0.00017
34.56	0.70852	0.70866	0.00016
34.52	0.70875	0.70866	0.00016
34.47	0.70838	0.70864	0.00016
34.43	0.70819	0.70865	0.00016
34.39	0.70901	0.70873	0.00014
34.34	0.70873	0.70866	0.00014
34.30	0.70875	0.70866	0.00014
34.26	0.70860	0.70869	0.00015
34.21	0.70899	0.70871	0.00015
34.17	0.70864	0.70868	0.00014
34.13	0.70851	0.70870	0.00014
34.08	0.70859	0.70870	0.00014
34.04	0.70844	0.70870	0.00014
34.00	0.70902	0.70874	0.00012
33.96	0.70835	0.70869	0.00011
33.91	0.70876	0.70875	0.00009
33.87	0.70895	0.70873	0.00009
33.83	0.70884	0.70869	0.00008
33.78	0.70872	0.70870	0.00009
33.74	0.70879	0.70867	0.00010
33.70	0.70852	0.70862	0.00013
33.65	0.70865	0.70863	0.00013
33.61	0.70882	0.70863	0.00013

33.57	0.70854	0.70860	0.00012
33.52	0.70887	0.70864	0.00014
33.48	0.70862	0.70861	0.00013
33.44	0.70857	0.70863	0.00013
33.39	0.70892	0.70863	0.00013
33.35	0.70845	0.70862	0.00012
33.31	0.70824	0.70860	0.00014
33.26	0.70864	0.70864	0.00012
33.22	0.70864	0.70866	0.00013
33.18	0.70850	0.70868	0.00013
33.13	0.70890	0.70871	0.00013
33.09	0.70861	0.70870	0.00012
33.05	0.70879	0.70871	0.00012
33.00	0.70863	0.70872	0.00012
32.96	0.70883	0.70871	0.00013
32.92	0.70824	0.70869	0.00013
32.88	0.70859	0.70868	0.00013
32.83	0.70888	0.70871	0.00014
32.79	0.70884	0.70870	0.00013
32.75	0.70878	0.70868	0.00013
32.70	0.70881	0.70862	0.00017
32.66	0.70874	0.70861	0.00017
32.62	0.70888	0.70859	0.00017
32.57	0.70854	0.70858	0.00016
32.53	0.70856	0.70859	0.00016
32.49	0.70821	0.70861	0.00017
32.44	0.70889	0.70865	0.00014
32.40	0.70870	0.70860	0.00014
32.36	0.70874	0.70860	0.00014
32.31	0.70810	0.70863	0.00016
32.27	0.70878	0.70867	0.00012
32.23	0.70850	0.70869	0.00012
32.18	0.70883	0.70868	0.00013
32.14	0.70863	0.70868	0.00013
32.10	0.70872	0.70873	0.00015
32.05	0.70864	0.70875	0.00015
32.01	0.70840	0.70874	0.00016
31.97	0.70869	0.70876	0.00015
31.92	0.70904	0.70874	0.00016
31.88	0.70852	0.70870	0.00014
31.84	0.70889	0.70872	0.00014
31.80	0.70846	0.70869	0.00013
31.75	0.70884	0.70872	0.00013
31.71	0.70906	0.70871	0.00014
31.67	0.70897	0.70865	0.00011
31.62	0.70850	0.70860	0.00007
31.58	0.70859	0.70862	0.00007
31.54	0.70848	0.70863	0.00008
31.49	0.70867	0.70868	0.00006
31.45	0.70878	0.70868	0.00009

31.41	0.70858	0.70858	0.00006
30.97	0.70907	0.70878	0.00017
30.93	0.70833	0.70876	0.00015
30.89	0.70886	0.70877	0.00015
30.84	0.70862	0.70872	0.00016
30.80	0.70894	0.70871	0.00016
30.76	0.70911	0.70871	0.00016
30.72	0.70842	0.70865	0.00013
30.67	0.70888	0.70865	0.00014
30.63	0.70867	0.70859	0.00015
30.59	0.70889	0.70859	0.00014
30.54	0.70888	0.70857	0.00013
30.50	0.70840	0.70856	0.00012
30.46	0.70841	0.70857	0.00012
30.41	0.70855	0.70857	0.00012
30.37	0.70887	0.70859	0.00012
30.33	0.70854	0.70859	0.00011
30.28	0.70839	0.70864	0.00015
30.24	0.70827	0.70867	0.00014
30.20	0.70867	0.70872	0.00010
30.15	0.70868	0.70875	0.00011
30.11	0.70879	0.70876	0.00011
30.07	0.70848	0.70871	0.00014
30.02	0.70849	0.70876	0.00013
29.98	0.70876	0.70879	0.00012
29.94	0.70878	0.70878	0.00013
29.89	0.70906	0.70881	0.00014
29.85	0.70877	0.70878	0.00013
29.81	0.70873	0.70877	0.00013
29.76	0.70893	0.70877	0.00013
29.72	0.70881	0.70873	0.00014
29.68	0.70835	0.70866	0.00017
29.64	0.70898	0.70869	0.00016
29.59	0.70878	0.70869	0.00015
29.55	0.70860	0.70863	0.00018
29.51	0.70910	0.70865	0.00019
29.46	0.70878	0.70864	0.00018
29.42	0.70868	0.70865	0.00018
29.38	0.70867	0.70863	0.00018
29.33	0.70853	0.70860	0.00019
29.29	0.70816	0.70861	0.00019
29.25	0.70865	0.70864	0.00016
29.20	0.70890	0.70860	0.00018
29.16	0.70819	0.70860	0.00018
29.12	0.70885	0.70866	0.00016
29.07	0.70901	0.70862	0.00015
29.03	0.70881	0.70861	0.00014
28.99	0.70855	0.70861	0.00014
28.94	0.70833	0.70864	0.00015
28.90	0.70861	0.70869	0.00014

28.86	0.70850	0.70872	0.00014
28.81	0.70826	0.70873	0.00013
28.77	0.70886	0.70874	0.00012
28.73	0.70878	0.70873	0.00012
28.68	0.70851	0.70869	0.00013
28.64	0.70894	0.70871	0.00013
28.60	0.70874	0.70867	0.00012
28.56	0.70891	0.70867	0.00012
28.51	0.70874	0.70860	0.00014
28.47	0.70892	0.70862	0.00016
28.43	0.70867	0.70855	0.00016
28.38	0.70837	0.70857	0.00017
28.34	0.70870	0.70863	0.00018
28.30	0.70838	0.70863	0.00018
28.25	0.70878	0.70865	0.00017
28.21	0.70850	0.70863	0.00017
28.17	0.70872	0.70864	0.00017
28.12	0.70820	0.70862	0.00017
28.08	0.70895	0.70865	0.00014
28.04	0.70823	0.70863	0.00013
27.99	0.70886	0.70864	0.00011
27.95	0.70893	0.70863	0.00010
27.91	0.70875	0.70858	0.00008
27.86	0.70859	0.70857	0.00007
27.82	0.70855	0.70862	0.00013
27.78	0.70861	0.70862	0.00013
27.73	0.70850	0.70862	0.00013
27.69	0.70854	0.70867	0.00014
27.65	0.70875	0.70867	0.00015
27.60	0.70836	0.70868	0.00015
27.56	0.70868	0.70874	0.00014
27.52	0.70851	0.70875	0.00014
27.48	0.70859	0.70875	0.00014
27.43	0.70910	0.70874	0.00015
27.39	0.70856	0.70871	0.00013
27.35	0.70864	0.70870	0.00014
27.30	0.70900	0.70870	0.00014
27.26	0.70850	0.70865	0.00012
27.22	0.70881	0.70866	0.00012
27.17	0.70902	0.70868	0.00013
27.13	0.70873	0.70864	0.00011
27.09	0.70857	0.70869	0.00016
27.04	0.70841	0.70870	0.00016
27.00	0.70887	0.70874	0.00015
26.96	0.70849	0.70872	0.00014
26.91	0.70863	0.70874	0.00014
26.87	0.70851	0.70873	0.00014
26.83	0.70857	0.70873	0.00014
26.78	0.70896	0.70880	0.00017
26.74	0.70868	0.70876	0.00017

26.70	0.70923	0.70872	0.00020
26.65	0.70867	0.70867	0.00017
26.61	0.70882	0.70866	0.00017
26.57	0.70862	0.70866	0.00017
26.52	0.70865	0.70867	0.00017
26.48	0.70858	0.70863	0.00019
26.44	0.70847	0.70865	0.00019
26.40	0.70928	0.70866	0.00019
26.35	0.70863	0.70856	0.00014
26.31	0.70826	0.70857	0.00015
26.27	0.70875	0.70861	0.00013
26.22	0.70854	0.70862	0.00013
26.18	0.70878	0.70864	0.00013
26.14	0.70876	0.70861	0.00013
26.09	0.70825	0.70859	0.00013
26.05	0.70882	0.70862	0.00010
26.01	0.70856	0.70859	0.00009
25.96	0.70829	0.70864	0.00012
25.92	0.70871	0.70867	0.00010
25.88	0.70865	0.70865	0.00010
25.83	0.70883	0.70864	0.00010
25.79	0.70871	0.70866	0.00012
25.75	0.70850	0.70870	0.00016
25.70	0.70861	0.70875	0.00016
25.66	0.70856	0.70880	0.00017
25.62	0.70853	0.70880	0.00017
25.57	0.70900	0.70881	0.00016
25.53	0.70856	0.70877	0.00016
25.49	0.70858	0.70878	0.00015
25.44	0.70851	0.70878	0.00016
25.40	0.70902	0.70877	0.00016
25.36	0.70916	0.70872	0.00016
25.32	0.70899	0.70866	0.00013
25.27	0.70906	0.70866	0.00012
25.23	0.70855	0.70861	0.00008
25.19	0.70865	0.70860	0.00009
25.14	0.70863	0.70859	0.00009
25.10	0.70868	0.70856	0.00010
25.06	0.70857	0.70856	0.00010
25.01	0.70842	0.70860	0.00012
24.97	0.70851	0.70863	0.00012
24.93	0.70858	0.70868	0.00014
24.88	0.70891	0.70870	0.00014
24.84	0.70860	0.70872	0.00016
24.80	0.70847	0.70874	0.00016
24.75	0.70858	0.70874	0.00016
24.71	0.70831	0.70873	0.00016
24.67	0.70867	0.70875	0.00015
24.62	0.70893	0.70869	0.00020
24.58	0.70871	0.70869	0.00020

24.54	0.70903	0.70869	0.00020
24.49	0.70879	0.70864	0.00019
24.45	0.70913	0.70860	0.00019
24.41	0.70877	0.70855	0.00015
24.36	0.70843	0.70852	0.00014
24.32	0.70850	0.70853	0.00014
24.28	0.70850	0.70855	0.00015
24.24	0.70807	0.70860	0.00017
24.19	0.70892	0.70862	0.00015
24.15	0.70873	0.70856	0.00014
24.11	0.70855	0.70854	0.00014
24.06	0.70844	0.70855	0.00014
24.02	0.70861	0.70857	0.00014
23.98	0.70850	0.70855	0.00014
23.93	0.70844	0.70859	0.00015
23.89	0.70876	0.70861	0.00015
23.85	0.70899	0.70861	0.00015
23.80	0.70822	0.70858	0.00013
23.76	0.70835	0.70860	0.00010
23.72	0.70855	0.70864	0.00009
23.67	0.70864	0.70864	0.00009
23.63	0.70866	0.70860	0.00012
23.59	0.70839	0.70860	0.00012
23.54	0.70885	0.70859	0.00013
23.50	0.70872	0.70862	0.00017
23.46	0.70874	0.70865	0.00018
23.41	0.70871	0.70863	0.00018
23.37	0.70845	0.70860	0.00019
23.33	0.70868	0.70863	0.00019
23.28	0.70861	0.70859	0.00020
23.24	0.70819	0.70866	0.00025
23.20	0.70864	0.70870	0.00023
23.16	0.70834	0.70866	0.00025
23.11	0.70917	0.70871	0.00024
23.07	0.70900	0.70865	0.00022
23.03	0.70856	0.70862	0.00020
22.98	0.70840	0.70864	0.00020
22.94	0.70873	0.70867	0.00020
22.90	0.70822	0.70866	0.00020
22.85	0.70935	0.70867	0.00018
22.81	0.70855	0.70860	0.00011
22.77	0.70829	0.70863	0.00012
22.72	0.70884	0.70868	0.00010
22.68	0.70860	0.70869	0.00012
22.64	0.70870	0.70869	0.00012
22.59	0.70871	0.70869	0.00012
22.55	0.70868	0.70868	0.00012
22.51	0.70868	0.70867	0.00012
22.46	0.70833	0.70866	0.00012
22.42	0.70860	0.70865	0.00014

22.38	0.70891	0.70863	0.00014
22.33	0.70871	0.70860	0.00013
22.29	0.70901	0.70863	0.00015
22.25	0.70852	0.70863	0.00014
22.20	0.70872	0.70865	0.00014
22.16	0.70861	0.70868	0.00016
22.12	0.70860	0.70870	0.00016
22.08	0.70857	0.70873	0.00017
22.03	0.70821	0.70875	0.00016
21.99	0.70844	0.70880	0.00011
21.95	0.70865	0.70882	0.00009
21.90	0.70898	0.70878	0.00015
21.86	0.70896	0.70879	0.00015
21.82	0.70877	0.70875	0.00015
21.77	0.70903	0.70873	0.00015
21.73	0.70874	0.70868	0.00014
21.69	0.70890	0.70868	0.00014
21.64	0.70880	0.70864	0.00014
21.60	0.70876	0.70865	0.00015
21.56	0.70865	0.70864	0.00015
21.51	0.70821	0.70865	0.00015
21.47	0.70903	0.70869	0.00012
21.43	0.70858	0.70867	0.00010
21.38	0.70860	0.70864	0.00012
21.34	0.70853	0.70867	0.00013
21.30	0.70874	0.70872	0.00014
21.25	0.70845	0.70871	0.00014
21.21	0.70897	0.70871	0.00014
21.17	0.70863	0.70872	0.00015
21.12	0.70881	0.70872	0.00015
21.08	0.70860	0.70873	0.00016
21.04	0.70877	0.70874	0.00015
21.00	0.70832	0.70878	0.00019
20.95	0.70885	0.70886	0.00016
20.91	0.70904	0.70884	0.00016
20.87	0.70867	0.70887	0.00018
20.82	0.70846	0.70888	0.00018
20.78	0.70904	0.70893	0.00015
20.74	0.70860	0.70888	0.00017
20.69	0.70896	0.70889	0.00016
20.65	0.70866	0.70889	0.00016
20.61	0.70925	0.70885	0.00020
20.56	0.70906	0.70886	0.00021
20.52	0.70870	0.70885	0.00021
20.48	0.70926	0.70887	0.00020
20.43	0.70879	0.70888	0.00022
20.39	0.70895	0.70885	0.00023
20.35	0.70852	0.70886	0.00023
20.30	0.70874	0.70885	0.00024
20.26	0.70896	0.70888	0.00024

20.22	0.70824	0.70886	0.00024
20.17	0.70935	0.70895	0.00021
20.13	0.70895	0.70885	0.00022
20.09	0.70889	0.70883	0.00022
20.04	0.70940	0.70883	0.00022
20.00	0.70849	0.70876	0.00018
19.96	0.70905	0.70877	0.00017
19.92	0.70843	0.70874	0.00016
19.87	0.70903	0.70880	0.00016
19.83	0.70875	0.70873	0.00017
19.79	0.70916	0.70871	0.00018
19.74	0.70836	0.70870	0.00017
19.70	0.70875	0.70871	0.00016
19.66	0.70892	0.70867	0.00017
19.61	0.70868	0.70865	0.00016
19.57	0.70857	0.70860	0.00018
19.53	0.70873	0.70860	0.00018
19.48	0.70909	0.70859	0.00018
19.44	0.70833	0.70857	0.00016
19.40	0.70855	0.70860	0.00015
19.35	0.70905	0.70861	0.00015
19.31	0.70843	0.70860	0.00015
19.27	0.70842	0.70862	0.00014
19.22	0.70866	0.70862	0.00015
19.18	0.70823	0.70862	0.00015
19.14	0.70850	0.70868	0.00012
19.09	0.70862	0.70871	0.00012
19.05	0.70893	0.70864	0.00020
19.01	0.70864	0.70864	0.00020
18.96	0.70859	0.70865	0.00020
18.92	0.70901	0.70863	0.00020
18.88	0.70864	0.70859	0.00019
18.84	0.70838	0.70862	0.00020
18.79	0.70866	0.70867	0.00019
18.75	0.70886	0.70868	0.00019
18.71	0.70881	0.70863	0.00020
18.66	0.70792	0.70861	0.00020
18.62	0.70891	0.70870	0.00013
18.58	0.70869	0.70868	0.00012
18.53	0.70839	0.70868	0.00012
18.49	0.70866	0.70872	0.00010
18.45	0.70890	0.70878	0.00015
18.40	0.70886	0.70871	0.00019
18.36	0.70880	0.70870	0.00019
18.32	0.70836	0.70869	0.00019
18.27	0.70862	0.70874	0.00017
18.23	0.70883	0.70880	0.00020
18.19	0.70866	0.70882	0.00020
18.14	0.70871	0.70879	0.00021
18.10	0.70877	0.70879	0.00021

18.06	0.70931	0.70879	0.00021
18.01	0.70819	0.70875	0.00018
17.97	0.70874	0.70881	0.00013
17.93	0.70867	0.70881	0.00013
17.88	0.70887	0.70882	0.00013
17.84	0.70921	0.70882	0.00013
17.80	0.70902	0.70878	0.00010
17.76	0.70844	0.70877	0.00009
17.71	0.70872	0.70875	0.00012
17.67	0.70873	0.70876	0.00013
17.63	0.70893	0.70876	0.00013
17.58	0.70875	0.70871	0.00014
17.54	0.70880	0.70871	0.00014
17.50	0.70871	0.70870	0.00014
17.45	0.70885	0.70868	0.00014
17.41	0.70887	0.70870	0.00015
17.37	0.70887	0.70869	0.00015
17.32	0.70823	0.70870	0.00015
17.28	0.70884	0.70881	0.00017
17.24	0.70875	0.70881	0.00017
17.19	0.70840	0.70889	0.00021
17.15	0.70878	0.70896	0.00019
17.11	0.70872	0.70893	0.00020
17.06	0.70854	0.70895	0.00020
17.02	0.70897	0.70902	0.00018
16.98	0.70884	0.70898	0.00020
16.93	0.70889	0.70897	0.00020
16.89	0.70938	0.70897	0.00020
16.85	0.70884	0.70894	0.00018
16.80	0.70951	0.70893	0.00019
16.76	0.70910	0.70886	0.00014
16.72	0.70852	0.70878	0.00016
16.68	0.70891	0.70885	0.00017
16.63	0.70922	0.70883	0.00017
16.59	0.70862	0.70882	0.00016
16.55	0.70875	0.70887	0.00016
16.50	0.70890	0.70888	0.00016
16.46	0.70907	0.70891	0.00017
16.42	0.70871	0.70888	0.00017
16.37	0.70877	0.70886	0.00018
16.33	0.70836	0.70885	0.00018
16.29	0.70919	0.70890	0.00014
16.24	0.70872	0.70882	0.00016
16.20	0.70907	0.70886	0.00017
16.16	0.70915	0.70885	0.00016
16.11	0.70887	0.70880	0.00015
16.07	0.70914	0.70879	0.00015
16.03	0.70881	0.70880	0.00015
15.98	0.70853	0.70881	0.00015
15.94	0.70868	0.70886	0.00015

15.90	0.70886	0.70883	0.00017
15.85	0.70840	0.70881	0.00018
15.81	0.70910	0.70888	0.00016
15.77	0.70894	0.70883	0.00016
15.72	0.70867	0.70883	0.00016
15.68	0.70880	0.70883	0.00016
15.64	0.70916	0.70879	0.00017
15.60	0.70895	0.70875	0.00015
15.55	0.70906	0.70872	0.00014
15.51	0.70839	0.70874	0.00017
15.47	0.70862	0.70879	0.00015
15.42	0.70909	0.70880	0.00015
15.38	0.70866	0.70876	0.00013
15.34	0.70890	0.70875	0.00014
15.29	0.70865	0.70875	0.00014
15.25	0.70848	0.70879	0.00014
15.21	0.70869	0.70884	0.00013
15.16	0.70866	0.70885	0.00013
15.12	0.70927	0.70883	0.00014
15.08	0.70884	0.70876	0.00012
15.03	0.70880	0.70876	0.00012
14.99	0.70868	0.70873	0.00014
14.95	0.70856	0.70873	0.00014
14.90	0.70887	0.70876	0.00014
14.86	0.70901	0.70876	0.00014
14.82	0.70901	0.70870	0.00014
14.77	0.70873	0.70870	0.00014
14.73	0.70853	0.70869	0.00014
14.69	0.70850	0.70874	0.00015
14.64	0.70893	0.70877	0.00014
14.60	0.70841	0.70878	0.00015
14.56	0.70876	0.70880	0.00014
14.52	0.70883	0.70883	0.00014
14.47	0.70884	0.70881	0.00015
14.43	0.70841	0.70887	0.00019
14.39	0.70904	0.70890	0.00017
14.34	0.70865	0.70885	0.00018
14.30	0.70898	0.70886	0.00018
14.26	0.70880	0.70883	0.00018
14.21	0.70911	0.70886	0.00018
14.17	0.70857	0.70884	0.00017
14.13	0.70903	0.70880	0.00022
14.08	0.70869	0.70877	0.00022
14.04	0.70943	0.70878	0.00022
14.00	0.70875	0.70872	0.00016
13.95	0.70853	0.70877	0.00019
13.91	0.70868	0.70880	0.00019
13.87	0.70873	0.70887	0.00022
13.82	0.70908	0.70889	0.00021
13.78	0.70893	0.70883	0.00022

13.74	0.70811	0.70879	0.00023
13.69	0.70874	0.70882	0.00020
13.65	0.70885	0.70885	0.00020
13.61	0.70879	0.70882	0.00022
13.56	0.70923	0.70885	0.00023
13.52	0.70889	0.70879	0.00021
13.48	0.70937	0.70876	0.00022
13.44	0.70888	0.70869	0.00017
13.39	0.70846	0.70868	0.00016
13.35	0.70857	0.70871	0.00016
13.31	0.70839	0.70872	0.00016
13.26	0.70911	0.70877	0.00014
13.22	0.70846	0.70871	0.00012
13.18	0.70912	0.70880	0.00015
13.13	0.70866	0.70880	0.00015
13.09	0.70854	0.70880	0.00015
13.05	0.70871	0.70882	0.00014
13.00	0.70874	0.70882	0.00014
12.96	0.70882	0.70885	0.00014
12.92	0.70863	0.70884	0.00015
12.87	0.70888	0.70886	0.00014
12.83	0.70859	0.70884	0.00014
12.79	0.70929	0.70887	0.00013
12.74	0.70915	0.70884	0.00010
12.70	0.70871	0.70883	0.00008
12.66	0.70871	0.70883	0.00008
12.61	0.70874	0.70887	0.00009
12.57	0.70900	0.70893	0.00013
12.53	0.70873	0.70889	0.00014
12.48	0.70878	0.70892	0.00014
12.44	0.70872	0.70892	0.00014
12.40	0.70892	0.70895	0.00013
12.36	0.70894	0.70898	0.00014
12.31	0.70905	0.70899	0.00014
12.27	0.70873	0.70898	0.00014
12.23	0.70906	0.70901	0.00014
12.18	0.70937	0.70900	0.00015
12.14	0.70863	0.70895	0.00012
12.10	0.70904	0.70900	0.00009
12.05	0.70878	0.70900	0.00010
12.01	0.70897	0.70905	0.00008
11.97	0.70922	0.70908	0.00009
11.92	0.70908	0.70901	0.00006
11.88	0.70894	0.70894	0.00008
11.10	0.70899	0.70874	0.00011
11.06	0.70880	0.70874	0.00010
11.02	0.70871	0.70868	0.00014
10.97	0.70857	0.70866	0.00014
10.93	0.70861	0.70871	0.00016
10.89	0.70865	0.70873	0.00016

10.84	0.70849	0.70873	0.00016
10.80	0.70895	0.70880	0.00017
10.76	0.70888	0.70883	0.00019
10.71	0.70880	0.70883	0.00019
10.67	0.70891	0.70880	0.00021
10.63	0.70826	0.70881	0.00021
10.58	0.70851	0.70881	0.00020
10.54	0.70904	0.70885	0.00019
10.50	0.70883	0.70883	0.00018
10.45	0.70864	0.70878	0.00021
10.41	0.70918	0.70881	0.00021
10.37	0.70929	0.70880	0.00020
10.32	0.70890	0.70873	0.00017
10.28	0.70850	0.70872	0.00017
10.24	0.70893	0.70874	0.00016
10.20	0.70832	0.70876	0.00018
10.15	0.70889	0.70884	0.00015
10.11	0.70884	0.70881	0.00016
10.07	0.70832	0.70882	0.00016
10.02	0.70893	0.70881	0.00017
9.98	0.70906	0.70880	0.00017
9.94	0.70863	0.70880	0.00017
9.89	0.70878	0.70881	0.00017
9.85	0.70873	0.70882	0.00017
9.81	0.70915	0.70879	0.00019
9.76	0.70907	0.70875	0.00017
9.72	0.70859	0.70873	0.00016
9.68	0.70892	0.70874	0.00016
9.63	0.70827	0.70870	0.00016
9.59	0.70874	0.70876	0.00013
9.55	0.70911	0.70878	0.00014
9.50	0.70876	0.70875	0.00011
9.46	0.70885	0.70874	0.00011
9.42	0.70838	0.70872	0.00012
9.37	0.70882	0.70876	0.00009
9.33	0.70882	0.70875	0.00009
9.29	0.70875	0.70874	0.00009
9.24	0.70847	0.70872	0.00010
9.20	0.70888	0.70875	0.00008
9.16	0.70895	0.70874	0.00007
9.12	0.70878	0.70872	0.00006
9.07	0.70872	0.70873	0.00007
9.03	0.70860	0.70875	0.00007
8.99	0.70879	0.70880	0.00010
8.94	0.70872	0.70881	0.00010
8.90	0.70880	0.70885	0.00011
8.86	0.70853	0.70885	0.00012
8.81	0.70871	0.70886	0.00010
8.77	0.70880	0.70886	0.00010
8.73	0.70875	0.70888	0.00011

8.68	0.70891	0.70890	0.00010
8.64	0.70885	0.70891	0.00010
8.60	0.70913	0.70892	0.00011
8.55	0.70893	0.70886	0.00012
8.51	0.70912	0.70888	0.00014
8.47	0.70874	0.70883	0.00014
8.42	0.70864	0.70882	0.00014
8.38	0.70876	0.70884	0.00014
8.34	0.70902	0.70886	0.00014
8.29	0.70887	0.70885	0.00013
8.25	0.70902	0.70885	0.00013
8.21	0.70903	0.70885	0.00014
8.16	0.70850	0.70884	0.00013
8.12	0.70915	0.70882	0.00015
8.08	0.70857	0.70878	0.00013
8.04	0.70866	0.70878	0.00013
7.99	0.70884	0.70880	0.00013
7.95	0.70898	0.70877	0.00013
7.91	0.70886	0.70876	0.00012
7.86	0.70885	0.70876	0.00013
7.82	0.70906	0.70871	0.00015
7.78	0.70888	0.70869	0.00013
7.73	0.70837	0.70867	0.00013
7.69	0.70870	0.70872	0.00012
7.65	0.70859	0.70875	0.00013
7.60	0.70884	0.70877	0.00012
7.56	0.70860	0.70879	0.00013
7.52	0.70887	0.70886	0.00016
7.47	0.70887	0.70887	0.00016
7.43	0.70834	0.70886	0.00017
7.39	0.70886	0.70889	0.00014
7.34	0.70863	0.70887	0.00014
7.30	0.70894	0.70888	0.00014
7.26	0.70899	0.70889	0.00014
7.21	0.70877	0.70889	0.00014
7.17	0.70903	0.70889	0.00014
7.13	0.70933	0.70887	0.00013
7.08	0.70896	0.70881	0.00009
7.04	0.70877	0.70880	0.00008
7.00	0.70857	0.70885	0.00011
6.96	0.70871	0.70888	0.00009
6.91	0.70875	0.70891	0.00009
6.87	0.70898	0.70888	0.00014
6.83	0.70901	0.70888	0.00014
6.78	0.70880	0.70887	0.00014
6.74	0.70879	0.70890	0.00014
6.70	0.70876	0.70889	0.00015
6.65	0.70889	0.70890	0.00015
6.61	0.70920	0.70890	0.00015
6.57	0.70892	0.70887	0.00013

6.52	0.70903	0.70886	0.00013
6.48	0.70838	0.70887	0.00014
6.44	0.70902	0.70889	0.00010
6.39	0.70887	0.70886	0.00010
6.35	0.70913	0.70884	0.00011
6.31	0.70873	0.70879	0.00009
6.26	0.70881	0.70882	0.00010
6.22	0.70887	0.70885	0.00012
6.18	0.70897	0.70883	0.00013
6.13	0.70877	0.70878	0.00014
6.09	0.70909	0.70877	0.00014
6.05	0.70865	0.70872	0.00012
6.00	0.70866	0.70876	0.00014
5.96	0.70868	0.70878	0.00014
5.92	0.70871	0.70883	0.00015
5.88	0.70899	0.70885	0.00015
5.83	0.70916	0.70884	0.00015
5.79	0.70858	0.70879	0.00014
5.75	0.70853	0.70879	0.00014
5.70	0.70861	0.70885	0.00015
5.66	0.70865	0.70886	0.00014
5.62	0.70906	0.70889	0.00013
5.57	0.70884	0.70886	0.00013
5.53	0.70914	0.70889	0.00014
5.49	0.70897	0.70890	0.00015
5.44	0.70888	0.70888	0.00015
5.40	0.70868	0.70884	0.00017
5.36	0.70850	0.70887	0.00016
5.31	0.70917	0.70890	0.00014
5.27	0.70872	0.70886	0.00013
5.23	0.70895	0.70887	0.00012
5.18	0.70876	0.70884	0.00013
5.14	0.70916	0.70883	0.00013
5.10	0.70922	0.70883	0.00013
5.05	0.70872	0.70880	0.00010
5.01	0.70857	0.70882	0.00010
4.97	0.70890	0.70886	0.00009
4.92	0.70882	0.70887	0.00009
4.88	0.70880	0.70888	0.00009
4.84	0.70883	0.70891	0.00010
4.80	0.70859	0.70894	0.00011
4.75	0.70872	0.70893	0.00013
4.71	0.70909	0.70894	0.00013
4.67	0.70894	0.70893	0.00012
4.62	0.70894	0.70891	0.00013
4.58	0.70901	0.70891	0.00013
4.54	0.70898	0.70888	0.00013
4.49	0.70892	0.70889	0.00014
4.45	0.70911	0.70890	0.00014
4.41	0.70913	0.70888	0.00014

4.36	0.70844	0.70882	0.00014
4.32	0.70887	0.70886	0.00011
4.28	0.70896	0.70884	0.00012
4.23	0.70876	0.70883	0.00011
4.19	0.70893	0.70886	0.00013
4.15	0.70866	0.70887	0.00013
4.10	0.70907	0.70887	0.00013
4.06	0.70910	0.70887	0.00014
4.02	0.70882	0.70883	0.00013
3.97	0.70857	0.70882	0.00013
3.93	0.70885	0.70885	0.00012
3.89	0.70864	0.70886	0.00012
3.84	0.70887	0.70891	0.00012
3.80	0.70913	0.70891	0.00012
3.76	0.70899	0.70887	0.00012
3.72	0.70861	0.70887	0.00011
3.67	0.70912	0.70889	0.00010
3.63	0.70865	0.70885	0.00009
3.59	0.70880	0.70885	0.00009
3.54	0.70882	0.70892	0.00014
3.50	0.70895	0.70894	0.00014
3.46	0.70916	0.70897	0.00016
3.41	0.70883	0.70891	0.00016
3.37	0.70878	0.70889	0.00017
3.33	0.70895	0.70893	0.00017
3.28	0.70881	0.70894	0.00018
3.24	0.70876	0.70897	0.00018
3.20	0.70867	0.70896	0.00019
3.15	0.70944	0.70897	0.00018
3.11	0.70900	0.70891	0.00015
3.07	0.70924	0.70895	0.00017
3.02	0.70864	0.70896	0.00018
2.98	0.70864	0.70898	0.00017
2.94	0.70911	0.70900	0.00016
2.89	0.70910	0.70898	0.00016
2.85	0.70911	0.70898	0.00016
2.81	0.70863	0.70900	0.00017
2.76	0.70876	0.70906	0.00015
2.72	0.70886	0.70904	0.00017
2.68	0.70937	0.70904	0.00017
2.64	0.70937	0.70903	0.00016
2.59	0.70885	0.70893	0.00018
2.55	0.70885	0.70896	0.00018
2.51	0.70893	0.70894	0.00019
2.46	0.70909	0.70895	0.00019
2.42	0.70930	0.70890	0.00020
2.38	0.70925	0.70889	0.00020
2.33	0.70859	0.70881	0.00020
2.29	0.70884	0.70883	0.00019
2.25	0.70920	0.70884	0.00019

2.20	0.70842	0.70880	0.00018
2.16	0.70913	0.70884	0.00015
2.12	0.70865	0.70874	0.00019
2.07	0.70902	0.70873	0.00019
2.03	0.70860	0.70873	0.00019
1.99	0.70923	0.70875	0.00019
1.94	0.70845	0.70868	0.00016
1.90	0.70875	0.70873	0.00016
1.86	0.70891	0.70880	0.00021
1.81	0.70881	0.70875	0.00022
1.77	0.70881	0.70876	0.00023
1.73	0.70813	0.70879	0.00024
1.68	0.70861	0.70889	0.00019
1.64	0.70897	0.70891	0.00018
1.60	0.70880	0.70888	0.00019
1.56	0.70859	0.70888	0.00019
1.51	0.70895	0.70890	0.00018
1.47	0.70944	0.70887	0.00019
1.43	0.70838	0.70885	0.00016
1.38	0.70891	0.70891	0.00012
1.34	0.70913	0.70891	0.00012
1.30	0.70911	0.70891	0.00012
1.25	0.70883	0.70891	0.00012
1.21	0.70864	0.70890	0.00012
1.17	0.70880	0.70893	0.00010
1.12	0.70885	0.70895	0.00010
1.08	0.70864	0.70897	0.00010
1.04	0.70917	0.70902	0.00008
0.99	0.70903	0.70897	0.00009
0.95	0.70895	0.70895	0.00010
0.91	0.70909	0.70893	0.00011
0.86	0.70907	0.70887	0.00013
0.82	0.70876	0.70883	0.00013
0.78	0.70892	0.70890	0.00017
0.73	0.70907	0.70888	0.00017
0.69	0.70902	0.70886	0.00017
0.65	0.70914	0.70886	0.00017
0.60	0.70868	0.70882	0.00016
0.56	0.70880	0.70884	0.00015
0.52	0.70876	0.70881	0.00017
0.48	0.70849	0.70885	0.00019
0.43	0.70869	0.70886	0.00018
0.39	0.70941	0.70889	0.00018
0.35	0.70875	0.70881	0.00014
0.30	0.70887	0.70882	0.00015
0.26	0.70904	0.70881	0.00016
0.22	0.70872	0.70878	0.00016
0.17	0.70888	0.70879	0.00017
0.13	0.70849	0.70877	0.00019
0.09	0.70919	0.70884	0.00019

0.04	0.70855

ARB 27.2.1 (M2)

Distance from cervix (mm)	$^{87}Sr/^{86}Sr$	10 point mov. average	2 SE on mov. average
26.90	0.70976	0.70969	0.00021
26.86	0.70905	0.70966	0.00021
26.82	0.70992	0.70970	0.00018
26.77	0.70947	0.70967	0.00017
26.73	0.70934	0.70967	0.00017
26.69	0.70982	0.70967	0.00017
26.65	0.70974	0.70967	0.00017
26.60	0.71015	0.70964	0.00018
26.56	0.70960	0.70962	0.00016
26.52	0.71004	0.70969	0.00020
26.47	0.70947	0.70969	0.00020
26.43	0.70941	0.70969	0.00020
26.39	0.70971	0.70967	0.00021
26.35	0.70946	0.70961	0.00024
26.30	0.70931	0.70959	0.00025
26.26	0.70980	0.70958	0.00025
26.22	0.70947	0.70960	0.00026
26.18	0.70998	0.70960	0.00026
26.13	0.71022	0.70961	0.00027
26.09	0.71008	0.70961	0.00027
26.05	0.70943	0.70952	0.00026
26.00	0.70929	0.70957	0.00028
25.96	0.70912	0.70956	0.00028
25.92	0.70926	0.70959	0.00027
25.88	0.70919	0.70960	0.00026
25.83	0.70998	0.70956	0.00030
25.79	0.70951	0.70947	0.00030
25.75	0.71005	0.70949	0.00030
25.70	0.71022	0.70948	0.00030
25.66	0.70913	0.70948	0.00029
25.62	0.70996	0.70943	0.00033
25.58	0.70917	0.70945	0.00034
25.53	0.70945	0.70947	0.00034
25.49	0.70930	0.70947	0.00034
25.45	0.70878	0.70949	0.00034
25.41	0.70912	0.70959	0.00030
25.36	0.70972	0.70967	0.00029
25.32	0.70998	0.70962	0.00031
25.28	0.71017	0.70954	0.00030
25.23	0.70866	0.70949	0.00027
25.19	0.71017	0.70962	0.00021
25.15	0.70932	0.70957	0.00017
25.11	0.70951	0.70961	0.00017
25.06	0.70942	0.70967	0.00019
25.02	0.70980	0.70970	0.00018
24.98	0.70994	0.70971	0.00019
24.94	0.70920	0.70963	0.00022

24.89	0.70923	0.70968	0.00019
24.85	0.70966	0.70969	0.00018
24.81	0.70992	0.70974	0.00020
24.76	0.70970	0.70976	0.00021
24.72	0.70970	0.70975	0.00021
24.68	0.71010	0.70976	0.00022
24.64	0.70972	0.70969	0.00021
24.59	0.70995	0.70969	0.00023
24.55	0.70912	0.70960	0.00024
24.51	0.70968	0.70966	0.00021
24.46	0.70936	0.70970	0.00023
24.42	0.71010	0.70965	0.00028
24.38	0.71014	0.70958	0.00026
24.34	0.70959	0.70952	0.00023
24.29	0.70987	0.70958	0.00026
24.25	0.70938	0.70959	0.00027
24.17	0.70915	0.70968	0.00028
24.12	0.70966	0.70974	0.00025
24.08	0.71008	0.70973	0.00025
24.04	0.70888	0.70964	0.00026
23.99	0.70943	0.70973	0.00020
23.95	0.70962	0.70982	0.00022
23.91	0.71013	0.70979	0.00024
23.87	0.71000	0.70979	0.00024
23.82	0.71021	0.70980	0.00024
23.78	0.70967	0.70977	0.00023
23.74	0.70970	0.70980	0.00023
23.69	0.70962	0.70988	0.00027
23.65	0.70918	0.70993	0.00027
23.61	0.70974	0.71000	0.00021
23.57	0.71037	0.70999	0.00021
23.52	0.70932	0.70983	0.00031
23.48	0.71009	0.70984	0.00031
23.44	0.71008	0.70980	0.00030
23.40	0.70988	0.70977	0.00029
23.35	0.71006	0.70974	0.00030
23.31	0.71051	0.70970	0.00029
23.27	0.71011	0.70965	0.00024
23.22	0.70982	0.70955	0.00024
23.18	0.70972	0.70958	0.00026
23.14	0.70877	0.70959	0.00026
23.10	0.70935	0.70968	0.00019
23.05	0.70973	0.70972	0.00017
23.01	0.70982	0.70975	0.00019
22.97	0.70956	0.70981	0.00022
22.92	0.70965	0.70988	0.00023
22.88	0.71003	0.70986	0.00024
22.84	0.70910	0.70986	0.00024
22.80	0.71006	0.70997	0.00019
22.75	0.70986	0.70999	0.00020

22.71	0.70968	0.71001	0.00020
22.67	0.70966	0.71005	0.00020
22.63	0.71010	0.71011	0.00019
22.58	0.71034	0.71011	0.00021
22.54	0.71027	0.71006	0.00022
22.50	0.70946	0.71001	0.00024
22.45	0.71006	0.71006	0.00017
22.41	0.71024	0.70985	0.00032
22.37	0.71027	0.70975	0.00028
22.07	0.70969	0.70983	0.00017
22.03	0.70920	0.70984	0.00017
21.98	0.70984	0.70989	0.00010
21.94	0.70993	0.70983	0.00016
21.90	0.70996	0.70982	0.00016
21.86	0.71009	0.70977	0.00017
21.81	0.71001	0.70971	0.00016
21.77	0.70976	0.70974	0.00019
21.73	0.71010	0.70974	0.00019
21.68	0.70968	0.70974	0.00019
21.64	0.70982	0.70976	0.00019
21.60	0.70971	0.70978	0.00020
21.56	0.70927	0.70979	0.00020
21.51	0.70979	0.70992	0.00021
21.47	0.70947	0.70998	0.00023
21.43	0.70951	0.71001	0.00021
21.39	0.71027	0.71004	0.00018
21.34	0.70978	0.71003	0.00017
21.30	0.71013	0.71006	0.00016
21.26	0.70990	0.71015	0.00026
21.21	0.71002	0.71017	0.00026
21.17	0.70981	0.71020	0.00025
21.13	0.71052	0.71024	0.00024
21.09	0.71042	0.71014	0.00026
21.04	0.70972	0.71003	0.00031
21.00	0.70985	0.71010	0.00031
20.96	0.71014	0.71013	0.00030
20.91	0.71009	0.71016	0.00031
20.87	0.71107	0.71019	0.00031
20.83	0.71004	0.71008	0.00024
20.79	0.71030	0.71002	0.00027
20.74	0.71021	0.70998	0.00027
20.70	0.70956	0.70994	0.00027
20.66	0.70928	0.71005	0.00028
20.62	0.71046	0.71007	0.00025
20.57	0.71014	0.71001	0.00024
20.53	0.71041	0.71000	0.00024
20.49	0.71041	0.70996	0.00022
20.44	0.71001	0.70994	0.00021
20.40	0.70945	0.70994	0.00022
20.36	0.70987	0.71000	0.00020

20.32	0.70985	0.71002	0.00022
20.27	0.71059	0.71004	0.00023
20.23	0.70955	0.70993	0.00017
20.19	0.70981	0.70985	0.00028
20.14	0.71008	0.70987	0.00028
20.10	0.70994	0.70988	0.00029
20.06	0.71028	0.70991	0.00029
19.80	0.70911	0.70993	0.00019
19.76	0.70996	0.71005	0.00009
19.72	0.71012	0.71009	0.00011
19.67	0.71011	0.71011	0.00012
19.63	0.71016	0.71014	0.00013
19.59	0.70981	0.71012	0.00014
19.55	0.70998	0.71010	0.00016
19.50	0.70990	0.71016	0.00019
19.46	0.71005	0.71010	0.00026
19.42	0.71007	0.71006	0.00028
19.37	0.71035	0.71005	0.00028
19.33	0.71037	0.71001	0.00027
19.29	0.71033	0.70991	0.00029
19.25	0.71039	0.70994	0.00031
19.20	0.70990	0.70990	0.00030
19.16	0.70966	0.70990	0.00030
19.12	0.71063	0.70992	0.00029
19.08	0.70922	0.70987	0.00025
19.03	0.70966	0.70991	0.00022
18.99	0.70996	0.70998	0.00023
18.95	0.70998	0.71003	0.00025
18.90	0.70935	0.71002	0.00025
18.86	0.71064	0.71008	0.00020
18.82	0.71004	0.71000	0.00017
18.78	0.70989	0.71010	0.00027
18.73	0.70988	0.71008	0.00028
18.69	0.71009	0.71008	0.00028
18.65	0.70958	0.71000	0.00032
18.61	0.71039	0.71006	0.00030
18.56	0.71045	0.71005	0.00030
18.52	0.70987	0.71007	0.00031
18.48	0.70997	0.71010	0.00031
18.43	0.70982	0.71010	0.00031
18.39	0.71107	0.71005	0.00034
18.35	0.70965	0.70996	0.00026
18.31	0.70987	0.70996	0.00026
18.26	0.70937	0.70998	0.00026
18.22	0.71019	0.71003	0.00023
18.18	0.71027	0.71006	0.00025
18.13	0.71065	0.70996	0.00030
18.09	0.71014	0.70991	0.00026
18.05	0.70994	0.70991	0.00026
18.01	0.70935	0.70988	0.00027

17.96	0.71019	0.70994	0.00024
17.92	0.70963	0.70991	0.00023
17.88	0.71011	0.70988	0.00026
17.84	0.70978	0.70988	0.00026
17.79	0.71057	0.70991	0.00026
17.75	0.70921	0.70988	0.00023
17.71	0.71020	0.70996	0.00018
17.66	0.71009	0.70994	0.00018
17.62	0.70966	0.70991	0.00017
17.58	0.70992	0.70995	0.00016
17.54	0.70995	0.70992	0.00018
17.49	0.70929	0.70989	0.00019
17.45	0.71009	0.70995	0.00013
17.41	0.71014	0.70996	0.00014
17.36	0.71024	0.70994	0.00013
17.32	0.71003	0.70993	0.00013
17.28	0.70996	0.70999	0.00018
17.24	0.70984	0.70994	0.00021
17.19	0.71005	0.70998	0.00021
17.15	0.70959	0.70994	0.00022
17.11	0.70964	0.71001	0.00021
17.07	0.70993	0.71005	0.00020
17.02	0.71019	0.71002	0.00021
16.98	0.70991	0.71004	0.00022
16.94	0.71020	0.71000	0.00024
16.89	0.71057	0.70998	0.00024
16.85	0.70950	0.70994	0.00021
16.81	0.71019	0.71001	0.00019
16.77	0.70968	0.71002	0.00020
16.72	0.71028	0.71010	0.00020
16.68	0.71003	0.71009	0.00020
16.64	0.70964	0.71011	0.00020
16.59	0.71040	0.71013	0.00018
16.55	0.70954	0.71008	0.00017
16.51	0.70995	0.71016	0.00013
16.47	0.71022	0.71013	0.00016
16.42	0.71012	0.71010	0.00017
16.38	0.71035	0.71004	0.00021
16.34	0.71048	0.71003	0.00020
16.30	0.71011	0.71003	0.00020
16.25	0.71028	0.71005	0.00021
16.21	0.70984	0.71001	0.00020
16.17	0.70993	0.71001	0.00020
16.12	0.71031	0.71003	0.00020
16.08	0.70964	0.70997	0.00020
16.04	0.70994	0.70994	0.00023
16.00	0.70949	0.70992	0.00023
15.95	0.71029	0.71003	0.00026
15.91	0.71044	0.71002	0.00025
15.87	0.71032	0.71005	0.00027

15.82	0.70992	0.71001	0.00027
15.78	0.70984	0.71000	0.00027
15.74	0.71008	0.70995	0.00030
15.70	0.70976	0.70994	0.00030
15.65	0.70930	0.70998	0.00030
15.61	0.70973	0.71008	0.00026
15.57	0.71067	0.71006	0.00027
15.53	0.71019	0.70994	0.00025
15.48	0.71067	0.70999	0.00029
15.44	0.70991	0.70998	0.00027
15.40	0.70982	0.70994	0.00029
15.35	0.70937	0.70995	0.00029
15.31	0.71000	0.71000	0.00026
15.27	0.71018	0.71007	0.00030
15.23	0.71022	0.71001	0.00031
15.18	0.70957	0.70994	0.00032
15.14	0.70948	0.71003	0.00032
15.10	0.71064	0.71008	0.00029
15.06	0.71057	0.71010	0.00031
15.01	0.70952	0.71007	0.00030
14.97	0.70994	0.71016	0.00028
14.93	0.70984	0.71016	0.00028
14.88	0.71071	0.71016	0.00028
14.84	0.70960	0.71014	0.00026
14.80	0.70958	0.71023	0.00025
14.76	0.71041	0.71026	0.00022
14.71	0.70999	0.71020	0.00023
14.67	0.71083	0.71023	0.00023
14.63	0.71024	0.71012	0.00019
14.58	0.71045	0.71010	0.00019
14.54	0.70992	0.71006	0.00018
14.50	0.70983	0.71008	0.00017
14.46	0.71049	0.71009	0.00017
14.41	0.71057	0.71003	0.00015
14.37	0.70985	0.70996	0.00009
14.33	0.70984	0.70997	0.00008
14.29	0.71022	0.70989	0.00019
14.24	0.70982	0.70994	0.00024
14.20	0.71003	0.70992	0.00025
14.16	0.71007	0.70986	0.00027
14.11	0.71009	0.70982	0.00027
14.07	0.70994	0.70976	0.00027
14.03	0.70984	0.70974	0.00027
13.99	0.70986	0.70973	0.00027
13.94	0.70996	0.70975	0.00027
13.90	0.70911	0.70972	0.00027
13.86	0.71069	0.70980	0.00024
13.81	0.70966	0.70971	0.00013
13.77	0.70936	0.70974	0.00014
13.73	0.70966	0.70974	0.00014

13.69	0.70947	0.70984	0.00023
13.64	0.70981	0.70983	0.00023
13.60	0.70974	0.70982	0.00024
13.56	0.71005	0.70983	0.00025
13.52	0.70963	0.70981	0.00024
13.47	0.70997	0.70981	0.00024
13.43	0.70973	0.70983	0.00025
13.39	0.70996	0.70980	0.00026
13.34	0.70939	0.70978	0.00026
13.30	0.71066	0.70985	0.00024
13.26	0.70939	0.70975	0.00015
13.22	0.70972	0.70982	0.00013
13.13	0.70985	0.70989	0.00016
13.09	0.70965	0.70996	0.00021
13.04	0.71015	0.71001	0.00020
13.00	0.70946	0.70999	0.00020
12.96	0.70974	0.71004	0.00016
12.92	0.70997	0.71012	0.00018
12.87	0.70978	0.71013	0.00017
12.83	0.71004	0.71016	0.00016
12.79	0.70989	0.71017	0.00016
12.75	0.71032	0.71013	0.00021
12.70	0.71059	0.71010	0.00021
12.66	0.71010	0.71003	0.00017
12.62	0.70995	0.71000	0.00017
12.57	0.71000	0.71004	0.00019
12.53	0.71055	0.71003	0.00019
12.49	0.71005	0.70994	0.00015
12.45	0.71011	0.70989	0.00016
12.36	0.70950	0.70989	0.00017
12.32	0.71004	0.71000	0.00020
12.28	0.71000	0.70998	0.00020
12.23	0.70980	0.70992	0.00023
12.19	0.71034	0.70986	0.00027
12.15	0.70986	0.70987	0.00028
12.10	0.70975	0.70984	0.00029
12.06	0.70964	0.70990	0.00031
12.02	0.71027	0.70994	0.00030
11.98	0.70970	0.70993	0.00030
11.93	0.71060	0.71000	0.00030
11.89	0.70984	0.70994	0.00027
11.85	0.70941	0.70987	0.00032
11.80	0.70921	0.70992	0.00030
11.76	0.71046	0.70998	0.00026
11.72	0.70953	0.70988	0.00026
11.68	0.71032	0.70995	0.00025
11.63	0.71011	0.70994	0.00025
11.59	0.71014	0.70999	0.00028
11.55	0.71039	0.70995	0.00028
11.51	0.71000	0.70989	0.00027

11.46	0.70911	0.70987	0.00027
11.42	0.70998	0.70995	0.00021
11.38	0.70974	0.70997	0.00022
11.33	0.70947	0.70996	0.00022
11.29	0.71024	0.70995	0.00024
11.25	0.71022	0.70995	0.00024
11.21	0.71061	0.70993	0.00023
11.16	0.70979	0.70985	0.00018
11.12	0.70973	0.70984	0.00018
11.08	0.70984	0.70989	0.00020
11.03	0.70985	0.70993	0.00020
10.99	0.71026	0.70998	0.00022
10.95	0.70959	0.70996	0.00021
10.91	0.70933	0.70996	0.00021
10.86	0.71027	0.71012	0.00023
10.82	0.71001	0.71004	0.00026
10.78	0.70985	0.70993	0.00034
10.74	0.70969	0.70993	0.00034
10.69	0.71025	0.70998	0.00034
10.65	0.71015	0.70997	0.00034
10.61	0.71036	0.70991	0.00035
10.56	0.71006	0.70990	0.00034
10.52	0.70963	0.70990	0.00034
10.48	0.71089	0.70987	0.00036
10.44	0.70948	0.70980	0.00030
10.39	0.70896	0.70985	0.00029
10.35	0.70979	0.70991	0.00022
10.31	0.71025	0.70983	0.00029
10.26	0.71014	0.70984	0.00030
10.22	0.70951	0.70981	0.00029
10.18	0.71031	0.70988	0.00029
10.14	0.71005	0.70989	0.00030
10.09	0.70930	0.70984	0.00030
10.05	0.71025	0.70996	0.00029
10.01	0.70993	0.70994	0.00029
9.97	0.70957	0.70996	0.00029
9.92	0.70898	0.70994	0.00030
9.88	0.71033	0.71001	0.00023
9.84	0.70991	0.71001	0.00023
9.79	0.71015	0.70995	0.00027
9.75	0.71042	0.70993	0.00027
9.71	0.70962	0.70986	0.00025
9.67	0.71045	0.70991	0.00024
9.62	0.71007	0.70978	0.00025
9.58	0.71014	0.70980	0.00026
9.54	0.70938	0.70978	0.00025
9.49	0.70962	0.70984	0.00024
9.45	0.71032	0.70987	0.00023
9.41	0.70929	0.70988	0.00025
9.37	0.71002	0.70996	0.00021

9.32	0.70974	0.70996	0.00021
9.28	0.71005	0.70996	0.00021
9.24	0.70919	0.70994	0.00021
9.20	0.71022	0.71002	0.00013
9.15	0.70995	0.70998	0.00012
9.11	0.71001	0.71003	0.00015
9.07	0.70985	0.71002	0.00015
9.02	0.71045	0.71003	0.00015
8.98	0.71013	0.70998	0.00011
8.94	0.71003	0.70992	0.00014
8.90	0.70974	0.70987	0.00016
8.85	0.70985	0.70988	0.00016
8.81	0.70997	0.70986	0.00016
8.77	0.70984	0.70975	0.00025
8.73	0.71038	0.70971	0.00026
8.68	0.70995	0.70967	0.00022
8.64	0.70991	0.70963	0.00021
8.60	0.71001	0.70959	0.00020
8.55	0.70955	0.70960	0.00021
8.51	0.70948	0.70964	0.00022
8.47	0.70985	0.70972	0.00025
8.43	0.70966	0.70973	0.00026
8.38	0.70891	0.70985	0.00034
8.34	0.70940	0.70991	0.00028
8.30	0.70995	0.70999	0.00026
8.25	0.70960	0.70999	0.00026
8.21	0.70951	0.71010	0.00027
8.17	0.71012	0.71013	0.00024
8.13	0.70991	0.71006	0.00028
8.08	0.71033	0.70999	0.00033
8.04	0.70991	0.71000	0.00033
8.00	0.71084	0.70996	0.00034
7.96	0.70953	0.70992	0.00030
7.91	0.71022	0.70998	0.00029
7.87	0.70999	0.70995	0.00028
7.83	0.71060	0.71005	0.00036
7.78	0.70981	0.70998	0.00034
7.74	0.70948	0.71002	0.00034
7.70	0.70921	0.71006	0.00032
7.66	0.71038	0.71018	0.00026
7.61	0.70957	0.71008	0.00029
7.57	0.71045	0.71017	0.00027
7.53	0.71008	0.71003	0.00034
7.48	0.70994	0.71001	0.00034
7.44	0.71103	0.70999	0.00034
7.40	0.70986	0.70990	0.00026
7.36	0.71017	0.70993	0.00026
7.31	0.70994	0.70986	0.00027
7.27	0.71036	0.70976	0.00033
7.23	0.70944	0.70968	0.00030

7.19	0.71039	0.70976	0.00032
7.14	0.70906	0.70974	0.00030
7.10	0.70986	0.70976	0.00028
7.06	0.70979	0.70978	0.00029
7.01	0.71009	0.70977	0.00029
6.97	0.71016	0.70976	0.00028
6.93	0.70952	0.70973	0.00027
6.89	0.70892	0.70971	0.00028
6.84	0.70955	0.70983	0.00022
6.80	0.71028	0.70986	0.00021
6.76	0.71017	0.70983	0.00020
6.71	0.70925	0.70980	0.00018
6.67	0.71002	0.70986	0.00013
6.63	0.70971	0.70981	0.00014
6.59	0.70997	0.70975	0.00020
6.54	0.70991	0.70973	0.00019
6.50	0.70935	0.70972	0.00019
6.46	0.71006	0.70970	0.00020
6.42	0.70985	0.70969	0.00019
6.37	0.71005	0.70963	0.00021
6.33	0.70986	0.70962	0.00020
6.29	0.70978	0.70954	0.00021
6.24	0.70954	0.70953	0.00021
6.20	0.70910	0.70952	0.00021
6.16	0.70977	0.70958	0.00019
6.12	0.70979	0.70960	0.00020
6.07	0.70922	0.70958	0.00020
6.03	0.70991	0.70961	0.00018
5.99	0.70927	0.70960	0.00018
5.95	0.70993	0.70968	0.00018
5.90	0.70911	0.70963	0.00017
5.86	0.70964	0.70969	0.00013
5.82	0.70945	0.70968	0.00013
5.77	0.70967	0.70975	0.00015
5.73	0.70999	0.70974	0.00016
5.69	0.70957	0.70968	0.00016
5.65	0.70954	0.70966	0.00017
5.60	0.70987	0.70970	0.00018
5.56	0.71001	0.70967	0.00017
5.52	0.70946	0.70966	0.00016
5.47	0.70972	0.70960	0.00024
5.43	0.70951	0.70959	0.00024
5.39	0.71016	0.70965	0.00026
5.35	0.70960	0.70959	0.00023
5.30	0.70936	0.70959	0.00023
5.26	0.70939	0.70959	0.00023
5.22	0.70989	0.70965	0.00024
5.18	0.70960	0.70960	0.00024
5.13	0.70992	0.70964	0.00025
5.09	0.70881	0.70956	0.00027

5.05	0.70969	0.70968	0.00022
5.00	0.71012	0.70972	0.00023
4.96	0.70949	0.70969	0.00021
4.92	0.70967	0.70972	0.00021
4.88	0.70936	0.70972	0.00021
4.83	0.70998	0.70975	0.00019
4.79	0.70937	0.70969	0.00020
4.75	0.71004	0.70969	0.00020
4.70	0.70912	0.70967	0.00019
4.66	0.71002	0.70966	0.00020
4.62	0.71002	0.70965	0.00019
4.58	0.70988	0.70961	0.00017
4.53	0.70974	0.70961	0.00017
4.49	0.70971	0.70961	0.00017
4.45	0.70963	0.70954	0.00020
4.41	0.70939	0.70953	0.00020
4.36	0.70940	0.70958	0.00020
4.32	0.70983	0.70962	0.00020
4.28	0.70903	0.70961	0.00020
4.23	0.70984	0.70966	0.00016
4.19	0.70962	0.70962	0.00015
4.15	0.70988	0.70961	0.00016
4.11	0.70977	0.70961	0.00015
4.06	0.70906	0.70957	0.00015
4.02	0.70954	0.70962	0.00010
3.98	0.70983	0.70964	0.00010
3.93	0.70977	0.70964	0.00010
3.89	0.70979	0.70962	0.00010
3.85	0.70947	0.70956	0.00012
3.81	0.70951	0.70954	0.00014
3.76	0.70952	0.70956	0.00015
3.72	0.70981	0.70957	0.00015
3.68	0.70943	0.70960	0.00017
3.64	0.70953	0.70964	0.00017
3.59	0.70978	0.70965	0.00017
3.55	0.70980	0.70964	0.00017
3.51	0.70960	0.70961	0.00017
3.46	0.70921	0.70957	0.00019
3.42	0.70919	0.70964	0.00017
3.38	0.70975	0.70962	0.00019
3.34	0.70966	0.70968	0.00023
3.29	0.71006	0.70971	0.00024
3.25	0.70984	0.70966	0.00023
3.21	0.70959	0.70961	0.00024
3.16	0.70972	0.70961	0.00024
3.12	0.70947	0.70959	0.00024
3.08	0.70924	0.70964	0.00025
3.04	0.70987	0.70966	0.00023
2.99	0.70905	0.70965	0.00023
2.95	0.71028	0.70968	0.00020

2.91	0.71003	0.70962	0.00015
2.87	0.70956	0.70958	0.00012
2.82	0.70927	0.70962	0.00014
2.78	0.70960	0.70971	0.00017
2.74	0.70955	0.70973	0.00017
2.69	0.70995	0.70973	0.00017
2.65	0.70947	0.70969	0.00016
2.61	0.70971	0.70975	0.00016
2.57	0.70934	0.70972	0.00017
2.52	0.70968	0.70978	0.00015
2.48	0.70966	0.70980	0.00015
2.44	0.70994	0.70980	0.00015
2.40	0.71024	0.70975	0.00017
2.35	0.70976	0.70969	0.00013
2.31	0.70954	0.70975	0.00018
2.27	0.70959	0.70976	0.00017
2.22	0.71002	0.70979	0.00017
2.18	0.70946	0.70979	0.00017
2.14	0.70987	0.70978	0.00018
2.10	0.70989	0.70981	0.00019
2.05	0.70972	0.70975	0.00021
2.01	0.70939	0.70977	0.00021
1.97	0.70970	0.70978	0.00021
1.92	0.71032	0.70977	0.00021
1.88	0.70968	0.70978	0.00023
1.84	0.70989	0.70982	0.00024
1.80	0.70999	0.70983	0.00024
1.75	0.70935	0.70986	0.00025
1.71	0.71015	0.70989	0.00023
1.67	0.70935	0.70984	0.00023
1.63	0.70993	0.70985	0.00022
1.58	0.70944	0.70983	0.00022
1.54	0.70959	0.70989	0.00021
1.50	0.71045	0.70991	0.00020
1.45	0.71009	0.70983	0.00017
1.41	0.70997	0.70972	0.00022
1.37	0.71027	0.70968	0.00021
1.33	0.70970	0.70961	0.00017
1.28	0.70966	0.70960	0.00017
1.24	0.70938	0.70963	0.00018
1.20	0.70978	0.70965	0.00018
1.15	0.71004	0.70967	0.00019
1.11	0.70973	0.70958	0.00020
1.07	0.70965	0.70957	0.00019
1.03	0.70904	0.70956	0.00021
0.98	0.70959	0.70963	0.00017
0.94	0.70951	0.70964	0.00019
0.90	0.70963	0.70966	0.00020
0.86	0.70997	0.70966	0.00023
0.81	0.70952	0.70959	0.00023

0.77	0.70999	0.70961	0.00028
0.73	0.70913	0.70941	0.00025
0.68	0.70970		

ARB 29.2.1 (M2)

Distance from cervix (mm)	$^{87}Sr/^{86}Sr$	10 point mov. average	2 SE on mov. average
42.63	0.70995	0.70997	0.00009
42.59	0.70985	0.70995	0.00010
42.54	0.70972	0.70996	0.00009
42.50	0.70982	0.70997	0.00009
42.46	0.71011	0.71002	0.00010
42.41	0.71013	0.70998	0.00012
42.37	0.71004	0.71000	0.00014
42.33	0.71009	0.70998	0.00015
42.28	0.70998	0.70997	0.00014
42.24	0.71004	0.70998	0.00015
42.20	0.70974	0.70998	0.00015
42.15	0.70995	0.71003	0.00015
42.11	0.70981	0.71006	0.00015
42.07	0.71031	0.71011	0.00015
42.02	0.70966	0.71007	0.00014
41.98	0.71037	0.71013	0.00012
41.94	0.70984	0.71011	0.00011
41.89	0.70999	0.71013	0.00009
41.85	0.71013	0.71015	0.00008
41.80	0.70994	0.71019	0.00011
41.76	0.71028	0.71022	0.00009
41.72	0.71022	0.71021	0.00009
41.67	0.71031	0.71018	0.00011
41.63	0.70997	0.71016	0.00011
41.59	0.71029	0.71015	0.00011
41.54	0.71019	0.71009	0.00014
41.50	0.71004	0.71009	0.00014
41.46	0.71019	0.71006	0.00016
41.41	0.71050	0.71008	0.00017
41.37	0.71024	0.71004	0.00014
41.33	0.71018	0.71006	0.00015
41.28	0.70993	0.71006	0.00016
41.24	0.71006	0.71010	0.00016
41.20	0.70994	0.71009	0.00017
41.15	0.70968	0.71011	0.00016
41.11	0.71017	0.71017	0.00013
41.07	0.70974	0.71017	0.00013
41.02	0.71041	0.71021	0.00010
40.98	0.71006	0.71020	0.00009
40.94	0.71038	0.71021	0.00008
40.89	0.71026	0.71019	0.00008
40.85	0.71030	0.71019	0.00008
40.81	0.70992	0.71015	0.00010
40.76	0.71020	0.71016	0.00009
40.72	0.71029	0.71012	0.00011
40.68	0.71015	0.71007	0.00012
40.63	0.71015	0.71007	0.00012

40.59	0.71032	0.71004	0.00012
40.55	0.71015	0.71002	0.00011
40.50	0.71012	0.70998	0.00012
40.46	0.71033	0.70994	0.00012
40.42	0.70987	0.70992	0.00009
40.37	0.71001	0.70993	0.00009
40.33	0.70982	0.70991	0.00010
40.29	0.70981	0.70991	0.00009
40.24	0.71006	0.70996	0.00012
40.20	0.70991	0.71001	0.00016
40.16	0.71012	0.71005	0.00017
40.11	0.70970	0.71005	0.00017
40.07	0.70981	0.71010	0.00016
40.02	0.71011	0.71012	0.00014
39.98	0.70998	0.71014	0.00015
39.94	0.70975	0.71019	0.00016
39.89	0.70988	0.71022	0.00013
39.85	0.71029	0.71026	0.00010
39.81	0.71054	0.71023	0.00011
39.76	0.71030	0.71013	0.00016
39.72	0.71015	0.71010	0.00016
39.68	0.71017	0.71006	0.00017
39.63	0.71006	0.71009	0.00019
39.59	0.71030	0.71012	0.00019
39.55	0.71048	0.71008	0.00019
39.50	0.71007	0.71006	0.00018
39.46	0.71025	0.71007	0.00018
39.42	0.71000	0.71006	0.00018
39.37	0.70953	0.71001	0.00021
39.33	0.70998	0.71007	0.00018
39.29	0.70980	0.71006	0.00018
39.24	0.71047	0.71004	0.00019
39.20	0.71031	0.71000	0.00017
39.16	0.70987	0.70995	0.00016
39.11	0.71027	0.70997	0.00016
39.07	0.71026	0.71000	0.00019
39.03	0.71012	0.70996	0.00018
38.98	0.70953	0.70995	0.00018
38.94	0.71005	0.71000	0.00016
38.90	0.70988	0.70997	0.00016
38.85	0.70962	0.71003	0.00019
38.81	0.71009	0.71010	0.00017
38.77	0.70977	0.71007	0.00018
38.72	0.71010	0.71012	0.00016
38.68	0.71054	0.71017	0.00018
38.64	0.70993	0.71015	0.00017
38.59	0.70998	0.71020	0.00017
38.51	0.70984	0.71019	0.00017
38.46	0.71043	0.71023	0.00015
38.42	0.71026	0.71021	0.00015

38.38	0.70981	0.71020	0.00015
38.33	0.71023	0.71027	0.00013
38.29	0.71053	0.71024	0.00014
38.25	0.71032	0.71021	0.00013
38.20	0.71043	0.71020	0.00013
38.16	0.71020	0.71018	0.00011
38.11	0.70982	0.71017	0.00011
38.07	0.71027	0.71022	0.00009
38.03	0.71027	0.71021	0.00009
37.98	0.71014	0.71023	0.00010
37.94	0.71049	0.71024	0.00009
37.90	0.70996	0.71024	0.00009
37.85	0.71020	0.71028	0.00007
37.81	0.71021	0.71028	0.00007
37.77	0.71020	0.71026	0.00008
37.72	0.71016	0.71029	0.00009
37.68	0.71030	0.71026	0.00011
37.64	0.71022	0.71026	0.00011
37.59	0.71043	0.71027	0.00011
37.55	0.71029	0.71024	0.00011
37.51	0.71048	0.71026	0.00011
37.46	0.71032	0.71020	0.00012
37.42	0.71017	0.71019	0.00012
37.38	0.71006	0.71018	0.00012
37.33	0.71043	0.71023	0.00014
37.29	0.70990	0.71022	0.00014
37.25	0.71033	0.71028	0.00013
37.20	0.71029	0.71031	0.00015
37.16	0.71015	0.71029	0.00015
37.12	0.71042	0.71032	0.00015
37.07	0.70990	0.71029	0.00015
37.03	0.71020	0.71034	0.00013
36.99	0.71008	0.71035	0.00012
36.94	0.71058	0.71040	0.00012
36.90	0.71036	0.71037	0.00011
36.86	0.71045	0.71038	0.00011
36.81	0.71066	0.71036	0.00012
36.77	0.71010	0.71035	0.00011
36.73	0.71041	0.71038	0.00009
36.68	0.71016	0.71037	0.00009
36.64	0.71035	0.71038	0.00008
36.60	0.71033	0.71035	0.00010
36.55	0.71061	0.71038	0.00011
36.51	0.71024	0.71037	0.00010
36.47	0.71048	0.71035	0.00012
36.42	0.71027	0.71034	0.00011
36.38	0.71057	0.71037	0.00012
36.33	0.71034	0.71037	0.00012
36.29	0.71032	0.71038	0.00012
36.25	0.71031	0.71037	0.00012

36.20	0.71007	0.71041	0.00014
36.16	0.71059	0.71045	0.00011
36.12	0.71049	0.71042	0.00011
36.07	0.71007	0.71045	0.00013
36.03	0.71038	0.71046	0.00013
35.99	0.71060	0.71049	0.00013
35.94	0.71053	0.71049	0.00014
35.90	0.71044	0.71049	0.00014
35.86	0.71026	0.71049	0.00014
35.81	0.71070	0.71047	0.00015
35.77	0.71046	0.71043	0.00015
35.73	0.71031	0.71046	0.00016
35.68	0.71079	0.71046	0.00016
35.64	0.71013	0.71046	0.00016
35.60	0.71066	0.71050	0.00014
35.55	0.71066	0.71048	0.00014
35.51	0.71049	0.71046	0.00014
35.47	0.71044	0.71049	0.00014
35.42	0.71006	0.71051	0.00015
35.38	0.71034	0.71059	0.00013
35.34	0.71073	0.71066	0.00013
35.29	0.71027	0.71064	0.00013
35.25	0.71080	0.71065	0.00013
35.21	0.71056	0.71063	0.00012
35.16	0.71040	0.71061	0.00013
35.12	0.71054	0.71064	0.00013
35.08	0.71070	0.71064	0.00013
35.03	0.71073	0.71062	0.00013
34.99	0.71086	0.71062	0.00013
34.95	0.71096	0.71060	0.00012
34.90	0.71058	0.71057	0.00009
34.86	0.71034	0.71055	0.00009
34.82	0.71062	0.71059	0.00008
34.77	0.71037	0.71063	0.00012
34.73	0.71075	0.71066	0.00010
34.69	0.71053	0.71063	0.00011
34.64	0.71051	0.71062	0.00011
34.60	0.71070	0.71064	0.00011
34.56	0.71065	0.71064	0.00011
34.51	0.71065	0.71063	0.00011
34.47	0.71040	0.71062	0.00011
34.42	0.71070	0.71065	0.00010
34.38	0.71100	0.71066	0.00011
34.34	0.71069	0.71060	0.00008
34.29	0.71045	0.71057	0.00010
34.25	0.71048	0.71054	0.00012
34.21	0.71063	0.71055	0.00012
34.16	0.71074	0.71057	0.00014
34.12	0.71058	0.71055	0.00013
34.08	0.71051	0.71058	0.00014

34.03	0.71068	0.71059	0.00014
33.99	0.71080	0.71057	0.00014
33.95	0.71048	0.71053	0.00014
33.90	0.71029	0.71056	0.00014
33.86	0.71018	0.71059	0.00013
33.82	0.71056	0.71066	0.00010
33.77	0.71086	0.71070	0.00011
33.73	0.71056	0.71068	0.00011
33.69	0.71086	0.71069	0.00010
33.64	0.71063	0.71069	0.00011
33.60	0.71049	0.71068	0.00011
33.56	0.71038	0.71070	0.00010
33.51	0.71075	0.71069	0.00012
33.47	0.71067	0.71070	0.00013
33.43	0.71083	0.71071	0.00013
33.38	0.71091	0.71069	0.00013
33.34	0.71072	0.71068	0.00012
33.30	0.71063	0.71069	0.00013
33.25	0.71087	0.71071	0.00013
33.21	0.71050	0.71071	0.00013
33.17	0.71076	0.71073	0.00012
33.12	0.71026	0.71074	0.00012
33.08	0.71087	0.71077	0.00008
33.04	0.71077	0.71072	0.00011
32.99	0.71060	0.71072	0.00011
32.95	0.71083	0.71077	0.00013
32.91	0.71084	0.71077	0.00014
32.86	0.71077	0.71077	0.00014
32.82	0.71092	0.71077	0.00014
32.78	0.71072	0.71073	0.00014
32.73	0.71086	0.71072	0.00014
32.69	0.71051	0.71071	0.00014
32.65	0.71035	0.71075	0.00014
32.60	0.71082	0.71077	0.00011
32.56	0.71110	0.71076	0.00011
32.47	0.71086	0.71075	0.00009
32.43	0.71077	0.71073	0.00009
32.38	0.71062	0.71070	0.00011
32.34	0.71057	0.71072	0.00011
32.30	0.71079	0.71074	0.00010
32.25	0.71086	0.71074	0.00010
32.21	0.71051	0.71072	0.00010
32.17	0.71074	0.71073	0.00009
32.12	0.71097	0.71072	0.00009
32.08	0.71079	0.71073	0.00011
32.04	0.71072	0.71074	0.00011
31.99	0.71043	0.71072	0.00013
31.95	0.71084	0.71074	0.00011
31.91	0.71077	0.71070	0.00013
31.86	0.71072	0.71068	0.00013

31.82	0.71075	0.71065	0.00014
31.78	0.71056	0.71055	0.00022
31.73	0.71068	0.71056	0.00023
31.69	0.71108	0.71052	0.00023
31.65	0.71089	0.71048	0.00020
31.60	0.71047	0.71043	0.00018
31.56	0.71064	0.71044	0.00019
31.52	0.71040	0.71046	0.00020
31.47	0.71057	0.71046	0.00020
31.43	0.71043	0.71041	0.00021
31.39	0.70973	0.71039	0.00021
31.34	0.71069	0.71042	0.00018
31.30	0.71030	0.71037	0.00017
31.26	0.71071	0.71038	0.00017
31.21	0.71030	0.71036	0.00016
31.17	0.71065	0.71038	0.00016
31.13	0.71079	0.71038	0.00016
31.08	0.71044	0.71036	0.00014
31.04	0.71007	0.71032	0.00015
31.00	0.71027	0.71033	0.00015
30.95	0.70998	0.71034	0.00014
30.91	0.71016	0.71037	0.00012
30.87	0.71042	0.71042	0.00013
30.82	0.71053	0.71041	0.00014
30.78	0.71048	0.71041	0.00014
30.73	0.71065	0.71041	0.00014
30.69	0.71056	0.71040	0.00013
30.65	0.71011	0.71039	0.00013
30.60	0.71011	0.71043	0.00011
30.56	0.71035	0.71044	0.00010
30.52	0.71030	0.71043	0.00010
30.47	0.71073	0.71038	0.00017
30.43	0.71025	0.71033	0.00015
30.39	0.71059	0.71031	0.00016
30.34	0.71048	0.71025	0.00016
30.30	0.71047	0.71022	0.00015
30.26	0.71045	0.71021	0.00014
30.21	0.71055	0.71017	0.00013
30.17	0.71025	0.71014	0.00011
30.13	0.71027	0.71013	0.00010
30.08	0.70979	0.71013	0.00011
30.04	0.71021	0.71016	0.00008
30.00	0.71001	0.71016	0.00008
29.95	0.71002	0.71017	0.00007
29.91	0.71017	0.71020	0.00007
29.87	0.71039	0.71022	0.00008
29.82	0.71009	0.71019	0.00008
29.78	0.71021	0.71022	0.00008
29.74	0.71011	0.71024	0.00009
29.69	0.71030	0.71026	0.00009

29.65	0.71009	0.71026	0.00009
29.61	0.71019	0.71027	0.00008
29.56	0.71008	0.71030	0.00009
29.52	0.71032	0.71031	0.00007
29.48	0.71041	0.71031	0.00007
29.43	0.71008	0.71029	0.00007
29.39	0.71039	0.71030	0.00006
29.35	0.71045	0.71028	0.00007
29.30	0.71030	0.71024	0.00007
29.26	0.71025	0.71023	0.00007
29.22	0.71022	0.71025	0.00008
29.17	0.71046	0.71024	0.00009
29.13	0.71024	0.71023	0.00008
29.09	0.71028	0.71024	0.00008
29.04	0.71025	0.71024	0.00008
29.00	0.71015	0.71022	0.00009
28.96	0.71015	0.71026	0.00011
28.91	0.71005	0.71025	0.00011
28.87	0.71026	0.71029	0.00011
28.82	0.71047	0.71030	0.00011
28.78	0.71009	0.71029	0.00011
28.74	0.71037	0.71033	0.00010
28.69	0.71030	0.71031	0.00010
28.65	0.71031	0.71028	0.00012
28.61	0.71005	0.71025	0.00013
28.56	0.71054	0.71031	0.00014
28.52	0.71006	0.71030	0.00014
28.48	0.71042	0.71030	0.00013
28.43	0.71037	0.71031	0.00014
28.39	0.71042	0.71033	0.00015
28.35	0.71044	0.71032	0.00015
28.30	0.71018	0.71033	0.00016
28.26	0.71003	0.71037	0.00017
28.22	0.71000	0.71042	0.00015
28.17	0.71062	0.71045	0.00011
28.13	0.71045	0.71047	0.00012
28.09	0.71007	0.71042	0.00015
28.04	0.71054	0.71045	0.00013
28.00	0.71060	0.71043	0.00013
27.96	0.71024	0.71042	0.00013
27.91	0.71062	0.71044	0.00012
27.87	0.71059	0.71047	0.00015
27.83	0.71044	0.71045	0.00015
27.78	0.71038	0.71044	0.00015
27.74	0.71072	0.71042	0.00016
27.70	0.71003	0.71044	0.00018
27.65	0.71037	0.71047	0.00015
27.61	0.71030	0.71050	0.00016
27.57	0.71047	0.71056	0.00016
27.52	0.71043	0.71058	0.00016

27.48	0.71091	0.71058	0.00016
27.44	0.71047	0.71056	0.00015
27.39	0.71027	0.71057	0.00015
27.35	0.71020	0.71060	0.00014
27.31	0.71090	0.71063	0.00011
27.26	0.71040	0.71064	0.00013
27.22	0.71067	0.71073	0.00015
27.18	0.71083	0.71070	0.00016
27.13	0.71072	0.71070	0.00016
27.09	0.71048	0.71070	0.00016
27.05	0.71065	0.71073	0.00015
27.00	0.71058	0.71077	0.00017
26.91	0.71045	0.71079	0.00016
26.87	0.71103	0.71082	0.00014
26.83	0.71115	0.71080	0.00013
26.78	0.71042	0.71073	0.00012
26.74	0.71082	0.71074	0.00011
26.70	0.71073	0.71074	0.00011
26.65	0.71075	0.71075	0.00011
26.61	0.71103	0.71076	0.00011
26.57	0.71091	0.71075	0.00010
26.52	0.71062	0.71076	0.00011
26.48	0.71073	0.71077	0.00011
26.44	0.71081	0.71077	0.00011
26.39	0.71047	0.71077	0.00011
26.35	0.71052	0.71078	0.00010
26.31	0.71087	0.71083	0.00008
26.26	0.71084	0.71081	0.00009
26.22	0.71081	0.71082	0.00009
26.18	0.71089	0.71079	0.00011
26.13	0.71106	0.71073	0.00015
26.09	0.71074	0.71071	0.00013
26.05	0.71074	0.71072	0.00013
26.00	0.71078	0.71072	0.00013
25.96	0.71058	0.71074	0.00014
25.92	0.71096	0.71073	0.00014
25.87	0.71072	0.71076	0.00016
25.83	0.71096	0.71078	0.00017
25.79	0.71048	0.71074	0.00017
25.74	0.71031	0.71078	0.00016
25.70	0.71078	0.71083	0.00013
25.66	0.71086	0.71083	0.00013
25.61	0.71076	0.71079	0.00015
25.57	0.71095	0.71080	0.00015
25.53	0.71053	0.71077	0.00014
25.48	0.71119	0.71077	0.00014
25.44	0.71099	0.71072	0.00011
25.40	0.71055	0.71072	0.00011
25.35	0.71091	0.71069	0.00014
25.31	0.71075	0.71066	0.00013

25.27	0.71075	0.71064	0.00013
25.22	0.71046	0.71061	0.00013
25.18	0.71086	0.71061	0.00013
25.13	0.71075	0.71059	0.00012
25.09	0.71051	0.71060	0.00012
25.05	0.71069	0.71061	0.00012
25.00	0.71095	0.71059	0.00012
24.96	0.71027	0.71055	0.00009
24.92	0.71058	0.71060	0.00008
24.87	0.71059	0.71059	0.00008
24.83	0.71047	0.71058	0.00008
24.79	0.71044	0.71059	0.00008
24.74	0.71065	0.71063	0.00009
24.70	0.71080	0.71065	0.00010
24.66	0.71062	0.71063	0.00009
24.61	0.71055	0.71060	0.00011
24.57	0.71048	0.71066	0.00014
24.53	0.71079	0.71065	0.00015
24.48	0.71048	0.71061	0.00015
24.44	0.71053	0.71064	0.00015
24.40	0.71053	0.71060	0.00017
24.35	0.71087	0.71059	0.00018
24.31	0.71083	0.71051	0.00019
24.27	0.71057	0.71046	0.00018
24.22	0.71039	0.71039	0.00022
24.18	0.71111	0.71037	0.00022
24.14	0.71043	0.71023	0.00018
24.09	0.71040	0.71020	0.00018
24.05	0.71071	0.71018	0.00017
24.01	0.71019	0.71012	0.00012
23.96	0.71038	0.71009	0.00013
23.92	0.71010	0.71007	0.00011
23.88	0.71034	0.71005	0.00012
23.83	0.70980	0.71001	0.00010
23.79	0.71018	0.71002	0.00009
23.75	0.70978	0.70997	0.00010
23.70	0.71015	0.71002	0.00010
23.66	0.71015	0.71002	0.00010
23.62	0.71011	0.71000	0.00010
23.57	0.70992	0.70999	0.00010
23.53	0.71012	0.70999	0.00010
23.49	0.70992	0.70991	0.00014
23.44	0.70997	0.70993	0.00014
23.40	0.70991	0.70990	0.00015
23.36	0.70973	0.70990	0.00015
23.31	0.71024	0.70992	0.00014
23.27	0.71015	0.70988	0.00012
23.22	0.70992	0.70987	0.00011
23.14	0.70992	0.70991	0.00013
23.09	0.70946	0.70992	0.00014

23.05	0.71003	0.70999	0.00010
23.01	0.70978	0.70999	0.00010
22.96	0.70987	0.71001	0.00008
22.92	0.70995	0.71003	0.00008
22.88	0.70982	0.71001	0.00009
22.83	0.71006	0.71004	0.00008
22.79	0.71025	0.71002	0.00009
22.75	0.70991	0.70998	0.00008
22.70	0.71011	0.71000	0.00008
22.66	0.71012	0.70997	0.00009
22.62	0.71004	0.70995	0.00008
22.57	0.71000	0.70992	0.00009
22.53	0.71005	0.70991	0.00009
22.49	0.70977	0.70990	0.00009
22.44	0.71010	0.70991	0.00008
22.40	0.70986	0.70986	0.00009
22.36	0.70990	0.70987	0.00009
22.31	0.71010	0.70991	0.00012
22.27	0.70974	0.70992	0.00013
22.23	0.70996	0.70993	0.00012
22.18	0.70971	0.70992	0.00012
22.14	0.70994	0.70999	0.00015
22.10	0.70989	0.71002	0.00015
22.05	0.70990	0.71006	0.00016
22.01	0.70964	0.71008	0.00015
21.97	0.70989	0.71012	0.00012
21.92	0.71029	0.71013	0.00011
21.88	0.71021	0.71014	0.00012
21.84	0.70990	0.71016	0.00012
21.79	0.70987	0.71014	0.00014
21.75	0.71040	0.71019	0.00013
21.71	0.71022	0.71020	0.00014
21.66	0.71027	0.71022	0.00015
21.62	0.71010	0.71028	0.00019
21.58	0.71002	0.71030	0.00019
21.53	0.71007	0.71033	0.00018
21.49	0.71037	0.71035	0.00017
21.45	0.71036	0.71036	0.00017
21.40	0.70973	0.71036	0.00017
21.36	0.71035	0.71046	0.00011
21.31	0.71047	0.71048	0.00011
21.27	0.71048	0.71050	0.00011
21.23	0.71083	0.71053	0.00013
21.18	0.71029	0.71054	0.00015
21.14	0.71036	0.71056	0.00014
21.10	0.71027	0.71056	0.00014
21.05	0.71041	0.71064	0.00015
21.01	0.71044	0.71064	0.00016
20.97	0.71066	0.71063	0.00017
20.92	0.71056	0.71063	0.00017

20.88	0.71066	0.71061	0.00017
20.84	0.71079	0.71059	0.00017
20.79	0.71100	0.71058	0.00017
20.75	0.71046	0.71054	0.00014
20.71	0.71039	0.71057	0.00015
20.66	0.71107	0.71062	0.00015
20.62	0.71037	0.71058	0.00012
20.58	0.71030	0.71058	0.00012
20.53	0.71066	0.71063	0.00010
20.49	0.71039	0.71062	0.00011
20.45	0.71048	0.71062	0.00010
20.40	0.71067	0.71063	0.00010
20.36	0.71062	0.71063	0.00010
20.32	0.71078	0.71064	0.00010
20.27	0.71082	0.71064	0.00010
20.23	0.71074	0.71063	0.00010
20.19	0.71037	0.71060	0.00011
20.14	0.71077	0.71063	0.00009
20.10	0.71055	0.71058	0.00010
20.06	0.71044	0.71057	0.00011
20.01	0.71055	0.71057	0.00011
19.97	0.71065	0.71058	0.00011
19.93	0.71076	0.71057	0.00011
19.88	0.71073	0.71053	0.00010
19.84	0.71079	0.71053	0.00010
19.80	0.71036	0.71052	0.00008
19.75	0.71067	0.71058	0.00013
19.71	0.71034	0.71063	0.00016
19.67	0.71041	0.71062	0.00017
19.62	0.71048	0.71062	0.00017
19.58	0.71058	0.71061	0.00017
19.53	0.71057	0.71061	0.00019
19.49	0.71041	0.71064	0.00019
19.45	0.71073	0.71064	0.00019
19.40	0.71061	0.71058	0.00021
19.36	0.71104	0.71056	0.00021
19.32	0.71110	0.71052	0.00018
19.27	0.71031	0.71052	0.00018
19.23	0.71034	0.71055	0.00018
19.19	0.71039	0.71057	0.00017
19.10	0.71085	0.71058	0.00016
19.06	0.71038	0.71051	0.00016
19.01	0.71023	0.71050	0.00017
18.97	0.71046	0.71052	0.00016
18.93	0.71063	0.71053	0.00016
18.88	0.71110	0.71051	0.00016
18.84	0.71061	0.71046	0.00010
18.80	0.71046	0.71042	0.00010
18.75	0.71056	0.71044	0.00011
18.71	0.71052	0.71046	0.00012

18.67	0.71016	0.71047	0.00013
18.62	0.71029	0.71051	0.00011
18.58	0.71041	0.71055	0.00011
18.54	0.71061	0.71055	0.00011
18.49	0.71037	0.71052	0.00012
18.45	0.71063	0.71051	0.00012
18.41	0.71024	0.71046	0.00014
18.36	0.71059	0.71048	0.00013
18.32	0.71075	0.71046	0.00013
18.28	0.71065	0.71039	0.00011
18.19	0.71071	0.71041	0.00011
18.15	0.71037	0.71037	0.00009
18.10	0.71036	0.71039	0.00010
18.06	0.71030	0.71041	0.00010
18.02	0.71018	0.71039	0.00011
17.97	0.71039	0.71041	0.00010
17.93	0.71040	0.71047	0.00014
17.89	0.71020	0.71044	0.00016
17.84	0.71065	0.71045	0.00015
17.80	0.71056	0.71042	0.00015
17.76	0.71032	0.71037	0.00017
17.71	0.71051	0.71032	0.00019
17.67	0.71057	0.71034	0.00020
17.62	0.71016	0.71029	0.00020
17.58	0.71037	0.71031	0.00019
17.54	0.71093	0.71028	0.00020
17.49	0.71011	0.71021	0.00013
17.45	0.71032	0.71021	0.00013
17.41	0.71040	0.71022	0.00013
17.36	0.70999	0.71020	0.00013
17.32	0.70989	0.71023	0.00012
17.28	0.71063	0.71025	0.00010
17.23	0.71013	0.71020	0.00006
17.19	0.71027	0.71023	0.00008
17.15	0.71010	0.71022	0.00008
17.10	0.71020	0.71023	0.00007
17.06	0.71019	0.71023	0.00007
17.02	0.71036	0.71029	0.00013
16.97	0.71023	0.71033	0.00016
16.93	0.71027	0.71030	0.00017
16.89	0.71012	0.71028	0.00018
16.84	0.71006	0.71030	0.00018
16.80	0.71045	0.71028	0.00020
16.76	0.71019	0.71025	0.00020
16.71	0.71021	0.71026	0.00020
16.67	0.71024	0.71027	0.00020
16.63	0.71076	0.71027	0.00020
16.58	0.71077	0.71021	0.00017
16.54	0.70995	0.71015	0.00011
16.50	0.71002	0.71017	0.00010

16.45	0.71037	0.71020	0.00011
16.41	0.70980	0.71018	0.00010
16.37	0.71020	0.71022	0.00006
16.32	0.71024	0.71021	0.00006
16.28	0.71030	0.71017	0.00009
16.24	0.71031	0.71016	0.00009
16.19	0.71015	0.71008	0.00015
16.15	0.71016	0.71007	0.00015
16.11	0.71012	0.71004	0.00015
16.06	0.71039	0.71002	0.00015
16.02	0.71011	0.70994	0.00015
15.98	0.71020	0.70991	0.00014
15.93	0.71008	0.70985	0.00014
15.89	0.70989	0.70984	0.00014
15.85	0.71017	0.70985	0.00014
15.80	0.70952	0.70985	0.00014
15.76	0.71003	0.70992	0.00014
15.71	0.70990	0.70984	0.00018
15.67	0.70989	0.70981	0.00019
15.63	0.70959	0.70978	0.00019
15.58	0.70986	0.70983	0.00020
15.54	0.70954	0.70983	0.00020
15.50	0.70999	0.70984	0.00019
15.45	0.71000	0.70982	0.00019
15.41	0.71016	0.70980	0.00019
15.37	0.71020	0.70975	0.00017
15.32	0.70931	0.70971	0.00014
15.28	0.70954	0.70975	0.00010
15.24	0.70956	0.70975	0.00010
15.19	0.71012	0.70975	0.00011
15.15	0.70991	0.70973	0.00009
15.11	0.70966	0.70970	0.00009
15.06	0.70976	0.70969	0.00009
15.02	0.70974	0.70968	0.00009
14.98	0.70973	0.70964	0.00011
14.93	0.70972	0.70961	0.00011
14.89	0.70973	0.70957	0.00012
14.85	0.70958	0.70962	0.00017
14.80	0.70950	0.70965	0.00018
14.76	0.70998	0.70965	0.00018
14.72	0.70954	0.70962	0.00017
14.67	0.70961	0.70960	0.00017
14.63	0.70963	0.70959	0.00017
14.59	0.70934	0.70957	0.00018
14.54	0.70950	0.70956	0.00018
14.50	0.70932	0.70955	0.00019
14.46	0.71021	0.70959	0.00018
14.41	0.70988	0.70954	0.00012
14.37	0.70948	0.70951	0.00010
14.33	0.70965	0.70952	0.00010

14.28	0.70938	0.70953	0.00011
14.24	0.70954	0.70953	0.00011
14.20	0.70943	0.70949	0.00014
14.15	0.70922	0.70949	0.00014
14.11	0.70940	0.70956	0.00015
14.07	0.70972	0.70961	0.00016
14.02	0.70970	0.70958	0.00017
13.98	0.70955	0.70957	0.00017
13.93	0.70956	0.70954	0.00018
13.89	0.70980	0.70955	0.00018
13.85	0.70939	0.70950	0.00017
13.80	0.70910	0.70953	0.00017
13.76	0.70946	0.70957	0.00015
13.72	0.70990	0.70956	0.00015
13.67	0.70997	0.70953	0.00013
13.63	0.70937	0.70946	0.00009
13.59	0.70957	0.70947	0.00008
13.54	0.70926	0.70947	0.00009
13.50	0.70967	0.70953	0.00010
13.46	0.70936	0.70947	0.00012
13.41	0.70960	0.70945	0.00013
13.37	0.70954	0.70946	0.00014
13.33	0.70940	0.70946	0.00013
13.28	0.70952	0.70949	0.00015
13.24	0.70931	0.70950	0.00015
13.20	0.70943	0.70954	0.00015
13.15	0.70962	0.70956	0.00015
13.11	0.70980	0.70954	0.00014
13.07	0.70910	0.70950	0.00014
13.02	0.70920	0.70953	0.00011
12.98	0.70964	0.70956	0.00008
12.94	0.70953	0.70952	0.00010
12.89	0.70975	0.70954	0.00011
12.85	0.70958	0.70953	0.00010
12.81	0.70970	0.70950	0.00011
12.76	0.70963	0.70950	0.00011
12.72	0.70952	0.70947	0.00011
12.68	0.70931	0.70946	0.00011
12.63	0.70948	0.70948	0.00011
12.59	0.70947	0.70949	0.00011
12.55	0.70923	0.70948	0.00011
12.50	0.70969	0.70953	0.00010
12.46	0.70965	0.70951	0.00010
12.42	0.70929	0.70950	0.00009
12.37	0.70971	0.70952	0.00008
12.33	0.70934	0.70948	0.00007
12.29	0.70946	0.70948	0.00007
12.24	0.70945	0.70949	0.00007
12.20	0.70959	0.70945	0.00011
12.16	0.70940	0.70941	0.00011

12.11	0.70969	0.70941	0.00011
12.02	0.70954	0.70938	0.00009
11.98	0.70952	0.70940	0.00012
11.94	0.70935	0.70934	0.00015
11.89	0.70934	0.70936	0.00016
11.85	0.70953	0.70937	0.00016
11.81	0.70909	0.70939	0.00017
11.76	0.70927	0.70943	0.00016
11.72	0.70934	0.70946	0.00015
11.68	0.70930	0.70951	0.00017
11.63	0.70949	0.70956	0.00017
11.59	0.70978	0.70952	0.00019
11.55	0.70891	0.70948	0.00019
11.50	0.70957	0.70954	0.00014
11.46	0.70945	0.70949	0.00016
11.42	0.70970	0.70947	0.00017
11.37	0.70952	0.70947	0.00017
11.33	0.70951	0.70946	0.00017
11.29	0.70988	0.70945	0.00017
11.24	0.70978	0.70944	0.00016
11.20	0.70912	0.70939	0.00015
11.16	0.70937	0.70943	0.00014
11.11	0.70950	0.70943	0.00014
11.07	0.70909	0.70942	0.00014
11.03	0.70919	0.70946	0.00012
10.98	0.70969	0.70953	0.00013
10.94	0.70948	0.70948	0.00014
10.90	0.70938	0.70949	0.00014
10.85	0.70979	0.70950	0.00014
10.81	0.70926	0.70941	0.00017
10.77	0.70951	0.70946	0.00018
10.72	0.70944	0.70947	0.00018
10.68	0.70933	0.70948	0.00018
10.64	0.70952	0.70946	0.00019
10.59	0.70987	0.70945	0.00019
10.55	0.70917	0.70941	0.00017
10.51	0.70966	0.70946	0.00017
10.46	0.70940	0.70940	0.00017
10.42	0.70892	0.70938	0.00018
10.38	0.70978	0.70944	0.00014
10.33	0.70961	0.70939	0.00012
10.29	0.70956	0.70939	0.00012
10.24	0.70915	0.70938	0.00012
10.20	0.70938	0.70942	0.00011
10.16	0.70946	0.70943	0.00011
10.11	0.70967	0.70941	0.00012
10.07	0.70908	0.70935	0.00012
10.03	0.70923	0.70933	0.00014
9.98	0.70948	0.70937	0.00014
9.94	0.70931	0.70934	0.00015

9.90	0.70959	0.70930	0.00017
9.85	0.70942	0.70927	0.00015
9.81	0.70957	0.70925	0.00015
9.77	0.70947	0.70924	0.00014
9.72	0.70924	0.70919	0.00014
9.68	0.70908	0.70920	0.00015
9.64	0.70893	0.70923	0.00015
9.59	0.70961	0.70926	0.00013
9.55	0.70919	0.70923	0.00011
9.51	0.70895	0.70925	0.00011
9.46	0.70921	0.70930	0.00009
9.42	0.70925	0.70931	0.00009
9.38	0.70950	0.70933	0.00009
9.33	0.70896	0.70932	0.00008
9.29	0.70933	0.70938	0.00005
9.25	0.70935	0.70938	0.00005
9.20	0.70929	0.70938	0.00005
9.16	0.70932	0.70938	0.00005
9.12	0.70938	0.70937	0.00006
9.07	0.70940	0.70940	0.00008
9.03	0.70933	0.70939	0.00008
8.99	0.70944	0.70938	0.00009
8.94	0.70936	0.70938	0.00009
8.90	0.70957	0.70934	0.00013
8.86	0.70932	0.70932	0.00012
8.81	0.70941	0.70933	0.00012
8.77	0.70926	0.70930	0.00013
8.73	0.70922	0.70928	0.00013
8.68	0.70966	0.70930	0.00014
8.64	0.70934	0.70920	0.00015
8.60	0.70922	0.70922	0.00016
8.55	0.70947	0.70920	0.00017
8.51	0.70892	0.70920	0.00017
8.47	0.70942	0.70930	0.00020
8.42	0.70935	0.70930	0.00020
8.38	0.70912	0.70931	0.00020
8.33	0.70907	0.70935	0.00020
8.29	0.70940	0.70937	0.00019
8.25	0.70873	0.70934	0.00020
8.20	0.70950	0.70941	0.00015
8.16	0.70905	0.70941	0.00015
8.12	0.70947	0.70944	0.00013
8.07	0.70985	0.70943	0.00013
8.03	0.70948	0.70938	0.00009
7.99	0.70944	0.70938	0.00009
7.94	0.70955	0.70938	0.00009
7.90	0.70921	0.70936	0.00009
7.86	0.70907	0.70936	0.00008
7.81	0.70953	0.70942	0.00007
7.77	0.70940	0.70940	0.00007

7.73	0.70944	0.70940	0.00007
7.68	0.70937	0.70944	0.00011
7.64	0.70929	0.70941	0.00014
7.60	0.70949	0.70943	0.00014
7.55	0.70943	0.70939	0.00016
7.51	0.70934	0.70941	0.00016
7.47	0.70927	0.70941	0.00016
7.42	0.70962	0.70946	0.00018
7.38	0.70929	0.70943	0.00018
7.34	0.70949	0.70945	0.00017
7.29	0.70982	0.70947	0.00018
7.25	0.70902	0.70946	0.00017
7.21	0.70956	0.70953	0.00015
7.16	0.70908	0.70954	0.00015
7.12	0.70961	0.70958	0.00011
7.08	0.70930	0.70961	0.00013
7.03	0.70981	0.70960	0.00014
6.99	0.70929	0.70959	0.00014
6.95	0.70952	0.70962	0.00013
6.90	0.70969	0.70965	0.00014
6.86	0.70969	0.70960	0.00017
6.82	0.70975	0.70958	0.00017
6.77	0.70967	0.70957	0.00016
6.73	0.70950	0.70959	0.00017
6.69	0.70991	0.70960	0.00017
6.64	0.70918	0.70953	0.00017
6.60	0.70974	0.70956	0.00015
6.56	0.70950	0.70953	0.00015
6.51	0.70990	0.70956	0.00016
6.47	0.70915	0.70953	0.00014
6.42	0.70953	0.70955	0.00012
6.38	0.70956	0.70954	0.00012
6.34	0.70988	0.70951	0.00014
6.29	0.70959	0.70944	0.00012
6.25	0.70926	0.70943	0.00011
6.21	0.70946	0.70943	0.00012
6.16	0.70943	0.70943	0.00012
6.12	0.70982	0.70943	0.00012
6.08	0.70958	0.70940	0.00008
6.03	0.70939	0.70938	0.00007
5.99	0.70941	0.70940	0.00009
5.95	0.70924	0.70942	0.00009
5.90	0.70923	0.70947	0.00010
5.86	0.70950	0.70952	0.00010
5.82	0.70920	0.70949	0.00012
5.77	0.70948	0.70955	0.00012
5.73	0.70948	0.70953	0.00013
5.69	0.70945	0.70951	0.00014
5.64	0.70942	0.70954	0.00015
5.60	0.70961	0.70957	0.00015

5.56	0.70957	0.70958	0.00015
5.51	0.70974	0.70952	0.00019
5.47	0.70977	0.70951	0.00018
5.43	0.70920	0.70950	0.00018
5.38	0.70981	0.70953	0.00016
5.34	0.70922	0.70952	0.00016
5.30	0.70930	0.70950	0.00017
5.25	0.70978	0.70953	0.00017
5.21	0.70967	0.70952	0.00016
5.17	0.70970	0.70954	0.00017
5.12	0.70904	0.70951	0.00017
5.08	0.70957	0.70954	0.00014
5.04	0.70969	0.70950	0.00016
4.99	0.70952	0.70948	0.00015
4.95	0.70967	0.70949	0.00016
4.91	0.70906	0.70948	0.00015
4.86	0.70963	0.70952	0.00012
4.82	0.70963	0.70957	0.00017
4.78	0.70985	0.70959	0.00018
4.73	0.70943	0.70956	0.00017
4.69	0.70938	0.70959	0.00017
4.64	0.70912	0.70962	0.00016
4.60	0.70953	0.70968	0.00012
4.56	0.70957	0.70963	0.00017
4.51	0.70960	0.70963	0.00017
4.47	0.70945	0.70962	0.00017
4.43	0.71010	0.70961	0.00017
4.38	0.70987	0.70955	0.00014
4.34	0.70957	0.70958	0.00019
4.30	0.70972	0.70960	0.00019
4.25	0.70967	0.70958	0.00019
4.21	0.70969	0.70952	0.00021
4.17	0.70907	0.70953	0.00021
4.12	0.70955	0.70959	0.00019
4.08	0.70948	0.70959	0.00019
4.04	0.70944	0.70961	0.00019
3.99	0.70941	0.70961	0.00019
3.95	0.71023	0.70963	0.00018
3.91	0.70979	0.70957	0.00012
3.86	0.70947	0.70953	0.00011
3.82	0.70910	0.70955	0.00012
3.78	0.70972	0.70961	0.00006
3.73	0.70968	0.70959	0.00006
3.69	0.70954	0.70954	0.00009
3.65	0.70966	0.70953	0.00010
3.60	0.70945	0.70948	0.00012
3.56	0.70965	0.70949	0.00012
3.52	0.70959	0.70944	0.00013
3.47	0.70944	0.70943	0.00014
3.43	0.70969	0.70946	0.00015

3.39	0.70965	0.70947	0.00016
3.34	0.70952	0.70949	0.00018
3.30	0.70920	0.70946	0.00018
3.26	0.70949	0.70956	0.00021
3.21	0.70912	0.70960	0.00022
3.17	0.70956	0.70964	0.00019
3.13	0.70918	0.70964	0.00019
3.04	0.70976	0.70966	0.00019
3.00	0.70974	0.70963	0.00019
2.95	0.70988	0.70960	0.00020
2.91	0.70926	0.70959	0.00019
2.87	0.71009	0.70964	0.00018
2.82	0.70981	0.70959	0.00015
2.78	0.70952	0.70958	0.00014
2.73	0.70955	0.70956	0.00015
2.69	0.70989	0.70955	0.00016
2.65	0.70908	0.70952	0.00014
2.60	0.70954	0.70958	0.00010
2.56	0.70943	0.70959	0.00010
2.52	0.70970	0.70961	0.00009
2.47	0.70983	0.70959	0.00009
2.43	0.70960	0.70957	0.00008
2.39	0.70968	0.70959	0.00009
2.34	0.70933	0.70958	0.00009
2.30	0.70939	0.70962	0.00008
2.26	0.70965	0.70958	0.00014
2.21	0.70969	0.70958	0.00014
2.17	0.70962	0.70958	0.00014
2.13	0.70963	0.70959	0.00015
2.08	0.70950	0.70964	0.00018
2.04	0.70958	0.70966	0.00018
2.00	0.70979	0.70964	0.00018
1.95	0.70960	0.70963	0.00018
1.91	0.70979	0.70961	0.00019
1.87	0.70900	0.70958	0.00019
1.82	0.70962	0.70960	0.00016
1.78	0.70965	0.70961	0.00016
1.74	0.70980	0.70961	0.00016
1.69	0.71011	0.70959	0.00016
1.65	0.70963	0.70951	0.00011
1.61	0.70945	0.70952	0.00012
1.56	0.70971	0.70953	0.00012
1.52	0.70932	0.70949	0.00011
1.48	0.70958	0.70947	0.00013
1.43	0.70920	0.70950	0.00015
1.39	0.70964	0.70954	0.00013
1.35	0.70969	0.70957	0.00016
1.30	0.70958	0.70952	0.00017
1.26	0.70933	0.70953	0.00017
1.22	0.70971	0.70954	0.00017

1.17	0.70951	0.70952	0.00016
1.13	0.70934	0.70949	0.00017
1.09	0.70916	0.70947	0.00019
1.04	0.70987	0.70949	0.00018
1.00	0.70957	0.70942	0.00016
0.96	0.70995	0.70939	0.00016
0.91	0.70917	0.70936	0.00012
0.87	0.70965	0.70941	0.00012
0.82	0.70947	0.70941	0.00013
0.78	0.70948	0.70945	0.00015
0.74	0.70926	0.70942	0.00016
0.69	0.70909	0.70947	0.00016
0.65	0.70935	0.70955	0.00016
0.61	0.70923	0.70959	0.00016
0.56	0.70929	0.70965	0.00014
0.52	0.70963	0.70965	0.00014
0.48	0.70964	0.70964	0.00014
0.43	0.70967	0.70960	0.00017
0.39	0.70984	0.70959	0.00018
0.35	0.70924	0.70956	0.00018
0.30	0.70973	0.70961	0.00017
0.26	0.70991	0.70959	0.00019
0.22	0.70976	0.70952	0.00017
0.17	0.70983	0.70946	0.00018
0.13	0.70929	0.70934	0.00010
0.09	0.70953	0.70937	0.00014
0.04	0.70921		

ARB 33.4.1 (M1)

Distance from cervix (mm)	$^{87}Sr/^{86}Sr$	10 point mov. average	2 SE on mov. average
17.70	0.71181	0.71192	0.00016
17.66	0.71203	0.71191	0.00016
17.61	0.71201	0.71187	0.00017
17.57	0.71221	0.71187	0.00017
17.52	0.71234	0.71183	0.00015
17.48	0.71181	0.71178	0.00010
17.44	0.71152	0.71178	0.00010
17.39	0.71156	0.71181	0.00009
17.35	0.71195	0.71182	0.00007
17.30	0.71192	0.71182	0.00007
17.26	0.71178	0.71184	0.00009
17.22	0.71161	0.71182	0.00011
17.17	0.71201	0.71191	0.00015
17.13	0.71177	0.71190	0.00015
17.08	0.71185	0.71193	0.00015
17.04	0.71179	0.71193	0.00015
17.00	0.71185	0.71196	0.00015
16.95	0.71173	0.71195	0.00016
16.91	0.71195	0.71199	0.00015
16.86	0.71211	0.71198	0.00016
16.82	0.71156	0.71199	0.00016
16.78	0.71244	0.71203	0.00014
16.73	0.71192	0.71199	0.00010
16.69	0.71210	0.71198	0.00011
16.64	0.71186	0.71196	0.00010
16.60	0.71204	0.71199	0.00011
16.55	0.71175	0.71199	0.00011
16.51	0.71216	0.71205	0.00011
16.47	0.71183	0.71206	0.00012
16.42	0.71226	0.71207	0.00012
16.38	0.71190	0.71203	0.00011
16.33	0.71205	0.71205	0.00011
16.29	0.71184	0.71205	0.00011
16.25	0.71187	0.71208	0.00010
16.20	0.71221	0.71214	0.00012
16.16	0.71207	0.71215	0.00012
16.11	0.71230	0.71217	0.00012
16.07	0.71228	0.71216	0.00012
16.03	0.71188	0.71218	0.00013
15.98	0.71194	0.71225	0.00014
15.94	0.71203	0.71227	0.00012
15.89	0.71203	0.71228	0.00012
15.85	0.71221	0.71232	0.00012
15.81	0.71246	0.71231	0.00012
15.76	0.71224	0.71228	0.00012
15.72	0.71233	0.71226	0.00013
15.67	0.71219	0.71221	0.00016

ARB 33.2.1 (M2)

Distance from cervix (mm)	$^{87}Sr/^{86}Sr$	10 point mov. average	2 SE on mov. average
26.89	0.71022	0.70997	0.00027
26.85	0.71021	0.70985	0.00034
26.80	0.70995	0.70990	0.00037
26.76	0.70948	0.70986	0.00038
26.72	0.71075	0.71002	0.00044
26.67	0.70975	0.70994	0.00041
26.63	0.70960	0.70994	0.00041
26.58	0.71037	0.70996	0.00040
26.54	0.70936	0.70997	0.00040
26.50	0.71006	0.71007	0.00039
26.45	0.70894	0.71008	0.00039
26.41	0.71070	0.71014	0.00032
26.37	0.70961	0.71001	0.00032
26.32	0.71110	0.71003	0.00031
26.28	0.70989	0.70997	0.00023
26.24	0.70979	0.70993	0.00025
26.19	0.70978	0.71004	0.00031
26.15	0.71042	0.71011	0.00031
26.11	0.71038	0.71003	0.00032
26.06	0.71016	0.70995	0.00032
26.02	0.70959	0.70992	0.00032
25.97	0.70939	0.70997	0.00031
25.93	0.70984	0.70992	0.00037
25.89	0.71046	0.70987	0.00039
25.84	0.70952	0.70973	0.00039
25.80	0.71086	0.70976	0.00039
25.76	0.71049	0.70973	0.00035
25.71	0.70961	0.70971	0.00034
25.67	0.70960	0.70969	0.00034
25.63	0.70985	0.70984	0.00043
25.58	0.71013	0.71000	0.00054
25.54	0.70886	0.71000	0.00054
25.50	0.70930	0.71009	0.00048
25.45	0.70908	0.71012	0.00046
25.41	0.70987	0.71033	0.00044
25.36	0.71049	0.71042	0.00043
25.32	0.71034	0.71038	0.00044
25.28	0.70945	0.71040	0.00044
25.23	0.71107	0.71045	0.00040
25.19	0.71142	0.71042	0.00039
25.15	0.71012	0.71029	0.00032
25.10	0.70974	0.71029	0.00032
25.06	0.70966	0.71027	0.00034
25.02	0.71115	0.71034	0.00031
24.97	0.71075	0.71024	0.00026
24.93	0.71007	0.71016	0.00023
24.89	0.71060	0.71015	0.00024

15.63	0.71245	0.71220	0.00016
15.59	0.71263	0.71222	0.00017
15.54	0.71214	0.71218	0.00014
15.50	0.71206	0.71217	0.00014
15.45	0.71250	0.71219	0.00014
15.41	0.71208	0.71212	0.00014
15.37	0.71215	0.71212	0.00014
15.32	0.71209	0.71212	0.00014
15.28	0.71180	0.71215	0.00015
15.23	0.71215	0.71220	0.00013
15.19	0.71256	0.71223	0.00014
15.15	0.71229	0.71219	0.00012
15.10	0.71202	0.71219	0.00012
15.06	0.71224	0.71223	0.00012
15.01	0.71183	0.71222	0.00013
14.97	0.71205	0.71230	0.00012
14.93	0.71213	0.71230	0.00011
14.88	0.71245	0.71233	0.00011
14.84	0.71228	0.71229	0.00011
14.79	0.71243	0.71229	0.00011
14.75	0.71217	0.71229	0.00011
14.71	0.71227	0.71235	0.00014
14.66	0.71246	0.71236	0.00014
14.62	0.71214	0.71232	0.00014
14.57	0.71262	0.71234	0.00014
14.53	0.71207	0.71229	0.00013
14.49	0.71239	0.71235	0.00014
14.44	0.71211	0.71234	0.00014
14.40	0.71229	0.71235	0.00014
14.35	0.71241	0.71234	0.00014
14.31	0.71272	0.71233	0.00014
14.27	0.71238	0.71229	0.00011
14.22	0.71210	0.71228	0.00011
14.18	0.71236	0.71232	0.00010
14.13	0.71204	0.71229	0.00011
14.09	0.71267	0.71237	0.00013
14.05	0.71227	0.71229	0.00015
14.00	0.71229	0.71227	0.00015
13.96	0.71219	0.71227	0.00015
13.91	0.71231	0.71229	0.00015
13.87	0.71228	0.71228	0.00015
13.83	0.71234	0.71227	0.00016
13.78	0.71243	0.71226	0.00016
13.74	0.71210	0.71229	0.00018
13.69	0.71279	0.71233	0.00018
13.65	0.71187	0.71229	0.00015
13.61	0.71211	0.71232	0.00012
13.56	0.71231	0.71228	0.00016
13.52	0.71240	0.71229	0.00017
13.47	0.71220	0.71230	0.00017

24.84	0.70989	0.71012	0.00022
24.80	0.71078	0.71019	0.00023
24.75	0.71014	0.71009	0.00020
24.71	0.71013	0.71006	0.00020
24.67	0.70947	0.71005	0.00020
24.62	0.71038	0.71008	0.00017
24.58	0.71018	0.71013	0.00023
24.54	0.70998	0.71013	0.00023
24.49	0.70994	0.71021	0.00026
24.45	0.71026	0.71012	0.00035
24.41	0.71060	0.71010	0.00035
24.36	0.70978	0.71002	0.00034
24.32	0.70984	0.71000	0.00034
24.28	0.71002	0.71003	0.00034
24.23	0.70981	0.71005	0.00034
24.19	0.71087	0.71008	0.00034
24.14	0.71022	0.71003	0.00030
24.10	0.71080	0.71002	0.00030
24.06	0.70903	0.70994	0.00025
24.01	0.71001	0.71002	0.00015
23.97	0.70977	0.71007	0.00019
23.93	0.70962	0.71022	0.00029
23.88	0.71013	0.71036	0.00030
23.84	0.71019	0.71036	0.00030
23.80	0.71013	0.71030	0.00034
23.75	0.71042	0.71032	0.00034
23.71	0.71009	0.71030	0.00034
23.66	0.71004	0.71029	0.00034
23.62	0.70975	0.71030	0.00034
23.58	0.71056	0.71034	0.00032
23.53	0.71127	0.71033	0.00032
23.49	0.71104	0.71030	0.00028
23.45	0.71011	0.71028	0.00026
23.40	0.70957	0.71030	0.00026
23.36	0.71040	0.71032	0.00024
23.32	0.71021	0.71031	0.00024
23.27	0.70998	0.71036	0.00025
23.23	0.71007	0.71046	0.00026
23.19	0.71016	0.71049	0.00025
23.14	0.71052	0.71052	0.00024
23.10	0.71095	0.71046	0.00027
23.05	0.71085	0.71037	0.00026
23.01	0.71030	0.71033	0.00024
22.97	0.70976	0.71033	0.00024
22.92	0.71030	0.71044	0.00023
22.88	0.71071	0.71041	0.00024
22.84	0.71100	0.71044	0.00026
22.79	0.71036	0.71041	0.00023
22.75	0.71049	0.71038	0.00024
22.71	0.70989	0.71033	0.00025

13.43	0.71216	0.71230	0.00017	22.66	0.71000	0.71037	0.00023
13.38	0.71223	0.71232	0.00017	22.62	0.71054	0.71045	0.00023
13.34	0.71271	0.71233	0.00017	22.58	0.71023	0.71041	0.00023
13.30	0.71256	0.71232	0.00015	22.53	0.71086	0.71050	0.00027
13.25	0.71234	0.71230	0.00015	22.49	0.71008	0.71047	0.00026
13.21	0.71213	0.71232	0.00015	22.44	0.71099	0.71048	0.00026
13.16	0.71175	0.71230	0.00017	22.40	0.71066	0.71049	0.00027
13.12	0.71244	0.71237	0.00012	22.36	0.71006	0.71047	0.00027
13.08	0.71249	0.71237	0.00012	22.31	0.71003	0.71047	0.00027
13.03	0.71222	0.71237	0.00012	22.27	0.71028	0.71049	0.00026
12.99	0.71236	0.71238	0.00011	22.23	0.71074	0.71042	0.00032
12.94	0.71233	0.71242	0.00013	22.18	0.71014	0.71045	0.00033
12.90	0.71254	0.71242	0.00013	22.14	0.71119	0.71044	0.00034
12.86	0.71243	0.71242	0.00014	22.10	0.71056	0.71036	0.00029
12.81	0.71250	0.71244	0.00014	22.05	0.71014	0.71030	0.00030
12.77	0.71191	0.71244	0.00014	22.01	0.71112	0.71024	0.00034
12.72	0.71244	0.71250	0.00008	21.97	0.71042	0.71017	0.00028
12.68	0.71252	0.71246	0.00013	21.92	0.71007	0.71019	0.00029
12.64	0.71247	0.71244	0.00013	21.88	0.71027	0.71019	0.00029
12.59	0.71233	0.71244	0.00013	21.83	0.70955	0.71013	0.00031
12.55	0.71273	0.71247	0.00013	21.79	0.71100	0.71019	0.00028
12.50	0.71230	0.71246	0.00012	21.75	0.71006	0.71014	0.00023
12.46	0.71256	0.71249	0.00012	21.70	0.71038	0.71020	0.00025
12.42	0.71261	0.71246	0.00013	21.66	0.71000	0.71022	0.00026
12.37	0.71251	0.71246	0.00013	21.62	0.70951	0.71022	0.00026
12.33	0.71258	0.71245	0.00013	21.57	0.71043	0.71032	0.00021
12.28	0.71202	0.71246	0.00014	21.53	0.71063	0.71031	0.00021
12.24	0.71231	0.71249	0.00010	21.49	0.71009	0.71027	0.00020
12.20	0.71245	0.71255	0.00011	21.44	0.70963	0.71029	0.00019
12.15	0.71263	0.71251	0.00015	21.40	0.71019	0.71035	0.00013
12.11	0.71264	0.71247	0.00015	21.36	0.71045	0.71033	0.00015
12.06	0.71263	0.71246	0.00015	21.31	0.71065	0.71032	0.00015
12.02	0.71225	0.71244	0.00014	21.27	0.71064	0.71024	0.00015
11.98	0.71261	0.71245	0.00014	21.22	0.71002	0.71018	0.00013
11.93	0.71236	0.71241	0.00014	21.18	0.71049	0.71024	0.00015
11.89	0.71269	0.71241	0.00014	21.14	0.71033	0.71017	0.00015
11.84	0.71237	0.71237	0.00013	21.09	0.71020	0.71018	0.00016
11.80	0.71283	0.71239	0.00013	21.05	0.71033	0.71028	0.00025
11.76	0.71209	0.71237	0.00010	21.01	0.71025	0.71024	0.00026
11.71	0.71227	0.71244	0.00012	20.96	0.70991	0.71030	0.00028
11.67	0.71253	0.71245	0.00011	20.92	0.71037	0.71027	0.00030
11.62	0.71243	0.71245	0.00011	20.88	0.70990	0.71036	0.00035
11.58	0.71228	0.71245	0.00011	20.83	0.71000	0.71039	0.00034
11.54	0.71221	0.71249	0.00011	20.79	0.71058	0.71035	0.00036
11.49	0.71237	0.71251	0.00009	20.74	0.70985	0.71032	0.00036
11.45	0.71235	0.71255	0.00009	20.70	0.71044	0.71031	0.00036
11.40	0.71253	0.71256	0.00009	20.66	0.71115	0.71021	0.00040
11.36	0.71261	0.71254	0.00009	20.61	0.70997	0.71012	0.00034
11.32	0.71282	0.71252	0.00009	20.57	0.71079	0.71005	0.00039
11.27	0.71240	0.71250	0.00007	20.53	0.70969	0.71006	0.00040

11.23	0.71246	0.71248	0.00009	20.48	0.71119	0.71010	0.00039
11.18	0.71251	0.71248	0.00009	20.44	0.71020	0.70995	0.00031
11.14	0.71260	0.71248	0.00009	20.40	0.70967	0.70996	0.00031
11.10	0.71245	0.71246	0.00008	20.35	0.71028	0.71002	0.00031
11.05	0.71273	0.71242	0.00011	20.31	0.70977	0.71004	0.00033
11.01	0.71245	0.71241	0.00009	20.27	0.70943	0.71004	0.00033
10.96	0.71239	0.71240	0.00009	20.22	0.71025	0.71010	0.00030
10.92	0.71243	0.71244	0.00012	20.18	0.70921	0.71011	0.00031
10.88	0.71256	0.71241	0.00013	20.13	0.71092	0.71023	0.00023
10.83	0.71222	0.71240	0.00013	20.09	0.71011	0.71015	0.00017
10.79	0.71249	0.71238	0.00014	20.05	0.70971	0.71010	0.00021
10.74	0.71247	0.71235	0.00014	20.00	0.71022	0.71016	0.00020
10.70	0.71238	0.71236	0.00014	19.96	0.71030	0.71016	0.00020
10.66	0.71211	0.71237	0.00015	19.92	0.71053	0.71014	0.00020
10.61	0.71258	0.71244	0.00015	19.87	0.70976	0.71012	0.00018
10.57	0.71240	0.71242	0.00015	19.83	0.70996	0.71012	0.00018
10.52	0.71275	0.71243	0.00015	19.79	0.71044	0.71010	0.00019
10.48	0.71213	0.71240	0.00013	19.74	0.71032	0.71011	0.00020
10.43	0.71247	0.71243	0.00012	19.70	0.71020	0.71016	0.00023
10.39	0.71206	0.71243	0.00012	19.66	0.70954	0.71013	0.00024
10.35	0.71220	0.71250	0.00011	19.61	0.71032	0.71019	0.00020
10.30	0.71248	0.71249	0.00013	19.57	0.71024	0.71024	0.00023
10.26	0.71256	0.71246	0.00014	19.52	0.71008	0.71029	0.00026
10.21	0.71274	0.71245	0.00014	19.48	0.71029	0.71033	0.00026
10.17	0.71238	0.71244	0.00013	19.44	0.70977	0.71031	0.00026
10.13	0.71249	0.71243	0.00013	19.39	0.70982	0.71038	0.00023
10.08	0.71246	0.71241	0.00013	19.35	0.71054	0.71040	0.00021
10.04	0.71245	0.71238	0.00014	19.31	0.71075	0.71038	0.00021
9.99	0.71247	0.71236	0.00014	19.26	0.70989	0.71043	0.00026
9.95	0.71281	0.71234	0.00014	19.22	0.71017	0.71044	0.00026
9.91	0.71205	0.71229	0.00009	19.18	0.71079	0.71036	0.00033
9.86	0.71224	0.71232	0.00007	19.13	0.71081	0.71035	0.00032
9.82	0.71246	0.71235	0.00008	19.09	0.71045	0.71028	0.00031
9.77	0.71257	0.71232	0.00009	19.05	0.71005	0.71031	0.00032
9.73	0.71227	0.71230	0.00007	19.00	0.71047	0.71035	0.00032
9.69	0.71229	0.71227	0.00009	18.96	0.71010	0.71029	0.00032
9.64	0.71220	0.71225	0.00010	18.91	0.71034	0.71033	0.00032
9.60	0.71220	0.71225	0.00010	18.87	0.71122	0.71033	0.00032
9.55	0.71234	0.71226	0.00010	18.83	0.70994	0.71029	0.00028
9.51	0.71227	0.71226	0.00010	18.78	0.70940	0.71033	0.00027
9.47	0.71232	0.71225	0.00010	18.74	0.71073	0.71038	0.00020
9.42	0.71253	0.71221	0.00012	18.70	0.71013	0.71039	0.00021
9.38	0.71217	0.71220	0.00011	18.65	0.71071	0.71036	0.00023
9.33	0.71239	0.71219	0.00011	18.61	0.71045	0.71028	0.00023
9.29	0.71202	0.71217	0.00011	18.57	0.70992	0.71015	0.00032
9.25	0.71206	0.71217	0.00010	18.52	0.71050	0.71015	0.00032
9.20	0.71220	0.71220	0.00011	18.48	0.71030	0.71011	0.00031
9.16	0.71224	0.71218	0.00012	18.44	0.71081	0.71011	0.00031
9.11	0.71236	0.71216	0.00012	18.39	0.71033	0.71001	0.00027
9.07	0.71225	0.71208	0.00016	18.35	0.70996	0.70997	0.00026

9.03	0.71188	0.71207	0.00016	18.30	0.71081	0.71001	0.00028
8.98	0.71244	0.71207	0.00017	18.26	0.70981	0.70990	0.00023
8.94	0.71205	0.71202	0.00014	18.22	0.70991	0.70992	0.00023
8.89	0.71216	0.71206	0.00016	18.17	0.70917	0.71001	0.00029
8.85	0.71206	0.71200	0.00018	18.13	0.70986	0.71011	0.00022
8.81	0.71240	0.71201	0.00018	18.09	0.71012	0.71020	0.00024
8.76	0.71199	0.71197	0.00016	18.04	0.71034	0.71016	0.00026
8.72	0.71199	0.71198	0.00016	18.00	0.70980	0.71012	0.00026
8.67	0.71155	0.71197	0.00016	17.96	0.70987	0.71016	0.00025
8.63	0.71219	0.71199	0.00014	17.91	0.71045	0.71023	0.00026
8.59	0.71183	0.71195	0.00014	17.87	0.70967	0.71016	0.00027
8.54	0.71201	0.71198	0.00013	17.82	0.70999	0.71024	0.00025
8.50	0.71238	0.71198	0.00014	17.78	0.71082	0.71025	0.00024
8.45	0.71164	0.71197	0.00012	17.74	0.71017	0.71019	0.00021
8.41	0.71210	0.71199	0.00011	17.69	0.71073	0.71015	0.00023
8.37	0.71208	0.71197	0.00010	17.65	0.70980	0.71010	0.00019
8.32	0.71204	0.71197	0.00010	17.61	0.70989	0.71010	0.00019
8.28	0.71192	0.71191	0.00015	17.56	0.71019	0.71017	0.00020
8.23	0.71174	0.71189	0.00016	17.52	0.71061	0.71014	0.00021
8.19	0.71180	0.71188	0.00016	17.48	0.70975	0.71001	0.00024
8.15	0.71206	0.71188	0.00016	17.43	0.71041	0.71007	0.00024
8.10	0.71206	0.71187	0.00016	17.39	0.71009	0.71008	0.00025
8.06	0.71229	0.71186	0.00016	17.35	0.71030	0.71007	0.00025
8.01	0.71182	0.71184	0.00014	17.30	0.70974	0.71006	0.00025
7.97	0.71193	0.71184	0.00014	17.26	0.71028	0.71005	0.00026
7.93	0.71207	0.71185	0.00015	17.21	0.70978	0.70999	0.00026
7.88	0.71139	0.71184	0.00014	17.17	0.71052	0.70998	0.00027
7.84	0.71173	0.71190	0.00010	17.13	0.70993	0.70997	0.00026
7.79	0.71163	0.71187	0.00013	17.08	0.70929	0.70993	0.00027
7.75	0.71185	0.71191	0.00012	17.04	0.71036	0.71002	0.00023
7.71	0.71196	0.71193	0.00012	17.00	0.71052	0.71004	0.00024
7.66	0.71192	0.71192	0.00012	16.95	0.71000	0.70999	0.00021
7.62	0.71213	0.71191	0.00012	16.91	0.71022	0.71000	0.00021
7.57	0.71176	0.71191	0.00012	16.87	0.70958	0.70998	0.00021
7.53	0.71212	0.71193	0.00011	16.82	0.70971	0.71008	0.00022
7.48	0.71197	0.71193	0.00011	16.78	0.70967	0.71013	0.00021
7.44	0.71191	0.71193	0.00011	16.74	0.71040	0.71015	0.00019
7.40	0.71150	0.71195	0.00012	16.69	0.70960	0.71017	0.00020
7.35	0.71201	0.71198	0.00008	16.65	0.71015	0.71027	0.00017
7.31	0.71203	0.71194	0.00010	16.60	0.71052	0.71027	0.00017
7.26	0.71181	0.71190	0.00011	16.56	0.71007	0.71011	0.00031
7.22	0.71187	0.71192	0.00011	16.52	0.71003	0.71018	0.00034
7.18	0.71208	0.71191	0.00011	16.47	0.71007	0.71015	0.00035
7.13	0.71197	0.71188	0.00011	16.43	0.71059	0.71012	0.00035
7.09	0.71214	0.71184	0.00012	16.39	0.71018	0.71014	0.00037
7.04	0.71198	0.71183	0.00011	16.34	0.70994	0.71014	0.00037
7.00	0.71211	0.71178	0.00012	16.30	0.71060	0.71021	0.00038
6.96	0.71176	0.71176	0.00010	16.26	0.71053	0.71017	0.00037
6.91	0.71164	0.71179	0.00012	16.21	0.71016	0.71004	0.00041
6.87	0.71168	0.71180	0.00012	16.17	0.70889	0.71001	0.00041

6.82	0.71196	0.71178	0.00013
6.78	0.71178	0.71174	0.00013
6.74	0.71179	0.71175	0.00014
6.69	0.71157	0.71173	0.00014
6.65	0.71197	0.71175	0.00014
6.60	0.71151	0.71172	0.00013
6.56	0.71194	0.71174	0.00012
6.52	0.71207	0.71175	0.00014
6.47	0.71174	0.71172	0.00012
6.43	0.71148	0.71166	0.00017
6.38	0.71153	0.71171	0.00017
6.34	0.71190	0.71175	0.00017
6.30	0.71155	0.71173	0.00017
6.25	0.71186	0.71176	0.00016
6.21	0.71162	0.71173	0.00016
6.16	0.71173	0.71174	0.00016
6.12	0.71207	0.71176	0.00016
6.08	0.71175	0.71175	0.00016
6.03	0.71113	0.71173	0.00016
5.99	0.71199	0.71177	0.00010
5.94	0.71187	0.71179	0.00013
5.90	0.71172	0.71180	0.00013
5.86	0.71184	0.71183	0.00013
5.81	0.71162	0.71185	0.00014
5.77	0.71172	0.71189	0.00014
5.72	0.71190	0.71191	0.00013
5.68	0.71197	0.71192	0.00013
5.64	0.71158	0.71192	0.00013
5.59	0.71153	0.71195	0.00011
5.55	0.71219	0.71198	0.00007
5.50	0.71190	0.71196	0.00005
5.46	0.71202	0.71194	0.00008
5.42	0.71206	0.71192	0.00008
5.37	0.71204	0.71190	0.00007
5.33	0.71190	0.71186	0.00008
5.28	0.71204	0.71186	0.00008
5.24	0.71195	0.71181	0.00009
5.20	0.71192	0.71179	0.00008
5.15	0.71183	0.71179	0.00008
5.11	0.71195	0.71175	0.00010
5.06	0.71166	0.71176	0.00011
5.02	0.71182	0.71175	0.00011
4.98	0.71193	0.71170	0.00013
4.93	0.71163	0.71169	0.00012
4.89	0.71184	0.71172	0.00013
4.84	0.71157	0.71166	0.00016
4.80	0.71179	0.71169	0.00016
4.76	0.71188	0.71169	0.00016
4.71	0.71147	0.71166	0.00016
4.67	0.71198	0.71167	0.00015

16.13	0.71076	0.71019	0.00034
16.08	0.70974	0.71018	0.00033
16.04	0.70982	0.71021	0.00032
15.99	0.71076	0.71019	0.00033
15.95	0.71017	0.71009	0.00031
15.91	0.71065	0.71006	0.00032
15.86	0.71024	0.71007	0.00033
15.82	0.70917	0.71008	0.00033
15.78	0.70990	0.71019	0.00026
15.73	0.71071	0.71018	0.00028
15.69	0.71061	0.71015	0.00026
15.65	0.71006	0.71009	0.00024
15.60	0.70960	0.71011	0.00024
15.56	0.70980	0.71020	0.00022
15.52	0.70980	0.71021	0.00022
15.47	0.71075	0.71027	0.00020
15.43	0.71043	0.71022	0.00017
15.38	0.71026	0.71021	0.00016
15.34	0.70973	0.71027	0.00021
15.30	0.71048	0.71029	0.00019
15.25	0.71001	0.71025	0.00018
15.21	0.71023	0.71034	0.00021
15.17	0.71052	0.71030	0.00024
15.12	0.70990	0.71023	0.00025
15.08	0.71044	0.71022	0.00025
15.04	0.71021	0.71015	0.00027
14.99	0.71031	0.71019	0.00029
14.95	0.71083	0.71020	0.00029
14.90	0.70994	0.71011	0.00026
14.86	0.71012	0.71007	0.00028
14.82	0.71090	0.71008	0.00028
14.77	0.70984	0.71003	0.00023
14.73	0.70984	0.71000	0.00025
14.69	0.70982	0.71000	0.00025
14.64	0.70967	0.71006	0.00026
14.60	0.71068	0.71017	0.00028
14.56	0.71039	0.71018	0.00028
14.51	0.70990	0.71020	0.00029
14.47	0.70956	0.71025	0.00029
14.43	0.71022	0.71042	0.00030
14.38	0.71041	0.71042	0.00030
14.34	0.70950	0.71036	0.00032
14.29	0.70991	0.71043	0.00026
14.25	0.71042	0.71047	0.00024
14.21	0.71076	0.71047	0.00024
14.16	0.71071	0.71036	0.00028
14.12	0.71061	0.71028	0.00028
14.08	0.71042	0.71017	0.00031
14.03	0.71120	0.71011	0.00031
13.99	0.71026	0.71001	0.00020

4.62	0.71162	0.71166	0.00014
4.58	0.71135	0.71162	0.00017
4.54	0.71175	0.71164	0.00016
4.49	0.71198	0.71163	0.00016
4.45	0.71125	0.71161	0.00014
4.40	0.71180	0.71164	0.00012
4.36	0.71181	0.71162	0.00011
4.31	0.71160	0.71161	0.00011
4.27	0.71159	0.71162	0.00011
4.23	0.71185	0.71163	0.00011
4.18	0.71120	0.71161	0.00010
4.14	0.71158	0.71164	0.00006
4.09	0.71169	0.71164	0.00005
4.05	0.71170	0.71166	0.00007
4.01	0.71154	0.71165	0.00008
3.96	0.71169	0.71164	0.00008
3.92	0.71165	0.71162	0.00008
3.87	0.71172	0.71164	0.00009
3.83	0.71172	0.71162	0.00009
3.79	0.71161	0.71163	0.00010
3.74	0.71146	0.71161	0.00011
3.70	0.71163	0.71163	0.00010
3.65	0.71190	0.71162	0.00010
3.61	0.71154	0.71159	0.00008
3.57	0.71152	0.71164	0.00012
3.52	0.71150	0.71165	0.00012
3.48	0.71180	0.71167	0.00011
3.43	0.71155	0.71168	0.00012
3.39	0.71183	0.71169	0.00011
3.35	0.71141	0.71164	0.00013
3.30	0.71158	0.71163	0.00014
3.26	0.71158	0.71166	0.00014
3.21	0.71162	0.71168	0.00015
3.17	0.71203	0.71171	0.00016
3.13	0.71164	0.71168	0.00014
3.08	0.71164	0.71169	0.00014
3.04	0.71189	0.71170	0.00014
2.99	0.71163	0.71171	0.00015
2.95	0.71134	0.71176	0.00017
2.91	0.71131	0.71180	0.00014
2.86	0.71187	0.71186	0.00009
2.82	0.71178	0.71184	0.00010
2.77	0.71200	0.71182	0.00011
2.73	0.71174	0.71177	0.00012
2.69	0.71176	0.71179	0.00013
2.64	0.71170	0.71177	0.00013
2.60	0.71194	0.71178	0.00013
2.55	0.71217	0.71179	0.00014
2.51	0.71172	0.71177	0.00012
2.47	0.71189	0.71175	0.00013

13.95	0.70982	0.71000	0.00019
13.90	0.71021	0.71000	0.00019
13.86	0.71032	0.71003	0.00021
13.82	0.71043	0.71006	0.00023
13.77	0.70963	0.71003	0.00022
13.73	0.70987	0.71014	0.00024
13.68	0.70949	0.71019	0.00024
13.64	0.70990	0.71029	0.00018
13.60	0.71020	0.71033	0.00016
13.55	0.71008	0.71035	0.00016
13.51	0.70990	0.71038	0.00015
13.47	0.71048	0.71041	0.00011
13.42	0.71060	0.71032	0.00021
13.38	0.71014	0.71031	0.00020
13.34	0.71074	0.71036	0.00021
13.29	0.71042	0.71033	0.00020
13.25	0.71042	0.71033	0.00020
13.21	0.71030	0.71034	0.00020
13.16	0.71038	0.71036	0.00021
13.12	0.71042	0.71038	0.00021
13.07	0.71020	0.71038	0.00021
13.03	0.70952	0.71031	0.00027
12.99	0.71052	0.71038	0.00021
12.94	0.71071	0.71038	0.00021
12.90	0.71038	0.71037	0.00021
12.86	0.71042	0.71040	0.00022
12.81	0.71057	0.71038	0.00022
12.77	0.71051	0.71039	0.00022
12.73	0.71058	0.71034	0.00024
12.68	0.71039	0.71037	0.00026
12.64	0.70949	0.71034	0.00027
12.60	0.71026	0.71043	0.00019
12.55	0.71048	0.71041	0.00021
12.51	0.71060	0.71041	0.00021
12.46	0.71073	0.71041	0.00021
12.42	0.71021	0.71041	0.00021
12.38	0.71064	0.71040	0.00022
12.33	0.70998	0.71032	0.00024
12.29	0.71094	0.71036	0.00023
12.25	0.71005	0.71035	0.00022
12.20	0.71047	0.71041	0.00021
12.16	0.71003	0.71039	0.00021
12.12	0.71043	0.71041	0.00020
12.07	0.71068	0.71032	0.00026
12.03	0.71073	0.71028	0.00025
11.99	0.71003	0.71019	0.00024
11.94	0.70986	0.71020	0.00024
11.90	0.71039	0.71026	0.00023
11.85	0.71085	0.71023	0.00023
11.81	0.71065	0.71014	0.00019

2.42	0.71172	0.71176	0.00014
2.38	0.71154	0.71172	0.00017
2.33	0.71149	0.71173	0.00016
2.29	0.71195	0.71175	0.00015
2.25	0.71163	0.71172	0.00015
2.20	0.71171	0.71176	0.00016
2.16	0.71208	0.71180	0.00018
2.11	0.71197	0.71178	0.00017
2.07	0.71154	0.71180	0.00018
2.03	0.71198	0.71184	0.00017
1.98	0.71131	0.71185	0.00018
1.94	0.71163	0.71192	0.00013
1.89	0.71176	0.71194	0.00011
1.85	0.71158	0.71193	0.00012
1.81	0.71200	0.71199	0.00010
1.76	0.71219	0.71196	0.00012
1.72	0.71189	0.71191	0.00011
1.67	0.71213	0.71191	0.00011
1.63	0.71193	0.71187	0.00010
1.59	0.71211	0.71188	0.00011
1.54	0.71193	0.71185	0.00009
1.50	0.71191	0.71182	0.00011
1.45	0.71167	0.71183	0.00011
1.41	0.71211	0.71183	0.00011
1.36	0.71168	0.71180	0.00009
1.32	0.71170	0.71181	0.00009
1.28	0.71190	0.71181	0.00009
1.23	0.71175	0.71182	0.00010
1.19	0.71202	0.71183	0.00010
1.14	0.71183	0.71187	0.00014
1.10	0.71159	0.71189	0.00015
1.06	0.71200	0.71192	0.00013
1.01	0.71170	0.71191	0.00013
0.97	0.71178	0.71189	0.00014
0.92	0.71183	0.71192	0.00015
0.88	0.71167	0.71193	0.00015
0.84	0.71206	0.71197	0.00014
0.79	0.71182	0.71195	0.00013
0.75	0.71236	0.71198	0.00013
0.70	0.71207	0.71196	0.00011
0.66	0.71191	0.71197	0.00012
0.62	0.71190	0.71201	0.00013
0.57	0.71154	0.71197	0.00016
0.53	0.71207	0.71202	0.00013
0.48	0.71189	0.71199	0.00014
0.44	0.71207	0.71195	0.00017
0.40	0.71192	0.71193	0.00018
0.35	0.71205	0.71193	0.00019
0.31	0.71220	0.71192	0.00020
0.26	0.71214	0.71187	0.00021

11.77	0.71025	0.71014	0.00019
11.72	0.71021	0.71013	0.00019
11.68	0.70960	0.71014	0.00019
11.64	0.71026	0.71021	0.00015
11.59	0.70978	0.71020	0.00015
11.55	0.71016	0.71023	0.00012
11.51	0.71043	0.71032	0.00021
11.46	0.71010	0.71034	0.00022
11.42	0.70999	0.71032	0.00023
11.37	0.71062	0.71036	0.00022
11.33	0.71010	0.71028	0.00024
11.29	0.71038	0.71033	0.00024
11.24	0.71027	0.71035	0.00025
11.20	0.71015	0.71037	0.00025
11.16	0.71010	0.71042	0.00025
11.11	0.71108	0.71045	0.00024
11.07	0.71064	0.71035	0.00020
11.03	0.70989	0.71031	0.00019
10.98	0.71040	0.71037	0.00016
10.94	0.70981	0.71039	0.00018
10.90	0.71062	0.71045	0.00012
10.85	0.71056	0.71040	0.00013
10.81	0.71042	0.71039	0.00013
10.76	0.71066	0.71040	0.00013
10.72	0.71043	0.71046	0.00022
10.68	0.71010	0.71039	0.00026
10.63	0.71022	0.71035	0.00029
10.59	0.71042	0.71039	0.00030
10.55	0.71068	0.71032	0.00032
10.50	0.71042	0.71027	0.00031
10.46	0.71009	0.71031	0.00033
10.42	0.71048	0.71030	0.00033
10.37	0.71045	0.71028	0.00032
10.33	0.71129	0.71023	0.00033
10.29	0.70975	0.71013	0.00023
10.24	0.70967	0.71016	0.00022
10.20	0.71059	0.71024	0.00020
10.15	0.70980	0.71022	0.00019
10.11	0.71014	0.71025	0.00017
10.07	0.71079	0.71030	0.00018
10.02	0.71007	0.71024	0.00015
9.98	0.71024	0.71023	0.00016
9.94	0.70999	0.71027	0.00018
9.89	0.71030	0.71043	0.00030
9.85	0.70996	0.71040	0.00031
9.81	0.71048	0.71043	0.00030
9.76	0.71045	0.71033	0.00035
9.72	0.71005	0.71024	0.00038
9.68	0.71066	0.71022	0.00039
9.63	0.71018	0.71019	0.00037

0.22	0.71229	0.71182	0.00021
0.18	0.71156	0.71170	0.00015
0.13	0.71202	0.71175	0.00017
0.09	0.71173	0.71161	0.00010
0.04	0.71149		

9.59	0.70994	0.71026	0.00040
9.54	0.71068	0.71024	0.00041
9.50	0.71155	0.71023	0.00040
9.46	0.71006	0.71012	0.00029
9.41	0.71020	0.71014	0.00029
9.37	0.70955	0.71016	0.00029
9.33	0.70957	0.71032	0.00032
9.28	0.70979	0.71038	0.00028
9.24	0.71035	0.71040	0.00026
9.20	0.71088	0.71038	0.00027
9.15	0.70975	0.71036	0.00025
9.11	0.71057	0.71040	0.00022
9.07	0.71050	0.71034	0.00023
9.02	0.71020	0.71036	0.00024
8.98	0.71042	0.71034	0.00025
8.93	0.71118	0.71033	0.00025
8.89	0.71012	0.71026	0.00017
8.85	0.71004	0.71030	0.00018
8.80	0.71018	0.71030	0.00018
8.76	0.71064	0.71033	0.00018
8.72	0.71021	0.71027	0.00017
8.67	0.70995	0.71022	0.00022
8.63	0.71070	0.71028	0.00022
8.59	0.70994	0.71023	0.00020
8.54	0.71034	0.71032	0.00022
8.50	0.71049	0.71035	0.00023
8.45	0.71054	0.71038	0.00024
8.41	0.70997	0.71038	0.00025
8.37	0.71048	0.71040	0.00024
8.32	0.71013	0.71044	0.00026
8.28	0.70961	0.71045	0.00025
8.24	0.71056	0.71051	0.00018
8.19	0.71023	0.71050	0.00018
8.15	0.71083	0.71049	0.00019
8.11	0.71069	0.71045	0.00017
8.06	0.71074	0.71040	0.00017
8.02	0.71059	0.71032	0.00017
7.98	0.71009	0.71032	0.00017
7.93	0.71088	0.71039	0.00018
7.89	0.71028	0.71033	0.00015
7.84	0.71020	0.71035	0.00015
7.80	0.71042	0.71045	0.00021
7.76	0.71014	0.71045	0.00021
7.71	0.71043	0.71047	0.00020
7.67	0.71024	0.71043	0.00022
7.63	0.70998	0.71051	0.00024
7.58	0.71057	0.71051	0.00024
7.54	0.71078	0.71048	0.00024
7.50	0.71024	0.71042	0.00023
7.45	0.71053	0.71046	0.00023

7.41	0.71113	0.71041	0.00025
7.37	0.71051	0.71024	0.00026
7.32	0.71032	0.71021	0.00025
7.28	0.71004	0.71019	0.00025
7.23	0.71095	0.71030	0.00030
7.19	0.70999	0.71019	0.00028
7.15	0.71026	0.71020	0.00027
7.10	0.71026	0.71026	0.00030
7.06	0.71059	0.71028	0.00030
7.02	0.71003	0.71025	0.00029
6.97	0.70943	0.71026	0.00029
6.93	0.71022	0.71038	0.00023
6.89	0.71017	0.71039	0.00023
6.84	0.71107	0.71039	0.00023
6.80	0.70986	0.71034	0.00018
6.76	0.71015	0.71033	0.00020
6.71	0.71081	0.71037	0.00020
6.67	0.71047	0.71034	0.00018
6.62	0.71035	0.71030	0.00018
6.58	0.71012	0.71036	0.00023
6.54	0.71061	0.71041	0.00022
6.49	0.71029	0.71041	0.00022
6.45	0.71021	0.71042	0.00022
6.41	0.71056	0.71043	0.00022
6.36	0.70969	0.71045	0.00024
6.32	0.71061	0.71047	0.00022
6.28	0.71048	0.71042	0.00023
6.23	0.71008	0.71044	0.00023
6.19	0.71096	0.71054	0.00025
6.15	0.71063	0.71052	0.00024
6.10	0.71059	0.71049	0.00024
6.06	0.71041	0.71046	0.00024
6.01	0.71023	0.71047	0.00024
5.97	0.71085	0.71041	0.00030
5.93	0.70984	0.71041	0.00030
5.88	0.71009	0.71047	0.00027
5.84	0.71068	0.71057	0.00028
5.80	0.71110	0.71055	0.00028
5.75	0.71076	0.71045	0.00026
5.71	0.71036	0.71044	0.00025
5.67	0.71030	0.71047	0.00026
5.62	0.71052	0.71042	0.00030
5.58	0.70956	0.71038	0.00030
5.53	0.71086	0.71044	0.00025
5.49	0.71052	0.71037	0.00024
5.45	0.71100	0.71033	0.00024
5.40	0.71053	0.71030	0.00020
5.36	0.71013	0.71026	0.00020
5.32	0.71061	0.71027	0.00020
5.27	0.71070	0.71027	0.00020

5.23	0.70971	0.71029	0.00022
5.19	0.71014	0.71032	0.00019
5.14	0.71019	0.71037	0.00020
5.10	0.71016	0.71034	0.00022
5.06	0.71009	0.71036	0.00022
5.01	0.71069	0.71034	0.00023
4.97	0.71015	0.71027	0.00023
4.92	0.71019	0.71046	0.00042
4.88	0.71069	0.71053	0.00042
4.84	0.71087	0.71052	0.00042
4.79	0.71002	0.71046	0.00041
4.75	0.71066	0.71051	0.00040
4.71	0.70987	0.71048	0.00040
4.66	0.71038	0.71047	0.00041
4.62	0.70988	0.71039	0.00044
4.58	0.71003	0.71046	0.00043
4.53	0.71204	0.71050	0.00042
4.49	0.71084	0.71027	0.00027
4.45	0.71062	0.71025	0.00026
4.40	0.71026	0.71022	0.00024
4.36	0.71050	0.71018	0.00025
4.31	0.71035	0.71020	0.00026
4.27	0.70976	0.71015	0.00027
4.23	0.70960	0.71020	0.00025
4.18	0.71058	0.71024	0.00022
4.14	0.71049	0.71024	0.00022
4.10	0.70971	0.71028	0.00025
4.05	0.71063	0.71032	0.00022
4.01	0.71028	0.71030	0.00021
3.97	0.70992	0.71040	0.00029
3.92	0.71068	0.71045	0.00027
3.88	0.70986	0.71040	0.00026
3.84	0.71028	0.71042	0.00025
3.79	0.71003	0.71042	0.00025
3.75	0.71054	0.71046	0.00023
3.70	0.71088	0.71043	0.00024
3.66	0.71012	0.71034	0.00022
3.62	0.71039	0.71043	0.00025
3.57	0.71127	0.71043	0.00025
3.53	0.71043	0.71033	0.00016
3.49	0.71023	0.71034	0.00017
3.44	0.71006	0.71036	0.00017
3.40	0.71030	0.71041	0.00015
3.36	0.71043	0.71041	0.00015
3.31	0.71018	0.71037	0.00017
3.27	0.71004	0.71043	0.00019
3.23	0.71093	0.71052	0.00019
3.18	0.71045	0.71049	0.00017
3.14	0.71026	0.71049	0.00017
3.09	0.71054	0.71046	0.00020

3.05	0.71046	0.71045	0.00020
3.01	0.71052	0.71046	0.00020
2.96	0.71032	0.71042	0.00021
2.92	0.71000	0.71037	0.00024
2.88	0.71083	0.71043	0.00023
2.83	0.71088	0.71040	0.00021
2.79	0.71063	0.71034	0.00018
2.75	0.71047	0.71030	0.00017
2.70	0.70990	0.71027	0.00017
2.66	0.71053	0.71031	0.00015
2.61	0.71049	0.71036	0.00020
2.57	0.71019	0.71031	0.00021
2.53	0.70981	0.71035	0.00021
2.48	0.71053	0.71037	0.00019
2.44	0.71052	0.71038	0.00020
2.40	0.71036	0.71033	0.00021
2.35	0.71016	0.71029	0.00023
2.31	0.71020	0.71029	0.00022
2.27	0.71032	0.71035	0.00024
2.22	0.71103	0.71032	0.00025
2.18	0.71001	0.71021	0.00020
2.14	0.71052	0.71022	0.00020
2.09	0.71004	0.71032	0.00032
2.05	0.71068	0.71037	0.00032
2.00	0.70998	0.71035	0.00031
1.96	0.70993	0.71040	0.00030
1.92	0.71021	0.71040	0.00030
1.87	0.71078	0.71038	0.00031
1.83	0.71002	0.71037	0.00031
1.79	0.70999	0.71040	0.00030
1.74	0.71008	0.71040	0.00030
1.70	0.71147	0.71040	0.00029
1.66	0.71061	0.71026	0.00019
1.61	0.71045	0.71026	0.00019
1.57	0.71046	0.71029	0.00021
1.53	0.70988	0.71025	0.00021
1.48	0.71002	0.71029	0.00020
1.44	0.71072	0.71032	0.00019
1.39	0.71031	0.71040	0.00030
1.35	0.71000	0.71048	0.00033
1.31	0.71012	0.71055	0.00031
1.26	0.70998	0.71065	0.00031
1.22	0.71068	0.71077	0.00029
1.18	0.71072	0.71074	0.00030
1.13	0.71004	0.71069	0.00032
1.09	0.71028	0.71078	0.00029
1.05	0.71037	0.71086	0.00027
1.00	0.71152	0.71090	0.00025
0.96	0.71112	0.71085	0.00021
0.92	0.71064	0.71080	0.00021

0.87	0.71110	0.71084	0.00021
0.83	0.71121	0.71080	0.00021
0.78	0.71041	0.71082	0.00023
0.74	0.71019	0.71086	0.00021
0.70	0.71100	0.71102	0.00023
0.65	0.71104	0.71095	0.00027
0.61	0.71076	0.71092	0.00027
0.57	0.71104	0.71098	0.00028
0.52	0.71059	0.71096	0.00028
0.48	0.71104	0.71107	0.00030
0.44	0.71072	0.71105	0.00030
0.39	0.71146	0.71108	0.00031
0.35	0.71078	0.71104	0.00032
0.31	0.71178	0.71108	0.00034
0.26	0.71031	0.71096	0.00031
0.22	0.71077	0.71109	0.00026
0.17	0.71129	0.71117	0.00027
0.13	0.71084	0.71113	0.00032
0.09	0.71172	0.71127	0.00040
0.04	0.71082		

ARB 33.3.1 (M3)

Distance from cervix (mm)	$^{87}Sr/^{86}Sr$	10 point mov. average	2 SE on mov. average
29.58	0.71067	0.71048	0.00021
29.54	0.71065	0.71040	0.00023
29.49	0.71067	0.71042	0.00025
29.45	0.71033	0.71043	0.00025
29.41	0.71076	0.71045	0.00025
29.36	0.71042	0.71041	0.00024
29.32	0.71085	0.71038	0.00025
29.28	0.70973	0.71034	0.00022
29.23	0.71025	0.71043	0.00018
29.19	0.71041	0.71052	0.00023
29.15	0.70989	0.71054	0.00023
29.10	0.71086	0.71065	0.00019
29.06	0.71075	0.71064	0.00018
29.02	0.71057	0.71071	0.00025
28.97	0.71034	0.71077	0.00026
28.93	0.71018	0.71071	0.00032
28.89	0.71043	0.71073	0.00031
28.84	0.71059	0.71069	0.00034
28.80	0.71116	0.71060	0.00039
28.75	0.71067	0.71046	0.00040
28.71	0.71097	0.71047	0.00041
28.67	0.71076	0.71040	0.00039
28.62	0.71145	0.71036	0.00038
28.58	0.71116	0.71019	0.00031
28.54	0.70974	0.71002	0.00026
28.49	0.71034	0.71011	0.00028
28.45	0.71006	0.71014	0.00030
28.41	0.70968	0.71018	0.00030
28.36	0.70977	0.71019	0.00029
28.32	0.71080	0.71008	0.00042
28.28	0.71025	0.70994	0.00040
28.23	0.71032	0.70994	0.00040
28.19	0.70982	0.70987	0.00040
28.15	0.70941	0.70990	0.00040
28.10	0.71070	0.70989	0.00041
28.06	0.71058	0.70975	0.00037
28.02	0.71044	0.70959	0.00035
27.97	0.70980	0.70951	0.00030
27.93	0.70864	0.70955	0.00032
27.89	0.70947	0.70964	0.00026
27.84	0.71019	0.70967	0.00026
27.80	0.70962	0.70954	0.00027
27.76	0.71011	0.70956	0.00028
27.71	0.70930	0.70954	0.00027
27.67	0.70937	0.70961	0.00027
27.63	0.70898	0.70960	0.00027
27.58	0.70959	0.70966	0.00024

27.54	0.71018	0.70964	0.00024
27.50	0.70953	0.70964	0.00024
27.45	0.70985	0.70968	0.00025
27.41	0.70887	0.70970	0.00026
27.36	0.70980	0.70965	0.00034
27.32	0.70994	0.70967	0.00034
27.28	0.70994	0.70958	0.00036
27.23	0.70930	0.70953	0.00035
27.19	0.70959	0.70951	0.00036
27.15	0.70942	0.70950	0.00036
27.10	0.71019	0.70947	0.00036
27.06	0.70986	0.70940	0.00033
27.02	0.71011	0.70931	0.00032
26.97	0.70837	0.70921	0.00027
26.93	0.71000	0.70938	0.00024
26.89	0.70906	0.70937	0.00023
26.84	0.70938	0.70941	0.00022
26.80	0.70916	0.70953	0.00032
26.76	0.70946	0.70960	0.00031
26.71	0.70913	0.70956	0.00033
26.67	0.70945	0.70964	0.00032
26.63	0.70896	0.70962	0.00032
26.58	0.70909	0.70963	0.00032
26.54	0.71005	0.70953	0.00043
26.50	0.70991	0.70952	0.00043
26.45	0.70955	0.70953	0.00043
26.41	0.71053	0.70949	0.00044
26.37	0.70985	0.70934	0.00038
26.32	0.70908	0.70927	0.00036
26.28	0.70989	0.70925	0.00037
26.24	0.70928	0.70916	0.00034
26.19	0.70905	0.70919	0.00035
26.15	0.70809	0.70919	0.00035
26.11	0.70996	0.70935	0.00026
26.06	0.70999	0.70928	0.00023
26.02	0.70917	0.70920	0.00016
25.97	0.70904	0.70918	0.00017
25.93	0.70920	0.70924	0.00019
25.89	0.70883	0.70925	0.00019
25.84	0.70904	0.70936	0.00021
25.80	0.70953	0.70945	0.00023
25.76	0.70902	0.70948	0.00024
25.71	0.70970	0.70951	0.00022
25.67	0.70929	0.70956	0.00026
25.63	0.70924	0.70964	0.00027
25.58	0.70892	0.70962	0.00029
25.54	0.70967	0.70977	0.00028
25.50	0.70926	0.70985	0.00031
25.45	0.70989	0.70990	0.00028
25.41	0.70996	0.70979	0.00036

25.37	0.70986	0.70966	0.00042
25.32	0.70928	0.70970	0.00044
25.28	0.71019	0.70969	0.00044
25.24	0.71009	0.70969	0.00044
25.19	0.70907	0.70964	0.00043
25.15	0.71042	0.70975	0.00042
25.11	0.71044	0.70966	0.00040
25.06	0.70979	0.70955	0.00036
25.02	0.70881	0.70964	0.00043
24.98	0.70862	0.70975	0.00039
24.93	0.71029	0.70985	0.00030
24.89	0.70919	0.70974	0.00031
24.85	0.71018	0.70985	0.00030
24.80	0.70961	0.70981	0.00029
24.76	0.71010	0.70977	0.00032
24.72	0.70958	0.70966	0.00034
24.67	0.70930	0.70964	0.00035
24.63	0.71068	0.70962	0.00036
24.58	0.70999	0.70955	0.00029
24.54	0.70954	0.70946	0.00029
24.50	0.70921	0.70950	0.00030
24.45	0.71035	0.70954	0.00030
24.41	0.70978	0.70950	0.00026
24.37	0.70916	0.70942	0.00027
24.32	0.70906	0.70949	0.00028
24.28	0.70937	0.70950	0.00027
24.24	0.70906	0.70953	0.00027
24.19	0.71001	0.70965	0.00028
24.15	0.70903	0.70965	0.00028
24.11	0.70993	0.70976	0.00025
24.06	0.70968	0.70969	0.00027
24.02	0.70994	0.70970	0.00027
23.98	0.70894	0.70963	0.00027
23.93	0.70986	0.70968	0.00024
23.89	0.70921	0.70970	0.00025
23.85	0.70965	0.70983	0.00026
23.80	0.71022	0.70982	0.00026
23.76	0.71008	0.70978	0.00025
23.72	0.71005	0.70979	0.00025
23.67	0.70925	0.70972	0.00026
23.63	0.70979	0.70968	0.00029
23.59	0.70930	0.70970	0.00030
23.54	0.70939	0.70983	0.00034
23.50	0.71008	0.70985	0.00033
23.46	0.71044	0.70982	0.00032
23.41	0.70960	0.70981	0.00031
23.37	0.70978	0.70976	0.00034
23.33	0.71017	0.70976	0.00034
23.28	0.70936	0.70969	0.00033
23.24	0.70890	0.70963	0.00038

23.19	0.70992	0.70971	0.00035
23.15	0.71064	0.70977	0.00038
23.11	0.70961	0.70976	0.00037
23.06	0.70976	0.70973	0.00038
23.02	0.71034	0.70976	0.00038
22.98	0.70910	0.70962	0.00039
22.93	0.70976	0.70966	0.00038
22.89	0.70950	0.70963	0.00038
22.85	0.70872	0.70959	0.00039
22.80	0.70978	0.70965	0.00035
22.76	0.71051	0.70971	0.00038
22.72	0.71051	0.70958	0.00034
22.67	0.70931	0.70952	0.00028
22.63	0.71005	0.70955	0.00028
22.59	0.70896	0.70945	0.00027
22.54	0.70946	0.70946	0.00026
22.50	0.70952	0.70949	0.00027
22.46	0.70911	0.70955	0.00030
22.41	0.70926	0.70957	0.00029
22.37	0.71036	0.70963	0.00028
22.33	0.70930	0.70954	0.00023
22.28	0.70992	0.70959	0.00024
22.24	0.70952	0.70956	0.00022
22.20	0.70905	0.70948	0.00029
22.15	0.70905	0.70955	0.00028
22.11	0.70982	0.70958	0.00026
22.07	0.71013	0.70962	0.00028
22.02	0.70932	0.70954	0.00026
21.98	0.70981	0.70953	0.00026
21.94	0.70944	0.70950	0.00025
21.89	0.70986	0.70953	0.00025
21.85	0.70964	0.70945	0.00026
21.80	0.70867	0.70942	0.00026
21.76	0.70975	0.70953	0.00021
21.72	0.70940	0.70959	0.00026
21.67	0.71014	0.70963	0.00026
21.63	0.70936	0.70963	0.00026
21.59	0.70927	0.70963	0.00026
21.54	0.70950	0.70968	0.00024
21.50	0.70972	0.70967	0.00025
21.46	0.70902	0.70966	0.00025
21.41	0.70933	0.70976	0.00021
21.37	0.70980	0.70979	0.00019
21.33	0.71033	0.70978	0.00019
21.28	0.70986	0.70967	0.00017
21.24	0.71011	0.70961	0.00018
21.20	0.70940	0.70956	0.00015
21.15	0.70975	0.70961	0.00015
21.11	0.70936	0.70962	0.00016
21.07	0.70967	0.70957	0.00022

21.02	0.71001	0.70968	0.00032
20.98	0.70967	0.70966	0.00032
20.94	0.70962	0.70969	0.00032
20.89	0.70926	0.70972	0.00033
20.85	0.70928	0.70973	0.00032
20.81	0.70962	0.70974	0.00032
20.76	0.70982	0.70968	0.00034
20.72	0.70991	0.70967	0.00034
20.68	0.70884	0.70962	0.00034
20.63	0.71074	0.70972	0.00029
20.59	0.70987	0.70963	0.00019
20.55	0.70991	0.70958	0.00019
20.50	0.70997	0.70960	0.00021
20.46	0.70934	0.70959	0.00020
20.41	0.70939	0.70960	0.00020
20.37	0.70905	0.70960	0.00020
20.33	0.70964	0.70972	0.00021
20.28	0.70948	0.70975	0.00021
20.24	0.70979	0.70978	0.00020
20.20	0.70989	0.70981	0.00022
20.15	0.70934	0.70976	0.00023
20.11	0.71006	0.70981	0.00021
20.07	0.70993	0.70982	0.00022
20.02	0.70946	0.70983	0.00022
19.98	0.70933	0.70988	0.00021
19.94	0.71032	0.70989	0.00020
19.89	0.70991	0.70978	0.00021
19.85	0.70973	0.70973	0.00021
19.81	0.71015	0.70971	0.00022
19.76	0.70940	0.70965	0.00020
19.72	0.70981	0.70964	0.00020
19.68	0.71019	0.70966	0.00022
19.63	0.71000	0.70956	0.00020
19.59	0.70996	0.70952	0.00017
19.55	0.70939	0.70951	0.00017
19.50	0.70927	0.70950	0.00017
19.46	0.70944	0.70953	0.00016
19.42	0.70949	0.70950	0.00018
19.37	0.70955	0.70950	0.00018
19.33	0.70932	0.70948	0.00018
19.29	0.71003	0.70948	0.00018
19.24	0.70920	0.70944	0.00014
19.20	0.70956	0.70944	0.00014
19.16	0.70988	0.70942	0.00014
19.11	0.70929	0.70942	0.00014
19.07	0.70950	0.70946	0.00015
19.03	0.70917	0.70946	0.00015
18.98	0.70945	0.70950	0.00013
18.94	0.70942	0.70947	0.00016
18.89	0.70926	0.70947	0.00016

18.85	0.70964	0.70951	0.00016
18.81	0.70920	0.70949	0.00016
18.76	0.70938	0.70955	0.00015
18.72	0.70988	0.70962	0.00019
18.68	0.70974	0.70959	0.00018
18.63	0.70944	0.70956	0.00018
18.59	0.70963	0.70955	0.00018
18.55	0.70907	0.70956	0.00018
18.50	0.70942	0.70963	0.00015
18.46	0.70968	0.70969	0.00016
18.42	0.70944	0.70970	0.00016
18.37	0.70979	0.70972	0.00015
18.33	0.71015	0.70974	0.00016
18.29	0.70953	0.70960	0.00023
18.24	0.70942	0.70965	0.00025
18.20	0.70938	0.70968	0.00025
18.16	0.70968	0.70970	0.00024
18.11	0.70982	0.70968	0.00024
18.07	0.71001	0.70973	0.00028
18.03	0.70975	0.70977	0.00031
17.98	0.70968	0.70974	0.00032
17.94	0.71000	0.70972	0.00033
17.90	0.70874	0.70969	0.00032
17.85	0.71006	0.70979	0.00024
17.81	0.70968	0.70970	0.00026
17.77	0.70962	0.70973	0.00027
17.72	0.70940	0.70971	0.00028
17.68	0.71032	0.70970	0.00028
17.64	0.71048	0.70966	0.00025
17.59	0.70939	0.70951	0.00021
17.55	0.70945	0.70953	0.00020
17.50	0.70969	0.70956	0.00021
17.46	0.70984	0.70965	0.00030
17.42	0.70915	0.70964	0.00030
17.37	0.70996	0.70973	0.00029
17.33	0.70937	0.70966	0.00029
17.29	0.70936	0.70968	0.00029
17.24	0.70989	0.70972	0.00028
17.20	0.70899	0.70961	0.00033
17.16	0.70955	0.70969	0.00030
17.11	0.70976	0.70967	0.00030
17.07	0.71064	0.70962	0.00031
17.03	0.70975	0.70953	0.00022
16.98	0.71004	0.70947	0.00023
16.94	0.70927	0.70947	0.00023
16.90	0.70954	0.70953	0.00023
16.85	0.70976	0.70948	0.00024
16.81	0.70881	0.70951	0.00026
16.77	0.70976	0.70955	0.00022
16.72	0.70942	0.70952	0.00021

16.68	0.70925	0.70951	0.00022
16.64	0.70971	0.70962	0.00026
16.59	0.70918	0.70963	0.00027
16.55	0.71003	0.70976	0.00030
16.51	0.70978	0.70977	0.00030
16.46	0.70913	0.70982	0.00032
16.42	0.70998	0.70982	0.00032
16.38	0.70926	0.70975	0.00034
16.33	0.70940	0.70973	0.00035
16.29	0.70932	0.70985	0.00038
16.25	0.71034	0.70986	0.00038
16.20	0.70988	0.70984	0.00037
16.16	0.71049	0.70984	0.00037
16.11	0.71011	0.70965	0.00041
16.07	0.71031	0.70964	0.00041
16.03	0.70914	0.70955	0.00038
15.98	0.70919	0.70963	0.00038
15.94	0.70912	0.70967	0.00037
15.90	0.71063	0.70971	0.00035
15.85	0.70937	0.70959	0.00028
15.81	0.71011	0.70964	0.00028
15.77	0.70994	0.70960	0.00027
15.72	0.70859	0.70960	0.00026
15.68	0.71003	0.70978	0.00020
15.64	0.70942	0.70972	0.00020
15.59	0.70991	0.70974	0.00019
15.55	0.70957	0.70972	0.00018
15.51	0.70953	0.70973	0.00018
15.46	0.70940	0.70971	0.00019
15.42	0.70989	0.70973	0.00018
15.38	0.70976	0.70979	0.00023
15.33	0.70991	0.70985	0.00025
15.29	0.71038	0.70990	0.00028
15.25	0.70944	0.70981	0.00027
15.20	0.70962	0.70980	0.00028
15.16	0.70969	0.70976	0.00030
15.12	0.70967	0.70972	0.00032
15.07	0.70937	0.70967	0.00033
15.03	0.70961	0.70981	0.00039
14.99	0.71044	0.70995	0.00044
14.94	0.71035	0.70983	0.00045
14.90	0.71046	0.70982	0.00044
14.86	0.70950	0.70986	0.00047
14.81	0.70930	0.70997	0.00048
14.77	0.70926	0.70998	0.00048
14.72	0.70922	0.71000	0.00046
14.68	0.70923	0.71010	0.00043
14.64	0.71077	0.71021	0.00039
14.59	0.71092	0.71019	0.00038
14.55	0.70930	0.71011	0.00034

14.51	0.71027	0.71013	0.00033
14.46	0.71080	0.71021	0.00039
14.42	0.71060	0.71012	0.00037
14.38	0.70938	0.71015	0.00039
14.33	0.70950	0.71017	0.00037
14.29	0.71023	0.71015	0.00039
14.25	0.71028	0.71011	0.00039
14.20	0.71066	0.71022	0.00047
14.16	0.71011	0.71012	0.00047
14.12	0.70944	0.71009	0.00048
14.07	0.71114	0.71012	0.00046
14.03	0.70986	0.70994	0.00042
13.99	0.71089	0.70988	0.00044
13.94	0.70961	0.70986	0.00042
13.90	0.70932	0.70983	0.00043
13.86	0.70980	0.70993	0.00042
13.81	0.71138	0.70997	0.00042
13.77	0.70964	0.70981	0.00028
13.73	0.70977	0.70978	0.00029
13.68	0.70974	0.70977	0.00030
13.64	0.70940	0.70979	0.00030
13.60	0.70928	0.70984	0.00029
13.55	0.71064	0.70994	0.00027
13.51	0.70935	0.70985	0.00022
13.47	0.71031	0.70979	0.00030
13.42	0.71022	0.70967	0.00031
13.38	0.70976	0.70965	0.00030
13.33	0.70938	0.70964	0.00030
13.29	0.70960	0.70976	0.00034
13.25	0.70992	0.70980	0.00034
13.20	0.70992	0.70981	0.00034
13.16	0.71030	0.70980	0.00034
13.12	0.70977	0.70973	0.00033
13.07	0.70876	0.70975	0.00033
13.03	0.70903	0.70982	0.00026
12.99	0.71006	0.70992	0.00018
12.94	0.70969	0.70989	0.00018
12.90	0.71052	0.70996	0.00021
12.86	0.71000	0.70994	0.00018
12.81	0.71004	0.70986	0.00023
12.77	0.70983	0.70982	0.00023
12.73	0.70955	0.70994	0.00033
12.68	0.70999	0.70993	0.00034
12.64	0.70952	0.70993	0.00034
12.60	0.70997	0.70998	0.00032
12.55	0.70975	0.71009	0.00040
12.51	0.71044	0.71015	0.00039
12.47	0.71025	0.71013	0.00039
12.42	0.70925	0.71010	0.00039
12.38	0.70966	0.71021	0.00034

12.34	0.71103	0.71030	0.00033
12.29	0.70944	0.71017	0.00031
12.25	0.71002	0.71021	0.00027
12.21	0.70999	0.71016	0.00031
12.16	0.71113	0.71016	0.00031
12.12	0.71032	0.71002	0.00023
12.08	0.71025	0.70997	0.00022
12.03	0.70989	0.70996	0.00021
11.99	0.71036	0.70993	0.00023
11.94	0.71062	0.70986	0.00021
11.90	0.70965	0.70987	0.00024
11.86	0.70990	0.70986	0.00025
11.81	0.70948	0.70989	0.00026
11.77	0.71001	0.70994	0.00024
11.73	0.70973	0.70997	0.00025
11.68	0.70986	0.70995	0.00026
11.64	0.71012	0.70990	0.00028
11.60	0.70957	0.70981	0.00031
11.55	0.70964	0.70979	0.00032
11.51	0.71077	0.70968	0.00041
11.47	0.70948	0.70954	0.00033
11.42	0.71022	0.70956	0.00033
11.38	0.71003	0.70957	0.00034
11.34	0.71027	0.70954	0.00032
11.29	0.70950	0.70947	0.00028
11.25	0.70942	0.70953	0.00031
11.21	0.70921	0.70949	0.00032
11.16	0.70934	0.70952	0.00032
11.12	0.70855	0.70953	0.00031
11.08	0.70942	0.70971	0.00027
11.03	0.70963	0.70970	0.00027
10.99	0.71033	0.70971	0.00027
10.95	0.70975	0.70961	0.00024
10.90	0.70956	0.70961	0.00024
10.86	0.71007	0.70960	0.00024
10.82	0.70902	0.70956	0.00022
10.77	0.70954	0.70961	0.00018
10.73	0.70945	0.70970	0.00024
10.69	0.71034	0.70968	0.00025
10.64	0.70934	0.70961	0.00021
10.60	0.70974	0.70971	0.00023
10.55	0.70932	0.70968	0.00024
10.51	0.70968	0.70965	0.00026
10.47	0.70950	0.70963	0.00026
10.42	0.70968	0.70962	0.00026
10.38	0.70951	0.70962	0.00026
10.34	0.71040	0.70960	0.00027
10.29	0.70925	0.70952	0.00020
10.25	0.70970	0.70957	0.00020
10.21	0.71027	0.70963	0.00025

10.16	0.70943	0.70956	0.00020
10.12	0.70910	0.70964	0.00023
10.08	0.70946	0.70970	0.00020
10.03	0.70943	0.70972	0.00019
9.99	0.70963	0.70979	0.00019
9.95	0.70929	0.70983	0.00020
9.90	0.70965	0.70992	0.00017
9.86	0.70971	0.70993	0.00016
9.82	0.71030	0.70996	0.00015
9.77	0.70965	0.70992	0.00013
9.73	0.71018	0.70996	0.00012
9.69	0.70969	0.70998	0.00015
9.64	0.70968	0.71005	0.00014
9.60	0.71009	0.71015	0.00017
9.56	0.71003	0.71011	0.00019
9.51	0.71020	0.71011	0.00019
9.47	0.70981	0.71004	0.00023
9.43	0.70997	0.71016	0.00030
9.38	0.70991	0.71009	0.00035
9.34	0.71000	0.71007	0.00035
9.30	0.71045	0.71002	0.00037
9.25	0.71031	0.70992	0.00038
9.21	0.71069	0.70986	0.00037
9.17	0.70970	0.70982	0.00034
9.12	0.71008	0.70982	0.00034
9.08	0.70946	0.70975	0.00034
9.03	0.71106	0.70992	0.00043
8.99	0.70929	0.70983	0.00036
8.95	0.70968	0.70984	0.00035
8.90	0.70954	0.70996	0.00041
8.86	0.70939	0.71004	0.00040
8.82	0.70974	0.71004	0.00040
8.77	0.71031	0.71012	0.00040
8.73	0.70962	0.71013	0.00040
8.69	0.70943	0.71019	0.00039
8.64	0.71116	0.71025	0.00035
8.60	0.71013	0.71013	0.00029
8.56	0.70937	0.71019	0.00032
8.51	0.71089	0.71026	0.00026
8.47	0.71036	0.71017	0.00023
8.43	0.70944	0.71008	0.00026
8.38	0.71048	0.71028	0.00034
8.34	0.71040	0.71040	0.00043
8.30	0.71020	0.71041	0.00043
8.25	0.71008	0.71034	0.00047
8.21	0.70992	0.71044	0.00049
8.17	0.71076	0.71052	0.00048
8.12	0.71011	0.71056	0.00050
8.08	0.70993	0.71061	0.00048
8.04	0.70950	0.71064	0.00047

7.99	0.71147	0.71082	0.00040
7.95	0.71161	0.71078	0.00038
7.91	0.71049	0.71072	0.00034
7.86	0.70949	0.71079	0.00035
7.82	0.71113	0.71103	0.00028
7.78	0.71067	0.71100	0.00028
7.73	0.71119	0.71108	0.00029
7.69	0.71063	0.71110	0.00030
7.64	0.71028	0.71115	0.00028
7.60	0.71126	0.71116	0.00026
7.56	0.71101	0.71113	0.00026
7.51	0.71110	0.71114	0.00026
7.47	0.71117	0.71109	0.00028
7.43	0.71192	0.71106	0.00028
7.38	0.71075	0.71094	0.00021
7.34	0.71147	0.71093	0.00022
7.30	0.71140	0.71087	0.00018
7.25	0.71110	0.71082	0.00014
7.21	0.71043	0.71081	0.00013
7.17	0.71100	0.71084	0.00010
7.12	0.71103	0.71080	0.00011
7.08	0.71061	0.71083	0.00015
7.04	0.71094	0.71083	0.00015
6.99	0.71064	0.71077	0.00017
6.95	0.71063	0.71081	0.00017
6.91	0.71090	0.71085	0.00017
6.86	0.71092	0.71082	0.00018
6.82	0.71097	0.71079	0.00018
6.78	0.71079	0.71080	0.00018
6.73	0.71057	0.71077	0.00019
6.69	0.71133	0.71074	0.00021
6.65	0.71059	0.71069	0.00016
6.60	0.71040	0.71071	0.00016
6.56	0.71099	0.71072	0.00016
6.52	0.71101	0.71074	0.00017
6.47	0.71062	0.71069	0.00017
6.43	0.71067	0.71070	0.00017
6.39	0.71100	0.71070	0.00017
6.34	0.71053	0.71063	0.00018
6.30	0.71030	0.71059	0.00020
6.25	0.71076	0.71059	0.00020
6.21	0.71086	0.71067	0.00028
6.17	0.71044	0.71059	0.00030
6.12	0.71118	0.71059	0.00030
6.08	0.71054	0.71056	0.00028
6.04	0.71072	0.71060	0.00028
5.99	0.71070	0.71049	0.00034
5.95	0.71024	0.71044	0.00034
5.91	0.71014	0.71034	0.00042
5.86	0.71030	0.71039	0.00042

5.82	0.71157	0.71039	0.00042
5.78	0.71012	0.71023	0.00033
5.73	0.71044	0.71021	0.00034
5.69	0.71087	0.71021	0.00034
5.65	0.71088	0.71015	0.00031
5.60	0.70961	0.71013	0.00030
5.56	0.71023	0.71021	0.00028
5.52	0.70923	0.71021	0.00028
5.47	0.71064	0.71031	0.00017
5.43	0.71029	0.71032	0.00018
5.39	0.70998	0.71037	0.00020
5.34	0.70987	0.71044	0.00020
5.30	0.71053	0.71044	0.00020
5.26	0.71019	0.71040	0.00021
5.21	0.71074	0.71051	0.00026
5.17	0.71040	0.71050	0.00026
5.13	0.71019	0.71044	0.00029
5.08	0.71025	0.71049	0.00029
5.04	0.71074	0.71046	0.00031
5.00	0.71077	0.71042	0.00030
4.95	0.71074	0.71038	0.00029
4.91	0.70982	0.71030	0.00029
4.86	0.71020	0.71038	0.00028
4.82	0.71127	0.71035	0.00030
4.78	0.71057	0.71026	0.00022
4.73	0.70986	0.71030	0.00025
4.69	0.71071	0.71035	0.00023
4.65	0.70988	0.71036	0.00024
4.60	0.71040	0.71041	0.00021
4.56	0.71029	0.71041	0.00021
4.52	0.70997	0.71044	0.00021
4.47	0.71069	0.71034	0.00036
4.43	0.70985	0.71034	0.00036
4.39	0.71043	0.71041	0.00035
4.34	0.71091	0.71049	0.00038
4.30	0.71038	0.71038	0.00039
4.26	0.71081	0.71038	0.00039
4.21	0.71033	0.71032	0.00038
4.17	0.71041	0.71026	0.00040
4.13	0.71061	0.71023	0.00040
4.08	0.70895	0.71026	0.00041
4.04	0.71067	0.71039	0.00029
4.00	0.71059	0.71044	0.00034
3.95	0.71122	0.71050	0.00037
3.91	0.70979	0.71045	0.00034
3.87	0.71045	0.71045	0.00034
3.82	0.71015	0.71045	0.00034
3.78	0.70980	0.71045	0.00034
3.74	0.71011	0.71050	0.00031
3.69	0.71089	0.71049	0.00032

3.65	0.71019	0.71046	0.00031
3.61	0.71122	0.71040	0.00035
3.56	0.71123	0.71034	0.00030
3.52	0.71068	0.71022	0.00024
3.47	0.70976	0.71021	0.00023
3.43	0.71049	0.71033	0.00025
3.39	0.71013	0.71028	0.00025
3.34	0.71035	0.71031	0.00025
3.30	0.70998	0.71033	0.00025
3.26	0.71054	0.71039	0.00024
3.21	0.70963	0.71034	0.00025
3.17	0.71067	0.71038	0.00021
3.13	0.71000	0.71034	0.00020
3.08	0.71055	0.71034	0.00020
3.04	0.71093	0.71036	0.00022
3.00	0.71004	0.71033	0.00019
2.95	0.71044	0.71035	0.00018
2.91	0.71050	0.71034	0.00018
2.87	0.71060	0.71029	0.00019
2.82	0.71000	0.71026	0.00018
2.78	0.71003	0.71027	0.00017
2.74	0.71027	0.71033	0.00018
2.69	0.71000	0.71036	0.00019
2.65	0.71080	0.71045	0.00019
2.61	0.71065	0.71032	0.00025
2.56	0.71024	0.71027	0.00025
2.52	0.71035	0.71033	0.00027
2.48	0.70998	0.71035	0.00027
2.43	0.71024	0.71049	0.00033
2.39	0.71013	0.71049	0.00033
2.35	0.71065	0.71061	0.00036
2.30	0.71063	0.71072	0.00043
2.26	0.71088	0.71069	0.00044
2.22	0.70951	0.71063	0.00044
2.17	0.71009	0.71077	0.00037
2.13	0.71081	0.71090	0.00035
2.08	0.71060	0.71086	0.00036
2.04	0.71140	0.71090	0.00036
2.00	0.71020	0.71076	0.00038
1.95	0.71137	0.71080	0.00036
1.91	0.71175	0.71078	0.00035
1.87	0.71034	0.71071	0.00029
1.82	0.71020	0.71075	0.00027
1.78	0.71091	0.71083	0.00025
1.74	0.71137	0.71078	0.00026
1.69	0.71044	0.71067	0.00024
1.65	0.71102	0.71063	0.00028
1.61	0.71003	0.71054	0.00027
1.56	0.71059	0.71060	0.00025
1.52	0.71111	0.71058	0.00025

1.48	0.71112	0.71049	0.00023
1.43	0.71069	0.71042	0.00018
1.39	0.71101	0.71036	0.00018
1.35	0.71038	0.71028	0.00010
1.30	0.71032	0.71031	0.00013
1.26	0.70999	0.71035	0.00015
1.22	0.71020	0.71033	0.00017
1.17	0.71054	0.71035	0.00017
1.13	0.71044	0.71039	0.00020
1.09	0.71024	0.71046	0.00026
1.04	0.71038	0.71050	0.00025
1.00	0.71008	0.71053	0.00026
0.96	0.71027	0.71052	0.00027
0.91	0.71064	0.71053	0.00026
0.87	0.71074	0.71050	0.00026
0.83	0.70981	0.71048	0.00026
0.78	0.71035	0.71048	0.00026
0.74	0.71093	0.71047	0.00026
0.69	0.71118	0.71040	0.00024
0.65	0.71059	0.71026	0.00019
0.61	0.71068	0.71022	0.00018
0.56	0.70996	0.71018	0.00015
0.52	0.71037	0.71021	0.00014
0.48	0.71039	0.71018	0.00014
0.43	0.71050	0.71020	0.00016
0.39	0.70982	0.71017	0.00015
0.35	0.71029	0.71021	0.00014
0.30	0.71020	0.71020	0.00014
0.26	0.70983	0.71020	0.00016
0.22	0.71012	0.71027	0.00012
0.17	0.71032	0.71031	0.00013
0.13	0.71027	0.71030	0.00016
0.09	0.71007	0.71032	0.00023
0.04	0.71057		

ARB 34.2.1 (M2)

Distance from cervix (mm)	$^{87}Sr/^{86}Sr$	10 point mov. average	2 SE on mov. average
46.50	0.71049	0.71038	0.00007
46.46	0.71051	0.71039	0.00008
46.41	0.71041	0.71034	0.00011
46.37	0.71027	0.71032	0.00011
46.32	0.71018	0.71035	0.00012
46.28	0.71036	0.71039	0.00011
46.24	0.71029	0.71037	0.00012
46.19	0.71049	0.71036	0.00012
46.15	0.71029	0.71034	0.00012
46.11	0.71048	0.71032	0.00013
46.06	0.71060	0.71031	0.00013
46.02	0.70999	0.71022	0.00015
45.97	0.71029	0.71027	0.00015
45.93	0.71056	0.71031	0.00017
45.89	0.71051	0.71029	0.00016
45.84	0.71021	0.71030	0.00017
45.80	0.71019	0.71034	0.00017
45.76	0.71034	0.71034	0.00017
45.71	0.71008	0.71034	0.00017
45.67	0.71032	0.71042	0.00019
45.62	0.70976	0.71046	0.00020
45.58	0.71049	0.71055	0.00012
45.54	0.71065	0.71056	0.00012
45.49	0.71035	0.71054	0.00012
45.45	0.71061	0.71058	0.00012
45.41	0.71057	0.71051	0.00018
45.36	0.71022	0.71051	0.00018
45.32	0.71038	0.71055	0.00017
45.27	0.71089	0.71054	0.00018
45.23	0.71073	0.71053	0.00017
45.19	0.71059	0.71049	0.00017
45.14	0.71058	0.71049	0.00017
45.10	0.71045	0.71048	0.00017
45.06	0.71075	0.71044	0.00019
45.01	0.70991	0.71039	0.00018
44.97	0.71063	0.71046	0.00014
44.92	0.71061	0.71045	0.00014
44.88	0.71025	0.71046	0.00014
44.84	0.71085	0.71052	0.00016
44.79	0.71030	0.71051	0.00015
44.75	0.71053	0.71057	0.00017
44.71	0.71048	0.71060	0.00017
44.66	0.71011	0.71064	0.00018
44.62	0.71024	0.71072	0.00014
44.57	0.71061	0.71076	0.00009
44.53	0.71052	0.71080	0.00009
44.49	0.71069	0.71083	0.00007

44.44	0.71085	0.71081	0.00010
44.40	0.71074	0.71083	0.00010
44.36	0.71098	0.71084	0.00010
44.31	0.71079	0.71080	0.00011
44.27	0.71092	0.71085	0.00015
44.22	0.71088	0.71087	0.00016
44.18	0.71067	0.71088	0.00016
44.14	0.71093	0.71089	0.00016
44.09	0.71090	0.71089	0.00016
44.05	0.71046	0.71088	0.00016
44.01	0.71100	0.71094	0.00013
43.96	0.71083	0.71096	0.00014
43.92	0.71060	0.71101	0.00015
43.87	0.71135	0.71104	0.00013
43.83	0.71110	0.71103	0.00012
43.79	0.71101	0.71103	0.00012
43.74	0.71074	0.71101	0.00013
43.70	0.71091	0.71103	0.00011
43.66	0.71082	0.71105	0.00011
43.61	0.71110	0.71106	0.00010
43.57	0.71114	0.71107	0.00011
43.52	0.71133	0.71106	0.00011
43.48	0.71095	0.71104	0.00010
43.44	0.71125	0.71099	0.00015
43.39	0.71106	0.71093	0.00015
43.35	0.71080	0.71092	0.00015
43.31	0.71092	0.71092	0.00015
43.26	0.71110	0.71090	0.00016
43.22	0.71091	0.71090	0.00016
43.17	0.71128	0.71092	0.00017
43.13	0.71103	0.71090	0.00015
43.09	0.71107	0.71088	0.00015
43.04	0.71047	0.71083	0.00015
43.00	0.71064	0.71084	0.00014
42.96	0.71098	0.71088	0.00014
42.91	0.71081	0.71089	0.00015
42.87	0.71066	0.71093	0.00016
42.82	0.71119	0.71091	0.00018
42.78	0.71106	0.71084	0.00019
42.74	0.71105	0.71079	0.00019
42.69	0.71087	0.71079	0.00019
42.65	0.71062	0.71077	0.00019
42.61	0.71053	0.71079	0.00019
42.56	0.71107	0.71078	0.00020
42.52	0.71109	0.71079	0.00020
42.47	0.71120	0.71077	0.00019
42.43	0.71038	0.71070	0.00017
42.39	0.71056	0.71073	0.00016
42.34	0.71051	0.71078	0.00016
42.30	0.71106	0.71079	0.00016

42.26	0.71069	0.71075	0.00014
42.21	0.71085	0.71071	0.00017
42.17	0.71041	0.71072	0.00017
42.12	0.71118	0.71076	0.00016
42.08	0.71082	0.71069	0.00013
42.04	0.71051	0.71064	0.00016
41.99	0.71071	0.71061	0.00017
41.95	0.71102	0.71064	0.00019
41.91	0.71067	0.71060	0.00017
41.86	0.71067	0.71058	0.00018
41.82	0.71029	0.71059	0.00018
41.78	0.71086	0.71059	0.00018
41.73	0.71086	0.71058	0.00018
41.69	0.71052	0.71057	0.00017
41.64	0.71027	0.71057	0.00017
41.60	0.71023	0.71060	0.00015
41.56	0.71104	0.71062	0.00014
41.51	0.71062	0.71059	0.00011
41.47	0.71042	0.71055	0.00013
41.43	0.71076	0.71057	0.00013
41.38	0.71031	0.71054	0.00012
41.34	0.71080	0.71058	0.00011
41.29	0.71069	0.71061	0.00015
41.25	0.71059	0.71063	0.00017
41.21	0.71057	0.71066	0.00017
41.16	0.71037	0.71062	0.00020
41.12	0.71077	0.71063	0.00020
41.08	0.71022	0.71061	0.00020
41.03	0.71063	0.71063	0.00018
40.99	0.71050	0.71060	0.00019
40.94	0.71064	0.71060	0.00019
40.90	0.71110	0.71058	0.00020
40.86	0.71097	0.71052	0.00016
40.81	0.71089	0.71048	0.00012
40.77	0.71013	0.71044	0.00009
40.73	0.71048	0.71044	0.00009
40.68	0.71051	0.71045	0.00009
40.64	0.71047	0.71045	0.00009
40.59	0.71030	0.71046	0.00009
40.55	0.71056	0.71049	0.00009
40.51	0.71039	0.71048	0.00009
40.46	0.71049	0.71049	0.00009
40.42	0.71061	0.71049	0.00009
40.38	0.71046	0.71048	0.00008
40.33	0.71013	0.71052	0.00011
40.29	0.71058	0.71053	0.00008
40.24	0.71053	0.71050	0.00010
40.20	0.71051	0.71047	0.00011
40.16	0.71058	0.71044	0.00012
40.11	0.71049	0.71045	0.00012

40.07	0.71054	0.71042	0.00013
40.03	0.71046	0.71037	0.00015
39.98	0.71051	0.71040	0.00016
39.94	0.71084	0.71041	0.00017
39.89	0.71030	0.71039	0.00015
39.85	0.71024	0.71037	0.00016
39.81	0.71026	0.71036	0.00017
39.76	0.71022	0.71039	0.00017
39.72	0.71063	0.71040	0.00017
39.68	0.71024	0.71032	0.00019
39.63	0.71003	0.71032	0.00019
39.59	0.71071	0.71040	0.00020
39.54	0.71063	0.71030	0.00022
39.50	0.71064	0.71026	0.00020
39.46	0.71011	0.71018	0.00020
39.41	0.71010	0.71017	0.00020
39.37	0.71061	0.71020	0.00020
39.33	0.71027	0.71013	0.00019
39.28	0.70989	0.71015	0.00019
39.24	0.71024	0.71016	0.00019
39.19	0.71076	0.71015	0.00019
39.15	0.70979	0.71009	0.00013
39.11	0.71019	0.71009	0.00013
39.06	0.70982	0.71011	0.00014
39.02	0.71005	0.71012	0.00014
38.98	0.71035	0.71016	0.00015
38.93	0.70997	0.71017	0.00016
38.89	0.71041	0.71019	0.00016
38.84	0.71002	0.71018	0.00015
38.80	0.71010	0.71024	0.00017
38.76	0.71024	0.71025	0.00017
38.71	0.70978	0.71027	0.00017
38.67	0.71037	0.71034	0.00014
38.63	0.70987	0.71034	0.00014
38.58	0.71044	0.71039	0.00009
38.54	0.71051	0.71040	0.00010
38.49	0.71015	0.71041	0.00011
38.45	0.71035	0.71044	0.00009
38.41	0.71058	0.71046	0.00009
38.36	0.71018	0.71038	0.00016
38.32	0.71045	0.71042	0.00016
38.28	0.71054	0.71040	0.00017
38.23	0.71030	0.71042	0.00018
38.19	0.71037	0.71044	0.00018
38.14	0.71060	0.71045	0.00018
38.10	0.71062	0.71036	0.00022
38.06	0.71036	0.71035	0.00022
38.01	0.71058	0.71035	0.00022
37.97	0.70977	0.71027	0.00024
37.93	0.71061	0.71033	0.00021

37.88	0.71027	0.71028	0.00020
37.84	0.71074	0.71025	0.00021
37.79	0.71052	0.71022	0.00018
37.75	0.71040	0.71021	0.00018
37.71	0.70974	0.71020	0.00018
37.66	0.71048	0.71022	0.00015
37.62	0.71036	0.71020	0.00014
37.58	0.70978	0.71020	0.00014
37.53	0.71040	0.71028	0.00012
37.49	0.71010	0.71028	0.00012
37.44	0.71002	0.71030	0.00012
37.40	0.71038	0.71033	0.00010
37.36	0.71050	0.71028	0.00013
37.31	0.71022	0.71025	0.00013
37.27	0.71001	0.71028	0.00014
37.23	0.71022	0.71032	0.00012
37.18	0.71041	0.71032	0.00013
37.14	0.71054	0.71031	0.00012
37.09	0.71043	0.71026	0.00012
37.05	0.71030	0.71024	0.00012
37.01	0.71030	0.71020	0.00013
36.96	0.70986	0.71021	0.00013
36.92	0.71022	0.71027	0.00012
36.88	0.71052	0.71025	0.00013
36.83	0.71043	0.71021	0.00011
36.79	0.71021	0.71020	0.00011
36.74	0.71031	0.71023	0.00012
36.70	0.71005	0.71019	0.00014
36.66	0.71017	0.71020	0.00013
36.61	0.70997	0.71023	0.00015
36.57	0.71039	0.71027	0.00014
36.53	0.71046	0.71026	0.00013
36.48	0.71002	0.71024	0.00013
36.44	0.71006	0.71027	0.00012
36.39	0.71034	0.71026	0.00013
36.35	0.71049	0.71027	0.00013
36.31	0.70992	0.71023	0.00013
36.26	0.71015	0.71026	0.00011
36.22	0.71052	0.71024	0.00012
36.18	0.71034	0.71019	0.00012
36.13	0.71030	0.71019	0.00012
36.09	0.71026	0.71020	0.00013
36.04	0.71037	0.71018	0.00013
36.00	0.70993	0.71011	0.00015
35.96	0.71045	0.71010	0.00016
35.91	0.71008	0.71005	0.00014
35.87	0.71022	0.71007	0.00015
35.83	0.70997	0.71008	0.00016
35.78	0.70998	0.71013	0.00017
35.74	0.71030	0.71014	0.00017

35.69	0.71044	0.71011	0.00017
35.65	0.71005	0.71014	0.00020
35.61	0.70969	0.71014	0.00020
35.56	0.70985	0.71021	0.00018
35.52	0.70988	0.71023	0.00017
35.48	0.71033	0.71029	0.00016
35.43	0.71032	0.71030	0.00016
35.39	0.71044	0.71027	0.00016
35.34	0.71013	0.71027	0.00016
35.30	0.70992	0.71030	0.00016
35.26	0.71074	0.71039	0.00016
35.21	0.71006	0.71031	0.00015
35.17	0.71038	0.71031	0.00016
35.13	0.71007	0.71028	0.00016
35.08	0.71050	0.71029	0.00016
35.04	0.71041	0.71029	0.00016
34.99	0.71008	0.71028	0.00016
34.95	0.71042	0.71031	0.00015
34.91	0.71044	0.71032	0.00015
34.86	0.71075	0.71026	0.00017
34.82	0.71002	0.71020	0.00014
34.78	0.71004	0.71021	0.00013
34.73	0.71012	0.71022	0.00013
34.69	0.71009	0.71022	0.00013
34.64	0.71056	0.71026	0.00013
34.60	0.71032	0.71023	0.00012
34.56	0.71036	0.71022	0.00012
34.51	0.71046	0.71016	0.00014
34.47	0.70989	0.71010	0.00014
34.43	0.71016	0.71016	0.00015
34.38	0.71011	0.71015	0.00015
34.34	0.71013	0.71011	0.00017
34.29	0.71014	0.71013	0.00018
34.25	0.71051	0.71013	0.00018
34.21	0.71020	0.71009	0.00016
34.16	0.71025	0.71008	0.00016
34.12	0.70976	0.71008	0.00016
34.08	0.70987	0.71006	0.00017
34.03	0.71048	0.71010	0.00017
33.99	0.71004	0.71012	0.00019
33.94	0.70971	0.71013	0.00019
33.90	0.71037	0.71018	0.00016
33.86	0.71014	0.71014	0.00016
33.81	0.71012	0.71015	0.00016
33.77	0.71008	0.71019	0.00018
33.73	0.71023	0.71020	0.00017
33.68	0.70961	0.71021	0.00017
33.64	0.71027	0.71027	0.00011
33.59	0.71062	0.71029	0.00012
33.55	0.71016	0.71026	0.00010

33.51	0.71017	0.71026	0.00010
33.46	0.70997	0.71025	0.00011
33.42	0.71029	0.71027	0.00009
33.38	0.71051	0.71027	0.00009
33.33	0.71021	0.71025	0.00007
33.29	0.71027	0.71022	0.00010
33.24	0.71020	0.71021	0.00010
33.20	0.71048	0.71020	0.00010
33.16	0.71037	0.71017	0.00008
33.11	0.71011	0.71014	0.00007
33.07	0.71009	0.71017	0.00008
33.03	0.71019	0.71017	0.00008
32.98	0.71032	0.71017	0.00008
32.94	0.71028	0.71016	0.00007
32.89	0.70993	0.71014	0.00007
32.85	0.71011	0.71014	0.00007
32.81	0.71012	0.71016	0.00008
32.76	0.71021	0.71015	0.00008
32.72	0.71009	0.71013	0.00009
32.68	0.71032	0.71014	0.00009
32.63	0.71012	0.71015	0.00009
32.59	0.71022	0.71018	0.00011
32.54	0.71019	0.71022	0.00014
32.50	0.71013	0.71023	0.00014
32.46	0.70988	0.71022	0.00014
32.41	0.71031	0.71021	0.00015
32.37	0.71007	0.71018	0.00016
32.33	0.70996	0.71020	0.00015
32.28	0.71022	0.71024	0.00015
32.24	0.71037	0.71025	0.00015
32.19	0.71044	0.71025	0.00015
32.15	0.71057	0.71024	0.00015
32.11	0.71032	0.71019	0.00013
32.06	0.71004	0.71020	0.00013
32.02	0.70985	0.71021	0.00013
31.98	0.70992	0.71024	0.00010
31.93	0.71028	0.71029	0.00007
31.89	0.71038	0.71031	0.00008
31.84	0.71029	0.71032	0.00009
31.80	0.71037	0.71031	0.00009
31.76	0.71038	0.71029	0.00011
31.71	0.71010	0.71028	0.00010
31.67	0.71039	0.71026	0.00012
31.63	0.71013	0.71022	0.00013
31.58	0.71017	0.71022	0.00013
31.54	0.71042	0.71021	0.00013
31.49	0.71044	0.71019	0.00012
31.45	0.71054	0.71013	0.00013
31.41	0.71022	0.71006	0.00010
31.36	0.71007	0.71005	0.00009

31.32	0.71032	0.71006	0.00010
31.28	0.70990	0.71004	0.00008
31.23	0.71004	0.71006	0.00007
31.19	0.71006	0.71009	0.00009
31.14	0.71015	0.71010	0.00009
31.10	0.71013	0.71008	0.00010
31.06	0.70984	0.71007	0.00010
31.01	0.70987	0.71012	0.00009
30.97	0.71009	0.71011	0.00009
30.93	0.71019	0.71014	0.00010
30.88	0.71012	0.71012	0.00010
30.84	0.71011	0.71014	0.00011
30.79	0.71033	0.71012	0.00011
30.75	0.71019	0.71010	0.00011
30.71	0.70991	0.71012	0.00012
30.66	0.71009	0.71012	0.00013
30.62	0.71028	0.71010	0.00013
30.58	0.70984	0.71007	0.00013
30.53	0.71031	0.71006	0.00013
30.49	0.71004	0.71008	0.00015
30.44	0.71025	0.71008	0.00015
30.40	0.70992	0.71004	0.00015
30.36	0.71021	0.71004	0.00015
30.31	0.71039	0.71000	0.00015
30.27	0.70984	0.70999	0.00013
30.23	0.70996	0.71000	0.00013
30.18	0.70992	0.71004	0.00016
30.14	0.70977	0.71007	0.00016
30.09	0.71046	0.71012	0.00015
30.05	0.71006	0.71010	0.00013
30.01	0.70989	0.71010	0.00013
29.96	0.70990	0.71011	0.00013
29.92	0.70985	0.71012	0.00012
29.88	0.71026	0.71011	0.00013
29.83	0.70989	0.71009	0.00013
29.79	0.71043	0.71012	0.00012
29.75	0.71023	0.71007	0.00011
29.70	0.71022	0.71005	0.00010
29.66	0.71029	0.71005	0.00010
29.61	0.71008	0.70999	0.00010
29.57	0.70993	0.70999	0.00010
29.53	0.71000	0.70997	0.00010
29.48	0.70978	0.70995	0.00011
29.44	0.71003	0.70996	0.00010
29.40	0.71025	0.70997	0.00011
29.35	0.70994	0.70997	0.00011
29.31	0.71003	0.70998	0.00011
29.26	0.71014	0.70997	0.00011
29.22	0.70973	0.70993	0.00011
29.18	0.71002	0.70995	0.00010

29.13	0.70980	0.70996	0.00011
29.09	0.70980	0.70999	0.00011
29.05	0.70989	0.71000	0.00010
29.00	0.71008	0.71003	0.00010
28.96	0.71029	0.71003	0.00010
28.91	0.71000	0.70999	0.00008
28.87	0.70998	0.70999	0.00008
28.83	0.70971	0.70998	0.00008
28.78	0.70994	0.71002	0.00006
28.74	0.71014	0.70999	0.00010
28.70	0.71004	0.71002	0.00013
28.65	0.70997	0.71005	0.00015
28.61	0.71018	0.71003	0.00016
28.56	0.71003	0.71005	0.00017
28.52	0.70992	0.71010	0.00019
28.48	0.70999	0.71011	0.00019
28.43	0.70989	0.71011	0.00019
28.39	0.71011	0.71009	0.00021
28.35	0.70964	0.71010	0.00021
28.30	0.71045	0.71015	0.00018
28.26	0.71035	0.71016	0.00019
28.21	0.70978	0.71013	0.00018
28.17	0.71039	0.71019	0.00017
28.13	0.71052	0.71017	0.00017
28.08	0.71000	0.71011	0.00015
28.04	0.71001	0.71011	0.00015
28.00	0.70967	0.71010	0.00016
27.95	0.71021	0.71015	0.00012
27.91	0.71009	0.71014	0.00012
27.86	0.71053	0.71010	0.00016
27.82	0.71006	0.71006	0.00013
27.78	0.71041	0.71005	0.00013
27.73	0.71017	0.70997	0.00013
27.69	0.70999	0.70994	0.00012
27.65	0.70993	0.70995	0.00012
27.60	0.70995	0.70997	0.00013
27.56	0.71011	0.70997	0.00013
27.51	0.71018	0.70991	0.00016
27.47	0.70964	0.70989	0.00014
27.43	0.71017	0.70993	0.00013
27.38	0.70994	0.70990	0.00012
27.34	0.70961	0.70995	0.00016
27.30	0.70992	0.70998	0.00015
27.25	0.71004	0.70998	0.00015
27.21	0.71014	0.71005	0.00021
27.16	0.70999	0.71005	0.00021
27.12	0.70951	0.71003	0.00022
27.08	0.70998	0.71009	0.00019
27.03	0.71001	0.71013	0.00019
26.99	0.70990	0.71014	0.00019

26.95	0.71044	0.71014	0.00019
26.90	0.70988	0.71011	0.00017
26.86	0.70991	0.71011	0.00017
26.81	0.71075	0.71011	0.00017
26.77	0.71017	0.71008	0.00012
26.73	0.70979	0.71008	0.00012
26.68	0.71011	0.71008	0.00012
26.64	0.71032	0.71005	0.00013
26.60	0.71011	0.71001	0.00011
26.55	0.70996	0.70997	0.00012
26.51	0.71005	0.70995	0.00013
26.46	0.70993	0.70995	0.00013
26.42	0.70994	0.70993	0.00013
26.38	0.71038	0.70988	0.00016
26.33	0.71019	0.70986	0.00013
26.29	0.70978	0.70983	0.00011
26.25	0.70983	0.70983	0.00011
26.20	0.70989	0.70982	0.00011
26.16	0.70973	0.70983	0.00012
26.11	0.70981	0.70985	0.00012
26.07	0.71003	0.70984	0.00012
26.03	0.70974	0.70980	0.00012
25.98	0.70947	0.70984	0.00014
25.94	0.71011	0.70985	0.00013
25.90	0.70993	0.70982	0.00011
25.85	0.70978	0.70984	0.00012
25.81	0.70970	0.70987	0.00013
25.76	0.71003	0.70990	0.00013
25.72	0.70987	0.70988	0.00013
25.68	0.70980	0.70990	0.00013
25.63	0.70959	0.70988	0.00015
25.59	0.71014	0.70991	0.00013
25.55	0.70959	0.70987	0.00012
25.50	0.70981	0.70986	0.00013
25.46	0.71009	0.70990	0.00014
25.41	0.71006	0.70988	0.00014
25.37	0.71002	0.70983	0.00014
25.33	0.70979	0.70980	0.00014
25.28	0.71010	0.70977	0.00015
25.24	0.70958	0.70978	0.00015
25.20	0.70990	0.70980	0.00014
25.15	0.70975	0.70976	0.00015
25.11	0.70953	0.70977	0.00015
25.06	0.71016	0.70978	0.00014
25.02	0.70992	0.70972	0.00012
24.98	0.70958	0.70975	0.00015
24.93	0.70964	0.70977	0.00015
24.89	0.70958	0.70981	0.00015
24.85	0.71012	0.70982	0.00015
24.80	0.70979	0.70979	0.00013

24.76	0.70956	0.70976	0.00014
24.71	0.70979	0.70975	0.00015
24.67	0.70968	0.70975	0.00015
24.63	0.70953	0.70974	0.00016
24.58	0.71021	0.70976	0.00015
24.54	0.70976	0.70972	0.00011
24.50	0.71007	0.70968	0.00013
24.45	0.70968	0.70966	0.00010
24.41	0.70979	0.70965	0.00010
24.36	0.70956	0.70965	0.00010
24.32	0.70941	0.70969	0.00012
24.28	0.70982	0.70969	0.00013
24.23	0.70959	0.70970	0.00013
24.19	0.70970	0.70976	0.00017
24.15	0.70977	0.70977	0.00017
24.10	0.70942	0.70977	0.00017
24.06	0.70983	0.70979	0.00015
24.01	0.70965	0.70980	0.00015
23.97	0.70974	0.70981	0.00015
23.93	0.71001	0.70980	0.00015
23.88	0.70933	0.70981	0.00016
23.84	0.70992	0.70988	0.00012
23.80	0.71025	0.70985	0.00013
23.75	0.70976	0.70980	0.00009
23.71	0.70974	0.70979	0.00010
23.66	0.70971	0.70979	0.00010
23.62	0.70992	0.70979	0.00010
23.58	0.70970	0.70978	0.00011
23.53	0.70967	0.70979	0.00011
23.49	0.71012	0.70981	0.00012
23.45	0.70996	0.70975	0.00008
23.40	0.70971	0.70969	0.00003
23.36	0.70970	0.70969	0.00004
23.31	0.70962	0.70968	0.00006
23.27	0.70974	0.70974	0.00003
22.48	0.70903	0.70963	0.00020
22.44	0.70972	0.70968	0.00014
22.40	0.70987	0.70966	0.00015
22.35	0.70973	0.70964	0.00015
22.31	0.71000	0.70965	0.00015
22.26	0.70954	0.70955	0.00016
22.22	0.70996	0.70955	0.00016
22.18	0.70974	0.70950	0.00014
22.13	0.70940	0.70947	0.00013
22.09	0.70930	0.70951	0.00014
22.05	0.70959	0.70959	0.00017
22.00	0.70943	0.70958	0.00018
21.96	0.70972	0.70958	0.00018
21.91	0.70978	0.70955	0.00017
21.87	0.70908	0.70951	0.00017

21.83	0.70955	0.70956	0.00014
21.78	0.70938	0.70963	0.00020
21.74	0.70952	0.70967	0.00020
21.70	0.70979	0.70966	0.00021
21.65	0.71009	0.70964	0.00020
21.61	0.70945	0.70960	0.00018
21.56	0.70947	0.70965	0.00018
21.52	0.70942	0.70966	0.00018
21.48	0.70942	0.70966	0.00018
21.43	0.70948	0.70966	0.00018
21.39	0.71031	0.70969	0.00018
21.35	0.70978	0.70958	0.00014
21.30	0.70937	0.70954	0.00014
21.26	0.70960	0.70960	0.00015
21.21	0.70971	0.70965	0.00019
21.17	0.70989	0.70968	0.00020
21.13	0.70963	0.70964	0.00020
21.08	0.70935	0.70964	0.00020
21.04	0.70948	0.70971	0.00020
21.00	0.70976	0.70975	0.00020
20.95	0.70919	0.70974	0.00020
20.91	0.70940	0.70977	0.00016
20.86	0.70994	0.70985	0.00016
20.82	0.71013	0.70985	0.00016
20.78	0.71003	0.70984	0.00015
20.73	0.70950	0.70982	0.00014
20.69	0.70963	0.70982	0.00014
20.65	0.71007	0.70980	0.00015
20.60	0.70982	0.70976	0.00015
20.56	0.70971	0.70969	0.00020
20.51	0.70952	0.70967	0.00020
20.47	0.71019	0.70967	0.00020
20.43	0.70991	0.70963	0.00017
20.38	0.70999	0.70962	0.00016
20.34	0.70983	0.70959	0.00014
20.30	0.70957	0.70954	0.00014
20.25	0.70944	0.70954	0.00014
20.21	0.70962	0.70960	0.00016
20.16	0.70909	0.70960	0.00016
20.12	0.70952	0.70967	0.00012
20.08	0.70952	0.70971	0.00012
20.03	0.70982	0.70975	0.00013
19.99	0.70979	0.70970	0.00016
19.95	0.70970	0.70970	0.00016
19.90	0.70936	0.70965	0.00019
19.86	0.70957	0.70975	0.00021
19.81	0.71000	0.70978	0.00021
19.77	0.70959	0.70973	0.00022
19.73	0.70982	0.70976	0.00022
19.68	0.70990	0.70976	0.00022

19.64	0.71000	0.70971	0.00023
19.60	0.70925	0.70965	0.00023
19.55	0.70980	0.70970	0.00021
19.51	0.70924	0.70973	0.00023
19.46	0.71031	0.70975	0.00022
19.42	0.70994	0.70971	0.00018
19.38	0.70943	0.70967	0.00018
19.33	0.70995	0.70965	0.00020
19.29	0.70984	0.70963	0.00019
19.25	0.70940	0.70958	0.00019
19.20	0.70933	0.70960	0.00018
19.16	0.70976	0.70964	0.00018
19.11	0.71013	0.70966	0.00019
19.07	0.70940	0.70958	0.00016
19.03	0.70990	0.70963	0.00018
18.98	0.70961	0.70964	0.00018
18.94	0.70919	0.70968	0.00020
18.90	0.70974	0.70973	0.00016
18.85	0.70935	0.70975	0.00017
18.81	0.70955	0.70984	0.00018
18.76	0.70973	0.70986	0.00017
18.72	0.70997	0.70990	0.00018
18.68	0.70931	0.70985	0.00020
18.63	0.70997	0.70991	0.00016
18.59	0.71000	0.70983	0.00022
18.55	0.70999	0.70978	0.00022
18.50	0.70965	0.70976	0.00022
18.46	0.70993	0.70978	0.00022
18.41	0.71034	0.70973	0.00022
18.37	0.70974	0.70966	0.00018
18.33	0.71013	0.70963	0.00018
18.28	0.70941	0.70963	0.00018
18.24	0.70992	0.70966	0.00017
18.20	0.70919	0.70966	0.00017
18.15	0.70947	0.70973	0.00014
18.11	0.70982	0.70973	0.00014
18.07	0.70987	0.70974	0.00015
18.02	0.70947	0.70977	0.00016
17.98	0.70962	0.70982	0.00015
17.93	0.70945	0.70985	0.00015
17.89	0.71008	0.70987	0.00012
17.85	0.70974	0.70984	0.00012
17.80	0.70987	0.70980	0.00016
17.76	0.70993	0.70981	0.00016
17.72	0.70946	0.70978	0.00016
17.67	0.70995	0.70980	0.00015
17.63	0.71008	0.70981	0.00016
17.58	0.71001	0.70976	0.00015
17.54	0.70995	0.70974	0.00014
17.50	0.70966	0.70975	0.00016

17.45	0.70976	0.70973	0.00017
17.41	0.70933	0.70971	0.00017
17.37	0.70997	0.70979	0.00017
17.32	0.70963	0.70977	0.00016
17.28	0.70962	0.70979	0.00016
17.23	0.71010	0.70981	0.00016
17.19	0.70954	0.70981	0.00016
17.15	0.70979	0.70983	0.00014
17.10	0.71012	0.70984	0.00014
17.06	0.70942	0.70980	0.00013
17.02	0.70957	0.70985	0.00010
16.97	0.71013	0.70984	0.00012
16.93	0.70982	0.70980	0.00010
16.88	0.70980	0.70981	0.00011
16.84	0.70986	0.70985	0.00013
16.80	0.71007	0.70982	0.00014
16.75	0.70977	0.70981	0.00013
16.71	0.70986	0.70981	0.00013
16.67	0.70971	0.70980	0.00013
16.62	0.70993	0.70983	0.00013
16.58	0.70943	0.70983	0.00013
16.53	0.70977	0.70984	0.00012
16.49	0.70990	0.70988	0.00014
16.45	0.71018	0.70990	0.00015
16.40	0.70963	0.70982	0.00018
16.36	0.70992	0.70991	0.00023
16.32	0.70979	0.70988	0.00024
16.27	0.70979	0.70995	0.00026
16.23	0.70998	0.70996	0.00026
16.18	0.70993	0.70991	0.00028
16.14	0.70949	0.70987	0.00029
16.10	0.71017	0.70991	0.00027
16.05	0.71018	0.70986	0.00027
16.01	0.70928	0.70983	0.00027
15.97	0.71060	0.70991	0.00024
15.92	0.70962	0.70986	0.00019
15.88	0.71045	0.70989	0.00019
15.83	0.70990	0.70981	0.00014
15.79	0.70944	0.70978	0.00015
15.75	0.70960	0.70981	0.00013
15.70	0.70989	0.70984	0.00012
15.66	0.70960	0.70988	0.00015
15.62	0.70989	0.70988	0.00016
15.57	0.71010	0.70985	0.00017
15.53	0.71013	0.70981	0.00016
15.48	0.70991	0.70980	0.00015
15.44	0.70967	0.70983	0.00018
15.40	0.70962	0.70985	0.00017
15.35	0.70966	0.70987	0.00017
15.31	0.70998	0.70986	0.00017

15.27	0.71029	0.70982	0.00018
15.22	0.70954	0.70975	0.00015
15.18	0.70963	0.70976	0.00014
15.13	0.70967	0.70978	0.00014
15.09	0.71005	0.70975	0.00016
15.05	0.71024	0.70969	0.00016
15.00	0.70982	0.70961	0.00011
14.96	0.70978	0.70962	0.00012
14.92	0.70963	0.70965	0.00016
14.87	0.70953	0.70970	0.00019
14.83	0.70964	0.70977	0.00021
14.78	0.70962	0.70984	0.00023
14.74	0.70985	0.70983	0.00023
14.70	0.70936	0.70984	0.00023
14.65	0.70940	0.70988	0.00020
14.61	0.70946	0.70989	0.00019
14.57	0.70990	0.70989	0.00019
14.52	0.71015	0.70988	0.00019
14.48	0.71015	0.70982	0.00019
14.43	0.71021	0.70981	0.00018
14.39	0.71029	0.70973	0.00018
14.35	0.70958	0.70970	0.00014
14.30	0.70988	0.70971	0.00014
14.26	0.70977	0.70973	0.00015
14.22	0.70949	0.70974	0.00015
14.17	0.70950	0.70978	0.00015
14.13	0.70979	0.70979	0.00014
14.08	0.70958	0.70980	0.00014
14.04	0.71001	0.70986	0.00014
14.00	0.70938	0.70985	0.00013
13.95	0.70999	0.70992	0.00009
13.91	0.70976	0.70989	0.00010
13.87	0.71007	0.70994	0.00011
13.82	0.70982	0.70991	0.00012
13.78	0.70990	0.70990	0.00012
13.73	0.70964	0.70992	0.00013
13.69	0.70991	0.70991	0.00014
13.65	0.71009	0.70992	0.00014
13.60	0.70992	0.70991	0.00013
13.56	0.71009	0.70992	0.00014
13.52	0.70973	0.70991	0.00013
13.47	0.71023	0.70998	0.00015
13.43	0.70978	0.70990	0.00017
13.38	0.70969	0.70990	0.00017
13.34	0.71011	0.70992	0.00016
13.30	0.70958	0.70990	0.00016
13.25	0.70998	0.70990	0.00016
13.21	0.70997	0.70991	0.00016
13.17	0.71007	0.70992	0.00016
13.12	0.70998	0.70991	0.00016

13.08	0.71039	0.70987	0.00017
13.03	0.70951	0.70978	0.00014
12.99	0.70975	0.70986	0.00016
12.95	0.70989	0.70986	0.00016
12.90	0.70989	0.70984	0.00016
12.86	0.70961	0.70984	0.00016
12.82	0.71002	0.70985	0.00016
12.77	0.71010	0.70985	0.00016
12.73	0.70995	0.70985	0.00016
12.68	0.70964	0.70984	0.00016
12.64	0.70949	0.70989	0.00016
12.60	0.71030	0.70992	0.00014
12.55	0.70974	0.70992	0.00014
12.51	0.70964	0.70999	0.00016
12.47	0.70995	0.71001	0.00015
12.42	0.70971	0.71004	0.00015
12.38	0.71001	0.71005	0.00014
12.33	0.71007	0.71010	0.00017
12.29	0.70982	0.71007	0.00018
12.25	0.71014	0.71003	0.00021
12.20	0.70976	0.71004	0.00022
12.16	0.71034	0.71003	0.00022
12.12	0.71041	0.70997	0.00021
12.07	0.70987	0.70991	0.00019
12.03	0.71023	0.70999	0.00023
11.98	0.70986	0.70995	0.00023
11.94	0.71049	0.71001	0.00025
11.90	0.70977	0.71000	0.00024
11.85	0.70945	0.71005	0.00024
11.81	0.71019	0.71015	0.00021
11.77	0.70974	0.71009	0.00024
11.72	0.70971	0.71013	0.00022
11.68	0.70982	0.71017	0.00020
11.63	0.71059	0.71015	0.00022
11.59	0.70990	0.71007	0.00021
11.55	0.71047	0.71001	0.00027
11.50	0.71031	0.70998	0.00025
11.46	0.71035	0.70998	0.00025
11.42	0.71046	0.70996	0.00024
11.37	0.70959	0.70989	0.00022
11.33	0.71011	0.70988	0.00022
11.28	0.71011	0.70992	0.00026
11.24	0.70962	0.70992	0.00026
11.20	0.70979	0.70997	0.00025
11.15	0.70925	0.70996	0.00026
11.11	0.71022	0.71005	0.00021
11.07	0.71028	0.70999	0.00022
11.02	0.71016	0.70996	0.00022
10.98	0.70975	0.70994	0.00021
10.93	0.70947	0.70998	0.00021

10.89	0.71056	0.71006	0.00018
10.85	0.71015	0.71002	0.00015
10.80	0.71011	0.71000	0.00014
10.76	0.70960	0.70997	0.00015
10.72	0.71018	0.70999	0.00013
10.67	0.70962	0.70995	0.00012
10.63	0.70999	0.71004	0.00014
10.58	0.71000	0.71003	0.00015
10.54	0.71016	0.71004	0.00015
10.50	0.71027	0.71004	0.00015
10.45	0.71011	0.70997	0.00017
10.41	0.70996	0.70998	0.00018
10.37	0.70978	0.70997	0.00018
10.32	0.70981	0.71001	0.00018
10.28	0.70985	0.70999	0.00018
10.23	0.71050	0.71001	0.00018
10.19	0.70987	0.70995	0.00014
10.15	0.71011	0.70993	0.00016
10.10	0.71017	0.70996	0.00019
10.06	0.70950	0.70993	0.00018
10.02	0.71023	0.70996	0.00016
9.97	0.70990	0.70995	0.00016
9.93	0.71011	0.70993	0.00016
9.88	0.70968	0.70994	0.00017
9.84	0.71006	0.70999	0.00016
9.80	0.70989	0.70998	0.00016
9.75	0.70962	0.70999	0.00016
9.71	0.71046	0.71006	0.00015
9.67	0.70988	0.71003	0.00012
9.62	0.70973	0.71005	0.00012
9.58	0.71012	0.71007	0.00010
9.53	0.70974	0.71008	0.00011
9.49	0.71020	0.71013	0.00008
9.45	0.71018	0.71014	0.00009
9.40	0.70996	0.71011	0.00011
9.36	0.71000	0.71011	0.00011
9.32	0.71029	0.71011	0.00011
9.27	0.71018	0.71010	0.00010
9.23	0.71009	0.71006	0.00013
9.18	0.70989	0.71007	0.00013
9.14	0.71027	0.71010	0.00012
9.10	0.71020	0.71002	0.00017
9.05	0.71034	0.71001	0.00016
9.01	0.70987	0.70996	0.00014
8.97	0.70995	0.70993	0.00017
8.92	0.71003	0.70993	0.00017
8.88	0.71021	0.70990	0.00017
8.83	0.70973	0.70990	0.00018
8.79	0.71024	0.70992	0.00017
8.75	0.71015	0.70993	0.00018

8.70	0.70950	0.70994	0.00019
8.66	0.71007	0.70997	0.00016
8.62	0.70989	0.71004	0.00022
8.57	0.70951	0.70999	0.00025
8.53	0.70995	0.71004	0.00022
8.48	0.70973	0.71005	0.00022
8.44	0.71025	0.71006	0.00022
8.40	0.70994	0.71003	0.00022
8.35	0.71036	0.71001	0.00022
8.31	0.71021	0.70999	0.00021
8.27	0.70985	0.70997	0.00021
8.22	0.71071	0.71003	0.00022
8.18	0.70944	0.70992	0.00018
8.13	0.70994	0.70999	0.00014
8.09	0.71008	0.71002	0.00015
8.05	0.70983	0.71001	0.00015
8.00	0.70994	0.71001	0.00015
7.96	0.70975	0.71004	0.00015
7.92	0.71011	0.71006	0.00014
7.87	0.71010	0.71005	0.00014
7.83	0.71041	0.71004	0.00014
7.78	0.70962	0.70995	0.00014
7.74	0.71010	0.70998	0.00012
7.70	0.71023	0.70994	0.00013
7.65	0.71006	0.70990	0.00012
7.61	0.70981	0.70992	0.00013
7.57	0.71017	0.70991	0.00014
7.52	0.70998	0.70988	0.00013
7.48	0.71006	0.70990	0.00014
7.43	0.70990	0.70988	0.00014
7.39	0.70956	0.70991	0.00016
7.35	0.70993	0.70994	0.00014
7.30	0.70969	0.70994	0.00014
7.26	0.70987	0.70996	0.00013
7.22	0.71025	0.70998	0.00013
7.17	0.70966	0.70998	0.00013
7.13	0.70990	0.71000	0.00011
7.08	0.71021	0.71002	0.00011
7.04	0.70978	0.70996	0.00013
7.00	0.71025	0.70995	0.00013
6.95	0.70987	0.70988	0.00014
6.91	0.70993	0.70989	0.00014
6.87	0.70987	0.70987	0.00014
6.82	0.71011	0.70986	0.00014
6.78	0.71025	0.70986	0.00014
6.73	0.70986	0.70982	0.00011
6.69	0.71008	0.70980	0.00011
6.65	0.70964	0.70978	0.00009
6.60	0.70968	0.70979	0.00009
6.56	0.70957	0.70979	0.00008

6.52	0.70990	0.70981	0.00007
6.47	0.70975	0.70982	0.00008
6.43	0.70979	0.70983	0.00008
6.38	0.71007	0.70983	0.00008
6.34	0.70983	0.70984	0.00010
6.30	0.70973	0.70979	0.00014
6.25	0.70982	0.70982	0.00015
6.21	0.70972	0.70980	0.00016
6.17	0.70970	0.70984	0.00017
6.12	0.70979	0.70978	0.00023
6.08	0.70999	0.70979	0.00024
6.04	0.70983	0.70981	0.00025
5.99	0.70978	0.70982	0.00025
5.95	0.71021	0.70981	0.00025
5.90	0.70931	0.70982	0.00025
5.86	0.71002	0.70986	0.00023
5.82	0.70962	0.70984	0.00022
5.77	0.71017	0.70982	0.00023
5.73	0.70905	0.70980	0.00022
5.69	0.70995	0.70992	0.00016
5.64	0.71019	0.70991	0.00016
5.60	0.70988	0.70989	0.00015
5.55	0.70974	0.70990	0.00016
5.51	0.71026	0.70988	0.00017
5.47	0.70973	0.70982	0.00016
5.42	0.70977	0.70984	0.00016
5.38	0.70948	0.70984	0.00016
5.34	0.70992	0.70990	0.00014
5.29	0.71029	0.70990	0.00014
5.25	0.70989	0.70984	0.00012
5.20	0.70990	0.70987	0.00014
5.16	0.71004	0.70980	0.00019
5.12	0.70949	0.70978	0.00018
5.07	0.70967	0.70983	0.00017
5.03	0.70996	0.70988	0.00018
4.99	0.70977	0.70986	0.00018
4.94	0.71011	0.70991	0.00020
4.90	0.70990	0.70988	0.00019
4.85	0.70965	0.70992	0.00021
4.81	0.71021	0.70999	0.00021
4.77	0.70924	0.70993	0.00021
4.72	0.70984	0.71003	0.00015
4.68	0.70998	0.71005	0.00015
4.64	0.71011	0.71001	0.00017
4.59	0.70977	0.70997	0.00018
4.55	0.71030	0.71000	0.00018
4.50	0.70983	0.70997	0.00016
4.46	0.71029	0.70998	0.00016
4.42	0.71031	0.70993	0.00015
4.37	0.70967	0.70993	0.00015

4.33	0.71023	0.70995	0.00014
4.29	0.70996	0.70992	0.00013
4.24	0.70964	0.70993	0.00013
4.20	0.70968	0.70999	0.00012
4.15	0.71006	0.71003	0.00010
4.11	0.71002	0.71006	0.00012
4.07	0.70998	0.71003	0.00014
4.02	0.70976	0.71006	0.00015
3.98	0.71032	0.71008	0.00014
3.94	0.70987	0.71006	0.00013
3.89	0.70995	0.71006	0.00013
3.85	0.71004	0.71006	0.00013
3.80	0.71020	0.71009	0.00013
3.76	0.71014	0.71005	0.00014
3.72	0.71034	0.71006	0.00014
3.67	0.70970	0.70998	0.00015
3.63	0.71032	0.70999	0.00015
3.59	0.70994	0.70996	0.00013
3.54	0.71010	0.70998	0.00013
3.50	0.70986	0.70995	0.00013
3.45	0.70999	0.70998	0.00013
3.41	0.71027	0.70997	0.00014
3.37	0.70983	0.70988	0.00015
3.32	0.71021	0.70988	0.00015
3.28	0.70961	0.70981	0.00015
3.24	0.70979	0.70985	0.00015
3.19	0.71000	0.70987	0.00015
3.15	0.71011	0.70987	0.00015
3.10	0.70981	0.70988	0.00016
3.06	0.71016	0.70995	0.00020
3.02	0.70986	0.70991	0.00020
2.97	0.70945	0.70989	0.00020
2.93	0.70984	0.70993	0.00018
2.89	0.70949	0.70997	0.00018
2.84	0.70993	0.71001	0.00016
2.80	0.71009	0.71003	0.00016
2.75	0.70996	0.71002	0.00016
2.71	0.71024	0.71001	0.00016
2.67	0.71047	0.71000	0.00015
2.62	0.70975	0.70993	0.00012
2.58	0.70969	0.70997	0.00012
2.54	0.70987	0.71005	0.00013
2.49	0.71023	0.71006	0.00013
2.45	0.70983	0.71002	0.00013
2.40	0.71013	0.71004	0.00012
2.36	0.70999	0.71003	0.00012
2.32	0.70992	0.71003	0.00012
2.27	0.71017	0.71003	0.00012
2.23	0.70976	0.71001	0.00012
2.19	0.71015	0.71001	0.00012

2.14	0.71045	0.70996	0.00013
2.10	0.71002	0.70995	0.00011
2.05	0.70983	0.70996	0.00012
2.01	0.71003	0.71002	0.00014
1.97	0.70996	0.70999	0.00014
1.92	0.71002	0.71001	0.00015
1.88	0.70993	0.71000	0.00015
1.84	0.70991	0.71001	0.00015
1.79	0.70980	0.71004	0.00015
1.75	0.70967	0.71009	0.00015
1.70	0.71031	0.71018	0.00014
1.66	0.71015	0.71014	0.00014
1.62	0.71037	0.71016	0.00015
1.57	0.70978	0.71013	0.00014
1.53	0.71013	0.71013	0.00014
1.49	0.70995	0.71014	0.00014
1.44	0.71003	0.71017	0.00014
1.40	0.71020	0.71017	0.00014
1.35	0.71028	0.71019	0.00014
1.31	0.71053	0.71018	0.00014
1.27	0.70995	0.71015	0.00012
1.22	0.71036	0.71016	0.00012
1.18	0.71010	0.71018	0.00014
1.14	0.70976	0.71019	0.00014
1.09	0.71023	0.71024	0.00010
1.05	0.71023	0.71020	0.00012
1.00	0.71003	0.71022	0.00013
0.96	0.71037	0.71023	0.00013
0.92	0.71027	0.71022	0.00013
0.87	0.71020	0.71019	0.00014
0.83	0.71004	0.71018	0.00014
0.79	0.71059	0.71019	0.00014
0.74	0.71021	0.71012	0.00012
0.70	0.71022	0.71009	0.00013
0.65	0.70989	0.71005	0.00013
0.61	0.71040	0.71005	0.00013
0.57	0.71006	0.71001	0.00011
0.52	0.71036	0.70998	0.00011
0.48	0.70992	0.70994	0.00008
0.44	0.71015	0.70994	0.00009
0.39	0.71013	0.70991	0.00007
0.35	0.70988	0.70988	0.00004
0.30	0.70984	0.70988	0.00005
0.26	0.70991	0.70989	0.00005
0.22	0.70983	0.70988	0.00006
0.17	0.70999	0.70990	0.00008
0.13	0.70982		

ARB 43.2.1 (M2)

Distance from cervix (mm)	$^{87}Sr/^{86}Sr$	10 point mov. average	2 SE on mov. average
46.50	0.70863	0.70867	0.00013
46.46	0.70907	0.70867	0.00013
46.42	0.70874	0.70862	0.00009
46.37	0.70854	0.70860	0.00009
46.33	0.70831	0.70860	0.00009
46.29	0.70860	0.70861	0.00007
46.24	0.70872	0.70862	0.00008
46.20	0.70877	0.70861	0.00007
46.16	0.70879	0.70859	0.00006
46.11	0.70855	0.70858	0.00005
46.07	0.70858	0.70858	0.00005
46.03	0.70861	0.70857	0.00006
45.98	0.70848	0.70857	0.00006
45.94	0.70859	0.70858	0.00005
45.90	0.70843	0.70856	0.00006
45.85	0.70869	0.70859	0.00006
45.81	0.70859	0.70855	0.00008
45.77	0.70856	0.70855	0.00008
45.72	0.70867	0.70851	0.00010
45.68	0.70860	0.70851	0.00010
45.64	0.70845	0.70850	0.00010
45.59	0.70865	0.70848	0.00011
45.55	0.70856	0.70844	0.00012
45.51	0.70843	0.70846	0.00013
45.47	0.70865	0.70850	0.00015
45.42	0.70830	0.70848	0.00015
45.38	0.70859	0.70851	0.00014
45.34	0.70820	0.70850	0.00014
45.29	0.70866	0.70855	0.00013
45.21	0.70832	0.70859	0.00014
45.16	0.70822	0.70860	0.00013
45.12	0.70874	0.70864	0.00010
45.08	0.70886	0.70865	0.00011
45.03	0.70847	0.70862	0.00010
44.99	0.70852	0.70862	0.00010
44.95	0.70850	0.70864	0.00011
44.90	0.70870	0.70867	0.00010
44.86	0.70862	0.70866	0.00010
44.82	0.70893	0.70868	0.00011
44.77	0.70846	0.70862	0.00010
44.73	0.70864	0.70865	0.00010
44.69	0.70886	0.70864	0.00010
44.64	0.70850	0.70861	0.00009
44.60	0.70847	0.70860	0.00010
44.56	0.70877	0.70863	0.00010
44.51	0.70878	0.70862	0.00009
44.47	0.70860	0.70858	0.00009

44.43	0.70877	0.70857	0.00009
44.38	0.70839	0.70852	0.00010
44.34	0.70875	0.70857	0.00013
44.30	0.70852	0.70855	0.00013
44.25	0.70855	0.70854	0.00013
44.21	0.70844	0.70854	0.00013
44.17	0.70877	0.70854	0.00013
44.12	0.70863	0.70850	0.00012
44.08	0.70839	0.70851	0.00013
44.04	0.70849	0.70852	0.00012
43.99	0.70824	0.70851	0.00013
43.95	0.70895	0.70855	0.00011
43.91	0.70847	0.70848	0.00008
43.86	0.70844	0.70847	0.00009
43.82	0.70859	0.70850	0.00010
43.78	0.70842	0.70852	0.00011
43.73	0.70839	0.70852	0.00012
43.69	0.70869	0.70853	0.00011
43.65	0.70848	0.70849	0.00011
43.60	0.70838	0.70851	0.00012
43.56	0.70867	0.70851	0.00011
43.52	0.70831	0.70852	0.00012
43.47	0.70836	0.70852	0.00012
43.43	0.70873	0.70858	0.00014
43.39	0.70879	0.70855	0.00014
43.34	0.70839	0.70854	0.00013
43.30	0.70853	0.70852	0.00014
43.26	0.70831	0.70854	0.00015
43.21	0.70859	0.70857	0.00014
43.17	0.70846	0.70854	0.00015
43.13	0.70874	0.70856	0.00015
43.08	0.70829	0.70851	0.00016
43.04	0.70896	0.70848	0.00018
43.00	0.70846	0.70842	0.00015
42.95	0.70864	0.70844	0.00016
42.91	0.70822	0.70843	0.00015
42.87	0.70872	0.70848	0.00015
42.82	0.70860	0.70847	0.00015
42.78	0.70833	0.70843	0.00016
42.74	0.70863	0.70843	0.00016
42.69	0.70819	0.70842	0.00015
42.65	0.70799	0.70849	0.00017
42.61	0.70837	0.70851	0.00014
42.56	0.70866	0.70847	0.00018
42.52	0.70858	0.70845	0.00017
42.48	0.70868	0.70848	0.00020
42.43	0.70867	0.70848	0.00020
42.39	0.70820	0.70844	0.00020
42.35	0.70834	0.70846	0.00019
42.30	0.70853	0.70847	0.00019

42.26	0.70889	0.70848	0.00019
42.22	0.70820	0.70844	0.00017
42.17	0.70799	0.70847	0.00016
42.13	0.70838	0.70854	0.00013
42.09	0.70891	0.70857	0.00012
42.04	0.70871	0.70853	0.00010
42.00	0.70822	0.70851	0.00009
41.96	0.70839	0.70853	0.00006
41.91	0.70846	0.70856	0.00007
41.87	0.70866	0.70856	0.00007
41.83	0.70849	0.70853	0.00007
41.78	0.70851	0.70853	0.00007
41.74	0.70868	0.70853	0.00007
41.70	0.70866	0.70851	0.00007
41.65	0.70852	0.70848	0.00006
41.61	0.70849	0.70850	0.00008
41.57	0.70843	0.70848	0.00009
41.52	0.70872	0.70849	0.00009
41.48	0.70841	0.70844	0.00008
41.44	0.70839	0.70846	0.00008
41.39	0.70847	0.70849	0.00009
41.35	0.70850	0.70858	0.00021
41.31	0.70850	0.70858	0.00021
41.26	0.70837	0.70857	0.00021
41.22	0.70870	0.70861	0.00020
41.18	0.70830	0.70859	0.00020
41.14	0.70849	0.70856	0.00023
41.09	0.70829	0.70855	0.00023
41.05	0.70853	0.70856	0.00023
41.01	0.70870	0.70857	0.00023
40.96	0.70941	0.70855	0.00022
40.92	0.70849	0.70847	0.00012
40.88	0.70843	0.70850	0.00014
40.83	0.70871	0.70847	0.00016
40.79	0.70848	0.70848	0.00017
40.75	0.70802	0.70848	0.00017
40.70	0.70844	0.70855	0.00014
40.66	0.70837	0.70855	0.00014
40.62	0.70863	0.70859	0.00013
40.57	0.70851	0.70859	0.00013
40.53	0.70859	0.70859	0.00013
40.49	0.70884	0.70865	0.00017
40.44	0.70811	0.70861	0.00017
40.40	0.70878	0.70868	0.00013
40.36	0.70849	0.70863	0.00015
40.31	0.70873	0.70859	0.00018
40.27	0.70848	0.70854	0.00019
40.23	0.70873	0.70857	0.00019
40.18	0.70860	0.70856	0.00019
40.14	0.70859	0.70857	0.00019

40.10	0.70913	0.70853	0.00020
40.05	0.70842	0.70846	0.00015
40.01	0.70881	0.70844	0.00016
39.97	0.70827	0.70839	0.00014
39.92	0.70814	0.70842	0.00014
39.88	0.70825	0.70848	0.00014
39.84	0.70872	0.70854	0.00015
39.79	0.70866	0.70848	0.00016
39.75	0.70869	0.70845	0.00015
39.71	0.70820	0.70843	0.00014
39.66	0.70842	0.70848	0.00015
39.62	0.70822	0.70851	0.00015
39.58	0.70830	0.70854	0.00014
39.53	0.70858	0.70854	0.00014
39.49	0.70874	0.70852	0.00014
39.45	0.70883	0.70849	0.00013
39.40	0.70818	0.70840	0.00016
39.36	0.70835	0.70839	0.00016
39.32	0.70846	0.70841	0.00016
39.27	0.70873	0.70840	0.00016
39.23	0.70876	0.70839	0.00015
39.19	0.70845	0.70837	0.00013
39.14	0.70831	0.70839	0.00014
39.10	0.70837	0.70845	0.00018
39.06	0.70846	0.70848	0.00019
39.01	0.70792	0.70853	0.00020
38.97	0.70811	0.70858	0.00015
38.93	0.70852	0.70860	0.00013
38.88	0.70841	0.70859	0.00014
38.84	0.70859	0.70857	0.00015
38.80	0.70858	0.70854	0.00016
38.75	0.70862	0.70852	0.00016
38.71	0.70897	0.70848	0.00018
38.67	0.70864	0.70847	0.00017
38.62	0.70891	0.70848	0.00018
38.58	0.70847	0.70846	0.00015
38.54	0.70830	0.70845	0.00016
38.49	0.70837	0.70849	0.00016
38.45	0.70822	0.70853	0.00016
38.41	0.70830	0.70857	0.00015
38.36	0.70843	0.70861	0.00014
38.32	0.70816	0.70865	0.00013
38.28	0.70892	0.70871	0.00008
38.23	0.70872	0.70872	0.00008
38.19	0.70869	0.70872	0.00008
38.15	0.70843	0.70871	0.00009
38.10	0.70868	0.70871	0.00009
38.06	0.70871	0.70870	0.00009
38.02	0.70865	0.70863	0.00016
37.97	0.70874	0.70862	0.00016

37.93	0.70876	0.70857	0.00018
37.89	0.70884	0.70855	0.00017
37.84	0.70894	0.70851	0.00016
37.80	0.70875	0.70847	0.00013
37.76	0.70858	0.70845	0.00012
37.71	0.70844	0.70845	0.00012
37.67	0.70857	0.70844	0.00012
37.63	0.70805	0.70844	0.00012
37.58	0.70857	0.70849	0.00009
37.54	0.70819	0.70850	0.00009
37.50	0.70853	0.70852	0.00006
37.45	0.70849	0.70852	0.00006
37.41	0.70859	0.70853	0.00006
37.37	0.70849	0.70851	0.00007
37.32	0.70860	0.70850	0.00007
37.28	0.70832	0.70853	0.00009
37.24	0.70858	0.70850	0.00013
37.19	0.70855	0.70847	0.00014
37.15	0.70866	0.70848	0.00014
37.11	0.70844	0.70846	0.00014
37.06	0.70850	0.70844	0.00015
37.02	0.70859	0.70845	0.00015
36.98	0.70837	0.70845	0.00015
36.93	0.70843	0.70843	0.00016
36.89	0.70882	0.70847	0.00018
36.85	0.70801	0.70842	0.00016
36.81	0.70828	0.70847	0.00013
36.76	0.70866	0.70853	0.00015
36.72	0.70851	0.70855	0.00016
36.68	0.70821	0.70858	0.00017
36.63	0.70866	0.70861	0.00015
36.59	0.70859	0.70857	0.00017
36.55	0.70817	0.70856	0.00017
36.50	0.70880	0.70860	0.00014
36.46	0.70836	0.70850	0.00020
36.42	0.70848	0.70853	0.00019
36.37	0.70889	0.70852	0.00019
36.33	0.70882	0.70849	0.00018
36.29	0.70887	0.70844	0.00016
36.24	0.70844	0.70841	0.00014
36.20	0.70828	0.70844	0.00015
36.16	0.70850	0.70848	0.00015
36.11	0.70854	0.70850	0.00016
36.07	0.70786	0.70853	0.00017
36.03	0.70858	0.70860	0.00009
35.98	0.70841	0.70861	0.00009
35.94	0.70858	0.70863	0.00008
35.90	0.70835	0.70861	0.00009
35.85	0.70859	0.70861	0.00009
35.81	0.70874	0.70861	0.00009

35.77	0.70867	0.70859	0.00009
35.72	0.70870	0.70857	0.00009
35.68	0.70880	0.70854	0.00009
35.64	0.70860	0.70854	0.00010
35.59	0.70861	0.70853	0.00010
35.55	0.70865	0.70852	0.00010
35.51	0.70842	0.70852	0.00009
35.46	0.70833	0.70855	0.00010
35.42	0.70853	0.70852	0.00015
35.38	0.70857	0.70852	0.00015
35.33	0.70846	0.70854	0.00016
35.29	0.70838	0.70854	0.00016
35.25	0.70886	0.70855	0.00015
35.20	0.70852	0.70856	0.00017
35.16	0.70853	0.70859	0.00017
35.12	0.70861	0.70859	0.00017
35.07	0.70875	0.70859	0.00017
35.03	0.70799	0.70861	0.00018
34.99	0.70852	0.70870	0.00013
34.94	0.70879	0.70867	0.00015
34.90	0.70849	0.70871	0.00018
34.86	0.70846	0.70873	0.00017
34.81	0.70899	0.70875	0.00016
34.77	0.70873	0.70871	0.00016
34.73	0.70855	0.70869	0.00016
34.68	0.70861	0.70874	0.00017
34.64	0.70897	0.70871	0.00018
34.60	0.70888	0.70868	0.00017
34.55	0.70826	0.70867	0.00017
34.51	0.70917	0.70872	0.00014
34.47	0.70871	0.70866	0.00010
34.42	0.70867	0.70862	0.00013
34.38	0.70860	0.70860	0.00013
34.34	0.70852	0.70861	0.00013
34.29	0.70897	0.70862	0.00013
34.25	0.70841	0.70856	0.00010
34.21	0.70865	0.70858	0.00010
34.16	0.70878	0.70859	0.00010
34.12	0.70877	0.70858	0.00010
34.08	0.70855	0.70854	0.00009
34.03	0.70827	0.70855	0.00010
33.99	0.70851	0.70857	0.00008
33.95	0.70869	0.70862	0.00011
33.90	0.70857	0.70865	0.00013
33.86	0.70842	0.70864	0.00013
33.82	0.70863	0.70875	0.00021
33.77	0.70875	0.70874	0.00021
33.73	0.70861	0.70876	0.00022
33.69	0.70838	0.70877	0.00022
33.64	0.70870	0.70881	0.00020

33.60	0.70848	0.70885	0.00020
33.56	0.70898	0.70881	0.00025
33.51	0.70895	0.70874	0.00027
33.47	0.70849	0.70873	0.00027
33.43	0.70952	0.70876	0.00026
33.38	0.70856	0.70868	0.00020
33.34	0.70898	0.70863	0.00023
33.30	0.70862	0.70858	0.00022
33.25	0.70887	0.70856	0.00022
33.21	0.70905	0.70854	0.00021
33.17	0.70807	0.70850	0.00018
33.12	0.70825	0.70857	0.00016
33.08	0.70889	0.70866	0.00018
33.04	0.70882	0.70863	0.00018
32.99	0.70866	0.70858	0.00018
32.95	0.70814	0.70858	0.00018
32.91	0.70839	0.70863	0.00016
32.86	0.70850	0.70865	0.00015
32.82	0.70862	0.70863	0.00016
32.78	0.70864	0.70866	0.00017
32.73	0.70880	0.70866	0.00017
32.69	0.70919	0.70867	0.00017
32.65	0.70857	0.70862	0.00013
32.60	0.70828	0.70864	0.00013
32.56	0.70872	0.70866	0.00011
32.52	0.70856	0.70862	0.00013
32.48	0.70864	0.70857	0.00017
32.43	0.70832	0.70860	0.00018
32.39	0.70891	0.70863	0.00017
32.35	0.70861	0.70858	0.00017
32.30	0.70888	0.70858	0.00017
32.26	0.70869	0.70855	0.00015
32.22	0.70876	0.70857	0.00017
32.17	0.70847	0.70856	0.00016
32.13	0.70837	0.70855	0.00017
32.09	0.70806	0.70857	0.00016
32.04	0.70891	0.70862	0.00011
32.00	0.70864	0.70861	0.00010
31.96	0.70837	0.70865	0.00013
31.91	0.70862	0.70872	0.00014
31.87	0.70860	0.70875	0.00015
31.83	0.70893	0.70871	0.00017
31.78	0.70861	0.70869	0.00017
31.74	0.70842	0.70871	0.00017
31.70	0.70850	0.70871	0.00017
31.65	0.70863	0.70871	0.00017
31.61	0.70878	0.70870	0.00017
31.57	0.70898	0.70867	0.00018
31.52	0.70911	0.70859	0.00019
31.48	0.70893	0.70854	0.00015

31.44	0.70825	0.70851	0.00012
31.39	0.70870	0.70857	0.00012
31.35	0.70882	0.70854	0.00012
31.31	0.70840	0.70851	0.00010
31.26	0.70850	0.70854	0.00010
31.22	0.70853	0.70852	0.00011
31.18	0.70844	0.70852	0.00011
31.13	0.70821	0.70855	0.00012
31.09	0.70857	0.70858	0.00009
31.05	0.70862	0.70858	0.00009
31.00	0.70885	0.70856	0.00010
30.96	0.70849	0.70854	0.00007
30.92	0.70851	0.70855	0.00007
30.87	0.70865	0.70855	0.00008
30.83	0.70835	0.70854	0.00007
30.79	0.70854	0.70855	0.00006
30.74	0.70875	0.70854	0.00006
30.70	0.70848	0.70850	0.00006
30.66	0.70856	0.70852	0.00007
30.61	0.70843	0.70855	0.00010
30.57	0.70861	0.70859	0.00011
30.53	0.70866	0.70854	0.00014
30.48	0.70848	0.70855	0.00014
30.44	0.70852	0.70857	0.00015
30.40	0.70844	0.70857	0.00015
30.35	0.70850	0.70858	0.00014
30.31	0.70830	0.70858	0.00015
30.27	0.70870	0.70862	0.00014
30.22	0.70887	0.70866	0.00017
30.18	0.70877	0.70862	0.00016
30.14	0.70815	0.70861	0.00016
30.09	0.70876	0.70863	0.00013
30.05	0.70872	0.70864	0.00014
30.01	0.70847	0.70863	0.00014
29.96	0.70858	0.70865	0.00013
29.92	0.70844	0.70868	0.00014
29.88	0.70870	0.70872	0.00013
29.83	0.70912	0.70868	0.00015
29.79	0.70853	0.70861	0.00013
29.75	0.70858	0.70861	0.00013
29.70	0.70844	0.70860	0.00013
29.66	0.70885	0.70858	0.00015
29.62	0.70855	0.70854	0.00013
29.57	0.70867	0.70853	0.00014
29.53	0.70896	0.70853	0.00014
29.49	0.70877	0.70847	0.00010
29.44	0.70834	0.70846	0.00009
29.40	0.70842	0.70849	0.00009
29.36	0.70854	0.70848	0.00009
29.31	0.70846	0.70848	0.00009

29.27	0.70824	0.70847	0.00010
29.23	0.70847	0.70851	0.00010
29.18	0.70842	0.70850	0.00010
29.14	0.70870	0.70853	0.00010
29.10	0.70840	0.70850	0.00010
29.05	0.70865	0.70855	0.00013
29.01	0.70859	0.70854	0.00013
28.97	0.70835	0.70849	0.00015
28.92	0.70856	0.70849	0.00015
28.88	0.70828	0.70848	0.00015
28.84	0.70871	0.70850	0.00014
28.79	0.70836	0.70853	0.00018
28.75	0.70866	0.70852	0.00019
28.71	0.70842	0.70857	0.00023
28.66	0.70896	0.70857	0.00022
28.62	0.70846	0.70853	0.00021
28.58	0.70815	0.70854	0.00021
28.53	0.70833	0.70858	0.00019
28.49	0.70843	0.70858	0.00018
28.45	0.70850	0.70859	0.00018
28.40	0.70908	0.70854	0.00022
28.36	0.70823	0.70849	0.00018
28.32	0.70913	0.70852	0.00017
28.27	0.70846	0.70846	0.00010
28.23	0.70855	0.70848	0.00011
28.19	0.70853	0.70845	0.00012
28.15	0.70852	0.70844	0.00012
28.10	0.70840	0.70847	0.00014
28.06	0.70853	0.70845	0.00015
28.02	0.70801	0.70846	0.00016
27.97	0.70852	0.70853	0.00012
27.93	0.70853	0.70859	0.00017
27.89	0.70851	0.70867	0.00022
27.84	0.70865	0.70872	0.00023
27.80	0.70824	0.70873	0.00023
27.76	0.70852	0.70875	0.00021
27.71	0.70881	0.70874	0.00022
27.67	0.70817	0.70868	0.00024
27.63	0.70867	0.70869	0.00023
27.58	0.70868	0.70868	0.00023
27.54	0.70910	0.70866	0.00024
27.50	0.70934	0.70862	0.00022
27.45	0.70901	0.70853	0.00015
27.41	0.70876	0.70847	0.00010
27.37	0.70847	0.70847	0.00009
27.32	0.70835	0.70846	0.00009
27.28	0.70831	0.70853	0.00015
27.24	0.70824	0.70852	0.00015
27.19	0.70854	0.70854	0.00014
27.15	0.70848	0.70856	0.00014

27.11	0.70866	0.70858	0.00015
27.06	0.70842	0.70859	0.00015
27.02	0.70847	0.70861	0.00014
26.98	0.70872	0.70862	0.00014
26.93	0.70837	0.70861	0.00014
26.89	0.70905	0.70862	0.00013
26.85	0.70828	0.70860	0.00011
26.80	0.70841	0.70861	0.00010
26.76	0.70868	0.70872	0.00020
26.72	0.70875	0.70869	0.00020
26.67	0.70872	0.70868	0.00020
26.63	0.70859	0.70863	0.00022
26.59	0.70858	0.70864	0.00022
26.54	0.70865	0.70866	0.00022
26.50	0.70850	0.70865	0.00022
26.46	0.70886	0.70866	0.00022
26.41	0.70835	0.70861	0.00022
26.37	0.70950	0.70868	0.00022
26.33	0.70842	0.70860	0.00013
26.28	0.70859	0.70860	0.00013
26.24	0.70829	0.70860	0.00013
26.20	0.70866	0.70863	0.00011
26.15	0.70883	0.70860	0.00012
26.11	0.70851	0.70856	0.00012
26.07	0.70860	0.70858	0.00012
26.02	0.70840	0.70858	0.00012
25.98	0.70896	0.70859	0.00011
25.94	0.70870	0.70856	0.00008
25.89	0.70843	0.70850	0.00011
25.85	0.70864	0.70852	0.00011
25.81	0.70858	0.70852	0.00011
25.76	0.70837	0.70849	0.00012
25.72	0.70837	0.70856	0.00016
25.68	0.70871	0.70858	0.00015
25.63	0.70858	0.70855	0.00015
25.59	0.70856	0.70856	0.00016
25.55	0.70865	0.70857	0.00016
25.50	0.70812	0.70859	0.00017
25.46	0.70865	0.70866	0.00014
25.42	0.70859	0.70868	0.00015
25.37	0.70833	0.70864	0.00018
25.33	0.70903	0.70864	0.00018
25.29	0.70859	0.70859	0.00016
25.24	0.70834	0.70858	0.00016
25.20	0.70876	0.70867	0.00020
25.16	0.70862	0.70866	0.00020
25.11	0.70886	0.70865	0.00020
25.07	0.70878	0.70866	0.00020
25.03	0.70893	0.70863	0.00020
24.98	0.70815	0.70857	0.00020

24.94	0.70832	0.70861	0.00018
24.90	0.70858	0.70864	0.00017
24.85	0.70845	0.70863	0.00017
24.81	0.70923	0.70866	0.00016
24.77	0.70864	0.70857	0.00012
24.72	0.70861	0.70860	0.00014
24.68	0.70892	0.70856	0.00016
24.64	0.70852	0.70857	0.00016
24.59	0.70826	0.70860	0.00017
24.55	0.70856	0.70866	0.00016
24.51	0.70862	0.70860	0.00021
24.46	0.70853	0.70863	0.00022
24.42	0.70870	0.70866	0.00023
24.38	0.70830	0.70868	0.00023
24.33	0.70896	0.70870	0.00022
24.29	0.70827	0.70869	0.00021
24.25	0.70897	0.70873	0.00019
24.20	0.70885	0.70871	0.00018
24.16	0.70883	0.70867	0.00018
24.12	0.70796	0.70865	0.00018
24.07	0.70890	0.70872	0.00010
24.03	0.70890	0.70871	0.00009
23.99	0.70885	0.70869	0.00009
23.94	0.70853	0.70865	0.00009
23.90	0.70881	0.70867	0.00008
23.86	0.70873	0.70867	0.00008
23.82	0.70874	0.70867	0.00008
23.77	0.70845	0.70864	0.00009
23.73	0.70859	0.70864	0.00009
23.69	0.70866	0.70864	0.00009
23.64	0.70886	0.70867	0.00011
23.60	0.70862	0.70865	0.00010
23.56	0.70850	0.70868	0.00011
23.51	0.70871	0.70870	0.00011
23.47	0.70883	0.70871	0.00011
23.43	0.70872	0.70867	0.00013
23.38	0.70849	0.70864	0.00013
23.34	0.70841	0.70870	0.00015
23.30	0.70866	0.70874	0.00014
23.25	0.70894	0.70876	0.00014
23.21	0.70863	0.70872	0.00014
23.17	0.70891	0.70874	0.00014
23.12	0.70866	0.70874	0.00014
23.08	0.70887	0.70872	0.00015
23.04	0.70838	0.70866	0.00016
22.99	0.70848	0.70869	0.00015
22.95	0.70909	0.70871	0.00014
22.91	0.70877	0.70864	0.00012
22.86	0.70883	0.70863	0.00011
22.82	0.70856	0.70861	0.00010

22.78	0.70883	0.70864	0.00012
22.73	0.70887	0.70862	0.00011
22.69	0.70846	0.70855	0.00012
22.65	0.70833	0.70857	0.00012
22.60	0.70862	0.70859	0.00011
22.56	0.70867	0.70857	0.00011
22.52	0.70848	0.70859	0.00012
22.47	0.70866	0.70857	0.00013
22.43	0.70856	0.70857	0.00013
22.39	0.70891	0.70859	0.00013
22.34	0.70859	0.70858	0.00012
22.30	0.70824	0.70859	0.00012
22.26	0.70860	0.70861	0.00010
22.21	0.70853	0.70863	0.00010
22.17	0.70849	0.70862	0.00010
22.13	0.70882	0.70864	0.00010
22.08	0.70833	0.70864	0.00010
22.04	0.70864	0.70864	0.00010
22.00	0.70877	0.70865	0.00010
21.95	0.70877	0.70864	0.00010
21.91	0.70868	0.70865	0.00011
21.87	0.70851	0.70864	0.00011
21.82	0.70873	0.70865	0.00011
21.78	0.70849	0.70866	0.00011
21.74	0.70868	0.70868	0.00010
21.69	0.70882	0.70864	0.00013
21.65	0.70830	0.70861	0.00013
21.61	0.70876	0.70860	0.00014
21.56	0.70866	0.70859	0.00013
21.52	0.70885	0.70857	0.00013
21.48	0.70855	0.70851	0.00013
21.43	0.70868	0.70849	0.00013
21.39	0.70882	0.70850	0.00014
21.35	0.70866	0.70846	0.00012
21.30	0.70826	0.70845	0.00012
21.26	0.70851	0.70850	0.00012
21.22	0.70822	0.70850	0.00012
21.17	0.70863	0.70853	0.00011
21.13	0.70847	0.70849	0.00012
21.09	0.70827	0.70846	0.00014
21.04	0.70842	0.70843	0.00016
21.00	0.70878	0.70843	0.00016
20.96	0.70833	0.70839	0.00014
20.91	0.70861	0.70842	0.00015
20.87	0.70877	0.70843	0.00016
20.83	0.70850	0.70843	0.00016
20.78	0.70852	0.70843	0.00016
20.74	0.70827	0.70845	0.00016
20.70	0.70811	0.70843	0.00018
20.65	0.70801	0.70848	0.00017

20.61	0.70837	0.70857	0.00015
20.57	0.70842	0.70857	0.00015
20.52	0.70861	0.70861	0.00015
20.48	0.70872	0.70863	0.00016
20.44	0.70876	0.70863	0.00016
20.39	0.70853	0.70862	0.00015
20.35	0.70869	0.70863	0.00015
20.31	0.70807	0.70862	0.00015
20.26	0.70862	0.70870	0.00010
20.22	0.70888	0.70871	0.00010
20.18	0.70839	0.70868	0.00010
20.13	0.70885	0.70871	0.00007
20.09	0.70878	0.70868	0.00007
20.05	0.70877	0.70869	0.00008
20.00	0.70860	0.70869	0.00008
19.96	0.70861	0.70868	0.00008
19.92	0.70860	0.70864	0.00013
19.87	0.70889	0.70865	0.00013
19.83	0.70875	0.70859	0.00014
19.79	0.70860	0.70854	0.00015
19.74	0.70865	0.70853	0.00015
19.70	0.70855	0.70849	0.00016
19.66	0.70888	0.70852	0.00017
19.61	0.70876	0.70851	0.00016
19.57	0.70851	0.70847	0.00015
19.53	0.70819	0.70848	0.00015
19.49	0.70874	0.70851	0.00014
19.44	0.70825	0.70852	0.00015
19.40	0.70823	0.70858	0.00015
19.36	0.70850	0.70862	0.00013
19.31	0.70829	0.70864	0.00013
19.27	0.70883	0.70868	0.00010
19.23	0.70875	0.70865	0.00010
19.18	0.70842	0.70867	0.00011
19.14	0.70864	0.70869	0.00010
19.10	0.70845	0.70870	0.00010
19.05	0.70884	0.70870	0.00010
19.01	0.70888	0.70868	0.00009
18.97	0.70857	0.70865	0.00008
18.92	0.70876	0.70865	0.00008
18.88	0.70870	0.70869	0.00013
18.84	0.70854	0.70868	0.00013
18.79	0.70893	0.70869	0.00013
18.75	0.70859	0.70865	0.00012
18.71	0.70870	0.70865	0.00012
18.66	0.70849	0.70860	0.00015
18.62	0.70866	0.70861	0.00015
18.58	0.70857	0.70858	0.00015
18.53	0.70855	0.70858	0.00015
18.49	0.70915	0.70863	0.00017

18.45	0.70864	0.70864	0.00019
18.40	0.70858	0.70863	0.00019
18.36	0.70856	0.70863	0.00019
18.32	0.70864	0.70856	0.00023
18.27	0.70821	0.70858	0.00024
18.23	0.70850	0.70858	0.00023
18.19	0.70844	0.70861	0.00024
18.14	0.70856	0.70860	0.00024
18.10	0.70898	0.70858	0.00024
18.06	0.70927	0.70855	0.00023
18.01	0.70859	0.70849	0.00017
17.97	0.70852	0.70853	0.00020
17.93	0.70794	0.70852	0.00020
17.88	0.70877	0.70860	0.00015
17.84	0.70824	0.70860	0.00015
17.80	0.70879	0.70862	0.00013
17.75	0.70836	0.70863	0.00014
17.71	0.70836	0.70868	0.00013
17.67	0.70868	0.70870	0.00011
17.62	0.70862	0.70879	0.00022
17.58	0.70900	0.70886	0.00024
17.54	0.70843	0.70883	0.00024
17.49	0.70870	0.70888	0.00022
17.45	0.70880	0.70881	0.00027
17.41	0.70848	0.70881	0.00027
17.36	0.70884	0.70883	0.00027
17.32	0.70885	0.70878	0.00029
17.28	0.70855	0.70876	0.00029
17.23	0.70966	0.70882	0.00029
17.19	0.70928	0.70870	0.00023
17.15	0.70871	0.70863	0.00020
17.10	0.70890	0.70865	0.00020
17.06	0.70807	0.70863	0.00020
17.02	0.70877	0.70864	0.00019
16.97	0.70870	0.70866	0.00020
16.93	0.70829	0.70868	0.00021
16.89	0.70866	0.70867	0.00021
16.84	0.70918	0.70866	0.00021
16.80	0.70846	0.70859	0.00018
16.76	0.70856	0.70861	0.00018
16.71	0.70888	0.70869	0.00023
16.67	0.70874	0.70865	0.00023
16.63	0.70813	0.70865	0.00023
16.58	0.70899	0.70868	0.00021
16.54	0.70889	0.70864	0.00020
16.50	0.70825	0.70862	0.00019
16.45	0.70849	0.70868	0.00018
16.41	0.70848	0.70870	0.00017
16.37	0.70870	0.70869	0.00018
16.32	0.70937	0.70869	0.00018

16.28	0.70844	0.70862	0.00010
16.24	0.70878	0.70866	0.00011
16.19	0.70844	0.70864	0.00011
16.15	0.70855	0.70867	0.00011
16.11	0.70870	0.70867	0.00011
16.06	0.70884	0.70866	0.00011
16.02	0.70870	0.70864	0.00010
15.98	0.70835	0.70864	0.00010
15.93	0.70875	0.70870	0.00009
15.89	0.70861	0.70862	0.00016
15.85	0.70893	0.70863	0.00016
15.80	0.70852	0.70857	0.00015
15.76	0.70874	0.70859	0.00016
15.72	0.70855	0.70855	0.00016
15.67	0.70857	0.70859	0.00018
15.63	0.70871	0.70857	0.00018
15.59	0.70870	0.70852	0.00019
15.54	0.70890	0.70845	0.00021
15.50	0.70803	0.70842	0.00019
15.46	0.70862	0.70853	0.00021
15.41	0.70833	0.70850	0.00021
15.37	0.70875	0.70850	0.00021
15.33	0.70836	0.70852	0.00022
15.28	0.70892	0.70860	0.00025
15.24	0.70838	0.70862	0.00026
15.20	0.70827	0.70865	0.00026
15.16	0.70798	0.70867	0.00025
15.11	0.70860	0.70876	0.00020
15.07	0.70907	0.70876	0.00019
15.03	0.70836	0.70872	0.00018
14.98	0.70836	0.70877	0.00017
14.94	0.70889	0.70882	0.00015
14.90	0.70914	0.70881	0.00016
14.85	0.70915	0.70876	0.00014
14.81	0.70872	0.70869	0.00010
14.77	0.70845	0.70869	0.00011
14.72	0.70886	0.70875	0.00008
14.68	0.70864	0.70871	0.00009
14.64	0.70862	0.70875	0.00011
14.59	0.70887	0.70887	0.00008
13.47	0.70873	0.70878	0.00017
13.42	0.70884	0.70878	0.00017
13.38	0.70877	0.70878	0.00017
13.34	0.70838	0.70880	0.00018
13.29	0.70906	0.70885	0.00015
13.25	0.70895	0.70881	0.00015
13.21	0.70831	0.70877	0.00015
13.16	0.70865	0.70880	0.00012
13.12	0.70901	0.70884	0.00012
13.08	0.70911	0.70881	0.00012

13.03	0.70877	0.70881	0.00011
12.99	0.70879	0.70877	0.00014
12.95	0.70895	0.70880	0.00015
12.90	0.70893	0.70879	0.00015
12.86	0.70866	0.70874	0.00016
12.82	0.70855	0.70875	0.00016
12.77	0.70860	0.70878	0.00015
12.73	0.70899	0.70881	0.00015
12.69	0.70876	0.70875	0.00016
12.64	0.70908	0.70877	0.00017
12.60	0.70837	0.70874	0.00015
12.56	0.70908	0.70879	0.00013
12.51	0.70885	0.70871	0.00014
12.47	0.70845	0.70872	0.00015
12.43	0.70878	0.70878	0.00014
12.38	0.70886	0.70878	0.00014
12.34	0.70886	0.70877	0.00014
12.30	0.70843	0.70879	0.00015
12.25	0.70900	0.70889	0.00018
12.21	0.70877	0.70890	0.00018
12.17	0.70881	0.70885	0.00023
12.12	0.70832	0.70884	0.00023
12.08	0.70895	0.70885	0.00022
12.04	0.70900	0.70880	0.00023
11.99	0.70883	0.70877	0.00024
11.95	0.70878	0.70877	0.00026
11.91	0.70899	0.70877	0.00028
11.86	0.70944	0.70873	0.00029
11.82	0.70912	0.70859	0.00022
11.78	0.70822	0.70845	0.00012
11.73	0.70869	0.70853	0.00009
11.69	0.70847	0.70844	0.00002
11.65	0.70842	0.70842	0.00004
10.95	0.70918	0.70883	0.00021
10.91	0.70890	0.70878	0.00020
10.87	0.70887	0.70881	0.00021
10.83	0.70823	0.70876	0.00022
10.78	0.70864	0.70881	0.00019
10.74	0.70865	0.70886	0.00020
10.70	0.70901	0.70887	0.00020
10.65	0.70844	0.70888	0.00020
10.61	0.70925	0.70892	0.00018
10.57	0.70910	0.70884	0.00018
10.52	0.70868	0.70881	0.00017
10.48	0.70919	0.70887	0.00019
10.44	0.70843	0.70882	0.00018
10.39	0.70874	0.70877	0.00023
10.35	0.70916	0.70875	0.00024
10.31	0.70873	0.70872	0.00023
10.26	0.70912	0.70875	0.00023

10.22	0.70875	0.70874	0.00023
10.18	0.70845	0.70871	0.00023
10.13	0.70887	0.70874	0.00022
10.09	0.70924	0.70881	0.00029
10.05	0.70871	0.70870	0.00030
10.00	0.70798	0.70868	0.00030
9.96	0.70850	0.70872	0.00027
9.92	0.70889	0.70873	0.00027
9.87	0.70895	0.70871	0.00027
9.83	0.70903	0.70866	0.00026
9.79	0.70851	0.70869	0.00029
9.74	0.70869	0.70875	0.00029
9.70	0.70964	0.70876	0.00029
9.66	0.70814	0.70865	0.00022
9.61	0.70846	0.70874	0.00019
9.57	0.70836	0.70880	0.00019
9.53	0.70865	0.70890	0.00020
9.48	0.70863	0.70890	0.00020
9.44	0.70852	0.70890	0.00019
9.40	0.70933	0.70892	0.00018
9.35	0.70905	0.70884	0.00017
9.31	0.70879	0.70880	0.00017
9.27	0.70859	0.70882	0.00017
9.22	0.70901	0.70882	0.00017
9.18	0.70903	0.70874	0.00020
9.14	0.70940	0.70878	0.00024
9.09	0.70865	0.70877	0.00023
9.05	0.70868	0.70882	0.00024
9.01	0.70869	0.70879	0.00025
8.96	0.70856	0.70881	0.00025
8.92	0.70864	0.70877	0.00028
8.88	0.70892	0.70874	0.00029
8.83	0.70867	0.70870	0.00029
8.79	0.70817	0.70871	0.00029
8.75	0.70939	0.70879	0.00027
8.70	0.70937	0.70874	0.00024
8.66	0.70907	0.70868	0.00019
8.62	0.70841	0.70863	0.00017
8.57	0.70890	0.70863	0.00017
8.53	0.70811	0.70861	0.00016
8.49	0.70838	0.70865	0.00012
8.44	0.70855	0.70872	0.00013
8.40	0.70876	0.70878	0.00014
8.36	0.70900	0.70881	0.00015
8.31	0.70887	0.70882	0.00016
8.27	0.70872	0.70881	0.00016
8.23	0.70860	0.70880	0.00017
8.18	0.70845	0.70878	0.00017
8.14	0.70862	0.70879	0.00017
8.10	0.70860	0.70880	0.00017

8.05	0.70907	0.70886	0.00018
8.01	0.70907	0.70883	0.00018
7.97	0.70912	0.70870	0.00026
7.92	0.70911	0.70877	0.00033
7.88	0.70869	0.70869	0.00034
7.84	0.70862	0.70867	0.00034
7.79	0.70847	0.70865	0.00034
7.75	0.70852	0.70871	0.00035
7.71	0.70870	0.70870	0.00035
7.66	0.70924	0.70865	0.00036
7.62	0.70875	0.70862	0.00035
7.58	0.70782	0.70861	0.00035
7.53	0.70982	0.70872	0.00030
7.49	0.70831	0.70866	0.00021
7.45	0.70842	0.70869	0.00019
7.40	0.70850	0.70873	0.00018
7.36	0.70903	0.70875	0.00018
7.32	0.70846	0.70875	0.00018
7.27	0.70820	0.70873	0.00019
7.23	0.70892	0.70884	0.00018
7.19	0.70868	0.70880	0.00019
7.14	0.70891	0.70881	0.00019
7.10	0.70913	0.70883	0.00020
7.06	0.70870	0.70879	0.00018
7.01	0.70878	0.70880	0.00018
6.97	0.70864	0.70880	0.00019
6.93	0.70903	0.70882	0.00020
6.88	0.70829	0.70879	0.00021
6.84	0.70934	0.70888	0.00017
6.80	0.70854	0.70878	0.00011
6.75	0.70877	0.70885	0.00008
6.71	0.70903	0.70887	0.00009
6.67	0.70875	0.70879	0.00004
6.62	0.70883	0.70883	0.00004
6.15	0.70929	0.70897	0.00014
6.11	0.70904	0.70894	0.00012
6.06	0.70909	0.70896	0.00013
6.02	0.70883	0.70896	0.00013
5.98	0.70894	0.70898	0.00013
5.93	0.70885	0.70894	0.00016
5.89	0.70924	0.70893	0.00016
5.85	0.70865	0.70890	0.00015
5.80	0.70905	0.70896	0.00015
5.76	0.70868	0.70888	0.00021
5.72	0.70899	0.70891	0.00021
5.67	0.70925	0.70894	0.00022
5.63	0.70915	0.70886	0.00023
5.59	0.70900	0.70881	0.00022
5.54	0.70849	0.70882	0.00023
5.50	0.70883	0.70883	0.00022

5.46	0.70894	0.70886	0.00023
5.41	0.70923	0.70888	0.00024
5.37	0.70821	0.70881	0.00023
5.33	0.70901	0.70886	0.00020
5.28	0.70931	0.70876	0.00025
5.24	0.70848	0.70873	0.00023
5.20	0.70863	0.70874	0.00023
5.15	0.70910	0.70876	0.00022
5.11	0.70856	0.70871	0.00021
5.07	0.70919	0.70876	0.00022
5.02	0.70910	0.70877	0.00023
4.98	0.70853	0.70875	0.00022
4.94	0.70866	0.70878	0.00022
4.89	0.70805	0.70884	0.00023
4.85	0.70905	0.70893	0.00015
4.81	0.70858	0.70889	0.00016
4.76	0.70875	0.70892	0.00014
4.72	0.70862	0.70894	0.00014
4.68	0.70910	0.70895	0.00013
4.63	0.70930	0.70893	0.00013
4.59	0.70884	0.70881	0.00019
4.55	0.70887	0.70880	0.00019
4.50	0.70921	0.70884	0.00021
4.46	0.70896	0.70885	0.00021
4.42	0.70865	0.70880	0.00022
4.37	0.70894	0.70879	0.00023
4.33	0.70888	0.70880	0.00023
4.29	0.70871	0.70876	0.00024
4.24	0.70893	0.70879	0.00024
4.20	0.70808	0.70881	0.00025
4.16	0.70875	0.70891	0.00019
4.11	0.70926	0.70896	0.00020
4.07	0.70928	0.70895	0.00020
4.03	0.70853	0.70898	0.00022
3.98	0.70850	0.70901	0.00020
3.94	0.70903	0.70906	0.00016
3.90	0.70854	0.70906	0.00016
3.85	0.70903	0.70907	0.00014
3.81	0.70903	0.70905	0.00015
3.77	0.70913	0.70903	0.00016
3.72	0.70928	0.70907	0.00018
3.68	0.70918	0.70901	0.00019
3.64	0.70952	0.70897	0.00019
3.59	0.70883	0.70892	0.00014
3.55	0.70897	0.70895	0.00015
3.51	0.70902	0.70896	0.00015
3.46	0.70874	0.70893	0.00015
3.42	0.70882	0.70893	0.00015
3.38	0.70884	0.70897	0.00016
3.33	0.70947	0.70895	0.00018

3.29	0.70867	0.70886	0.00014
3.25	0.70884	0.70890	0.00014
3.20	0.70903	0.70894	0.00015
3.16	0.70912	0.70887	0.00019
3.12	0.70902	0.70886	0.00019
3.07	0.70871	0.70886	0.00019
3.03	0.70880	0.70884	0.00020
2.99	0.70922	0.70889	0.00021
2.94	0.70858	0.70885	0.00020
2.90	0.70860	0.70889	0.00019
2.86	0.70911	0.70890	0.00019
2.81	0.70920	0.70886	0.00018
2.77	0.70837	0.70888	0.00020
2.73	0.70903	0.70892	0.00017
2.68	0.70901	0.70891	0.00017
2.64	0.70851	0.70891	0.00017
2.60	0.70925	0.70890	0.00017
2.55	0.70878	0.70884	0.00016
2.51	0.70904	0.70890	0.00019
2.47	0.70873	0.70891	0.00020
2.42	0.70870	0.70893	0.00020
2.38	0.70938	0.70893	0.00019
2.34	0.70878	0.70890	0.00017
2.29	0.70893	0.70891	0.00017
2.25	0.70897	0.70885	0.00020
2.21	0.70847	0.70893	0.00027
2.17	0.70859	0.70902	0.00026
2.12	0.70937	0.70912	0.00026
2.08	0.70918	0.70907	0.00026
2.04	0.70890	0.70906	0.00026
1.99	0.70878	0.70904	0.00027
1.95	0.70907	0.70909	0.00026
1.91	0.70880	0.70907	0.00026
1.86	0.70839	0.70911	0.00026
1.82	0.70978	0.70921	0.00021
1.78	0.70938	0.70915	0.00016
1.73	0.70956	0.70913	0.00016
1.69	0.70886	0.70908	0.00012
1.65	0.70904	0.70912	0.00011
1.60	0.70877	0.70904	0.00021
1.56	0.70920	0.70907	0.00020
1.52	0.70888	0.70903	0.00021
1.47	0.70926	0.70903	0.00021
1.43	0.70937	0.70902	0.00020
1.39	0.70922	0.70899	0.00019
1.34	0.70915	0.70895	0.00019
1.30	0.70910	0.70897	0.00020
1.26	0.70918	0.70899	0.00021
1.21	0.70824	0.70897	0.00021
1.17	0.70914	0.70905	0.00013

1.13	0.70880	0.70909	0.00017
1.08	0.70883	0.70910	0.00017
1.04	0.70915	0.70917	0.00017
1.00	0.70909	0.70918	0.00017
0.95	0.70881	0.70912	0.00022
0.91	0.70937	0.70918	0.00022
0.87	0.70933	0.70908	0.00027
0.82	0.70896	0.70898	0.00030
0.78	0.70901	0.70896	0.00030
0.74	0.70960	0.70895	0.00030
0.69	0.70888	0.70886	0.00026
0.65	0.70949	0.70889	0.00027
0.61	0.70925	0.70888	0.00026
0.56	0.70846	0.70887	0.00026
0.52	0.70943	0.70888	0.00025
0.48	0.70837	0.70889	0.00025
0.43	0.70832	0.70894	0.00023
0.39	0.70881	0.70900	0.00019
0.35	0.70889	0.70903	0.00020
0.30	0.70873	0.70905	0.00021
0.26	0.70920	0.70910	0.00021
0.22	0.70939	0.70908	0.00023
0.17	0.70908	0.70901	0.00023
0.13	0.70860	0.70898	0.00029
0.09	0.70948	0.70918	0.00027
0.04	0.70887		

ARB 109.2.1 (M2)

Distance from cervix (mm)	$^{87}Sr/^{86}Sr$	10 point mov. average	2 SE on mov. average
22.99	0.70971	0.70953	0.00020
22.95	0.70998	0.70950	0.00019
22.90	0.70914	0.70948	0.00018
22.86	0.70935	0.70952	0.00016
22.82	0.70972	0.70951	0.00016
22.77	0.70954	0.70951	0.00016
22.73	0.70940	0.70952	0.00016
22.69	0.70937	0.70956	0.00017
22.64	0.70913	0.70955	0.00017
22.60	0.70997	0.70962	0.00015
22.56	0.70940	0.70965	0.00018
22.51	0.70981	0.70961	0.00021
22.47	0.70946	0.70953	0.00023
22.43	0.70929	0.70958	0.00025
22.38	0.70975	0.70963	0.00024
22.34	0.70960	0.70962	0.00024
22.30	0.70978	0.70962	0.00024
22.25	0.70936	0.70964	0.00024
22.21	0.70977	0.70966	0.00024
22.17	0.71023	0.70963	0.00024
22.12	0.70908	0.70959	0.00020
22.08	0.70903	0.70961	0.00018
22.04	0.70996	0.70968	0.00013
21.99	0.70977	0.70962	0.00013
21.95	0.70966	0.70955	0.00017
21.91	0.70959	0.70951	0.00018
21.86	0.70993	0.70948	0.00018
21.82	0.70961	0.70946	0.00016
21.78	0.70947	0.70946	0.00016
21.73	0.70978	0.70946	0.00016
21.69	0.70928	0.70943	0.00015
21.65	0.70978	0.70944	0.00015
21.60	0.70934	0.70942	0.00014
21.56	0.70902	0.70948	0.00016
21.52	0.70928	0.70949	0.00015
21.47	0.70933	0.70956	0.00016
21.43	0.70976	0.70957	0.00016
21.39	0.70959	0.70951	0.00017
21.34	0.70945	0.70950	0.00017
21.30	0.70942	0.70947	0.00018
21.25	0.70962	0.70950	0.00017
21.21	0.70982	0.70949	0.00017
21.17	0.70912	0.70949	0.00017
21.12	0.70994	0.70953	0.00015
21.08	0.70943	0.70950	0.00012
21.04	0.70920	0.70948	0.00013
20.99	0.70946	0.70951	0.00012

20.95	0.70923	0.70952	0.00012
20.91	0.70949	0.70955	0.00010
20.86	0.70971	0.70956	0.00010
20.82	0.70954	0.70954	0.00009
20.78	0.70980	0.70954	0.00009
20.73	0.70955	0.70951	0.00008
20.69	0.70959	0.70950	0.00008
20.65	0.70921	0.70947	0.00008
20.60	0.70952	0.70948	0.00007
20.56	0.70953	0.70944	0.00009
20.52	0.70952	0.70944	0.00009
20.47	0.70963	0.70943	0.00009
20.43	0.70950	0.70943	0.00009
20.39	0.70955	0.70942	0.00009
20.34	0.70944	0.70944	0.00010
20.30	0.70932	0.70948	0.00014
20.26	0.70927	0.70950	0.00013
20.21	0.70922	0.70953	0.00012
20.17	0.70950	0.70958	0.00011
20.13	0.70943	0.70963	0.00013
20.08	0.70965	0.70962	0.00013
20.04	0.70945	0.70960	0.00014
20.00	0.70975	0.70958	0.00016
19.95	0.70934	0.70956	0.00015
19.91	0.70988	0.70956	0.00015
19.87	0.70951	0.70952	0.00014
19.82	0.70958	0.70949	0.00015
19.78	0.70975	0.70948	0.00015
19.74	0.70995	0.70943	0.00014
19.69	0.70936	0.70934	0.00010
19.65	0.70944	0.70937	0.00012
19.61	0.70922	0.70936	0.00012
19.56	0.70961	0.70934	0.00014
19.52	0.70934	0.70931	0.00013
19.48	0.70939	0.70935	0.00015
19.43	0.70924	0.70934	0.00016
19.39	0.70950	0.70934	0.00016
19.35	0.70926	0.70931	0.00016
19.30	0.70904	0.70930	0.00016
19.22	0.70969	0.70937	0.00018
19.17	0.70932	0.70938	0.00018
19.13	0.70900	0.70938	0.00018
19.09	0.70937	0.70943	0.00016
19.04	0.70974	0.70943	0.00016
19.00	0.70921	0.70941	0.00014
18.96	0.70930	0.70946	0.00015
18.91	0.70915	0.70947	0.00015
18.87	0.70914	0.70951	0.00013
18.83	0.70981	0.70949	0.00017
18.78	0.70972	0.70940	0.00019

18.74	0.70940	0.70938	0.00018
18.70	0.70942	0.70935	0.00019
18.65	0.70938	0.70928	0.00023
18.61	0.70952	0.70924	0.00023
18.57	0.70976	0.70920	0.00022
18.52	0.70936	0.70917	0.00020
18.48	0.70960	0.70917	0.00020
18.44	0.70891	0.70909	0.00019
18.39	0.70890	0.70914	0.00019
18.35	0.70956	0.70918	0.00018
18.31	0.70904	0.70909	0.00019
18.26	0.70871	0.70912	0.00020
18.22	0.70899	0.70913	0.00019
18.18	0.70915	0.70916	0.00019
18.13	0.70947	0.70912	0.00020
18.09	0.70940	0.70907	0.00019
18.04	0.70880	0.70904	0.00017
18.00	0.70941	0.70909	0.00017
17.96	0.70929	0.70907	0.00016
17.91	0.70864	0.70905	0.00015
17.87	0.70937	0.70907	0.00013
17.83	0.70884	0.70900	0.00014
17.78	0.70919	0.70903	0.00013
17.74	0.70877	0.70898	0.00014
17.70	0.70894	0.70903	0.00014
17.65	0.70913	0.70903	0.00014
17.61	0.70931	0.70902	0.00014
17.57	0.70920	0.70900	0.00013
17.52	0.70911	0.70903	0.00015
17.48	0.70886	0.70905	0.00016
17.44	0.70866	0.70910	0.00017
17.39	0.70908	0.70909	0.00018
17.35	0.70873	0.70906	0.00019
17.31	0.70927	0.70913	0.00018
17.26	0.70897	0.70914	0.00018
17.22	0.70904	0.70918	0.00019
17.18	0.70912	0.70919	0.00019
17.13	0.70948	0.70916	0.00020
17.09	0.70926	0.70911	0.00019
17.05	0.70937	0.70908	0.00018
17.00	0.70856	0.70910	0.00020
16.96	0.70885	0.70912	0.00018
16.92	0.70943	0.70917	0.00018
16.87	0.70929	0.70914	0.00017
16.83	0.70942	0.70911	0.00017
16.79	0.70910	0.70910	0.00016
16.74	0.70883	0.70909	0.00016
16.70	0.70897	0.70907	0.00019
16.66	0.70902	0.70908	0.00018
16.61	0.70956	0.70911	0.00019

16.57	0.70871	0.70907	0.00016
16.53	0.70936	0.70911	0.00014
16.48	0.70915	0.70907	0.00013
16.44	0.70900	0.70905	0.00014
16.40	0.70934	0.70903	0.00015
16.35	0.70898	0.70897	0.00014
16.31	0.70860	0.70896	0.00014
16.27	0.70904	0.70902	0.00012
16.22	0.70933	0.70905	0.00013
16.18	0.70918	0.70905	0.00013
16.14	0.70912	0.70904	0.00013
16.09	0.70896	0.70905	0.00013
16.05	0.70891	0.70906	0.00013
16.01	0.70878	0.70909	0.00013
15.96	0.70878	0.70912	0.00011
15.92	0.70892	0.70917	0.00008
15.88	0.70919	0.70919	0.00006
15.83	0.70933	0.70915	0.00009
15.79	0.70932	0.70913	0.00008
15.75	0.70911	0.70907	0.00010
15.70	0.70917	0.70911	0.00012
15.66	0.70913	0.70913	0.00013
15.62	0.70918	0.70910	0.00015
15.57	0.70910	0.70915	0.00018
15.53	0.70928	0.70916	0.00018
15.49	0.70909	0.70918	0.00019
15.44	0.70884	0.70915	0.00020
15.40	0.70906	0.70916	0.00020
15.36	0.70877	0.70910	0.00024
15.31	0.70945	0.70914	0.00023
15.27	0.70937	0.70908	0.00023
15.23	0.70885	0.70908	0.00022
15.18	0.70964	0.70913	0.00023
15.14	0.70920	0.70912	0.00022
15.10	0.70949	0.70910	0.00022
15.05	0.70883	0.70909	0.00021
15.01	0.70892	0.70913	0.00020
14.97	0.70849	0.70915	0.00020
14.92	0.70918	0.70925	0.00014
14.88	0.70883	0.70921	0.00018
14.84	0.70935	0.70923	0.00016
14.79	0.70941	0.70917	0.00018
14.75	0.70952	0.70910	0.00019
14.70	0.70898	0.70904	0.00017
14.66	0.70935	0.70903	0.00018
14.62	0.70932	0.70902	0.00017
14.57	0.70912	0.70897	0.00016
14.53	0.70948	0.70895	0.00016
14.49	0.70870	0.70890	0.00011
14.44	0.70903	0.70893	0.00010

14.40	0.70879	0.70889	0.00013
14.36	0.70873	0.70890	0.00013
14.31	0.70886	0.70897	0.00016
14.27	0.70893	0.70902	0.00017
14.23	0.70926	0.70903	0.00017
14.18	0.70876	0.70897	0.00017
14.14	0.70892	0.70901	0.00017
14.10	0.70896	0.70897	0.00019
14.05	0.70909	0.70898	0.00019
14.01	0.70854	0.70894	0.00019
13.97	0.70897	0.70899	0.00017
13.92	0.70944	0.70903	0.00019
13.88	0.70932	0.70896	0.00017
13.84	0.70900	0.70898	0.00019
13.79	0.70871	0.70896	0.00019
13.75	0.70913	0.70898	0.00018
13.71	0.70855	0.70898	0.00018
13.66	0.70905	0.70902	0.00016
13.62	0.70875	0.70904	0.00016
13.58	0.70895	0.70905	0.00015
13.53	0.70940	0.70902	0.00018
13.49	0.70880	0.70897	0.00015
13.45	0.70947	0.70899	0.00015
13.40	0.70883	0.70895	0.00011
13.36	0.70885	0.70892	0.00013
13.32	0.70915	0.70892	0.00013
13.27	0.70899	0.70890	0.00012
13.23	0.70918	0.70889	0.00012
13.19	0.70887	0.70883	0.00011
13.14	0.70861	0.70883	0.00011
13.10	0.70889	0.70888	0.00011
13.06	0.70903	0.70890	0.00012
13.01	0.70909	0.70893	0.00016
12.97	0.70858	0.70892	0.00015
12.93	0.70885	0.70897	0.00014
12.88	0.70887	0.70903	0.00016
12.84	0.70890	0.70905	0.00016
12.80	0.70865	0.70906	0.00015
12.75	0.70881	0.70906	0.00015
12.71	0.70910	0.70905	0.00017
12.67	0.70907	0.70905	0.00017
12.62	0.70942	0.70904	0.00017
12.54	0.70892	0.70899	0.00014
12.49	0.70915	0.70900	0.00014
12.45	0.70944	0.70894	0.00017
12.41	0.70904	0.70889	0.00013
12.36	0.70904	0.70887	0.00012
12.32	0.70865	0.70889	0.00014
12.28	0.70867	0.70894	0.00013
12.23	0.70909	0.70899	0.00013

12.19	0.70900	0.70897	0.00012
12.10	0.70892	0.70892	0.00015
12.06	0.70901	0.70894	0.00016
12.02	0.70853	0.70894	0.00016
11.97	0.70893	0.70896	0.00013
11.93	0.70887	0.70900	0.00015
11.89	0.70923	0.70903	0.00015
11.84	0.70913	0.70897	0.00016
11.80	0.70917	0.70897	0.00016
11.76	0.70892	0.70893	0.00015
11.71	0.70853	0.70893	0.00016
11.67	0.70912	0.70893	0.00015
11.63	0.70894	0.70888	0.00016
11.58	0.70880	0.70887	0.00016
11.54	0.70931	0.70883	0.00019
11.50	0.70915	0.70880	0.00016
11.45	0.70867	0.70880	0.00017
11.41	0.70913	0.70882	0.00016
11.36	0.70876	0.70879	0.00015
11.32	0.70887	0.70882	0.00015
11.28	0.70857	0.70884	0.00017
11.23	0.70858	0.70887	0.00016
11.19	0.70884	0.70890	0.00015
11.15	0.70837	0.70891	0.00015
11.10	0.70904	0.70893	0.00011
11.06	0.70918	0.70893	0.00011
11.02	0.70885	0.70886	0.00012
10.97	0.70885	0.70884	0.00013
10.93	0.70901	0.70887	0.00015
10.89	0.70913	0.70881	0.00017
10.84	0.70886	0.70878	0.00016
10.80	0.70892	0.70879	0.00016
10.76	0.70857	0.70880	0.00017
10.71	0.70900	0.70885	0.00016
10.67	0.70859	0.70880	0.00017
10.63	0.70859	0.70881	0.00016
10.58	0.70921	0.70879	0.00018
10.54	0.70843	0.70873	0.00015
10.50	0.70886	0.70877	0.00014
10.45	0.70898	0.70877	0.00013
10.41	0.70897	0.70876	0.00013
10.37	0.70902	0.70875	0.00012
10.32	0.70859	0.70874	0.00011
10.28	0.70867	0.70871	0.00014
10.24	0.70836	0.70872	0.00014
10.19	0.70873	0.70878	0.00012
10.15	0.70877	0.70881	0.00013
10.11	0.70891	0.70879	0.00014
10.06	0.70868	0.70878	0.00013
10.02	0.70893	0.70881	0.00013

9.98	0.70888	0.70881	0.00014
9.93	0.70886	0.70883	0.00015
9.89	0.70829	0.70882	0.00015
9.85	0.70884	0.70885	0.00011
9.80	0.70894	0.70883	0.00012
9.76	0.70897	0.70881	0.00012
9.72	0.70857	0.70877	0.00012
9.67	0.70886	0.70879	0.00011
9.63	0.70892	0.70874	0.00014
9.59	0.70896	0.70870	0.00015
9.54	0.70914	0.70867	0.00013
9.50	0.70872	0.70865	0.00010
9.46	0.70861	0.70866	0.00011
9.41	0.70865	0.70868	0.00011
9.37	0.70869	0.70867	0.00012
9.33	0.70856	0.70866	0.00012
9.28	0.70882	0.70866	0.00012
9.24	0.70835	0.70865	0.00011
9.20	0.70847	0.70866	0.00010
9.15	0.70874	0.70870	0.00009
9.11	0.70889	0.70871	0.00010
9.07	0.70882	0.70872	0.00011
9.02	0.70885	0.70874	0.00013
8.98	0.70852	0.70874	0.00013
8.94	0.70856	0.70876	0.00012
8.89	0.70861	0.70876	0.00012
8.85	0.70868	0.70881	0.00014
8.81	0.70849	0.70883	0.00014
8.76	0.70881	0.70891	0.00015
8.72	0.70892	0.70891	0.00015
8.68	0.70896	0.70887	0.00016
8.63	0.70905	0.70886	0.00016
8.59	0.70882	0.70889	0.00019
8.55	0.70873	0.70891	0.00019
8.50	0.70849	0.70896	0.00020
8.46	0.70916	0.70899	0.00018
8.42	0.70884	0.70896	0.00017
8.37	0.70931	0.70896	0.00017
8.33	0.70878	0.70889	0.00017
8.29	0.70857	0.70886	0.00019
8.24	0.70881	0.70892	0.00018
8.20	0.70940	0.70893	0.00018
8.15	0.70899	0.70887	0.00015
8.11	0.70925	0.70885	0.00014
8.07	0.70874	0.70879	0.00012
8.02	0.70895	0.70882	0.00012
7.98	0.70881	0.70882	0.00012
7.94	0.70858	0.70881	0.00012
7.89	0.70850	0.70885	0.00012
7.85	0.70913	0.70892	0.00011

7.81	0.70894	0.70885	0.00013
7.76	0.70881	0.70883	0.00014
7.72	0.70880	0.70880	0.00015
7.68	0.70867	0.70881	0.00015
7.63	0.70896	0.70882	0.00015
7.59	0.70898	0.70882	0.00015
7.55	0.70872	0.70879	0.00015
7.50	0.70899	0.70876	0.00016
7.46	0.70923	0.70874	0.00015
7.42	0.70845	0.70874	0.00014
7.37	0.70867	0.70874	0.00014
7.33	0.70850	0.70875	0.00014
7.29	0.70889	0.70878	0.00012
7.24	0.70884	0.70874	0.00013
7.20	0.70893	0.70875	0.00014
7.16	0.70863	0.70872	0.00014
7.11	0.70850	0.70871	0.00014
7.07	0.70879	0.70873	0.00013
7.03	0.70915	0.70874	0.00013
6.98	0.70853	0.70869	0.00010
6.94	0.70875	0.70872	0.00009
6.90	0.70879	0.70873	0.00010
6.85	0.70850	0.70875	0.00011
6.81	0.70896	0.70880	0.00011
6.77	0.70859	0.70880	0.00011
6.72	0.70856	0.70881	0.00011
6.68	0.70866	0.70887	0.00010
6.64	0.70888	0.70888	0.00010
6.59	0.70866	0.70884	0.00012
6.55	0.70881	0.70884	0.00012
6.51	0.70883	0.70882	0.00013
6.46	0.70904	0.70883	0.00013
6.42	0.70904	0.70878	0.00012
6.38	0.70893	0.70874	0.00011
6.33	0.70870	0.70870	0.00011
6.29	0.70911	0.70869	0.00012
6.25	0.70875	0.70865	0.00008
6.20	0.70853	0.70863	0.00008
6.16	0.70861	0.70870	0.00013
6.12	0.70870	0.70872	0.00013
6.07	0.70888	0.70871	0.00013
6.03	0.70859	0.70866	0.00013
5.99	0.70865	0.70865	0.00014
5.94	0.70852	0.70867	0.00014
5.90	0.70851	0.70874	0.00017
5.86	0.70880	0.70876	0.00017
5.81	0.70854	0.70879	0.00018
5.77	0.70914	0.70882	0.00017
5.73	0.70884	0.70880	0.00016
5.68	0.70858	0.70876	0.00018

5.64	0.70846	0.70881	0.00018
5.60	0.70848	0.70881	0.00018
5.55	0.70887	0.70882	0.00017
5.51	0.70921	0.70880	0.00018
5.47	0.70865	0.70873	0.00016
5.42	0.70911	0.70878	0.00018
5.38	0.70887	0.70879	0.00019
5.34	0.70895	0.70876	0.00019
5.29	0.70840	0.70873	0.00019
5.25	0.70907	0.70876	0.00018
5.21	0.70848	0.70874	0.00016
5.16	0.70864	0.70882	0.00018
5.12	0.70860	0.70887	0.00019
5.08	0.70856	0.70888	0.00018
5.03	0.70915	0.70893	0.00017
4.99	0.70923	0.70890	0.00017
4.95	0.70857	0.70885	0.00015
4.90	0.70858	0.70890	0.00014
4.86	0.70875	0.70887	0.00017
4.81	0.70888	0.70892	0.00017
4.77	0.70924	0.70887	0.00020
4.73	0.70915	0.70883	0.00018
4.68	0.70868	0.70878	0.00017
4.64	0.70910	0.70880	0.00017
4.60	0.70884	0.70879	0.00016
4.55	0.70876	0.70883	0.00019
4.51	0.70900	0.70886	0.00019
4.47	0.70835	0.70886	0.00019
4.42	0.70917	0.70891	0.00015
4.38	0.70843	0.70887	0.00014
4.34	0.70882	0.70894	0.00011
4.29	0.70868	0.70893	0.00011
4.25	0.70888	0.70898	0.00010
4.21	0.70900	0.70899	0.00011
4.16	0.70926	0.70898	0.00011
4.12	0.70903	0.70892	0.00010
4.08	0.70895	0.70891	0.00010
4.03	0.70887	0.70886	0.00014
3.99	0.70881	0.70886	0.00014
3.95	0.70912	0.70889	0.00015
3.90	0.70874	0.70882	0.00015
3.86	0.70915	0.70886	0.00016
3.82	0.70892	0.70885	0.00015
3.77	0.70868	0.70881	0.00017
3.73	0.70899	0.70886	0.00018
3.69	0.70848	0.70890	0.00021
3.64	0.70881	0.70891	0.00019
3.60	0.70912	0.70892	0.00019
3.56	0.70851	0.70887	0.00020
3.51	0.70912	0.70890	0.00018

3.47	0.70881	0.70890	0.00018
3.43	0.70909	0.70892	0.00018
3.38	0.70849	0.70890	0.00018
3.34	0.70917	0.70896	0.00016
3.30	0.70939	0.70894	0.00015
3.25	0.70863	0.70892	0.00012
3.21	0.70891	0.70892	0.00012
3.17	0.70858	0.70896	0.00014
3.12	0.70883	0.70898	0.00012
3.08	0.70911	0.70899	0.00012
3.04	0.70903	0.70895	0.00013
2.99	0.70886	0.70894	0.00013
2.95	0.70906	0.70893	0.00013
2.91	0.70902	0.70891	0.00013
2.86	0.70912	0.70890	0.00013
2.82	0.70870	0.70891	0.00013
2.78	0.70930	0.70891	0.00012
2.69	0.70872	0.70884	0.00011
2.65	0.70899	0.70885	0.00011
2.60	0.70870	0.70883	0.00010
2.56	0.70889	0.70883	0.00010
2.52	0.70883	0.70881	0.00010
2.47	0.70878	0.70876	0.00014
2.43	0.70899	0.70875	0.00014
2.39	0.70915	0.70873	0.00013
2.34	0.70876	0.70872	0.00011
2.30	0.70855	0.70867	0.00014
2.26	0.70880	0.70868	0.00014
2.21	0.70879	0.70864	0.00014
2.17	0.70874	0.70862	0.00014
2.13	0.70868	0.70865	0.00015
2.08	0.70833	0.70867	0.00016
2.04	0.70872	0.70869	0.00014
2.00	0.70880	0.70867	0.00015
1.95	0.70899	0.70865	0.00015
1.91	0.70826	0.70863	0.00013
1.87	0.70867	0.70867	0.00011
1.82	0.70847	0.70868	0.00011
1.74	0.70859	0.70871	0.00010
1.69	0.70896	0.70872	0.00009
1.65	0.70889	0.70871	0.00009
1.60	0.70856	0.70870	0.00008
1.56	0.70846	0.70871	0.00007
1.52	0.70865	0.70873	0.00005
1.47	0.70880	0.70872	0.00006
1.43	0.70869	0.70874	0.00009
1.39	0.70876	0.70877	0.00010
1.34	0.70875	0.70877	0.00010
1.30	0.70867	0.70877	0.00010
1.26	0.70890	0.70877	0.00010

1.21	0.70872	0.70876	0.00010
1.17	0.70867	0.70876	0.00010
1.13	0.70865	0.70874	0.00012
1.08	0.70857	0.70876	0.00012
1.04	0.70905	0.70875	0.00012
1.00	0.70899	0.70874	0.00011
0.95	0.70869	0.70874	0.00011
0.91	0.70879	0.70877	0.00012
0.87	0.70866	0.70874	0.00013
0.82	0.70883	0.70875	0.00013
0.78	0.70868	0.70875	0.00013
0.74	0.70845	0.70872	0.00015
0.69	0.70888	0.70873	0.00014
0.65	0.70849	0.70874	0.00014
0.61	0.70892	0.70876	0.00013
0.56	0.70900	0.70876	0.00013
0.52	0.70896	0.70876	0.00013
0.48	0.70851	0.70875	0.00013
0.43	0.70874	0.70884	0.00016
0.39	0.70886	0.70885	0.00017
0.35	0.70839	0.70885	0.00018
0.30	0.70859	0.70891	0.00015
0.26	0.70889	0.70896	0.00013
0.22	0.70877	0.70898	0.00015
0.17	0.70884	0.70903	0.00014
0.13	0.70904	0.70910	0.00014
0.09	0.70891	0.70913	0.00020
0.04	0.70935		

ARB 110.3.1 (M1)

Distance from cervix (mm)	$^{87}Sr/^{86}Sr$	10 point mov. average	2 SE on mov. average
11.41	0.70964	0.70938	0.00014
11.37	0.70943	0.70935	0.00012
11.32	0.70960	0.70935	0.00012
11.28	0.70916	0.70930	0.00012
11.23	0.70928	0.70933	0.00012
11.19	0.70920	0.70934	0.00012
11.14	0.70964	0.70934	0.00012
11.10	0.70953	0.70932	0.00010
11.05	0.70922	0.70927	0.00010
11.01	0.70906	0.70927	0.00010
10.97	0.70939	0.70929	0.00009
10.92	0.70944	0.70926	0.00010
10.88	0.70910	0.70926	0.00010
10.83	0.70941	0.70926	0.00010
10.79	0.70940	0.70923	0.00009
10.74	0.70919	0.70924	0.00010
10.70	0.70942	0.70925	0.00010
10.65	0.70906	0.70924	0.00009
10.61	0.70918	0.70925	0.00008
10.56	0.70930	0.70928	0.00009
10.52	0.70909	0.70927	0.00010
10.47	0.70944	0.70929	0.00009
10.43	0.70912	0.70926	0.00009
10.39	0.70912	0.70926	0.00009
10.34	0.70944	0.70926	0.00009
10.30	0.70929	0.70923	0.00008
10.25	0.70931	0.70920	0.00009
10.21	0.70921	0.70920	0.00009
10.16	0.70952	0.70920	0.00009
10.12	0.70912	0.70916	0.00005
10.07	0.70931	0.70914	0.00006
10.03	0.70917	0.70915	0.00006
9.98	0.70914	0.70915	0.00006
9.94	0.70914	0.70917	0.00008
9.90	0.70910	0.70918	0.00008
9.85	0.70904	0.70922	0.00010
9.81	0.70925	0.70924	0.00009
9.76	0.70919	0.70923	0.00010
9.72	0.70910	0.70925	0.00010
9.67	0.70901	0.70928	0.00010
9.63	0.70936	0.70932	0.00009
9.58	0.70915	0.70928	0.00011
9.54	0.70938	0.70927	0.00012
9.49	0.70918	0.70925	0.00012
9.45	0.70951	0.70926	0.00012
9.41	0.70929	0.70924	0.00010
9.36	0.70911	0.70925	0.00011

ARB 110.2.1 (M2)

Distance from cervix (mm)	$^{87}Sr/^{86}Sr$	10 point mov. average	2 SE on mov. average
15.13	0.71083	0.71085	0.00009
15.09	0.71054	0.71090	0.00013
15.04	0.71107	0.71094	0.00011
15.00	0.71079	0.71091	0.00010
14.96	0.71095	0.71093	0.00010
14.91	0.71091	0.71091	0.00011
14.87	0.71079	0.71090	0.00012
14.82	0.71084	0.71085	0.00016
14.78	0.71088	0.71084	0.00016
14.74	0.71085	0.71084	0.00016
14.69	0.71135	0.71079	0.00019
14.65	0.71092	0.71075	0.00015
14.61	0.71081	0.71070	0.00016
14.56	0.71102	0.71070	0.00015
14.52	0.71076	0.71064	0.00014
14.48	0.71073	0.71064	0.00014
14.43	0.71037	0.71066	0.00015
14.39	0.71071	0.71068	0.00014
14.35	0.71091	0.71064	0.00016
14.30	0.71032	0.71063	0.00015
14.26	0.71096	0.71068	0.00013
14.21	0.71044	0.71066	0.00012
14.17	0.71076	0.71068	0.00011
14.13	0.71048	0.71064	0.00013
14.08	0.71072	0.71066	0.00012
14.04	0.71093	0.71060	0.00016
14.00	0.71055	0.71053	0.00016
13.95	0.71033	0.71050	0.00016
13.91	0.71078	0.71053	0.00016
13.87	0.71080	0.71049	0.00015
13.82	0.71077	0.71047	0.00014
13.78	0.71069	0.71042	0.00013
13.73	0.71035	0.71034	0.00015
13.69	0.71067	0.71034	0.00015
13.65	0.71013	0.71033	0.00013
13.60	0.71024	0.71036	0.00013
13.56	0.71028	0.71035	0.00013
13.52	0.71061	0.71030	0.00017
13.47	0.71033	0.71023	0.00018
13.43	0.71060	0.71017	0.00020
13.39	0.71028	0.71010	0.00018
13.34	0.70993	0.71005	0.00018
13.30	0.71035	0.71009	0.00019
13.26	0.71050	0.71003	0.00018
13.21	0.71046	0.70994	0.00017
13.17	0.71015	0.70990	0.00013
13.12	0.70981	0.70986	0.00012

9.32	0.70938	0.70928	0.00010	13.08	0.70988	0.70990	0.00014
9.27	0.70942	0.70926	0.00010	13.04	0.70969	0.70989	0.00014
9.23	0.70944	0.70927	0.00011	12.99	0.70990	0.70991	0.00013
9.18	0.70899	0.70926	0.00010	12.95	0.70987	0.70986	0.00017
9.14	0.70903	0.70925	0.00012	12.91	0.71024	0.70980	0.00020
9.09	0.70912	0.70928	0.00011	12.86	0.70978	0.70974	0.00018
9.05	0.70930	0.70931	0.00011	12.82	0.70961	0.70973	0.00018
9.00	0.70935	0.70930	0.00011	12.78	0.71009	0.70975	0.00018
8.96	0.70933	0.70928	0.00012	12.73	0.70970	0.70972	0.00016
8.91	0.70940	0.70928	0.00012	12.69	0.71022	0.70974	0.00016
8.87	0.70925	0.70928	0.00012	12.64	0.70983	0.70969	0.00012
8.83	0.70950	0.70928	0.00012	12.60	0.70983	0.70972	0.00015
8.78	0.70935	0.70926	0.00011	12.56	0.70938	0.70971	0.00015
8.74	0.70887	0.70927	0.00011	12.51	0.70932	0.70977	0.00013
8.69	0.70929	0.70929	0.00008	12.47	0.70968	0.70977	0.00012
8.65	0.70947	0.70929	0.00008	12.43	0.70963	0.70976	0.00012
8.60	0.70916	0.70928	0.00008	12.38	0.70984	0.70985	0.00018
8.56	0.70919	0.70934	0.00011	12.34	0.70980	0.70979	0.00021
8.51	0.70936	0.70936	0.00011	12.30	0.70984	0.70979	0.00021
8.47	0.70939	0.70937	0.00011	12.25	0.70980	0.70982	0.00021
8.42	0.70920	0.70936	0.00011	12.21	0.71011	0.70982	0.00021
8.38	0.70935	0.70938	0.00010	12.17	0.70972	0.70974	0.00022
8.34	0.70943	0.70940	0.00011	12.12	0.70992	0.70977	0.00023
8.29	0.70907	0.70939	0.00011	12.08	0.70939	0.70974	0.00023
8.25	0.70928	0.70944	0.00009	12.03	0.70959	0.70975	0.00022
8.20	0.70943	0.70945	0.00008	11.99	0.71044	0.70975	0.00023
8.16	0.70973	0.70945	0.00008	11.95	0.70931	0.70968	0.00017
8.11	0.70937	0.70943	0.00006	11.90	0.70983	0.70974	0.00015
8.07	0.70942	0.70942	0.00007	11.86	0.71005	0.70969	0.00017
8.02	0.70932	0.70946	0.00011	11.82	0.70980	0.70962	0.00016
7.98	0.70942	0.70947	0.00010	11.77	0.70934	0.70961	0.00016
7.93	0.70959	0.70946	0.00011	11.73	0.71006	0.70965	0.00015
7.89	0.70928	0.70946	0.00011	11.69	0.70959	0.70963	0.00013
7.84	0.70952	0.70948	0.00010	11.64	0.70952	0.70964	0.00013
7.80	0.70942	0.70946	0.00011	11.60	0.70954	0.70963	0.00014
7.76	0.70942	0.70946	0.00011	11.55	0.70974	0.70957	0.00020
7.71	0.70956	0.70947	0.00011	11.51	0.70995	0.70955	0.00020
7.67	0.70924	0.70944	0.00012	11.47	0.70931	0.70952	0.00018
7.62	0.70981	0.70944	0.00012	11.42	0.70935	0.70951	0.00018
7.58	0.70942	0.70944	0.00011	11.38	0.70966	0.70955	0.00018
7.53	0.70932	0.70943	0.00011	11.34	0.70982	0.70952	0.00018
7.49	0.70966	0.70941	0.00012	11.29	0.70983	0.70948	0.00017
7.44	0.70943	0.70939	0.00011	11.25	0.70970	0.70945	0.00016
7.40	0.70929	0.70940	0.00011	11.21	0.70943	0.70941	0.00015
7.35	0.70945	0.70943	0.00011	11.16	0.70890	0.70945	0.00018
7.31	0.70954	0.70946	0.00014	11.12	0.70956	0.70951	0.00013
7.27	0.70920	0.70944	0.00014	11.07	0.70965	0.70949	0.00013
7.22	0.70931	0.70946	0.00013	11.03	0.70925	0.70942	0.00016
7.18	0.70974	0.70946	0.00013	10.99	0.70970	0.70947	0.00016
7.13	0.70934	0.70946	0.00013	10.94	0.70934	0.70946	0.00016

7.09	0.70914	0.70949	0.00013
7.04	0.70945	0.70953	0.00011
7.00	0.70954	0.70957	0.00012
6.95	0.70956	0.70957	0.00012
6.91	0.70981	0.70955	0.00013
6.86	0.70935	0.70952	0.00012
6.82	0.70933	0.70953	0.00011
6.78	0.70930	0.70954	0.00011
6.73	0.70976	0.70959	0.00010
6.69	0.70964	0.70959	0.00010
6.64	0.70956	0.70961	0.00012
6.60	0.70981	0.70961	0.00012
6.55	0.70958	0.70957	0.00012
6.51	0.70934	0.70958	0.00013
6.46	0.70954	0.70961	0.00011
6.42	0.70945	0.70960	0.00012
6.37	0.70940	0.70964	0.00012
6.33	0.70980	0.70965	0.00011
6.28	0.70976	0.70960	0.00013
6.24	0.70990	0.70956	0.00013
6.20	0.70952	0.70955	0.00012
6.15	0.70938	0.70957	0.00013
6.11	0.70974	0.70960	0.00013
6.06	0.70961	0.70955	0.00014
6.02	0.70949	0.70956	0.00014
5.97	0.70986	0.70959	0.00015
5.93	0.70947	0.70956	0.00013
5.88	0.70932	0.70956	0.00013
5.84	0.70931	0.70961	0.00013
5.79	0.70978	0.70962	0.00012
5.75	0.70975	0.70963	0.00012
5.71	0.70972	0.70962	0.00012
5.66	0.70923	0.70957	0.00013
5.62	0.70967	0.70964	0.00012
5.57	0.70975	0.70963	0.00012
5.53	0.70958	0.70962	0.00012
5.48	0.70951	0.70967	0.00015
5.44	0.70980	0.70967	0.00015
5.39	0.70939	0.70966	0.00015
5.35	0.70985	0.70966	0.00014
5.30	0.70966	0.70965	0.00014
5.26	0.70931	0.70966	0.00014
5.22	0.70989	0.70970	0.00012
5.17	0.70956	0.70974	0.00016
5.13	0.70970	0.70973	0.00016
5.08	0.71005	0.70976	0.00017
5.04	0.70948	0.70974	0.00016
4.99	0.70969	0.70977	0.00015
4.95	0.70943	0.70981	0.00016
4.90	0.70976	0.70985	0.00014

10.90	0.70939	0.70947	0.00016
10.86	0.70959	0.70946	0.00016
10.81	0.70924	0.70943	0.00016
10.77	0.70984	0.70946	0.00015
10.73	0.70954	0.70941	0.00013
10.68	0.70936	0.70940	0.00012
10.64	0.70899	0.70940	0.00012
10.60	0.70970	0.70942	0.00010
10.55	0.70962	0.70940	0.00008
10.51	0.70941	0.70936	0.00006
10.46	0.70926	0.70936	0.00006
10.42	0.70937	0.70940	0.00008
10.38	0.70947	0.70945	0.00011
10.33	0.70935	0.70946	0.00011
10.29	0.70945	0.70949	0.00012
10.25	0.70933	0.70948	0.00012
10.20	0.70921	0.70949	0.00011
10.16	0.70950	0.70954	0.00010
10.12	0.70929	0.70958	0.00013
10.07	0.70942	0.70963	0.00011
10.03	0.70966	0.70962	0.00012
9.98	0.70978	0.70964	0.00013
9.94	0.70962	0.70957	0.00016
9.90	0.70961	0.70958	0.00017
9.85	0.70940	0.70956	0.00017
9.81	0.70939	0.70960	0.00018
9.77	0.70973	0.70953	0.00025
9.72	0.70990	0.70950	0.00025
9.68	0.70976	0.70947	0.00023
9.64	0.70936	0.70948	0.00024
9.59	0.70986	0.70948	0.00024
9.55	0.70907	0.70944	0.00022
9.51	0.70975	0.70952	0.00022
9.46	0.70936	0.70950	0.00021
9.42	0.70982	0.70951	0.00021
9.37	0.70872	0.70947	0.00020
9.33	0.70942	0.70955	0.00012
9.29	0.70961	0.70951	0.00014
9.24	0.70985	0.70951	0.00014
9.20	0.70935	0.70945	0.00013
9.16	0.70942	0.70951	0.00016
9.11	0.70985	0.70954	0.00017
9.07	0.70963	0.70948	0.00016
9.03	0.70946	0.70942	0.00018
8.98	0.70938	0.70944	0.00018
8.94	0.70948	0.70945	0.00018
8.89	0.70911	0.70947	0.00019
8.85	0.70959	0.70951	0.00017
8.81	0.70921	0.70954	0.00019
8.76	0.70993	0.70960	0.00017

4.86	0.70971	0.70987	0.00014	8.72	0.70975	0.70955	0.00016
4.81	0.70976	0.70988	0.00013	8.68	0.70929	0.70955	0.00016
4.77	0.71025	0.70989	0.00013	8.63	0.70904	0.70961	0.00017
4.72	0.70951	0.70983	0.00011	8.59	0.70965	0.70963	0.00014
4.68	0.70999	0.70984	0.00010	8.55	0.70950	0.70960	0.00015
4.64	0.70983	0.70980	0.00011	8.50	0.70964	0.70962	0.00015
4.59	0.70976	0.70981	0.00012	8.46	0.70950	0.70959	0.00016
4.55	0.71012	0.70978	0.00014	8.42	0.70990	0.70958	0.00016
4.50	0.70984	0.70972	0.00013	8.37	0.70977	0.70954	0.00014
4.46	0.70998	0.70970	0.00013	8.33	0.70944	0.70949	0.00014
4.41	0.70980	0.70967	0.00011	8.28	0.70978	0.70952	0.00015
4.37	0.70983	0.70966	0.00011	8.24	0.70992	0.70948	0.00014
4.32	0.70967	0.70968	0.00012	8.20	0.70918	0.70942	0.00010
4.28	0.70962	0.70968	0.00012	8.15	0.70939	0.70945	0.00008
4.23	0.70952	0.70966	0.00014	8.11	0.70965	0.70944	0.00009
4.19	0.71001	0.70967	0.00013	8.07	0.70941	0.70945	0.00009
4.15	0.70945	0.70962	0.00011	8.02	0.70941	0.70944	0.00010
4.10	0.70949	0.70965	0.00010	7.98	0.70946	0.70944	0.00010
4.06	0.70967	0.70967	0.00010	7.94	0.70928	0.70936	0.00017
4.01	0.70961	0.70968	0.00010	7.89	0.70971	0.70941	0.00018
3.97	0.70978	0.70967	0.00011	7.85	0.70941	0.70933	0.00019
3.92	0.70998	0.70967	0.00011	7.80	0.70932	0.70932	0.00019
3.88	0.70968	0.70964	0.00009	7.76	0.70943	0.70936	0.00020
3.83	0.70937	0.70965	0.00009	7.72	0.70933	0.70937	0.00021
3.79	0.70965	0.70967	0.00007	7.67	0.70972	0.70936	0.00021
3.74	0.70957	0.70970	0.00009	7.63	0.70933	0.70932	0.00019
3.70	0.70971	0.70973	0.00009	7.59	0.70937	0.70932	0.00019
3.66	0.70966	0.70973	0.00009	7.54	0.70873	0.70932	0.00019
3.61	0.70981	0.70975	0.00010	7.50	0.70970	0.70938	0.00014
3.57	0.70947	0.70974	0.00010	7.46	0.70895	0.70938	0.00014
3.52	0.70975	0.70975	0.00009	7.41	0.70934	0.70948	0.00014
3.48	0.70976	0.70978	0.00011	7.37	0.70966	0.70947	0.00014
3.43	0.70972	0.70978	0.00011	7.33	0.70957	0.70948	0.00014
3.39	0.70955	0.70976	0.00012	7.28	0.70926	0.70944	0.00016
3.34	0.70999	0.70978	0.00011	7.24	0.70933	0.70942	0.00017
3.30	0.70983	0.70977	0.00010	7.19	0.70930	0.70942	0.00017
3.25	0.70974	0.70975	0.00010	7.15	0.70933	0.70944	0.00017
3.21	0.70993	0.70978	0.00011	7.11	0.70937	0.70945	0.00016
3.16	0.70966	0.70976	0.00010	7.06	0.70972	0.70946	0.00016
3.12	0.70958	0.70978	0.00010	7.02	0.70989	0.70943	0.00015
3.08	0.71005	0.70981	0.00010	6.98	0.70928	0.70938	0.00011
3.03	0.70976	0.70981	0.00010	6.93	0.70973	0.70937	0.00011
2.99	0.70955	0.70982	0.00010	6.89	0.70917	0.70933	0.00008
2.94	0.70975	0.70985	0.00008	6.85	0.70908	0.70934	0.00008
2.90	0.70984	0.70988	0.00008	6.80	0.70934	0.70937	0.00005
2.85	0.70968	0.70989	0.00008	6.76	0.70951	0.70939	0.00007
2.81	0.70997	0.70992	0.00007	6.71	0.70944	0.70932	0.00013
2.76	0.70971	0.70988	0.00010	6.67	0.70945	0.70931	0.00012
2.72	0.70986	0.70989	0.00009	6.63	0.70938	0.70930	0.00012
2.67	0.70994	0.70989	0.00009	6.58	0.70937	0.70929	0.00012

2.63	0.71007	0.70986	0.00010	6.54	0.70927	0.70933	0.00015
2.59	0.70985	0.70980	0.00012	6.50	0.70929	0.70932	0.00016
2.54	0.70981	0.70978	0.00013	6.45	0.70926	0.70933	0.00016
2.50	0.71005	0.70977	0.00013	6.41	0.70935	0.70934	0.00016
2.45	0.70998	0.70974	0.00011	6.37	0.70959	0.70932	0.00016
2.41	0.70999	0.70972	0.00010	6.32	0.70883	0.70931	0.00015
2.36	0.70959	0.70970	0.00008	6.28	0.70935	0.70935	0.00011
2.32	0.70981	0.70971	0.00008	6.24	0.70935	0.70939	0.00015
2.27	0.70980	0.70971	0.00008	6.19	0.70926	0.70941	0.00015
2.23	0.70971	0.70971	0.00008	6.15	0.70977	0.70940	0.00015
2.18	0.70939	0.70972	0.00009	6.10	0.70914	0.70937	0.00013
2.14	0.70971	0.70975	0.00004	6.06	0.70935	0.70942	0.00013
2.09	0.70966	0.70975	0.00005	6.02	0.70937	0.70941	0.00013
2.05	0.70978	0.70975	0.00005	5.97	0.70921	0.70946	0.00016
2.01	0.70978	0.70974	0.00005	5.93	0.70947	0.70948	0.00015
1.96	0.70981	0.70974	0.00005	5.89	0.70917	0.70948	0.00015
1.92	0.70962	0.70975	0.00006	5.84	0.70980	0.70951	0.00013
1.87	0.70982	0.70976	0.00006	5.80	0.70956	0.70944	0.00014
1.83	0.70981	0.70977	0.00006	5.76	0.70917	0.70943	0.00014
1.78	0.70980	0.70978	0.00007	5.71	0.70946	0.70942	0.00015
1.74	0.70972	0.70978	0.00007	5.67	0.70959	0.70941	0.00015
1.69	0.70964	0.70978	0.00007	5.62	0.70930	0.70941	0.00014
1.65	0.70974	0.70980	0.00007	5.58	0.70987	0.70939	0.00015
1.60	0.70966	0.70980	0.00007	5.54	0.70940	0.70935	0.00011
1.56	0.70977	0.70984	0.00008	5.49	0.70946	0.70930	0.00014
1.52	0.70995	0.70985	0.00008	5.45	0.70951	0.70930	0.00014
1.47	0.70969	0.70985	0.00008	5.41	0.70909	0.70929	0.00014
1.43	0.70986	0.70987	0.00007	5.36	0.70941	0.70930	0.00013
1.38	0.70995	0.70986	0.00008	5.32	0.70908	0.70930	0.00014
1.34	0.70978	0.70984	0.00008	5.28	0.70943	0.70933	0.00013
1.29	0.70970	0.70983	0.00009	5.23	0.70951	0.70930	0.00014
1.25	0.70991	0.70985	0.00008	5.19	0.70915	0.70933	0.00017
1.20	0.70971	0.70983	0.00009	5.15	0.70950	0.70936	0.00017
1.16	0.71009	0.70982	0.00009	5.10	0.70889	0.70932	0.00017
1.11	0.70988	0.70981	0.00007	5.06	0.70948	0.70939	0.00014
1.07	0.70994	0.70982	0.00008	5.01	0.70937	0.70938	0.00014
1.03	0.70988	0.70985	0.00011	4.97	0.70916	0.70938	0.00014
0.98	0.70973	0.70984	0.00011	4.93	0.70946	0.70939	0.00013
0.94	0.70977	0.70985	0.00010	4.88	0.70939	0.70941	0.00014
0.89	0.70966	0.70989	0.00011	4.84	0.70906	0.70939	0.00014
0.85	0.70992	0.70988	0.00012	4.80	0.70984	0.70944	0.00013
0.80	0.70970	0.70990	0.00012	4.75	0.70940	0.70943	0.00011
0.76	0.70968	0.70988	0.00014	4.71	0.70917	0.70942	0.00012
0.71	0.70998	0.70990	0.00013	4.67	0.70952	0.70942	0.00012
0.67	0.70999	0.70982	0.00019	4.62	0.70940	0.70940	0.00012
0.62	0.71017	0.70977	0.00020	4.58	0.70941	0.70938	0.00013
0.58	0.70979	0.70969	0.00019	4.53	0.70924	0.70937	0.00013
0.53	0.70989	0.70967	0.00019	4.49	0.70962	0.70937	0.00012
0.49	0.71010	0.70963	0.00019	4.45	0.70927	0.70927	0.00019
0.45	0.70961	0.70958	0.00017	4.40	0.70955	0.70929	0.00019

0.40	0.71008	0.70957	0.00018
0.36	0.70951	0.70950	0.00013
0.31	0.70983	0.70950	0.00014
0.27	0.70920	0.70943	0.00011
0.22	0.70951	0.70949	0.00008
0.18	0.70934	0.70948	0.00010
0.13	0.70964	0.70955	0.00009
0.09	0.70945		

4.36	0.70975	0.70928	0.00019
4.32	0.70928	0.70931	0.00022
4.27	0.70918	0.70934	0.00023
4.23	0.70932	0.70940	0.00025
4.19	0.70917	0.70941	0.00025
4.14	0.70934	0.70946	0.00024
4.10	0.70927	0.70945	0.00025
4.06	0.70859	0.70948	0.00024
4.01	0.70948	0.70956	0.00015
3.97	0.70945	0.70959	0.00015
3.92	0.71003	0.70960	0.00015
3.88	0.70954	0.70953	0.00012
3.84	0.70985	0.70951	0.00013
3.79	0.70939	0.70949	0.00011
3.75	0.70967	0.70945	0.00014
3.71	0.70928	0.70942	0.00013
3.66	0.70951	0.70942	0.00013
3.62	0.70939	0.70944	0.00014
3.58	0.70977	0.70949	0.00017
3.53	0.70959	0.70948	0.00016
3.49	0.70928	0.70950	0.00018
3.44	0.70939	0.70946	0.00022
3.40	0.70961	0.70945	0.00022
3.36	0.70906	0.70945	0.00022
3.31	0.70936	0.70952	0.00021
3.27	0.70927	0.70953	0.00020
3.23	0.70966	0.70957	0.00020
3.18	0.70994	0.70957	0.00020
3.14	0.70963	0.70952	0.00018
3.10	0.70984	0.70946	0.00020
3.05	0.70886	0.70941	0.00018
3.01	0.70925	0.70948	0.00013
2.96	0.70966	0.70950	0.00012
2.92	0.70969	0.70946	0.00013
2.88	0.70952	0.70946	0.00012
2.83	0.70969	0.70946	0.00013
2.79	0.70961	0.70944	0.00012
2.75	0.70945	0.70943	0.00011
2.70	0.70906	0.70941	0.00012
2.66	0.70932	0.70946	0.00010
2.62	0.70952	0.70944	0.00012
2.57	0.70951	0.70943	0.00012
2.53	0.70924	0.70946	0.00014
2.49	0.70965	0.70945	0.00014
2.44	0.70953	0.70944	0.00014
2.40	0.70952	0.70942	0.00014
2.35	0.70955	0.70939	0.00014
2.31	0.70917	0.70936	0.00014
2.27	0.70959	0.70937	0.00014
2.22	0.70910	0.70936	0.00014

2.18	0.70946	0.70943	0.00014
2.14	0.70976	0.70944	0.00014
2.09	0.70917	0.70945	0.00016
2.05	0.70952	0.70951	0.00015
2.01	0.70939	0.70955	0.00017
1.96	0.70914	0.70960	0.00018
1.92	0.70928	0.70963	0.00016
1.87	0.70925	0.70967	0.00014
1.83	0.70957	0.70970	0.00010
1.79	0.70971	0.70970	0.00010
1.74	0.70957	0.70964	0.00017
1.70	0.70989	0.70959	0.00020
1.66	0.70974	0.70959	0.00020
1.61	0.70992	0.70953	0.00021
1.57	0.70993	0.70952	0.00021
1.53	0.70948	0.70944	0.00019
1.48	0.70963	0.70940	0.00020
1.44	0.70960	0.70938	0.00020
1.40	0.70957	0.70938	0.00020
1.35	0.70902	0.70938	0.00020
1.31	0.70912	0.70946	0.00019
1.26	0.70986	0.70952	0.00018
1.22	0.70914	0.70946	0.00017
1.18	0.70981	0.70948	0.00016
1.13	0.70917	0.70941	0.00016
1.09	0.70909	0.70947	0.00016
1.05	0.70947	0.70950	0.00014
1.00	0.70955	0.70946	0.00016
0.96	0.70960	0.70942	0.00017
0.92	0.70974	0.70941	0.00017
0.87	0.70974	0.70942	0.00018
0.83	0.70924	0.70936	0.00017
0.78	0.70935	0.70937	0.00017
0.74	0.70911	0.70939	0.00017
0.70	0.70977	0.70945	0.00017
0.65	0.70943	0.70940	0.00015
0.61	0.70911	0.70938	0.00016
0.57	0.70914	0.70941	0.00015
0.52	0.70946	0.70947	0.00014
0.48	0.70984	0.70946	0.00015
0.44	0.70914	0.70940	0.00013
0.39	0.70937	0.70943	0.00012
0.35	0.70954	0.70943	0.00013
0.31	0.70967	0.70942	0.00013
0.26	0.70930	0.70937	0.00012
0.22	0.70920	0.70939	0.00014
0.17	0.70948	0.70944	0.00014
0.13	0.70972	0.70942	0.00017
0.09	0.70933	0.70928	0.00005
0.04	0.70922		

ARB 110.4.1 (M3)

Distance from cervix (mm)	$^{87}Sr/^{86}Sr$	10 point mov. average	2 SE on mov. average
26.96	0.71026	0.71039	0.00016
26.92	0.71004	0.71037	0.00017
26.87	0.71008	0.71040	0.00015
26.83	0.71040	0.71039	0.00017
26.78	0.71043	0.71034	0.00019
26.74	0.71059	0.71036	0.00020
26.70	0.71036	0.71031	0.00020
26.65	0.71061	0.71025	0.00023
26.61	0.71022	0.71019	0.00022
26.57	0.71086	0.71019	0.00022
26.52	0.71010	0.71018	0.00020
26.48	0.71038	0.71026	0.00026
26.43	0.70991	0.71028	0.00026
26.39	0.70998	0.71023	0.00030
26.35	0.71063	0.71026	0.00030
26.30	0.71007	0.71019	0.00029
26.26	0.70975	0.71018	0.00029
26.22	0.70998	0.71022	0.00028
26.17	0.71022	0.71023	0.00027
26.13	0.71075	0.71023	0.00027
26.08	0.71098	0.71013	0.00026
26.04	0.71053	0.71008	0.00020
26.00	0.70947	0.71005	0.00018
25.95	0.71019	0.71013	0.00012
25.91	0.70996	0.71013	0.00012
25.87	0.70997	0.71016	0.00012
25.82	0.71016	0.71015	0.00013
25.78	0.71006	0.71010	0.00016
25.73	0.71022	0.71017	0.00021
25.69	0.70979	0.71018	0.00021
25.65	0.71049	0.71024	0.00019
25.60	0.71017	0.71024	0.00020
25.56	0.71030	0.71026	0.00020
25.52	0.71015	0.71029	0.00021
25.47	0.71028	0.71024	0.00024
25.43	0.70987	0.71020	0.00025
25.38	0.70967	0.71022	0.00024
25.34	0.71078	0.71026	0.00021
25.30	0.71027	0.71019	0.00018
25.25	0.71039	0.71019	0.00018
25.21	0.71050	0.71019	0.00018
25.17	0.71037	0.71014	0.00017
25.12	0.71058	0.71018	0.00020
25.08	0.70971	0.71015	0.00018
25.03	0.70989	0.71023	0.00017
24.99	0.71007	0.71026	0.00015
24.95	0.71008	0.71024	0.00017

24.90	0.71003	0.71023	0.00017
24.86	0.71027	0.71027	0.00017
24.82	0.71041	0.71026	0.00017
24.77	0.70999	0.71019	0.00020
24.73	0.71073	0.71021	0.00019
24.68	0.71033	0.71009	0.00019
24.64	0.71049	0.71008	0.00019
24.60	0.71016	0.70999	0.00018
24.55	0.70987	0.71000	0.00018
24.51	0.71004	0.71002	0.00018
24.47	0.71044	0.70999	0.00019
24.42	0.71013	0.70992	0.00016
24.38	0.70969	0.70992	0.00016
24.33	0.71022	0.70995	0.00016
24.29	0.70957	0.70992	0.00015
24.25	0.71014	0.70999	0.00014
24.20	0.70969	0.70996	0.00014
24.16	0.71024	0.70999	0.00013
24.12	0.71007	0.70998	0.00012
24.07	0.70971	0.70999	0.00013
24.03	0.70974	0.71003	0.00011
23.98	0.71011	0.71003	0.00011
23.94	0.71006	0.70998	0.00014
23.90	0.70991	0.71001	0.00015
23.85	0.71028	0.71003	0.00016
23.81	0.70975	0.70993	0.00021
23.77	0.71007	0.70995	0.00021
23.72	0.71006	0.70995	0.00021
23.68	0.71021	0.70996	0.00021
23.63	0.71008	0.70993	0.00021
23.59	0.70974	0.70993	0.00021
23.55	0.70962	0.70999	0.00022
23.50	0.71035	0.71002	0.00020
23.46	0.71016	0.70997	0.00019
23.41	0.70924	0.70995	0.00019
23.37	0.70998	0.71007	0.00012
23.33	0.71008	0.71005	0.00013
23.28	0.71018	0.71004	0.00013
23.24	0.70989	0.71003	0.00013
23.20	0.71008	0.71006	0.00012
23.15	0.71036	0.71004	0.00013
23.11	0.70986	0.71005	0.00014
23.06	0.70990	0.71010	0.00015
23.02	0.70997	0.71009	0.00016
22.98	0.71040	0.71011	0.00015
22.93	0.70983	0.71007	0.00014
22.89	0.70994	0.71009	0.00013
22.85	0.71011	0.71016	0.00016
22.80	0.71011	0.71016	0.00016
22.76	0.70992	0.71016	0.00016

22.71	0.71045	0.71016	0.00016
22.67	0.71040	0.71013	0.00014
22.63	0.70979	0.71008	0.00013
22.58	0.71012	0.71011	0.00012
22.54	0.71001	0.71011	0.00012
22.50	0.71007	0.71010	0.00012
22.45	0.71057	0.71010	0.00012
22.41	0.71013	0.71003	0.00007
22.36	0.71010	0.71001	0.00007
22.32	0.70993	0.71001	0.00007
22.28	0.71019	0.71002	0.00007
22.23	0.70992	0.71001	0.00006
22.19	0.71003	0.71009	0.00015
22.15	0.71012	0.71007	0.00016
22.10	0.70991	0.71008	0.00017
22.06	0.71007	0.71010	0.00016
22.01	0.70991	0.71011	0.00017
21.97	0.70989	0.71017	0.00017
21.93	0.71009	0.71016	0.00018
21.88	0.71004	0.71017	0.00018
21.84	0.71009	0.71014	0.00020
21.80	0.71073	0.71013	0.00020
21.75	0.70983	0.71008	0.00015
21.71	0.71030	0.71011	0.00014
21.66	0.71003	0.71008	0.00014
21.62	0.71021	0.71012	0.00016
21.58	0.71047	0.71011	0.00016
21.53	0.70980	0.71008	0.00014
21.49	0.71021	0.71011	0.00012
21.45	0.70972	0.71009	0.00012
21.40	0.70998	0.71015	0.00009
21.36	0.71022	0.71019	0.00010
21.31	0.71017	0.71020	0.00010
21.27	0.70996	0.71018	0.00011
21.23	0.71044	0.71021	0.00010
21.18	0.71009	0.71019	0.00009
21.14	0.71021	0.71021	0.00008
21.10	0.71005	0.71022	0.00009
21.05	0.71009	0.71028	0.00012
21.01	0.71029	0.71036	0.00016
20.96	0.71042	0.71035	0.00016
20.92	0.71029	0.71033	0.00016
20.88	0.70998	0.71032	0.00016
20.83	0.71028	0.71034	0.00015
20.79	0.71020	0.71037	0.00016
20.75	0.71024	0.71036	0.00016
20.70	0.71032	0.71032	0.00019
20.66	0.71066	0.71029	0.00020
20.61	0.71087	0.71027	0.00018
20.57	0.71024	0.71023	0.00013

20.53	0.71019	0.71024	0.00013
20.48	0.71020	0.71026	0.00014
20.44	0.71018	0.71031	0.00017
20.40	0.71058	0.71035	0.00017
20.35	0.71010	0.71034	0.00016
20.31	0.70985	0.71040	0.00017
20.26	0.71007	0.71042	0.00015
20.22	0.71044	0.71044	0.00013
20.18	0.71042	0.71047	0.00014
20.13	0.71035	0.71053	0.00017
20.09	0.71040	0.71059	0.00019
20.04	0.71075	0.71065	0.00020
20.00	0.71052	0.71066	0.00020
19.96	0.71045	0.71070	0.00021
19.91	0.71075	0.71077	0.00022
19.87	0.71004	0.71082	0.00024
19.83	0.71033	0.71086	0.00018
19.78	0.71068	0.71090	0.00015
19.74	0.71099	0.71089	0.00015
19.69	0.71102	0.71091	0.00017
19.65	0.71096	0.71092	0.00017
19.61	0.71086	0.71093	0.00017
19.56	0.71096	0.71097	0.00019
19.52	0.71115	0.71098	0.00019
19.48	0.71123	0.71096	0.00018
19.43	0.71046	0.71093	0.00017
19.39	0.71068	0.71098	0.00014
19.34	0.71060	0.71097	0.00015
19.30	0.71121	0.71094	0.00019
19.26	0.71109	0.71090	0.00018
19.21	0.71102	0.71088	0.00017
19.17	0.71130	0.71088	0.00017
19.13	0.71110	0.71082	0.00015
19.08	0.71088	0.71077	0.00014
19.04	0.71094	0.71073	0.00015
18.99	0.71093	0.71070	0.00014
18.95	0.71059	0.71067	0.00013
18.91	0.71032	0.71065	0.00014
18.86	0.71082	0.71066	0.00014
18.82	0.71092	0.71063	0.00013
18.78	0.71100	0.71057	0.00013
18.73	0.71070	0.71055	0.00010
18.69	0.71056	0.71055	0.00011
18.64	0.71047	0.71054	0.00011
18.60	0.71063	0.71057	0.00012
18.56	0.71068	0.71054	0.00013
18.51	0.71037	0.71052	0.00013
18.47	0.71038	0.71053	0.00013
18.43	0.71058	0.71055	0.00012
18.38	0.71030	0.71061	0.00019

18.34	0.71078	0.71062	0.00019
18.29	0.71076	0.71059	0.00018
18.25	0.71042	0.71057	0.00018
18.21	0.71083	0.71060	0.00018
18.16	0.71029	0.71062	0.00020
18.12	0.71048	0.71065	0.00019
18.08	0.71046	0.71069	0.00019
18.03	0.71057	0.71070	0.00019
17.99	0.71126	0.71071	0.00019
17.94	0.71032	0.71061	0.00016
17.90	0.71052	0.71063	0.00015
17.86	0.71052	0.71060	0.00017
17.81	0.71077	0.71057	0.00019
17.77	0.71105	0.71054	0.00018
17.73	0.71049	0.71049	0.00015
17.68	0.71095	0.71049	0.00015
17.64	0.71053	0.71050	0.00016
17.59	0.71071	0.71048	0.00016
17.55	0.71028	0.71042	0.00018
17.51	0.71048	0.71044	0.00017
17.46	0.71023	0.71043	0.00017
17.42	0.71018	0.71045	0.00017
17.38	0.71046	0.71052	0.00018
17.33	0.71058	0.71049	0.00019
17.29	0.71045	0.71048	0.00019
17.24	0.71107	0.71046	0.00020
17.20	0.71039	0.71041	0.00015
17.16	0.71007	0.71044	0.00016
17.11	0.71054	0.71045	0.00015
17.07	0.71038	0.71042	0.00016
17.03	0.71035	0.71043	0.00016
16.98	0.71089	0.71041	0.00016
16.94	0.71015	0.71035	0.00012
16.89	0.71050	0.71038	0.00012
16.85	0.71030	0.71037	0.00011
16.81	0.71058	0.71036	0.00012
16.76	0.71069	0.71031	0.00012
16.72	0.71014	0.71027	0.00009
16.67	0.71021	0.71028	0.00009
16.63	0.71049	0.71032	0.00010
16.59	0.71019	0.71029	0.00010
16.54	0.71027	0.71027	0.00011
16.50	0.71047	0.71030	0.00012
16.46	0.71037	0.71025	0.00013
16.41	0.71019	0.71021	0.00014
16.37	0.71006	0.71022	0.00014
16.32	0.71034	0.71021	0.00014
16.28	0.71024	0.71025	0.00018
16.24	0.71059	0.71026	0.00018
16.19	0.71022	0.71026	0.00018

16.15	0.70999	0.71027	0.00018
16.11	0.71052	0.71034	0.00018
16.06	0.71002	0.71032	0.00018
16.02	0.70993	0.71031	0.00019
15.97	0.71025	0.71034	0.00017
15.93	0.71000	0.71037	0.00017
15.89	0.71076	0.71045	0.00017
15.84	0.71037	0.71037	0.00018
15.80	0.71055	0.71042	0.00020
15.76	0.71034	0.71043	0.00020
15.71	0.71066	0.71042	0.00021
15.67	0.71035	0.71040	0.00020
15.62	0.70991	0.71044	0.00021
15.58	0.71022	0.71054	0.00019
15.54	0.71058	0.71056	0.00018
15.49	0.71074	0.71061	0.00021
15.45	0.70996	0.71057	0.00021
15.41	0.71084	0.71061	0.00017
15.36	0.71065	0.71055	0.00017
15.32	0.71030	0.71051	0.00018
15.27	0.71046	0.71056	0.00018
15.23	0.71074	0.71057	0.00018
15.19	0.71093	0.71051	0.00019
15.14	0.71039	0.71041	0.00021
15.10	0.71103	0.71042	0.00021
15.06	0.71040	0.71033	0.00016
15.01	0.71032	0.71037	0.00018
14.97	0.71026	0.71043	0.00021
14.92	0.71025	0.71049	0.00022
14.88	0.71082	0.71053	0.00022
14.84	0.71054	0.71053	0.00022
14.79	0.71020	0.71057	0.00023
14.75	0.70988	0.71057	0.00023
14.71	0.71048	0.71061	0.00018
14.66	0.71016	0.71067	0.00020
14.62	0.71074	0.71070	0.00017
14.57	0.71095	0.71068	0.00017
14.53	0.71083	0.71064	0.00016
14.49	0.71071	0.71065	0.00018
14.44	0.71077	0.71068	0.00019
14.40	0.71092	0.71066	0.00019
14.36	0.71026	0.71057	0.00022
14.31	0.71030	0.71059	0.00021
14.27	0.71104	0.71064	0.00020
14.22	0.71051	0.71066	0.00023
14.18	0.71050	0.71066	0.00023
14.14	0.71051	0.71066	0.00023
14.09	0.71099	0.71067	0.00023
14.05	0.71101	0.71066	0.00022
14.01	0.71056	0.71059	0.00022

13.96	0.70998	0.71056	0.00023
13.92	0.71055	0.71062	0.00019
13.87	0.71077	0.71058	0.00021
13.83	0.71127	0.71053	0.00021
13.79	0.71049	0.71046	0.00014
13.74	0.71052	0.71044	0.00014
13.70	0.71059	0.71046	0.00015
13.66	0.71091	0.71046	0.00015
13.61	0.71030	0.71039	0.00011
13.57	0.71025	0.71034	0.00017
13.52	0.71057	0.71034	0.00017
13.48	0.71018	0.71028	0.00017
13.44	0.71026	0.71025	0.00020
13.39	0.71055	0.71024	0.00020
13.35	0.71030	0.71019	0.00019
13.30	0.71069	0.71017	0.00019
13.26	0.71057	0.71011	0.00015
13.22	0.71027	0.71007	0.00011
13.17	0.70974	0.71005	0.00010
13.13	0.71024	0.71010	0.00007
13.09	0.71006	0.71006	0.00007
13.04	0.70982	0.71006	0.00007
13.00	0.71012	0.71008	0.00006
12.95	0.71005	0.71006	0.00006
12.91	0.71012	0.71006	0.00006
12.87	0.71009	0.71005	0.00006
12.82	0.71015	0.71006	0.00006
12.78	0.71008	0.71006	0.00006
12.74	0.71023	0.71006	0.00006
12.69	0.70993	0.71003	0.00005
12.65	0.70998	0.71003	0.00005
12.60	0.71002	0.71005	0.00006
12.56	0.71001	0.71005	0.00006
12.52	0.71004	0.71001	0.00011
12.47	0.71000	0.71002	0.00011
12.43	0.71019	0.71009	0.00018
12.39	0.71011	0.71008	0.00018
12.34	0.71006	0.71010	0.00018
12.30	0.71000	0.71004	0.00022
12.25	0.70989	0.71005	0.00022
12.21	0.71020	0.71012	0.00024
12.17	0.71005	0.71012	0.00024
12.12	0.70959	0.71013	0.00024
12.08	0.71010	0.71021	0.00021
12.04	0.71071	0.71013	0.00028
11.99	0.71007	0.71001	0.00027
11.95	0.71031	0.71000	0.00027
11.90	0.70951	0.70997	0.00026
11.86	0.71006	0.71001	0.00024
11.82	0.71060	0.71004	0.00025

11.77	0.71021	0.70999	0.00022
11.73	0.71017	0.70998	0.00022
11.69	0.71036	0.70996	0.00021
11.64	0.70929	0.70999	0.00023
11.60	0.70951	0.71008	0.00018
11.55	0.71002	0.71009	0.00016
11.51	0.70998	0.71009	0.00016
11.47	0.70993	0.71015	0.00018
11.42	0.71032	0.71020	0.00019
11.38	0.71008	0.71017	0.00019
11.34	0.71018	0.71010	0.00026
11.29	0.70998	0.71007	0.00026
11.25	0.71057	0.71002	0.00028
11.20	0.71020	0.71003	0.00029
11.16	0.70962	0.70995	0.00031
11.12	0.71003	0.70998	0.00030
11.07	0.71055	0.71000	0.00030
11.03	0.71053	0.70998	0.00028
10.99	0.71001	0.71000	0.00031
10.94	0.70930	0.70999	0.00031
10.90	0.70989	0.71012	0.00029
10.85	0.70956	0.71013	0.00029
10.81	0.71061	0.71018	0.00026
10.77	0.70943	0.71015	0.00025
10.72	0.70991	0.71016	0.00024
10.68	0.71025	0.71018	0.00023
10.64	0.71026	0.71014	0.00024
10.59	0.71080	0.71012	0.00024
10.55	0.70992	0.71010	0.00022
10.50	0.71060	0.71011	0.00021
10.46	0.70994	0.71012	0.00023
10.42	0.71005	0.71015	0.00023
10.37	0.71035	0.71018	0.00023
10.33	0.70951	0.71009	0.00027
10.29	0.71013	0.71011	0.00025
10.24	0.70980	0.71008	0.00026
10.20	0.71011	0.71013	0.00025
10.15	0.71058	0.71009	0.00027
10.11	0.71000	0.71003	0.00025
10.07	0.71073	0.71003	0.00025
10.02	0.71029	0.71005	0.00027
9.98	0.71034	0.71005	0.00027
9.93	0.70945	0.71006	0.00027
9.89	0.70969	0.71011	0.00024
9.85	0.70984	0.71017	0.00022
9.80	0.71032	0.71024	0.00022
9.76	0.70966	0.71019	0.00024
9.72	0.70997	0.71023	0.00021
9.67	0.71001	0.71032	0.00023
9.63	0.71089	0.71031	0.00023

9.58	0.71030	0.71026	0.00019
9.54	0.71044	0.71021	0.00021
9.50	0.70997	0.71015	0.00021
9.45	0.71029	0.71009	0.00027
9.41	0.71057	0.71010	0.00027
9.37	0.70982	0.71007	0.00025
9.32	0.71008	0.71010	0.00025
9.28	0.71080	0.71015	0.00026
9.23	0.70996	0.71005	0.00022
9.19	0.71032	0.71004	0.00022
9.15	0.70986	0.71002	0.00021
9.10	0.70987	0.71006	0.00021
9.06	0.70934	0.71012	0.00023
9.02	0.71040	0.71025	0.00017
8.97	0.71022	0.71024	0.00016
8.93	0.71019	0.71025	0.00016
8.88	0.71051	0.71026	0.00016
8.84	0.70982	0.71018	0.00018
8.80	0.70988	0.71027	0.00020
8.75	0.71010	0.71038	0.00022
8.71	0.71024	0.71038	0.00021
8.67	0.71054	0.71042	0.00022
8.62	0.71057	0.71044	0.00022
8.58	0.71036	0.71047	0.00024
8.53	0.71027	0.71053	0.00026
8.49	0.71028	0.71053	0.00026
8.45	0.70973	0.71059	0.00026
8.40	0.71073	0.71067	0.00017
8.36	0.71095	0.71066	0.00017
8.32	0.71015	0.71061	0.00016
8.27	0.71060	0.71064	0.00013
8.23	0.71073	0.71060	0.00016
8.18	0.71091	0.71059	0.00015
8.14	0.71090	0.71053	0.00014
8.10	0.71028	0.71054	0.00016
8.05	0.71086	0.71057	0.00015
8.01	0.71058	0.71049	0.00016
7.97	0.71059	0.71049	0.00016
7.92	0.71055	0.71048	0.00015
7.88	0.71039	0.71047	0.00015
7.83	0.71025	0.71049	0.00016
7.79	0.71056	0.71054	0.00015
7.75	0.71034	0.71052	0.00016
7.70	0.71102	0.71055	0.00015
7.66	0.71055	0.71050	0.00011
7.62	0.71010	0.71053	0.00013
7.57	0.71055	0.71059	0.00009
7.53	0.71047	0.71060	0.00009
7.48	0.71050	0.71059	0.00011
7.44	0.71061	0.71063	0.00012

7.40	0.71075	0.71062	0.00013
7.35	0.71032	0.71061	0.00012
7.31	0.71063	0.71065	0.00011
7.27	0.71055	0.71066	0.00011
7.22	0.71082	0.71065	0.00011
7.18	0.71071	0.71062	0.00011
7.13	0.71067	0.71057	0.00013
7.09	0.71030	0.71057	0.00013
7.05	0.71091	0.71055	0.00015
7.00	0.71055	0.71046	0.00016
6.96	0.71069	0.71045	0.00016
6.92	0.71066	0.71043	0.00016
6.87	0.71072	0.71039	0.00015
6.83	0.71051	0.71039	0.00015
6.78	0.71047	0.71043	0.00018
6.74	0.71022	0.71039	0.00019
6.70	0.71067	0.71039	0.00019
6.65	0.71014	0.71038	0.00019
6.61	0.70997	0.71034	0.00022
6.56	0.71048	0.71038	0.00021
6.52	0.71046	0.71039	0.00021
6.48	0.71031	0.71037	0.00021
6.43	0.71070	0.71034	0.00022
6.39	0.71092	0.71028	0.00021
6.35	0.71007	0.71028	0.00020
6.30	0.71023	0.71030	0.00020
6.26	0.71050	0.71030	0.00020
6.21	0.70972	0.71025	0.00020
6.17	0.71043	0.71027	0.00018
6.13	0.71055	0.71025	0.00018
6.08	0.71025	0.71016	0.00020
6.04	0.71007	0.71018	0.00021
6.00	0.71009	0.71016	0.00021
5.95	0.71087	0.71013	0.00023
5.91	0.71030	0.71005	0.00015
5.86	0.71022	0.71002	0.00014
5.82	0.70999	0.71003	0.00015
5.78	0.70996	0.71005	0.00015
5.73	0.71019	0.71004	0.00015
5.69	0.70963	0.71003	0.00015
5.65	0.71044	0.71008	0.00012
5.60	0.70989	0.71004	0.00009
5.56	0.70982	0.71004	0.00009
5.51	0.71001	0.71009	0.00009
5.47	0.71008	0.71011	0.00009
5.43	0.71030	0.71012	0.00009
5.38	0.71015	0.71014	0.00011
5.34	0.70990	0.71012	0.00011
5.30	0.71007	0.71014	0.00010
5.25	0.71010	0.71015	0.00010

5.21	0.71012	0.71015	0.00010
5.16	0.70987	0.71017	0.00011
5.12	0.71032	0.71021	0.00009
5.08	0.71016	0.71017	0.00011
5.03	0.71025	0.71018	0.00011
4.99	0.71044	0.71012	0.00014
4.95	0.70996	0.71009	0.00012
4.90	0.71015	0.71009	0.00012
4.86	0.71011	0.71005	0.00014
4.81	0.71008	0.71002	0.00015
4.77	0.71036	0.71003	0.00015
4.73	0.71032	0.71002	0.00014
4.68	0.70991	0.70998	0.00012
4.64	0.71019	0.70998	0.00012
4.60	0.70973	0.71000	0.00014
4.55	0.71011	0.71006	0.00015
4.51	0.70999	0.71009	0.00016
4.46	0.70968	0.71012	0.00016
4.42	0.70983	0.71024	0.00019
4.38	0.71022	0.71029	0.00017
4.33	0.71018	0.71030	0.00017
4.29	0.70993	0.71030	0.00017
4.25	0.70994	0.71032	0.00015
4.20	0.71038	0.71031	0.00017
4.16	0.71038	0.71032	0.00017
4.11	0.71037	0.71026	0.00021
4.07	0.71028	0.71025	0.00021
4.03	0.71087	0.71022	0.00021
3.98	0.71038	0.71015	0.00016
3.94	0.71023	0.71012	0.00015
3.90	0.71021	0.71013	0.00015
3.85	0.71013	0.71014	0.00015
3.81	0.70981	0.71014	0.00015
3.76	0.71056	0.71023	0.00017
3.72	0.70975	0.71021	0.00016
3.68	0.71023	0.71027	0.00013
3.63	0.70999	0.71026	0.00013
3.59	0.71019	0.71031	0.00012
3.55	0.71010	0.71033	0.00011
3.50	0.71025	0.71034	0.00011
3.46	0.71035	0.71034	0.00011
3.41	0.71016	0.71032	0.00012
3.37	0.71072	0.71035	0.00012
3.33	0.71030	0.71030	0.00009
3.28	0.71041	0.71032	0.00010
3.24	0.71017	0.71028	0.00012
3.19	0.71046	0.71031	0.00012
3.15	0.71041	0.71027	0.00012
3.11	0.71017	0.71029	0.00014
3.06	0.71022	0.71027	0.00015

3.02	0.71016	0.71029	0.00015
2.98	0.71050	0.71032	0.00015
2.93	0.71019	0.71035	0.00018
2.89	0.71053	0.71033	0.00019
2.84	0.70997	0.71028	0.00019
2.80	0.71050	0.71032	0.00018
2.76	0.71010	0.71034	0.00019
2.71	0.71059	0.71035	0.00018
2.67	0.70999	0.71030	0.00018
2.63	0.71042	0.71032	0.00017
2.58	0.71038	0.71031	0.00017
2.54	0.71082	0.71032	0.00017
2.49	0.70997	0.71024	0.00014
2.45	0.71010	0.71021	0.00017
2.41	0.71036	0.71024	0.00018
2.36	0.71067	0.71023	0.00018
2.32	0.71024	0.71013	0.00017
2.28	0.71010	0.71019	0.00022
2.23	0.71012	0.71020	0.00022
2.19	0.71031	0.71020	0.00022
2.14	0.71050	0.71016	0.00023
2.10	0.71005	0.71011	0.00021
2.06	0.70966	0.71011	0.00022
2.01	0.71045	0.71015	0.00019
1.97	0.71020	0.71016	0.00020
1.93	0.70973	0.71018	0.00020
1.88	0.71083	0.71018	0.00020
1.84	0.71016	0.71011	0.00014
1.79	0.71009	0.71012	0.00015
1.75	0.70993	0.71012	0.00015
1.71	0.71005	0.71016	0.00015
1.66	0.70996	0.71017	0.00015
1.62	0.71012	0.71015	0.00016
1.58	0.71057	0.71019	0.00018
1.53	0.71035	0.71012	0.00017
1.49	0.70973	0.71007	0.00017
1.44	0.71015	0.71011	0.00015
1.40	0.71024	0.71014	0.00017
1.36	0.71008	0.71013	0.00017
1.31	0.71036	0.71011	0.00018
1.27	0.71010	0.71012	0.00018
1.23	0.70977	0.71014	0.00018
1.18	0.71054	0.71019	0.00016
1.14	0.70992	0.71016	0.00015
1.09	0.70978	0.71017	0.00014
1.05	0.71019	0.71021	0.00011
1.01	0.71048	0.71017	0.00014
0.96	0.71011	0.71015	0.00013
0.92	0.70989	0.71015	0.00013
0.88	0.71039	0.71016	0.00012

0.83	0.71034	0.71016	0.00011
0.79	0.71024	0.71016	0.00011
0.74	0.71023	0.71014	0.00011
0.70	0.71008	0.71018	0.00015
0.66	0.71018	0.71024	0.00018
0.61	0.70974	0.71024	0.00018
0.57	0.71026	0.71027	0.00015
0.53	0.71012	0.71027	0.00015
0.48	0.71005	0.71030	0.00015
0.44	0.71032	0.71031	0.00014
0.39	0.71036	0.71031	0.00015
0.35	0.71006	0.71031	0.00016
0.31	0.71062	0.71034	0.00016
0.26	0.71074	0.71030	0.00015
0.22	0.71011	0.71021	0.00008
0.18	0.71010	0.71024	0.00008
0.13	0.71024	0.71028	0.00007
0.09	0.71041	0.71030	0.00010
0.04	0.71019		

ARB 111.2.1 (M2)

Distance from cervix (mm)	$^{87}Sr/^{86}Sr$	10 point mov. average	2 SE on mov. average
25.87	0.70997	0.70992	0.00018
25.82	0.71017	0.70992	0.00018
25.78	0.70968	0.70996	0.00022
25.74	0.70958	0.70996	0.00022
25.69	0.70996	0.70998	0.00021
25.65	0.70976	0.70997	0.00021
25.61	0.71021	0.70997	0.00021
25.56	0.70945	0.70995	0.00021
25.52	0.71025	0.70996	0.00019
25.48	0.71017	0.70998	0.00021
25.43	0.70996	0.70995	0.00020
25.39	0.71059	0.70993	0.00021
25.35	0.70971	0.70985	0.00014
25.30	0.70970	0.70986	0.00014
25.26	0.70988	0.70990	0.00014
25.22	0.70978	0.70991	0.00014
25.17	0.71002	0.70993	0.00014
25.13	0.70959	0.70990	0.00014
25.09	0.71042	0.70993	0.00013
25.04	0.70981	0.70990	0.00008
25.00	0.70976	0.70988	0.00009
24.96	0.70985	0.70982	0.00016
24.91	0.70982	0.70986	0.00019
24.87	0.71003	0.70985	0.00019
24.83	0.71006	0.70986	0.00019
24.78	0.70996	0.70983	0.00019
24.74	0.70973	0.70985	0.00020
24.70	0.70991	0.70986	0.00020
24.65	0.71003	0.70981	0.00021
24.61	0.70962	0.70979	0.00021
24.57	0.70920	0.70982	0.00020
24.52	0.71027	0.70987	0.00016
24.48	0.70971	0.70985	0.00014
24.44	0.71009	0.70990	0.00015
24.39	0.70977	0.70987	0.00015
24.35	0.71016	0.70992	0.00016
24.31	0.70982	0.70988	0.00016
24.26	0.70946	0.70985	0.00017
24.22	0.70979	0.70985	0.00017
24.18	0.70996	0.70989	0.00018
24.13	0.70966	0.70986	0.00018
24.09	0.71006	0.70991	0.00018
24.05	0.71024	0.70992	0.00018
24.00	0.70980	0.70981	0.00022
23.96	0.71023	0.70986	0.00024
23.92	0.70975	0.70982	0.00022
23.87	0.70956	0.70980	0.00022

23.83	0.70946	0.70984	0.00022
23.79	0.71014	0.70982	0.00024
23.74	0.70974	0.70980	0.00023
23.70	0.71012	0.70976	0.00025
23.66	0.71013	0.70970	0.00024
23.61	0.70921	0.70966	0.00022
23.57	0.71029	0.70966	0.00023
23.53	0.70977	0.70959	0.00018
23.48	0.70961	0.70956	0.00018
23.44	0.70995	0.70958	0.00019
23.40	0.70922	0.70954	0.00017
23.35	0.70999	0.70962	0.00018
23.31	0.70935	0.70958	0.00016
23.27	0.70951	0.70963	0.00015
23.22	0.70975	0.70968	0.00017
23.18	0.70914	0.70969	0.00017
23.14	0.70957	0.70974	0.00013
23.09	0.70946	0.70972	0.00013
23.05	0.70987	0.70974	0.00012
23.01	0.70956	0.70968	0.00015
22.96	0.71000	0.70965	0.00017
22.92	0.70961	0.70958	0.00017
22.88	0.70979	0.70955	0.00018
22.83	0.71003	0.70949	0.00018
22.79	0.70985	0.70945	0.00014
22.75	0.70962	0.70944	0.00013
22.70	0.70946	0.70948	0.00017
22.66	0.70966	0.70949	0.00018
22.62	0.70926	0.70953	0.00021
22.57	0.70927	0.70960	0.00022
22.53	0.70927	0.70969	0.00022
22.49	0.70928	0.70972	0.00021
22.44	0.70922	0.70980	0.00019
22.40	0.70956	0.70987	0.00014
22.36	0.70977	0.70989	0.00012
22.31	0.71005	0.70996	0.00016
22.27	0.70958	0.70992	0.00017
22.23	0.71006	0.70998	0.00016
22.18	0.70999	0.70999	0.00017
22.14	0.71012	0.70999	0.00017
22.10	0.70961	0.71000	0.00018
22.05	0.70999	0.70999	0.00018
22.01	0.70997	0.70993	0.00022
21.97	0.70977	0.70987	0.00025
21.92	0.71044	0.70989	0.00025
21.88	0.70962	0.70989	0.00025
21.84	0.71024	0.70983	0.00030
21.79	0.71018	0.70982	0.00030
21.75	0.70992	0.70989	0.00037
21.71	0.71025	0.70986	0.00037

21.66	0.70954	0.70986	0.00037
21.62	0.70939	0.70985	0.00038
21.58	0.70937	0.70988	0.00037
21.53	0.70991	0.70995	0.00035
21.49	0.71049	0.71000	0.00036
21.45	0.70902	0.70998	0.00035
21.40	0.71011	0.71009	0.00028
21.36	0.71095	0.71009	0.00028
21.32	0.70961	0.71012	0.00032
21.27	0.71022	0.71020	0.00030
21.23	0.70942	0.71016	0.00031
21.19	0.70967	0.71021	0.00027
21.14	0.71007	0.71027	0.00024
21.10	0.71038	0.71023	0.00026
21.06	0.71033	0.71024	0.00027
21.01	0.71009	0.71021	0.00027
20.97	0.71016	0.71030	0.00031
20.93	0.71121	0.71033	0.00031
20.88	0.71046	0.71024	0.00024
20.84	0.70981	0.71017	0.00026
20.80	0.70995	0.71017	0.00026
20.75	0.71019	0.71019	0.00025
20.71	0.70974	0.71017	0.00025
20.67	0.71045	0.71028	0.00026
20.62	0.71000	0.71032	0.00029
20.58	0.71103	0.71042	0.00031
20.54	0.71041	0.71029	0.00031
20.49	0.71039	0.71026	0.00031
20.45	0.70976	0.71030	0.00033
20.41	0.70982	0.71038	0.00031
20.36	0.71009	0.71035	0.00033
20.32	0.71004	0.71037	0.00033
20.28	0.71078	0.71038	0.00033
20.23	0.71091	0.71031	0.00032
20.19	0.71098	0.71024	0.00029
20.15	0.70968	0.71025	0.00030
20.10	0.71012	0.71033	0.00028
20.06	0.71087	0.71034	0.00027
20.02	0.71053	0.71027	0.00025
19.97	0.70954	0.71018	0.00027
19.93	0.71025	0.71024	0.00023
19.89	0.71010	0.71026	0.00023
19.84	0.71012	0.71022	0.00026
19.80	0.71017	0.71018	0.00028
19.76	0.71110	0.71025	0.00032
19.71	0.71047	0.71020	0.00026
19.67	0.71027	0.71013	0.00027
19.63	0.71017	0.71015	0.00027
19.58	0.70965	0.71013	0.00028
19.54	0.71013	0.71018	0.00025

19.50	0.71039	0.71016	0.00026
19.45	0.70970	0.71008	0.00028
19.41	0.70971	0.71017	0.00028
19.37	0.71095	0.71017	0.00028
19.32	0.71054	0.71014	0.00025
19.28	0.70980	0.71006	0.00024
19.24	0.71045	0.71006	0.00024
19.19	0.71004	0.71005	0.00024
19.15	0.71012	0.71000	0.00026
19.11	0.70993	0.71003	0.00027
19.06	0.70955	0.71003	0.00027
19.02	0.71061	0.71011	0.00025
18.98	0.70977	0.71008	0.00023
18.93	0.71064	0.71005	0.00025
18.89	0.70972	0.70995	0.00022
18.85	0.70980	0.70999	0.00022
18.80	0.71035	0.71002	0.00021
18.76	0.70955	0.70994	0.00021
18.72	0.71034	0.71002	0.00021
18.67	0.71000	0.71000	0.00020
18.63	0.71029	0.70996	0.00021
18.59	0.71030	0.70989	0.00021
18.54	0.70949	0.70989	0.00021
18.50	0.70965	0.70991	0.00020
18.46	0.71012	0.70998	0.00021
18.41	0.71007	0.70999	0.00021
18.37	0.70958	0.70993	0.00023
18.33	0.71031	0.71001	0.00023
18.28	0.71019	0.71000	0.00023
18.24	0.70964	0.71000	0.00023
18.20	0.70954	0.71000	0.00023
18.15	0.71027	0.71008	0.00022
18.11	0.70970	0.71011	0.00024
18.07	0.71041	0.71014	0.00022
18.02	0.71013	0.71009	0.00022
17.98	0.70949	0.71013	0.00024
17.94	0.71038	0.71021	0.00020
17.89	0.71025	0.71020	0.00020
17.85	0.71018	0.71013	0.00024
17.81	0.70961	0.71010	0.00024
17.76	0.71037	0.71008	0.00026
17.72	0.71059	0.71012	0.00028
17.68	0.71003	0.71003	0.00027
17.63	0.70984	0.71005	0.00027
17.59	0.71060	0.71008	0.00027
17.55	0.71022	0.71007	0.00026
17.50	0.71035	0.71005	0.00026
17.46	0.70953	0.71007	0.00027
17.42	0.70984	0.71015	0.00025
17.37	0.70949	0.71015	0.00025

17.33	0.71071	0.71023	0.00020
17.29	0.70973	0.71018	0.00017
17.24	0.71015	0.71027	0.00016
17.20	0.71014	0.71029	0.00016
17.16	0.71049	0.71028	0.00016
17.11	0.71006	0.71019	0.00020
17.07	0.71054	0.71021	0.00020
17.03	0.71039	0.71024	0.00023
16.98	0.70980	0.71025	0.00023
16.94	0.71026	0.71027	0.00022
16.90	0.71021	0.71027	0.00022
16.85	0.71066	0.71029	0.00022
16.81	0.71029	0.71020	0.00023
16.77	0.71007	0.71019	0.00023
16.72	0.70963	0.71019	0.00023
16.68	0.71026	0.71025	0.00020
16.64	0.71083	0.71017	0.00024
16.59	0.71053	0.71004	0.00023
16.55	0.70996	0.70997	0.00020
16.51	0.71024	0.71000	0.00021
16.46	0.71045	0.70998	0.00020
16.42	0.70973	0.70997	0.00019
16.38	0.71022	0.70998	0.00018
16.33	0.71005	0.70995	0.00018
16.29	0.71018	0.71001	0.00021
16.25	0.70951	0.70994	0.00023
16.20	0.70953	0.71001	0.00022
16.16	0.70985	0.71007	0.00019
16.12	0.71026	0.71003	0.00022
16.07	0.71005	0.71002	0.00022
16.03	0.71030	0.70998	0.00023
15.99	0.70982	0.70989	0.00024
15.94	0.71000	0.70994	0.00026
15.90	0.71057	0.70993	0.00026
15.86	0.70951	0.70996	0.00030
15.81	0.71023	0.71002	0.00029
15.77	0.71013	0.70996	0.00029
15.73	0.70946	0.70993	0.00029
15.68	0.71015	0.70999	0.00027
15.64	0.70960	0.71000	0.00028
15.60	0.70941	0.71003	0.00027
15.55	0.71029	0.71004	0.00025
15.51	0.70989	0.70996	0.00027
15.47	0.71094	0.70998	0.00027
15.42	0.71010	0.70991	0.00018
15.38	0.70965	0.70993	0.00019
15.34	0.70979	0.70997	0.00018
15.29	0.71005	0.70999	0.00018
15.25	0.71029	0.71004	0.00021
15.21	0.70989	0.71000	0.00020

15.16	0.70955	0.71001	0.00020
15.12	0.70950	0.71005	0.00017
15.08	0.71001	0.71006	0.00015
15.03	0.71026	0.71007	0.00015
14.99	0.71032	0.71008	0.00015
14.95	0.71000	0.71001	0.00017
14.90	0.71008	0.71004	0.00018
14.86	0.71049	0.71008	0.00020
14.82	0.70990	0.71007	0.00019
14.77	0.70994	0.71013	0.00021
14.73	0.70996	0.71012	0.00021
14.69	0.70967	0.71013	0.00021
14.64	0.71008	0.71019	0.00018
14.60	0.71034	0.71020	0.00018
14.56	0.70962	0.71015	0.00019
14.51	0.71036	0.71021	0.00015
14.47	0.71040	0.71018	0.00015
14.43	0.71046	0.71023	0.00021
14.38	0.71049	0.71022	0.00020
14.34	0.70984	0.71023	0.00021
14.30	0.71006	0.71030	0.00020
14.25	0.71027	0.71030	0.00020
14.21	0.71014	0.71023	0.00025
14.17	0.70986	0.71022	0.00026
14.12	0.71019	0.71026	0.00024
14.08	0.71007	0.71024	0.00025
14.04	0.71092	0.71025	0.00025
13.99	0.71033	0.71022	0.00021
13.95	0.71067	0.71020	0.00022
13.91	0.71047	0.71015	0.00019
13.86	0.71013	0.71012	0.00017
13.82	0.70950	0.71012	0.00017
13.78	0.71007	0.71019	0.00011
13.73	0.71025	0.71019	0.00011
13.69	0.70999	0.71018	0.00011
13.65	0.71023	0.71020	0.00010
13.60	0.71060	0.71024	0.00014
13.56	0.71005	0.71027	0.00018
13.52	0.71020	0.71022	0.00022
13.47	0.71014	0.71025	0.00023
13.43	0.71016	0.71026	0.00022
13.39	0.71024	0.71026	0.00022
13.34	0.70999	0.71020	0.00025
13.30	0.71022	0.71020	0.00025
13.26	0.71016	0.71023	0.00026
13.21	0.71067	0.71017	0.00029
13.17	0.71088	0.71014	0.00027
13.13	0.70959	0.71004	0.00022
13.08	0.71045	0.71008	0.00020
13.04	0.71024	0.71003	0.00018

13.00	0.71014	0.70997	0.00019
12.96	0.70966	0.70996	0.00019
12.91	0.71005	0.71001	0.00018
12.87	0.71044	0.71002	0.00018
12.83	0.70957	0.70997	0.00016
12.78	0.71039	0.71001	0.00013
12.74	0.70990	0.70996	0.00010
12.70	0.70996	0.71001	0.00013
12.65	0.70997	0.71001	0.00013
12.61	0.70963	0.70995	0.00018
12.57	0.71001	0.70996	0.00017
12.52	0.71024	0.70995	0.00017
12.48	0.71011	0.70991	0.00016
12.44	0.70994	0.70986	0.00016
12.39	0.70993	0.70986	0.00016
12.35	0.70989	0.70987	0.00016
12.31	0.71042	0.70986	0.00016
12.26	0.70993	0.70976	0.00013
12.22	0.70944	0.70975	0.00013
12.18	0.70973	0.70981	0.00012
12.13	0.70988	0.70990	0.00020
12.09	0.70980	0.70989	0.00020
12.05	0.70965	0.70994	0.00021
12.00	0.70993	0.71002	0.00022
11.96	0.71002	0.71003	0.00022
11.92	0.70981	0.71006	0.00023
11.87	0.70940	0.71006	0.00022
11.83	0.70982	0.71020	0.00021
11.79	0.71009	0.71024	0.00020
11.74	0.71059	0.71025	0.00019
11.70	0.70981	0.71018	0.00019
11.66	0.71028	0.71018	0.00020
11.61	0.71043	0.71012	0.00021
11.57	0.71004	0.71010	0.00021
11.53	0.71029	0.71008	0.00022
11.48	0.70983	0.71005	0.00021
11.44	0.71078	0.71006	0.00021
11.40	0.71023	0.71000	0.00014
11.35	0.71024	0.71001	0.00015
11.31	0.70991	0.70999	0.00014
11.27	0.70972	0.71001	0.00014
11.22	0.70973	0.71007	0.00014
11.18	0.71027	0.71017	0.00017
11.14	0.70979	0.71018	0.00017
11.09	0.70998	0.71020	0.00016
11.05	0.70997	0.71021	0.00015
11.01	0.71021	0.71020	0.00016
10.96	0.71032	0.71017	0.00017
10.92	0.70997	0.71015	0.00017
10.88	0.71011	0.71005	0.00029

10.83	0.71033	0.71006	0.00030
10.79	0.71073	0.70999	0.00030
10.75	0.71037	0.70990	0.00025
10.70	0.70999	0.70986	0.00023
10.66	0.71013	0.70988	0.00024
10.62	0.70980	0.70983	0.00024
10.57	0.70990	0.70983	0.00024
10.53	0.71019	0.70978	0.00025
10.49	0.70896	0.70977	0.00024
10.44	0.71019	0.70990	0.00019
10.40	0.70967	0.70982	0.00021
10.36	0.70984	0.70987	0.00022
10.31	0.70998	0.70993	0.00025
10.27	0.71018	0.70993	0.00025
10.23	0.70958	0.70993	0.00025
10.18	0.70979	0.70997	0.00023
10.14	0.70942	0.70996	0.00024
10.10	0.71005	0.70999	0.00021
10.05	0.71034	0.70998	0.00021
10.01	0.70932	0.70990	0.00021
9.97	0.71018	0.70999	0.00017
9.92	0.71047	0.70999	0.00018
9.88	0.71001	0.70996	0.00014
9.84	0.71015	0.70992	0.00016
9.79	0.70998	0.70999	0.00024
9.75	0.70965	0.71000	0.00024
9.71	0.70977	0.71007	0.00023
9.66	0.70994	0.71014	0.00024
9.62	0.70956	0.71017	0.00024
9.58	0.71018	0.71016	0.00024
9.53	0.71020	0.71009	0.00028
9.49	0.71014	0.71006	0.00028
9.45	0.70962	0.71007	0.00028
9.40	0.71082	0.71012	0.00026
9.36	0.71016	0.71006	0.00021
9.32	0.71030	0.71016	0.00031
9.27	0.71052	0.71020	0.00033
9.23	0.71018	0.71013	0.00033
9.19	0.70953	0.71021	0.00037
9.14	0.70947	0.71028	0.00034
9.10	0.70988	0.71030	0.00032
9.06	0.71024	0.71036	0.00030
9.01	0.71014	0.71036	0.00030
8.97	0.71017	0.71041	0.00030
8.93	0.71118	0.71039	0.00031
8.88	0.71072	0.71028	0.00026
8.84	0.70979	0.71021	0.00025
8.80	0.71095	0.71017	0.00029
8.75	0.71029	0.71006	0.00024
8.71	0.70968	0.71007	0.00025

8.67	0.71044	0.71018	0.00027
8.62	0.71022	0.71015	0.00026
8.58	0.71066	0.71010	0.00028
8.54	0.71000	0.71008	0.00026
8.49	0.71000	0.71008	0.00026
8.45	0.71012	0.71007	0.00027
8.41	0.70933	0.71007	0.00027
8.36	0.70984	0.71018	0.00021
8.32	0.71038	0.71018	0.00021
8.28	0.71077	0.71013	0.00021
8.23	0.71021	0.71008	0.00016
8.19	0.70966	0.71007	0.00016
8.15	0.71047	0.71006	0.00018
8.10	0.71007	0.71003	0.00016
8.06	0.70986	0.71007	0.00018
8.02	0.71012	0.71005	0.00019
7.97	0.71038	0.71004	0.00019
7.93	0.70993	0.71003	0.00019
7.89	0.70986	0.71004	0.00019
7.84	0.71027	0.71009	0.00019
7.80	0.71010	0.71012	0.00021
7.76	0.70951	0.71011	0.00021
7.71	0.71020	0.71019	0.00017
7.67	0.71049	0.71022	0.00018
7.63	0.70969	0.71015	0.00019
7.58	0.70995	0.71024	0.00017
7.54	0.71025	0.71026	0.00016
7.50	0.71007	0.71030	0.00018
7.45	0.71036	0.71029	0.00019
7.41	0.71053	0.71024	0.00020
7.37	0.71000	0.71018	0.00020
7.32	0.71037	0.71017	0.00021
7.28	0.71052	0.71016	0.00020
7.24	0.70980	0.71010	0.00019
7.19	0.71057	0.71010	0.00019
7.15	0.71010	0.71009	0.00018
7.11	0.71064	0.71006	0.00019
7.06	0.70996	0.70997	0.00014
7.02	0.70995	0.70990	0.00021
6.98	0.70985	0.70989	0.00021
6.93	0.70990	0.70989	0.00021
6.89	0.71029	0.70993	0.00022
6.85	0.70992	0.70991	0.00021
6.80	0.70985	0.70991	0.00021
6.76	0.71045	0.70997	0.00024
6.72	0.70976	0.70994	0.00021
6.67	0.70978	0.70996	0.00021
6.63	0.70921	0.70998	0.00021
6.59	0.70987	0.71015	0.00020
6.54	0.70987	0.71017	0.00020

6.50	0.71027	0.71022	0.00019
6.46	0.71017	0.71015	0.00023
6.41	0.70991	0.71011	0.00024
6.37	0.71043	0.71007	0.00026
6.33	0.71011	0.71002	0.00025
6.28	0.71002	0.71000	0.00025
6.24	0.70995	0.70995	0.00027
6.20	0.71091	0.70989	0.00029
6.15	0.71003	0.70986	0.00024
6.11	0.71038	0.70982	0.00024
6.07	0.70959	0.70980	0.00023
6.02	0.70974	0.70987	0.00024
5.98	0.70953	0.70990	0.00024
5.94	0.70990	0.70995	0.00023
5.89	0.70993	0.70995	0.00023
5.85	0.70955	0.70994	0.00023
5.81	0.70938	0.71005	0.00024
5.76	0.71055	0.71015	0.00020
5.72	0.70962	0.71016	0.00021
5.68	0.71022	0.71018	0.00018
5.63	0.71025	0.71017	0.00018
5.59	0.71008	0.71020	0.00020
5.55	0.71004	0.71018	0.00021
5.50	0.70987	0.71027	0.00026
5.46	0.70986	0.71027	0.00026
5.42	0.71061	0.71029	0.00025
5.37	0.71037	0.71019	0.00027
5.33	0.71064	0.71018	0.00027
5.29	0.70989	0.71009	0.00026
5.24	0.71010	0.71008	0.00026
5.20	0.71055	0.71011	0.00027
5.16	0.70984	0.71014	0.00029
5.11	0.71096	0.71011	0.00032
5.07	0.70986	0.71000	0.00025
5.03	0.71003	0.71002	0.00025
4.98	0.70964	0.71001	0.00025
4.94	0.71030	0.71007	0.00024
4.90	0.70972	0.71013	0.00029
4.85	0.70985	0.71014	0.00028
4.81	0.71036	0.71020	0.00028
4.77	0.71085	0.71016	0.00028
4.72	0.70951	0.71011	0.00024
4.68	0.70986	0.71015	0.00020
4.64	0.71011	0.71016	0.00020
4.59	0.70994	0.71011	0.00022
4.55	0.71021	0.71014	0.00022
4.51	0.71085	0.71011	0.00023
4.46	0.70985	0.70998	0.00019
4.42	0.71045	0.70996	0.00019
4.38	0.70999	0.70991	0.00016

4.33	0.71033	0.70990	0.00016
4.29	0.70990	0.70985	0.00013
4.25	0.70996	0.70987	0.00014
4.20	0.70962	0.70994	0.00020
4.16	0.71024	0.70996	0.00018
4.12	0.70991	0.70995	0.00018
4.07	0.70951	0.70996	0.00018
4.03	0.70972	0.71000	0.00015
3.99	0.70995	0.71003	0.00014
3.94	0.70988	0.71005	0.00014
3.90	0.70986	0.71004	0.00014
3.86	0.71010	0.71007	0.00014
3.81	0.71058	0.71008	0.00014
3.77	0.70987	0.71007	0.00013
3.73	0.71013	0.71010	0.00012
3.68	0.71005	0.71009	0.00012
3.64	0.70985	0.71006	0.00014
3.60	0.70999	0.71010	0.00014
3.55	0.71016	0.71006	0.00018
3.51	0.70981	0.70999	0.00021
3.47	0.71012	0.70996	0.00023
3.42	0.71021	0.70993	0.00023
3.38	0.71048	0.70990	0.00022
3.34	0.71025	0.70991	0.00023
3.29	0.71003	0.70983	0.00023
3.25	0.70973	0.70984	0.00024
3.21	0.71026	0.70986	0.00024
3.16	0.70951	0.70979	0.00022
3.12	0.70949	0.70981	0.00022
3.08	0.70957	0.70987	0.00021
3.03	0.70974	0.70994	0.00021
2.99	0.70998	0.70999	0.00021
2.95	0.71055	0.70997	0.00022
2.90	0.70945	0.70991	0.00017
2.86	0.71016	0.70998	0.00014
2.82	0.70989	0.71000	0.00016
2.77	0.70961	0.71001	0.00016
2.73	0.70969	0.71006	0.00013
2.69	0.71010	0.71010	0.00011
2.64	0.71027	0.71005	0.00014
2.60	0.71023	0.71004	0.00013
2.56	0.70979	0.71001	0.00013
2.51	0.70993	0.71006	0.00013
2.47	0.71009	0.71006	0.00012
2.43	0.71038	0.71006	0.00012
2.38	0.71000	0.71002	0.00010
2.34	0.71011	0.71002	0.00010
2.30	0.71008	0.71000	0.00011
2.25	0.70965	0.71002	0.00012
2.21	0.71012	0.71002	0.00012

2.17	0.70993	0.71000	0.00012
2.12	0.71027	0.71002	0.00012
2.08	0.70999	0.70999	0.00011
2.04	0.71007	0.70998	0.00011
1.99	0.70997	0.70993	0.00014
1.95	0.71003	0.70990	0.00015
1.91	0.70987	0.70986	0.00015
1.86	0.71032	0.70986	0.00015
1.82	0.70967	0.70981	0.00011
1.78	0.70992	0.70989	0.00016
1.73	0.71011	0.70989	0.00016
1.69	0.70995	0.70990	0.00016
1.65	0.70993	0.70993	0.00018
1.60	0.70950	0.70987	0.00022
1.56	0.70964	0.70992	0.00020
1.52	0.70973	0.70994	0.00019
1.47	0.70982	0.71000	0.00020
1.43	0.70985	0.71003	0.00020
1.39	0.71041	0.71009	0.00021
1.34	0.70997	0.70998	0.00025
1.30	0.71017	0.70997	0.00025
1.26	0.71030	0.70994	0.00025
1.21	0.70933	0.70989	0.00023
1.17	0.70996	0.70996	0.00020
1.13	0.70990	0.70995	0.00020
1.08	0.71032	0.70992	0.00022
1.04	0.71010	0.70984	0.00021
1.00	0.71043	0.70986	0.00022
0.95	0.70926	0.70981	0.00019
0.91	0.70992	0.70987	0.00015
0.87	0.70984	0.70989	0.00015
0.82	0.70984	0.70986	0.00016
0.78	0.71001	0.70989	0.00018
0.74	0.70991	0.70983	0.00020
0.69	0.70954	0.70979	0.00021
0.65	0.70950	0.70979	0.00021
0.61	0.71030	0.70981	0.00020
0.56	0.71001	0.70978	0.00017
0.52	0.70983	0.70977	0.00017
0.48	0.71008	0.70978	0.00017
0.43	0.70958	0.70973	0.00016
0.39	0.71019	0.70975	0.00017
0.35	0.70937	0.70970	0.00014
0.30	0.70951	0.70974	0.00013
0.26	0.70957	0.70978	0.00012
0.22	0.70969	0.70982	0.00011
0.17	0.70998	0.70985	0.00012
0.13	0.70988	0.70981	0.00013
0.09	0.70997	0.70978	0.00018
0.04	0.70958		

ARB 112.2.1 (M2)

Distance from cervix (mm)	$^{87}Sr/^{86}Sr$	10 point mov. average	2 SE on mov. average
40.02	0.70912	0.70904	0.00010
39.98	0.70902	0.70902	0.00010
39.93	0.70920	0.70901	0.00010
39.89	0.70896	0.70897	0.00010
39.85	0.70887	0.70899	0.00011
39.80	0.70935	0.70899	0.00011
39.76	0.70882	0.70894	0.00008
39.72	0.70897	0.70895	0.00008
39.67	0.70905	0.70895	0.00008
39.63	0.70902	0.70890	0.00010
39.59	0.70895	0.70890	0.00010
39.54	0.70886	0.70893	0.00013
39.50	0.70881	0.70899	0.00016
39.46	0.70920	0.70901	0.00016
39.41	0.70883	0.70900	0.00015
39.37	0.70889	0.70899	0.00016
39.33	0.70887	0.70903	0.00017
39.28	0.70900	0.70905	0.00016
39.24	0.70859	0.70906	0.00016
39.20	0.70893	0.70910	0.00013
39.15	0.70935	0.70911	0.00012
39.11	0.70943	0.70910	0.00012
39.07	0.70899	0.70908	0.00010
39.02	0.70907	0.70908	0.00010
38.98	0.70877	0.70909	0.00010
38.94	0.70926	0.70914	0.00007
38.89	0.70905	0.70912	0.00007
38.85	0.70916	0.70913	0.00007
38.81	0.70903	0.70912	0.00007
38.76	0.70896	0.70914	0.00007
38.72	0.70930	0.70914	0.00007
38.68	0.70919	0.70913	0.00006
38.63	0.70904	0.70908	0.00009
38.59	0.70912	0.70910	0.00009
38.55	0.70925	0.70911	0.00010
38.50	0.70911	0.70909	0.00009
38.46	0.70910	0.70907	0.00010
38.42	0.70905	0.70909	0.00011
38.37	0.70929	0.70913	0.00012
38.33	0.70899	0.70910	0.00012
38.29	0.70911	0.70910	0.00012
38.24	0.70876	0.70911	0.00013
38.20	0.70920	0.70915	0.00010
38.16	0.70921	0.70916	0.00010
38.11	0.70909	0.70916	0.00010
38.07	0.70893	0.70916	0.00010
38.03	0.70932	0.70918	0.00009

ARB 112.3.1 (M3)

Distance from cervix (mm)	$^{87}Sr/^{86}Sr$	10 point mov. average	2 SE on mov. average
46.67	0.70822	0.70878	0.00019
46.62	0.70881	0.70887	0.00016
46.56	0.70882	0.70890	0.00017
46.51	0.70857	0.70894	0.00017
46.46	0.70878	0.70899	0.00016
46.40	0.70865	0.70900	0.00015
46.35	0.70909	0.70900	0.00015
46.29	0.70877	0.70896	0.00016
46.24	0.70871	0.70894	0.00018
46.19	0.70940	0.70895	0.00018
46.13	0.70913	0.70891	0.00015
46.08	0.70910	0.70889	0.00014
46.03	0.70917	0.70884	0.00014
45.97	0.70912	0.70884	0.00013
45.92	0.70884	0.70882	0.00011
45.86	0.70868	0.70884	0.00013
45.81	0.70871	0.70889	0.00014
45.76	0.70849	0.70894	0.00014
45.70	0.70887	0.70900	0.00011
45.65	0.70897	0.70900	0.00011
45.60	0.70893	0.70899	0.00011
45.54	0.70864	0.70897	0.00012
45.49	0.70909	0.70898	0.00011
45.43	0.70893	0.70897	0.00011
45.38	0.70910	0.70895	0.00012
45.33	0.70915	0.70895	0.00012
45.27	0.70922	0.70890	0.00012
45.22	0.70912	0.70888	0.00010
45.17	0.70884	0.70884	0.00009
45.11	0.70888	0.70885	0.00009
45.06	0.70874	0.70889	0.00013
45.01	0.70875	0.70893	0.00013
44.95	0.70898	0.70899	0.00015
44.90	0.70874	0.70901	0.00015
44.84	0.70911	0.70905	0.00014
44.79	0.70867	0.70902	0.00016
44.74	0.70896	0.70901	0.00017
44.68	0.70875	0.70901	0.00017
44.63	0.70890	0.70903	0.00016
44.58	0.70929	0.70905	0.00015
44.52	0.70912	0.70897	0.00017
44.47	0.70937	0.70896	0.00017
44.41	0.70916	0.70890	0.00015
44.36	0.70919	0.70887	0.00014
44.31	0.70874	0.70888	0.00015
44.25	0.70857	0.70893	0.00016
44.20	0.70900	0.70899	0.00015

37.98	0.70940	0.70918	0.00009
37.94	0.70898	0.70919	0.00011
37.90	0.70897	0.70919	0.00011
37.85	0.70928	0.70919	0.00011
37.81	0.70915	0.70913	0.00015
37.77	0.70923	0.70911	0.00015
37.72	0.70926	0.70910	0.00015
37.68	0.70907	0.70906	0.00015
37.64	0.70908	0.70907	0.00015
37.59	0.70932	0.70909	0.00016
37.55	0.70953	0.70908	0.00015
37.51	0.70896	0.70899	0.00014
37.46	0.70899	0.70897	0.00014
37.42	0.70867	0.70896	0.00015
37.38	0.70895	0.70900	0.00014
37.33	0.70915	0.70906	0.00017
37.29	0.70889	0.70907	0.00018
37.25	0.70916	0.70908	0.00017
37.20	0.70928	0.70906	0.00017
37.16	0.70923	0.70903	0.00017
37.12	0.70866	0.70901	0.00016
37.07	0.70875	0.70907	0.00015
37.03	0.70882	0.70911	0.00014
36.99	0.70915	0.70919	0.00014
36.94	0.70953	0.70918	0.00015
36.90	0.70929	0.70913	0.00013
36.86	0.70898	0.70915	0.00014
36.81	0.70894	0.70914	0.00014
36.77	0.70895	0.70917	0.00014
36.73	0.70901	0.70918	0.00013
36.68	0.70930	0.70919	0.00013
36.64	0.70917	0.70914	0.00014
36.60	0.70956	0.70915	0.00014
36.56	0.70908	0.70909	0.00011
36.51	0.70903	0.70908	0.00011
36.47	0.70946	0.70910	0.00011
36.43	0.70894	0.70908	0.00009
36.38	0.70921	0.70907	0.00010
36.34	0.70898	0.70904	0.00010
36.30	0.70912	0.70906	0.00010
36.25	0.70886	0.70907	0.00011
36.21	0.70923	0.70907	0.00010
36.17	0.70899	0.70903	0.00011
36.12	0.70901	0.70904	0.00011
36.08	0.70921	0.70903	0.00011
36.04	0.70924	0.70900	0.00010
35.99	0.70884	0.70898	0.00009
35.95	0.70889	0.70902	0.00009
35.91	0.70921	0.70904	0.00009
35.86	0.70921	0.70902	0.00008
44.15	0.70899	0.70897	0.00015
44.09	0.70902	0.70898	0.00015
44.04	0.70853	0.70900	0.00016
43.98	0.70902	0.70903	0.00013
43.93	0.70876	0.70902	0.00014
43.88	0.70886	0.70906	0.00013
43.82	0.70929	0.70908	0.00012
43.77	0.70929	0.70903	0.00012
43.72	0.70916	0.70896	0.00014
43.66	0.70879	0.70897	0.00015
43.61	0.70906	0.70897	0.00015
43.56	0.70926	0.70899	0.00017
43.50	0.70883	0.70894	0.00016
43.45	0.70890	0.70897	0.00016
43.39	0.70917	0.70899	0.00017
43.34	0.70909	0.70897	0.00016
43.29	0.70879	0.70898	0.00017
43.23	0.70856	0.70897	0.00017
43.18	0.70928	0.70900	0.00015
43.13	0.70871	0.70895	0.00014
43.07	0.70930	0.70895	0.00014
43.02	0.70876	0.70892	0.00012
42.96	0.70913	0.70898	0.00014
42.91	0.70915	0.70896	0.00013
42.86	0.70894	0.70894	0.00013
42.80	0.70917	0.70889	0.00016
42.75	0.70873	0.70889	0.00015
42.70	0.70884	0.70894	0.00016
42.64	0.70880	0.70897	0.00017
42.59	0.70867	0.70899	0.00016
42.53	0.70902	0.70904	0.00015
42.48	0.70933	0.70903	0.00015
42.43	0.70897	0.70898	0.00014
42.37	0.70894	0.70894	0.00015
42.32	0.70846	0.70892	0.00016
42.27	0.70911	0.70900	0.00013
42.21	0.70922	0.70899	0.00013
42.16	0.70914	0.70897	0.00012
42.11	0.70905	0.70894	0.00012
42.05	0.70916	0.70893	0.00012
42.00	0.70889	0.70893	0.00012
41.94	0.70882	0.70895	0.00012
41.89	0.70866	0.70895	0.00012
41.84	0.70873	0.70896	0.00010
41.78	0.70918	0.70896	0.00011
41.73	0.70906	0.70892	0.00010
41.68	0.70906	0.70890	0.00010
41.62	0.70875	0.70890	0.00010
41.57	0.70901	0.70893	0.00010
41.51	0.70915	0.70889	0.00012

35.82	0.70888	0.70900	0.00007
35.78	0.70884	0.70901	0.00006
35.73	0.70905	0.70903	0.00005
35.69	0.70893	0.70898	0.00010
35.65	0.70894	0.70898	0.00010
35.60	0.70903	0.70900	0.00010
35.56	0.70922	0.70900	0.00011
35.52	0.70907	0.70897	0.00009
35.47	0.70902	0.70898	0.00011
35.43	0.70898	0.70898	0.00011
35.39	0.70901	0.70897	0.00011
35.34	0.70901	0.70899	0.00011
35.30	0.70860	0.70899	0.00011
35.26	0.70888	0.70901	0.00009
35.21	0.70913	0.70904	0.00009
35.17	0.70907	0.70905	0.00010
35.13	0.70891	0.70902	0.00011
35.08	0.70923	0.70903	0.00011
35.04	0.70899	0.70899	0.00011
34.95	0.70892	0.70896	0.00012
34.91	0.70918	0.70897	0.00012
34.87	0.70901	0.70893	0.00011
34.82	0.70878	0.70894	0.00011
34.78	0.70920	0.70894	0.00011
34.74	0.70918	0.70894	0.00011
34.69	0.70876	0.70896	0.00013
34.65	0.70908	0.70901	0.00013
34.61	0.70877	0.70897	0.00014
34.56	0.70876	0.70900	0.00014
34.52	0.70898	0.70901	0.00013
34.48	0.70881	0.70902	0.00013
34.43	0.70902	0.70902	0.00013
34.39	0.70886	0.70901	0.00013
34.35	0.70918	0.70901	0.00013
34.30	0.70933	0.70898	0.00013
34.26	0.70929	0.70896	0.00010
34.22	0.70871	0.70892	0.00007
34.17	0.70902	0.70893	0.00006
34.13	0.70886	0.70896	0.00010
34.09	0.70907	0.70897	0.00010
34.04	0.70882	0.70898	0.00011
34.00	0.70895	0.70900	0.00010
33.96	0.70891	0.70902	0.00011
33.91	0.70888	0.70905	0.00010
33.87	0.70906	0.70904	0.00011
33.83	0.70891	0.70904	0.00011
33.78	0.70882	0.70908	0.00011
33.74	0.70936	0.70910	0.00009
33.70	0.70892	0.70908	0.00008
33.65	0.70915	0.70908	0.00008
41.46	0.70902	0.70887	0.00011
41.41	0.70887	0.70891	0.00015
41.35	0.70881	0.70890	0.00015
41.30	0.70864	0.70889	0.00015
41.25	0.70882	0.70896	0.00016
41.19	0.70888	0.70897	0.00015
41.14	0.70909	0.70898	0.00015
41.08	0.70903	0.70898	0.00015
41.03	0.70858	0.70898	0.00015
40.98	0.70896	0.70900	0.00013
40.92	0.70939	0.70902	0.00013
40.87	0.70880	0.70899	0.00011
40.82	0.70876	0.70901	0.00010
40.76	0.70929	0.70902	0.00009
40.71	0.70892	0.70900	0.00007
40.65	0.70900	0.70898	0.00009
40.60	0.70907	0.70895	0.00010
40.55	0.70905	0.70890	0.00012
40.49	0.70879	0.70886	0.00012
40.44	0.70909	0.70887	0.00012
40.39	0.70917	0.70888	0.00013
40.33	0.70896	0.70887	0.00012
40.28	0.70888	0.70893	0.00019
40.23	0.70902	0.70892	0.00019
40.17	0.70874	0.70893	0.00019
40.12	0.70871	0.70895	0.00019
40.06	0.70860	0.70896	0.00019
40.01	0.70865	0.70899	0.00017
39.96	0.70887	0.70903	0.00015
39.90	0.70915	0.70905	0.00015
39.85	0.70913	0.70900	0.00017
39.80	0.70958	0.70902	0.00018
39.74	0.70878	0.70892	0.00016
39.69	0.70909	0.70894	0.00016
39.63	0.70895	0.70892	0.00015
39.58	0.70878	0.70889	0.00016
39.53	0.70886	0.70889	0.00017
39.47	0.70907	0.70889	0.00017
39.42	0.70912	0.70888	0.00016
39.37	0.70863	0.70884	0.00016
39.31	0.70936	0.70888	0.00016
39.26	0.70853	0.70880	0.00013
39.20	0.70903	0.70881	0.00012
39.15	0.70892	0.70880	0.00011
39.10	0.70863	0.70877	0.00012
39.04	0.70875	0.70880	0.00012
38.99	0.70883	0.70881	0.00012
38.94	0.70905	0.70883	0.00012
38.88	0.70866	0.70881	0.00011
38.83	0.70904	0.70884	0.00011

33.61	0.70903	0.70909	0.00009	38.78	0.70857	0.70884	0.00012
33.57	0.70918	0.70911	0.00009	38.72	0.70864	0.70891	0.00013
33.52	0.70915	0.70910	0.00009	38.67	0.70896	0.70889	0.00015
33.48	0.70883	0.70913	0.00011	38.61	0.70858	0.70887	0.00015
33.44	0.70910	0.70914	0.00010	38.56	0.70888	0.70887	0.00015
33.39	0.70922	0.70913	0.00011	38.51	0.70887	0.70888	0.00015
33.35	0.70911	0.70911	0.00010	38.45	0.70904	0.70888	0.00015
33.31	0.70911	0.70912	0.00010	38.40	0.70883	0.70886	0.00014
33.26	0.70888	0.70913	0.00011	38.35	0.70896	0.70890	0.00016
33.22	0.70931	0.70916	0.00010	38.29	0.70907	0.70888	0.00016
33.18	0.70919	0.70916	0.00010	38.24	0.70926	0.70888	0.00016
33.13	0.70915	0.70911	0.00013	38.18	0.70844	0.70884	0.00014
33.09	0.70941	0.70909	0.00014	38.13	0.70872	0.70886	0.00011
33.05	0.70893	0.70904	0.00012	38.08	0.70864	0.70888	0.00011
33.00	0.70898	0.70903	0.00012	38.02	0.70894	0.70892	0.00010
32.96	0.70905	0.70903	0.00012	37.97	0.70888	0.70892	0.00010
32.92	0.70913	0.70904	0.00013	37.92	0.70888	0.70892	0.00010
32.87	0.70930	0.70904	0.00013	37.86	0.70923	0.70892	0.00010
32.83	0.70913	0.70901	0.00012	37.81	0.70875	0.70887	0.00008
32.79	0.70931	0.70900	0.00011	37.75	0.70902	0.70886	0.00009
32.74	0.70869	0.70895	0.00009	37.70	0.70886	0.70884	0.00008
32.70	0.70896	0.70899	0.00008	37.65	0.70871	0.70882	0.00009
32.66	0.70893	0.70900	0.00008	37.59	0.70888	0.70884	0.00008
32.61	0.70882	0.70898	0.00010	37.54	0.70910	0.70885	0.00009
32.57	0.70897	0.70899	0.00009	37.49	0.70893	0.70884	0.00007
32.53	0.70912	0.70902	0.00010	37.43	0.70884	0.70885	0.00008
32.48	0.70919	0.70902	0.00010	37.38	0.70886	0.70888	0.00010
32.44	0.70897	0.70902	0.00010	37.33	0.70875	0.70889	0.00010
32.40	0.70901	0.70905	0.00011	37.27	0.70862	0.70891	0.00010
32.35	0.70884	0.70902	0.00013	37.22	0.70884	0.70895	0.00007
32.31	0.70912	0.70904	0.00012	37.16	0.70873	0.70896	0.00007
32.27	0.70905	0.70905	0.00013	37.11	0.70886	0.70894	0.00009
32.22	0.70872	0.70907	0.00013	37.06	0.70899	0.70895	0.00009
32.18	0.70897	0.70913	0.00011	37.00	0.70896	0.70891	0.00012
32.14	0.70919	0.70913	0.00011	36.95	0.70907	0.70892	0.00013
32.09	0.70916	0.70914	0.00011	36.90	0.70910	0.70889	0.00012
32.05	0.70917	0.70910	0.00013	36.84	0.70897	0.70887	0.00011
32.01	0.70930	0.70908	0.00013	36.79	0.70900	0.70883	0.00013
31.96	0.70871	0.70905	0.00012	36.73	0.70893	0.70884	0.00013
31.92	0.70906	0.70910	0.00009	36.68	0.70894	0.70883	0.00013
31.88	0.70920	0.70908	0.00010	36.63	0.70856	0.70884	0.00013
31.83	0.70921	0.70912	0.00014	36.57	0.70900	0.70887	0.00012
31.79	0.70929	0.70911	0.00014	36.52	0.70854	0.70884	0.00012
31.75	0.70900	0.70909	0.00014	36.47	0.70905	0.70888	0.00010
31.70	0.70926	0.70909	0.00014	36.41	0.70882	0.70891	0.00014
31.66	0.70883	0.70910	0.00014	36.36	0.70886	0.70891	0.00013
31.62	0.70896	0.70912	0.00013	36.30	0.70857	0.70893	0.00013
31.57	0.70901	0.70917	0.00013	36.25	0.70909	0.70891	0.00016
31.53	0.70912	0.70918	0.00013	36.20	0.70888	0.70891	0.00016
31.49	0.70892	0.70914	0.00016	36.14	0.70901	0.70890	0.00016

31.44	0.70960	0.70921	0.00017
31.40	0.70912	0.70915	0.00015
31.36	0.70910	0.70915	0.00015
31.31	0.70899	0.70910	0.00018
31.27	0.70934	0.70910	0.00018
31.23	0.70906	0.70907	0.00017
31.18	0.70939	0.70907	0.00017
31.14	0.70918	0.70905	0.00016
31.10	0.70873	0.70902	0.00016
31.05	0.70959	0.70906	0.00015
31.01	0.70903	0.70904	0.00012
30.97	0.70905	0.70907	0.00013
30.92	0.70866	0.70902	0.00016
30.88	0.70896	0.70904	0.00015
30.84	0.70904	0.70907	0.00016
30.79	0.70906	0.70906	0.00016
30.75	0.70923	0.70903	0.00017
30.71	0.70887	0.70905	0.00019
30.66	0.70913	0.70906	0.00018
30.62	0.70935	0.70901	0.00020
30.58	0.70936	0.70897	0.00018
30.53	0.70860	0.70894	0.00016
30.49	0.70882	0.70898	0.00015
30.45	0.70928	0.70899	0.00014
30.40	0.70896	0.70894	0.00013
30.36	0.70872	0.70896	0.00014
30.32	0.70941	0.70900	0.00013
30.27	0.70895	0.70897	0.00010
30.23	0.70866	0.70898	0.00010
30.19	0.70897	0.70903	0.00008
30.14	0.70899	0.70904	0.00008
30.10	0.70902	0.70902	0.00009
30.06	0.70898	0.70902	0.00009
30.01	0.70875	0.70903	0.00010
29.97	0.70911	0.70905	0.00008
29.93	0.70918	0.70906	0.00008
29.89	0.70908	0.70905	0.00008
29.84	0.70907	0.70904	0.00008
29.80	0.70919	0.70906	0.00009
29.76	0.70903	0.70903	0.00008
29.71	0.70880	0.70902	0.00009
29.67	0.70897	0.70907	0.00009
29.63	0.70915	0.70909	0.00009
29.58	0.70897	0.70905	0.00011
29.54	0.70921	0.70902	0.00014
29.50	0.70904	0.70897	0.00014
29.45	0.70898	0.70895	0.00014
29.41	0.70924	0.70895	0.00014
29.37	0.70897	0.70891	0.00013
29.32	0.70884	0.70891	0.00013

36.09	0.70884	0.70891	0.00017
36.04	0.70870	0.70895	0.00017
35.98	0.70895	0.70897	0.00016
35.93	0.70935	0.70896	0.00017
35.88	0.70888	0.70892	0.00014
35.82	0.70898	0.70890	0.00015
35.77	0.70839	0.70886	0.00016
35.71	0.70909	0.70894	0.00014
35.66	0.70882	0.70892	0.00013
35.61	0.70913	0.70894	0.00013
35.55	0.70920	0.70892	0.00012
35.50	0.70895	0.70890	0.00011
35.45	0.70882	0.70890	0.00011
35.39	0.70890	0.70892	0.00011
35.34	0.70870	0.70895	0.00012
35.28	0.70856	0.70895	0.00012
35.23	0.70918	0.70897	0.00010
35.18	0.70890	0.70894	0.00009
35.12	0.70902	0.70897	0.00010
35.07	0.70900	0.70896	0.00010
35.02	0.70897	0.70895	0.00010
34.96	0.70895	0.70893	0.00011
34.91	0.70899	0.70896	0.00011
34.85	0.70920	0.70898	0.00013
34.80	0.70873	0.70897	0.00012
34.75	0.70877	0.70897	0.00012
34.69	0.70883	0.70901	0.00012
34.64	0.70922	0.70902	0.00012
34.59	0.70894	0.70899	0.00011
34.53	0.70890	0.70897	0.00012
34.48	0.70882	0.70901	0.00014
34.43	0.70916	0.70902	0.00014
34.37	0.70926	0.70893	0.00020
34.32	0.70906	0.70889	0.00018
34.26	0.70871	0.70886	0.00018
34.21	0.70920	0.70889	0.00018
34.16	0.70895	0.70890	0.00018
34.10	0.70892	0.70891	0.00019
34.05	0.70873	0.70892	0.00019
34.00	0.70931	0.70895	0.00018
33.94	0.70888	0.70888	0.00017
33.89	0.70827	0.70886	0.00017
33.83	0.70884	0.70889	0.00014
33.78	0.70883	0.70892	0.00015
33.73	0.70899	0.70894	0.00015
33.67	0.70928	0.70897	0.00016
33.62	0.70907	0.70900	0.00019
33.57	0.70896	0.70898	0.00019
33.51	0.70903	0.70897	0.00019
33.46	0.70868	0.70898	0.00020

29.28	0.70930	0.70893	0.00013	33.40	0.70868	0.70903	0.00019
29.24	0.70917	0.70888	0.00010	33.35	0.70855	0.70912	0.00020
29.19	0.70879	0.70886	0.00008	33.30	0.70915	0.70914	0.00018
29.15	0.70862	0.70887	0.00008	33.24	0.70903	0.70914	0.00018
29.11	0.70876	0.70886	0.00009	33.19	0.70925	0.70916	0.00017
29.06	0.70879	0.70890	0.00010	33.14	0.70955	0.70914	0.00018
29.02	0.70900	0.70890	0.00010	33.08	0.70895	0.70913	0.00017
28.98	0.70882	0.70892	0.00011	33.03	0.70878	0.70917	0.00017
28.93	0.70899	0.70894	0.00011	32.98	0.70918	0.70917	0.00018
28.89	0.70903	0.70895	0.00012	32.92	0.70923	0.70917	0.00018
28.85	0.70881	0.70897	0.00012	32.87	0.70956	0.70914	0.00018
28.80	0.70895	0.70900	0.00012	32.81	0.70875	0.70909	0.00016
28.76	0.70891	0.70903	0.00013	32.76	0.70916	0.70912	0.00014
28.72	0.70855	0.70906	0.00013	32.71	0.70920	0.70911	0.00014
28.67	0.70911	0.70911	0.00007	32.65	0.70902	0.70909	0.00014
28.63	0.70887	0.70908	0.00009	32.60	0.70950	0.70911	0.00014
28.59	0.70918	0.70911	0.00008	32.55	0.70937	0.70904	0.00011
28.54	0.70901	0.70912	0.00009	32.49	0.70873	0.70901	0.00009
28.50	0.70913	0.70910	0.00011	32.44	0.70916	0.70902	0.00008
28.46	0.70914	0.70911	0.00011	32.38	0.70893	0.70902	0.00009
28.41	0.70920	0.70910	0.00011	32.33	0.70909	0.70906	0.00010
28.37	0.70918	0.70904	0.00014	32.28	0.70900	0.70906	0.00010
28.33	0.70926	0.70902	0.00014	32.22	0.70908	0.70908	0.00010
28.28	0.70901	0.70897	0.00014	32.17	0.70903	0.70908	0.00010
28.24	0.70884	0.70899	0.00015	32.12	0.70917	0.70909	0.00010
28.20	0.70918	0.70900	0.00014	32.06	0.70887	0.70909	0.00010
28.15	0.70929	0.70898	0.00014	32.01	0.70907	0.70914	0.00010
28.11	0.70878	0.70895	0.00012	31.95	0.70876	0.70914	0.00011
28.07	0.70922	0.70898	0.00012	31.90	0.70923	0.70919	0.00007
28.02	0.70902	0.70897	0.00011	31.85	0.70928	0.70917	0.00008
27.98	0.70862	0.70899	0.00012	31.79	0.70911	0.70918	0.00009
27.94	0.70897	0.70901	0.00009	31.74	0.70921	0.70913	0.00014
27.89	0.70877	0.70900	0.00010	31.69	0.70906	0.70914	0.00014
27.85	0.70924	0.70905	0.00010	31.63	0.70913	0.70915	0.00014
27.81	0.70894	0.70903	0.00009	31.58	0.70921	0.70911	0.00017
27.76	0.70895	0.70898	0.00014	31.53	0.70936	0.70907	0.00018
27.72	0.70899	0.70898	0.00014	31.47	0.70902	0.70907	0.00018
27.68	0.70910	0.70899	0.00014	31.42	0.70931	0.70908	0.00018
27.63	0.70911	0.70898	0.00014	31.36	0.70900	0.70905	0.00018
27.59	0.70917	0.70894	0.00015	31.31	0.70937	0.70902	0.00020
27.55	0.70885	0.70892	0.00014	31.26	0.70863	0.70896	0.00019
27.50	0.70885	0.70896	0.00015	31.20	0.70927	0.70901	0.00017
27.46	0.70926	0.70896	0.00015	31.15	0.70923	0.70897	0.00017
27.42	0.70907	0.70889	0.00016	31.10	0.70868	0.70892	0.00016
27.37	0.70848	0.70888	0.00015	31.04	0.70880	0.70896	0.00016
27.33	0.70892	0.70896	0.00014	30.99	0.70943	0.70903	0.00019
27.29	0.70912	0.70895	0.00014	30.93	0.70912	0.70899	0.00017
27.24	0.70896	0.70894	0.00014	30.88	0.70903	0.70901	0.00018
27.20	0.70875	0.70895	0.00014	30.83	0.70861	0.70904	0.00019
27.16	0.70895	0.70897	0.00014	30.77	0.70880	0.70912	0.00017

27.11	0.70926	0.70897	0.00014
27.07	0.70883	0.70894	0.00012
27.03	0.70854	0.70896	0.00012
26.98	0.70901	0.70900	0.00008
26.94	0.70927	0.70899	0.00009
26.90	0.70881	0.70894	0.00007
26.85	0.70898	0.70896	0.00006
26.81	0.70910	0.70893	0.00008
26.77	0.70894	0.70893	0.00009
26.72	0.70898	0.70894	0.00009
26.68	0.70890	0.70890	0.00010
26.64	0.70909	0.70889	0.00011
26.59	0.70887	0.70887	0.00010
26.55	0.70891	0.70890	0.00011
26.51	0.70877	0.70892	0.00012
26.46	0.70901	0.70893	0.00012
26.42	0.70869	0.70894	0.00012
26.38	0.70916	0.70895	0.00012
26.33	0.70898	0.70895	0.00013
26.29	0.70865	0.70897	0.00013
26.25	0.70880	0.70899	0.00011
26.20	0.70891	0.70901	0.00011
26.16	0.70914	0.70901	0.00010
26.12	0.70914	0.70897	0.00012
26.07	0.70883	0.70893	0.00012
26.03	0.70915	0.70895	0.00012
25.99	0.70871	0.70893	0.00011
25.94	0.70922	0.70897	0.00010
25.90	0.70911	0.70895	0.00009
25.86	0.70891	0.70893	0.00008
25.81	0.70893	0.70892	0.00009
25.77	0.70897	0.70893	0.00009
25.73	0.70869	0.70892	0.00009
25.68	0.70877	0.70896	0.00008
25.64	0.70906	0.70896	0.00007
25.60	0.70889	0.70895	0.00007
25.55	0.70912	0.70895	0.00007
25.51	0.70904	0.70893	0.00006
25.47	0.70892	0.70890	0.00006
25.42	0.70878	0.70889	0.00006
25.38	0.70904	0.70891	0.00006
25.34	0.70893	0.70890	0.00005
25.29	0.70903	0.70892	0.00007
25.25	0.70880	0.70890	0.00006
25.21	0.70899	0.70890	0.00006
25.16	0.70885	0.70891	0.00008
25.12	0.70890	0.70889	0.00010
25.08	0.70879	0.70888	0.00010
25.03	0.70883	0.70889	0.00010
24.99	0.70892	0.70889	0.00010

30.72	0.70916	0.70912	0.00017
30.67	0.70880	0.70913	0.00017
30.61	0.70876	0.70914	0.00016
30.56	0.70909	0.70919	0.00013
30.50	0.70954	0.70914	0.00018
30.45	0.70901	0.70912	0.00016
30.40	0.70929	0.70917	0.00017
30.34	0.70937	0.70917	0.00017
30.29	0.70935	0.70912	0.00018
30.24	0.70885	0.70907	0.00017
30.18	0.70920	0.70908	0.00017
30.13	0.70897	0.70904	0.00018
30.08	0.70925	0.70905	0.00017
30.02	0.70860	0.70905	0.00018
29.97	0.70932	0.70908	0.00015
29.91	0.70947	0.70906	0.00015
29.86	0.70931	0.70899	0.00012
29.81	0.70885	0.70890	0.00015
29.75	0.70893	0.70899	0.00023
29.70	0.70894	0.70899	0.00023
29.65	0.70880	0.70895	0.00025
29.59	0.70907	0.70897	0.00025
29.54	0.70926	0.70900	0.00026
29.48	0.70882	0.70894	0.00026
29.43	0.70912	0.70892	0.00027
29.38	0.70880	0.70893	0.00027
29.32	0.70840	0.70897	0.00027
29.27	0.70976	0.70902	0.00024
29.22	0.70894	0.70892	0.00018
29.16	0.70848	0.70895	0.00019
29.11	0.70906	0.70902	0.00016
29.05	0.70936	0.70895	0.00021
29.00	0.70865	0.70899	0.00026
28.95	0.70866	0.70902	0.00025
28.89	0.70921	0.70903	0.00025
28.84	0.70918	0.70900	0.00025
28.79	0.70891	0.70898	0.00024
28.73	0.70880	0.70897	0.00024
28.68	0.70916	0.70900	0.00024
28.62	0.70924	0.70903	0.00026
28.57	0.70833	0.70908	0.00030
28.52	0.70982	0.70914	0.00025
28.46	0.70887	0.70907	0.00020
28.41	0.70876	0.70911	0.00020
28.36	0.70895	0.70914	0.00019
28.30	0.70899	0.70915	0.00019
28.25	0.70880	0.70916	0.00018
28.20	0.70904	0.70918	0.00017
28.14	0.70948	0.70920	0.00016
28.09	0.70978	0.70916	0.00015

24.95	0.70898	0.70891	0.00011	28.03	0.70889	0.70908	0.00008
24.90	0.70911	0.70890	0.00011	27.98	0.70917	0.70912	0.00008
24.86	0.70885	0.70888	0.00010	27.93	0.70928	0.70911	0.00008
24.82	0.70880	0.70891	0.00012	27.87	0.70899	0.70914	0.00012
24.77	0.70912	0.70893	0.00011	27.82	0.70907	0.70913	0.00013
24.73	0.70862	0.70889	0.00011	27.77	0.70907	0.70913	0.00013
24.69	0.70875	0.70893	0.00009	27.71	0.70908	0.70912	0.00013
24.64	0.70892	0.70896	0.00008	27.66	0.70921	0.70914	0.00013
24.60	0.70883	0.70896	0.00008	27.60	0.70908	0.70915	0.00014
24.56	0.70916	0.70895	0.00009	27.55	0.70894	0.70914	0.00015
24.51	0.70886	0.70891	0.00009	27.50	0.70934	0.70912	0.00016
24.47	0.70888	0.70892	0.00009	27.44	0.70906	0.70908	0.00015
24.43	0.70916	0.70890	0.00010	27.39	0.70958	0.70909	0.00015
24.38	0.70900	0.70887	0.00008	27.34	0.70885	0.70902	0.00011
24.34	0.70877	0.70885	0.00008	27.28	0.70905	0.70902	0.00011
24.30	0.70897	0.70886	0.00008	27.23	0.70904	0.70906	0.00013
24.25	0.70905	0.70887	0.00009	27.17	0.70922	0.70906	0.00013
24.21	0.70896	0.70886	0.00008	27.12	0.70935	0.70900	0.00015
24.17	0.70873	0.70887	0.00010	27.07	0.70892	0.70901	0.00017
24.12	0.70876	0.70888	0.00010	27.01	0.70881	0.70903	0.00017
24.08	0.70895	0.70888	0.00010	26.96	0.70895	0.70905	0.00016
24.04	0.70865	0.70885	0.00010	26.91	0.70910	0.70905	0.00016
23.99	0.70886	0.70888	0.00010	26.85	0.70886	0.70906	0.00017
23.95	0.70875	0.70888	0.00010	26.80	0.70889	0.70912	0.00018
23.91	0.70892	0.70892	0.00010	26.75	0.70941	0.70917	0.00017
23.86	0.70907	0.70895	0.00012	26.69	0.70908	0.70911	0.00018
23.82	0.70889	0.70894	0.00011	26.64	0.70860	0.70908	0.00019
23.78	0.70916	0.70890	0.00014	26.58	0.70947	0.70914	0.00016
23.73	0.70878	0.70888	0.00013	26.53	0.70915	0.70915	0.00017
23.69	0.70871	0.70891	0.00014	26.48	0.70894	0.70912	0.00019
23.65	0.70870	0.70894	0.00013	26.42	0.70898	0.70906	0.00024
23.60	0.70901	0.70897	0.00012	26.37	0.70926	0.70906	0.00024
23.56	0.70886	0.70897	0.00012	26.32	0.70945	0.70902	0.00024
23.52	0.70910	0.70900	0.00012	26.26	0.70934	0.70892	0.00024
23.47	0.70921	0.70897	0.00012	26.21	0.70879	0.70889	0.00023
23.43	0.70896	0.70897	0.00012	26.15	0.70883	0.70893	0.00023
23.39	0.70852	0.70897	0.00012	26.10	0.70920	0.70894	0.00023
23.34	0.70896	0.70899	0.00010	26.05	0.70960	0.70890	0.00023
23.30	0.70913	0.70898	0.00010	25.99	0.70878	0.70887	0.00018
23.26	0.70901	0.70896	0.00009	25.94	0.70834	0.70891	0.00019
23.22	0.70894	0.70894	0.00010	25.89	0.70897	0.70896	0.00014
23.17	0.70899	0.70894	0.00010	25.83	0.70886	0.70897	0.00015
23.13	0.70918	0.70894	0.00010	25.78	0.70847	0.70901	0.00015
23.09	0.70886	0.70894	0.00010	25.72	0.70907	0.70906	0.00010
23.04	0.70914	0.70893	0.00010	25.67	0.70922	0.70905	0.00010
23.00	0.70903	0.70891	0.00009	25.62	0.70885	0.70902	0.00009
22.96	0.70866	0.70889	0.00009	25.56	0.70888	0.70904	0.00009
22.91	0.70889	0.70894	0.00008	25.51	0.70924	0.70911	0.00012
22.87	0.70891	0.70897	0.00010	25.46	0.70915	0.70913	0.00014
22.83	0.70876	0.70897	0.00010	25.40	0.70890	0.70912	0.00015

22.78	0.70901	0.70899	0.00009	25.35	0.70907	0.70914	0.00014
22.74	0.70897	0.70898	0.00009	25.30	0.70924	0.70915	0.00014
22.70	0.70912	0.70896	0.00010	25.24	0.70897	0.70917	0.00014
22.65	0.70881	0.70892	0.00011	25.19	0.70902	0.70920	0.00014
22.61	0.70897	0.70896	0.00013	25.13	0.70887	0.70924	0.00014
22.57	0.70879	0.70897	0.00013	25.08	0.70909	0.70926	0.00013
22.52	0.70916	0.70898	0.00012	25.03	0.70952	0.70928	0.00012
22.48	0.70923	0.70896	0.00012	24.97	0.70950	0.70923	0.00012
22.44	0.70884	0.70892	0.00010	24.92	0.70899	0.70919	0.00011
22.39	0.70896	0.70891	0.00011	24.87	0.70916	0.70921	0.00010
22.35	0.70896	0.70889	0.00011	24.81	0.70918	0.70919	0.00010
22.31	0.70877	0.70888	0.00011	24.76	0.70937	0.70919	0.00011
22.26	0.70866	0.70889	0.00011	24.70	0.70926	0.70917	0.00010
22.22	0.70924	0.70891	0.00010	24.65	0.70951	0.70916	0.00009
22.18	0.70909	0.70886	0.00007	24.60	0.70906	0.70910	0.00006
22.13	0.70893	0.70880	0.00008	24.54	0.70926	0.70911	0.00006
22.09	0.70892	0.70879	0.00007	24.49	0.70897	0.70908	0.00006
22.05	0.70880	0.70878	0.00006	24.44	0.70916	0.70908	0.00005
22.00	0.70878	0.70877	0.00006	24.38	0.70916	0.70910	0.00008
21.96	0.70873	0.70880	0.00008	24.33	0.70902	0.70911	0.00008
21.92	0.70887	0.70883	0.00009	24.27	0.70909	0.70913	0.00009
21.87	0.70887	0.70884	0.00010	24.22	0.70919	0.70908	0.00015
21.83	0.70888	0.70885	0.00010	24.17	0.70914	0.70905	0.00015
21.79	0.70870	0.70884	0.00010	24.11	0.70895	0.70906	0.00016
21.74	0.70854	0.70887	0.00010	24.06	0.70912	0.70906	0.00016
21.70	0.70882	0.70891	0.00007	24.01	0.70898	0.70905	0.00016
21.66	0.70877	0.70894	0.00008	23.95	0.70899	0.70902	0.00017
21.61	0.70874	0.70896	0.00007	23.90	0.70938	0.70904	0.00017
21.57	0.70904	0.70897	0.00006	23.85	0.70920	0.70902	0.00016
21.53	0.70904	0.70895	0.00005	23.79	0.70930	0.70899	0.00015
21.48	0.70903	0.70892	0.00006	23.74	0.70854	0.70898	0.00014
21.44	0.70889	0.70893	0.00008	23.68	0.70889	0.70903	0.00010
21.40	0.70887	0.70896	0.00009	23.63	0.70927	0.70908	0.00012
21.35	0.70891	0.70899	0.00009	23.58	0.70890	0.70903	0.00012
21.31	0.70903	0.70901	0.00010	23.52	0.70902	0.70904	0.00012
21.27	0.70908	0.70902	0.00010	23.47	0.70875	0.70900	0.00015
21.22	0.70892	0.70902	0.00010	23.42	0.70913	0.70898	0.00017
21.18	0.70884	0.70899	0.00012	23.36	0.70917	0.70896	0.00016
21.14	0.70890	0.70900	0.00012	23.31	0.70893	0.70896	0.00016
21.09	0.70874	0.70905	0.00015	23.25	0.70916	0.70894	0.00017
21.05	0.70914	0.70908	0.00013	23.20	0.70912	0.70891	0.00016
21.01	0.70922	0.70905	0.00014	23.15	0.70937	0.70891	0.00016
20.96	0.70908	0.70902	0.00013	23.09	0.70876	0.70885	0.00013
20.92	0.70919	0.70902	0.00013	23.04	0.70902	0.70884	0.00014
20.88	0.70915	0.70901	0.00013	22.99	0.70861	0.70883	0.00013
20.83	0.70900	0.70899	0.00013	22.93	0.70856	0.70882	0.00014
20.79	0.70868	0.70897	0.00013	22.88	0.70890	0.70889	0.00015
20.75	0.70889	0.70900	0.00011	22.82	0.70916	0.70887	0.00015
20.70	0.70945	0.70899	0.00012	22.77	0.70872	0.70887	0.00015
20.66	0.70900	0.70895	0.00007	22.72	0.70887	0.70889	0.00015

20.62	0.70883	0.70892	0.00009
20.57	0.70896	0.70893	0.00009
20.53	0.70904	0.70893	0.00009
20.49	0.70905	0.70893	0.00009
20.44	0.70898	0.70893	0.00009
20.40	0.70881	0.70893	0.00009
20.36	0.70901	0.70896	0.00009
20.31	0.70873	0.70895	0.00009
20.27	0.70909	0.70901	0.00009
20.23	0.70867	0.70901	0.00010
20.18	0.70892	0.70904	0.00007
20.14	0.70901	0.70905	0.00006
20.10	0.70905	0.70910	0.00011
20.05	0.70899	0.70909	0.00011
20.01	0.70906	0.70909	0.00011
19.97	0.70907	0.70907	0.00012
19.92	0.70897	0.70907	0.00012
19.88	0.70926	0.70908	0.00012
19.84	0.70917	0.70908	0.00012
19.79	0.70894	0.70905	0.00012
19.75	0.70897	0.70907	0.00012
19.71	0.70950	0.70906	0.00013
19.66	0.70900	0.70903	0.00010
19.62	0.70899	0.70903	0.00010
19.58	0.70883	0.70898	0.00014
19.53	0.70911	0.70901	0.00014
19.49	0.70908	0.70900	0.00014
19.45	0.70919	0.70900	0.00014
19.40	0.70894	0.70897	0.00013
19.36	0.70912	0.70898	0.00013
19.32	0.70881	0.70896	0.00013
19.27	0.70928	0.70898	0.00013
19.23	0.70890	0.70901	0.00016
19.19	0.70852	0.70904	0.00016
19.14	0.70910	0.70904	0.00016
19.10	0.70908	0.70902	0.00016
19.06	0.70908	0.70902	0.00016
19.01	0.70889	0.70901	0.00016
18.97	0.70905	0.70903	0.00016
18.93	0.70890	0.70904	0.00016
18.88	0.70898	0.70902	0.00018
18.84	0.70955	0.70901	0.00018
18.80	0.70921	0.70894	0.00013
18.75	0.70855	0.70894	0.00013
18.71	0.70889	0.70900	0.00010
18.67	0.70908	0.70897	0.00013
18.62	0.70899	0.70895	0.00013
18.58	0.70906	0.70894	0.00013
18.54	0.70918	0.70892	0.00012
18.49	0.70868	0.70890	0.00011

22.66	0.70914	0.70888	0.00015
22.61	0.70880	0.70885	0.00014
22.56	0.70859	0.70885	0.00014
22.50	0.70891	0.70887	0.00012
22.45	0.70857	0.70888	0.00013
22.40	0.70922	0.70891	0.00010
22.34	0.70873	0.70886	0.00009
22.29	0.70915	0.70888	0.00008
22.23	0.70896	0.70886	0.00006
22.18	0.70876	0.70888	0.00008
22.13	0.70879	0.70889	0.00007
22.07	0.70880	0.70894	0.00010
22.02	0.70885	0.70896	0.00010
21.97	0.70897	0.70895	0.00011
21.91	0.70890	0.70896	0.00011
21.86	0.70868	0.70895	0.00012
21.80	0.70897	0.70896	0.00011
21.75	0.70893	0.70895	0.00011
21.70	0.70911	0.70896	0.00011
21.64	0.70892	0.70897	0.00011
21.59	0.70925	0.70897	0.00011
21.54	0.70903	0.70892	0.00011
21.48	0.70872	0.70891	0.00011
21.43	0.70910	0.70892	0.00010
21.37	0.70878	0.70893	0.00010
21.32	0.70879	0.70891	0.00012
21.27	0.70892	0.70892	0.00012
21.21	0.70900	0.70894	0.00012
21.16	0.70920	0.70895	0.00013
21.11	0.70894	0.70892	0.00011
21.05	0.70869	0.70891	0.00012
21.00	0.70898	0.70895	0.00012
20.95	0.70884	0.70893	0.00012
20.89	0.70912	0.70893	0.00012
20.84	0.70858	0.70890	0.00012
20.78	0.70893	0.70891	0.00010
20.73	0.70912	0.70887	0.00014
20.68	0.70910	0.70886	0.00013
20.62	0.70892	0.70882	0.00012
20.57	0.70879	0.70881	0.00012
20.52	0.70914	0.70881	0.00012
20.46	0.70874	0.70875	0.00011
20.41	0.70888	0.70873	0.00012
20.35	0.70874	0.70876	0.00015
20.30	0.70877	0.70881	0.00018
20.25	0.70846	0.70885	0.00019
20.19	0.70909	0.70886	0.00019
20.14	0.70869	0.70882	0.00018
20.09	0.70877	0.70888	0.00021
20.03	0.70878	0.70890	0.00020

18.45	0.70895	0.70895	0.00011	19.98	0.70856	0.70892	0.00020
18.41	0.70884	0.70893	0.00012	19.92	0.70852	0.70898	0.00019
18.36	0.70915	0.70896	0.00012	19.87	0.70923	0.70904	0.00016
18.32	0.70914	0.70893	0.00012	19.82	0.70923	0.70900	0.00016
18.28	0.70859	0.70889	0.00011	19.76	0.70918	0.70898	0.00016
18.23	0.70888	0.70893	0.00009	19.71	0.70851	0.70898	0.00016
18.19	0.70893	0.70895	0.00009	19.66	0.70869	0.70903	0.00012
18.15	0.70890	0.70894	0.00009	19.60	0.70932	0.70905	0.00010
18.10	0.70893	0.70896	0.00010	19.55	0.70897	0.70894	0.00018
18.06	0.70921	0.70898	0.00010	19.50	0.70905	0.70896	0.00018
18.02	0.70874	0.70892	0.00011	19.44	0.70911	0.70895	0.00018
17.97	0.70910	0.70894	0.00010	19.39	0.70914	0.70894	0.00018
17.93	0.70887	0.70894	0.00010	19.33	0.70877	0.70895	0.00018
17.89	0.70876	0.70890	0.00013	19.28	0.70910	0.70898	0.00018
17.84	0.70902	0.70890	0.00013	19.23	0.70918	0.70897	0.00018
17.80	0.70905	0.70891	0.00014	19.17	0.70903	0.70895	0.00017
17.76	0.70885	0.70888	0.00014	19.12	0.70883	0.70897	0.00018
17.71	0.70910	0.70886	0.00015	19.07	0.70823	0.70895	0.00020
17.67	0.70907	0.70884	0.00014	19.01	0.70912	0.70901	0.00012
17.63	0.70864	0.70883	0.00013	18.96	0.70900	0.70896	0.00015
17.58	0.70893	0.70885	0.00012	18.90	0.70904	0.70895	0.00015
17.54	0.70910	0.70882	0.00013	18.85	0.70925	0.70895	0.00015
17.50	0.70852	0.70880	0.00011	18.80	0.70906	0.70892	0.00014
17.45	0.70870	0.70882	0.00010	18.74	0.70891	0.70888	0.00015
17.41	0.70911	0.70885	0.00010	18.69	0.70904	0.70886	0.00015
17.37	0.70880	0.70879	0.00010	18.64	0.70926	0.70883	0.00014
17.32	0.70861	0.70881	0.00011	18.58	0.70858	0.70876	0.00012
17.28	0.70897	0.70882	0.00010	18.53	0.70889	0.70875	0.00013
17.24	0.70893	0.70881	0.00010	18.47	0.70854	0.70873	0.00013
17.19	0.70880	0.70882	0.00010	18.42	0.70896	0.70878	0.00012
17.15	0.70866	0.70880	0.00011	18.37	0.70904	0.70878	0.00013
17.11	0.70892	0.70883	0.00011	18.31	0.70892	0.70873	0.00013
17.06	0.70873	0.70883	0.00011	18.26	0.70862	0.70872	0.00012
17.02	0.70899	0.70884	0.00010	18.21	0.70870	0.70876	0.00013
16.98	0.70851	0.70883	0.00010	18.15	0.70879	0.70878	0.00013
16.93	0.70897	0.70887	0.00007	18.10	0.70853	0.70880	0.00014
16.89	0.70871	0.70888	0.00008	18.05	0.70846	0.70882	0.00013
16.85	0.70891	0.70891	0.00007	17.99	0.70879	0.70887	0.00010
16.80	0.70896	0.70893	0.00008	17.94	0.70896	0.70889	0.00010
16.76	0.70865	0.70892	0.00008	17.88	0.70901	0.70892	0.00012
16.72	0.70894	0.70892	0.00009	17.83	0.70850	0.70887	0.00014
16.67	0.70891	0.70887	0.00012	17.78	0.70888	0.70893	0.00013
16.63	0.70884	0.70887	0.00012	17.72	0.70902	0.70891	0.00014
16.59	0.70888	0.70886	0.00012	17.67	0.70885	0.70886	0.00015
16.55	0.70891	0.70888	0.00012	17.62	0.70901	0.70880	0.00019
16.50	0.70908	0.70885	0.00012	17.56	0.70874	0.70879	0.00019
16.46	0.70899	0.70884	0.00012	17.51	0.70896	0.70879	0.00019
16.42	0.70910	0.70880	0.00012	17.45	0.70898	0.70875	0.00019
16.37	0.70888	0.70878	0.00010	17.40	0.70922	0.70872	0.00019
16.33	0.70861	0.70880	0.00012	17.35	0.70850	0.70864	0.00015

16.29	0.70852	0.70881	0.00011	17.29	0.70910	0.70862	0.00016
16.24	0.70885	0.70882	0.00010	17.24	0.70867	0.70856	0.00013
16.20	0.70880	0.70882	0.00010	17.19	0.70860	0.70854	0.00013
16.16	0.70900	0.70884	0.00010	17.13	0.70824	0.70859	0.00017
16.11	0.70870	0.70885	0.00011	17.08	0.70891	0.70861	0.00015
16.07	0.70895	0.70886	0.00011	17.02	0.70875	0.70860	0.00014
16.03	0.70860	0.70886	0.00010	16.97	0.70851	0.70857	0.00014
15.98	0.70888	0.70889	0.00009	16.92	0.70864	0.70862	0.00016
15.94	0.70906	0.70892	0.00011	16.86	0.70850	0.70861	0.00016
15.90	0.70869	0.70892	0.00011	16.81	0.70830	0.70862	0.00016
15.85	0.70871	0.70893	0.00010	16.76	0.70844	0.70869	0.00015
15.81	0.70885	0.70896	0.00009	16.70	0.70850	0.70872	0.00014
15.77	0.70891	0.70897	0.00009	16.65	0.70909	0.70874	0.00014
15.72	0.70913	0.70897	0.00009	16.59	0.70852	0.70869	0.00012
15.68	0.70877	0.70894	0.00009	16.54	0.70873	0.70869	0.00012
15.64	0.70894	0.70894	0.00009	16.49	0.70847	0.70875	0.00017
15.59	0.70892	0.70893	0.00009	16.43	0.70902	0.70878	0.00015
15.55	0.70919	0.70893	0.00009	16.38	0.70854	0.70878	0.00015
15.51	0.70911	0.70888	0.00009	16.33	0.70864	0.70878	0.00015
15.46	0.70879	0.70887	0.00008	16.27	0.70893	0.70880	0.00015
15.42	0.70894	0.70885	0.00009	16.22	0.70881	0.70876	0.00015
15.38	0.70903	0.70887	0.00011	16.17	0.70871	0.70874	0.00016
15.33	0.70893	0.70889	0.00013	16.11	0.70850	0.70872	0.00016
15.29	0.70875	0.70888	0.00013	16.06	0.70859	0.70873	0.00016
15.25	0.70878	0.70892	0.00013	16.00	0.70928	0.70877	0.00016
15.20	0.70892	0.70894	0.00013	15.95	0.70879	0.70872	0.00011
15.16	0.70887	0.70890	0.00015	15.90	0.70899	0.70869	0.00012
15.12	0.70865	0.70887	0.00016	15.84	0.70853	0.70870	0.00013
15.07	0.70903	0.70892	0.00016	15.79	0.70882	0.70876	0.00015
15.03	0.70860	0.70891	0.00016	15.74	0.70860	0.70872	0.00017
14.99	0.70915	0.70893	0.00015	15.68	0.70855	0.70871	0.00017
14.94	0.70921	0.70893	0.00015	15.63	0.70854	0.70873	0.00017
14.90	0.70887	0.70890	0.00014	15.57	0.70860	0.70877	0.00017
14.86	0.70916	0.70893	0.00015	15.52	0.70899	0.70877	0.00017
14.81	0.70891	0.70893	0.00015	15.47	0.70875	0.70876	0.00016
14.77	0.70859	0.70892	0.00016	15.41	0.70855	0.70874	0.00017
14.73	0.70856	0.70899	0.00016	15.36	0.70906	0.70873	0.00018
14.68	0.70914	0.70900	0.00015	15.31	0.70916	0.70873	0.00018
14.64	0.70886	0.70896	0.00015	15.25	0.70837	0.70867	0.00015
14.60	0.70882	0.70899	0.00016	15.20	0.70850	0.70872	0.00014
14.55	0.70922	0.70900	0.00015	15.14	0.70877	0.70876	0.00013
14.51	0.70886	0.70898	0.00014	15.09	0.70892	0.70878	0.00014
14.47	0.70916	0.70900	0.00014	15.04	0.70861	0.70878	0.00014
14.42	0.70921	0.70896	0.00015	14.98	0.70895	0.70881	0.00014
14.38	0.70874	0.70896	0.00015	14.93	0.70848	0.70880	0.00013
14.34	0.70933	0.70901	0.00015	14.88	0.70847	0.70882	0.00011
14.29	0.70861	0.70896	0.00013	14.82	0.70906	0.70885	0.00009
14.25	0.70880	0.70897	0.00012	14.77	0.70857	0.70886	0.00009
14.21	0.70912	0.70899	0.00012	14.72	0.70889	0.70888	0.00007
14.16	0.70894	0.70898	0.00011	14.66	0.70890	0.70889	0.00008

14.12	0.70902	0.70897	0.00012	14.61	0.70898	0.70891	0.00009
14.08	0.70908	0.70894	0.00012	14.55	0.70889	0.70888	0.00010
14.03	0.70870	0.70896	0.00014	14.50	0.70892	0.70885	0.00011
13.99	0.70921	0.70900	0.00013	14.45	0.70880	0.70884	0.00011
13.95	0.70924	0.70897	0.00012	14.39	0.70874	0.70887	0.00012
13.90	0.70886	0.70893	0.00011	14.34	0.70878	0.70891	0.00013
13.86	0.70873	0.70894	0.00011	14.29	0.70912	0.70888	0.00015
13.82	0.70901	0.70899	0.00010	14.23	0.70873	0.70885	0.00014
13.77	0.70900	0.70898	0.00011	14.18	0.70903	0.70888	0.00015
13.73	0.70882	0.70900	0.00012	14.12	0.70907	0.70886	0.00014
13.69	0.70878	0.70901	0.00012	14.07	0.70869	0.70886	0.00014
13.64	0.70928	0.70907	0.00011	14.02	0.70861	0.70891	0.00015
13.60	0.70911	0.70904	0.00010	13.96	0.70879	0.70897	0.00014
13.56	0.70891	0.70903	0.00010	13.91	0.70910	0.70898	0.00014
13.51	0.70882	0.70906	0.00011	13.86	0.70912	0.70899	0.00014
13.47	0.70898	0.70911	0.00010	13.80	0.70851	0.70897	0.00014
13.43	0.70917	0.70912	0.00009	13.75	0.70881	0.70903	0.00010
13.38	0.70888	0.70909	0.00010	13.69	0.70909	0.70909	0.00011
13.34	0.70927	0.70909	0.00010	13.64	0.70883	0.70907	0.00011
13.30	0.70895	0.70909	0.00010	13.59	0.70902	0.70908	0.00011
13.25	0.70930	0.70909	0.00010	13.53	0.70924	0.70908	0.00011
13.21	0.70904	0.70903	0.00012	13.48	0.70915	0.70910	0.00013
13.17	0.70899	0.70900	0.00013	13.43	0.70893	0.70902	0.00019
13.12	0.70922	0.70898	0.00014	13.37	0.70921	0.70900	0.00020
13.08	0.70927	0.70894	0.00013	13.32	0.70892	0.70896	0.00019
13.04	0.70908	0.70884	0.00017	13.27	0.70908	0.70897	0.00019
12.99	0.70889	0.70882	0.00016	13.21	0.70941	0.70898	0.00020
12.95	0.70891	0.70879	0.00016	13.16	0.70894	0.70893	0.00017
12.91	0.70923	0.70879	0.00016	13.10	0.70886	0.70892	0.00017
12.86	0.70900	0.70874	0.00013	13.05	0.70904	0.70893	0.00017
12.82	0.70863	0.70871	0.00012	13.00	0.70942	0.70892	0.00017
12.78	0.70874	0.70873	0.00012	12.94	0.70840	0.70884	0.00014
12.73	0.70885	0.70872	0.00012	12.89	0.70874	0.70887	0.00011
12.69	0.70883	0.70871	0.00012	12.84	0.70877	0.70886	0.00011
12.65	0.70825	0.70873	0.00013	12.78	0.70903	0.70885	0.00011
12.60	0.70885	0.70877	0.00008	12.73	0.70916	0.70883	0.00011
12.56	0.70864	0.70880	0.00012	12.67	0.70898	0.70876	0.00011
12.52	0.70891	0.70882	0.00011	12.62	0.70881	0.70871	0.00010
12.47	0.70869	0.70887	0.00015	12.57	0.70899	0.70872	0.00010
12.43	0.70871	0.70890	0.00015	12.51	0.70887	0.70868	0.00009
12.39	0.70880	0.70895	0.00016	12.46	0.70862	0.70866	0.00008
12.34	0.70865	0.70895	0.00016	12.41	0.70872	0.70867	0.00008
12.30	0.70873	0.70897	0.00015	12.35	0.70869	0.70869	0.00009
12.26	0.70903	0.70902	0.00015	12.30	0.70867	0.70870	0.00010
12.21	0.70868	0.70901	0.00015	12.24	0.70881	0.70871	0.00010
12.17	0.70919	0.70905	0.00013	12.19	0.70844	0.70868	0.00010
12.13	0.70883	0.70905	0.00013	12.14	0.70853	0.70871	0.00008
12.08	0.70936	0.70902	0.00016	12.08	0.70884	0.70870	0.00009
12.04	0.70896	0.70897	0.00014	12.03	0.70858	0.70870	0.00009
12.00	0.70924	0.70897	0.00014	11.98	0.70871	0.70872	0.00009

11.95	0.70883	0.70891	0.00014
11.91	0.70880	0.70891	0.00014
11.87	0.70928	0.70890	0.00015
11.82	0.70889	0.70885	0.00012
11.78	0.70912	0.70889	0.00014
11.74	0.70914	0.70890	0.00015
11.69	0.70857	0.70885	0.00015
11.65	0.70882	0.70890	0.00014
11.61	0.70898	0.70894	0.00015
11.56	0.70871	0.70894	0.00015
11.52	0.70879	0.70896	0.00014
11.48	0.70868	0.70897	0.00014
11.43	0.70882	0.70897	0.00014
11.39	0.70924	0.70897	0.00014
11.35	0.70926	0.70891	0.00014
11.30	0.70867	0.70887	0.00011
11.26	0.70900	0.70888	0.00011
11.22	0.70921	0.70889	0.00012
11.17	0.70899	0.70887	0.00010
11.13	0.70893	0.70887	0.00010
11.09	0.70892	0.70886	0.00010
11.04	0.70867	0.70887	0.00010
11.00	0.70879	0.70890	0.00009
10.96	0.70863	0.70890	0.00009
10.91	0.70891	0.70892	0.00007
10.87	0.70877	0.70893	0.00008
10.83	0.70912	0.70895	0.00007
10.78	0.70899	0.70893	0.00006
10.74	0.70896	0.70891	0.00007
10.70	0.70884	0.70889	0.00007
10.65	0.70897	0.70889	0.00007
10.61	0.70904	0.70894	0.00012
10.57	0.70878	0.70891	0.00012
10.52	0.70881	0.70891	0.00012
10.48	0.70904	0.70893	0.00012
10.44	0.70896	0.70889	0.00013
10.39	0.70892	0.70887	0.00013
10.35	0.70875	0.70893	0.00018
10.31	0.70879	0.70897	0.00019
10.26	0.70886	0.70894	0.00021
10.22	0.70940	0.70896	0.00021
10.18	0.70883	0.70892	0.00018
10.13	0.70874	0.70892	0.00018
10.09	0.70900	0.70895	0.00018
10.05	0.70867	0.70897	0.00019
10.00	0.70873	0.70900	0.00017
9.96	0.70949	0.70903	0.00016
9.92	0.70921	0.70898	0.00013
9.88	0.70848	0.70897	0.00012
9.83	0.70901	0.70901	0.00006

11.92	0.70872	0.70872	0.00009
11.87	0.70893	0.70873	0.00010
11.82	0.70878	0.70874	0.00010
11.76	0.70877	0.70872	0.00010
11.71	0.70853	0.70872	0.00010
11.65	0.70871	0.70875	0.00009
11.60	0.70846	0.70873	0.00011
11.55	0.70885	0.70873	0.00011
11.49	0.70873	0.70869	0.00011
11.44	0.70868	0.70869	0.00011
11.39	0.70889	0.70872	0.00012
11.33	0.70896	0.70869	0.00011
11.28	0.70862	0.70869	0.00011
11.22	0.70879	0.70866	0.00012
11.17	0.70884	0.70866	0.00012
11.12	0.70846	0.70865	0.00012
11.06	0.70847	0.70867	0.00011
11.01	0.70849	0.70869	0.00010
10.96	0.70872	0.70868	0.00011
10.90	0.70890	0.70873	0.00015
10.85	0.70868	0.70872	0.00014
10.79	0.70887	0.70869	0.00016
10.74	0.70838	0.70870	0.00017
10.69	0.70880	0.70876	0.00016
10.63	0.70874	0.70873	0.00017
10.58	0.70862	0.70871	0.00017
10.53	0.70864	0.70874	0.00017
10.47	0.70845	0.70876	0.00017
10.42	0.70919	0.70880	0.00016
10.37	0.70877	0.70876	0.00013
10.31	0.70838	0.70875	0.00013
10.26	0.70904	0.70877	0.00011
10.20	0.70895	0.70873	0.00009
10.15	0.70848	0.70869	0.00009
10.10	0.70862	0.70870	0.00008
10.04	0.70891	0.70870	0.00008
9.99	0.70877	0.70865	0.00009
9.94	0.70888	0.70864	0.00009
9.88	0.70876	0.70863	0.00008
9.83	0.70870	0.70862	0.00008
9.77	0.70861	0.70864	0.00009
9.72	0.70866	0.70862	0.00010
9.67	0.70856	0.70861	0.00010
9.61	0.70852	0.70865	0.00013
9.56	0.70869	0.70865	0.00013
9.51	0.70839	0.70865	0.00013
9.45	0.70859	0.70869	0.00013
9.40	0.70887	0.70869	0.00013
9.34	0.70866	0.70869	0.00012
9.29	0.70883	0.70872	0.00013

9.79	0.70903	0.70897	0.00010
9.75	0.70883	0.70894	0.00011
9.70	0.70907	0.70896	0.00011
9.66	0.70915	0.70894	0.00010
9.62	0.70899	0.70892	0.00009
9.57	0.70901	0.70891	0.00009
9.53	0.70905	0.70890	0.00009
9.49	0.70904	0.70888	0.00008
9.44	0.70888	0.70890	0.00010
9.40	0.70863	0.70890	0.00010
9.36	0.70871	0.70894	0.00009
9.31	0.70903	0.70897	0.00007
9.27	0.70894	0.70895	0.00007
9.23	0.70895	0.70896	0.00008
9.18	0.70886	0.70897	0.00008
9.14	0.70888	0.70901	0.00010
9.10	0.70890	0.70899	0.00013
9.05	0.70922	0.70897	0.00014
9.01	0.70886	0.70893	0.00013
8.97	0.70907	0.70890	0.00015
8.92	0.70898	0.70890	0.00015
8.88	0.70885	0.70889	0.00014
8.84	0.70907	0.70890	0.00014
8.79	0.70897	0.70887	0.00014
8.75	0.70933	0.70890	0.00016
8.71	0.70863	0.70888	0.00014
8.66	0.70871	0.70890	0.00013
8.62	0.70887	0.70891	0.00012
8.58	0.70857	0.70891	0.00012
8.53	0.70906	0.70894	0.00010
8.49	0.70884	0.70892	0.00010
8.45	0.70896	0.70894	0.00010
8.40	0.70879	0.70896	0.00010
8.36	0.70926	0.70894	0.00013
8.32	0.70909	0.70891	0.00011
8.27	0.70880	0.70886	0.00012
8.23	0.70890	0.70888	0.00012
8.19	0.70879	0.70890	0.00013
8.14	0.70886	0.70896	0.00016
8.10	0.70893	0.70896	0.00016
8.06	0.70904	0.70895	0.00016
8.01	0.70913	0.70895	0.00015
7.97	0.70856	0.70893	0.00015
7.93	0.70904	0.70896	0.00013
7.88	0.70858	0.70894	0.00013
7.84	0.70892	0.70901	0.00012
7.80	0.70915	0.70902	0.00012
7.75	0.70935	0.70902	0.00012
7.71	0.70887	0.70897	0.00009
7.67	0.70890	0.70900	0.00010

9.24	0.70847	0.70874	0.00015
9.18	0.70849	0.70879	0.00014
9.13	0.70904	0.70884	0.00013
9.08	0.70846	0.70883	0.00012
9.02	0.70868	0.70887	0.00009
8.97	0.70883	0.70887	0.00009
8.92	0.70860	0.70887	0.00009
8.86	0.70887	0.70890	0.00007
8.81	0.70891	0.70890	0.00007
8.75	0.70909	0.70887	0.00009
8.70	0.70888	0.70888	0.00009
8.65	0.70901	0.70883	0.00013
8.59	0.70897	0.70878	0.00014
8.54	0.70884	0.70874	0.00013
8.49	0.70867	0.70877	0.00016
8.43	0.70885	0.70876	0.00016
8.38	0.70893	0.70876	0.00016
8.32	0.70885	0.70874	0.00015
8.27	0.70865	0.70876	0.00016
8.22	0.70911	0.70877	0.00016
8.16	0.70840	0.70873	0.00014
8.11	0.70853	0.70879	0.00013
8.06	0.70859	0.70883	0.00012
8.00	0.70914	0.70884	0.00011
7.95	0.70857	0.70881	0.00009
7.89	0.70882	0.70883	0.00007
7.84	0.70879	0.70880	0.00010
7.79	0.70900	0.70877	0.00011
7.73	0.70874	0.70878	0.00012
7.68	0.70874	0.70874	0.00015
7.63	0.70898	0.70875	0.00015
7.57	0.70895	0.70873	0.00014
7.52	0.70864	0.70870	0.00014
7.47	0.70885	0.70871	0.00014
7.41	0.70880	0.70873	0.00016
7.36	0.70848	0.70876	0.00017
7.30	0.70857	0.70881	0.00016
7.25	0.70905	0.70881	0.00016
7.20	0.70830	0.70878	0.00015
7.14	0.70885	0.70883	0.00011
7.09	0.70881	0.70880	0.00012
7.04	0.70862	0.70881	0.00012
6.98	0.70874	0.70884	0.00012
6.93	0.70909	0.70887	0.00012
6.87	0.70909	0.70883	0.00011
6.82	0.70896	0.70882	0.00010
6.77	0.70857	0.70882	0.00011
6.71	0.70877	0.70884	0.00009
6.66	0.70876	0.70883	0.00010
6.61	0.70862	0.70882	0.00010

7.62	0.70895	0.70902	0.00010	6.55	0.70886	0.70883	0.00010
7.58	0.70898	0.70900	0.00010	6.50	0.70896	0.70885	0.00011
7.54	0.70882	0.70899	0.00011	6.44	0.70901	0.70884	0.00011
7.49	0.70889	0.70902	0.00011	6.39	0.70867	0.70882	0.00010
7.45	0.70931	0.70902	0.00011	6.34	0.70899	0.70882	0.00010
7.41	0.70901	0.70896	0.00011	6.28	0.70903	0.70881	0.00010
7.36	0.70911	0.70892	0.00013	6.23	0.70874	0.70880	0.00010
7.32	0.70887	0.70890	0.00013	6.18	0.70868	0.70881	0.00010
7.28	0.70919	0.70893	0.00014	6.12	0.70865	0.70883	0.00009
7.23	0.70902	0.70893	0.00014	6.07	0.70868	0.70886	0.00009
7.19	0.70882	0.70893	0.00014	6.02	0.70906	0.70886	0.00009
7.15	0.70880	0.70895	0.00014	5.96	0.70887	0.70885	0.00008
7.10	0.70921	0.70895	0.00014	5.91	0.70889	0.70885	0.00008
7.06	0.70887	0.70891	0.00013	5.85	0.70863	0.70886	0.00009
7.02	0.70873	0.70886	0.00016	5.80	0.70884	0.70888	0.00007
6.97	0.70855	0.70891	0.00017	5.75	0.70901	0.70884	0.00011
6.93	0.70893	0.70892	0.00016	5.69	0.70876	0.70884	0.00011
6.89	0.70918	0.70893	0.00016	5.64	0.70893	0.70889	0.00013
6.84	0.70917	0.70887	0.00016	5.59	0.70891	0.70887	0.00013
6.80	0.70907	0.70889	0.00018	5.53	0.70867	0.70884	0.00015
6.76	0.70903	0.70886	0.00018	5.48	0.70901	0.70885	0.00015
6.71	0.70877	0.70884	0.00017	5.42	0.70882	0.70882	0.00014
6.67	0.70877	0.70885	0.00017	5.37	0.70900	0.70884	0.00015
6.63	0.70845	0.70889	0.00018	5.32	0.70886	0.70883	0.00015
6.58	0.70915	0.70893	0.00015	5.26	0.70847	0.70880	0.00016
6.54	0.70868	0.70890	0.00014	5.21	0.70898	0.70884	0.00014
6.50	0.70899	0.70892	0.00014	5.16	0.70925	0.70880	0.00015
6.45	0.70859	0.70892	0.00014	5.10	0.70876	0.70873	0.00011
6.41	0.70938	0.70899	0.00014	5.05	0.70853	0.70871	0.00012
6.37	0.70883	0.70901	0.00015	4.99	0.70883	0.70874	0.00011
6.32	0.70880	0.70903	0.00015	4.94	0.70872	0.70869	0.00014
6.28	0.70888	0.70903	0.00015	4.89	0.70904	0.70869	0.00014
6.24	0.70915	0.70903	0.00015	4.83	0.70886	0.70869	0.00014
6.19	0.70886	0.70903	0.00015	4.78	0.70853	0.70864	0.00014
6.15	0.70886	0.70905	0.00015	4.73	0.70891	0.70869	0.00017
6.11	0.70883	0.70906	0.00014	4.67	0.70854	0.70870	0.00017
6.06	0.70906	0.70911	0.00014	4.62	0.70856	0.70873	0.00017
6.02	0.70931	0.70905	0.00019	4.56	0.70861	0.70874	0.00016
5.98	0.70952	0.70902	0.00018	4.51	0.70881	0.70878	0.00017
5.93	0.70903	0.70894	0.00016	4.46	0.70828	0.70873	0.00019
5.89	0.70883	0.70894	0.00017	4.40	0.70881	0.70877	0.00017
5.85	0.70884	0.70897	0.00016	4.35	0.70896	0.70880	0.00018
5.80	0.70922	0.70897	0.00016	4.30	0.70838	0.70876	0.00018
5.76	0.70901	0.70890	0.00017	4.24	0.70908	0.70883	0.00017
5.72	0.70897	0.70890	0.00017	4.19	0.70898	0.70881	0.00016
5.67	0.70927	0.70889	0.00017	4.14	0.70880	0.70878	0.00016
5.63	0.70845	0.70888	0.00016	4.08	0.70864	0.70879	0.00016
5.59	0.70910	0.70891	0.00013	4.03	0.70903	0.70885	0.00018
5.54	0.70862	0.70889	0.00012	3.97	0.70831	0.70879	0.00019
5.50	0.70912	0.70894	0.00012	3.92	0.70873	0.70886	0.00016

5.46	0.70907	0.70896	0.00013	3.87	0.70906	0.70887	0.00016
5.41	0.70884	0.70894	0.00014	3.81	0.70857	0.70890	0.00018
5.37	0.70855	0.70898	0.00015	3.76	0.70910	0.70893	0.00016
5.33	0.70898	0.70903	0.00011	3.71	0.70886	0.70887	0.00017
5.28	0.70891	0.70902	0.00011	3.65	0.70866	0.70890	0.00018
5.24	0.70911	0.70903	0.00011	3.60	0.70893	0.70889	0.00019
5.20	0.70883	0.70904	0.00011	3.54	0.70925	0.70889	0.00019
5.15	0.70884	0.70899	0.00017	3.49	0.70845	0.70883	0.00018
5.11	0.70913	0.70902	0.00017	3.44	0.70894	0.70888	0.00016
5.07	0.70931	0.70896	0.00019	3.38	0.70892	0.70887	0.00016
5.02	0.70919	0.70892	0.00016	3.33	0.70930	0.70885	0.00016
4.98	0.70894	0.70891	0.00015	3.28	0.70888	0.70881	0.00013
4.94	0.70892	0.70890	0.00015	3.22	0.70853	0.70882	0.00013
4.89	0.70896	0.70891	0.00016	3.17	0.70913	0.70884	0.00011
4.85	0.70920	0.70888	0.00016	3.11	0.70858	0.70881	0.00009
4.81	0.70839	0.70889	0.00016	3.06	0.70891	0.70885	0.00008
4.76	0.70910	0.70894	0.00012	3.01	0.70860	0.70885	0.00008
4.72	0.70863	0.70894	0.00012	2.95	0.70903	0.70885	0.00008
4.68	0.70892	0.70896	0.00010	2.90	0.70877	0.70884	0.00007
4.63	0.70891	0.70891	0.00015	2.85	0.70876	0.70885	0.00007
4.59	0.70912	0.70889	0.00015	2.79	0.70890	0.70883	0.00009
4.55	0.70883	0.70884	0.00015	2.74	0.70893	0.70879	0.00011
4.50	0.70903	0.70884	0.00015	2.69	0.70880	0.70879	0.00010
4.46	0.70871	0.70884	0.00015	2.63	0.70879	0.70881	0.00011
4.42	0.70925	0.70886	0.00015	2.58	0.70901	0.70882	0.00011
4.37	0.70891	0.70883	0.00013	2.52	0.70892	0.70883	0.00012
4.33	0.70905	0.70878	0.00015	2.47	0.70864	0.70884	0.00013
4.29	0.70890	0.70876	0.00014	2.42	0.70891	0.70887	0.00012
4.24	0.70842	0.70876	0.00014	2.36	0.70887	0.70889	0.00013
4.20	0.70869	0.70880	0.00012	2.31	0.70856	0.70888	0.00013
4.16	0.70864	0.70886	0.00015	2.26	0.70853	0.70891	0.00011
4.11	0.70876	0.70887	0.00014	2.20	0.70891	0.70896	0.00007
4.07	0.70906	0.70889	0.00014	2.15	0.70901	0.70896	0.00007
4.03	0.70890	0.70891	0.00017	2.09	0.70886	0.70895	0.00007
3.98	0.70900	0.70894	0.00017	2.04	0.70906	0.70897	0.00008
3.94	0.70841	0.70890	0.00018	1.99	0.70906	0.70895	0.00008
3.90	0.70883	0.70893	0.00015	1.93	0.70889	0.70894	0.00008
3.85	0.70885	0.70893	0.00016	1.88	0.70911	0.70897	0.00009
3.81	0.70884	0.70895	0.00016	1.83	0.70878	0.70895	0.00009
3.77	0.70929	0.70896	0.00016	1.77	0.70886	0.70899	0.00009
3.72	0.70874	0.70891	0.00015	1.72	0.70905	0.70903	0.00010
3.68	0.70895	0.70889	0.00016	1.66	0.70892	0.70899	0.00013
3.64	0.70932	0.70891	0.00016	1.61	0.70885	0.70898	0.00013
3.59	0.70915	0.70888	0.00014	1.56	0.70912	0.70896	0.00015
3.55	0.70860	0.70886	0.00013	1.50	0.70882	0.70891	0.00016
3.51	0.70875	0.70889	0.00012	1.45	0.70903	0.70894	0.00016
3.46	0.70876	0.70893	0.00012	1.40	0.70920	0.70891	0.00017
3.42	0.70912	0.70891	0.00014	1.34	0.70887	0.70890	0.00016
3.38	0.70895	0.70889	0.00013	1.29	0.70920	0.70897	0.00021
3.33	0.70874	0.70882	0.00017	1.24	0.70928	0.70890	0.00022

3.29	0.70858	0.70881	0.00018
3.25	0.70910	0.70885	0.00017
3.21	0.70908	0.70882	0.00017
3.16	0.70889	0.70881	0.00016
3.12	0.70894	0.70880	0.00016
3.08	0.70917	0.70880	0.00016
3.03	0.70857	0.70878	0.00014
2.99	0.70883	0.70882	0.00014
2.95	0.70831	0.70883	0.00014
2.90	0.70859	0.70888	0.00009
2.86	0.70898	0.70894	0.00008
2.82	0.70882	0.70897	0.00012
2.77	0.70898	0.70896	0.00013
2.73	0.70876	0.70898	0.00014
2.69	0.70901	0.70896	0.00016
2.64	0.70891	0.70892	0.00017
2.60	0.70905	0.70891	0.00017
2.56	0.70892	0.70894	0.00020
2.51	0.70877	0.70895	0.00020
2.47	0.70919	0.70897	0.00019
2.43	0.70933	0.70896	0.00019
2.38	0.70864	0.70893	0.00017
2.34	0.70921	0.70896	0.00016
2.30	0.70859	0.70894	0.00015
2.25	0.70860	0.70896	0.00013
2.21	0.70878	0.70901	0.00010
2.17	0.70940	0.70901	0.00010
2.12	0.70897	0.70898	0.00005
2.08	0.70899	0.70894	0.00009
2.04	0.70909	0.70890	0.00012
1.99	0.70903	0.70888	0.00011
1.95	0.70892	0.70886	0.00011
1.91	0.70899	0.70884	0.00011
1.86	0.70887	0.70887	0.00014
1.82	0.70905	0.70888	0.00014
1.78	0.70883	0.70886	0.00014
1.73	0.70905	0.70887	0.00014
1.69	0.70861	0.70886	0.00013
1.65	0.70854	0.70886	0.00013
1.60	0.70887	0.70887	0.00012
1.56	0.70890	0.70884	0.00014
1.52	0.70870	0.70889	0.00018
1.47	0.70927	0.70891	0.00017
1.43	0.70903	0.70887	0.00015
1.39	0.70881	0.70886	0.00015
1.34	0.70892	0.70891	0.00017
1.30	0.70892	0.70889	0.00017
1.26	0.70869	0.70888	0.00018
1.21	0.70861	0.70888	0.00018
1.17	0.70854	0.70888	0.00018
1.18	0.70866	0.70890	0.00022
1.13	0.70883	0.70895	0.00022
1.07	0.70856	0.70900	0.00023
1.02	0.70867	0.70905	0.00021
0.97	0.70913	0.70907	0.00020
0.91	0.70875	0.70910	0.00021
0.86	0.70910	0.70920	0.00024
0.81	0.70955	0.70918	0.00025
0.75	0.70847	0.70909	0.00025
0.70	0.70926	0.70908	0.00027
0.64	0.70916	0.70909	0.00028
0.59	0.70939	0.70908	0.00028
0.54	0.70900	0.70902	0.00027
0.48	0.70885	0.70902	0.00029
0.43	0.70945	0.70904	0.00031
0.38	0.70982	0.70899	0.00031
0.32	0.70885	0.70885	0.00023
0.27	0.70869	0.70885	0.00026
0.21	0.70831	0.70889	0.00029
0.16	0.70939	0.70908	0.00020
0.11	0.70909	0.70893	0.00015
0.05	0.70876		

1.13	0.70939	0.70891	0.00016
1.08	0.70897	0.70889	0.00014
1.04	0.70879	0.70889	0.00014
1.00	0.70898	0.70889	0.00014
0.95	0.70929	0.70885	0.00015
0.91	0.70877	0.70885	0.00015
0.87	0.70879	0.70883	0.00016
0.82	0.70867	0.70884	0.00016
0.78	0.70860	0.70888	0.00017
0.74	0.70889	0.70891	0.00015
0.69	0.70919	0.70894	0.00016
0.65	0.70894	0.70889	0.00016
0.61	0.70872	0.70887	0.00016
0.56	0.70859	0.70892	0.00017
0.52	0.70930	0.70893	0.00016
0.48	0.70857	0.70888	0.00013
0.43	0.70888	0.70891	0.00012
0.39	0.70912	0.70891	0.00012
0.35	0.70886	0.70889	0.00012
0.30	0.70920	0.70889	0.00013
0.26	0.70874	0.70884	0.00011
0.22	0.70875	0.70886	0.00012
0.17	0.70918	0.70889	0.00013
0.13	0.70875	0.70879	0.00003
0.09	0.70879	0.70882	0.00002
0.04	0.70884		

ARB 113.2.1 (M2)

Distance from cervix (mm)	$^{87}Sr/^{86}Sr$	10 point mov. average	2 SE on mov. average
38.78	0.70857	0.70857	0.00014
38.74	0.70890	0.70856	0.00014
38.69	0.70817	0.70852	0.00012
38.65	0.70865	0.70853	0.00011
38.61	0.70827	0.70852	0.00011
38.56	0.70854	0.70858	0.00011
38.52	0.70877	0.70857	0.00011
38.48	0.70850	0.70855	0.00010
38.43	0.70854	0.70856	0.00010
38.39	0.70876	0.70856	0.00010
38.35	0.70846	0.70853	0.00009
38.30	0.70857	0.70858	0.00013
38.26	0.70824	0.70856	0.00014
38.22	0.70853	0.70860	0.00012
38.17	0.70884	0.70861	0.00012
38.13	0.70848	0.70858	0.00011
38.09	0.70857	0.70860	0.00011
38.05	0.70861	0.70857	0.00012
38.00	0.70850	0.70855	0.00013
37.96	0.70852	0.70853	0.00014
37.92	0.70898	0.70851	0.00015
37.87	0.70829	0.70848	0.00011
37.83	0.70868	0.70851	0.00011
37.79	0.70867	0.70848	0.00010
37.74	0.70851	0.70847	0.00009
37.70	0.70865	0.70844	0.00010
37.66	0.70834	0.70845	0.00011
37.61	0.70841	0.70848	0.00012
37.57	0.70823	0.70850	0.00012
37.53	0.70835	0.70855	0.00011
37.48	0.70869	0.70861	0.00013
37.44	0.70853	0.70857	0.00014
37.40	0.70839	0.70858	0.00014
37.35	0.70858	0.70862	0.00014
37.31	0.70824	0.70863	0.00014
37.27	0.70874	0.70864	0.00013
37.22	0.70865	0.70863	0.00013
37.18	0.70861	0.70859	0.00014
37.14	0.70868	0.70859	0.00014
37.09	0.70899	0.70859	0.00014
37.05	0.70831	0.70855	0.00011
37.01	0.70859	0.70856	0.00010
36.96	0.70880	0.70859	0.00011
36.92	0.70873	0.70857	0.00010
36.88	0.70834	0.70855	0.00009
36.83	0.70856	0.70857	0.00008
36.79	0.70834	0.70859	0.00008

ARB 113.3.1 (M3)

Distance from cervix (mm)	$^{87}Sr/^{86}Sr$	10 point mov. average	2 SE on mov. average
44.11	0.70901	0.70865	0.00016
44.07	0.70882	0.70862	0.00013
44.02	0.70876	0.70859	0.00013
43.98	0.70852	0.70857	0.00012
43.94	0.70845	0.70860	0.00013
43.89	0.70878	0.70864	0.00013
43.85	0.70862	0.70865	0.00014
43.81	0.70839	0.70868	0.00015
43.76	0.70825	0.70866	0.00017
43.72	0.70893	0.70870	0.00015
43.68	0.70866	0.70869	0.00014
43.64	0.70853	0.70869	0.00014
43.59	0.70860	0.70870	0.00014
43.55	0.70879	0.70871	0.00014
43.51	0.70882	0.70872	0.00014
43.46	0.70890	0.70870	0.00014
43.42	0.70891	0.70868	0.00013
43.38	0.70817	0.70867	0.00012
43.33	0.70869	0.70868	0.00010
43.29	0.70882	0.70865	0.00012
43.25	0.70872	0.70864	0.00012
43.20	0.70859	0.70866	0.00013
43.16	0.70866	0.70866	0.00013
43.12	0.70889	0.70862	0.00016
43.07	0.70861	0.70858	0.00015
43.03	0.70878	0.70857	0.00015
42.99	0.70872	0.70859	0.00017
42.94	0.70833	0.70859	0.00017
42.90	0.70834	0.70864	0.00016
42.86	0.70878	0.70864	0.00016
42.82	0.70893	0.70862	0.00016
42.77	0.70861	0.70859	0.00014
42.73	0.70822	0.70859	0.00014
42.69	0.70846	0.70862	0.00012
42.64	0.70853	0.70863	0.00012
42.60	0.70902	0.70865	0.00011
42.56	0.70873	0.70864	0.00011
42.51	0.70879	0.70864	0.00011
42.47	0.70834	0.70861	0.00011
42.43	0.70853	0.70861	0.00011
42.38	0.70863	0.70863	0.00011
42.34	0.70859	0.70866	0.00013
42.30	0.70852	0.70862	0.00016
42.25	0.70862	0.70861	0.00016
42.21	0.70871	0.70861	0.00016
42.17	0.70896	0.70862	0.00016
42.12	0.70875	0.70860	0.00015

36.75	0.70859	0.70860	0.00007	42.08	0.70845	0.70862	0.00016
36.70	0.70864	0.70858	0.00008	42.04	0.70833	0.70863	0.00016
36.66	0.70863	0.70858	0.00008	42.00	0.70874	0.70864	0.00015
36.62	0.70843	0.70854	0.00010	41.95	0.70892	0.70863	0.00015
36.58	0.70880	0.70859	0.00012	41.91	0.70816	0.70862	0.00014
36.53	0.70861	0.70859	0.00012	41.87	0.70850	0.70868	0.00010
36.49	0.70857	0.70857	0.00013	41.82	0.70859	0.70867	0.00010
36.45	0.70856	0.70857	0.00013	41.78	0.70879	0.70868	0.00010
36.40	0.70868	0.70857	0.00013	41.74	0.70879	0.70863	0.00013
36.36	0.70843	0.70854	0.00013	41.69	0.70894	0.70860	0.00012
36.32	0.70842	0.70856	0.00013	41.65	0.70856	0.70857	0.00010
36.27	0.70865	0.70858	0.00013	41.61	0.70844	0.70855	0.00011
36.23	0.70826	0.70856	0.00013	41.56	0.70864	0.70855	0.00011
36.19	0.70890	0.70857	0.00012	41.52	0.70880	0.70852	0.00011
36.14	0.70882	0.70852	0.00010	41.48	0.70871	0.70846	0.00011
36.10	0.70837	0.70851	0.00008	41.43	0.70844	0.70849	0.00013
36.06	0.70858	0.70851	0.00008	41.39	0.70869	0.70851	0.00014
36.01	0.70858	0.70854	0.00009	41.35	0.70828	0.70847	0.00015
35.97	0.70837	0.70855	0.00010	41.30	0.70850	0.70850	0.00014
35.93	0.70871	0.70853	0.00012	41.26	0.70864	0.70854	0.00016
35.88	0.70852	0.70852	0.00012	41.22	0.70837	0.70855	0.00017
35.84	0.70854	0.70851	0.00012	41.18	0.70846	0.70856	0.00016
35.80	0.70837	0.70854	0.00014	41.13	0.70832	0.70857	0.00016
35.75	0.70840	0.70856	0.00013	41.09	0.70825	0.70862	0.00016
35.71	0.70866	0.70862	0.00015	41.05	0.70893	0.70866	0.00014
35.67	0.70842	0.70857	0.00016	41.00	0.70871	0.70859	0.00014
35.62	0.70880	0.70862	0.00017	40.96	0.70821	0.70862	0.00015
35.58	0.70870	0.70861	0.00017	40.92	0.70862	0.70865	0.00013
35.54	0.70817	0.70858	0.00017	40.87	0.70885	0.70867	0.00013
35.49	0.70860	0.70860	0.00016	40.83	0.70877	0.70865	0.00012
35.45	0.70843	0.70860	0.00016	40.79	0.70844	0.70867	0.00013
35.41	0.70886	0.70862	0.00015	40.74	0.70854	0.70868	0.00013
35.36	0.70859	0.70857	0.00014	40.70	0.70884	0.70868	0.00013
35.32	0.70893	0.70857	0.00015	40.66	0.70863	0.70866	0.00012
35.28	0.70822	0.70855	0.00013	40.61	0.70830	0.70865	0.00013
35.23	0.70889	0.70857	0.00011	40.57	0.70894	0.70871	0.00010
35.19	0.70873	0.70854	0.00008	40.53	0.70859	0.70865	0.00011
35.15	0.70837	0.70855	0.00009	40.48	0.70881	0.70866	0.00011
35.11	0.70840	0.70857	0.00008	40.44	0.70867	0.70864	0.00011
35.06	0.70860	0.70858	0.00008	40.40	0.70894	0.70867	0.00014
35.02	0.70857	0.70858	0.00008	40.36	0.70851	0.70863	0.00012
34.98	0.70840	0.70857	0.00008	40.31	0.70855	0.70868	0.00014
34.93	0.70865	0.70859	0.00008	40.27	0.70862	0.70868	0.00015
34.89	0.70866	0.70856	0.00009	40.23	0.70857	0.70871	0.00015
34.85	0.70845	0.70854	0.00008	40.18	0.70886	0.70873	0.00015
34.80	0.70853	0.70858	0.00010	40.14	0.70835	0.70871	0.00015
34.76	0.70883	0.70856	0.00011	40.10	0.70876	0.70874	0.00013
34.72	0.70864	0.70855	0.00010	40.05	0.70852	0.70872	0.00013
34.67	0.70847	0.70855	0.00011	40.01	0.70902	0.70874	0.00013
34.63	0.70862	0.70855	0.00011	39.97	0.70856	0.70869	0.00011

34.59	0.70843	0.70851	0.00012
34.54	0.70860	0.70854	0.00013
34.50	0.70837	0.70851	0.00013
34.46	0.70849	0.70850	0.00014
34.41	0.70886	0.70848	0.00015
34.37	0.70833	0.70842	0.00013
34.33	0.70866	0.70842	0.00013
34.28	0.70871	0.70837	0.00012
34.24	0.70840	0.70838	0.00012
34.20	0.70822	0.70837	0.00013
34.15	0.70872	0.70840	0.00013
34.11	0.70838	0.70839	0.00011
34.07	0.70820	0.70842	0.00013
34.02	0.70831	0.70847	0.00013
33.98	0.70827	0.70848	0.00013
33.94	0.70828	0.70848	0.00013
33.89	0.70826	0.70850	0.00012
33.85	0.70874	0.70853	0.00011
33.81	0.70829	0.70852	0.00010
33.76	0.70855	0.70855	0.00009
33.72	0.70861	0.70856	0.00009
33.68	0.70868	0.70854	0.00009
33.64	0.70872	0.70848	0.00012
33.59	0.70838	0.70844	0.00011
33.55	0.70827	0.70842	0.00013
33.51	0.70849	0.70842	0.00013
33.46	0.70853	0.70844	0.00015
33.42	0.70866	0.70844	0.00014
33.38	0.70862	0.70842	0.00014
33.33	0.70859	0.70840	0.00013
33.29	0.70843	0.70837	0.00012
33.25	0.70808	0.70836	0.00012
33.20	0.70836	0.70838	0.00011
33.16	0.70816	0.70841	0.00011
33.12	0.70825	0.70842	0.00010
33.07	0.70876	0.70841	0.00011
33.03	0.70853	0.70842	0.00012
32.99	0.70845	0.70844	0.00014
32.94	0.70837	0.70846	0.00015
32.90	0.70834	0.70846	0.00015
32.86	0.70834	0.70846	0.00015
32.81	0.70827	0.70848	0.00015
32.77	0.70858	0.70852	0.00014
32.73	0.70834	0.70851	0.00014
32.68	0.70813	0.70854	0.00014
32.64	0.70881	0.70859	0.00011
32.60	0.70880	0.70857	0.00010
32.55	0.70865	0.70852	0.00009
32.51	0.70833	0.70850	0.00009
32.47	0.70838	0.70859	0.00016

39.92	0.70902	0.70866	0.00014
39.88	0.70850	0.70866	0.00014
39.84	0.70891	0.70865	0.00015
39.79	0.70882	0.70861	0.00014
39.75	0.70869	0.70859	0.00013
39.71	0.70862	0.70856	0.00013
39.66	0.70853	0.70857	0.00014
39.62	0.70870	0.70859	0.00014
39.58	0.70856	0.70854	0.00015
39.53	0.70824	0.70855	0.00015
39.49	0.70902	0.70858	0.00014
39.45	0.70839	0.70855	0.00010
39.41	0.70854	0.70857	0.00010
39.36	0.70859	0.70859	0.00010
39.32	0.70842	0.70863	0.00013
39.28	0.70876	0.70866	0.00013
39.23	0.70867	0.70862	0.00014
39.19	0.70823	0.70866	0.00017
39.15	0.70864	0.70867	0.00015
39.10	0.70859	0.70867	0.00015
39.06	0.70872	0.70867	0.00015
39.02	0.70859	0.70862	0.00018
38.97	0.70868	0.70862	0.00018
38.93	0.70904	0.70861	0.00018
38.89	0.70870	0.70855	0.00015
38.84	0.70833	0.70853	0.00015
38.80	0.70907	0.70852	0.00015
38.76	0.70833	0.70847	0.00010
38.71	0.70863	0.70851	0.00010
38.67	0.70862	0.70854	0.00013
38.63	0.70823	0.70857	0.00015
38.59	0.70855	0.70861	0.00013
38.54	0.70857	0.70864	0.00014
38.50	0.70845	0.70863	0.00015
38.46	0.70849	0.70866	0.00014
38.41	0.70823	0.70865	0.00015
38.37	0.70862	0.70869	0.00012
38.33	0.70873	0.70871	0.00012
38.28	0.70890	0.70871	0.00012
38.24	0.70893	0.70868	0.00012
38.20	0.70860	0.70863	0.00011
38.15	0.70889	0.70864	0.00011
38.11	0.70845	0.70864	0.00011
38.07	0.70873	0.70864	0.00011
38.02	0.70836	0.70864	0.00011
37.98	0.70870	0.70868	0.00009
37.94	0.70878	0.70867	0.00009
37.89	0.70878	0.70860	0.00014
37.85	0.70853	0.70860	0.00014
37.81	0.70850	0.70863	0.00015

32.42	0.70854	0.70860	0.00015	37.77	0.70868	0.70864	0.00014
32.38	0.70869	0.70859	0.00016	37.72	0.70886	0.70865	0.00015
32.34	0.70842	0.70862	0.00018	37.68	0.70845	0.70866	0.00015
32.30	0.70871	0.70863	0.00018	37.64	0.70878	0.70873	0.00017
32.25	0.70863	0.70861	0.00018	37.59	0.70875	0.70869	0.00018
32.21	0.70838	0.70862	0.00017	37.55	0.70859	0.70868	0.00018
32.17	0.70843	0.70865	0.00016	37.51	0.70810	0.70872	0.00019
32.12	0.70916	0.70866	0.00016	37.46	0.70872	0.70879	0.00013
32.08	0.70843	0.70861	0.00012	37.42	0.70884	0.70880	0.00013
32.04	0.70844	0.70860	0.00013	37.38	0.70862	0.70880	0.00013
31.99	0.70900	0.70863	0.00012	37.33	0.70881	0.70879	0.00013
31.95	0.70848	0.70856	0.00010	37.29	0.70892	0.70878	0.00013
31.91	0.70855	0.70857	0.00010	37.25	0.70917	0.70878	0.00014
31.86	0.70878	0.70857	0.00010	37.20	0.70842	0.70875	0.00011
31.82	0.70858	0.70855	0.00009	37.16	0.70865	0.70878	0.00008
31.78	0.70871	0.70856	0.00009	37.12	0.70896	0.70879	0.00007
31.73	0.70846	0.70851	0.00011	37.07	0.70881	0.70872	0.00011
31.69	0.70872	0.70855	0.00014	37.03	0.70878	0.70871	0.00011
31.65	0.70832	0.70855	0.00013	36.99	0.70883	0.70870	0.00011
31.60	0.70869	0.70854	0.00014	36.95	0.70858	0.70872	0.00012
31.56	0.70832	0.70852	0.00013	36.90	0.70868	0.70875	0.00012
31.52	0.70862	0.70855	0.00013	36.86	0.70895	0.70876	0.00012
31.47	0.70852	0.70853	0.00013	36.82	0.70883	0.70878	0.00014
31.43	0.70859	0.70849	0.00015	36.77	0.70868	0.70876	0.00015
31.39	0.70863	0.70848	0.00015	36.73	0.70877	0.70873	0.00016
31.34	0.70820	0.70846	0.00015	36.69	0.70831	0.70873	0.00016
31.30	0.70890	0.70850	0.00013	36.64	0.70864	0.70875	0.00014
31.26	0.70868	0.70846	0.00010	36.60	0.70874	0.70877	0.00014
31.21	0.70829	0.70843	0.00009	36.56	0.70897	0.70878	0.00014
31.17	0.70843	0.70849	0.00013	36.51	0.70892	0.70872	0.00015
31.13	0.70861	0.70852	0.00014	36.47	0.70878	0.70871	0.00014
31.08	0.70848	0.70852	0.00014	36.43	0.70915	0.70869	0.00014
31.04	0.70811	0.70855	0.00014	36.38	0.70859	0.70867	0.00011
31.00	0.70850	0.70861	0.00011	36.34	0.70845	0.70865	0.00012
30.95	0.70844	0.70861	0.00011	36.30	0.70876	0.70874	0.00017
30.91	0.70854	0.70862	0.00011	36.25	0.70849	0.70872	0.00018
30.87	0.70851	0.70861	0.00011	36.21	0.70887	0.70879	0.00019
30.83	0.70837	0.70860	0.00012	36.17	0.70881	0.70878	0.00019
30.78	0.70894	0.70862	0.00011	36.13	0.70841	0.70878	0.00019
30.74	0.70872	0.70858	0.00008	36.08	0.70877	0.70880	0.00017
30.70	0.70859	0.70858	0.00008	36.04	0.70866	0.70877	0.00019
30.65	0.70876	0.70856	0.00009	36.00	0.70892	0.70876	0.00019
30.61	0.70875	0.70853	0.00008	35.95	0.70842	0.70878	0.00020
30.57	0.70847	0.70853	0.00008	35.91	0.70931	0.70878	0.00021
30.52	0.70860	0.70851	0.00008	35.87	0.70857	0.70868	0.00018
30.48	0.70840	0.70852	0.00009	35.82	0.70917	0.70869	0.00018
30.44	0.70843	0.70854	0.00009	35.78	0.70880	0.70868	0.00017
30.39	0.70850	0.70852	0.00010	35.74	0.70874	0.70871	0.00019
30.35	0.70860	0.70851	0.00011	35.69	0.70866	0.70869	0.00020
30.31	0.70874	0.70847	0.00012	35.65	0.70846	0.70869	0.00020

30.26	0.70838	0.70845	0.00010	35.61	0.70855	0.70875	0.00021
30.22	0.70846	0.70847	0.00010	35.56	0.70914	0.70876	0.00020
30.18	0.70868	0.70849	0.00011	35.52	0.70836	0.70871	0.00019
30.13	0.70835	0.70850	0.00011	35.48	0.70834	0.70876	0.00017
30.09	0.70871	0.70853	0.00011	35.43	0.70869	0.70879	0.00015
30.05	0.70857	0.70852	0.00011	35.39	0.70905	0.70884	0.00017
30.00	0.70826	0.70851	0.00011	35.35	0.70915	0.70879	0.00016
29.96	0.70831	0.70852	0.00011	35.31	0.70848	0.70874	0.00014
29.92	0.70828	0.70857	0.00011	35.26	0.70864	0.70878	0.00013
29.87	0.70853	0.70858	0.00010	35.22	0.70908	0.70881	0.00014
29.83	0.70858	0.70858	0.00010	35.18	0.70863	0.70881	0.00014
29.79	0.70867	0.70859	0.00010	35.13	0.70865	0.70880	0.00015
29.74	0.70871	0.70855	0.00011	35.09	0.70892	0.70880	0.00015
29.70	0.70869	0.70853	0.00011	35.05	0.70859	0.70875	0.00016
29.66	0.70865	0.70856	0.00014	35.00	0.70916	0.70869	0.00021
29.61	0.70848	0.70854	0.00014	34.96	0.70860	0.70862	0.00019
29.57	0.70832	0.70853	0.00015	34.92	0.70865	0.70864	0.00020
29.53	0.70885	0.70855	0.00014	34.87	0.70883	0.70867	0.00021
29.48	0.70838	0.70853	0.00013	34.83	0.70902	0.70864	0.00020
29.44	0.70848	0.70855	0.00012	34.79	0.70908	0.70860	0.00019
29.40	0.70865	0.70852	0.00015	34.74	0.70849	0.70856	0.00016
29.36	0.70833	0.70853	0.00015	34.70	0.70862	0.70859	0.00016
29.31	0.70845	0.70852	0.00015	34.66	0.70845	0.70860	0.00016
29.27	0.70900	0.70853	0.00015	34.61	0.70804	0.70865	0.00018
29.23	0.70846	0.70849	0.00011	34.57	0.70841	0.70873	0.00011
29.18	0.70836	0.70845	0.00015	34.53	0.70879	0.70876	0.00009
29.14	0.70852	0.70848	0.00016	34.49	0.70896	0.70876	0.00009
29.10	0.70871	0.70849	0.00016	34.44	0.70859	0.70869	0.00013
29.05	0.70851	0.70848	0.00016	34.40	0.70858	0.70872	0.00013
29.01	0.70817	0.70850	0.00016	34.36	0.70870	0.70875	0.00013
28.97	0.70875	0.70855	0.00015	34.31	0.70880	0.70871	0.00016
28.92	0.70832	0.70851	0.00014	34.27	0.70863	0.70870	0.00016
28.88	0.70848	0.70851	0.00014	34.23	0.70900	0.70871	0.00016
28.84	0.70865	0.70850	0.00015	34.18	0.70878	0.70871	0.00016
28.79	0.70800	0.70849	0.00015	34.14	0.70872	0.70867	0.00017
28.75	0.70867	0.70854	0.00010	34.10	0.70886	0.70864	0.00018
28.71	0.70867	0.70852	0.00010	34.05	0.70823	0.70859	0.00018
28.66	0.70860	0.70849	0.00009	34.01	0.70887	0.70859	0.00017
28.62	0.70868	0.70847	0.00009	33.97	0.70891	0.70854	0.00017
28.58	0.70868	0.70847	0.00008	33.92	0.70829	0.70851	0.00015
28.53	0.70836	0.70846	0.00008	33.88	0.70874	0.70850	0.00017
28.49	0.70832	0.70845	0.00008	33.84	0.70872	0.70851	0.00018
28.45	0.70833	0.70850	0.00010	33.79	0.70898	0.70849	0.00017
28.40	0.70862	0.70851	0.00009	33.75	0.70841	0.70848	0.00017
28.36	0.70847	0.70849	0.00009	33.71	0.70841	0.70847	0.00017
28.32	0.70844	0.70847	0.00010	33.67	0.70836	0.70847	0.00017
28.27	0.70842	0.70849	0.00010	33.62	0.70826	0.70850	0.00018
28.23	0.70841	0.70848	0.00011	33.58	0.70835	0.70853	0.00017
28.19	0.70864	0.70849	0.00011	33.54	0.70862	0.70856	0.00017
28.14	0.70861	0.70846	0.00010	33.49	0.70810	0.70856	0.00017

28.10	0.70830	0.70843	0.00010	33.45	0.70890	0.70860	0.00013
28.06	0.70878	0.70843	0.00010	33.41	0.70847	0.70859	0.00012
28.01	0.70840	0.70841	0.00008	33.36	0.70894	0.70858	0.00012
27.97	0.70841	0.70843	0.00009	33.32	0.70825	0.70854	0.00009
27.93	0.70828	0.70844	0.00009	33.28	0.70849	0.70862	0.00012
27.89	0.70863	0.70848	0.00010	33.23	0.70865	0.70866	0.00012
27.84	0.70832	0.70847	0.00009	33.19	0.70849	0.70866	0.00012
27.80	0.70850	0.70849	0.00008	33.15	0.70870	0.70867	0.00012
27.76	0.70839	0.70847	0.00010	33.10	0.70857	0.70868	0.00012
27.71	0.70830	0.70848	0.00010	33.06	0.70857	0.70868	0.00012
27.67	0.70829	0.70846	0.00012	33.02	0.70874	0.70868	0.00013
27.63	0.70857	0.70849	0.00012	32.97	0.70842	0.70868	0.00013
27.58	0.70861	0.70845	0.00014	32.93	0.70852	0.70870	0.00011
27.54	0.70851	0.70840	0.00015	32.89	0.70905	0.70871	0.00011
27.50	0.70871	0.70844	0.00018	32.85	0.70889	0.70870	0.00008
27.45	0.70849	0.70844	0.00018	32.80	0.70866	0.70866	0.00008
27.37	0.70855	0.70846	0.00019	32.76	0.70853	0.70864	0.00008
27.32	0.70824	0.70844	0.00019	32.72	0.70883	0.70867	0.00009
27.28	0.70854	0.70849	0.00019	32.67	0.70856	0.70866	0.00008
27.24	0.70806	0.70849	0.00019	32.63	0.70856	0.70867	0.00008
27.19	0.70863	0.70854	0.00016	32.59	0.70875	0.70865	0.00010
27.15	0.70812	0.70854	0.00016	32.54	0.70863	0.70862	0.00010
27.11	0.70815	0.70860	0.00013	32.50	0.70870	0.70861	0.00011
27.06	0.70892	0.70868	0.00011	32.46	0.70887	0.70861	0.00011
27.02	0.70871	0.70863	0.00010	32.41	0.70850	0.70860	0.00011
26.98	0.70870	0.70862	0.00010	32.37	0.70853	0.70858	0.00012
26.93	0.70838	0.70857	0.00012	32.33	0.70882	0.70861	0.00013
26.89	0.70867	0.70861	0.00012	32.28	0.70865	0.70862	0.00014
26.85	0.70857	0.70860	0.00012	32.24	0.70875	0.70863	0.00014
26.80	0.70860	0.70856	0.00015	32.20	0.70836	0.70865	0.00015
26.76	0.70858	0.70857	0.00015	32.15	0.70846	0.70863	0.00017
26.72	0.70872	0.70854	0.00016	32.11	0.70844	0.70861	0.00019
26.67	0.70895	0.70854	0.00016	32.07	0.70871	0.70866	0.00020
26.63	0.70841	0.70849	0.00013	32.03	0.70884	0.70862	0.00021
26.59	0.70859	0.70849	0.00013	31.98	0.70829	0.70862	0.00021
26.55	0.70824	0.70847	0.00014	31.94	0.70884	0.70867	0.00020
26.50	0.70872	0.70852	0.00014	31.90	0.70890	0.70866	0.00020
26.46	0.70863	0.70850	0.00014	31.85	0.70876	0.70863	0.00019
26.42	0.70816	0.70846	0.00014	31.81	0.70891	0.70863	0.00019
26.37	0.70872	0.70853	0.00014	31.77	0.70815	0.70857	0.00019
26.33	0.70829	0.70854	0.00015	31.72	0.70821	0.70861	0.00016
26.29	0.70871	0.70856	0.00013	31.68	0.70897	0.70862	0.00016
26.24	0.70846	0.70854	0.00013	31.64	0.70831	0.70856	0.00014
26.20	0.70843	0.70856	0.00013	31.59	0.70890	0.70860	0.00014
26.16	0.70831	0.70853	0.00016	31.55	0.70876	0.70853	0.00014
26.11	0.70878	0.70857	0.00015	31.51	0.70872	0.70854	0.00015
26.07	0.70829	0.70853	0.00014	31.46	0.70859	0.70855	0.00016
26.03	0.70877	0.70857	0.00013	31.42	0.70874	0.70853	0.00016
25.98	0.70884	0.70854	0.00012	31.38	0.70831	0.70851	0.00015
25.94	0.70846	0.70853	0.00011	31.33	0.70862	0.70856	0.00015

25.90	0.70851	0.70852	0.00012
25.85	0.70867	0.70851	0.00012
25.81	0.70813	0.70852	0.00012
25.77	0.70866	0.70857	0.00008
25.72	0.70842	0.70856	0.00008
25.68	0.70851	0.70855	0.00009
25.64	0.70870	0.70854	0.00009
25.59	0.70849	0.70850	0.00010
25.55	0.70872	0.70849	0.00010
25.51	0.70836	0.70848	0.00009
25.46	0.70847	0.70850	0.00009
25.42	0.70873	0.70853	0.00010
25.38	0.70862	0.70848	0.00010
25.33	0.70857	0.70845	0.00011
25.25	0.70831	0.70848	0.00013
25.20	0.70846	0.70848	0.00013
25.16	0.70827	0.70850	0.00013
25.12	0.70840	0.70853	0.00012
25.08	0.70856	0.70851	0.00014
25.03	0.70863	0.70852	0.00014
24.99	0.70873	0.70850	0.00014
24.95	0.70826	0.70851	0.00014
24.90	0.70829	0.70852	0.00013
24.86	0.70885	0.70855	0.00012
24.82	0.70835	0.70853	0.00011
24.77	0.70861	0.70852	0.00011
24.73	0.70865	0.70850	0.00011
24.69	0.70820	0.70846	0.00011
24.64	0.70860	0.70851	0.00010
24.60	0.70846	0.70851	0.00010
24.56	0.70878	0.70853	0.00011
24.51	0.70842	0.70852	0.00010
24.47	0.70859	0.70852	0.00010
24.43	0.70862	0.70854	0.00012
24.38	0.70832	0.70853	0.00012
24.34	0.70836	0.70854	0.00011
24.30	0.70829	0.70859	0.00012
24.25	0.70863	0.70864	0.00010
24.21	0.70862	0.70863	0.00011
24.17	0.70868	0.70864	0.00011
24.12	0.70870	0.70862	0.00011
24.08	0.70837	0.70860	0.00011
24.04	0.70885	0.70863	0.00009
23.99	0.70850	0.70860	0.00008
23.95	0.70844	0.70861	0.00008
23.91	0.70883	0.70858	0.00011
23.86	0.70879	0.70855	0.00010
23.82	0.70854	0.70854	0.00008
23.78	0.70866	0.70854	0.00008
23.73	0.70855	0.70854	0.00008

31.29	0.70825	0.70856	0.00015
31.25	0.70837	0.70861	0.00014
31.21	0.70872	0.70862	0.00014
31.16	0.70822	0.70857	0.00016
31.12	0.70888	0.70858	0.00015
31.08	0.70884	0.70857	0.00014
31.03	0.70841	0.70853	0.00013
30.99	0.70850	0.70860	0.00017
30.95	0.70879	0.70867	0.00021
30.90	0.70861	0.70862	0.00021
30.86	0.70877	0.70864	0.00022
30.82	0.70844	0.70861	0.00022
30.77	0.70822	0.70862	0.00021
30.73	0.70835	0.70867	0.00020
30.69	0.70879	0.70865	0.00022
30.64	0.70838	0.70866	0.00022
30.60	0.70914	0.70865	0.00023
30.56	0.70917	0.70863	0.00021
30.51	0.70833	0.70855	0.00018
30.47	0.70880	0.70856	0.00018
30.43	0.70848	0.70853	0.00017
30.38	0.70850	0.70854	0.00017
30.34	0.70876	0.70854	0.00017
30.30	0.70810	0.70852	0.00016
30.26	0.70889	0.70859	0.00014
30.21	0.70833	0.70860	0.00016
30.17	0.70895	0.70863	0.00015
30.13	0.70839	0.70859	0.00013
30.08	0.70838	0.70863	0.00012
30.04	0.70848	0.70863	0.00012
30.00	0.70864	0.70861	0.00014
29.95	0.70849	0.70863	0.00015
29.91	0.70852	0.70862	0.00016
29.87	0.70884	0.70858	0.00019
29.82	0.70902	0.70854	0.00018
29.78	0.70856	0.70849	0.00015
29.74	0.70859	0.70848	0.00015
29.69	0.70880	0.70851	0.00017
29.65	0.70841	0.70853	0.00019
29.61	0.70824	0.70857	0.00020
29.56	0.70889	0.70864	0.00019
29.52	0.70836	0.70868	0.00023
29.48	0.70815	0.70875	0.00023
29.44	0.70835	0.70876	0.00022
29.39	0.70855	0.70880	0.00020
29.35	0.70847	0.70887	0.00021
29.31	0.70886	0.70887	0.00020
29.26	0.70906	0.70881	0.00024
29.22	0.70879	0.70877	0.00023
29.18	0.70888	0.70880	0.00024

23.69	0.70850	0.70852	0.00009
23.65	0.70863	0.70850	0.00010
23.61	0.70859	0.70849	0.00009
23.56	0.70852	0.70847	0.00009
23.52	0.70819	0.70848	0.00009
23.48	0.70855	0.70853	0.00007
23.43	0.70863	0.70853	0.00007
23.39	0.70858	0.70848	0.00010
23.35	0.70861	0.70847	0.00009
23.30	0.70841	0.70848	0.00010
23.26	0.70830	0.70854	0.00014
23.22	0.70849	0.70853	0.00014
23.17	0.70842	0.70852	0.00015
23.13	0.70863	0.70852	0.00015
23.09	0.70867	0.70849	0.00015
23.04	0.70852	0.70849	0.00015
23.00	0.70819	0.70851	0.00015
22.96	0.70844	0.70853	0.00014
22.91	0.70868	0.70852	0.00014
22.87	0.70899	0.70849	0.00014
22.83	0.70830	0.70849	0.00014
22.78	0.70832	0.70849	0.00014
22.74	0.70841	0.70851	0.00013
22.70	0.70842	0.70851	0.00013
22.65	0.70866	0.70853	0.00013
22.61	0.70870	0.70850	0.00013
22.57	0.70841	0.70850	0.00013
22.52	0.70837	0.70850	0.00013
22.48	0.70835	0.70849	0.00014
22.44	0.70897	0.70854	0.00016
22.39	0.70831	0.70850	0.00013
22.35	0.70852	0.70851	0.00012
22.31	0.70836	0.70853	0.00012
22.26	0.70864	0.70853	0.00012
22.22	0.70836	0.70850	0.00012
22.18	0.70871	0.70850	0.00013
22.14	0.70845	0.70847	0.00012
22.09	0.70824	0.70849	0.00013
22.05	0.70886	0.70852	0.00011
22.01	0.70859	0.70846	0.00009
21.96	0.70839	0.70844	0.00009
21.92	0.70867	0.70847	0.00010
21.88	0.70836	0.70844	0.00009
21.83	0.70843	0.70845	0.00009
21.79	0.70828	0.70844	0.00009
21.75	0.70844	0.70847	0.00008
21.70	0.70867	0.70849	0.00009
21.66	0.70847	0.70848	0.00008
21.62	0.70830	0.70849	0.00008
21.57	0.70833	0.70853	0.00008

29.13	0.70933	0.70879	0.00024
29.09	0.70904	0.70873	0.00021
29.05	0.70825	0.70865	0.00021
29.00	0.70877	0.70867	0.00020
28.96	0.70921	0.70862	0.00021
28.92	0.70856	0.70854	0.00017
28.87	0.70824	0.70854	0.00017
28.83	0.70867	0.70859	0.00016
28.79	0.70909	0.70860	0.00016
28.74	0.70876	0.70858	0.00014
28.70	0.70869	0.70861	0.00017
28.66	0.70826	0.70856	0.00018
28.62	0.70843	0.70864	0.00019
28.57	0.70830	0.70869	0.00020
28.53	0.70840	0.70873	0.00018
28.49	0.70860	0.70876	0.00016
28.44	0.70868	0.70878	0.00016
28.40	0.70877	0.70878	0.00016
28.36	0.70887	0.70879	0.00016
28.31	0.70910	0.70877	0.00016
28.27	0.70822	0.70869	0.00016
28.23	0.70905	0.70872	0.00013
28.18	0.70894	0.70869	0.00011
28.14	0.70866	0.70869	0.00012
28.10	0.70876	0.70874	0.00014
28.05	0.70873	0.70871	0.00016
28.01	0.70867	0.70871	0.00016
27.97	0.70892	0.70871	0.00016
27.92	0.70863	0.70865	0.00017
27.88	0.70832	0.70867	0.00017
27.84	0.70852	0.70872	0.00016
27.80	0.70879	0.70873	0.00015
27.75	0.70895	0.70871	0.00015
27.71	0.70913	0.70871	0.00015
27.67	0.70844	0.70870	0.00014
27.62	0.70879	0.70872	0.00013
27.58	0.70861	0.70872	0.00013
27.54	0.70832	0.70873	0.00012
27.49	0.70889	0.70877	0.00009
27.45	0.70877	0.70876	0.00008
27.41	0.70864	0.70874	0.00009
27.36	0.70859	0.70877	0.00009
27.32	0.70892	0.70872	0.00016
27.28	0.70901	0.70868	0.00015
27.23	0.70866	0.70868	0.00015
27.19	0.70880	0.70871	0.00016
27.15	0.70866	0.70868	0.00017
27.10	0.70874	0.70870	0.00017
27.06	0.70877	0.70868	0.00017
27.02	0.70864	0.70868	0.00017

21.53	0.70869	0.70854	0.00007	26.98	0.70886	0.70867	0.00017
21.49	0.70845	0.70852	0.00007	26.93	0.70810	0.70867	0.00017
21.44	0.70839	0.70851	0.00008	26.89	0.70861	0.70873	0.00011
21.40	0.70839	0.70853	0.00008	26.85	0.70895	0.70871	0.00013
21.36	0.70855	0.70852	0.00008	26.80	0.70900	0.70867	0.00012
21.31	0.70861	0.70852	0.00008	26.76	0.70845	0.70863	0.00010
21.27	0.70862	0.70850	0.00008	26.72	0.70885	0.70861	0.00011
21.23	0.70856	0.70848	0.00008	26.67	0.70860	0.70860	0.00010
21.18	0.70870	0.70847	0.00007	26.63	0.70872	0.70858	0.00010
21.14	0.70847	0.70845	0.00006	26.59	0.70858	0.70857	0.00010
21.10	0.70849	0.70841	0.00010	26.54	0.70886	0.70860	0.00012
21.05	0.70829	0.70843	0.00012	26.50	0.70869	0.70859	0.00011
21.01	0.70859	0.70846	0.00012	26.46	0.70839	0.70860	0.00012
20.97	0.70835	0.70844	0.00012	26.41	0.70859	0.70860	0.00012
20.92	0.70852	0.70843	0.00013	26.37	0.70855	0.70860	0.00012
20.88	0.70846	0.70842	0.00013	26.33	0.70831	0.70862	0.00012
20.84	0.70839	0.70841	0.00013	26.28	0.70868	0.70865	0.00010
20.80	0.70846	0.70842	0.00013	26.24	0.70846	0.70867	0.00011
20.75	0.70853	0.70844	0.00013	26.20	0.70857	0.70870	0.00010
20.71	0.70802	0.70841	0.00014	26.16	0.70888	0.70868	0.00011
20.67	0.70874	0.70842	0.00012	26.11	0.70878	0.70870	0.00014
20.62	0.70858	0.70841	0.00010	26.07	0.70881	0.70869	0.00013
20.58	0.70837	0.70838	0.00010	26.03	0.70841	0.70870	0.00014
20.54	0.70819	0.70838	0.00010	25.98	0.70860	0.70872	0.00012
20.49	0.70841	0.70844	0.00012	25.94	0.70871	0.70870	0.00015
20.45	0.70837	0.70842	0.00012	25.90	0.70857	0.70874	0.00017
20.41	0.70853	0.70844	0.00012	25.85	0.70886	0.70876	0.00017
20.36	0.70860	0.70847	0.00015	25.81	0.70875	0.70872	0.00018
20.32	0.70826	0.70849	0.00016	25.77	0.70839	0.70868	0.00019
20.28	0.70819	0.70852	0.00015	25.72	0.70910	0.70868	0.00019
20.23	0.70860	0.70855	0.00013	25.68	0.70875	0.70868	0.00019
20.19	0.70824	0.70852	0.00014	25.64	0.70888	0.70864	0.00020
20.15	0.70837	0.70855	0.00013	25.59	0.70863	0.70862	0.00019
20.10	0.70878	0.70859	0.00013	25.55	0.70833	0.70859	0.00019
20.06	0.70828	0.70859	0.00013	25.51	0.70915	0.70861	0.00019
20.02	0.70852	0.70862	0.00011	25.46	0.70878	0.70855	0.00014
19.97	0.70885	0.70868	0.00015	25.42	0.70845	0.70850	0.00014
19.93	0.70877	0.70862	0.00017	25.38	0.70837	0.70855	0.00016
19.89	0.70858	0.70860	0.00017	25.34	0.70834	0.70856	0.00015
19.84	0.70849	0.70858	0.00017	25.29	0.70908	0.70857	0.00015
19.80	0.70831	0.70858	0.00017	25.25	0.70842	0.70849	0.00010
19.76	0.70853	0.70862	0.00016	25.21	0.70863	0.70852	0.00011
19.71	0.70886	0.70860	0.00018	25.16	0.70839	0.70852	0.00011
19.67	0.70871	0.70858	0.00017	25.12	0.70852	0.70857	0.00013
19.63	0.70858	0.70853	0.00018	25.08	0.70848	0.70858	0.00013
19.58	0.70915	0.70854	0.00019	25.03	0.70836	0.70860	0.00013
19.54	0.70825	0.70850	0.00014	24.99	0.70887	0.70865	0.00013
19.50	0.70857	0.70853	0.00013	24.95	0.70847	0.70862	0.00012
19.45	0.70834	0.70853	0.00013	24.90	0.70847	0.70864	0.00011
19.41	0.70852	0.70853	0.00013	24.86	0.70829	0.70868	0.00011

19.37	0.70870	0.70851	0.00013	24.82	0.70866	0.70874	0.00009
19.33	0.70829	0.70850	0.00013	24.77	0.70863	0.70871	0.00013
19.28	0.70868	0.70851	0.00013	24.73	0.70891	0.70872	0.00012
19.24	0.70816	0.70853	0.00015	24.69	0.70868	0.70864	0.00017
19.20	0.70873	0.70856	0.00013	24.64	0.70866	0.70868	0.00019
19.15	0.70871	0.70853	0.00013	24.60	0.70887	0.70872	0.00020
19.11	0.70856	0.70851	0.00012	24.56	0.70858	0.70870	0.00020
19.07	0.70859	0.70852	0.00012	24.52	0.70860	0.70866	0.00022
19.02	0.70830	0.70852	0.00012	24.47	0.70887	0.70865	0.00022
18.98	0.70836	0.70855	0.00011	24.43	0.70894	0.70863	0.00022
18.94	0.70862	0.70856	0.00010	24.39	0.70830	0.70856	0.00022
18.89	0.70835	0.70853	0.00012	24.34	0.70875	0.70859	0.00021
18.85	0.70894	0.70855	0.00011	24.30	0.70811	0.70859	0.00021
18.81	0.70846	0.70852	0.00008	24.26	0.70913	0.70866	0.00018
18.76	0.70839	0.70852	0.00008	24.21	0.70903	0.70865	0.00018
18.72	0.70855	0.70852	0.00008	24.17	0.70864	0.70863	0.00016
18.68	0.70860	0.70851	0.00008	24.13	0.70824	0.70867	0.00018
18.63	0.70863	0.70850	0.00008	24.08	0.70850	0.70869	0.00015
18.59	0.70856	0.70849	0.00008	24.04	0.70862	0.70869	0.00016
18.55	0.70852	0.70851	0.00009	24.00	0.70825	0.70873	0.00016
18.50	0.70826	0.70850	0.00009	23.95	0.70861	0.70874	0.00015
18.46	0.70862	0.70850	0.00009	23.91	0.70875	0.70872	0.00016
18.42	0.70865	0.70847	0.00009	23.87	0.70877	0.70872	0.00016
18.37	0.70839	0.70847	0.00009	23.82	0.70907	0.70869	0.00016
18.33	0.70846	0.70845	0.00010	23.78	0.70885	0.70867	0.00015
18.29	0.70840	0.70850	0.00014	23.74	0.70899	0.70868	0.00015
18.24	0.70849	0.70851	0.00014	23.70	0.70851	0.70869	0.00017
18.20	0.70859	0.70855	0.00015	23.65	0.70849	0.70873	0.00017
18.16	0.70871	0.70855	0.00015	23.61	0.70898	0.70871	0.00018
18.11	0.70846	0.70850	0.00017	23.57	0.70836	0.70866	0.00018
18.07	0.70824	0.70850	0.00017	23.52	0.70847	0.70869	0.00017
18.03	0.70829	0.70855	0.00016	23.48	0.70870	0.70873	0.00016
17.98	0.70865	0.70857	0.00015	23.44	0.70850	0.70870	0.00017
17.94	0.70824	0.70854	0.00015	23.39	0.70885	0.70871	0.00017
17.90	0.70894	0.70858	0.00013	23.35	0.70892	0.70871	0.00017
17.86	0.70853	0.70853	0.00011	23.31	0.70915	0.70869	0.00017
17.81	0.70881	0.70852	0.00011	23.26	0.70888	0.70867	0.00014
17.77	0.70866	0.70849	0.00009	23.22	0.70832	0.70864	0.00013
17.73	0.70817	0.70847	0.00008	23.18	0.70841	0.70861	0.00018
17.68	0.70852	0.70853	0.00007	23.13	0.70869	0.70862	0.00017
17.64	0.70866	0.70854	0.00007	23.09	0.70887	0.70861	0.00017
17.60	0.70854	0.70849	0.00011	23.05	0.70840	0.70857	0.00017
17.55	0.70839	0.70848	0.00011	23.00	0.70864	0.70863	0.00018
17.51	0.70858	0.70852	0.00013	22.96	0.70888	0.70867	0.00019
17.47	0.70840	0.70852	0.00013	22.92	0.70871	0.70867	0.00020
17.42	0.70850	0.70853	0.00012	22.88	0.70887	0.70865	0.00020
17.38	0.70846	0.70853	0.00013	22.83	0.70863	0.70866	0.00020
17.34	0.70852	0.70857	0.00014	22.79	0.70796	0.70869	0.00021
17.29	0.70873	0.70854	0.00015	22.75	0.70857	0.70874	0.00014
17.25	0.70866	0.70854	0.00016	22.70	0.70858	0.70882	0.00019

17.21	0.70811	0.70854	0.00016	22.66	0.70850	0.70886	0.00018
17.16	0.70844	0.70859	0.00013	22.62	0.70900	0.70889	0.00016
17.12	0.70884	0.70858	0.00013	22.57	0.70899	0.70887	0.00016
17.08	0.70859	0.70857	0.00012	22.53	0.70893	0.70881	0.00019
17.03	0.70847	0.70857	0.00012	22.49	0.70851	0.70881	0.00019
16.99	0.70847	0.70858	0.00012	22.44	0.70893	0.70883	0.00018
16.95	0.70882	0.70864	0.00015	22.40	0.70891	0.70883	0.00018
16.90	0.70823	0.70862	0.00015	22.36	0.70846	0.70886	0.00019
16.86	0.70881	0.70867	0.00012	22.31	0.70936	0.70888	0.00018
16.82	0.70863	0.70865	0.00011	22.27	0.70901	0.70879	0.00015
16.77	0.70863	0.70860	0.00015	22.23	0.70879	0.70874	0.00016
16.73	0.70834	0.70858	0.00016	22.18	0.70880	0.70878	0.00018
16.69	0.70869	0.70859	0.00015	22.14	0.70838	0.70874	0.00019
16.64	0.70860	0.70858	0.00015	22.10	0.70893	0.70878	0.00018
16.60	0.70856	0.70858	0.00015	22.06	0.70870	0.70875	0.00018
16.56	0.70906	0.70856	0.00015	22.01	0.70892	0.70877	0.00018
16.52	0.70868	0.70853	0.00012	21.97	0.70923	0.70877	0.00018
16.47	0.70874	0.70851	0.00011	21.93	0.70867	0.70874	0.00015
16.43	0.70857	0.70851	0.00011	21.88	0.70851	0.70875	0.00015
16.39	0.70814	0.70851	0.00011	21.84	0.70847	0.70880	0.00015
16.34	0.70842	0.70851	0.00009	21.80	0.70918	0.70879	0.00017
16.30	0.70844	0.70853	0.00009	21.75	0.70843	0.70878	0.00016
16.26	0.70856	0.70849	0.00013	21.71	0.70880	0.70885	0.00015
16.21	0.70860	0.70850	0.00013	21.67	0.70863	0.70880	0.00018
16.17	0.70838	0.70850	0.00013	21.62	0.70886	0.70882	0.00018
16.13	0.70874	0.70853	0.00013	21.58	0.70896	0.70887	0.00021
16.08	0.70855	0.70854	0.00014	21.54	0.70891	0.70884	0.00021
16.04	0.70867	0.70858	0.00016	21.49	0.70876	0.70877	0.00025
16.00	0.70855	0.70854	0.00017	21.45	0.70902	0.70876	0.00025
15.95	0.70823	0.70856	0.00017	21.41	0.70832	0.70868	0.00026
15.91	0.70857	0.70858	0.00016	21.36	0.70913	0.70870	0.00025
15.87	0.70808	0.70858	0.00016	21.32	0.70906	0.70861	0.00025
15.82	0.70865	0.70862	0.00012	21.28	0.70833	0.70856	0.00023
15.78	0.70856	0.70861	0.00012	21.23	0.70888	0.70864	0.00024
15.74	0.70869	0.70862	0.00013	21.19	0.70935	0.70861	0.00024
15.69	0.70885	0.70865	0.00015	21.15	0.70860	0.70854	0.00018
15.65	0.70894	0.70862	0.00014	21.11	0.70820	0.70857	0.00019
15.61	0.70831	0.70855	0.00014	21.06	0.70873	0.70861	0.00017
15.56	0.70873	0.70856	0.00014	21.02	0.70822	0.70867	0.00022
15.52	0.70841	0.70854	0.00013	20.98	0.70849	0.70875	0.00021
15.48	0.70860	0.70861	0.00016	20.93	0.70824	0.70880	0.00020
15.43	0.70847	0.70860	0.00016	20.89	0.70855	0.70882	0.00018
15.39	0.70850	0.70859	0.00017	20.85	0.70911	0.70885	0.00017
15.35	0.70873	0.70865	0.00019	20.80	0.70860	0.70883	0.00016
15.30	0.70898	0.70863	0.00019	20.76	0.70868	0.70884	0.00016
15.26	0.70857	0.70855	0.00018	20.72	0.70889	0.70882	0.00017
15.22	0.70825	0.70850	0.00021	20.67	0.70858	0.70878	0.00018
15.17	0.70834	0.70855	0.00020	20.63	0.70932	0.70878	0.00018
15.13	0.70857	0.70858	0.00020	20.59	0.70904	0.70869	0.00015
15.09	0.70907	0.70857	0.00020	20.54	0.70897	0.70869	0.00014

15.05	0.70856	0.70850	0.00017	20.50	0.70849	0.70869	0.00014
15.00	0.70837	0.70854	0.00019	20.46	0.70881	0.70873	0.00014
14.96	0.70903	0.70857	0.00019	20.41	0.70895	0.70874	0.00015
14.92	0.70852	0.70853	0.00016	20.37	0.70867	0.70874	0.00014
14.87	0.70826	0.70853	0.00016	20.33	0.70853	0.70875	0.00014
14.83	0.70805	0.70858	0.00015	20.29	0.70846	0.70874	0.00015
14.79	0.70873	0.70863	0.00009	20.24	0.70858	0.70879	0.00013
14.74	0.70860	0.70861	0.00009	20.20	0.70844	0.70874	0.00019
14.70	0.70852	0.70862	0.00009	20.16	0.70903	0.70874	0.00020
14.66	0.70841	0.70862	0.00009	20.11	0.70898	0.70870	0.00018
14.61	0.70894	0.70863	0.00008	20.07	0.70884	0.70869	0.00018
14.57	0.70865	0.70860	0.00004	20.03	0.70894	0.70860	0.00023
14.53	0.70863	0.70858	0.00005	19.98	0.70893	0.70857	0.00022
14.48	0.70855	0.70860	0.00007	19.94	0.70874	0.70854	0.00020
14.44	0.70870	0.70859	0.00007	19.90	0.70851	0.70851	0.00020
14.40	0.70859	0.70858	0.00007	19.85	0.70891	0.70855	0.00022
14.35	0.70851	0.70862	0.00012	19.81	0.70809	0.70852	0.00020
14.31	0.70869	0.70862	0.00012	19.77	0.70839	0.70860	0.00019
14.27	0.70850	0.70859	0.00012	19.72	0.70866	0.70862	0.00018
14.22	0.70856	0.70862	0.00013	19.68	0.70885	0.70861	0.00018
14.18	0.70859	0.70860	0.00013	19.64	0.70793	0.70860	0.00018
14.14	0.70847	0.70863	0.00014	19.59	0.70870	0.70865	0.00012
14.09	0.70882	0.70865	0.00014	19.55	0.70864	0.70865	0.00012
14.05	0.70848	0.70864	0.00013	19.51	0.70841	0.70869	0.00014
14.01	0.70857	0.70864	0.00014	19.47	0.70893	0.70871	0.00013
13.96	0.70906	0.70861	0.00015	19.42	0.70857	0.70863	0.00017
13.92	0.70849	0.70856	0.00012	19.38	0.70890	0.70863	0.00017
13.88	0.70840	0.70858	0.00012	19.34	0.70859	0.70862	0.00016
13.83	0.70873	0.70862	0.00012	19.29	0.70857	0.70861	0.00016
13.79	0.70840	0.70862	0.00012	19.25	0.70878	0.70864	0.00017
13.75	0.70886	0.70866	0.00011	19.21	0.70838	0.70857	0.00020
13.70	0.70871	0.70865	0.00010	19.16	0.70871	0.70855	0.00021
13.66	0.70872	0.70860	0.00014	19.12	0.70907	0.70854	0.00021
13.62	0.70843	0.70858	0.00013	19.08	0.70859	0.70852	0.00019
13.58	0.70832	0.70859	0.00013	19.03	0.70810	0.70853	0.00019
13.53	0.70868	0.70862	0.00011	18.99	0.70861	0.70857	0.00017
13.49	0.70876	0.70860	0.00012	18.95	0.70876	0.70860	0.00018
13.45	0.70866	0.70863	0.00014	18.90	0.70853	0.70857	0.00018
13.40	0.70879	0.70860	0.00015	18.86	0.70886	0.70858	0.00018
13.36	0.70880	0.70856	0.00015	18.82	0.70810	0.70853	0.00018
13.32	0.70824	0.70852	0.00014	18.77	0.70818	0.70856	0.00015
13.27	0.70851	0.70853	0.00014	18.73	0.70857	0.70861	0.00013
13.23	0.70855	0.70856	0.00015	18.69	0.70891	0.70863	0.00013
13.19	0.70874	0.70857	0.00015	18.65	0.70865	0.70857	0.00013
13.14	0.70847	0.70858	0.00016	18.60	0.70855	0.70855	0.00013
13.10	0.70848	0.70861	0.00016	18.56	0.70894	0.70855	0.00013
13.06	0.70902	0.70864	0.00016	18.52	0.70840	0.70852	0.00011
13.01	0.70843	0.70864	0.00016	18.47	0.70863	0.70852	0.00011
12.97	0.70836	0.70863	0.00017	18.43	0.70836	0.70853	0.00011
12.93	0.70843	0.70861	0.00018	18.39	0.70838	0.70853	0.00011

12.88	0.70827	0.70864	0.00018
12.84	0.70885	0.70865	0.00017
12.80	0.70868	0.70863	0.00017
12.71	0.70884	0.70865	0.00018
12.67	0.70877	0.70861	0.00017
12.62	0.70871	0.70858	0.00017
12.58	0.70902	0.70857	0.00017
12.54	0.70832	0.70851	0.00014
12.49	0.70819	0.70853	0.00013
12.45	0.70876	0.70858	0.00011
12.41	0.70830	0.70856	0.00010
12.36	0.70870	0.70857	0.00010
12.32	0.70886	0.70856	0.00009
12.28	0.70850	0.70853	0.00006
12.23	0.70847	0.70856	0.00009
12.19	0.70859	0.70857	0.00009
12.15	0.70839	0.70859	0.00010
12.11	0.70851	0.70863	0.00010
12.06	0.70869	0.70861	0.00011
12.02	0.70861	0.70859	0.00011
11.98	0.70837	0.70858	0.00011
11.93	0.70859	0.70858	0.00011
11.89	0.70856	0.70859	0.00011
11.85	0.70886	0.70858	0.00012
11.80	0.70850	0.70855	0.00010
11.76	0.70883	0.70855	0.00010
11.72	0.70875	0.70855	0.00010
11.67	0.70837	0.70852	0.00009
11.63	0.70850	0.70851	0.00011
11.59	0.70850	0.70852	0.00011
11.54	0.70835	0.70855	0.00012
11.50	0.70865	0.70861	0.00013
11.46	0.70849	0.70858	0.00013
11.41	0.70849	0.70853	0.00015
11.37	0.70881	0.70853	0.00015
11.33	0.70855	0.70851	0.00014
11.28	0.70821	0.70853	0.00015
11.24	0.70862	0.70856	0.00013
11.20	0.70878	0.70861	0.00016
11.15	0.70885	0.70859	0.00016
11.11	0.70841	0.70855	0.00014
11.07	0.70829	0.70856	0.00014
11.02	0.70825	0.70854	0.00017
10.98	0.70850	0.70860	0.00017
10.94	0.70869	0.70860	0.00017
10.89	0.70869	0.70855	0.00019
10.85	0.70857	0.70854	0.00018
10.81	0.70905	0.70854	0.00018
10.77	0.70858	0.70852	0.00016
10.72	0.70852	0.70856	0.00019

18.34	0.70872	0.70852	0.00013
18.30	0.70876	0.70849	0.00012
18.26	0.70832	0.70848	0.00011
18.21	0.70847	0.70860	0.00023
18.17	0.70849	0.70864	0.00024
18.13	0.70872	0.70867	0.00023
18.08	0.70835	0.70866	0.00023
18.04	0.70873	0.70872	0.00023
18.00	0.70841	0.70871	0.00023
17.95	0.70821	0.70873	0.00022
17.91	0.70845	0.70876	0.00020
17.87	0.70865	0.70879	0.00018
17.83	0.70954	0.70879	0.00018
17.78	0.70887	0.70875	0.00011
17.74	0.70875	0.70875	0.00011
17.70	0.70865	0.70876	0.00011
17.65	0.70891	0.70872	0.00016
17.61	0.70863	0.70870	0.00015
17.57	0.70861	0.70871	0.00015
17.52	0.70851	0.70871	0.00015
17.48	0.70878	0.70870	0.00015
17.44	0.70869	0.70870	0.00015
17.39	0.70910	0.70869	0.00016
17.35	0.70887	0.70865	0.00013
17.31	0.70889	0.70861	0.00013
17.26	0.70820	0.70864	0.00018
17.22	0.70877	0.70870	0.00015
17.18	0.70864	0.70868	0.00015
17.13	0.70861	0.70875	0.00020
17.09	0.70849	0.70875	0.00019
17.05	0.70878	0.70877	0.00019
17.01	0.70855	0.70879	0.00019
16.96	0.70874	0.70878	0.00019
16.92	0.70838	0.70877	0.00020
16.88	0.70925	0.70880	0.00018
16.83	0.70876	0.70874	0.00015
16.79	0.70860	0.70873	0.00015
16.75	0.70932	0.70874	0.00015
16.70	0.70866	0.70869	0.00007
16.66	0.70865	0.70865	0.00010
16.62	0.70895	0.70864	0.00011
16.57	0.70852	0.70857	0.00012
16.53	0.70863	0.70865	0.00020
16.49	0.70862	0.70859	0.00024
16.44	0.70868	0.70856	0.00025
16.40	0.70870	0.70853	0.00025
16.36	0.70870	0.70856	0.00027
16.31	0.70878	0.70854	0.00027
16.27	0.70831	0.70855	0.00027
16.23	0.70857	0.70860	0.00027

10.68	0.70846	0.70858	0.00019
10.64	0.70806	0.70861	0.00019
10.59	0.70892	0.70867	0.00015
10.55	0.70848	0.70866	0.00014
10.51	0.70821	0.70865	0.00014
10.46	0.70858	0.70867	0.00012
10.42	0.70854	0.70869	0.00011
10.38	0.70888	0.70871	0.00011
10.33	0.70898	0.70868	0.00011
10.29	0.70870	0.70861	0.00012
10.25	0.70874	0.70862	0.00012
10.20	0.70866	0.70860	0.00012
10.16	0.70880	0.70859	0.00012
10.12	0.70840	0.70856	0.00011
10.07	0.70846	0.70856	0.00011
10.03	0.70872	0.70859	0.00011
9.99	0.70878	0.70859	0.00012
9.94	0.70860	0.70857	0.00011
9.90	0.70823	0.70860	0.00013
9.86	0.70882	0.70863	0.00010
9.81	0.70852	0.70857	0.00011
9.77	0.70856	0.70857	0.00011
9.73	0.70853	0.70860	0.00013
9.68	0.70840	0.70857	0.00014
9.64	0.70872	0.70858	0.00014
9.60	0.70876	0.70854	0.00015
9.55	0.70856	0.70851	0.00014
9.51	0.70887	0.70853	0.00014
9.47	0.70853	0.70851	0.00013
9.42	0.70827	0.70855	0.00015
9.38	0.70847	0.70855	0.00014
9.34	0.70887	0.70857	0.00014
9.30	0.70826	0.70854	0.00013
9.25	0.70849	0.70858	0.00011
9.21	0.70832	0.70860	0.00011
9.17	0.70848	0.70860	0.00011
9.12	0.70869	0.70863	0.00011
9.08	0.70872	0.70859	0.00013
9.04	0.70888	0.70853	0.00016
8.99	0.70835	0.70850	0.00014
8.95	0.70864	0.70849	0.00014
8.91	0.70861	0.70849	0.00014
8.86	0.70863	0.70851	0.00015
8.82	0.70867	0.70850	0.00015
8.78	0.70832	0.70849	0.00015
8.73	0.70880	0.70853	0.00015
8.69	0.70826	0.70850	0.00014
8.65	0.70811	0.70852	0.00013
8.60	0.70856	0.70852	0.00013
8.56	0.70828	0.70855	0.00015

16.19	0.70819	0.70865	0.00029
16.14	0.70937	0.70870	0.00027
16.10	0.70797	0.70866	0.00024
16.06	0.70832	0.70877	0.00019
16.01	0.70840	0.70881	0.00016
15.97	0.70904	0.70890	0.00016
15.93	0.70840	0.70885	0.00017
15.88	0.70888	0.70890	0.00014
15.84	0.70889	0.70884	0.00018
15.80	0.70906	0.70885	0.00018
15.75	0.70866	0.70883	0.00018
15.71	0.70900	0.70888	0.00018
15.67	0.70905	0.70886	0.00018
15.62	0.70871	0.70884	0.00017
15.58	0.70932	0.70882	0.00019
15.54	0.70854	0.70874	0.00016
15.49	0.70886	0.70870	0.00020
15.45	0.70835	0.70869	0.00020
15.41	0.70900	0.70873	0.00018
15.37	0.70886	0.70871	0.00017
15.32	0.70911	0.70877	0.00022
15.28	0.70885	0.70883	0.00028
15.24	0.70884	0.70881	0.00028
15.19	0.70847	0.70879	0.00028
15.15	0.70854	0.70877	0.00030
15.11	0.70813	0.70884	0.00030
15.06	0.70877	0.70889	0.00026
15.02	0.70875	0.70894	0.00027
14.98	0.70881	0.70889	0.00030
14.93	0.70944	0.70883	0.00033
14.89	0.70966	0.70877	0.00030
14.85	0.70874	0.70868	0.00023
14.80	0.70864	0.70869	0.00024
14.76	0.70823	0.70875	0.00026
14.72	0.70920	0.70881	0.00023
14.67	0.70870	0.70872	0.00023
14.63	0.70922	0.70875	0.00024
14.59	0.70830	0.70872	0.00022
14.55	0.70815	0.70882	0.00023
14.50	0.70882	0.70886	0.00019
14.46	0.70875	0.70884	0.00020
14.42	0.70893	0.70881	0.00020
14.37	0.70918	0.70878	0.00021
14.33	0.70888	0.70873	0.00019
14.29	0.70830	0.70872	0.00018
14.24	0.70899	0.70875	0.00016
14.20	0.70892	0.70870	0.00016
14.16	0.70932	0.70867	0.00015
14.11	0.70851	0.70862	0.00006
14.07	0.70857	0.70863	0.00005

8.52	0.70869	0.70858	0.00014	14.03	0.70855	0.70866	0.00007
8.47	0.70879	0.70856	0.00014	13.98	0.70861	0.70872	0.00011
8.43	0.70851	0.70853	0.00013	13.94	0.70868	0.70874	0.00011
8.39	0.70860	0.70855	0.00013	13.90	0.70876	0.70873	0.00011
8.34	0.70867	0.70855	0.00013	13.85	0.70860	0.70871	0.00012
8.30	0.70857	0.70855	0.00013	13.81	0.70854	0.70868	0.00014
8.26	0.70837	0.70857	0.00014	13.77	0.70860	0.70866	0.00016
8.21	0.70811	0.70857	0.00014	13.73	0.70875	0.70866	0.00016
8.17	0.70891	0.70864	0.00010	13.68	0.70861	0.70865	0.00016
8.13	0.70856	0.70861	0.00008	13.64	0.70892	0.70863	0.00017
8.08	0.70854	0.70860	0.00008	13.60	0.70911	0.70859	0.00015
8.04	0.70849	0.70861	0.00008	13.55	0.70881	0.70853	0.00010
8.00	0.70873	0.70862	0.00008	13.51	0.70862	0.70849	0.00008
7.95	0.70858	0.70860	0.00008	13.47	0.70856	0.70849	0.00008
7.91	0.70869	0.70857	0.00010	13.42	0.70831	0.70847	0.00008
7.87	0.70870	0.70854	0.00010	13.38	0.70831	0.70850	0.00008
7.83	0.70836	0.70851	0.00010	13.34	0.70857	0.70854	0.00008
7.78	0.70879	0.70854	0.00010	13.29	0.70865	0.70859	0.00014
7.74	0.70860	0.70852	0.00008	13.25	0.70839	0.70864	0.00017
7.70	0.70851	0.70851	0.00008	13.21	0.70856	0.70866	0.00016
7.65	0.70860	0.70853	0.00009	13.16	0.70847	0.70866	0.00016
7.61	0.70866	0.70854	0.00010	13.12	0.70848	0.70872	0.00017
7.57	0.70845	0.70852	0.00010	13.08	0.70862	0.70870	0.00019
7.52	0.70830	0.70851	0.00010	13.03	0.70831	0.70874	0.00020
7.48	0.70840	0.70850	0.00011	12.99	0.70866	0.70878	0.00017
7.44	0.70838	0.70851	0.00011	12.95	0.70867	0.70878	0.00017
7.39	0.70869	0.70851	0.00010	12.90	0.70910	0.70875	0.00019
7.35	0.70862	0.70846	0.00012	12.86	0.70909	0.70866	0.00020
7.31	0.70848	0.70848	0.00013	12.82	0.70861	0.70863	0.00017
7.26	0.70869	0.70851	0.00015	12.78	0.70858	0.70867	0.00018
7.22	0.70873	0.70851	0.00015	12.73	0.70904	0.70863	0.00020
7.18	0.70842	0.70848	0.00014	12.69	0.70828	0.70865	0.00023
7.13	0.70834	0.70854	0.00017	12.65	0.70902	0.70864	0.00024
7.09	0.70826	0.70858	0.00017	12.60	0.70873	0.70859	0.00022
7.05	0.70844	0.70861	0.00016	12.56	0.70868	0.70862	0.00024
7.00	0.70843	0.70864	0.00015	12.52	0.70838	0.70866	0.00026
6.96	0.70816	0.70863	0.00016	12.47	0.70825	0.70868	0.00025
6.92	0.70882	0.70869	0.00012	12.43	0.70878	0.70876	0.00025
6.87	0.70877	0.70866	0.00012	12.39	0.70893	0.70876	0.00025
6.83	0.70877	0.70863	0.00012	12.34	0.70825	0.70872	0.00025
6.79	0.70840	0.70859	0.00013	12.30	0.70927	0.70882	0.00025
6.74	0.70897	0.70861	0.00012	12.26	0.70816	0.70882	0.00025
6.70	0.70876	0.70860	0.00010	12.21	0.70844	0.70888	0.00020
6.66	0.70858	0.70861	0.00011	12.17	0.70906	0.70894	0.00018
6.57	0.70872	0.70860	0.00011	12.13	0.70911	0.70892	0.00018
6.53	0.70837	0.70863	0.00013	12.08	0.70854	0.70889	0.00017
6.48	0.70874	0.70863	0.00013	12.04	0.70912	0.70891	0.00016
6.44	0.70849	0.70860	0.00014	12.00	0.70875	0.70889	0.00015
6.40	0.70849	0.70864	0.00015	11.96	0.70855	0.70885	0.00018
6.36	0.70838	0.70866	0.00015	11.91	0.70926	0.70889	0.00017

6.31	0.70864	0.70872	0.00015	11.87	0.70927	0.70882	0.00016
6.27	0.70880	0.70874	0.00015	11.83	0.70869	0.70877	0.00012
6.23	0.70888	0.70872	0.00015	11.78	0.70906	0.70876	0.00013
6.18	0.70850	0.70871	0.00015	11.74	0.70889	0.70874	0.00011
6.14	0.70897	0.70872	0.00015	11.70	0.70882	0.70870	0.00012
6.10	0.70844	0.70868	0.00014	11.65	0.70875	0.70880	0.00026
6.05	0.70838	0.70867	0.00015	11.61	0.70887	0.70880	0.00026
6.01	0.70898	0.70870	0.00014	11.57	0.70839	0.70880	0.00026
5.97	0.70865	0.70865	0.00013	11.52	0.70890	0.70881	0.00025
5.92	0.70895	0.70865	0.00013	11.48	0.70853	0.70878	0.00026
5.88	0.70888	0.70863	0.00011	11.44	0.70875	0.70873	0.00029
5.84	0.70852	0.70859	0.00010	11.39	0.70859	0.70869	0.00030
5.79	0.70887	0.70861	0.00010	11.35	0.70890	0.70870	0.00030
5.75	0.70856	0.70857	0.00008	11.31	0.70846	0.70863	0.00031
5.71	0.70856	0.70861	0.00011	11.26	0.70983	0.70870	0.00032
5.66	0.70829	0.70863	0.00011	11.22	0.70875	0.70859	0.00021
5.62	0.70869	0.70865	0.00009	11.18	0.70893	0.70858	0.00021
5.58	0.70853	0.70862	0.00011	11.14	0.70849	0.70854	0.00019
5.53	0.70865	0.70866	0.00012	11.09	0.70855	0.70862	0.00024
5.49	0.70873	0.70865	0.00012	11.05	0.70810	0.70863	0.00024
5.45	0.70853	0.70862	0.00013	11.01	0.70834	0.70865	0.00022
5.40	0.70864	0.70867	0.00014	10.96	0.70864	0.70866	0.00022
5.36	0.70853	0.70865	0.00015	10.92	0.70821	0.70867	0.00022
5.32	0.70895	0.70866	0.00014	10.88	0.70920	0.70874	0.00020
5.27	0.70880	0.70863	0.00013	10.83	0.70871	0.70870	0.00017
5.23	0.70846	0.70859	0.00013	10.79	0.70858	0.70867	0.00017
5.19	0.70836	0.70860	0.00013	10.75	0.70858	0.70869	0.00017
5.14	0.70892	0.70863	0.00011	10.70	0.70928	0.70869	0.00017
5.10	0.70858	0.70858	0.00010	10.66	0.70864	0.70861	0.00011
5.06	0.70847	0.70854	0.00013	10.62	0.70834	0.70856	0.00015
5.02	0.70895	0.70851	0.00015	10.57	0.70840	0.70858	0.00014
4.97	0.70849	0.70847	0.00011	10.53	0.70875	0.70865	0.00016
4.93	0.70863	0.70850	0.00013	10.49	0.70892	0.70867	0.00018
4.89	0.70864	0.70853	0.00015	10.44	0.70875	0.70868	0.00019
4.84	0.70843	0.70852	0.00015	10.40	0.70846	0.70871	0.00020
4.80	0.70849	0.70853	0.00015	10.36	0.70873	0.70875	0.00019
4.76	0.70864	0.70850	0.00016	10.32	0.70859	0.70874	0.00020
4.71	0.70842	0.70850	0.00016	10.27	0.70856	0.70872	0.00020
4.67	0.70818	0.70851	0.00016	10.23	0.70815	0.70875	0.00020
4.63	0.70819	0.70858	0.00015	10.19	0.70849	0.70880	0.00016
4.58	0.70856	0.70860	0.00013	10.14	0.70906	0.70883	0.00015
4.54	0.70884	0.70859	0.00013	10.10	0.70903	0.70876	0.00015
4.50	0.70890	0.70855	0.00012	10.06	0.70902	0.70874	0.00015
4.45	0.70854	0.70853	0.00010	10.01	0.70899	0.70867	0.00016
4.41	0.70853	0.70858	0.00014	9.97	0.70894	0.70869	0.00017
4.37	0.70824	0.70861	0.00015	9.93	0.70855	0.70866	0.00017
4.32	0.70862	0.70865	0.00012	9.88	0.70843	0.70869	0.00017
4.28	0.70854	0.70863	0.00013	9.84	0.70889	0.70872	0.00016
4.24	0.70880	0.70865	0.00013	9.80	0.70856	0.70869	0.00016
4.19	0.70845	0.70862	0.00013	9.75	0.70879	0.70871	0.00016

4.15	0.70847	0.70861	0.00014	9.71	0.70843	0.70871	0.00016
4.11	0.70843	0.70862	0.00014	9.67	0.70886	0.70873	0.00014
4.06	0.70870	0.70861	0.00015	9.62	0.70830	0.70871	0.00014
4.02	0.70903	0.70860	0.00015	9.58	0.70917	0.70875	0.00011
3.98	0.70880	0.70857	0.00012	9.54	0.70860	0.70870	0.00006
3.93	0.70863	0.70851	0.00012	9.50	0.70890	0.70872	0.00006
3.89	0.70845	0.70853	0.00014	9.45	0.70872	0.70869	0.00005
3.85	0.70877	0.70851	0.00015	9.41	0.70861	0.70867	0.00007
3.80	0.70852	0.70848	0.00014	9.37	0.70868	0.70871	0.00010
3.76	0.70826	0.70848	0.00014	9.32	0.70879	0.70871	0.00010
3.72	0.70864	0.70850	0.00013	9.28	0.70865	0.70870	0.00010
3.67	0.70830	0.70852	0.00015	9.24	0.70868	0.70874	0.00012
3.63	0.70860	0.70854	0.00015	9.19	0.70868	0.70874	0.00012
3.59	0.70870	0.70854	0.00015	9.15	0.70865	0.70875	0.00012
3.55	0.70824	0.70853	0.00014	9.11	0.70888	0.70875	0.00012
3.50	0.70887	0.70855	0.00013	9.06	0.70859	0.70872	0.00012
3.46	0.70825	0.70851	0.00011	9.02	0.70847	0.70869	0.00014
3.42	0.70843	0.70855	0.00009	8.98	0.70901	0.70872	0.00013
3.37	0.70849	0.70857	0.00008	8.93	0.70874	0.70868	0.00011
3.33	0.70844	0.70859	0.00009	8.89	0.70867	0.70867	0.00011
3.29	0.70891	0.70861	0.00008	8.85	0.70906	0.70872	0.00016
3.24	0.70850	0.70858	0.00005	8.80	0.70863	0.70868	0.00014
3.20	0.70861	0.70859	0.00005	8.76	0.70877	0.70875	0.00019
3.16	0.70852	0.70855	0.00008	8.72	0.70868	0.70877	0.00019
3.11	0.70848	0.70857	0.00008	8.68	0.70856	0.70873	0.00021
3.07	0.70851	0.70857	0.00008	8.63	0.70834	0.70879	0.00022
3.03	0.70862	0.70862	0.00011	8.59	0.70874	0.70885	0.00020
2.98	0.70859	0.70864	0.00012	8.55	0.70865	0.70884	0.00020
2.94	0.70868	0.70863	0.00013	8.50	0.70861	0.70884	0.00020
2.90	0.70873	0.70864	0.00013	8.46	0.70920	0.70888	0.00020
2.85	0.70858	0.70862	0.00013	8.42	0.70868	0.70881	0.00019
2.81	0.70856	0.70867	0.00015	8.37	0.70933	0.70883	0.00019
2.77	0.70825	0.70866	0.00015	8.33	0.70890	0.70875	0.00016
2.72	0.70865	0.70871	0.00012	8.29	0.70828	0.70878	0.00018
2.68	0.70856	0.70872	0.00012	8.24	0.70915	0.70880	0.00016
2.64	0.70897	0.70869	0.00014	8.20	0.70896	0.70873	0.00015
2.59	0.70886	0.70865	0.00012	8.16	0.70868	0.70871	0.00015
2.55	0.70846	0.70859	0.00014	8.11	0.70866	0.70875	0.00017
2.51	0.70874	0.70859	0.00014	8.07	0.70894	0.70871	0.00020
2.46	0.70859	0.70856	0.00014	8.03	0.70855	0.70865	0.00020
2.42	0.70899	0.70854	0.00014	7.98	0.70884	0.70869	0.00020
2.38	0.70851	0.70846	0.00011	7.94	0.70855	0.70865	0.00020
2.33	0.70876	0.70849	0.00014	7.90	0.70917	0.70867	0.00020
2.29	0.70869	0.70846	0.00012	7.86	0.70851	0.70860	0.00017
2.25	0.70834	0.70844	0.00011	7.81	0.70840	0.70863	0.00018
2.20	0.70858	0.70848	0.00012	7.77	0.70880	0.70863	0.00018
2.16	0.70822	0.70852	0.00015	7.73	0.70912	0.70858	0.00018
2.12	0.70849	0.70855	0.00014	7.68	0.70821	0.70849	0.00015
2.08	0.70837	0.70857	0.00014	7.64	0.70839	0.70853	0.00014
2.03	0.70843	0.70858	0.00014	7.60	0.70889	0.70856	0.00014

1.99	0.70823	0.70861	0.00014
1.95	0.70882	0.70864	0.00011
1.90	0.70845	0.70864	0.00011
1.86	0.70851	0.70864	0.00010
1.82	0.70866	0.70868	0.00010
1.77	0.70899	0.70868	0.00010
1.73	0.70858	0.70860	0.00012
1.69	0.70867	0.70861	0.00012
1.64	0.70843	0.70861	0.00012
1.60	0.70873	0.70858	0.00016
1.56	0.70856	0.70855	0.00016
1.51	0.70878	0.70855	0.00016
1.47	0.70854	0.70851	0.00015
1.43	0.70883	0.70849	0.00015
1.38	0.70873	0.70846	0.00013
1.34	0.70819	0.70843	0.00012
1.30	0.70869	0.70847	0.00011
1.25	0.70866	0.70847	0.00011
1.21	0.70808	0.70841	0.00014
1.17	0.70848	0.70849	0.00016
1.12	0.70852	0.70852	0.00017
1.08	0.70836	0.70851	0.00017
1.04	0.70840	0.70852	0.00016
0.99	0.70853	0.70857	0.00018
0.95	0.70841	0.70858	0.00018
0.91	0.70858	0.70861	0.00018
0.86	0.70872	0.70863	0.00018
0.82	0.70799	0.70862	0.00018
0.78	0.70895	0.70872	0.00013
0.73	0.70877	0.70873	0.00014
0.69	0.70842	0.70873	0.00014
0.65	0.70842	0.70878	0.00013
0.61	0.70895	0.70880	0.00010
0.56	0.70857	0.70881	0.00011
0.52	0.70874	0.70879	0.00015
0.48	0.70881	0.70877	0.00015
0.43	0.70859	0.70873	0.00017
0.39	0.70899	0.70875	0.00017
0.35	0.70902	0.70872	0.00017
0.30	0.70876	0.70867	0.00017
0.26	0.70890	0.70866	0.00018
0.22	0.70866	0.70861	0.00019
0.17	0.70908	0.70860	0.00022
0.13	0.70830	0.70844	0.00009
0.09	0.70857	0.70851	0.00006
0.04	0.70844		

7.55	0.70849	0.70855	0.00012
7.51	0.70868	0.70852	0.00015
7.47	0.70851	0.70857	0.00020
7.42	0.70883	0.70855	0.00020
7.38	0.70834	0.70856	0.00021
7.34	0.70833	0.70856	0.00021
7.29	0.70825	0.70863	0.00023
7.25	0.70862	0.70867	0.00021
7.21	0.70868	0.70867	0.00021
7.16	0.70875	0.70870	0.00022
7.12	0.70816	0.70866	0.00023
7.08	0.70920	0.70870	0.00021
7.04	0.70837	0.70866	0.00018
6.99	0.70888	0.70867	0.00018
6.95	0.70833	0.70864	0.00017
6.91	0.70910	0.70867	0.00016
6.86	0.70865	0.70860	0.00012
6.82	0.70854	0.70859	0.00013
6.78	0.70899	0.70859	0.00013
6.73	0.70834	0.70848	0.00016
6.69	0.70858	0.70852	0.00016
6.65	0.70886	0.70856	0.00018
6.60	0.70840	0.70856	0.00019
6.56	0.70862	0.70860	0.00019
6.52	0.70857	0.70861	0.00019
6.47	0.70847	0.70861	0.00019
6.43	0.70854	0.70862	0.00019
6.39	0.70850	0.70866	0.00019
6.34	0.70791	0.70865	0.00020
6.30	0.70871	0.70874	0.00011
6.26	0.70899	0.70874	0.00011
6.22	0.70889	0.70874	0.00011
6.17	0.70875	0.70875	0.00012
6.13	0.70871	0.70879	0.00014
6.09	0.70866	0.70878	0.00014
6.04	0.70857	0.70881	0.00015
6.00	0.70891	0.70882	0.00014
5.96	0.70841	0.70879	0.00015
5.91	0.70877	0.70886	0.00013
5.87	0.70874	0.70883	0.00015
5.83	0.70900	0.70881	0.00016
5.78	0.70897	0.70878	0.00015
5.74	0.70914	0.70881	0.00018
5.70	0.70860	0.70874	0.00017
5.65	0.70899	0.70874	0.00017
5.61	0.70871	0.70869	0.00017
5.57	0.70856	0.70870	0.00017
5.52	0.70910	0.70878	0.00020
5.48	0.70851	0.70878	0.00020
5.44	0.70855	0.70882	0.00020

5.40	0.70869	0.70883	0.00019
5.35	0.70924	0.70881	0.00020
5.31	0.70846	0.70873	0.00019
5.27	0.70861	0.70869	0.00022
5.22	0.70851	0.70873	0.00023
5.18	0.70882	0.70877	0.00023
5.14	0.70928	0.70877	0.00023
5.09	0.70916	0.70870	0.00020
5.05	0.70886	0.70858	0.00022
5.01	0.70863	0.70859	0.00023
4.96	0.70851	0.70861	0.00023
4.92	0.70845	0.70867	0.00025
4.88	0.70807	0.70874	0.00026
4.83	0.70899	0.70880	0.00021
4.79	0.70892	0.70881	0.00022
4.75	0.70886	0.70883	0.00023
4.70	0.70857	0.70883	0.00023
4.66	0.70798	0.70883	0.00023
4.62	0.70894	0.70890	0.00014
4.58	0.70880	0.70890	0.00014
4.53	0.70909	0.70888	0.00015
4.49	0.70914	0.70884	0.00015
4.45	0.70866	0.70878	0.00015
4.40	0.70913	0.70877	0.00015
4.36	0.70915	0.70875	0.00013
4.32	0.70883	0.70870	0.00010
4.27	0.70855	0.70870	0.00010
4.23	0.70873	0.70871	0.00009
4.19	0.70894	0.70869	0.00010
4.14	0.70857	0.70866	0.00009
4.10	0.70873	0.70867	0.00008
4.06	0.70849	0.70867	0.00008
4.01	0.70857	0.70865	0.00012
3.97	0.70894	0.70867	0.00012
3.93	0.70870	0.70866	0.00011
3.88	0.70877	0.70869	0.00013
3.84	0.70870	0.70870	0.00013
3.80	0.70851	0.70874	0.00015
3.75	0.70860	0.70878	0.00015
3.71	0.70873	0.70879	0.00014
3.67	0.70872	0.70876	0.00016
3.63	0.70825	0.70872	0.00017
3.58	0.70876	0.70877	0.00014
3.54	0.70890	0.70878	0.00014
3.50	0.70895	0.70874	0.00014
3.45	0.70886	0.70876	0.00016
3.41	0.70910	0.70875	0.00016
3.37	0.70890	0.70875	0.00016
3.32	0.70868	0.70872	0.00016
3.28	0.70846	0.70871	0.00016

3.24	0.70838	0.70864	0.00024
3.19	0.70875	0.70868	0.00024
3.15	0.70881	0.70866	0.00024
3.11	0.70855	0.70868	0.00025
3.06	0.70914	0.70868	0.00025
3.02	0.70872	0.70870	0.00026
2.98	0.70912	0.70870	0.00026
2.93	0.70862	0.70864	0.00025
2.89	0.70859	0.70866	0.00025
2.85	0.70778	0.70866	0.00025
2.81	0.70874	0.70875	0.00015
2.76	0.70858	0.70873	0.00016
2.72	0.70900	0.70873	0.00016
2.68	0.70853	0.70876	0.00020
2.63	0.70932	0.70877	0.00019
2.59	0.70874	0.70873	0.00016
2.55	0.70856	0.70865	0.00023
2.50	0.70877	0.70867	0.00023
2.46	0.70856	0.70867	0.00023
2.42	0.70876	0.70872	0.00024
2.37	0.70852	0.70870	0.00024
2.33	0.70852	0.70868	0.00025
2.29	0.70932	0.70868	0.00025
2.24	0.70865	0.70858	0.00021
2.20	0.70894	0.70861	0.00022
2.16	0.70792	0.70859	0.00021
2.11	0.70879	0.70866	0.00015
2.07	0.70874	0.70860	0.00017
2.03	0.70906	0.70862	0.00019
1.99	0.70856	0.70857	0.00016
1.94	0.70830	0.70855	0.00016
1.90	0.70852	0.70862	0.00017
1.86	0.70834	0.70861	0.00017
1.81	0.70893	0.70864	0.00016
1.77	0.70875	0.70866	0.00019
1.73	0.70858	0.70868	0.00020
1.68	0.70823	0.70871	0.00020
1.64	0.70897	0.70872	0.00019
1.60	0.70852	0.70870	0.00018
1.55	0.70840	0.70870	0.00019
1.51	0.70896	0.70873	0.00017
1.47	0.70844	0.70868	0.00017
1.42	0.70859	0.70869	0.00017
1.38	0.70920	0.70864	0.00020
1.34	0.70896	0.70862	0.00018
1.29	0.70885	0.70857	0.00016
1.25	0.70833	0.70857	0.00016
1.21	0.70879	0.70862	0.00015
1.17	0.70846	0.70862	0.00016
1.12	0.70872	0.70863	0.00015

1.08	0.70845	0.70857	0.00018
1.04	0.70858	0.70858	0.00018
0.99	0.70811	0.70855	0.00019
0.95	0.70892	0.70860	0.00017
0.91	0.70852	0.70859	0.00016
0.86	0.70884	0.70856	0.00017
0.82	0.70880	0.70855	0.00017
0.78	0.70883	0.70856	0.00018
0.73	0.70851	0.70855	0.00017
0.69	0.70812	0.70852	0.00018
0.65	0.70862	0.70855	0.00016
0.60	0.70824	0.70850	0.00018
0.56	0.70859	0.70852	0.00017
0.52	0.70880	0.70851	0.00017
0.47	0.70822	0.70850	0.00017
0.43	0.70879	0.70856	0.00016
0.39	0.70889	0.70853	0.00016
0.35	0.70869	0.70849	0.00015
0.30	0.70823	0.70846	0.00015
0.26	0.70841	0.70849	0.00015
0.22	0.70811	0.70851	0.00016
0.17	0.70845	0.70861	0.00009
0.13	0.70853	0.70866	0.00008
0.09	0.70868	0.70873	0.00005
0.04	0.70878		

ARB 114.2.1 (M2)

Distance from cervix (mm)	$^{87}Sr/^{86}Sr$	10 point mov. average	2 SE on mov. average
30.75	0.70939	0.70966	0.00015
30.70	0.70986	0.70965	0.00016
30.66	0.70936	0.70960	0.00015
30.61	0.70954	0.70965	0.00015
30.57	0.70952	0.70967	0.00015
30.52	0.70969	0.70968	0.00015
30.48	0.70990	0.70970	0.00016
30.43	0.70992	0.70970	0.00015
30.39	0.70944	0.70965	0.00015
30.34	0.70995	0.70968	0.00015
30.30	0.70931	0.70967	0.00014
30.25	0.70942	0.70968	0.00013
30.16	0.70982	0.70967	0.00014
30.12	0.70973	0.70967	0.00014
30.07	0.70960	0.70965	0.00014
30.03	0.70995	0.70968	0.00015
29.98	0.70982	0.70969	0.00015
29.94	0.70944	0.70969	0.00015
29.89	0.70980	0.70969	0.00016
29.85	0.70985	0.70969	0.00016
29.80	0.70941	0.70969	0.00016
29.76	0.70929	0.70968	0.00016
29.71	0.70980	0.70976	0.00017
29.67	0.70956	0.70976	0.00017
29.62	0.70990	0.70978	0.00016
29.58	0.71002	0.70974	0.00017
29.53	0.70981	0.70965	0.00019
29.49	0.70942	0.70972	0.00026
29.44	0.70983	0.70970	0.00027
29.40	0.70981	0.70978	0.00034
29.35	0.70931	0.70977	0.00034
29.31	0.71017	0.70982	0.00033
29.26	0.70978	0.70986	0.00036
29.22	0.70972	0.70987	0.00036
29.17	0.70949	0.70994	0.00037
29.13	0.70917	0.71000	0.00036
29.08	0.71051	0.71012	0.00031
29.04	0.70917	0.71012	0.00031
28.99	0.71072	0.71024	0.00024
28.95	0.70964	0.71024	0.00023
28.90	0.70982	0.71031	0.00020
28.86	0.71062	0.71027	0.00025
28.81	0.70988	0.71034	0.00031
28.77	0.71041	0.71033	0.00031
28.72	0.71002	0.71029	0.00032
28.68	0.71036	0.71033	0.00031
28.63	0.71051	0.71035	0.00031

ARB 114.3.1 (M3)

Distance from cervix (mm)	$^{87}Sr/^{86}Sr$	10 point mov. average	2 SE on mov. average
42.59	0.70979	0.71017	0.00028
42.55	0.71023	0.71021	0.00027
42.50	0.71046	0.71022	0.00027
42.46	0.70952	0.71012	0.00031
42.42	0.71030	0.71021	0.00028
42.37	0.71088	0.71022	0.00028
42.33	0.70993	0.71023	0.00029
42.29	0.70979	0.71026	0.00028
42.24	0.71000	0.71023	0.00031
42.20	0.71080	0.71020	0.00032
42.16	0.71016	0.71013	0.00029
42.11	0.71041	0.71019	0.00032
42.07	0.70944	0.71004	0.00040
42.03	0.71040	0.71008	0.00038
41.98	0.71042	0.71018	0.00047
41.94	0.71094	0.71007	0.00049
41.90	0.71022	0.71002	0.00046
41.85	0.70950	0.71007	0.00048
41.81	0.70970	0.71009	0.00047
41.77	0.71015	0.71004	0.00050
41.72	0.71072	0.71005	0.00050
41.68	0.70891	0.71000	0.00048
41.64	0.70981	0.71011	0.00041
41.59	0.71142	0.71020	0.00042
41.55	0.70933	0.71004	0.00033
41.51	0.71043	0.71016	0.00030
41.46	0.71070	0.71016	0.00030
41.42	0.70971	0.71014	0.00029
41.38	0.70923	0.71022	0.00028
41.33	0.71022	0.71036	0.00019
41.29	0.71020	0.71036	0.00019
41.25	0.71003	0.71029	0.00025
41.20	0.71075	0.71033	0.00024
41.16	0.70979	0.71029	0.00022
41.12	0.71052	0.71034	0.00019
41.07	0.71045	0.71029	0.00020
41.03	0.71046	0.71024	0.00020
40.99	0.71057	0.71016	0.00022
40.94	0.71063	0.71016	0.00022
40.90	0.71016	0.71014	0.00020
40.86	0.70957	0.71021	0.00025
40.81	0.71044	0.71029	0.00021
40.77	0.71033	0.71028	0.00021
40.73	0.71023	0.71027	0.00021
40.68	0.71004	0.71031	0.00022
40.64	0.70999	0.71039	0.00023
40.59	0.70968	0.71037	0.00025

28.59	0.71046	0.71021	0.00038	40.55	0.71050	0.71036	0.00026
28.54	0.71068	0.71022	0.00038	40.51	0.71047	0.71036	0.00026
28.50	0.70950	0.71019	0.00037	40.46	0.71089	0.71026	0.00032
28.45	0.71120	0.71016	0.00041	40.42	0.71036	0.71022	0.00029
28.41	0.70982	0.71006	0.00033	40.38	0.71030	0.71018	0.00029
28.36	0.71010	0.71006	0.00033	40.33	0.71020	0.71017	0.00029
28.32	0.71033	0.71006	0.00033	40.29	0.71068	0.71020	0.00030
28.27	0.71055	0.71001	0.00033	40.25	0.71079	0.71025	0.00034
28.23	0.70928	0.70996	0.00031	40.20	0.70982	0.71030	0.00039
28.18	0.71052	0.70997	0.00030	40.16	0.70960	0.71031	0.00039
28.14	0.70988	0.70988	0.00028	40.12	0.71052	0.71032	0.00038
28.09	0.71077	0.70989	0.00029	40.07	0.70943	0.71029	0.00038
28.05	0.70917	0.70984	0.00023	40.03	0.71044	0.71039	0.00032
28.00	0.71016	0.70989	0.00018	39.99	0.71003	0.71039	0.00032
27.96	0.70984	0.70986	0.00017	39.94	0.71015	0.71042	0.00031
27.91	0.71011	0.70984	0.00017	39.90	0.71056	0.71045	0.00031
27.87	0.70984	0.70970	0.00027	39.86	0.71111	0.71043	0.00031
27.82	0.71006	0.70966	0.00028	39.81	0.71134	0.71039	0.00028
27.78	0.70936	0.70968	0.00029	39.77	0.70990	0.71028	0.00018
27.73	0.70956	0.70969	0.00028	39.73	0.70971	0.71030	0.00017
27.69	0.71004	0.70972	0.00028	39.68	0.71027	0.71028	0.00021
27.64	0.71022	0.70968	0.00027	39.64	0.71037	0.71025	0.00022
27.60	0.70975	0.70973	0.00032	39.60	0.71049	0.71016	0.00027
27.55	0.70987	0.70975	0.00032	39.55	0.71026	0.71008	0.00027
27.51	0.70961	0.70975	0.00032	39.51	0.71045	0.71006	0.00027
27.46	0.70871	0.70987	0.00039	39.47	0.71035	0.71002	0.00026
27.42	0.70946	0.70997	0.00029	39.42	0.71074	0.71004	0.00027
27.37	0.71019	0.71002	0.00027	39.38	0.71023	0.71003	0.00026
27.33	0.70954	0.71003	0.00027	39.34	0.71015	0.71002	0.00025
27.28	0.70986	0.71004	0.00027	39.29	0.70945	0.71007	0.00028
27.24	0.70963	0.71007	0.00026	39.25	0.71001	0.71017	0.00026
27.19	0.71065	0.71004	0.00029	39.21	0.70947	0.71025	0.00028
27.15	0.70995	0.70996	0.00026	39.16	0.70964	0.71036	0.00023
27.10	0.70987	0.71000	0.00027	39.12	0.71008	0.71036	0.00023
27.06	0.71084	0.71004	0.00027	39.08	0.71008	0.71043	0.00023
27.01	0.70976	0.70987	0.00027	39.03	0.71060	0.71040	0.00025
26.97	0.70994	0.70990	0.00028	38.99	0.71056	0.71032	0.00027
26.92	0.71031	0.70988	0.00028	38.95	0.71014	0.71028	0.00027
26.88	0.70960	0.70988	0.00028	38.90	0.71066	0.71030	0.00029
26.83	0.71011	0.70991	0.00027	38.86	0.71050	0.71036	0.00034
26.79	0.70934	0.70985	0.00028	38.82	0.71075	0.71030	0.00035
26.74	0.70988	0.70985	0.00027	38.77	0.71065	0.71028	0.00034
26.70	0.71038	0.70987	0.00028	38.73	0.70961	0.71020	0.00033
26.65	0.71028	0.70974	0.00029	38.69	0.71074	0.71031	0.00031
26.61	0.70908	0.70960	0.00030	38.64	0.70985	0.71030	0.00030
26.56	0.71010	0.70959	0.00031	38.60	0.70981	0.71038	0.00028
26.52	0.70972	0.70955	0.00029	38.56	0.71011	0.71041	0.00026
26.47	0.71030	0.70958	0.00031	38.47	0.71122	0.71023	0.00039
26.43	0.70986	0.70962	0.00035	38.43	0.71002	0.71013	0.00033
26.38	0.70953	0.70961	0.00035	38.38	0.71055	0.71017	0.00033

26.34	0.70935	0.70964	0.00035	38.34	0.70994	0.71016	0.00033
26.29	0.71005	0.70967	0.00034	38.30	0.71059	0.71016	0.00033
26.25	0.70910	0.70960	0.00033	38.25	0.71060	0.71010	0.00031
26.20	0.70892	0.70961	0.00033	38.21	0.71056	0.71010	0.00031
26.16	0.70900	0.70972	0.00029	38.17	0.71008	0.71010	0.00031
26.11	0.70966	0.70985	0.00027	38.12	0.70894	0.71014	0.00032
26.07	0.71007	0.70986	0.00027	38.08	0.70979	0.71024	0.00019
26.02	0.71063	0.70981	0.00027	38.04	0.71028	0.71035	0.00020
25.98	0.70981	0.70962	0.00028	37.99	0.71040	0.71044	0.00025
25.93	0.70978	0.70960	0.00028	37.95	0.71047	0.71045	0.00025
25.89	0.70966	0.70962	0.00029	37.91	0.70990	0.71037	0.00030
25.84	0.70942	0.70967	0.00031	37.86	0.70996	0.71046	0.00029
25.80	0.70921	0.70962	0.00033	37.82	0.71057	0.71059	0.00030
25.75	0.70995	0.70966	0.00032	37.78	0.71056	0.71058	0.00030
25.71	0.71036	0.70954	0.00037	37.73	0.71053	0.71060	0.00030
25.66	0.70972	0.70944	0.00032	37.69	0.70999	0.71057	0.00031
25.62	0.70958	0.70941	0.00031	37.65	0.71089	0.71058	0.00031
25.57	0.70868	0.70950	0.00037	37.60	0.71113	0.71058	0.00031
25.53	0.70964	0.70959	0.00033	37.56	0.71053	0.71044	0.00032
25.48	0.70996	0.70962	0.00033	37.52	0.70969	0.71026	0.00046
25.44	0.71013	0.70958	0.00032	37.47	0.71080	0.71039	0.00046
25.39	0.70897	0.70949	0.00030	37.43	0.71122	0.71041	0.00047
25.35	0.70964	0.70958	0.00029	37.39	0.71045	0.71032	0.00043
25.30	0.70869	0.70953	0.00030	37.34	0.71074	0.71034	0.00044
25.26	0.70940	0.70957	0.00025	37.30	0.71030	0.71032	0.00043
25.21	0.70944	0.70960	0.00025	37.26	0.71002	0.71031	0.00043
25.17	0.71043	0.70961	0.00025	37.21	0.71090	0.71037	0.00043
25.12	0.70962	0.70946	0.00020	37.17	0.70973	0.71028	0.00042
25.08	0.70992	0.70944	0.00020	37.13	0.70876	0.71034	0.00040
25.03	0.70954	0.70939	0.00017	37.08	0.71100	0.71057	0.00022
24.99	0.70929	0.70934	0.00018	37.04	0.71094	0.71048	0.00021
24.94	0.70980	0.70929	0.00021	37.00	0.71033	0.71038	0.00021
24.90	0.70916	0.70930	0.00022	36.95	0.71064	0.71049	0.00030
24.85	0.70913	0.70940	0.00028	36.91	0.71055	0.71045	0.00030
24.81	0.70965	0.70947	0.00028	36.87	0.71020	0.71051	0.00033
24.76	0.70955	0.70941	0.00029	36.82	0.71059	0.71047	0.00036
24.72	0.70892	0.70932	0.00033	36.78	0.71005	0.71043	0.00037
24.67	0.70944	0.70932	0.00033	36.73	0.71031	0.71052	0.00037
24.63	0.70942	0.70929	0.00033	36.69	0.71104	0.71054	0.00039
24.58	0.70899	0.70925	0.00033	36.65	0.71015	0.71048	0.00037
24.54	0.70883	0.70929	0.00032	36.60	0.70995	0.71039	0.00043
24.49	0.70991	0.70940	0.00033	36.56	0.71144	0.71046	0.00042
24.45	0.71019	0.70934	0.00031	36.52	0.71022	0.71042	0.00038
24.40	0.70980	0.70925	0.00025	36.47	0.71119	0.71044	0.00038
24.36	0.70903	0.70917	0.00022	36.43	0.70976	0.71039	0.00035
24.31	0.70865	0.70926	0.00027	36.39	0.71019	0.71052	0.00033
24.27	0.70890	0.70926	0.00027	36.34	0.71095	0.71055	0.00032
24.22	0.70916	0.70929	0.00026	36.26	0.71045	0.71060	0.00033
24.18	0.70906	0.70932	0.00026	36.21	0.70938	0.71055	0.00035
24.13	0.70932	0.70935	0.00025	36.17	0.71059	0.71066	0.00024

24.09	0.70996	0.70939	0.00026	36.13	0.71105	0.71068	0.00024
24.04	0.70931	0.70934	0.00023	36.08	0.71039	0.71060	0.00024
24.00	0.70933	0.70935	0.00023	36.04	0.71077	0.71057	0.00026
23.95	0.70899	0.70931	0.00025	36.00	0.71094	0.71054	0.00026
23.91	0.70995	0.70935	0.00024	35.95	0.71048	0.71044	0.00027
23.86	0.70865	0.70932	0.00021	35.91	0.71058	0.71051	0.00030
23.82	0.70914	0.70931	0.00022	35.87	0.71134	0.71042	0.00034
23.77	0.70944	0.70936	0.00023	35.82	0.71001	0.71036	0.00028
23.73	0.70940	0.70938	0.00023	35.78	0.71048	0.71046	0.00029
23.68	0.70970	0.70936	0.00023	35.74	0.71081	0.71048	0.00030
23.64	0.70945	0.70936	0.00023	35.69	0.71025	0.71051	0.00032
23.59	0.70946	0.70933	0.00024	35.65	0.71003	0.71051	0.00032
23.55	0.70889	0.70935	0.00025	35.61	0.71044	0.71061	0.00031
23.50	0.70946	0.70939	0.00023	35.56	0.70998	0.71058	0.00032
23.46	0.70958	0.70940	0.00023	35.52	0.71113	0.71068	0.00030
23.41	0.70856	0.70941	0.00023	35.48	0.70972	0.71067	0.00029
23.37	0.70967	0.70952	0.00014	35.43	0.71073	0.71083	0.00023
23.32	0.70963	0.70953	0.00014	35.39	0.71099	0.71087	0.00024
23.28	0.70921	0.70957	0.00018	35.35	0.71067	0.71087	0.00024
23.23	0.70970	0.70959	0.00017	35.30	0.71111	0.71082	0.00028
23.19	0.70912	0.70958	0.00016	35.26	0.71027	0.71071	0.00031
23.14	0.70966	0.70960	0.00015	35.22	0.71102	0.71071	0.00031
23.10	0.70929	0.70959	0.00014	35.17	0.71019	0.71066	0.00030
23.06	0.70961	0.70959	0.00014	35.13	0.71099	0.71066	0.00030
23.01	0.70968	0.70960	0.00015	35.09	0.71101	0.71059	0.00030
22.97	0.70964	0.70963	0.00016	35.04	0.71128	0.71057	0.00029
22.92	0.70973	0.70964	0.00016	35.00	0.71121	0.71039	0.00031
22.88	0.71005	0.70963	0.00016	34.96	0.71095	0.71025	0.00026
22.84	0.70938	0.70958	0.00013	34.91	0.71015	0.71027	0.00028
22.79	0.70964	0.70966	0.00017	34.87	0.71002	0.71039	0.00036
22.75	0.70928	0.70960	0.00021	34.83	0.71029	0.71046	0.00035
22.71	0.70960	0.70963	0.00020	34.78	0.71049	0.71045	0.00036
22.66	0.70930	0.70968	0.00022	34.74	0.71024	0.71048	0.00036
22.62	0.70973	0.70972	0.00021	34.70	0.71027	0.71057	0.00039
22.57	0.70996	0.70974	0.00021	34.65	0.71076	0.71059	0.00038
22.53	0.70975	0.70966	0.00023	34.61	0.70950	0.71051	0.00040
22.49	0.70956	0.70960	0.00026	34.57	0.70986	0.71057	0.00035
22.44	0.70957	0.70953	0.00029	34.52	0.71110	0.71057	0.00035
22.40	0.71018	0.70955	0.00030	34.48	0.71139	0.71053	0.00033
22.35	0.70906	0.70952	0.00027	34.44	0.71072	0.71040	0.00028
22.31	0.70954	0.70961	0.00026	34.39	0.71017	0.71034	0.00027
22.27	0.71016	0.70961	0.00026	34.35	0.71075	0.71040	0.00028
22.22	0.70970	0.70957	0.00023	34.31	0.71121	0.71034	0.00028
22.18	0.70994	0.70958	0.00024	34.26	0.71041	0.71025	0.00020
22.13	0.70917	0.70955	0.00023	34.22	0.71000	0.71023	0.00020
22.09	0.70913	0.70961	0.00021	34.18	0.71007	0.71033	0.00023
22.05	0.70889	0.70976	0.00027	34.13	0.70985	0.71029	0.00025
22.00	0.70974	0.70982	0.00020	34.09	0.71072	0.71043	0.00029
21.96	0.70986	0.70984	0.00020	34.05	0.71007	0.71039	0.00028
21.91	0.70996	0.70979	0.00022	34.00	0.71013	0.71042	0.00027

21.87	0.70959	0.70978	0.00022
21.83	0.70968	0.70974	0.00025
21.78	0.70983	0.70970	0.00026
21.74	0.70965	0.70963	0.00029
21.69	0.70979	0.70964	0.00030
21.65	0.71062	0.70962	0.00029
21.61	0.70948	0.70955	0.00021
21.56	0.70994	0.70953	0.00021
21.52	0.70935	0.70950	0.00019
21.48	0.70989	0.70954	0.00020
21.43	0.70917	0.70955	0.00020
21.39	0.70932	0.70961	0.00019
21.34	0.70904	0.70960	0.00020
21.30	0.70984	0.70968	0.00016
21.26	0.70957	0.70971	0.00018
21.21	0.70989	0.70968	0.00021
21.17	0.70932	0.70963	0.00021
21.12	0.70961	0.70955	0.00031
21.08	0.70977	0.70956	0.00031
21.04	0.70997	0.70958	0.00031
20.99	0.70978	0.70961	0.00034
20.95	0.70919	0.70957	0.00034
20.90	0.70988	0.70955	0.00035
20.86	0.71012	0.70957	0.00036
20.82	0.70923	0.70951	0.00034
20.77	0.70943	0.70951	0.00034
20.73	0.70848	0.70955	0.00034
20.68	0.70980	0.70961	0.00027
20.64	0.70989	0.70954	0.00029
20.60	0.71030	0.70951	0.00028
20.55	0.70934	0.70946	0.00024
20.51	0.70907	0.70952	0.00025
20.47	0.71008	0.70954	0.00024
20.42	0.70948	0.70950	0.00021
20.38	0.70921	0.70945	0.00023
20.33	0.70986	0.70943	0.00024
20.29	0.70911	0.70943	0.00024
20.25	0.70906	0.70944	0.00023
20.20	0.70957	0.70949	0.00021
20.16	0.70988	0.70948	0.00021
20.11	0.70992	0.70949	0.00022
20.07	0.70926	0.70942	0.00020
20.03	0.70965	0.70948	0.00021
19.98	0.70903	0.70944	0.00022
19.94	0.70901	0.70946	0.00020
19.89	0.70979	0.70953	0.00017
19.85	0.70928	0.70956	0.00020
19.81	0.70948	0.70969	0.00028
19.76	0.70953	0.70959	0.00036
19.72	0.70994	0.70959	0.00036

33.96	0.71084	0.71050	0.00028
33.92	0.71015	0.71042	0.00027
33.87	0.71029	0.71043	0.00027
33.83	0.71024	0.71043	0.00027
33.79	0.71090	0.71040	0.00029
33.74	0.70974	0.71037	0.00028
33.70	0.71119	0.71040	0.00026
33.66	0.71033	0.71032	0.00019
33.61	0.71045	0.71029	0.00020
33.57	0.71085	0.71024	0.00020
33.53	0.71011	0.71019	0.00016
33.48	0.71020	0.71020	0.00016
33.44	0.71032	0.71018	0.00016
33.40	0.70989	0.71014	0.00017
33.35	0.71066	0.71020	0.00017
33.31	0.70996	0.71024	0.00023
33.27	0.71041	0.71021	0.00024
33.22	0.71004	0.71026	0.00028
33.18	0.70995	0.71028	0.00027
33.14	0.71037	0.71033	0.00027
33.09	0.71020	0.71035	0.00027
33.05	0.71000	0.71036	0.00027
33.01	0.70988	0.71033	0.00029
32.96	0.71051	0.71042	0.00028
32.92	0.71104	0.71039	0.00028
32.87	0.70973	0.71027	0.00026
32.83	0.71091	0.71028	0.00025
32.79	0.71023	0.71026	0.00023
32.74	0.71048	0.71038	0.00033
32.70	0.71053	0.71035	0.00033
32.66	0.71029	0.71027	0.00035
32.61	0.70972	0.71029	0.00035
32.57	0.71073	0.71039	0.00033
32.53	0.71021	0.71038	0.00033
32.48	0.70984	0.71049	0.00038
32.44	0.70984	0.71061	0.00036
32.40	0.71073	0.71060	0.00037
32.35	0.71144	0.71049	0.00041
32.31	0.71015	0.71033	0.00037
32.27	0.70979	0.71035	0.00037
32.22	0.71040	0.71030	0.00042
32.18	0.71074	0.71043	0.00050
32.14	0.71062	0.71039	0.00050
32.09	0.71133	0.71026	0.00054
32.05	0.71102	0.71014	0.00048
32.01	0.70977	0.71007	0.00044
31.96	0.70965	0.71011	0.00044
31.92	0.70978	0.71021	0.00044
31.88	0.71042	0.71025	0.00043
31.83	0.70925	0.71032	0.00046

19.67	0.70922	0.70954	0.00036	31.79	0.71167	0.71042	0.00040
19.63	0.70987	0.70963	0.00037	31.75	0.71040	0.71031	0.00030
19.59	0.70922	0.70961	0.00036	31.70	0.70932	0.71027	0.00030
19.54	0.70929	0.70961	0.00036	31.66	0.71010	0.71027	0.00031
19.50	0.70963	0.70962	0.00036	31.62	0.71031	0.71035	0.00033
19.46	0.71010	0.70960	0.00036	31.57	0.71017	0.71027	0.00038
19.41	0.71058	0.70957	0.00035	31.53	0.71068	0.71027	0.00038
19.37	0.70851	0.70944	0.00027	31.49	0.71022	0.71013	0.00041
19.32	0.70953	0.70955	0.00017	31.44	0.71112	0.71016	0.00042
19.28	0.70944	0.70957	0.00018	31.40	0.71021	0.71004	0.00036
19.24	0.71016	0.70958	0.00017	31.36	0.71062	0.71004	0.00036
19.19	0.70963	0.70955	0.00014	31.31	0.71000	0.71000	0.00034
19.15	0.70923	0.70953	0.00014	31.27	0.70925	0.71007	0.00037
19.10	0.70937	0.70961	0.00014	31.23	0.71092	0.71022	0.00034
19.06	0.70946	0.70960	0.00015	31.18	0.70948	0.71022	0.00034
19.02	0.70981	0.70971	0.00025	31.14	0.71017	0.71020	0.00036
18.97	0.70930	0.70976	0.00027	31.10	0.70929	0.71031	0.00042
18.93	0.70958	0.70978	0.00025	31.05	0.71056	0.71047	0.00036
18.88	0.70972	0.70972	0.00030	31.01	0.70991	0.71040	0.00038
18.84	0.70956	0.70963	0.00036	30.97	0.71022	0.71044	0.00037
18.80	0.70989	0.70966	0.00036	30.92	0.71024	0.71049	0.00037
18.75	0.70941	0.70960	0.00037	30.88	0.71063	0.71039	0.00044
18.71	0.70995	0.70962	0.00036	30.84	0.71073	0.71029	0.00046
18.66	0.70931	0.70954	0.00036	30.79	0.71095	0.71030	0.00046
18.62	0.71060	0.70946	0.00042	30.75	0.70930	0.71021	0.00044
18.58	0.71027	0.70930	0.00034	30.71	0.71131	0.71039	0.00042
18.53	0.70954	0.70920	0.00026	30.66	0.71084	0.71030	0.00037
18.49	0.70899	0.70922	0.00028	30.62	0.70988	0.71021	0.00036
18.45	0.70873	0.70922	0.00027	30.58	0.71029	0.71023	0.00035
18.40	0.70988	0.70929	0.00025	30.53	0.71070	0.71019	0.00036
18.36	0.70930	0.70930	0.00026	30.49	0.70926	0.71011	0.00034
18.31	0.70963	0.70932	0.00027	30.45	0.70967	0.71025	0.00030
18.27	0.70919	0.70937	0.00030	30.40	0.71080	0.71033	0.00027
18.23	0.70849	0.70939	0.00030	30.36	0.71001	0.71029	0.00025
18.18	0.70900	0.70943	0.00025	30.32	0.71109	0.71038	0.00028
18.14	0.70924	0.70952	0.00025	30.27	0.71046	0.71024	0.00026
18.09	0.70971	0.70960	0.00027	30.23	0.70993	0.71021	0.00026
18.05	0.70906	0.70955	0.00028	30.19	0.71008	0.71023	0.00025
18.01	0.70937	0.70959	0.00026	30.14	0.70992	0.71020	0.00026
17.96	0.70998	0.70966	0.00027	30.10	0.70988	0.71026	0.00026
17.92	0.70958	0.70963	0.00028	30.06	0.71069	0.71031	0.00025
17.87	0.71006	0.70965	0.00028	30.01	0.71039	0.71030	0.00025
17.83	0.70941	0.70963	0.00027	29.97	0.71044	0.71027	0.00025
17.79	0.70889	0.70964	0.00027	29.93	0.71096	0.71018	0.00029
17.74	0.70989	0.70974	0.00020	29.88	0.70964	0.71011	0.00023
17.70	0.71009	0.70965	0.00025	29.84	0.71017	0.71026	0.00029
17.65	0.70921	0.70956	0.00023	29.80	0.71016	0.71028	0.00029
17.61	0.70939	0.70966	0.00024	29.75	0.70979	0.71027	0.00029
17.57	0.71012	0.70968	0.00024	29.71	0.71050	0.71028	0.00028
17.52	0.70983	0.70957	0.00023	29.67	0.71032	0.71020	0.00030

17.48	0.70984	0.70950	0.00023	29.62	0.71065	0.71015	0.00031
17.44	0.70947	0.70936	0.00029	29.58	0.71011	0.71012	0.00029
17.35	0.70985	0.70934	0.00029	29.54	0.70951	0.71012	0.00029
17.30	0.70902	0.70936	0.00032	29.49	0.71023	0.71013	0.00028
17.26	0.70930	0.70939	0.00031	29.45	0.71117	0.71012	0.00028
17.22	0.71012	0.70944	0.00032	29.41	0.71037	0.71007	0.00020
17.17	0.70959	0.70932	0.00029	29.36	0.71007	0.70998	0.00021
17.13	0.70958	0.70932	0.00029	29.32	0.70987	0.71003	0.00024
17.08	0.70909	0.70926	0.00029	29.28	0.70971	0.71003	0.00024
17.04	0.70911	0.70930	0.00028	29.23	0.70981	0.71006	0.00023
17.00	0.70850	0.70931	0.00028	29.19	0.71030	0.71006	0.00023
16.95	0.70920	0.70940	0.00022	29.15	0.71010	0.71013	0.00030
16.91	0.71012	0.70942	0.00021	29.10	0.70969	0.71009	0.00031
16.86	0.70934	0.70935	0.00014	29.06	0.71008	0.71016	0.00030
16.82	0.70976	0.70941	0.00018	29.01	0.71067	0.71019	0.00030
16.78	0.70896	0.70937	0.00017	28.97	0.70955	0.71015	0.00029
16.73	0.70951	0.70942	0.00014	28.93	0.71054	0.71024	0.00026
16.69	0.70904	0.70942	0.00014	28.88	0.70988	0.71022	0.00025
16.64	0.70944	0.70947	0.00011	28.84	0.71001	0.71030	0.00025
16.60	0.70927	0.70947	0.00012	28.80	0.70977	0.71033	0.00024
16.56	0.70940	0.70955	0.00016	28.75	0.71100	0.71044	0.00023
16.51	0.70936	0.70960	0.00017	28.71	0.70970	0.71035	0.00020
16.47	0.70946	0.70960	0.00018	28.67	0.71041	0.71045	0.00015
16.42	0.70991	0.70963	0.00018	28.62	0.71042	0.71059	0.00031
16.38	0.70931	0.70965	0.00020	28.58	0.71023	0.71064	0.00032
16.34	0.70949	0.70966	0.00020	28.54	0.71044	0.71069	0.00031
16.29	0.70949	0.70964	0.00021	28.49	0.71033	0.71066	0.00032
16.25	0.70961	0.70962	0.00022	28.45	0.71068	0.71064	0.00033
16.21	0.70938	0.70965	0.00023	28.41	0.71035	0.71061	0.00034
16.16	0.71007	0.70965	0.00022	28.36	0.71087	0.71064	0.00033
16.12	0.70996	0.70958	0.00021	28.32	0.71007	0.71058	0.00034
16.07	0.70930	0.70950	0.00020	28.28	0.71069	0.71067	0.00033
16.03	0.70976	0.70953	0.00020	28.23	0.71184	0.71060	0.00035
15.99	0.71018	0.70949	0.00019	28.19	0.71094	0.71042	0.00024
15.94	0.70935	0.70944	0.00013	28.15	0.71067	0.71031	0.00024
15.90	0.70929	0.70946	0.00013	28.10	0.71017	0.71032	0.00025
15.85	0.70931	0.70937	0.00023	28.06	0.71012	0.71043	0.00031
15.81	0.70988	0.70942	0.00024	28.02	0.71034	0.71053	0.00033
15.77	0.70945	0.70940	0.00023	27.97	0.71069	0.71059	0.00033
15.72	0.70936	0.70940	0.00023	27.93	0.71022	0.71050	0.00037
15.68	0.70919	0.70941	0.00023	27.89	0.71102	0.71057	0.00037
15.63	0.70952	0.70949	0.00026	27.84	0.71002	0.71052	0.00036
15.59	0.70939	0.70950	0.00026	27.80	0.70997	0.71062	0.00035
15.55	0.70972	0.70958	0.00029	27.76	0.70986	0.71066	0.00033
15.50	0.70946	0.70952	0.00031	27.71	0.71080	0.71063	0.00035
15.46	0.70848	0.70952	0.00031	27.67	0.71130	0.71059	0.00035
15.41	0.70972	0.70958	0.00023	27.63	0.71110	0.71055	0.00032
15.37	0.70974	0.70965	0.00028	27.58	0.71095	0.71044	0.00032
15.33	0.70942	0.70953	0.00035	27.54	0.70980	0.71039	0.00030
15.28	0.70943	0.70952	0.00036	27.50	0.71090	0.71045	0.00027

15.24	0.71004	0.70953	0.00035
15.20	0.70959	0.70963	0.00046
15.15	0.71022	0.70965	0.00046
15.11	0.70905	0.70957	0.00044
15.06	0.70952	0.70963	0.00043
15.02	0.70911	0.70964	0.00043
14.98	0.71037	0.70973	0.00042
14.93	0.70857	0.70967	0.00039
14.89	0.70931	0.70975	0.00032
14.84	0.70948	0.70979	0.00031
14.80	0.71105	0.70985	0.00030
14.76	0.70978	0.70973	0.00015
14.71	0.70946	0.70966	0.00019
14.67	0.70970	0.70969	0.00018
14.62	0.70960	0.70974	0.00020
14.58	0.71001	0.70975	0.00020
14.54	0.70977	0.70972	0.00019
14.49	0.70933	0.70972	0.00019
14.45	0.70974	0.70977	0.00017
14.40	0.71010	0.70978	0.00017
14.36	0.70982	0.70976	0.00016
14.32	0.70913	0.70975	0.00016
14.27	0.70973	0.70977	0.00013
14.23	0.71014	0.70973	0.00016
14.19	0.70975	0.70959	0.00022
14.14	0.70967	0.70953	0.00024
14.10	0.70980	0.70952	0.00024
14.05	0.70984	0.70958	0.00029
14.01	0.70987	0.70955	0.00029
13.97	0.70981	0.70949	0.00028
13.92	0.70979	0.70948	0.00028
13.88	0.70933	0.70946	0.00027
13.83	0.70931	0.70951	0.00028
13.79	0.70877	0.70957	0.00029
13.75	0.70911	0.70963	0.00023
13.70	0.70954	0.70962	0.00024
13.66	0.71041	0.70971	0.00028
13.61	0.70955	0.70963	0.00025
13.57	0.70933	0.70960	0.00026
13.53	0.70966	0.70959	0.00026
13.48	0.70958	0.70958	0.00026
13.44	0.70984	0.70956	0.00027
13.39	0.70996	0.70960	0.00030
13.35	0.70932	0.70956	0.00029
13.31	0.70905	0.70962	0.00029
13.26	0.71037	0.70976	0.00029
13.22	0.70929	0.70968	0.00023
13.18	0.70927	0.70977	0.00023
13.13	0.70951	0.70986	0.00021
13.04	0.70939	0.70993	0.00022

27.45	0.71049	0.71040	0.00025
27.41	0.71104	0.71039	0.00025
27.37	0.71031	0.71033	0.00020
27.32	0.70965	0.71037	0.00022
27.28	0.71040	0.71043	0.00016
27.24	0.71084	0.71041	0.00017
27.19	0.70997	0.71032	0.00016
27.15	0.71049	0.71033	0.00015
27.11	0.71036	0.71037	0.00018
27.06	0.71049	0.71037	0.00018
27.02	0.71033	0.71039	0.00019
26.98	0.71041	0.71037	0.00020
26.93	0.71078	0.71041	0.00022
26.89	0.71026	0.71041	0.00023
26.85	0.71016	0.71036	0.00026
26.80	0.70994	0.71038	0.00026
26.76	0.71010	0.71048	0.00027
26.72	0.71088	0.71057	0.00027
26.67	0.71039	0.71056	0.00026
26.63	0.71066	0.71055	0.00026
26.59	0.71010	0.71056	0.00027
26.54	0.71082	0.71061	0.00025
26.50	0.71084	0.71059	0.00024
26.46	0.70975	0.71058	0.00024
26.41	0.71028	0.71066	0.00015
26.37	0.71103	0.71071	0.00013
26.33	0.71096	0.71071	0.00012
26.28	0.71075	0.71072	0.00013
26.24	0.71033	0.71072	0.00013
26.20	0.71076	0.71068	0.00019
26.15	0.71053	0.71062	0.00022
26.11	0.71071	0.71065	0.00022
26.07	0.71067	0.71063	0.00022
26.02	0.71056	0.71058	0.00024
25.98	0.71082	0.71057	0.00024
25.94	0.71097	0.71050	0.00024
25.89	0.71106	0.71046	0.00022
25.85	0.71075	0.71040	0.00018
25.81	0.70998	0.71037	0.00016
25.76	0.71012	0.71037	0.00017
25.72	0.71087	0.71047	0.00022
25.68	0.71047	0.71045	0.00020
25.63	0.71024	0.71043	0.00021
25.59	0.71043	0.71048	0.00021
25.55	0.71017	0.71052	0.00022
25.50	0.71052	0.71052	0.00022
25.46	0.71048	0.71055	0.00023
25.42	0.71044	0.71054	0.00023
25.37	0.70993	0.71056	0.00023
25.33	0.71116	0.71058	0.00020

13.00	0.71026	0.71001	0.00018
12.96	0.70954	0.70998	0.00017
12.91	0.70988	0.70996	0.00019
12.87	0.71029	0.70999	0.00019
12.82	0.70977	0.70993	0.00019
12.78	0.70961	0.70997	0.00019
12.74	0.71014	0.71001	0.00018
12.69	0.71016	0.71001	0.00018
12.65	0.71031	0.70999	0.00017
12.60	0.71015	0.70997	0.00016
12.56	0.70996	0.70996	0.00015
12.52	0.70938	0.70992	0.00017
12.47	0.71009	0.71009	0.00025
12.43	0.70968	0.71010	0.00025
12.38	0.71021	0.71013	0.00023
12.34	0.71005	0.71014	0.00024
12.30	0.71006	0.71011	0.00025
12.25	0.71002	0.71008	0.00026
12.21	0.71007	0.71008	0.00026
12.16	0.71006	0.71009	0.00026
12.12	0.70962	0.71008	0.00026
12.08	0.71107	0.71006	0.00028
12.03	0.71018	0.70996	0.00016
11.99	0.70996	0.70998	0.00018
11.95	0.71035	0.71001	0.00019
11.90	0.70971	0.70997	0.00018
11.86	0.70978	0.70997	0.00017
11.81	0.71001	0.71010	0.00027
11.77	0.71011	0.71011	0.00027
11.73	0.71002	0.71010	0.00027
11.68	0.70945	0.71024	0.00037
11.64	0.71004	0.71032	0.00033
11.59	0.71042	0.71033	0.00032
11.55	0.71026	0.71030	0.00033
11.51	0.70988	0.71028	0.00033
11.46	0.70976	0.71021	0.00039
11.42	0.71103	0.71025	0.00038
11.37	0.71010	0.71010	0.00036
11.33	0.71008	0.71010	0.00036
11.29	0.71140	0.71010	0.00036
11.24	0.71020	0.70995	0.00022
11.20	0.71017	0.70991	0.00021
11.15	0.71010	0.70983	0.00023
11.11	0.71006	0.70989	0.00029
11.07	0.70918	0.71000	0.00039
11.02	0.71019	0.71013	0.00035
10.98	0.70950	0.71009	0.00036
10.94	0.71013	0.71021	0.00035
10.89	0.71010	0.71018	0.00035
10.85	0.70988	0.71015	0.00036

25.29	0.71064	0.71045	0.00021
25.24	0.71029	0.71057	0.00035
25.20	0.71079	0.71059	0.00034
25.15	0.71081	0.71077	0.00053
25.11	0.71019	0.71072	0.00054
25.07	0.71079	0.71073	0.00053
25.02	0.71036	0.71073	0.00053
24.98	0.71065	0.71075	0.00053
24.94	0.71017	0.71072	0.00053
24.89	0.70985	0.71070	0.00055
24.85	0.71184	0.71077	0.00051
24.81	0.71043	0.71072	0.00047
24.76	0.71259	0.71074	0.00047
24.72	0.71031	0.71059	0.00025
24.68	0.71035	0.71055	0.00029
24.63	0.71077	0.71055	0.00029
24.59	0.71058	0.71044	0.00033
24.55	0.71035	0.71044	0.00033
24.50	0.70991	0.71057	0.00041
24.46	0.71060	0.71062	0.00039
24.42	0.71127	0.71059	0.00039
24.37	0.71066	0.71055	0.00037
24.33	0.71112	0.71059	0.00038
24.29	0.70986	0.71044	0.00040
24.24	0.71036	0.71053	0.00038
24.20	0.70966	0.71052	0.00039
24.16	0.71056	0.71065	0.00035
24.11	0.71165	0.71060	0.00037
24.07	0.71044	0.71052	0.00029
24.03	0.71030	0.71057	0.00030
23.98	0.71091	0.71056	0.00030
23.94	0.71100	0.71050	0.00030
23.90	0.70964	0.71047	0.00028
23.85	0.71074	0.71055	0.00021
23.81	0.71025	0.71056	0.00022
23.77	0.71104	0.71057	0.00021
23.72	0.71005	0.71051	0.00018
23.68	0.71081	0.71051	0.00019
23.64	0.71095	0.71045	0.00018
23.59	0.71024	0.71045	0.00018
23.55	0.71031	0.71041	0.00021
23.51	0.71064	0.71045	0.00022
23.46	0.71048	0.71041	0.00022
23.42	0.71083	0.71038	0.00022
23.38	0.71039	0.71041	0.00025
23.33	0.71044	0.71030	0.00034
23.29	0.70998	0.71030	0.00034
23.25	0.71022	0.71036	0.00033
23.20	0.71093	0.71036	0.00033
23.16	0.70986	0.71034	0.00032

10.80	0.70979	0.71019	0.00036
10.76	0.70936	0.71018	0.00036
10.72	0.71072	0.71025	0.00032
10.67	0.71119	0.71018	0.00030
10.63	0.71046	0.71009	0.00021
10.58	0.70978	0.71007	0.00019
10.54	0.71066	0.71018	0.00023
10.50	0.70990	0.71015	0.00022
10.45	0.70972	0.71023	0.00023
10.41	0.71028	0.71027	0.00021
10.36	0.70973	0.71022	0.00023
10.32	0.71010	0.71021	0.00024
10.28	0.71001	0.71018	0.00025
10.23	0.71030	0.71024	0.00027
10.19	0.71024	0.71015	0.00031
10.14	0.71084	0.71012	0.00031
10.10	0.71041	0.71011	0.00030
10.06	0.71072	0.71007	0.00030
10.01	0.71003	0.70993	0.00029
9.97	0.70980	0.70987	0.00031
9.93	0.70965	0.70992	0.00032
9.88	0.70976	0.70994	0.00031
9.84	0.71064	0.71000	0.00031
9.79	0.70945	0.70995	0.00028
9.75	0.70991	0.70994	0.00029
9.71	0.71076	0.70998	0.00030
9.66	0.70999	0.70991	0.00025
9.62	0.70933	0.70993	0.00026
9.57	0.70945	0.71004	0.00023
9.53	0.71029	0.71011	0.00019
9.49	0.70987	0.71007	0.00019
9.44	0.71028	0.70993	0.00037
9.40	0.71020	0.70990	0.00036
9.35	0.70936	0.70990	0.00036
9.31	0.71032	0.70993	0.00034
9.27	0.71001	0.70989	0.00033
9.22	0.71024	0.70988	0.00033
9.18	0.71039	0.70981	0.00032
9.13	0.71015	0.70984	0.00035
9.09	0.70987	0.70985	0.00035
9.05	0.70852	0.70982	0.00035
9.00	0.70996	0.70996	0.00020
8.96	0.71015	0.70997	0.00020
8.92	0.70970	0.71001	0.00023
8.87	0.70996	0.71005	0.00022
8.83	0.70984	0.71003	0.00023
8.78	0.70958	0.71010	0.00025
8.74	0.71065	0.71019	0.00023
8.70	0.71024	0.71012	0.00022
8.65	0.70964	0.71010	0.00021

23.12	0.71071	0.71035	0.00031
23.07	0.71022	0.71032	0.00030
23.03	0.71027	0.71032	0.00030
22.99	0.71112	0.71036	0.00031
22.94	0.70929	0.71035	0.00030
22.90	0.71037	0.71046	0.00018
22.86	0.71060	0.71047	0.00018
22.81	0.71027	0.71040	0.00021
22.77	0.71069	0.71035	0.00024
22.73	0.70998	0.71025	0.00026
22.68	0.71035	0.71026	0.00026
22.64	0.71025	0.71024	0.00026
22.60	0.71065	0.71020	0.00027
22.55	0.71102	0.71023	0.00030
22.51	0.71045	0.71018	0.00025
22.47	0.71043	0.71026	0.00033
22.42	0.70994	0.71024	0.00033
22.38	0.70980	0.71026	0.00032
22.34	0.70968	0.71031	0.00031
22.29	0.71007	0.71032	0.00030
22.25	0.71016	0.71025	0.00035
22.21	0.70983	0.71036	0.00042
22.16	0.71098	0.71034	0.00043
22.12	0.71043	0.71028	0.00040
22.08	0.71126	0.71032	0.00042
22.03	0.71027	0.71022	0.00037
21.99	0.71015	0.71017	0.00038
21.95	0.71029	0.71018	0.00038
21.90	0.70978	0.71012	0.00039
21.86	0.70934	0.71011	0.00040
21.82	0.71134	0.71027	0.00039
21.77	0.70963	0.71019	0.00032
21.73	0.71029	0.71034	0.00033
21.69	0.71092	0.71041	0.00036
21.64	0.71019	0.71039	0.00035
21.60	0.70981	0.71043	0.00035
21.56	0.71026	0.71050	0.00032
21.51	0.70970	0.71052	0.00031
21.47	0.70963	0.71061	0.00026
21.43	0.71092	0.71072	0.00013
21.38	0.71060	0.71064	0.00018
21.34	0.71106	0.71065	0.00018
21.29	0.71102	0.71059	0.00016
21.25	0.71073	0.71053	0.00013
21.21	0.71054	0.71046	0.00016
21.16	0.71050	0.71040	0.00019
21.12	0.71048	0.71031	0.00024
21.08	0.71064	0.71024	0.00026
21.03	0.71075	0.71020	0.00024
20.99	0.71003	0.71009	0.00024

8.61	0.70993	0.71015	0.00019	20.95	0.71071	0.71011	0.00024
8.56	0.71000	0.71024	0.00023	20.90	0.71048	0.71004	0.00020
8.52	0.71055	0.71026	0.00022	20.86	0.71043	0.71005	0.00020
8.48	0.71011	0.71028	0.00025	20.82	0.71000	0.70998	0.00019
8.43	0.70974	0.71026	0.00026	20.77	0.70993	0.70996	0.00019
8.39	0.71056	0.71031	0.00023	20.73	0.70961	0.71004	0.00025
8.34	0.71053	0.71033	0.00025	20.69	0.70982	0.71006	0.00024
8.30	0.70990	0.71037	0.00027	20.64	0.71027	0.71004	0.00025
8.26	0.71001	0.71038	0.00027	20.60	0.70957	0.71001	0.00024
8.21	0.71018	0.71040	0.00026	20.56	0.71027	0.71006	0.00022
8.17	0.71086	0.71048	0.00028	20.51	0.71004	0.71010	0.00026
8.12	0.71011	0.71052	0.00031	20.47	0.71050	0.71008	0.00026
8.08	0.71084	0.71051	0.00032	20.43	0.70979	0.71006	0.00025
8.04	0.70986	0.71039	0.00035	20.38	0.70979	0.71013	0.00026
7.99	0.71024	0.71047	0.00033	20.34	0.71075	0.71016	0.00025
7.95	0.71078	0.71044	0.00036	20.30	0.70982	0.71010	0.00021
7.91	0.71094	0.71039	0.00035	20.25	0.70960	0.71013	0.00021
7.86	0.70997	0.71036	0.00033	20.21	0.70997	0.71016	0.00017
7.82	0.71020	0.71048	0.00036	20.17	0.71006	0.71012	0.00021
7.77	0.71103	0.71048	0.00036	20.12	0.71073	0.71014	0.00022
7.73	0.71121	0.71041	0.00034	20.08	0.70983	0.71010	0.00018
7.69	0.71001	0.71037	0.00030	20.04	0.71025	0.71009	0.00018
7.64	0.70964	0.71041	0.00030	19.99	0.71054	0.71011	0.00019
7.60	0.71073	0.71050	0.00026	19.95	0.71009	0.71004	0.00018
7.55	0.70984	0.71047	0.00027	19.91	0.71005	0.71005	0.00018
7.51	0.71032	0.71057	0.00023	19.86	0.71013	0.71005	0.00018
7.47	0.71064	0.71062	0.00024	19.82	0.70999	0.71005	0.00018
7.42	0.71118	0.71062	0.00028	19.78	0.70953	0.71011	0.00020
7.38	0.71018	0.71043	0.00018	19.73	0.71030	0.71014	0.00017
7.33	0.71038	0.71056	0.00016	19.69	0.71027	0.71012	0.00018
7.29	0.71074	0.71074	0.00020	19.65	0.70978	0.71004	0.00021
7.25	0.71069	0.71030	0.00024	19.60	0.71045	0.71003	0.00022
7.20	0.71014	0.71023	0.00023	19.56	0.70979	0.71004	0.00023
7.16	0.71123	0.71021	0.00027	19.52	0.71019	0.71009	0.00023
7.11	0.71031	0.71018	0.00018	19.47	0.71007	0.71000	0.00026
7.07	0.71011	0.71017	0.00022	19.43	0.71015	0.71005	0.00028
7.03	0.71026	0.71025	0.00023	19.39	0.71056	0.71010	0.00030
6.98	0.71018	0.71021	0.00029	19.34	0.70982	0.71001	0.00029
6.94	0.71008	0.71030	0.00032	19.26	0.70954	0.71002	0.00028
6.89	0.71009	0.71039	0.00032	19.21	0.70968	0.71001	0.00029
6.85	0.70992	0.71042	0.00032	19.17	0.71057	0.71011	0.00031
6.81	0.71000	0.71042	0.00032	19.13	0.71018	0.71000	0.00031
6.76	0.70990	0.71050	0.00032	19.08	0.70944	0.71000	0.00032
6.72	0.71094	0.71061	0.00029	19.04	0.71048	0.71004	0.00029
6.68	0.71025	0.71056	0.00031	19.00	0.71062	0.70999	0.00028
6.63	0.71086	0.71066	0.00034	18.95	0.70972	0.71003	0.00032
6.59	0.70985	0.71055	0.00036	18.91	0.70976	0.71007	0.00031
6.54	0.71115	0.71056	0.00033	18.87	0.71026	0.71003	0.00034
6.50	0.71098	0.71047	0.00031	18.82	0.70944	0.70997	0.00034
6.46	0.71038	0.71043	0.00030	18.78	0.71067	0.71001	0.00032

6.41	0.70990	0.71041	0.00031	18.74	0.70945	0.71002	0.00033
6.37	0.71082	0.71049	0.00028	18.69	0.71020	0.71000	0.00035
6.32	0.71097	0.71045	0.00028	18.65	0.70982	0.70999	0.00035
6.28	0.71042	0.71038	0.00031	18.61	0.70997	0.70997	0.00035
6.24	0.71125	0.71028	0.00037	18.56	0.71098	0.71003	0.00037
6.19	0.70979	0.71029	0.00033	18.52	0.71015	0.70987	0.00032
6.15	0.70995	0.71030	0.00033	18.48	0.70939	0.70978	0.00034
6.10	0.71023	0.71029	0.00033	18.43	0.70967	0.70981	0.00033
6.06	0.71062	0.71034	0.00034	18.39	0.70983	0.70981	0.00033
6.02	0.71011	0.71027	0.00034	18.35	0.71074	0.70991	0.00039
5.97	0.71077	0.71033	0.00036	18.30	0.70922	0.70982	0.00034
5.93	0.71043	0.71034	0.00035	18.26	0.71010	0.70989	0.00031
5.88	0.71021	0.71033	0.00036	18.22	0.70965	0.70985	0.00031
5.84	0.70947	0.71039	0.00036	18.17	0.71055	0.70986	0.00031
5.80	0.71136	0.71050	0.00031	18.13	0.70941	0.70971	0.00030
5.75	0.70982	0.71040	0.00026	18.09	0.70919	0.70975	0.00030
5.71	0.70992	0.71049	0.00022	18.04	0.70974	0.70978	0.00028
5.67	0.71072	0.71054	0.00021	18.00	0.70964	0.70981	0.00028
5.62	0.70991	0.71057	0.00021	17.96	0.71083	0.70985	0.00028
5.58	0.71064	0.71060	0.00017	17.91	0.70981	0.70975	0.00018
5.53	0.71096	0.71060	0.00018	17.87	0.70998	0.70969	0.00021
5.49	0.71025	0.71053	0.00016	17.83	0.70973	0.70961	0.00022
5.45	0.71081	0.71057	0.00015	17.78	0.70973	0.70965	0.00024
5.40	0.71063	0.71056	0.00024	17.74	0.70908	0.70975	0.00031
5.36	0.71033	0.71045	0.00025	17.70	0.70974	0.70983	0.00028
5.31	0.71075	0.71042	0.00025	17.65	0.70948	0.70981	0.00028
5.27	0.71044	0.71040	0.00027	17.61	0.71009	0.70979	0.00029
5.23	0.71099	0.71034	0.00037	17.57	0.71000	0.70984	0.00033
5.18	0.71024	0.71014	0.00035	17.52	0.70982	0.70965	0.00048
5.14	0.71059	0.71018	0.00035	17.48	0.70924	0.70963	0.00048
5.09	0.71030	0.71015	0.00034	17.44	0.70924	0.70963	0.00047
5.05	0.71065	0.71014	0.00038	17.39	0.71010	0.70968	0.00047
5.01	0.71067	0.71000	0.00036	17.35	0.71067	0.70969	0.00047
4.96	0.70955	0.70996	0.00034	17.30	0.70993	0.70958	0.00042
4.92	0.71000	0.71004	0.00033	17.26	0.70953	0.70957	0.00041
4.87	0.71058	0.71004	0.00033	17.22	0.70931	0.70961	0.00042
4.83	0.70980	0.71001	0.00035	17.17	0.71060	0.70952	0.00048
4.79	0.70900	0.71012	0.00035	17.13	0.70810	0.70937	0.00042
4.74	0.71070	0.71024	0.00026	17.09	0.70958	0.70951	0.00031
4.70	0.71023	0.71022	0.00026	17.04	0.70929	0.70953	0.00031
4.66	0.71025	0.71027	0.00027	17.00	0.70970	0.70957	0.00031
4.61	0.70926	0.71024	0.00028	16.96	0.71020	0.70952	0.00032
4.57	0.71023	0.71029	0.00021	16.91	0.70954	0.70950	0.00030
4.52	0.71033	0.71029	0.00022	16.87	0.70983	0.70940	0.00035
4.48	0.71005	0.71025	0.00022	16.83	0.70993	0.70927	0.00037
4.44	0.71021	0.71026	0.00022	16.78	0.70845	0.70917	0.00035
4.39	0.71091	0.71024	0.00025	16.74	0.70906	0.70920	0.00032
4.35	0.71025	0.71010	0.00020	16.70	0.70953	0.70923	0.00032
4.30	0.71052	0.71010	0.00021	16.65	0.70973	0.70929	0.00037
4.26	0.71071	0.71009	0.00019	16.61	0.70970	0.70925	0.00035

4.22	0.70992	0.71003	0.00016	16.57	0.70923	0.70921	0.00034
4.17	0.70979	0.71000	0.00017	16.52	0.70996	0.70921	0.00034
4.13	0.71016	0.71000	0.00016	16.48	0.70856	0.70919	0.00032
4.08	0.70997	0.70999	0.00018	16.44	0.70856	0.70926	0.00029
4.04	0.71011	0.70995	0.00018	16.39	0.70889	0.70934	0.00025
4.00	0.71002	0.70993	0.00018	16.35	0.70876	0.70935	0.00024
3.95	0.70959	0.70992	0.00018	16.31	0.70940	0.70949	0.00024
3.91	0.71019	0.70997	0.00017	16.26	0.71012	0.70947	0.00025
3.86	0.71042	0.70997	0.00017	16.22	0.70930	0.70935	0.00023
3.82	0.71014	0.70995	0.00017	16.18	0.70933	0.70934	0.00023
3.78	0.70957	0.70988	0.00016	16.13	0.70921	0.70945	0.00032
3.73	0.70984	0.70990	0.00016	16.09	0.70979	0.70946	0.00032
3.69	0.71004	0.70993	0.00016	16.05	0.70921	0.70948	0.00033
3.65	0.70954	0.70993	0.00016	16.00	0.70943	0.70946	0.00033
3.60	0.70998	0.70994	0.00017	15.96	0.70900	0.70937	0.00038
3.56	0.70992	0.70989	0.00017	15.92	0.71012	0.70942	0.00037
3.51	0.71004	0.70986	0.00023	15.87	0.70925	0.70943	0.00039
3.47	0.71018	0.70992	0.00024	15.83	0.70885	0.70950	0.00040
3.43	0.71022	0.70993	0.00027	15.79	0.70919	0.70956	0.00037
3.38	0.70947	0.70983	0.00027	15.74	0.71045	0.70950	0.00041
3.34	0.70981	0.70990	0.00026	15.70	0.70934	0.70936	0.00036
3.29	0.71015	0.70987	0.00028	15.66	0.70995	0.70939	0.00037
3.25	0.70998	0.70990	0.00028	15.61	0.70906	0.70942	0.00040
3.21	0.70962	0.70985	0.00029	15.57	0.70854	0.70953	0.00041
3.16	0.70951	0.70983	0.00029	15.53	0.70944	0.70962	0.00035
3.12	0.70963	0.70988	0.00035	15.48	0.71026	0.70961	0.00035
3.07	0.71066	0.71001	0.00037	15.44	0.70995	0.70951	0.00033
3.03	0.71024	0.70988	0.00035	15.40	0.70942	0.70932	0.00042
2.99	0.70923	0.70979	0.00035	15.35	0.70864	0.70941	0.00046
2.94	0.71018	0.70988	0.00033	15.31	0.70896	0.70948	0.00043
2.90	0.70954	0.70983	0.00034	15.27	0.70967	0.70945	0.00045
2.85	0.71036	0.70990	0.00034	15.22	0.71028	0.70947	0.00046
2.81	0.70958	0.70980	0.00033	15.18	0.71019	0.70940	0.00043
2.77	0.70941	0.70985	0.00039	15.14	0.70939	0.70932	0.00039
2.72	0.70995	0.70978	0.00042	15.09	0.70937	0.70926	0.00040
2.68	0.71098	0.70985	0.00042	15.05	0.70920	0.70931	0.00042
2.64	0.70936	0.70970	0.00035	15.01	0.70809	0.70931	0.00042
2.59	0.70927	0.70973	0.00035	14.96	0.71030	0.70949	0.00033
2.55	0.71018	0.70980	0.00034	14.92	0.70938	0.70938	0.00027
2.50	0.70965	0.70980	0.00037	14.88	0.70860	0.70944	0.00030
2.46	0.71029	0.70972	0.00040	14.83	0.70991	0.70952	0.00024
2.42	0.70936	0.70958	0.00039	14.79	0.70959	0.70943	0.00024
2.37	0.71005	0.70964	0.00039	14.75	0.70941	0.70948	0.00027
2.33	0.70870	0.70955	0.00038	14.70	0.70881	0.70944	0.00028
2.28	0.71062	0.70966	0.00037	14.66	0.70979	0.70944	0.00028
2.24	0.70953	0.70965	0.00033	14.62	0.70924	0.70941	0.00027
2.15	0.70960	0.70969	0.00035	14.57	0.70985	0.70941	0.00027
2.11	0.71003	0.70976	0.00035	14.53	0.70922	0.70936	0.00026
2.06	0.71015	0.70972	0.00036	14.49	0.71001	0.70939	0.00026
2.02	0.70892	0.70973	0.00037	14.44	0.70935	0.70937	0.00024

1.98	0.70886	0.70987	0.00032
1.93	0.70990	0.70999	0.00024
1.89	0.70917	0.70998	0.00023
1.84	0.70981	0.71006	0.00019
1.80	0.71049	0.71003	0.00019
1.76	0.70999	0.70995	0.00018
1.71	0.71028	0.70991	0.00022
1.67	0.70965	0.70994	0.00021
1.62	0.71027	0.70996	0.00033
1.58	0.71031	0.71007	0.00033
1.54	0.70998	0.71008	0.00033
1.49	0.70983	0.71009	0.00033
1.45	0.70993	0.71011	0.00035
1.41	0.70954	0.71019	0.00036
1.36	0.70975	0.71021	0.00034
1.32	0.70959	0.71024	0.00035
1.27	0.71056	0.71024	0.00045
1.23	0.70986	0.71037	0.00046
1.19	0.71133	0.71037	0.00047
1.14	0.71046	0.71034	0.00043
1.10	0.71000	0.71033	0.00042
1.05	0.71003	0.71036	0.00042
1.01	0.71078	0.71037	0.00042
0.97	0.70970	0.71036	0.00043
0.92	0.71004	0.71044	0.00041
0.88	0.70959	0.71045	0.00041
0.83	0.71188	0.71058	0.00036
0.79	0.70989	0.71039	0.00021
0.75	0.71102	0.71045	0.00018
0.70	0.71039	0.71036	0.00018
0.66	0.71026	0.71029	0.00021
0.61	0.71017	0.71036	0.00021
0.57	0.71067	0.71040	0.00024
0.53	0.71020	0.71029	0.00023
0.48	0.71073	0.71034	0.00024
0.44	0.71019	0.71034	0.00024
0.40	0.71046	0.71036	0.00025
0.35	0.71018	0.71035	0.00027
0.31	0.70977	0.71037	0.00029
0.26	0.71086	0.71047	0.00026
0.22	0.71054	0.71039	0.00025
0.18	0.70977	0.71036	0.00029
0.13	0.71025	0.71055	0.00017
0.09	0.71062	0.71070	0.00007
0.04	0.71079		

14.40	0.70905	0.70940	0.00024
14.36	0.71006	0.70938	0.00026
14.31	0.70904	0.70928	0.00022
14.27	0.70879	0.70938	0.00026
14.23	0.70946	0.70954	0.00029
14.18	0.70925	0.70956	0.00029
14.14	0.70933	0.70963	0.00029
14.10	0.70959	0.70975	0.00033
14.05	0.70978	0.70971	0.00034
14.01	0.70963	0.70974	0.00035
13.97	0.70886	0.70967	0.00039
13.92	0.70904	0.70967	0.00039
13.88	0.71004	0.70965	0.00041
13.84	0.71039	0.70956	0.00041
13.79	0.70966	0.70945	0.00037
13.75	0.70997	0.70947	0.00037
13.71	0.71050	0.70944	0.00036
13.66	0.70925	0.70949	0.00044
13.62	0.71009	0.70952	0.00043
13.58	0.70895	0.70947	0.00041
13.53	0.70882	0.70963	0.00045
13.49	0.70880	0.70972	0.00042
13.44	0.70919	0.70986	0.00037
13.40	0.70930	0.70988	0.00035
13.36	0.70983	0.70990	0.00034
13.31	0.70965	0.70987	0.00035
13.27	0.71102	0.70987	0.00035
13.23	0.70957	0.70965	0.00029
13.18	0.70953	0.70960	0.00032
13.14	0.71063	0.70950	0.00039
13.10	0.70963	0.70946	0.00034
13.05	0.71020	0.70944	0.00034
13.01	0.70943	0.70941	0.00031
12.97	0.70950	0.70941	0.00031
12.92	0.70948	0.70941	0.00031
12.88	0.70965	0.70950	0.00037
12.84	0.70889	0.70949	0.00037
12.79	0.70902	0.70956	0.00034
12.75	0.70853	0.70965	0.00033
12.71	0.71024	0.70974	0.00023
12.66	0.70950	0.70973	0.00022
12.62	0.70984	0.70977	0.00021
12.58	0.70948	0.70975	0.00021
12.53	0.70942	0.70974	0.00022
12.49	0.71041	0.70971	0.00024
12.45	0.70952	0.70961	0.00019
12.40	0.70959	0.70964	0.00020
12.36	0.70999	0.70968	0.00020
12.32	0.70942	0.70970	0.00022
12.27	0.71012	0.70973	0.00022

12.23	0.70990	0.70963	0.00022
12.19	0.70965	0.70963	0.00022
12.14	0.70933	0.70961	0.00022
12.10	0.70917	0.70966	0.00022
12.06	0.70942	0.70972	0.00019
12.01	0.70983	0.70971	0.00020
11.97	0.70992	0.70977	0.00025
11.93	0.71023	0.70970	0.00027
11.88	0.70972	0.70963	0.00025
11.84	0.70915	0.70970	0.00029
11.80	0.70984	0.70982	0.00029
11.75	0.70952	0.70978	0.00030
11.71	0.70984	0.70981	0.00030
11.67	0.70978	0.70981	0.00030
11.62	0.70928	0.70972	0.00034
11.58	0.71047	0.70972	0.00035
11.54	0.70918	0.70954	0.00036
11.49	0.70955	0.70961	0.00036
11.45	0.71037	0.70949	0.00044
11.41	0.71038	0.70927	0.00046
11.36	0.70942	0.70914	0.00039
11.32	0.70983	0.70913	0.00038
11.28	0.70981	0.70909	0.00036
11.23	0.70896	0.70904	0.00032
11.19	0.70920	0.70904	0.00032
11.15	0.70866	0.70896	0.00034
11.10	0.70991	0.70901	0.00034
11.06	0.70832	0.70901	0.00033
11.02	0.70822	0.70916	0.00032
10.97	0.70912	0.70925	0.00025
10.93	0.70930	0.70923	0.00026
10.89	0.70937	0.70931	0.00030
10.84	0.70930	0.70933	0.00031
10.80	0.70896	0.70935	0.00031
10.76	0.70846	0.70937	0.00031
10.71	0.70917	0.70949	0.00023
10.67	0.70984	0.70952	0.00022
10.63	0.70981	0.70953	0.00022
10.58	0.70918	0.70950	0.00022
10.54	0.70893	0.70951	0.00021
10.50	0.71003	0.70963	0.00019
10.45	0.70957	0.70957	0.00017
10.41	0.70957	0.70963	0.00021
10.37	0.70911	0.70967	0.00022
10.32	0.70969	0.70972	0.00018
10.28	0.70951	0.70978	0.00021
10.24	0.70985	0.70972	0.00026
10.19	0.70953	0.70969	0.00026
10.15	0.70933	0.70965	0.00029
10.11	0.71006	0.70964	0.00029

10.06	0.70943	0.70954	0.00030
10.02	0.71019	0.70960	0.00031
9.98	0.70995	0.70957	0.00030
9.93	0.70962	0.70953	0.00028
9.89	0.71027	0.70957	0.00030
9.85	0.70896	0.70942	0.00029
9.80	0.70956	0.70954	0.00031
9.76	0.70913	0.70955	0.00031
9.72	0.70921	0.70957	0.00030
9.67	0.70904	0.70958	0.00029
9.63	0.71001	0.70967	0.00027
9.58	0.70996	0.70965	0.00026
9.54	0.70954	0.70957	0.00026
9.50	0.70998	0.70967	0.00033
9.45	0.70877	0.70972	0.00036
9.41	0.71018	0.70986	0.00030
9.37	0.70963	0.70988	0.00031
9.32	0.70937	0.70987	0.00031
9.28	0.70935	0.71005	0.00039
9.24	0.70994	0.71012	0.00036
9.19	0.70974	0.71012	0.00036
9.15	0.70921	0.71010	0.00036
9.11	0.71056	0.71020	0.00031
9.06	0.71040	0.71014	0.00030
9.02	0.71017	0.71012	0.00030
8.98	0.71040	0.71001	0.00035
8.93	0.70955	0.70996	0.00034
8.89	0.71119	0.71002	0.00033
8.85	0.71005	0.70993	0.00022
8.80	0.70990	0.70987	0.00024
8.76	0.70959	0.70991	0.00026
8.72	0.71024	0.70994	0.00026
8.67	0.70987	0.70998	0.00029
8.63	0.71020	0.71002	0.00029
8.59	0.70913	0.70999	0.00029
8.54	0.70992	0.71011	0.00021
8.50	0.71014	0.71008	0.00023
8.46	0.71026	0.71002	0.00025
8.41	0.70942	0.70988	0.00032
8.37	0.71032	0.70989	0.00032
8.28	0.71056	0.70996	0.00033
8.24	0.71026	0.70989	0.00031
8.20	0.70987	0.70979	0.00031
8.15	0.71027	0.70975	0.00032
8.11	0.70966	0.70968	0.00030
8.07	0.70955	0.70966	0.00031
8.02	0.70903	0.70966	0.00031
7.98	0.70945	0.70979	0.00029
7.94	0.71042	0.71000	0.00045
7.89	0.71055	0.70998	0.00045

7.85	0.70982	0.70983	0.00047
7.81	0.70932	0.70986	0.00047
7.76	0.70944	0.70994	0.00046
7.72	0.70959	0.70997	0.00045
7.68	0.70938	0.70993	0.00047
7.63	0.70960	0.70995	0.00046
7.59	0.71028	0.70996	0.00045
7.55	0.71160	0.70985	0.00047
7.50	0.71025	0.70968	0.00027
7.46	0.70901	0.70960	0.00025
7.42	0.71016	0.70967	0.00021
7.37	0.71005	0.70970	0.00025
7.33	0.70974	0.70966	0.00024
7.29	0.70919	0.70972	0.00028
7.24	0.70965	0.70981	0.00026
7.20	0.70971	0.70991	0.00030
7.16	0.70917	0.70988	0.00031
7.11	0.70990	0.70994	0.00027
7.07	0.70938	0.70996	0.00028
7.03	0.70973	0.70999	0.00025
6.98	0.71048	0.70997	0.00026
6.94	0.70959	0.70985	0.00028
6.90	0.71037	0.70990	0.00027
6.85	0.71015	0.70972	0.00035
6.81	0.71058	0.70972	0.00035
6.77	0.70942	0.70966	0.00030
6.72	0.70979	0.70973	0.00030
6.68	0.71007	0.70962	0.00036
6.64	0.70975	0.70957	0.00035
6.59	0.70949	0.70952	0.00035
6.55	0.70923	0.70959	0.00037
6.51	0.71009	0.70967	0.00037
6.46	0.70866	0.70969	0.00039
6.42	0.71015	0.70984	0.00032
6.38	0.70991	0.70975	0.00033
6.33	0.71011	0.70974	0.00032
6.29	0.70874	0.70972	0.00032
6.25	0.70959	0.70975	0.00027
6.20	0.70923	0.70982	0.00029
6.16	0.71015	0.70992	0.00027
6.12	0.71004	0.70990	0.00026
6.07	0.71034	0.70984	0.00028
6.03	0.71011	0.70974	0.00026
5.99	0.70930	0.70969	0.00025
5.94	0.70980	0.70975	0.00024
5.90	0.70988	0.70976	0.00024
5.86	0.70908	0.70974	0.00024
5.81	0.71026	0.70979	0.00020
5.77	0.71025	0.70974	0.00017
5.72	0.70991	0.70965	0.00014

5.68	0.70943	0.70960	0.00013
5.64	0.70940	0.70959	0.00014
5.59	0.70954	0.70964	0.00015
5.55	0.70991	0.70964	0.00015
5.51	0.70997	0.70971	0.00024
5.46	0.70966	0.70975	0.00027
5.42	0.70962	0.70983	0.00030
5.38	0.70968	0.70986	0.00030
5.33	0.70937	0.70986	0.00030
5.29	0.70943	0.70991	0.00028
5.25	0.70931	0.70996	0.00026
5.20	0.70992	0.71001	0.00022
5.16	0.70956	0.71002	0.00022
5.12	0.71062	0.71011	0.00021
5.07	0.71032	0.71012	0.00023
5.03	0.71046	0.71012	0.00022
4.99	0.70994	0.71004	0.00023
4.94	0.70967	0.70999	0.00025
4.90	0.70988	0.71004	0.00024
4.86	0.70989	0.71002	0.00025
4.81	0.70985	0.71009	0.00027
4.77	0.70998	0.71013	0.00027
4.73	0.71046	0.71015	0.00026
4.68	0.71078	0.71004	0.00029
4.64	0.71027	0.70993	0.00026
4.60	0.70969	0.70983	0.00027
4.55	0.70946	0.70993	0.00032
4.51	0.71012	0.70996	0.00030
4.47	0.70971	0.70991	0.00031
4.42	0.71057	0.70999	0.00033
4.38	0.71028	0.70991	0.00031
4.34	0.71017	0.70982	0.00031
4.29	0.70938	0.70978	0.00030
4.25	0.70960	0.70982	0.00029
4.21	0.70927	0.70982	0.00029
4.16	0.71069	0.70985	0.00027
4.12	0.70985	0.70979	0.00020
4.08	0.70959	0.70975	0.00021
4.03	0.71053	0.70979	0.00021
3.99	0.70977	0.70968	0.00015
3.95	0.70936	0.70965	0.00015
3.90	0.70973	0.70964	0.00016
3.86	0.70978	0.70965	0.00016
3.82	0.70959	0.70966	0.00017
3.77	0.70960	0.70962	0.00018
3.73	0.71009	0.70963	0.00018
3.69	0.70946	0.70956	0.00015
3.64	0.70996	0.70964	0.00020
3.60	0.70944	0.70954	0.00023
3.56	0.70946	0.70952	0.00023

3.51	0.70927	0.70949	0.00024
3.47	0.70986	0.70953	0.00024
3.43	0.70980	0.70957	0.00027
3.38	0.70928	0.70960	0.00029
3.34	0.70965	0.70961	0.00029
3.30	0.70941	0.70963	0.00029
3.25	0.71023	0.70968	0.00029
3.21	0.70897	0.70967	0.00028
3.17	0.70929	0.70972	0.00025
3.12	0.70915	0.70979	0.00024
3.08	0.70966	0.70986	0.00019
3.04	0.71022	0.70988	0.00018
2.99	0.71016	0.70980	0.00018
2.95	0.70939	0.70978	0.00017
2.91	0.70981	0.70985	0.00015
2.86	0.70997	0.70985	0.00015
2.82	0.71010	0.70979	0.00018
2.78	0.70943	0.70980	0.00019
2.73	0.71006	0.70983	0.00018
2.69	0.70985	0.70981	0.00017
2.65	0.70981	0.70984	0.00018
2.60	0.70946	0.70980	0.00020
2.56	0.70994	0.70978	0.00021
2.52	0.71007	0.70974	0.00021
2.47	0.70985	0.70981	0.00029
2.43	0.70930	0.70981	0.00029
2.39	0.71022	0.70989	0.00027
2.34	0.70973	0.70983	0.00027
2.30	0.70987	0.70986	0.00027
2.26	0.71012	0.70985	0.00027
2.21	0.70939	0.70984	0.00026
2.17	0.70933	0.70990	0.00024
2.13	0.70955	0.70994	0.00021
2.08	0.71076	0.70999	0.00020
2.04	0.70985	0.70987	0.00012
2.00	0.71007	0.70982	0.00015
1.95	0.70964	0.70980	0.00014
1.91	0.71000	0.70976	0.00018
1.86	0.70977	0.70973	0.00018
1.82	0.71003	0.70966	0.00022
1.78	0.71002	0.70966	0.00022
1.73	0.70972	0.70954	0.00026
1.69	0.71004	0.70961	0.00032
1.65	0.70951	0.70953	0.00031
1.60	0.70944	0.70957	0.00032
1.56	0.70985	0.70961	0.00032
1.52	0.70920	0.70959	0.00032
1.47	0.70974	0.70963	0.00031
1.43	0.70905	0.70968	0.00033
1.39	0.71000	0.70973	0.00030

1.34	0.70884	0.70965	0.00031
1.30	0.71047	0.70970	0.00027
1.26	0.70922	0.70956	0.00023
1.21	0.70988	0.70962	0.00022
1.17	0.70988	0.70953	0.00024
1.13	0.70958	0.70948	0.00023
1.08	0.70968	0.70950	0.00024
1.04	0.71022	0.70948	0.00023
1.00	0.70951	0.70937	0.00017
0.95	0.70923	0.70931	0.00020
0.91	0.70930	0.70927	0.00022
0.87	0.70908	0.70930	0.00023
0.82	0.70980	0.70934	0.00023
0.78	0.70902	0.70927	0.00020
0.74	0.70938	0.70929	0.00020
0.69	0.70979	0.70928	0.00020
0.65	0.70947	0.70923	0.00016
0.61	0.70916	0.70919	0.00016
0.56	0.70884	0.70921	0.00016
0.52	0.70884	0.70924	0.00014
0.48	0.70960	0.70927	0.00011
0.43	0.70947	0.70926	0.00009
0.39	0.70917	0.70924	0.00009
0.35	0.70913	0.70925	0.00009
0.30	0.70934	0.70926	0.00009
0.26	0.70931	0.70925	0.00010
0.22	0.70905	0.70924	0.00011
0.17	0.70934	0.70928	0.00010
0.13	0.70911	0.70926	0.00012
0.09	0.70921	0.70934	0.00011
0.04	0.70947		

ARB 115.2.1 (M2)

Distance from cervix (mm)	$^{87}Sr/^{86}Sr$	10 point mov. average	2 SE on mov. average
12.29	0.71026	0.71032	0.00024
12.25	0.70997	0.71034	0.00025
12.21	0.70993	0.71042	0.00024
12.16	0.70957	0.71050	0.00022
12.12	0.71068	0.71063	0.00010
12.07	0.71067	0.71062	0.00010
12.03	0.71050	0.71060	0.00010
11.98	0.71062	0.71062	0.00010
11.94	0.71033	0.71060	0.00011
11.90	0.71065	0.71055	0.00018
11.85	0.71053	0.71056	0.00018
11.81	0.71072	0.71053	0.00019
11.76	0.71069	0.71051	0.00019
11.72	0.71094	0.71050	0.00019
11.68	0.71055	0.71049	0.00018
11.63	0.71048	0.71048	0.00018
11.59	0.71071	0.71042	0.00021
11.54	0.71038	0.71036	0.00021
11.50	0.70987	0.71035	0.00021
11.45	0.71076	0.71041	0.00018
11.41	0.71024	0.71040	0.00017
11.37	0.71047	0.71042	0.00017
11.32	0.71065	0.71037	0.00019
11.28	0.71083	0.71031	0.00019
11.23	0.71038	0.71027	0.00015
11.19	0.70993	0.71028	0.00016
11.14	0.71011	0.71034	0.00014
11.10	0.71024	0.71034	0.00014
11.06	0.71052	0.71034	0.00014
11.01	0.71065	0.71038	0.00018
10.97	0.71037	0.71035	0.00017
10.92	0.71002	0.71038	0.00017
10.88	0.71003	0.71044	0.00016
10.83	0.71047	0.71050	0.00013
10.79	0.71045	0.71048	0.00014
10.75	0.71056	0.71048	0.00014
10.70	0.71012	0.71048	0.00014
10.66	0.71023	0.71054	0.00012
10.61	0.71086	0.71055	0.00011
10.57	0.71043	0.71050	0.00009
10.53	0.71060	0.71052	0.00009
10.48	0.71064	0.71054	0.00011
10.44	0.71060	0.71051	0.00012
10.39	0.71032	0.71051	0.00012
10.35	0.71042	0.71052	0.00011
10.30	0.71060	0.71052	0.00012
10.26	0.71069	0.71052	0.00012

ARB 115.3.1 (M3)

Distance from cervix (mm)	$^{87}Sr/^{86}Sr$	10 point mov. average	2 SE on mov. average
12.81	0.71065	0.70964	0.00025
12.77	0.70954	0.70956	0.00013
12.69	0.70969	0.70952	0.00015
12.64	0.70934	0.70956	0.00018
12.60	0.70944	0.70954	0.00019
12.56	0.70936	0.70952	0.00020
12.52	0.70972	0.70953	0.00020
12.48	0.70945	0.70959	0.00026
12.43	0.70953	0.70958	0.00026
12.39	0.71001	0.70956	0.00027
12.35	0.70919	0.70952	0.00025
12.31	0.70949	0.70953	0.00024
12.27	0.71006	0.70954	0.00024
12.22	0.70914	0.70957	0.00027
12.18	0.70923	0.70961	0.00025
12.14	0.70950	0.70964	0.00024
12.10	0.71032	0.70970	0.00025
12.06	0.70934	0.70963	0.00021
12.01	0.70928	0.70967	0.00020
11.97	0.70959	0.70967	0.00020
11.93	0.70935	0.70964	0.00021
11.89	0.70958	0.70970	0.00021
11.85	0.71034	0.70978	0.00025
11.80	0.70957	0.70972	0.00021
11.76	0.70956	0.70972	0.00021
11.72	0.71005	0.70974	0.00021
11.68	0.70966	0.70965	0.00023
11.64	0.70969	0.70967	0.00023
11.59	0.70930	0.70965	0.00024
11.55	0.70926	0.70972	0.00023
11.51	0.71003	0.70975	0.00021
11.47	0.71033	0.70973	0.00020
11.43	0.70974	0.70973	0.00020
11.38	0.70960	0.70973	0.00020
11.34	0.70972	0.70971	0.00021
11.30	0.70913	0.70971	0.00021
11.26	0.70989	0.70979	0.00017
11.22	0.70947	0.70976	0.00018
11.17	0.71000	0.70981	0.00017
11.13	0.70957	0.70980	0.00016
11.09	0.70987	0.70983	0.00016
11.05	0.71030	0.70977	0.00019
11.01	0.70970	0.70969	0.00016
10.96	0.70944	0.70967	0.00016
10.92	0.70969	0.70967	0.00016
10.88	0.71001	0.70979	0.00028
10.84	0.70955	0.70977	0.00028

10.22	0.71030	0.71053	0.00013
10.17	0.71037	0.71056	0.00012
10.13	0.71063	0.71058	0.00011
10.08	0.71085	0.71060	0.00012
10.04	0.71030	0.71053	0.00014
9.99	0.71060	0.71054	0.00013
9.95	0.71045	0.71048	0.00016
9.91	0.71037	0.71040	0.00023
9.86	0.71061	0.71038	0.00024
9.82	0.71085	0.71038	0.00023
9.77	0.71051	0.71031	0.00021
9.73	0.71065	0.71029	0.00021
9.69	0.71079	0.71032	0.00024
9.64	0.71016	0.71034	0.00026
9.60	0.71035	0.71035	0.00025
9.55	0.71007	0.71031	0.00026
9.51	0.70964	0.71035	0.00026
9.46	0.71020	0.71043	0.00020
9.42	0.71056	0.71043	0.00020
9.38	0.71017	0.71043	0.00020
9.33	0.71033	0.71050	0.00021
9.29	0.71097	0.71050	0.00021
9.24	0.71097	0.71050	0.00021
9.20	0.71023	0.71045	0.00018
9.15	0.71002	0.71051	0.00019
9.11	0.71041	0.71053	0.00017
9.07	0.71049	0.71055	0.00016
9.02	0.71020	0.71050	0.00020
8.98	0.71056	0.71056	0.00020
8.93	0.71084	0.71057	0.00020
8.89	0.71033	0.71049	0.00021
8.84	0.71097	0.71050	0.00021
8.80	0.71044	0.71046	0.00018
8.76	0.71082	0.71044	0.00018
8.71	0.71025	0.71035	0.00018
8.67	0.71061	0.71038	0.00019
8.62	0.70998	0.71040	0.00020
8.58	0.71080	0.71052	0.00024
8.54	0.71062	0.71045	0.00024
8.49	0.71009	0.71049	0.00027
8.45	0.71041	0.71054	0.00025
8.40	0.71054	0.71055	0.00025
8.36	0.71024	0.71053	0.00025
8.31	0.70995	0.71057	0.00024
8.27	0.71052	0.71067	0.00021
8.23	0.71080	0.71072	0.00022
8.18	0.71122	0.71069	0.00023
8.14	0.71011	0.71064	0.00020
8.09	0.71106	0.71062	0.00022
8.05	0.71056	0.71051	0.00023
10.80	0.70994	0.70974	0.00029
10.75	0.70988	0.70974	0.00029
10.71	0.70993	0.70975	0.00029
10.67	0.70930	0.70977	0.00031
10.63	0.70947	0.70982	0.00029
10.59	0.70950	0.70987	0.00028
10.54	0.70948	0.70988	0.00028
10.50	0.71084	0.70996	0.00027
10.46	0.70980	0.70990	0.00020
10.42	0.70928	0.70985	0.00023
10.38	0.70990	0.70984	0.00023
10.33	0.70995	0.70983	0.00023
10.29	0.71023	0.70984	0.00024
10.25	0.70972	0.70983	0.00023
10.21	0.71001	0.70983	0.00023
10.17	0.70958	0.70977	0.00024
10.12	0.71033	0.70976	0.00024
10.08	0.71016	0.70973	0.00021
10.04	0.70933	0.70962	0.00023
10.00	0.70925	0.70970	0.00023
9.96	0.70980	0.70978	0.00022
9.91	0.71004	0.70979	0.00022
9.87	0.71007	0.70981	0.00023
9.83	0.70976	0.70983	0.00025
9.79	0.70933	0.70987	0.00026
9.75	0.70957	0.70994	0.00023
9.70	0.70997	0.71000	0.00022
9.66	0.70909	0.70999	0.00022
9.62	0.71010	0.71004	0.00015
9.58	0.71005	0.70995	0.00021
9.54	0.70988	0.70997	0.00022
9.49	0.71024	0.70996	0.00022
9.45	0.71026	0.70991	0.00022
9.41	0.71025	0.70990	0.00021
9.37	0.71001	0.70984	0.00020
9.33	0.71018	0.70986	0.00022
9.28	0.70986	0.70986	0.00021
9.24	0.70953	0.70987	0.00022
9.20	0.70925	0.70990	0.00020
9.16	0.71023	0.71001	0.00016
9.12	0.70983	0.70997	0.00015
9.07	0.70967	0.71000	0.00015
9.03	0.71021	0.70999	0.00016
8.99	0.70965	0.70990	0.00020
8.95	0.71022	0.70994	0.00020
8.91	0.71015	0.70991	0.00019
8.86	0.70997	0.70986	0.00019
8.82	0.70986	0.70987	0.00020
8.78	0.71032	0.70985	0.00020
8.74	0.70979	0.70979	0.00017

8.00	0.71046	0.71058	0.00027
7.96	0.71042	0.71059	0.00027
7.92	0.71059	0.71060	0.00027
7.87	0.71095	0.71068	0.00031
7.83	0.71107	0.71064	0.00031
7.78	0.71046	0.71057	0.00030
7.74	0.71070	0.71057	0.00030
7.69	0.70993	0.71060	0.00031
7.65	0.70995	0.71066	0.00027
7.61	0.71123	0.71071	0.00023
7.56	0.71060	0.71058	0.00024
7.52	0.71052	0.71054	0.00025
7.47	0.71140	0.71053	0.00026
7.43	0.71054	0.71047	0.00019
7.39	0.71036	0.71042	0.00021
7.34	0.71047	0.71039	0.00022
7.30	0.71096	0.71036	0.00023
7.25	0.71056	0.71028	0.00018
7.21	0.71046	0.71029	0.00019
7.16	0.70997	0.71023	0.00021
7.12	0.71013	0.71026	0.00020
7.08	0.71044	0.71028	0.00020
7.03	0.71085	0.71030	0.00021
6.99	0.71000	0.71021	0.00018
6.94	0.71003	0.71022	0.00017
6.90	0.71016	0.71031	0.00022
6.85	0.71025	0.71026	0.00026
6.81	0.71064	0.71034	0.00030
6.77	0.70980	0.71027	0.00030
6.72	0.71026	0.71029	0.00029
6.68	0.71039	0.71029	0.00029
6.63	0.71062	0.71029	0.00029
6.59	0.70994	0.71029	0.00029
6.55	0.71010	0.71036	0.00028
6.50	0.71096	0.71039	0.00028
6.46	0.70964	0.71032	0.00025
6.41	0.71102	0.71037	0.00020
6.37	0.71000	0.71032	0.00014
6.32	0.71000	0.71040	0.00016
6.28	0.71027	0.71045	0.00013
6.24	0.71036	0.71043	0.00014
6.19	0.71064	0.71038	0.00018
6.15	0.71059	0.71029	0.00022
6.10	0.71047	0.71027	0.00021
6.06	0.71018	0.71028	0.00022
6.01	0.71021	0.71027	0.00022
5.97	0.71045	0.71027	0.00022
5.93	0.71088	0.71021	0.00023
5.88	0.71042	0.71016	0.00018
5.84	0.71015	0.71015	0.00017

8.70	0.71013	0.70976	0.00018
8.65	0.70962	0.70972	0.00017
8.61	0.70928	0.70973	0.00016
8.57	0.71010	0.70981	0.00015
8.53	0.70991	0.70981	0.00015
8.49	0.70962	0.70977	0.00016
8.44	0.71012	0.70979	0.00016
8.40	0.70966	0.70984	0.00022
8.36	0.70970	0.70992	0.00025
8.32	0.70947	0.70988	0.00028
8.28	0.70973	0.70990	0.00026
8.23	0.70967	0.70993	0.00026
8.19	0.71013	0.70998	0.00026
8.15	0.71010	0.71001	0.00027
8.11	0.70947	0.71003	0.00028
8.07	0.70984	0.71003	0.00027
8.02	0.71063	0.71005	0.00027
7.98	0.71044	0.70999	0.00024
7.94	0.70930	0.70988	0.00025
7.90	0.70973	0.70995	0.00022
7.86	0.71000	0.70995	0.00022
7.81	0.71016	0.71002	0.00027
7.77	0.71045	0.71007	0.00030
7.73	0.71024	0.71005	0.00029
7.69	0.70953	0.71002	0.00029
7.65	0.71004	0.71008	0.00027
7.60	0.71005	0.71018	0.00032
7.56	0.70927	0.71019	0.00032
7.52	0.71002	0.71025	0.00026
7.48	0.70972	0.71025	0.00026
7.44	0.71072	0.71033	0.00024
7.39	0.71068	0.71029	0.00022
7.35	0.71019	0.71019	0.00023
7.31	0.71000	0.71018	0.00023
7.27	0.71010	0.71018	0.00023
7.23	0.71101	0.71020	0.00023
7.18	0.71018	0.71015	0.00016
7.14	0.70989	0.71018	0.00018
7.10	0.71005	0.71017	0.00018
7.06	0.71051	0.71023	0.00021
7.02	0.71030	0.71020	0.00020
6.97	0.70972	0.71017	0.00020
6.93	0.71002	0.71018	0.00019
6.89	0.71002	0.71010	0.00028
6.85	0.71035	0.71007	0.00029
6.81	0.71046	0.70998	0.00030
6.76	0.71049	0.70993	0.00028
6.72	0.70978	0.70987	0.00025
6.68	0.71067	0.70989	0.00025
6.64	0.71022	0.70985	0.00021

5.79	0.70986	0.71017	0.00018
5.75	0.70968	0.71024	0.00018
5.70	0.71039	0.71027	0.00016
5.66	0.71058	0.71023	0.00016
5.62	0.71008	0.71020	0.00014
5.57	0.71021	0.71023	0.00014
5.53	0.70990	0.71028	0.00017
5.48	0.71035	0.71027	0.00018
5.44	0.71029	0.71026	0.00018
5.40	0.71038	0.71022	0.00019
5.35	0.71059	0.71022	0.00019
5.31	0.70991	0.71014	0.00020
5.26	0.71006	0.71015	0.00019
5.22	0.71020	0.71014	0.00019
5.17	0.71039	0.71008	0.00023
5.13	0.71071	0.71004	0.00022
5.09	0.70981	0.70998	0.00016
5.04	0.71026	0.70995	0.00017
5.00	0.70992	0.70992	0.00016
4.95	0.71039	0.70991	0.00016
4.91	0.70972	0.70987	0.00012
4.86	0.71005	0.70990	0.00012
4.82	0.70997	0.70992	0.00014
4.78	0.70953	0.70989	0.00014
4.73	0.71004	0.70995	0.00012
4.69	0.71006	0.70992	0.00014
4.64	0.70958	0.70989	0.00013
4.60	0.70993	0.70997	0.00014
4.56	0.70984	0.70999	0.00014
4.51	0.70999	0.71007	0.00019
4.47	0.70999	0.71005	0.00020
4.42	0.71026	0.71008	0.00020
4.38	0.70973	0.71005	0.00019
4.33	0.71013	0.71004	0.00021
4.29	0.70966	0.71010	0.00025
4.25	0.70980	0.71024	0.00030
4.20	0.71035	0.71023	0.00031
4.16	0.71011	0.71025	0.00031
4.11	0.71066	0.71027	0.00031
4.07	0.70985	0.71024	0.00030
4.02	0.71022	0.71029	0.00029
3.98	0.71003	0.71030	0.00029
3.94	0.70957	0.71029	0.00029
3.89	0.71076	0.71039	0.00025
3.85	0.71110	0.71036	0.00023
3.80	0.70971	0.71026	0.00017
3.76	0.71046	0.71030	0.00013
3.71	0.71033	0.71030	0.00013
3.67	0.71040	0.71022	0.00020
3.63	0.71031	0.71019	0.00019

6.60	0.71002	0.70979	0.00020
6.55	0.70979	0.70975	0.00020
6.51	0.70917	0.70972	0.00020
6.47	0.70972	0.70975	0.00017
6.43	0.70953	0.70975	0.00017
6.39	0.70987	0.70979	0.00017
6.34	0.70991	0.70982	0.00018
6.30	0.70998	0.70987	0.00022
6.26	0.71033	0.70984	0.00022
6.22	0.70957	0.70986	0.00023
6.17	0.70965	0.70990	0.00022
6.13	0.70950	0.70999	0.00025
6.09	0.70944	0.71006	0.00023
6.05	0.70971	0.71017	0.00020
6.01	0.70998	0.71017	0.00020
5.96	0.71012	0.71015	0.00021
5.92	0.71041	0.71016	0.00021
5.88	0.70973	0.71022	0.00027
5.84	0.71047	0.71032	0.00026
5.80	0.70997	0.71030	0.00026
5.75	0.71054	0.71027	0.00028
5.71	0.71024	0.71017	0.00031
5.67	0.71053	0.71014	0.00031
5.63	0.70969	0.71011	0.00030
5.59	0.70979	0.71012	0.00029
5.54	0.71025	0.71012	0.00029
5.50	0.71097	0.71008	0.00030
5.46	0.71076	0.71000	0.00023
5.42	0.71024	0.70996	0.00018
5.38	0.70967	0.70998	0.00020
5.33	0.70955	0.71002	0.00019
5.29	0.70996	0.71006	0.00016
5.25	0.71024	0.71019	0.00028
5.21	0.70978	0.71017	0.00028
5.17	0.70979	0.71018	0.00028
5.12	0.70980	0.71020	0.00027
5.08	0.71018	0.71022	0.00026
5.04	0.71039	0.71017	0.00028
5.00	0.71048	0.71012	0.00028
4.96	0.71008	0.71007	0.00027
4.91	0.70988	0.71006	0.00027
4.87	0.71123	0.71008	0.00027
4.83	0.71005	0.70998	0.00009
4.79	0.70991	0.70993	0.00012
4.75	0.70997	0.70990	0.00014
4.70	0.71003	0.70983	0.00019
4.66	0.70971	0.70982	0.00018
4.62	0.70981	0.70984	0.00018
4.58	0.71005	0.70980	0.00020
4.54	0.70991	0.70979	0.00020

3.58	0.71035	0.71014	0.00020
3.54	0.70992	0.71015	0.00020
3.49	0.71061	0.71018	0.00020
3.45	0.71040	0.71010	0.00018
3.41	0.71016	0.71008	0.00017
3.36	0.71003	0.71012	0.00019
3.32	0.71045	0.71014	0.00019
3.27	0.70955	0.71013	0.00019
3.23	0.71011	0.71024	0.00016
3.18	0.70986	0.71022	0.00017
3.14	0.71036	0.71028	0.00016
3.10	0.71028	0.71030	0.00017
3.05	0.70982	0.71026	0.00018
3.01	0.71020	0.71032	0.00015
2.96	0.71050	0.71033	0.00015
2.92	0.71022	0.71025	0.00020
2.87	0.71036	0.71024	0.00020
2.83	0.71066	0.71024	0.00020
2.79	0.70995	0.71019	0.00018
2.74	0.71041	0.71012	0.00026
2.70	0.71056	0.71013	0.00027
2.65	0.70993	0.70996	0.00035
2.61	0.71045	0.70997	0.00035
2.56	0.71027	0.70996	0.00035
2.52	0.70966	0.70991	0.00034
2.48	0.71015	0.70998	0.00034
2.43	0.71033	0.70996	0.00034
2.39	0.71024	0.70992	0.00033
2.34	0.70922	0.70995	0.00035
2.30	0.71050	0.71009	0.00034
2.26	0.70883	0.71000	0.00034
2.21	0.71003	0.71009	0.00024
2.17	0.71034	0.71013	0.00025
2.12	0.70985	0.71006	0.00025
2.08	0.71032	0.71014	0.00027
2.03	0.70994	0.71013	0.00027
1.99	0.70990	0.71011	0.00028
1.95	0.71059	0.71012	0.00028
1.90	0.71065	0.71005	0.00026
1.86	0.70958	0.70997	0.00022
1.81	0.70965	0.71001	0.00021
1.77	0.71044	0.71012	0.00024
1.72	0.70971	0.71010	0.00023
1.68	0.71059	0.71017	0.00022
1.64	0.71026	0.71012	0.00020
1.59	0.70970	0.71007	0.00021
1.55	0.71005	0.71009	0.00020
1.50	0.70988	0.71010	0.00020
1.46	0.70981	0.71013	0.00019
1.42	0.71001	0.71010	0.00022

4.49	0.71014	0.70977	0.00020
4.45	0.71021	0.70973	0.00018
4.41	0.70959	0.70967	0.00014
4.37	0.70956	0.70970	0.00015
4.33	0.70927	0.70976	0.00017
4.28	0.70992	0.70987	0.00017
4.24	0.70992	0.70986	0.00017
4.20	0.70944	0.70978	0.00022
4.16	0.70997	0.70983	0.00021
4.12	0.70969	0.70983	0.00021
4.07	0.70971	0.70993	0.00026
4.03	0.70959	0.70991	0.00028
3.99	0.70993	0.70991	0.00028
3.95	0.71014	0.70990	0.00028
3.91	0.71038	0.70986	0.00027
3.86	0.70983	0.70988	0.00030
3.82	0.70917	0.70986	0.00030
3.78	0.70990	0.70994	0.00026
3.74	0.71000	0.70995	0.00026
3.70	0.71068	0.70996	0.00026
3.65	0.70947	0.70989	0.00021
3.61	0.70958	0.70988	0.00022
3.57	0.70982	0.70989	0.00021
3.53	0.70973	0.70995	0.00023
3.49	0.71061	0.71002	0.00025
3.44	0.70963	0.71006	0.00030
3.40	0.71000	0.71009	0.00028
3.36	0.70999	0.71009	0.00028
3.32	0.71015	0.71017	0.00032
3.28	0.70995	0.71016	0.00032
3.23	0.70937	0.71019	0.00032
3.19	0.70968	0.71019	0.00032
3.15	0.71039	0.71023	0.00030
3.11	0.71044	0.71019	0.00030
3.07	0.71101	0.71014	0.00030
3.02	0.70997	0.71009	0.00026
2.98	0.70995	0.71016	0.00027
2.94	0.71081	0.71020	0.00027
2.90	0.71002	0.71008	0.00026
2.86	0.71031	0.71000	0.00031
2.81	0.70934	0.70997	0.00030
2.77	0.71005	0.71003	0.00027
2.73	0.70996	0.70996	0.00030
2.69	0.70993	0.71008	0.00038
2.65	0.71059	0.71016	0.00041
2.60	0.71059	0.71010	0.00039
2.56	0.71037	0.70999	0.00039
2.52	0.70961	0.70997	0.00038
2.48	0.70923	0.71003	0.00038
2.44	0.71005	0.71019	0.00036

1.37	0.71076	0.71009	0.00022
1.33	0.71026	0.71005	0.00018
1.28	0.71042	0.71007	0.00020
1.24	0.71006	0.71003	0.00018
1.19	0.70977	0.71007	0.00020
1.15	0.70990	0.71008	0.00020
1.11	0.71011	0.71005	0.00022
1.06	0.71018	0.71002	0.00023
1.02	0.70951	0.71001	0.00022
0.97	0.70990	0.71005	0.00019
0.93	0.71041	0.71015	0.00025
0.88	0.71045	0.71013	0.00025
0.84	0.71001	0.71005	0.00025
0.80	0.71048	0.71006	0.00025
0.75	0.70987	0.71000	0.00024
0.71	0.70955	0.70999	0.00024
0.66	0.70981	0.71003	0.00022
0.62	0.71007	0.71003	0.00022
0.57	0.70997	0.70998	0.00024
0.53	0.71090	0.70997	0.00024
0.49	0.71022	0.70987	0.00012
0.44	0.70965	0.70985	0.00010
0.40	0.71005	0.70987	0.00009
0.35	0.70992	0.70985	0.00009
0.31	0.70979	0.70984	0.00009
0.27	0.70991	0.70984	0.00010
0.22	0.70986	0.70983	0.00011
0.18	0.70955	0.70983	0.00013
0.13	0.70982	0.70992	0.00007
0.09	0.70990	0.70997	0.00006
0.04	0.71003		

2.39	0.70994	0.71022	0.00036
2.35	0.70931	0.71025	0.00036
2.31	0.71113	0.71034	0.00029
2.27	0.71077	0.71022	0.00024
2.23	0.71000	0.71017	0.00021
2.18	0.70954	0.71018	0.00021
2.14	0.71009	0.71023	0.00016
2.10	0.71026	0.71033	0.00024
2.06	0.71080	0.71023	0.00032
2.02	0.71039	0.71015	0.00030
1.97	0.71018	0.71010	0.00030
1.93	0.71025	0.71008	0.00030
1.89	0.70988	0.71009	0.00030
1.85	0.71032	0.71022	0.00037
1.81	0.71009	0.71020	0.00037
1.76	0.71003	0.71030	0.00040
1.72	0.71113	0.71030	0.00040
1.68	0.70922	0.71022	0.00036
1.64	0.71002	0.71035	0.00029
1.60	0.70987	0.71043	0.00029
1.55	0.71003	0.71054	0.00028
1.51	0.71029	0.71059	0.00025
1.47	0.71118	0.71062	0.00025
1.43	0.71015	0.71057	0.00021
1.39	0.71109	0.71062	0.00019
1.34	0.71003	0.71056	0.00016
1.30	0.71035	0.71067	0.00015
1.26	0.71048	0.71075	0.00016
1.22	0.71079	0.71085	0.00019
1.18	0.71099	0.71088	0.00020
1.13	0.71059	0.71087	0.00020
1.09	0.71058	0.71087	0.00020
1.05	0.71062	0.71098	0.00024
1.01	0.71065	0.71102	0.00023
0.97	0.71055	0.71105	0.00021
0.92	0.71113	0.71103	0.00024
0.88	0.71115	0.71095	0.00027
0.84	0.71141	0.71089	0.00028
0.80	0.71118	0.71081	0.00026
0.76	0.71083	0.71075	0.00025
0.71	0.71062	0.71069	0.00027
0.67	0.71165	0.71071	0.00027
0.63	0.71099	0.71060	0.00017
0.59	0.71099	0.71055	0.00015
0.55	0.71033	0.71046	0.00013
0.50	0.71033	0.71053	0.00018
0.46	0.71062	0.71059	0.00019
0.42	0.71059	0.71061	0.00019
0.38	0.71052	0.71061	0.00020
0.34	0.71027	0.71062	0.00022

0.29	0.71084	0.71067	0.00021
0.25	0.71055	0.71064	0.00023
0.21	0.71041	0.71066	0.00025
0.17	0.71010	0.71072	0.00027
0.13	0.71106	0.71093	0.00008
0.08	0.71093	0.71086	0.00006
0.04	0.71080		

ARB 116.2.1 (M2)

Distance from cervix (mm)	$^{87}Sr/^{86}Sr$	10 point mov. average	2 SE on mov. average
14.83	0.71001	0.71016	0.00013
14.79	0.70992	0.71017	0.00012
14.74	0.71030	0.71018	0.00012
14.70	0.71023	0.71016	0.00012
14.65	0.71049	0.71009	0.00017
14.61	0.70989	0.71005	0.00015
14.57	0.71012	0.71010	0.00016
14.52	0.71037	0.71005	0.00019
14.48	0.71028	0.70995	0.00022
14.43	0.71004	0.70990	0.00021
14.39	0.71012	0.70989	0.00021
14.34	0.70995	0.70984	0.00021
14.30	0.71015	0.70985	0.00021
14.26	0.70952	0.70985	0.00021
14.21	0.71003	0.70990	0.00020
14.17	0.71043	0.70985	0.00020
14.12	0.70962	0.70980	0.00016
14.08	0.70935	0.70981	0.00016
14.03	0.70979	0.70986	0.00012
13.99	0.70998	0.70986	0.00012
13.95	0.70962	0.70981	0.00013
13.90	0.71003	0.70980	0.00014
13.86	0.71011	0.70978	0.00013
13.81	0.71006	0.70971	0.00013
13.77	0.70957	0.70968	0.00010
13.72	0.70987	0.70970	0.00010
13.68	0.70976	0.70973	0.00013
13.64	0.70978	0.70975	0.00015
13.59	0.70978	0.70973	0.00015
13.55	0.70956	0.70973	0.00015
13.50	0.70950	0.70975	0.00015
13.46	0.70981	0.70973	0.00016
13.41	0.70939	0.70976	0.00018
13.37	0.70982	0.70983	0.00017
13.33	0.70971	0.70979	0.00019
13.28	0.71015	0.70977	0.00020
13.24	0.71004	0.70969	0.00020
13.19	0.70958	0.70965	0.00018
13.15	0.70970	0.70963	0.00019
13.10	0.70979	0.70960	0.00019
13.06	0.70932	0.70966	0.00025
13.02	0.71013	0.70967	0.00025
12.97	0.71010	0.70962	0.00023
12.93	0.70940	0.70960	0.00021
12.88	0.70950	0.70961	0.00021
12.84	0.70932	0.70963	0.00021
12.79	0.70964	0.70969	0.00020

12.75	0.70944	0.70964	0.00023
12.71	0.70937	0.70968	0.00023
12.66	0.71041	0.70972	0.00022
12.62	0.70937	0.70961	0.00017
12.57	0.70963	0.70963	0.00016
12.53	0.70989	0.70959	0.00019
12.48	0.70954	0.70950	0.00021
12.44	0.70968	0.70953	0.00023
12.40	0.70991	0.70954	0.00023
12.35	0.70913	0.70952	0.00022
12.31	0.70987	0.70957	0.00020
12.26	0.70981	0.70958	0.00021
12.22	0.70932	0.70960	0.00022
12.18	0.70956	0.70965	0.00022
12.13	0.70916	0.70967	0.00022
12.09	0.70899	0.70972	0.00018
12.04	0.70992	0.70979	0.00009
12.00	0.70974	0.70981	0.00010
11.95	0.70970	0.70979	0.00011
11.91	0.70964	0.70974	0.00015
11.87	0.70995	0.70974	0.00015
11.82	0.71006	0.70969	0.00015
11.78	0.70982	0.70966	0.00013
11.73	0.70971	0.70968	0.00015
11.69	0.70969	0.70965	0.00016
11.64	0.70971	0.70963	0.00016
11.60	0.71004	0.70965	0.00017
11.56	0.70954	0.70964	0.00016
11.51	0.70927	0.70965	0.00016
11.47	0.70963	0.70969	0.00014
11.42	0.70946	0.70968	0.00014
11.38	0.70969	0.70971	0.00013
11.33	0.71005	0.70969	0.00013
11.29	0.70940	0.70960	0.00016
11.25	0.70951	0.70962	0.00015
11.20	0.70993	0.70963	0.00015
11.16	0.70990	0.70960	0.00013
11.11	0.70967	0.70954	0.00013
11.07	0.70961	0.70954	0.00014
11.02	0.70955	0.70953	0.00013
10.98	0.70974	0.70947	0.00018
10.94	0.70957	0.70944	0.00017
10.89	0.70909	0.70941	0.00017
10.85	0.70963	0.70951	0.00019
10.80	0.70962	0.70950	0.00019
10.76	0.70964	0.70951	0.00019
10.71	0.70925	0.70949	0.00019
10.67	0.70977	0.70952	0.00018
10.63	0.70949	0.70952	0.00018
10.58	0.70894	0.70952	0.00018

10.54	0.70947	0.70961	0.00013
10.49	0.70927	0.70967	0.00015
10.45	0.71001	0.70970	0.00013
10.40	0.70951	0.70970	0.00013
10.36	0.70972	0.70972	0.00012
10.32	0.70951	0.70965	0.00018
10.27	0.70955	0.70968	0.00018
10.23	0.70969	0.70969	0.00018
10.18	0.70955	0.70971	0.00019
10.14	0.70986	0.70970	0.00019
10.09	0.71003	0.70968	0.00019
10.05	0.70957	0.70963	0.00017
10.01	0.71005	0.70965	0.00017
9.96	0.70967	0.70960	0.00015
9.92	0.70904	0.70957	0.00015
9.87	0.70976	0.70965	0.00011
9.83	0.70968	0.70970	0.00016
9.78	0.70990	0.70966	0.00018
9.74	0.70940	0.70963	0.00017
9.70	0.70964	0.70966	0.00016
9.65	0.70959	0.70968	0.00017
9.61	0.70972	0.70967	0.00017
9.56	0.70959	0.70965	0.00017
9.52	0.70938	0.70965	0.00017
9.47	0.70982	0.70971	0.00017
9.43	0.71025	0.70971	0.00017
9.39	0.70928	0.70966	0.00013
9.34	0.70966	0.70973	0.00011
9.30	0.70970	0.70973	0.00011
9.25	0.70983	0.70974	0.00011
9.21	0.70946	0.70971	0.00011
9.16	0.70955	0.70972	0.00011
9.12	0.70959	0.70974	0.00010
9.08	0.70999	0.70975	0.00009
9.03	0.70976	0.70972	0.00008
8.99	0.70977	0.70972	0.00008
8.94	0.70998	0.70977	0.00013
8.90	0.70969	0.70974	0.00013
8.85	0.70978	0.70975	0.00013
8.81	0.70956	0.70977	0.00013
8.77	0.70954	0.70981	0.00014
8.72	0.70969	0.70984	0.00012
8.68	0.70971	0.70987	0.00012
8.63	0.70970	0.70990	0.00012
8.59	0.70982	0.70988	0.00013
8.54	0.71026	0.70991	0.00013
8.50	0.70963	0.70980	0.00017
8.46	0.70980	0.70982	0.00016
8.41	0.70996	0.70982	0.00016
8.37	0.71004	0.70976	0.00018

8.32	0.70980	0.70977	0.00019
8.28	0.70999	0.70981	0.00021
8.23	0.70998	0.70980	0.00021
8.19	0.70957	0.70983	0.00023
8.15	0.71004	0.70985	0.00022
8.10	0.70923	0.70987	0.00023
8.06	0.70980	0.70992	0.00019
8.01	0.70978	0.70992	0.00019
7.97	0.70934	0.70992	0.00019
7.92	0.71017	0.70999	0.00014
7.88	0.71020	0.70993	0.00016
7.84	0.70991	0.70988	0.00015
7.79	0.71026	0.70983	0.00018
7.75	0.70979	0.70979	0.00015
7.70	0.71024	0.70982	0.00017
7.66	0.70967	0.70981	0.00016
7.61	0.70983	0.70979	0.00017
7.57	0.70982	0.70978	0.00017
7.53	0.71005	0.70982	0.00019
7.48	0.70952	0.70976	0.00019
7.44	0.70974	0.70976	0.00019
7.39	0.70939	0.70977	0.00019
7.35	0.70982	0.70978	0.00018
7.31	0.71012	0.70977	0.00018
7.26	0.71015	0.70971	0.00017
7.22	0.70949	0.70973	0.00018
7.17	0.70969	0.70976	0.00017
7.13	0.71020	0.70976	0.00017
7.08	0.70950	0.70975	0.00017
7.04	0.70952	0.70976	0.00017
7.00	0.70978	0.70983	0.00018
6.95	0.70953	0.70981	0.00018
6.91	0.70973	0.70985	0.00017
6.86	0.70956	0.70988	0.00017
6.82	0.71026	0.70992	0.00016
6.77	0.70981	0.70988	0.00014
6.73	0.70967	0.70988	0.00014
6.69	0.71018	0.70991	0.00013
6.64	0.70955	0.70986	0.00012
6.60	0.71020	0.70988	0.00011
6.55	0.70966	0.70986	0.00009
6.51	0.70993	0.70989	0.00008
6.46	0.71002	0.70994	0.00013
6.42	0.70995	0.70991	0.00014
6.38	0.70980	0.70992	0.00014
6.33	0.70980	0.70991	0.00014
6.29	0.71001	0.70993	0.00014
6.24	0.70970	0.70992	0.00014
6.20	0.70972	0.70992	0.00014
6.15	0.70998	0.70997	0.00014

6.11	0.71005	0.70996	0.00014
6.07	0.71042	0.70996	0.00014
6.02	0.70972	0.70990	0.00011
5.98	0.70997	0.70989	0.00012
5.93	0.70971	0.70988	0.00012
5.89	0.71000	0.70993	0.00013
5.84	0.70994	0.70991	0.00013
5.80	0.70970	0.70992	0.00013
5.76	0.71016	0.70991	0.00014
5.71	0.70990	0.70987	0.00013
5.67	0.71010	0.70982	0.00015
5.62	0.70976	0.70977	0.00014
5.58	0.70961	0.70984	0.00019
5.53	0.70989	0.70986	0.00018
5.49	0.71021	0.70986	0.00018
5.45	0.70983	0.70986	0.00018
5.40	0.71001	0.70986	0.00018
5.36	0.70959	0.70981	0.00018
5.31	0.70977	0.70986	0.00018
5.27	0.70946	0.70989	0.00019
5.22	0.70960	0.70992	0.00016
5.18	0.71039	0.70993	0.00015
5.14	0.70981	0.70992	0.00013
5.09	0.70998	0.70988	0.00016
5.05	0.71012	0.70983	0.00018
5.00	0.70984	0.70984	0.00018
4.96	0.70955	0.70989	0.00020
4.91	0.71010	0.70989	0.00020
4.87	0.71004	0.70988	0.00019
4.83	0.70972	0.70991	0.00022
4.78	0.70976	0.70993	0.00021
4.74	0.71022	0.70994	0.00021
4.69	0.70947	0.70992	0.00020
4.65	0.70949	0.70997	0.00017
4.60	0.71022	0.71000	0.00014
4.56	0.71029	0.71000	0.00014
4.52	0.70962	0.70996	0.00013
4.47	0.70992	0.70997	0.00011
4.43	0.71036	0.70999	0.00012
4.38	0.70993	0.70998	0.00010
4.34	0.70992	0.70998	0.00010
4.29	0.70996	0.71002	0.00011
4.25	0.71005	0.71003	0.00011
4.21	0.70978	0.71001	0.00011
4.16	0.71019	0.71004	0.00010
4.12	0.70984	0.71002	0.00010
4.07	0.70981	0.71002	0.00010
4.03	0.71008	0.71006	0.00009
3.98	0.71022	0.71001	0.00013
3.94	0.71000	0.70998	0.00012

3.90	0.71024	0.70995	0.00013
3.85	0.71005	0.70989	0.00012
3.81	0.70985	0.70988	0.00012
3.76	0.71008	0.70989	0.00012
3.72	0.71001	0.70987	0.00011
3.67	0.70983	0.70992	0.00017
3.63	0.71019	0.70993	0.00017
3.59	0.70961	0.70987	0.00017
3.54	0.70993	0.70988	0.00016
3.50	0.70965	0.70987	0.00016
3.45	0.70970	0.70990	0.00015
3.41	0.70997	0.70992	0.00015
3.36	0.70987	0.70990	0.00015
3.32	0.70994	0.70991	0.00015
3.28	0.71050	0.70992	0.00015
3.23	0.70992	0.70982	0.00011
3.19	0.70962	0.70985	0.00012
3.14	0.70970	0.70982	0.00015
3.10	0.70982	0.70980	0.00016
3.05	0.70999	0.70979	0.00016
3.01	0.70987	0.70978	0.00016
2.97	0.70975	0.70974	0.00016
2.92	0.71000	0.70975	0.00016
2.88	0.71003	0.70977	0.00018
2.83	0.70954	0.70983	0.00026
2.79	0.71016	0.70989	0.00025
2.74	0.70939	0.70990	0.00026
2.70	0.70950	0.70995	0.00023
2.66	0.70964	0.71003	0.00022
2.61	0.70988	0.71006	0.00020
2.57	0.70955	0.71008	0.00020
2.52	0.70977	0.71014	0.00016
2.48	0.71021	0.71018	0.00014
2.44	0.71069	0.71013	0.00016
2.39	0.71012	0.71007	0.00010
2.35	0.71019	0.71010	0.00012
2.30	0.70996	0.71011	0.00013
2.26	0.71032	0.71016	0.00014
2.21	0.70987	0.71017	0.00015
2.17	0.71013	0.71017	0.00014
2.13	0.71013	0.71019	0.00014
2.08	0.71016	0.71016	0.00016
2.04	0.70977	0.71008	0.00022
1.99	0.71008	0.71011	0.00021
1.95	0.71036	0.71011	0.00021
1.90	0.71032	0.71009	0.00021
1.86	0.71051	0.71007	0.00020
1.82	0.71036	0.71008	0.00020
1.77	0.70991	0.71003	0.00020
1.73	0.71028	0.71002	0.00020

1.68	0.70983	0.71000	0.00019
1.64	0.70938	0.71001	0.00019
1.59	0.71010	0.70999	0.00022
1.55	0.71009	0.70996	0.00022
1.51	0.71015	0.70993	0.00023
1.46	0.71014	0.70995	0.00024
1.42	0.71055	0.70993	0.00024
1.37	0.70985	0.70987	0.00019
1.33	0.70983	0.70984	0.00020
1.28	0.71006	0.70982	0.00021
1.24	0.70997	0.70982	0.00020
1.20	0.70916	0.70977	0.00021
1.15	0.70983	0.70981	0.00017
1.11	0.70975	0.70983	0.00018
1.06	0.71037	0.70983	0.00018
1.02	0.70993	0.70977	0.00014
0.97	0.70998	0.70974	0.00014
0.93	0.70955	0.70970	0.00013
0.89	0.70964	0.70975	0.00013
0.84	0.71003	0.70978	0.00014
0.80	0.70950	0.70976	0.00013
0.75	0.70951	0.70978	0.00011
0.71	0.71006	0.70982	0.00010
0.66	0.70973	0.70976	0.00011
0.62	0.70975	0.70977	0.00011
0.58	0.70961	0.70980	0.00013
0.53	0.70963	0.70980	0.00013
0.49	0.70999	0.70983	0.00012
0.44	0.70996	0.70978	0.00014
0.40	0.70987	0.70976	0.00014
0.35	0.70969	0.70974	0.00015
0.31	0.70994	0.70975	0.00016
0.27	0.70943	0.70972	0.00017
0.22	0.70985	0.70978	0.00016
0.18	0.71005	0.70976	0.00018
0.13	0.70960	0.70966	0.00016
0.09	0.70994	0.70969	0.00022
0.04	0.70945		

ARB 117.2.1 (M2)

Distance from cervix (mm)	$^{87}Sr/^{86}Sr$	10 point mov. average	2 SE on mov. average
24.70	0.71087	0.71076	0.00017
24.66	0.71066	0.71070	0.00019
24.61	0.71056	0.71061	0.00028
24.57	0.71094	0.71074	0.00037
24.53	0.71020	0.71072	0.00037
24.48	0.71070	0.71073	0.00036
24.44	0.71058	0.71073	0.00036
24.40	0.71110	0.71076	0.00036
24.35	0.71092	0.71069	0.00036
24.31	0.71106	0.71070	0.00036
24.26	0.71033	0.71066	0.00035
24.22	0.70971	0.71076	0.00037
24.18	0.71185	0.71087	0.00029
24.13	0.71075	0.71077	0.00019
24.09	0.71028	0.71074	0.00021
24.05	0.71075	0.71078	0.00018
24.00	0.71086	0.71074	0.00020
23.96	0.71041	0.71080	0.00025
23.91	0.71101	0.71081	0.00025
23.87	0.71060	0.71071	0.00028
23.83	0.71137	0.71063	0.00034
23.78	0.71083	0.71051	0.00031
23.74	0.71086	0.71049	0.00030
23.70	0.71042	0.71051	0.00031
23.65	0.71065	0.71052	0.00031
23.61	0.71039	0.71052	0.00031
23.57	0.71149	0.71057	0.00031
23.52	0.71045	0.71045	0.00024
23.48	0.71008	0.71046	0.00024
23.43	0.70973	0.71052	0.00023
23.39	0.71023	0.71061	0.00014
23.35	0.71060	0.71065	0.00011
23.30	0.71103	0.71062	0.00014
23.26	0.71053	0.71057	0.00011
23.22	0.71068	0.71052	0.00014
23.17	0.71083	0.71049	0.00014
23.13	0.71038	0.71042	0.00014
23.08	0.71051	0.71041	0.00014
23.04	0.71074	0.71038	0.00015
23.00	0.71061	0.71033	0.00012
22.95	0.71062	0.71027	0.00013
22.91	0.71026	0.71027	0.00014
22.87	0.71051	0.71030	0.00015
22.82	0.71012	0.71031	0.00016
22.78	0.71038	0.71033	0.00016
22.73	0.71008	0.71040	0.00021
22.69	0.71029	0.71042	0.00020

ARB 117.3.1 (M3)

Distance from cervix (mm)	$^{87}Sr/^{86}Sr$	10 point mov. average	2 SE on mov. average
34.96	0.71165	0.71144	0.00026
34.92	0.71110	0.71142	0.00026
34.87	0.71199	0.71148	0.00025
34.83	0.71153	0.71138	0.00023
34.79	0.71210	0.71141	0.00025
34.74	0.71149	0.71129	0.00021
34.70	0.71092	0.71130	0.00021
34.66	0.71109	0.71129	0.00021
34.61	0.71096	0.71135	0.00022
34.57	0.71155	0.71141	0.00020
34.53	0.71149	0.71141	0.00021
34.48	0.71163	0.71137	0.00022
34.44	0.71106	0.71134	0.00021
34.40	0.71177	0.71134	0.00021
34.35	0.71092	0.71136	0.00023
34.31	0.71156	0.71145	0.00023
34.27	0.71090	0.71142	0.00023
34.22	0.71162	0.71141	0.00024
34.18	0.71162	0.71139	0.00023
34.14	0.71157	0.71138	0.00023
34.09	0.71105	0.71135	0.00023
34.05	0.71132	0.71142	0.00022
34.01	0.71104	0.71144	0.00023
33.96	0.71200	0.71159	0.00029
33.92	0.71185	0.71156	0.00027
33.88	0.71119	0.71154	0.00027
33.83	0.71087	0.71161	0.00027
33.79	0.71144	0.71172	0.00021
33.75	0.71151	0.71172	0.00021
33.70	0.71128	0.71174	0.00021
33.66	0.71166	0.71175	0.00020
33.62	0.71161	0.71169	0.00025
33.57	0.71249	0.71169	0.00025
33.53	0.71169	0.71163	0.00018
33.49	0.71161	0.71159	0.00019
33.44	0.71197	0.71156	0.00020
33.40	0.71193	0.71155	0.00020
33.36	0.71150	0.71152	0.00018
33.31	0.71168	0.71153	0.00018
33.27	0.71139	0.71153	0.00018
33.23	0.71099	0.71161	0.00023
33.18	0.71168	0.71165	0.00019
33.14	0.71186	0.71165	0.00019
33.09	0.71128	0.71162	0.00019
33.05	0.71130	0.71174	0.00023
33.01	0.71195	0.71177	0.00021
32.96	0.71154	0.71174	0.00021

22.65	0.71016	0.71045	0.00021
22.60	0.71031	0.71052	0.00021
22.56	0.70993	0.71061	0.00025
22.52	0.71068	0.71075	0.00023
22.47	0.71051	0.71076	0.00023
22.43	0.71069	0.71081	0.00023
22.38	0.71025	0.71085	0.00023
22.34	0.71106	0.71081	0.00029
22.30	0.71029	0.71080	0.00028
22.25	0.71065	0.71080	0.00028
22.21	0.71081	0.71084	0.00028
22.17	0.71125	0.71081	0.00029
22.12	0.71133	0.71065	0.00035
22.08	0.71079	0.71068	0.00038
22.03	0.71099	0.71069	0.00038
21.99	0.71107	0.71067	0.00038
21.95	0.70986	0.71061	0.00037
21.90	0.71091	0.71071	0.00033
21.86	0.71029	0.71068	0.00033
21.82	0.71108	0.71071	0.00032
21.77	0.71054	0.71074	0.00034
21.73	0.70968	0.71072	0.00035
21.69	0.71164	0.71083	0.00027
21.64	0.71080	0.71079	0.00022
21.60	0.71086	0.71081	0.00023
21.55	0.71045	0.71086	0.00026
21.51	0.71089	0.71096	0.00027
21.47	0.71059	0.71097	0.00027
21.42	0.71057	0.71108	0.00029
21.38	0.71141	0.71121	0.00031
21.34	0.71027	0.71117	0.00031
21.29	0.71079	0.71134	0.00027
21.25	0.71129	0.71141	0.00025
21.20	0.71093	0.71137	0.00027
21.16	0.71140	0.71136	0.00028
21.12	0.71149	0.71127	0.00033
21.07	0.71092	0.71130	0.00035
21.03	0.71169	0.71133	0.00034
20.99	0.71193	0.71132	0.00033
20.94	0.71096	0.71125	0.00030
20.90	0.71198	0.71112	0.00043
20.85	0.71155	0.71104	0.00039
20.77	0.71083	0.71103	0.00038
20.72	0.71085	0.71099	0.00039
20.64	0.71050	0.71112	0.00045
20.59	0.71185	0.71125	0.00044
20.55	0.71116	0.71131	0.00049
20.50	0.71160	0.71134	0.00050
20.46	0.71121	0.71139	0.00051
20.42	0.70973	0.71145	0.00052
32.92	0.71161	0.71176	0.00021
32.88	0.71169	0.71170	0.00025
32.83	0.71224	0.71168	0.00026
32.79	0.71139	0.71164	0.00023
32.75	0.71168	0.71173	0.00026
32.70	0.71157	0.71174	0.00026
32.66	0.71245	0.71185	0.00031
32.62	0.71154	0.71180	0.00028
32.57	0.71171	0.71188	0.00029
32.53	0.71174	0.71193	0.00030
32.49	0.71102	0.71193	0.00029
32.44	0.71144	0.71201	0.00022
32.40	0.71186	0.71210	0.00019
32.36	0.71231	0.71218	0.00021
32.31	0.71180	0.71215	0.00021
32.27	0.71262	0.71216	0.00021
32.23	0.71200	0.71211	0.00018
32.18	0.71225	0.71212	0.00018
32.14	0.71226	0.71209	0.00019
32.10	0.71176	0.71202	0.00021
32.05	0.71178	0.71201	0.00022
32.01	0.71232	0.71202	0.00021
31.97	0.71270	0.71193	0.00023
31.92	0.71203	0.71186	0.00016
31.88	0.71184	0.71185	0.00015
31.84	0.71213	0.71191	0.00020
31.79	0.71218	0.71194	0.00022
31.75	0.71190	0.71199	0.00026
31.71	0.71152	0.71204	0.00028
31.66	0.71168	0.71210	0.00025
31.62	0.71184	0.71213	0.00023
31.58	0.71145	0.71213	0.00024
31.53	0.71205	0.71220	0.00018
31.49	0.71187	0.71226	0.00020
31.45	0.71249	0.71229	0.00018
31.40	0.71245	0.71224	0.00018
31.36	0.71266	0.71214	0.00023
31.32	0.71242	0.71207	0.00020
31.27	0.71208	0.71211	0.00024
31.23	0.71201	0.71212	0.00024
31.19	0.71181	0.71213	0.00024
31.14	0.71214	0.71214	0.00023
31.10	0.71263	0.71216	0.00024
31.06	0.71219	0.71215	0.00023
31.01	0.71195	0.71216	0.00023
30.97	0.71148	0.71221	0.00024
30.93	0.71202	0.71233	0.00019
30.88	0.71278	0.71243	0.00022
30.84	0.71219	0.71241	0.00021
30.80	0.71213	0.71244	0.00021

20.37	0.71115	0.71162	0.00036
20.33	0.71140	0.71167	0.00034
20.29	0.71048	0.71161	0.00038
20.24	0.71208	0.71173	0.00029
20.20	0.71183	0.71166	0.00028
20.15	0.71246	0.71170	0.00030
20.11	0.71150	0.71162	0.00025
20.07	0.71207	0.71159	0.00027
20.02	0.71187	0.71148	0.00027
19.98	0.71136	0.71143	0.00025
19.94	0.71170	0.71145	0.00025
19.89	0.71080	0.71147	0.00026
19.85	0.71165	0.71159	0.00024
19.81	0.71139	0.71175	0.00040
19.76	0.71216	0.71177	0.00039
19.72	0.71174	0.71163	0.00043
19.67	0.71113	0.71167	0.00044
19.63	0.71101	0.71187	0.00051
19.59	0.71134	0.71195	0.00047
19.54	0.71162	0.71198	0.00046
19.50	0.71187	0.71194	0.00048
19.46	0.71203	0.71200	0.00049
19.41	0.71317	0.71195	0.00050
19.37	0.71167	0.71189	0.00044
19.32	0.71075	0.71198	0.00046
19.28	0.71214	0.71202	0.00042
19.24	0.71314	0.71207	0.00044
19.19	0.71181	0.71189	0.00040
19.15	0.71155	0.71183	0.00042
19.11	0.71123	0.71190	0.00042
19.06	0.71249	0.71195	0.00040
19.02	0.71155	0.71194	0.00039
18.97	0.71252	0.71203	0.00039
18.93	0.71262	0.71201	0.00038
18.89	0.71112	0.71198	0.00036
18.84	0.71269	0.71223	0.00044
18.80	0.71130	0.71213	0.00044
18.76	0.71127	0.71217	0.00042
18.71	0.71225	0.71220	0.00039
18.67	0.71169	0.71222	0.00039
18.62	0.71243	0.71227	0.00037
18.58	0.71244	0.71227	0.00037
18.54	0.71230	0.71217	0.00041
18.49	0.71229	0.71211	0.00041
18.45	0.71364	0.71209	0.00041
18.41	0.71165	0.71187	0.00025
18.36	0.71170	0.71188	0.00025
18.32	0.71162	0.71198	0.00029
18.27	0.71246	0.71205	0.00029
18.23	0.71216	0.71199	0.00028

30.75	0.71184	0.71247	0.00019
30.71	0.71235	0.71254	0.00013
30.67	0.71257	0.71256	0.00013
30.62	0.71227	0.71250	0.00018
30.58	0.71246	0.71255	0.00018
30.54	0.71271	0.71250	0.00021
30.49	0.71305	0.71249	0.00020
30.45	0.71249	0.71244	0.00016
30.41	0.71251	0.71242	0.00017
30.36	0.71248	0.71247	0.00021
30.32	0.71251	0.71258	0.00029
30.28	0.71259	0.71252	0.00032
30.23	0.71194	0.71244	0.00035
30.19	0.71273	0.71236	0.00042
30.15	0.71199	0.71228	0.00042
30.10	0.71259	0.71228	0.00042
30.06	0.71255	0.71224	0.00042
30.02	0.71231	0.71220	0.00041
29.97	0.71306	0.71219	0.00041
29.93	0.71351	0.71212	0.00037
29.89	0.71189	0.71198	0.00020
29.84	0.71179	0.71209	0.00029
29.80	0.71122	0.71212	0.00029
29.76	0.71190	0.71219	0.00021
29.71	0.71193	0.71225	0.00021
29.67	0.71223	0.71231	0.00021
29.62	0.71216	0.71230	0.00021
29.58	0.71216	0.71230	0.00021
29.54	0.71237	0.71229	0.00022
29.49	0.71211	0.71225	0.00022
29.45	0.71304	0.71226	0.00022
29.41	0.71204	0.71218	0.00014
29.36	0.71189	0.71217	0.00014
29.32	0.71254	0.71227	0.00019
29.28	0.71251	0.71223	0.00018
29.23	0.71218	0.71220	0.00017
29.19	0.71217	0.71227	0.00021
29.15	0.71199	0.71235	0.00024
29.10	0.71197	0.71236	0.00023
29.06	0.71222	0.71231	0.00029
29.02	0.71224	0.71230	0.00029
28.97	0.71196	0.71228	0.00029
28.93	0.71287	0.71234	0.00029
28.89	0.71222	0.71223	0.00027
28.80	0.71277	0.71227	0.00026
28.76	0.71287	0.71226	0.00026
28.71	0.71217	0.71220	0.00022
28.67	0.71151	0.71217	0.00023
28.63	0.71205	0.71222	0.00019
28.58	0.71211	0.71220	0.00019

18.19	0.71244	0.71202	0.00029
18.14	0.71143	0.71188	0.00033
18.10	0.71172	0.71197	0.00032
18.06	0.71212	0.71205	0.00033
18.01	0.71140	0.71201	0.00034
17.97	0.71171	0.71209	0.00031
17.93	0.71272	0.71209	0.00031
17.88	0.71235	0.71206	0.00029
17.84	0.71187	0.71204	0.00028
17.79	0.71244	0.71214	0.00033
17.75	0.71106	0.71216	0.00033
17.71	0.71231	0.71227	0.00023
17.66	0.71252	0.71228	0.00023
17.62	0.71176	0.71229	0.00023
17.58	0.71212	0.71235	0.00020
17.53	0.71175	0.71230	0.00024
17.49	0.71243	0.71230	0.00025
17.44	0.71213	0.71231	0.00025
17.40	0.71292	0.71227	0.00028
17.36	0.71255	0.71229	0.00030
17.31	0.71221	0.71224	0.00030
17.27	0.71242	0.71231	0.00033
17.23	0.71260	0.71237	0.00036
17.18	0.71234	0.71233	0.00036
17.14	0.71168	0.71236	0.00037
17.09	0.71169	0.71235	0.00037
17.05	0.71259	0.71245	0.00035
17.01	0.71170	0.71246	0.00035
16.96	0.71312	0.71260	0.00033
16.92	0.71201	0.71265	0.00038
16.88	0.71292	0.71275	0.00035
16.83	0.71309	0.71270	0.00035
16.79	0.71218	0.71259	0.00037
16.74	0.71259	0.71250	0.00045
16.70	0.71158	0.71249	0.00045
16.66	0.71269	0.71259	0.00040
16.61	0.71271	0.71252	0.00042
16.57	0.71313	0.71248	0.00042
16.53	0.71361	0.71243	0.00039
16.48	0.71296	0.71226	0.00030
16.44	0.71246	0.71218	0.00026
16.39	0.71201	0.71223	0.00030
16.35	0.71126	0.71230	0.00031
16.31	0.71252	0.71247	0.00023
16.26	0.71251	0.71242	0.00025
16.22	0.71202	0.71259	0.00044
16.18	0.71233	0.71262	0.00043
16.13	0.71262	0.71272	0.00044
16.09	0.71190	0.71270	0.00045
16.05	0.71219	0.71272	0.00043

28.54	0.71248	0.71220	0.00019
28.50	0.71188	0.71219	0.00019
28.45	0.71243	0.71218	0.00019
28.41	0.71238	0.71214	0.00018
28.37	0.71275	0.71215	0.00018
28.32	0.71226	0.71206	0.00013
28.28	0.71183	0.71212	0.00019
28.24	0.71199	0.71215	0.00018
28.19	0.71190	0.71219	0.00018
28.15	0.71212	0.71224	0.00017
28.11	0.71231	0.71222	0.00018
28.06	0.71185	0.71221	0.00018
28.02	0.71204	0.71228	0.00017
27.98	0.71240	0.71225	0.00020
27.93	0.71193	0.71223	0.00020
27.89	0.71278	0.71225	0.00019
27.85	0.71220	0.71214	0.00018
27.80	0.71239	0.71210	0.00019
27.76	0.71235	0.71198	0.00025
27.72	0.71197	0.71191	0.00024
27.67	0.71218	0.71194	0.00025
27.63	0.71257	0.71195	0.00025
27.59	0.71170	0.71192	0.00023
27.54	0.71223	0.71204	0.00030
27.50	0.71214	0.71200	0.00029
27.46	0.71169	0.71205	0.00031
27.41	0.71181	0.71209	0.00030
27.37	0.71121	0.71211	0.00030
27.33	0.71166	0.71223	0.00023
27.28	0.71220	0.71226	0.00020
27.24	0.71229	0.71223	0.00021
27.20	0.71223	0.71223	0.00021
27.15	0.71293	0.71220	0.00022
27.11	0.71186	0.71212	0.00015
27.07	0.71257	0.71218	0.00016
27.02	0.71212	0.71214	0.00013
26.98	0.71204	0.71207	0.00020
26.94	0.71243	0.71201	0.00024
26.89	0.71192	0.71194	0.00022
26.85	0.71192	0.71194	0.00022
26.81	0.71227	0.71199	0.00024
26.76	0.71189	0.71192	0.00024
26.72	0.71212	0.71194	0.00024
26.68	0.71249	0.71189	0.00024
26.63	0.71218	0.71185	0.00020
26.59	0.71142	0.71190	0.00026
26.55	0.71142	0.71198	0.00025
26.50	0.71175	0.71204	0.00021
26.46	0.71198	0.71203	0.00022
26.42	0.71237	0.71199	0.00024

16.00	0.71298	0.71273	0.00043
15.96	0.71264	0.71259	0.00048
15.91	0.71300	0.71256	0.00048
15.87	0.71202	0.71254	0.00048
15.83	0.71426	0.71263	0.00047
15.78	0.71232	0.71241	0.00031
15.74	0.71328	0.71244	0.00032
15.70	0.71243	0.71234	0.00026
15.65	0.71213	0.71238	0.00028
15.61	0.71226	0.71247	0.00031
15.56	0.71156	0.71244	0.00032
15.52	0.71235	0.71250	0.00027
15.48	0.71285	0.71259	0.00031
15.43	0.71290	0.71240	0.00044
15.39	0.71200	0.71233	0.00043
15.35	0.71268	0.71237	0.00042
15.30	0.71220	0.71232	0.00042
15.26	0.71286	0.71233	0.00042
15.21	0.71309	0.71222	0.00042
15.17	0.71194	0.71218	0.00038
15.13	0.71209	0.71223	0.00038
15.08	0.71330	0.71229	0.00040
15.04	0.71097	0.71226	0.00036
15.00	0.71217	0.71255	0.00037
14.95	0.71240	0.71264	0.00037
14.91	0.71222	0.71268	0.00037
14.86	0.71229	0.71271	0.00036
14.82	0.71170	0.71276	0.00034
14.78	0.71267	0.71280	0.00029
14.73	0.71245	0.71283	0.00029
14.69	0.71277	0.71284	0.00029
14.65	0.71299	0.71271	0.00040
14.60	0.71387	0.71270	0.00039
14.56	0.71301	0.71254	0.00030
14.52	0.71283	0.71246	0.00028
14.47	0.71254	0.71244	0.00027
14.43	0.71282	0.71245	0.00028
14.38	0.71210	0.71249	0.00031
14.34	0.71294	0.71245	0.00035
14.30	0.71253	0.71236	0.00034
14.25	0.71148	0.71249	0.00045
14.21	0.71282	0.71255	0.00040
14.17	0.71230	0.71245	0.00042
14.12	0.71221	0.71260	0.00050
14.08	0.71262	0.71261	0.00050
14.03	0.71266	0.71268	0.00052
13.99	0.71325	0.71259	0.00056
13.95	0.71168	0.71247	0.00054
13.90	0.71207	0.71256	0.00051
13.86	0.71385	0.71257	0.00051

26.37	0.71162	0.71205	0.00031
26.33	0.71204	0.71207	0.00030
26.29	0.71167	0.71206	0.00030
26.24	0.71204	0.71221	0.00036
26.20	0.71274	0.71229	0.00038
26.15	0.71218	0.71216	0.00040
26.11	0.71204	0.71214	0.00041
26.07	0.71162	0.71215	0.00041
26.02	0.71154	0.71217	0.00040
25.98	0.71300	0.71216	0.00040
25.94	0.71188	0.71213	0.00038
25.89	0.71188	0.71216	0.00037
25.85	0.71320	0.71222	0.00037
25.81	0.71287	0.71212	0.00031
25.76	0.71141	0.71206	0.00026
25.72	0.71201	0.71214	0.00022
25.68	0.71205	0.71226	0.00030
25.63	0.71183	0.71231	0.00031
25.59	0.71144	0.71220	0.00044
25.55	0.71269	0.71226	0.00041
25.50	0.71224	0.71221	0.00040
25.46	0.71249	0.71219	0.00040
25.42	0.71212	0.71218	0.00040
25.37	0.71235	0.71214	0.00041
25.33	0.71213	0.71214	0.00041
25.29	0.71321	0.71214	0.00041
25.24	0.71263	0.71210	0.00037
25.20	0.71069	0.71200	0.00036
25.16	0.71206	0.71218	0.00021
25.11	0.71219	0.71217	0.00021
25.07	0.71202	0.71221	0.00023
25.03	0.71244	0.71219	0.00024
24.98	0.71172	0.71221	0.00025
24.94	0.71236	0.71231	0.00024
24.90	0.71212	0.71236	0.00027
24.85	0.71275	0.71232	0.00029
24.81	0.71167	0.71223	0.00029
24.77	0.71246	0.71232	0.00027
24.72	0.71202	0.71224	0.00030
24.68	0.71257	0.71228	0.00030
24.64	0.71183	0.71225	0.00029
24.59	0.71261	0.71229	0.00028
24.55	0.71266	0.71237	0.00035
24.51	0.71291	0.71241	0.00037
24.46	0.71174	0.71240	0.00037
24.42	0.71182	0.71243	0.00035
24.38	0.71261	0.71255	0.00034
24.33	0.71163	0.71253	0.00034
24.29	0.71240	0.71259	0.00029
24.25	0.71234	0.71265	0.00030

13.82	0.71206	0.71251	0.00045	24.20	0.71221	0.71266	0.00030
13.77	0.71185	0.71254	0.00044	24.16	0.71343	0.71275	0.00029
13.73	0.71380	0.71264	0.00041	24.12	0.71303	0.71264	0.00026
13.68	0.71225	0.71248	0.00033	24.07	0.71278	0.71261	0.00024
13.64	0.71336	0.71247	0.00033	24.03	0.71204	0.71261	0.00024
13.60	0.71169	0.71241	0.00028	23.99	0.71302	0.71262	0.00023
13.55	0.71210	0.71253	0.00025	23.94	0.71244	0.71259	0.00021
13.51	0.71252	0.71252	0.00026	23.90	0.71219	0.71264	0.00022
13.47	0.71225	0.71247	0.00028	23.86	0.71306	0.71270	0.00019
13.42	0.71320	0.71248	0.00028	23.81	0.71237	0.71262	0.00019
13.38	0.71238	0.71233	0.00026	23.77	0.71313	0.71256	0.00026
13.33	0.71289	0.71237	0.00027	23.73	0.71237	0.71256	0.00026
13.29	0.71219	0.71232	0.00025	23.68	0.71275	0.71261	0.00026
13.25	0.71212	0.71231	0.00025	23.64	0.71270	0.71261	0.00026
13.20	0.71272	0.71241	0.00029	23.60	0.71221	0.71260	0.00026
13.16	0.71297	0.71239	0.00029	23.55	0.71268	0.71263	0.00025
13.12	0.71194	0.71231	0.00026	23.51	0.71295	0.71267	0.00026
13.07	0.71206	0.71221	0.00037	23.47	0.71278	0.71269	0.00028
13.03	0.71229	0.71222	0.00037	23.42	0.71228	0.71272	0.00029
12.98	0.71176	0.71216	0.00038	23.38	0.71174	0.71274	0.00028
12.94	0.71280	0.71227	0.00040	23.34	0.71311	0.71282	0.00018
12.90	0.71234	0.71224	0.00038	23.29	0.71295	0.71276	0.00018
12.85	0.71208	0.71219	0.00039	23.25	0.71268	0.71275	0.00017
12.81	0.71310	0.71215	0.00040	23.21	0.71261	0.71267	0.00024
12.77	0.71255	0.71199	0.00036	23.16	0.71251	0.71279	0.00032
12.72	0.71216	0.71185	0.00038	23.12	0.71305	0.71279	0.00032
12.68	0.71097	0.71177	0.00038	23.08	0.71321	0.71273	0.00032
12.64	0.71217	0.71175	0.00040	23.03	0.71307	0.71270	0.00031
12.59	0.71164	0.71165	0.00040	22.99	0.71247	0.71274	0.00034
12.55	0.71287	0.71169	0.00041	22.95	0.71259	0.71283	0.00036
12.50	0.71252	0.71149	0.00034	22.90	0.71247	0.71291	0.00037
12.46	0.71182	0.71136	0.00026	22.86	0.71282	0.71297	0.00036
12.42	0.71169	0.71125	0.00026	22.82	0.71194	0.71294	0.00037
12.37	0.71152	0.71128	0.00029	22.77	0.71377	0.71311	0.00032
12.33	0.71113	0.71122	0.00029	22.73	0.71249	0.71306	0.00028
12.29	0.71140	0.71127	0.00030	22.68	0.71247	0.71312	0.00025
12.24	0.71073	0.71125	0.00030	22.64	0.71286	0.71317	0.00021
12.20	0.71120	0.71118	0.00037	22.60	0.71348	0.71316	0.00022
12.15	0.71201	0.71125	0.00041	22.55	0.71339	0.71311	0.00021
12.11	0.71092	0.71119	0.00037	22.51	0.71343	0.71307	0.00020
12.07	0.71118	0.71122	0.00037	22.47	0.71306	0.71303	0.00019
12.02	0.71070	0.71127	0.00038	22.42	0.71251	0.71306	0.00020
11.98	0.71195	0.71136	0.00036	22.38	0.71363	0.71309	0.00017
11.94	0.71093	0.71128	0.00034	22.34	0.71323	0.71299	0.00015
11.89	0.71163	0.71130	0.00034	22.29	0.71316	0.71294	0.00014
11.85	0.71119	0.71131	0.00034	22.25	0.71293	0.71292	0.00014
11.80	0.71004	0.71132	0.00034	22.21	0.71281	0.71294	0.00014
11.76	0.71198	0.71141	0.00021	22.16	0.71291	0.71296	0.00014
11.72	0.71135	0.71122	0.00032	22.12	0.71298	0.71297	0.00014
11.67	0.71122	0.71116	0.00033	22.08	0.71307	0.71290	0.00020

11.63	0.71170	0.71111	0.00034
11.59	0.71166	0.71122	0.00046
11.54	0.71113	0.71124	0.00048
11.50	0.71111	0.71130	0.00048
11.45	0.71175	0.71130	0.00048
11.41	0.71123	0.71132	0.00049
11.37	0.71101	0.71134	0.00049
11.32	0.70999	0.71139	0.00048
11.28	0.71077	0.71162	0.00040
11.24	0.71077	0.71172	0.00036
11.19	0.71277	0.71180	0.00029
11.15	0.71189	0.71172	0.00020
11.10	0.71170	0.71167	0.00021
11.06	0.71115	0.71166	0.00021
11.02	0.71187	0.71177	0.00021
10.97	0.71148	0.71174	0.00021
10.93	0.71146	0.71181	0.00022
10.89	0.71231	0.71184	0.00020
10.84	0.71175	0.71184	0.00021
10.80	0.71165	0.71187	0.00021
10.76	0.71198	0.71185	0.00022
10.71	0.71140	0.71183	0.00022
10.67	0.71151	0.71179	0.00027
10.62	0.71225	0.71181	0.00026
10.58	0.71163	0.71178	0.00024
10.54	0.71218	0.71172	0.00028
10.49	0.71174	0.71166	0.00027
10.45	0.71235	0.71162	0.00027
10.41	0.71198	0.71154	0.00021
10.36	0.71146	0.71149	0.00019
10.32	0.71179	0.71148	0.00019
10.27	0.71098	0.71139	0.00021
10.23	0.71175	0.71141	0.00019
10.19	0.71191	0.71139	0.00018
10.14	0.71103	0.71130	0.00015
10.10	0.71160	0.71132	0.00014
10.06	0.71140	0.71123	0.00017
10.01	0.71155	0.71118	0.00018
9.97	0.71143	0.71113	0.00016
9.92	0.71134	0.71111	0.00015
9.88	0.71096	0.71104	0.00016
9.84	0.71116	0.71112	0.00020
9.79	0.71154	0.71109	0.00021
9.75	0.71102	0.71106	0.00018
9.71	0.71121	0.71101	0.00021
9.66	0.71070	0.71104	0.00024
9.62	0.71090	0.71110	0.00023
9.57	0.71104	0.71114	0.00023
9.53	0.71124	0.71107	0.00028
9.49	0.71068	0.71106	0.00028

22.03	0.71339	0.71292	0.00021
21.99	0.71273	0.71290	0.00020
21.95	0.71263	0.71288	0.00020
21.90	0.71274	0.71282	0.00027
21.86	0.71301	0.71282	0.00027
21.82	0.71308	0.71277	0.00027
21.77	0.71304	0.71269	0.00027
21.73	0.71306	0.71273	0.00030
21.69	0.71224	0.71276	0.00032
21.64	0.71326	0.71277	0.00032
21.60	0.71318	0.71281	0.00035
21.56	0.71261	0.71284	0.00037
21.51	0.71197	0.71284	0.00036
21.47	0.71273	0.71293	0.00031
21.43	0.71250	0.71295	0.00031
21.38	0.71235	0.71293	0.00032
21.34	0.71339	0.71292	0.00033
21.30	0.71338	0.71279	0.00035
21.25	0.71237	0.71275	0.00033
21.21	0.71365	0.71278	0.00032
21.17	0.71345	0.71260	0.00030
21.12	0.71264	0.71240	0.00031
21.08	0.71282	0.71230	0.00034
21.04	0.71295	0.71228	0.00033
20.99	0.71233	0.71215	0.00031
20.95	0.71225	0.71210	0.00032
20.91	0.71207	0.71205	0.00032
20.86	0.71298	0.71213	0.00036
20.82	0.71262	0.71203	0.00031
20.78	0.71185	0.71200	0.00029
20.73	0.71148	0.71211	0.00034
20.69	0.71162	0.71217	0.00031
20.65	0.71260	0.71224	0.00028
20.60	0.71167	0.71216	0.00028
20.56	0.71188	0.71219	0.00026
20.52	0.71169	0.71226	0.00026
20.47	0.71286	0.71239	0.00027
20.43	0.71198	0.71242	0.00030
20.39	0.71241	0.71241	0.00030
20.34	0.71288	0.71243	0.00031
20.30	0.71212	0.71240	0.00030
20.26	0.71231	0.71242	0.00029
20.21	0.71184	0.71243	0.00029
20.17	0.71194	0.71249	0.00026
20.13	0.71257	0.71254	0.00023
20.08	0.71296	0.71249	0.00025
20.04	0.71318	0.71237	0.00026
20.00	0.71186	0.71220	0.00025
19.95	0.71262	0.71223	0.00024
19.91	0.71264	0.71216	0.00023

9.44	0.71167	0.71110	0.00027
9.40	0.71095	0.71096	0.00028
9.36	0.71120	0.71097	0.00028
9.31	0.71051	0.71100	0.00030
9.27	0.71155	0.71105	0.00027
9.22	0.71129	0.71103	0.00026
9.18	0.71126	0.71099	0.00026
9.14	0.71031	0.71104	0.00029
9.09	0.71120	0.71113	0.00025
9.05	0.71105	0.71119	0.00028
9.01	0.71028	0.71129	0.00033
8.96	0.71107	0.71138	0.00024
8.92	0.71148	0.71142	0.00024
8.88	0.71097	0.71136	0.00026
8.83	0.71139	0.71139	0.00025
8.79	0.71091	0.71151	0.00034
8.74	0.71173	0.71160	0.00032
8.70	0.71125	0.71156	0.00032
8.66	0.71174	0.71162	0.00031
8.61	0.71209	0.71157	0.00032
8.57	0.71112	0.71152	0.00030
8.53	0.71154	0.71155	0.00029
8.48	0.71086	0.71143	0.00037
8.44	0.71126	0.71144	0.00036
8.39	0.71257	0.71141	0.00038
8.35	0.71183	0.71127	0.00028
8.31	0.71139	0.71121	0.00025
8.26	0.71179	0.71115	0.00026
8.22	0.71124	0.71109	0.00022
8.18	0.71160	0.71109	0.00022
8.13	0.71139	0.71097	0.00023
8.09	0.71039	0.71100	0.00026
8.04	0.71097	0.71114	0.00027
8.00	0.71091	0.71120	0.00028
7.96	0.71118	0.71126	0.00027
7.91	0.71125	0.71121	0.00029
7.87	0.71079	0.71127	0.00032
7.83	0.71120	0.71141	0.00035
7.78	0.71125	0.71133	0.00040
7.74	0.71037	0.71135	0.00041
7.69	0.71171	0.71145	0.00034
7.65	0.71181	0.71133	0.00038
7.61	0.71154	0.71127	0.00036
7.56	0.71149	0.71127	0.00036
7.52	0.71073	0.71122	0.00036
7.48	0.71182	0.71123	0.00036
7.43	0.71221	0.71119	0.00033
7.39	0.71037	0.71110	0.00025
7.34	0.71149	0.71119	0.00019
7.30	0.71127	0.71117	0.00018
19.87	0.71230	0.71214	0.00022
19.82	0.71234	0.71214	0.00021
19.78	0.71248	0.71211	0.00021
19.74	0.71246	0.71200	0.00023
19.69	0.71203	0.71194	0.00021
19.65	0.71176	0.71203	0.00028
19.61	0.71147	0.71208	0.00028
19.56	0.71217	0.71212	0.00025
19.52	0.71194	0.71207	0.00026
19.48	0.71249	0.71219	0.00034
19.43	0.71222	0.71222	0.00036
19.39	0.71203	0.71216	0.00038
19.35	0.71144	0.71207	0.00044
19.30	0.71186	0.71217	0.00042
19.26	0.71287	0.71219	0.00042
19.21	0.71232	0.71212	0.00039
19.17	0.71184	0.71202	0.00041
19.13	0.71169	0.71205	0.00041
19.08	0.71318	0.71202	0.00043
19.04	0.71278	0.71198	0.00038
19.00	0.71159	0.71188	0.00034
18.95	0.71108	0.71194	0.00033
18.91	0.71254	0.71203	0.00027
18.87	0.71200	0.71205	0.00029
18.82	0.71218	0.71209	0.00030
18.78	0.71137	0.71206	0.00030
18.74	0.71215	0.71207	0.00029
18.69	0.71137	0.71204	0.00029
18.65	0.71271	0.71208	0.00026
18.61	0.71183	0.71209	0.00027
18.56	0.71219	0.71214	0.00026
18.52	0.71199	0.71209	0.00028
18.48	0.71267	0.71210	0.00027
18.43	0.71245	0.71198	0.00027
18.39	0.71187	0.71190	0.00025
18.35	0.71149	0.71189	0.00026
18.30	0.71180	0.71195	0.00024
18.26	0.71182	0.71192	0.00025
18.22	0.71280	0.71197	0.00026
18.17	0.71231	0.71184	0.00020
18.13	0.71174	0.71179	0.00018
18.09	0.71205	0.71184	0.00019
18.04	0.71150	0.71188	0.00023
18.00	0.71159	0.71197	0.00023
17.96	0.71180	0.71202	0.00022
17.91	0.71209	0.71201	0.00022
17.87	0.71155	0.71200	0.00022
17.83	0.71228	0.71207	0.00020
17.78	0.71146	0.71200	0.00022
17.74	0.71188	0.71204	0.00019

7.26	0.71055	0.71117	0.00018	17.70	0.71216	0.71204	0.00019
7.21	0.71127	0.71115	0.00022	17.65	0.71248	0.71202	0.00019
7.17	0.71152	0.71111	0.00022	17.61	0.71239	0.71190	0.00021
7.13	0.71096	0.71111	0.00023	17.57	0.71210	0.71186	0.00018
7.08	0.71087	0.71116	0.00023	17.52	0.71166	0.71184	0.00017
7.04	0.71135	0.71117	0.00023	17.48	0.71201	0.71189	0.00017
7.00	0.71130	0.71112	0.00023	17.44	0.71226	0.71181	0.00021
6.95	0.71129	0.71108	0.00023	17.39	0.71159	0.71183	0.00024
6.91	0.71135	0.71115	0.00030	17.35	0.71184	0.71186	0.00023
6.86	0.71127	0.71113	0.00029	17.31	0.71186	0.71178	0.00028
6.82	0.71033	0.71110	0.00029	17.26	0.71199	0.71176	0.00028
6.78	0.71087	0.71124	0.00026	17.22	0.71128	0.71177	0.00029
6.73	0.71154	0.71140	0.00034	17.18	0.71203	0.71186	0.00027
6.69	0.71145	0.71142	0.00035	17.13	0.71191	0.71185	0.00027
6.65	0.71096	0.71140	0.00035	17.09	0.71208	0.71187	0.00028
6.60	0.71086	0.71140	0.00035	17.05	0.71125	0.71188	0.00028
6.56	0.71085	0.71140	0.00035	17.00	0.71247	0.71198	0.00025
6.51	0.71200	0.71142	0.00034	16.96	0.71185	0.71186	0.00026
6.47	0.71121	0.71139	0.00032	16.92	0.71105	0.71191	0.00028
6.43	0.71095	0.71137	0.00033	16.87	0.71167	0.71201	0.00021
6.38	0.71168	0.71136	0.00033	16.83	0.71211	0.71203	0.00020
6.34	0.71247	0.71129	0.00033	16.79	0.71216	0.71201	0.00020
6.30	0.71178	0.71121	0.00023	16.74	0.71193	0.71205	0.00022
6.25	0.71120	0.71115	0.00019	16.70	0.71216	0.71202	0.00024
6.21	0.71097	0.71113	0.00019	16.66	0.71213	0.71196	0.00025
6.16	0.71091	0.71113	0.00019	16.61	0.71230	0.71195	0.00025
6.12	0.71104	0.71122	0.00022	16.57	0.71127	0.71200	0.00029
6.08	0.71169	0.71123	0.00022	16.53	0.71237	0.71199	0.00030
6.03	0.71102	0.71125	0.00023	16.48	0.71203	0.71199	0.00030
5.99	0.71085	0.71133	0.00025	16.44	0.71182	0.71199	0.00030
5.95	0.71097	0.71133	0.00025	16.40	0.71194	0.71208	0.00033
5.90	0.71165	0.71140	0.00025	16.35	0.71255	0.71218	0.00036
5.86	0.71119	0.71137	0.00025	16.31	0.71162	0.71209	0.00036
5.81	0.71104	0.71132	0.00028	16.27	0.71162	0.71212	0.00035
5.77	0.71098	0.71135	0.00027	16.22	0.71203	0.71215	0.00034
5.73	0.71177	0.71139	0.00026	16.18	0.71277	0.71219	0.00034
5.68	0.71114	0.71136	0.00025	16.14	0.71115	0.71213	0.00032
5.64	0.71187	0.71138	0.00024	16.09	0.71234	0.71225	0.00023
5.60	0.71179	0.71136	0.00023	16.05	0.71210	0.71225	0.00023
5.55	0.71085	0.71137	0.00024	16.01	0.71273	0.71234	0.00027
5.51	0.71177	0.71147	0.00022	15.96	0.71285	0.71230	0.00026
5.46	0.71132	0.71160	0.00039	15.92	0.71173	0.71228	0.00024
5.42	0.71069	0.71165	0.00039	15.88	0.71191	0.71233	0.00021
5.38	0.71135	0.71177	0.00032	15.83	0.71185	0.71239	0.00019
5.33	0.71133	0.71183	0.00031	15.79	0.71243	0.71233	0.00027
5.29	0.71149	0.71179	0.00034	15.75	0.71225	0.71235	0.00028
5.25	0.71135	0.71183	0.00034	15.70	0.71233	0.71234	0.00028
5.20	0.71167	0.71187	0.00032	15.66	0.71235	0.71233	0.00028
5.16	0.71187	0.71194	0.00033	15.61	0.71299	0.71228	0.00030
5.12	0.71187	0.71185	0.00038	15.57	0.71234	0.71221	0.00026

5.07	0.71306	0.71175	0.00042
5.03	0.71184	0.71167	0.00033
4.98	0.71187	0.71157	0.00036
4.94	0.71195	0.71151	0.00036
4.90	0.71096	0.71143	0.00035
4.85	0.71181	0.71148	0.00033
4.81	0.71179	0.71145	0.00032
4.77	0.71233	0.71151	0.00036
4.72	0.71101	0.71139	0.00032
4.68	0.71091	0.71137	0.00033
4.63	0.71220	0.71137	0.00033
4.59	0.71091	0.71128	0.00027
4.55	0.71121	0.71126	0.00029
4.50	0.71120	0.71128	0.00029
4.46	0.71142	0.71128	0.00029
4.42	0.71157	0.71128	0.00029
4.37	0.71231	0.71125	0.00030
4.33	0.71119	0.71117	0.00020
4.28	0.71078	0.71118	0.00020
4.24	0.71092	0.71123	0.00019
4.20	0.71133	0.71126	0.00017
4.15	0.71070	0.71129	0.00018
4.11	0.71143	0.71139	0.00010
4.07	0.71118	0.71150	0.00024
4.02	0.71142	0.71157	0.00022
3.98	0.71160	0.71147	0.00026
3.93	0.71123	0.71148	0.00026
3.89	0.71121	0.71151	0.00024
3.85	0.71157	0.71154	0.00023
3.80	0.71148	0.71157	0.00023
3.76	0.71234	0.71153	0.00025
3.72	0.71169	0.71147	0.00018
3.67	0.71099	0.71152	0.00023
3.63	0.71112	0.71156	0.00020
3.59	0.71168	0.71167	0.00021
3.54	0.71151	0.71171	0.00022
3.50	0.71147	0.71175	0.00022
3.45	0.71159	0.71176	0.00022
3.41	0.71180	0.71182	0.00022
3.37	0.71112	0.71181	0.00022
3.32	0.71174	0.71189	0.00016
3.28	0.71219	0.71189	0.00017
3.24	0.71141	0.71192	0.00020
3.19	0.71218	0.71195	0.00017
3.15	0.71206	0.71192	0.00016
3.10	0.71193	0.71189	0.00016
3.06	0.71160	0.71186	0.00017
3.02	0.71212	0.71194	0.00018
2.97	0.71178	0.71200	0.00024
2.93	0.71190	0.71206	0.00025

15.53	0.71264	0.71217	0.00026
15.48	0.71222	0.71213	0.00024
15.44	0.71247	0.71205	0.00028
15.40	0.71129	0.71202	0.00026
15.35	0.71264	0.71209	0.00021
15.31	0.71211	0.71203	0.00017
15.27	0.71230	0.71202	0.00017
15.22	0.71180	0.71198	0.00016
15.18	0.71229	0.71203	0.00016
15.14	0.71197	0.71198	0.00016
15.09	0.71219	0.71199	0.00016
15.05	0.71140	0.71199	0.00016
15.01	0.71224	0.71211	0.00013
14.96	0.71199	0.71200	0.00022
14.92	0.71201	0.71204	0.00023
14.88	0.71204	0.71204	0.00023
14.83	0.71187	0.71202	0.00024
14.79	0.71225	0.71206	0.00024
14.75	0.71187	0.71203	0.00024
14.70	0.71205	0.71205	0.00024
14.66	0.71220	0.71209	0.00025
14.62	0.71253	0.71201	0.00028
14.57	0.71118	0.71192	0.00026
14.53	0.71239	0.71202	0.00021
14.49	0.71202	0.71196	0.00019
14.44	0.71179	0.71190	0.00023
14.40	0.71228	0.71196	0.00025
14.36	0.71198	0.71201	0.00029
14.31	0.71211	0.71206	0.00031
14.27	0.71241	0.71210	0.00032
14.23	0.71137	0.71202	0.00033
14.18	0.71170	0.71203	0.00032
14.14	0.71217	0.71208	0.00032
14.10	0.71180	0.71207	0.00032
14.05	0.71134	0.71209	0.00031
14.01	0.71247	0.71225	0.00030
13.97	0.71273	0.71228	0.00032
13.92	0.71252	0.71220	0.00031
13.88	0.71251	0.71211	0.00032
13.84	0.71157	0.71217	0.00037
13.79	0.71147	0.71225	0.00034
13.75	0.71225	0.71226	0.00034
13.71	0.71199	0.71231	0.00035
13.66	0.71203	0.71234	0.00035
13.62	0.71293	0.71238	0.00034
13.58	0.71279	0.71237	0.00034
13.53	0.71198	0.71237	0.00033
13.49	0.71162	0.71254	0.00042
13.45	0.71308	0.71268	0.00037
13.40	0.71238	0.71262	0.00036

2.89	0.71170	0.71210	0.00025
2.84	0.71251	0.71215	0.00023
2.80	0.71176	0.71212	0.00022
2.75	0.71184	0.71214	0.00021
2.71	0.71180	0.71210	0.00025
2.67	0.71162	0.71205	0.00029
2.62	0.71233	0.71213	0.00028
2.58	0.71273	0.71204	0.00030
2.54	0.71239	0.71195	0.00026
2.49	0.71231	0.71194	0.00025
2.45	0.71222	0.71184	0.00026
2.40	0.71219	0.71178	0.00025
2.36	0.71194	0.71171	0.00024
2.32	0.71141	0.71171	0.00024
2.27	0.71137	0.71160	0.00037
2.23	0.71236	0.71165	0.00037
2.19	0.71146	0.71163	0.00035
2.14	0.71183	0.71168	0.00035
2.10	0.71226	0.71171	0.00037
2.05	0.71132	0.71162	0.00035
2.01	0.71169	0.71165	0.00034
1.97	0.71142	0.71161	0.00035
1.92	0.71200	0.71167	0.00035
1.88	0.71032	0.71163	0.00035
1.84	0.71183	0.71186	0.00025
1.79	0.71213	0.71187	0.00025
1.75	0.71197	0.71177	0.00027
1.71	0.71214	0.71170	0.00029
1.66	0.71141	0.71169	0.00028
1.62	0.71157	0.71172	0.00027
1.57	0.71132	0.71167	0.00030
1.53	0.71201	0.71162	0.00033
1.49	0.71160	0.71157	0.00032
1.44	0.71260	0.71161	0.00034
1.40	0.71191	0.71150	0.00025
1.36	0.71119	0.71147	0.00024
1.31	0.71126	0.71161	0.00033
1.27	0.71198	0.71164	0.00032
1.22	0.71171	0.71162	0.00031
1.18	0.71109	0.71155	0.00033
1.14	0.71086	0.71157	0.00032
1.09	0.71148	0.71170	0.00029
1.05	0.71203	0.71169	0.00030
1.01	0.71153	0.71163	0.00029
0.96	0.71155	0.71156	0.00033
0.92	0.71265	0.71157	0.00033
0.87	0.71155	0.71138	0.00027
0.83	0.71175	0.71133	0.00027
0.79	0.71106	0.71124	0.00027
0.74	0.71129	0.71136	0.00034

13.36	0.71151	0.71262	0.00036
13.32	0.71280	0.71269	0.00028
13.27	0.71230	0.71262	0.00031
13.23	0.71244	0.71263	0.00030
13.19	0.71285	0.71257	0.00034
13.14	0.71272	0.71265	0.00039
13.10	0.71373	0.71270	0.00041
13.06	0.71299	0.71257	0.00034
13.01	0.71246	0.71247	0.00035
12.97	0.71245	0.71246	0.00035
12.93	0.71218	0.71244	0.00035
12.88	0.71209	0.71248	0.00035
12.84	0.71236	0.71244	0.00038
12.80	0.71187	0.71249	0.00039
12.75	0.71361	0.71248	0.00040
12.71	0.71324	0.71237	0.00031
12.67	0.71248	0.71227	0.00024
12.62	0.71194	0.71232	0.00028
12.58	0.71236	0.71238	0.00026
12.54	0.71223	0.71234	0.00027
12.49	0.71264	0.71235	0.00027
12.45	0.71166	0.71232	0.00026
12.41	0.71288	0.71237	0.00022
12.36	0.71179	0.71234	0.00020
12.32	0.71247	0.71241	0.00016
12.28	0.71229	0.71241	0.00016
12.23	0.71295	0.71230	0.00030
12.19	0.71249	0.71221	0.00027
12.14	0.71203	0.71226	0.00031
12.10	0.71228	0.71232	0.00031
12.06	0.71232	0.71230	0.00031
12.01	0.71223	0.71234	0.00032
11.97	0.71259	0.71233	0.00032
11.93	0.71248	0.71248	0.00049
11.88	0.71246	0.71245	0.00049
11.84	0.71113	0.71254	0.00053
11.80	0.71213	0.71262	0.00045
11.75	0.71300	0.71264	0.00044
11.71	0.71259	0.71249	0.00049
11.67	0.71213	0.71250	0.00049
11.62	0.71264	0.71245	0.00052
11.58	0.71212	0.71238	0.00053
11.54	0.71417	0.71232	0.00055
11.49	0.71216	0.71210	0.00037
11.45	0.71337	0.71213	0.00038
11.41	0.71193	0.71211	0.00035
11.36	0.71231	0.71220	0.00038
11.32	0.71144	0.71217	0.00038
11.28	0.71270	0.71224	0.00034
11.23	0.71161	0.71223	0.00033

0.70	0.71215	0.71142	0.00035
0.66	0.71134	0.71138	0.00032
0.61	0.71141	0.71140	0.00032
0.57	0.71084	0.71133	0.00036
0.52	0.71167	0.71141	0.00034
0.48	0.71073	0.71142	0.00035
0.44	0.71110	0.71145	0.00032
0.39	0.71083	0.71149	0.00033
0.35	0.71227	0.71157	0.00032
0.31	0.71182	0.71147	0.00028
0.26	0.71180	0.71141	0.00029
0.22	0.71157	0.71134	0.00030
0.17	0.71067	0.71128	0.00033
0.13	0.71168	0.71148	0.00025
0.09	0.71174	0.71139	0.00032
0.04	0.71103		

11.19	0.71199	0.71232	0.00031
11.15	0.71158	0.71235	0.00030
11.10	0.71188	0.71233	0.00033
11.06	0.71246	0.71236	0.00031
11.02	0.71318	0.71231	0.00032
10.97	0.71287	0.71210	0.00034
10.93	0.71203	0.71202	0.00029
10.89	0.71212	0.71192	0.00035
10.84	0.71257	0.71187	0.00035
10.80	0.71252	0.71182	0.00032
10.76	0.71234	0.71177	0.00029
10.71	0.71136	0.71181	0.00032
10.67	0.71212	0.71190	0.00032
10.63	0.71199	0.71187	0.00032
10.58	0.71113	0.71187	0.00032
10.54	0.71205	0.71204	0.00032
10.50	0.71102	0.71202	0.00032
10.45	0.71164	0.71210	0.00024
10.41	0.71204	0.71216	0.00021
10.37	0.71205	0.71221	0.00022
10.32	0.71265	0.71208	0.00037
10.28	0.71232	0.71194	0.00037
10.24	0.71177	0.71190	0.00036
10.19	0.71208	0.71190	0.00036
10.15	0.71278	0.71182	0.00038
10.11	0.71183	0.71165	0.00034
10.06	0.71188	0.71161	0.00034
10.02	0.71222	0.71155	0.00034
9.98	0.71251	0.71144	0.00031
9.93	0.71077	0.71142	0.00028
9.89	0.71129	0.71149	0.00024
9.85	0.71186	0.71157	0.00026
9.80	0.71181	0.71155	0.00025
9.76	0.71126	0.71165	0.00036
9.72	0.71105	0.71167	0.00035
9.67	0.71141	0.71172	0.00033
9.63	0.71134	0.71173	0.00032
9.59	0.71113	0.71171	0.00033
9.54	0.71226	0.71181	0.00032
9.50	0.71152	0.71176	0.00030
9.46	0.71205	0.71179	0.00030
9.41	0.71165	0.71178	0.00029
9.37	0.71284	0.71172	0.00033
9.33	0.71148	0.71152	0.00026
9.28	0.71146	0.71155	0.00026
9.24	0.71157	0.71153	0.00027
9.20	0.71113	0.71155	0.00027
9.15	0.71213	0.71170	0.00033
9.11	0.71171	0.71163	0.00032
9.07	0.71188	0.71165	0.00032

9.02	0.71194	0.71168	0.00034
8.98	0.71100	0.71154	0.00041
8.94	0.71093	0.71157	0.00039
8.89	0.71175	0.71168	0.00038
8.85	0.71124	0.71170	0.00038
8.81	0.71180	0.71180	0.00038
8.76	0.71260	0.71190	0.00043
8.72	0.71143	0.71177	0.00042
8.67	0.71193	0.71185	0.00042
8.63	0.71223	0.71187	0.00042
8.59	0.71050	0.71190	0.00044
8.54	0.71128	0.71205	0.00031
8.50	0.71208	0.71216	0.00026
8.46	0.71195	0.71217	0.00026
8.41	0.71222	0.71202	0.00044
8.37	0.71282	0.71199	0.00044
8.33	0.71126	0.71196	0.00042
8.28	0.71224	0.71190	0.00048
8.24	0.71209	0.71188	0.00047
8.20	0.71255	0.71183	0.00047
8.15	0.71199	0.71169	0.00046
8.11	0.71236	0.71162	0.00046
8.07	0.71225	0.71160	0.00045
8.02	0.71041	0.71152	0.00043
7.98	0.71188	0.71153	0.00041
7.94	0.71260	0.71154	0.00042
7.89	0.71067	0.71150	0.00038
7.85	0.71200	0.71161	0.00033
7.81	0.71157	0.71147	0.00037
7.76	0.71112	0.71146	0.00036
7.72	0.71138	0.71150	0.00036
7.68	0.71216	0.71142	0.00040
7.63	0.71138	0.71140	0.00039
7.59	0.71053	0.71156	0.00051
7.55	0.71199	0.71159	0.00048
7.50	0.71218	0.71152	0.00048
7.46	0.71177	0.71150	0.00047
7.42	0.71067	0.71141	0.00048
7.37	0.71147	0.71137	0.00051
7.33	0.71151	0.71137	0.00051
7.29	0.71055	0.71135	0.00051
7.24	0.71193	0.71146	0.00048
7.20	0.71302	0.71141	0.00047
7.16	0.71081	0.71120	0.00031
7.11	0.71133	0.71130	0.00033
7.07	0.71199	0.71137	0.00036
7.03	0.71082	0.71137	0.00036
6.98	0.71028	0.71143	0.00034
6.94	0.71150	0.71161	0.00024
6.90	0.71130	0.71161	0.00024

6.85	0.71165	0.71167	0.00024
6.81	0.71139	0.71167	0.00024
6.77	0.71089	0.71161	0.00029
6.72	0.71189	0.71165	0.00026
6.68	0.71203	0.71142	0.00048
6.64	0.71196	0.71143	0.00049
6.59	0.71142	0.71140	0.00048
6.55	0.71206	0.71142	0.00048
6.51	0.71149	0.71143	0.00048
6.46	0.71193	0.71145	0.00048
6.42	0.71162	0.71143	0.00048
6.38	0.71077	0.71153	0.00054
6.33	0.71133	0.71158	0.00052
6.29	0.70959	0.71161	0.00052
6.25	0.71218	0.71176	0.00029
6.20	0.71161	0.71159	0.00037
6.16	0.71166	0.71160	0.00037
6.12	0.71208	0.71158	0.00038
6.07	0.71173	0.71159	0.00038
6.03	0.71170	0.71170	0.00045
5.99	0.71269	0.71159	0.00050
5.94	0.71120	0.71150	0.00044
5.90	0.71163	0.71152	0.00044
5.86	0.71110	0.71158	0.00046
5.81	0.71047	0.71169	0.00046
5.77	0.71176	0.71177	0.00038
5.73	0.71145	0.71178	0.00038
5.68	0.71220	0.71180	0.00038
5.64	0.71281	0.71169	0.00039
5.60	0.71064	0.71163	0.00032
5.55	0.71171	0.71167	0.00028
5.51	0.71147	0.71164	0.00028
5.47	0.71221	0.71163	0.00028
5.42	0.71217	0.71181	0.00054
5.38	0.71131	0.71176	0.00053
5.34	0.71186	0.71178	0.00053
5.29	0.71162	0.71172	0.00054
5.25	0.71111	0.71172	0.00054
5.20	0.71219	0.71180	0.00052
5.16	0.71104	0.71180	0.00052
5.12	0.71140	0.71180	0.00052
5.07	0.71141	0.71180	0.00052
5.03	0.71395	0.71174	0.00055
4.99	0.71171	0.71137	0.00035
4.94	0.71155	0.71127	0.00037
4.90	0.71117	0.71133	0.00040
4.86	0.71172	0.71134	0.00040
4.81	0.71182	0.71126	0.00040
4.77	0.71221	0.71120	0.00038
4.73	0.71105	0.71113	0.00032

4.68	0.71138	0.71123	0.00037
4.64	0.71086	0.71130	0.00041
4.60	0.71027	0.71132	0.00040
4.55	0.71069	0.71139	0.00034
4.51	0.71213	0.71149	0.00031
4.47	0.71133	0.71145	0.00028
4.42	0.71088	0.71141	0.00030
4.38	0.71123	0.71144	0.00029
4.34	0.71152	0.71145	0.00028
4.29	0.71201	0.71149	0.00029
4.25	0.71214	0.71147	0.00029
4.21	0.71103	0.71144	0.00026
4.16	0.71093	0.71142	0.00028
4.12	0.71170	0.71145	0.00027
4.08	0.71175	0.71138	0.00027
4.03	0.71088	0.71127	0.00029
3.99	0.71123	0.71139	0.00031
3.95	0.71132	0.71126	0.00043
3.90	0.71187	0.71128	0.00043
3.86	0.71190	0.71118	0.00042
3.82	0.71184	0.71098	0.00044
3.77	0.71075	0.71087	0.00040
3.73	0.71124	0.71100	0.00046
3.69	0.71103	0.71103	0.00048
3.64	0.71068	0.71097	0.00049
3.60	0.71203	0.71107	0.00050
3.56	0.70994	0.71105	0.00048
3.51	0.71155	0.71117	0.00042
3.47	0.71080	0.71123	0.00045
3.43	0.70999	0.71130	0.00044
3.38	0.71070	0.71147	0.00034
3.34	0.71203	0.71158	0.00030
3.30	0.71157	0.71150	0.00029
3.25	0.71046	0.71152	0.00029
3.21	0.71168	0.71161	0.00018
3.17	0.71176	0.71156	0.00020
3.12	0.71118	0.71154	0.00019
3.08	0.71208	0.71144	0.00032
3.04	0.71151	0.71127	0.00036
2.99	0.71175	0.71126	0.00036
2.95	0.71181	0.71118	0.00034
2.91	0.71120	0.71112	0.00031
2.86	0.71175	0.71114	0.00032
2.82	0.71136	0.71113	0.00031
2.78	0.71123	0.71108	0.00031
2.73	0.71153	0.71115	0.00036
2.69	0.71023	0.71116	0.00037
2.65	0.71030	0.71131	0.00032
2.60	0.71143	0.71137	0.00025
2.56	0.71100	0.71128	0.00030

2.52	0.71116	0.71136	0.00031
2.47	0.71145	0.71142	0.00032
2.43	0.71164	0.71149	0.00035
2.39	0.71081	0.71141	0.00038
2.34	0.71194	0.71151	0.00036
2.30	0.71170	0.71148	0.00035
2.26	0.71172	0.71144	0.00034
2.21	0.71083	0.71136	0.00035
2.17	0.71054	0.71141	0.00033
2.13	0.71178	0.71143	0.00031
2.08	0.71177	0.71134	0.00031
2.04	0.71222	0.71129	0.00030
2.00	0.71079	0.71109	0.00029
1.95	0.71179	0.71112	0.00028
1.91	0.71162	0.71100	0.00025
1.87	0.71134	0.71101	0.00025
1.82	0.71094	0.71095	0.00024
1.78	0.71131	0.71099	0.00025
1.73	0.71074	0.71097	0.00024
1.69	0.71093	0.71097	0.00024
1.65	0.71119	0.71102	0.00025
1.60	0.71024	0.71107	0.00029
1.56	0.71109	0.71114	0.00023
1.52	0.71064	0.71118	0.00025
1.47	0.71163	0.71116	0.00026
1.43	0.71082	0.71106	0.00026
1.39	0.71128	0.71107	0.00026
1.34	0.71110	0.71100	0.00027
1.30	0.71081	0.71105	0.00030
1.26	0.71137	0.71112	0.00030
1.21	0.71176	0.71111	0.00030
1.17	0.71088	0.71111	0.00030
1.13	0.71152	0.71121	0.00033
1.08	0.71046	0.71112	0.00034
1.04	0.71060	0.71119	0.00031
1.00	0.71096	0.71118	0.00032
0.95	0.71056	0.71130	0.00037
0.91	0.71159	0.71135	0.00033
0.87	0.71145	0.71132	0.00033
0.82	0.71135	0.71122	0.00038
0.78	0.71170	0.71126	0.00039
0.74	0.71191	0.71119	0.00038
0.69	0.71064	0.71120	0.00039
0.65	0.71115	0.71124	0.00037
0.61	0.71049	0.71125	0.00037
0.56	0.71216	0.71132	0.00033
0.52	0.71108	0.71125	0.00028
0.48	0.71130	0.71125	0.00028
0.43	0.71040	0.71118	0.00030
0.39	0.71172	0.71127	0.00026

0.35	0.71103	0.71122	0.00026
0.30	0.71204	0.71124	0.00027
0.26	0.71103	0.71111	0.00017
0.22	0.71125	0.71113	0.00019
0.17	0.71118	0.71109	0.00021
0.13	0.71142	0.71107	0.00025
0.09	0.71115	0.71089	0.00023
0.04	0.71063		

ARB 118.2.1 (M2)

Distance from cervix (mm)	$^{87}Sr/^{86}Sr$	10 point mov. average	2 SE on mov. average
32.64	0.70864	0.70845	0.00011
32.60	0.70820	0.70845	0.00012
32.55	0.70843	0.70847	0.00011
32.51	0.70864	0.70847	0.00011
32.46	0.70860	0.70848	0.00012
32.42	0.70842	0.70849	0.00012
32.38	0.70821	0.70854	0.00015
32.33	0.70833	0.70861	0.00015
32.29	0.70867	0.70862	0.00014
32.24	0.70832	0.70861	0.00014
32.20	0.70868	0.70866	0.00012
32.16	0.70837	0.70867	0.00013
32.11	0.70843	0.70869	0.00011
32.07	0.70874	0.70871	0.00009
32.03	0.70870	0.70867	0.00012
31.98	0.70897	0.70865	0.00013
31.94	0.70886	0.70860	0.00011
31.89	0.70848	0.70857	0.00009
31.85	0.70857	0.70859	0.00010
31.81	0.70883	0.70862	0.00011
31.76	0.70874	0.70860	0.00010
31.72	0.70862	0.70858	0.00010
31.67	0.70861	0.70855	0.00012
31.63	0.70831	0.70856	0.00012
31.59	0.70847	0.70859	0.00011
31.54	0.70856	0.70858	0.00011
31.50	0.70847	0.70858	0.00012
31.45	0.70877	0.70858	0.00012
31.41	0.70887	0.70853	0.00012
31.37	0.70858	0.70853	0.00011
31.32	0.70855	0.70850	0.00012
31.28	0.70827	0.70848	0.00012
31.24	0.70873	0.70855	0.00014
31.19	0.70864	0.70850	0.00015
31.15	0.70837	0.70851	0.00015
31.10	0.70852	0.70854	0.00015
31.06	0.70848	0.70856	0.00015
31.02	0.70830	0.70860	0.00017
30.97	0.70882	0.70862	0.00016
30.93	0.70830	0.70865	0.00019
30.88	0.70839	0.70872	0.00018
30.84	0.70896	0.70874	0.00017
30.80	0.70827	0.70873	0.00017
30.75	0.70869	0.70880	0.00014
30.71	0.70871	0.70883	0.00014
30.67	0.70866	0.70885	0.00014
30.62	0.70891	0.70886	0.00014

ARB 118.3.1 (M3)

Distance from cervix (mm)	$^{87}Sr/^{86}Sr$	10 point mov. average	2 SE on mov. average
32.57	0.70872	0.70874	0.00017
32.53	0.70824	0.70872	0.00018
32.49	0.70850	0.70878	0.00015
32.44	0.70905	0.70875	0.00018
32.40	0.70894	0.70871	0.00017
32.36	0.70872	0.70872	0.00018
32.32	0.70845	0.70873	0.00018
32.27	0.70902	0.70874	0.00018
32.23	0.70897	0.70868	0.00018
32.19	0.70884	0.70861	0.00018
32.15	0.70844	0.70855	0.00019
32.10	0.70885	0.70858	0.00020
32.06	0.70822	0.70860	0.00020
32.02	0.70864	0.70861	0.00019
31.98	0.70910	0.70865	0.00020
31.93	0.70883	0.70862	0.00018
31.89	0.70854	0.70862	0.00018
31.85	0.70840	0.70869	0.00021
31.80	0.70827	0.70871	0.00020
31.76	0.70820	0.70880	0.00019
31.72	0.70880	0.70886	0.00014
31.68	0.70896	0.70885	0.00015
31.63	0.70840	0.70881	0.00016
31.59	0.70896	0.70882	0.00015
31.55	0.70885	0.70877	0.00015
31.51	0.70883	0.70874	0.00016
31.46	0.70918	0.70872	0.00016
31.42	0.70869	0.70869	0.00014
31.38	0.70916	0.70871	0.00014
31.33	0.70879	0.70869	0.00011
31.29	0.70871	0.70864	0.00013
31.25	0.70849	0.70864	0.00013
31.21	0.70850	0.70864	0.00012
31.16	0.70855	0.70864	0.00012
31.12	0.70850	0.70867	0.00012
31.08	0.70860	0.70870	0.00012
31.04	0.70890	0.70870	0.00013
30.99	0.70888	0.70871	0.00013
30.95	0.70894	0.70868	0.00013
30.91	0.70838	0.70867	0.00012
30.87	0.70864	0.70872	0.00011
30.82	0.70851	0.70872	0.00011
30.78	0.70854	0.70875	0.00010
30.74	0.70881	0.70874	0.00011
30.69	0.70884	0.70869	0.00014
30.65	0.70852	0.70864	0.00015
30.61	0.70899	0.70865	0.00014

30.58	0.70847	0.70882	0.00015
30.53	0.70916	0.70883	0.00014
30.49	0.70903	0.70878	0.00012
30.45	0.70858	0.70874	0.00011
30.40	0.70882	0.70879	0.00012
30.36	0.70893	0.70875	0.00015
30.31	0.70906	0.70875	0.00016
30.27	0.70885	0.70871	0.00014
30.23	0.70878	0.70872	0.00015
30.18	0.70856	0.70870	0.00015
30.14	0.70857	0.70873	0.00015
30.09	0.70862	0.70879	0.00017
30.05	0.70865	0.70880	0.00016
30.01	0.70908	0.70887	0.00019
29.96	0.70836	0.70882	0.00019
29.92	0.70903	0.70883	0.00018
29.88	0.70858	0.70880	0.00017
29.83	0.70899	0.70880	0.00017
29.79	0.70856	0.70876	0.00017
29.74	0.70888	0.70877	0.00017
29.70	0.70913	0.70875	0.00017
29.66	0.70877	0.70872	0.00015
29.61	0.70929	0.70873	0.00015
29.57	0.70862	0.70873	0.00016
29.52	0.70843	0.70873	0.00015
29.48	0.70876	0.70878	0.00014
29.44	0.70858	0.70881	0.00015
29.39	0.70863	0.70884	0.00014
29.35	0.70857	0.70892	0.00018
29.31	0.70876	0.70890	0.00021
29.26	0.70882	0.70894	0.00021
29.22	0.70881	0.70897	0.00021
29.17	0.70934	0.70898	0.00021
29.13	0.70865	0.70889	0.00022
29.09	0.70894	0.70890	0.00021
29.04	0.70897	0.70886	0.00023
29.00	0.70890	0.70887	0.00023
28.95	0.70948	0.70884	0.00024
28.91	0.70832	0.70883	0.00022
28.87	0.70917	0.70888	0.00019
28.82	0.70908	0.70882	0.00018
28.78	0.70897	0.70878	0.00018
28.73	0.70844	0.70877	0.00017
28.69	0.70876	0.70879	0.00016
28.65	0.70849	0.70878	0.00017
28.60	0.70912	0.70883	0.00016
28.56	0.70860	0.70881	0.00015
28.52	0.70932	0.70881	0.00015
28.47	0.70881	0.70876	0.00010
28.43	0.70864	0.70873	0.00011

30.57	0.70865	0.70861	0.00012
30.52	0.70885	0.70862	0.00012
30.48	0.70886	0.70858	0.00012
30.44	0.70860	0.70854	0.00010
30.40	0.70880	0.70855	0.00010
30.35	0.70844	0.70855	0.00010
30.31	0.70829	0.70859	0.00011
30.27	0.70841	0.70862	0.00009
30.22	0.70863	0.70864	0.00008
30.18	0.70857	0.70859	0.00011
30.14	0.70875	0.70863	0.00013
30.10	0.70849	0.70861	0.00013
30.05	0.70845	0.70861	0.00013
30.01	0.70864	0.70862	0.00013
29.97	0.70881	0.70864	0.00013
29.93	0.70882	0.70864	0.00013
29.88	0.70859	0.70860	0.00014
29.84	0.70860	0.70863	0.00015
29.80	0.70822	0.70863	0.00015
29.76	0.70892	0.70869	0.00013
29.71	0.70852	0.70871	0.00014
29.67	0.70858	0.70875	0.00015
29.63	0.70851	0.70875	0.00015
29.58	0.70886	0.70876	0.00014
29.54	0.70880	0.70871	0.00016
29.50	0.70836	0.70872	0.00016
29.46	0.70892	0.70877	0.00014
29.41	0.70857	0.70873	0.00014
29.37	0.70888	0.70871	0.00016
29.33	0.70907	0.70869	0.00016
29.29	0.70898	0.70871	0.00018
29.24	0.70856	0.70866	0.00018
29.20	0.70859	0.70868	0.00018
29.16	0.70841	0.70867	0.00018
29.11	0.70881	0.70869	0.00017
29.07	0.70892	0.70864	0.00018
29.03	0.70852	0.70861	0.00017
28.99	0.70831	0.70862	0.00017
28.94	0.70875	0.70866	0.00015
28.90	0.70926	0.70864	0.00015
28.86	0.70843	0.70861	0.00010
28.82	0.70875	0.70865	0.00010
28.77	0.70854	0.70862	0.00010
28.73	0.70858	0.70865	0.00011
28.69	0.70837	0.70868	0.00011
28.65	0.70860	0.70874	0.00010
28.60	0.70860	0.70873	0.00011
28.56	0.70873	0.70874	0.00011
28.52	0.70857	0.70872	0.00012
28.47	0.70894	0.70872	0.00012

28.38	0.70866	0.70875	0.00012
28.34	0.70888	0.70876	0.00012
28.30	0.70865	0.70875	0.00012
28.25	0.70860	0.70879	0.00013
28.21	0.70905	0.70882	0.00012
28.16	0.70887	0.70884	0.00014
28.12	0.70860	0.70884	0.00014
28.08	0.70887	0.70884	0.00014
28.03	0.70845	0.70880	0.00016
27.99	0.70892	0.70880	0.00016
27.95	0.70867	0.70883	0.00019
27.90	0.70883	0.70886	0.00019
27.86	0.70903	0.70888	0.00019
27.81	0.70889	0.70877	0.00025
27.77	0.70925	0.70872	0.00026
27.73	0.70889	0.70869	0.00024
27.68	0.70863	0.70865	0.00024
27.64	0.70840	0.70866	0.00024
27.59	0.70846	0.70872	0.00024
27.55	0.70929	0.70875	0.00024
27.51	0.70895	0.70869	0.00021
27.46	0.70898	0.70867	0.00020
27.42	0.70801	0.70862	0.00019
27.37	0.70838	0.70869	0.00013
27.33	0.70893	0.70880	0.00019
27.29	0.70848	0.70881	0.00019
27.24	0.70870	0.70886	0.00018
27.20	0.70906	0.70882	0.00021
27.16	0.70870	0.70876	0.00021
27.11	0.70874	0.70875	0.00021
27.07	0.70870	0.70878	0.00022
27.02	0.70854	0.70885	0.00025
26.98	0.70867	0.70884	0.00026
26.94	0.70950	0.70882	0.00026
26.89	0.70896	0.70871	0.00023
26.85	0.70902	0.70870	0.00023
26.80	0.70831	0.70865	0.00022
26.76	0.70847	0.70867	0.00021
26.72	0.70860	0.70871	0.00021
26.67	0.70902	0.70870	0.00021
26.63	0.70941	0.70872	0.00022
26.59	0.70845	0.70864	0.00015
26.54	0.70849	0.70869	0.00016
26.50	0.70832	0.70870	0.00015
26.45	0.70887	0.70876	0.00013
26.41	0.70859	0.70878	0.00014
26.37	0.70852	0.70877	0.00015
26.32	0.70881	0.70875	0.00016
26.28	0.70857	0.70876	0.00016
26.23	0.70914	0.70875	0.00016

28.43	0.70879	0.70870	0.00011
28.39	0.70850	0.70865	0.00013
28.35	0.70880	0.70869	0.00013
28.30	0.70889	0.70865	0.00014
28.26	0.70896	0.70868	0.00017
28.22	0.70849	0.70870	0.00019
28.18	0.70876	0.70876	0.00020
28.13	0.70845	0.70873	0.00021
28.09	0.70860	0.70875	0.00020
28.05	0.70874	0.70870	0.00024
28.00	0.70836	0.70869	0.00024
27.96	0.70890	0.70871	0.00023
27.92	0.70837	0.70868	0.00023
27.88	0.70915	0.70870	0.00022
27.83	0.70919	0.70866	0.00020
27.79	0.70908	0.70863	0.00016
27.75	0.70844	0.70859	0.00013
27.71	0.70870	0.70858	0.00014
27.66	0.70810	0.70858	0.00014
27.62	0.70861	0.70863	0.00010
27.58	0.70853	0.70864	0.00010
27.54	0.70861	0.70869	0.00012
27.49	0.70856	0.70869	0.00012
27.45	0.70877	0.70869	0.00012
27.41	0.70886	0.70867	0.00012
27.36	0.70873	0.70867	0.00012
27.32	0.70829	0.70870	0.00015
27.28	0.70871	0.70879	0.00014
27.24	0.70860	0.70878	0.00014
27.19	0.70876	0.70884	0.00016
27.15	0.70898	0.70884	0.00016
27.11	0.70858	0.70882	0.00015
27.07	0.70865	0.70889	0.00016
27.02	0.70848	0.70890	0.00016
26.98	0.70889	0.70888	0.00018
26.94	0.70906	0.70897	0.00025
26.89	0.70914	0.70890	0.00028
26.85	0.70866	0.70890	0.00028
26.81	0.70920	0.70891	0.00027
26.77	0.70874	0.70886	0.00027
26.72	0.70883	0.70890	0.00027
26.68	0.70924	0.70887	0.00029
26.64	0.70875	0.70884	0.00027
26.60	0.70834	0.70884	0.00027
26.55	0.70975	0.70892	0.00026
26.51	0.70831	0.70883	0.00018
26.47	0.70914	0.70888	0.00014
26.43	0.70878	0.70887	0.00013
26.38	0.70874	0.70886	0.00014
26.34	0.70913	0.70885	0.00014

26.19	0.70866	0.70873	0.00014	26.30	0.70847	0.70883	0.00013
26.15	0.70895	0.70875	0.00014	26.25	0.70895	0.70884	0.00011
26.10	0.70860	0.70870	0.00015	26.21	0.70879	0.70880	0.00012
26.06	0.70893	0.70870	0.00015	26.17	0.70919	0.70871	0.00021
26.01	0.70902	0.70873	0.00018	26.13	0.70885	0.70870	0.00020
25.97	0.70850	0.70873	0.00018	26.08	0.70878	0.70865	0.00021
25.93	0.70837	0.70877	0.00017	26.04	0.70903	0.70861	0.00021
25.88	0.70883	0.70880	0.00015	26.00	0.70871	0.70863	0.00023
25.84	0.70854	0.70873	0.00019	25.96	0.70866	0.70858	0.00025
25.80	0.70893	0.70877	0.00019	25.91	0.70884	0.70856	0.00025
25.75	0.70883	0.70874	0.00019	25.87	0.70863	0.70855	0.00024
25.71	0.70840	0.70871	0.00019	25.83	0.70854	0.70851	0.00025
25.66	0.70868	0.70877	0.00018	25.78	0.70792	0.70849	0.00025
25.62	0.70923	0.70877	0.00018	25.74	0.70905	0.70856	0.00021
25.58	0.70898	0.70875	0.00017	25.70	0.70838	0.70854	0.00020
25.53	0.70887	0.70871	0.00017	25.66	0.70839	0.70861	0.00022
25.49	0.70867	0.70867	0.00016	25.61	0.70922	0.70863	0.00021
25.44	0.70818	0.70866	0.00017	25.57	0.70818	0.70857	0.00017
25.40	0.70890	0.70873	0.00013	25.53	0.70850	0.70863	0.00014
25.36	0.70865	0.70872	0.00013	25.49	0.70864	0.70863	0.00014
25.31	0.70858	0.70873	0.00013	25.44	0.70824	0.70861	0.00015
25.27	0.70899	0.70874	0.00012	25.40	0.70837	0.70868	0.00013
25.23	0.70864	0.70872	0.00011	25.36	0.70866	0.70872	0.00011
25.18	0.70907	0.70870	0.00013	25.32	0.70883	0.70873	0.00011
25.14	0.70852	0.70866	0.00010	25.27	0.70905	0.70872	0.00011
25.09	0.70852	0.70868	0.00009	25.23	0.70858	0.70871	0.00010
25.05	0.70854	0.70867	0.00009	25.19	0.70867	0.70876	0.00012
25.01	0.70890	0.70874	0.00014	25.14	0.70874	0.70879	0.00012
24.96	0.70877	0.70872	0.00014	25.10	0.70852	0.70878	0.00013
24.92	0.70874	0.70868	0.00015	25.06	0.70846	0.70882	0.00012
24.87	0.70868	0.70866	0.00015	25.02	0.70890	0.70880	0.00015
24.83	0.70878	0.70870	0.00018	24.97	0.70877	0.70875	0.00017
24.79	0.70842	0.70875	0.00021	24.93	0.70876	0.70874	0.00017
24.74	0.70872	0.70880	0.00020	24.89	0.70877	0.70875	0.00017
24.70	0.70868	0.70879	0.00020	24.85	0.70897	0.70873	0.00018
24.65	0.70847	0.70886	0.00023	24.80	0.70905	0.70866	0.00020
24.61	0.70922	0.70901	0.00030	24.76	0.70897	0.70863	0.00018
24.57	0.70867	0.70896	0.00030	24.72	0.70862	0.70866	0.00021
24.52	0.70843	0.70897	0.00029	24.67	0.70896	0.70867	0.00021
24.48	0.70850	0.70902	0.00027	24.63	0.70822	0.70864	0.00020
24.44	0.70916	0.70910	0.00025	24.59	0.70838	0.70871	0.00019
24.39	0.70926	0.70911	0.00025	24.55	0.70871	0.70871	0.00018
24.35	0.70893	0.70905	0.00026	24.50	0.70889	0.70872	0.00018
24.30	0.70862	0.70911	0.00028	24.46	0.70857	0.70868	0.00018
24.26	0.70931	0.70919	0.00026	24.42	0.70819	0.70870	0.00018
24.22	0.70996	0.70917	0.00026	24.38	0.70882	0.70877	0.00015
24.17	0.70880	0.70914	0.00022	24.33	0.70927	0.70880	0.00016
24.13	0.70871	0.70920	0.00021	24.29	0.70868	0.70880	0.00015
24.08	0.70893	0.70921	0.00020	24.25	0.70866	0.70885	0.00017
24.04	0.70934	0.70917	0.00024	24.21	0.70888	0.70888	0.00017

24.00	0.70924	0.70912	0.00024
23.95	0.70869	0.70919	0.00029
23.91	0.70951	0.70923	0.00027
23.87	0.70943	0.70911	0.00032
23.82	0.70910	0.70898	0.00035
23.78	0.70966	0.70887	0.00041
23.73	0.70936	0.70880	0.00037
23.69	0.70884	0.70876	0.00035
23.65	0.70851	0.70868	0.00037
23.60	0.70889	0.70875	0.00038
23.56	0.70991	0.70873	0.00038
23.51	0.70907	0.70864	0.00028
23.47	0.70828	0.70857	0.00027
23.43	0.70821	0.70865	0.00028
23.38	0.70794	0.70875	0.00028
23.34	0.70894	0.70884	0.00021
23.29	0.70896	0.70879	0.00022
23.25	0.70812	0.70872	0.00025
23.21	0.70914	0.70880	0.00021
23.16	0.70874	0.70876	0.00019
23.12	0.70894	0.70874	0.00020
23.08	0.70842	0.70868	0.00020
23.03	0.70909	0.70869	0.00020
22.99	0.70919	0.70860	0.00020
22.94	0.70883	0.70855	0.00016
22.90	0.70848	0.70857	0.00018
22.86	0.70822	0.70863	0.00020
22.81	0.70892	0.70865	0.00019
22.77	0.70875	0.70859	0.00019
22.72	0.70858	0.70864	0.00024
22.68	0.70837	0.70868	0.00024
22.64	0.70843	0.70876	0.00025
22.59	0.70821	0.70879	0.00024
22.55	0.70874	0.70884	0.00021
22.51	0.70906	0.70890	0.00023
22.46	0.70906	0.70881	0.00027
22.42	0.70842	0.70873	0.00029
22.37	0.70827	0.70870	0.00031
22.33	0.70926	0.70871	0.00030
22.29	0.70897	0.70866	0.00027
22.24	0.70920	0.70869	0.00029
22.20	0.70868	0.70859	0.00028
22.15	0.70875	0.70859	0.00028
22.11	0.70935	0.70855	0.00028
22.07	0.70811	0.70847	0.00022
22.02	0.70825	0.70856	0.00023
21.98	0.70812	0.70862	0.00022
21.94	0.70841	0.70868	0.00019
21.89	0.70874	0.70869	0.00019
21.85	0.70924	0.70868	0.00019

24.16	0.70843	0.70887	0.00017
24.12	0.70881	0.70889	0.00015
24.08	0.70853	0.70893	0.00017
24.03	0.70870	0.70894	0.00015
23.99	0.70895	0.70895	0.00015
23.95	0.70913	0.70888	0.00020
23.91	0.70918	0.70883	0.00020
23.86	0.70923	0.70879	0.00018
23.82	0.70901	0.70874	0.00016
23.78	0.70875	0.70875	0.00016
23.74	0.70861	0.70877	0.00017
23.69	0.70922	0.70881	0.00017
23.65	0.70866	0.70874	0.00015
23.61	0.70871	0.70879	0.00017
23.57	0.70828	0.70881	0.00017
23.52	0.70866	0.70884	0.00013
23.48	0.70879	0.70885	0.00013
23.44	0.70873	0.70882	0.00015
23.39	0.70910	0.70885	0.00015
23.35	0.70891	0.70882	0.00014
23.31	0.70901	0.70877	0.00017
23.27	0.70856	0.70869	0.00018
23.22	0.70917	0.70868	0.00019
23.18	0.70885	0.70865	0.00016
23.14	0.70859	0.70857	0.00020
23.10	0.70877	0.70860	0.00021
23.05	0.70847	0.70863	0.00022
23.01	0.70903	0.70865	0.00022
22.97	0.70886	0.70862	0.00021
22.92	0.70835	0.70855	0.00021
22.88	0.70827	0.70855	0.00021
22.84	0.70845	0.70860	0.00021
22.80	0.70886	0.70859	0.00022
22.75	0.70804	0.70855	0.00021
22.71	0.70889	0.70868	0.00022
22.67	0.70905	0.70866	0.00021
22.63	0.70870	0.70861	0.00020
22.58	0.70872	0.70857	0.00020
22.54	0.70820	0.70858	0.00020
22.50	0.70832	0.70875	0.00031
22.46	0.70880	0.70882	0.00030
22.41	0.70833	0.70887	0.00032
22.37	0.70847	0.70890	0.00031
22.33	0.70928	0.70890	0.00030
22.28	0.70872	0.70883	0.00030
22.24	0.70857	0.70883	0.00030
22.20	0.70833	0.70887	0.00029
22.16	0.70874	0.70894	0.00027
22.11	0.70988	0.70891	0.00029
22.07	0.70906	0.70876	0.00020

21.80	0.70826	0.70866	0.00016	22.03	0.70934	0.70877	0.00021
21.76	0.70863	0.70865	0.00017	21.99	0.70856	0.70870	0.00017
21.72	0.70837	0.70866	0.00017	21.94	0.70855	0.70869	0.00017
21.67	0.70853	0.70873	0.00017	21.90	0.70853	0.70872	0.00017
21.63	0.70901	0.70875	0.00017	21.86	0.70873	0.70873	0.00017
21.58	0.70884	0.70867	0.00018	21.81	0.70897	0.70877	0.00018
21.54	0.70879	0.70869	0.00019	21.77	0.70903	0.70868	0.00023
21.50	0.70848	0.70872	0.00020	21.73	0.70842	0.70862	0.00022
21.45	0.70861	0.70872	0.00021	21.69	0.70842	0.70868	0.00023
21.41	0.70902	0.70868	0.00023	21.64	0.70917	0.70871	0.00022
21.36	0.70823	0.70869	0.00024	21.60	0.70858	0.70873	0.00024
21.32	0.70872	0.70869	0.00023	21.56	0.70849	0.70876	0.00024
21.28	0.70907	0.70875	0.00026	21.52	0.70888	0.70884	0.00025
21.23	0.70872	0.70876	0.00027	21.47	0.70865	0.70880	0.00026
21.19	0.70825	0.70878	0.00027	21.43	0.70909	0.70882	0.00025
21.15	0.70902	0.70881	0.00025	21.39	0.70802	0.70882	0.00026
21.10	0.70904	0.70877	0.00025	21.35	0.70843	0.70893	0.00019
21.06	0.70847	0.70878	0.00025	21.30	0.70902	0.70892	0.00020
21.01	0.70820	0.70879	0.00024	21.26	0.70878	0.70882	0.00026
20.97	0.70914	0.70882	0.00022	21.22	0.70935	0.70876	0.00029
20.93	0.70829	0.70877	0.00021	21.17	0.70887	0.70865	0.00028
20.88	0.70929	0.70881	0.00018	21.13	0.70927	0.70868	0.00029
20.84	0.70917	0.70874	0.00015	21.09	0.70854	0.70862	0.00026
20.79	0.70889	0.70873	0.00014	21.05	0.70883	0.70863	0.00026
20.75	0.70863	0.70875	0.00015	21.00	0.70912	0.70860	0.00025
20.71	0.70858	0.70871	0.00018	20.96	0.70909	0.70857	0.00023
20.66	0.70911	0.70879	0.00023	20.92	0.70831	0.70851	0.00020
20.62	0.70862	0.70878	0.00022	20.88	0.70808	0.70852	0.00020
20.58	0.70846	0.70877	0.00022	20.83	0.70818	0.70861	0.00019
20.53	0.70862	0.70879	0.00021	20.79	0.70824	0.70867	0.00017
20.49	0.70872	0.70885	0.00023	20.75	0.70909	0.70874	0.00015
20.44	0.70861	0.70887	0.00023	20.70	0.70873	0.70873	0.00014
20.40	0.70904	0.70891	0.00022	20.66	0.70867	0.70877	0.00017
20.36	0.70910	0.70885	0.00023	20.62	0.70849	0.70876	0.00017
20.31	0.70823	0.70884	0.00023	20.58	0.70883	0.70881	0.00016
20.27	0.70940	0.70888	0.00020	20.53	0.70848	0.70882	0.00016
20.22	0.70894	0.70878	0.00018	20.49	0.70840	0.70887	0.00015
20.18	0.70858	0.70876	0.00018	20.45	0.70899	0.70897	0.00015
20.14	0.70861	0.70878	0.00017	20.41	0.70873	0.70898	0.00015
20.09	0.70923	0.70881	0.00017	20.36	0.70901	0.70894	0.00018
20.05	0.70899	0.70876	0.00014	20.32	0.70897	0.70890	0.00020
20.00	0.70894	0.70877	0.00015	20.28	0.70917	0.70887	0.00020
19.96	0.70844	0.70873	0.00016	20.24	0.70857	0.70885	0.00019
19.92	0.70907	0.70877	0.00015	20.19	0.70896	0.70894	0.00021
19.87	0.70858	0.70875	0.00014	20.15	0.70892	0.70892	0.00022
19.83	0.70844	0.70880	0.00014	20.11	0.70899	0.70892	0.00022
19.79	0.70870	0.70884	0.00012	20.06	0.70944	0.70893	0.00022
19.74	0.70883	0.70886	0.00011	20.02	0.70900	0.70885	0.00020
19.70	0.70888	0.70882	0.00014	19.98	0.70838	0.70883	0.00019
19.65	0.70871	0.70877	0.00017	19.94	0.70859	0.70890	0.00017

19.61	0.70914	0.70874	0.00018
19.57	0.70848	0.70867	0.00017
19.52	0.70891	0.70873	0.00019
19.48	0.70886	0.70873	0.00018
19.43	0.70905	0.70866	0.00021
19.39	0.70885	0.70863	0.00020
19.35	0.70892	0.70859	0.00020
19.30	0.70844	0.70853	0.00019
19.26	0.70837	0.70853	0.00019
19.22	0.70839	0.70862	0.00023
19.17	0.70842	0.70866	0.00023
19.13	0.70914	0.70870	0.00022
19.08	0.70882	0.70868	0.00021
19.04	0.70816	0.70868	0.00021
19.00	0.70883	0.70873	0.00017
18.95	0.70840	0.70871	0.00017
18.91	0.70827	0.70875	0.00016
18.86	0.70853	0.70884	0.00015
18.82	0.70919	0.70890	0.00014
18.78	0.70884	0.70891	0.00014
18.73	0.70881	0.70896	0.00017
18.69	0.70893	0.70899	0.00016
18.64	0.70889	0.70894	0.00019
18.60	0.70865	0.70889	0.00023
18.56	0.70863	0.70894	0.00023
18.51	0.70873	0.70892	0.00025
18.47	0.70923	0.70892	0.00025
18.43	0.70912	0.70890	0.00024
18.38	0.70927	0.70883	0.00025
18.34	0.70934	0.70877	0.00023
18.29	0.70908	0.70873	0.00020
18.25	0.70849	0.70871	0.00018
18.21	0.70833	0.70876	0.00019
18.16	0.70920	0.70883	0.00017
18.12	0.70838	0.70881	0.00016
18.07	0.70878	0.70885	0.00013
18.03	0.70898	0.70882	0.00014
17.99	0.70847	0.70878	0.00015
17.94	0.70862	0.70882	0.00013
17.90	0.70899	0.70889	0.00015
17.86	0.70880	0.70887	0.00015
17.81	0.70909	0.70883	0.00017
17.77	0.70898	0.70886	0.00019
17.72	0.70903	0.70889	0.00021
17.68	0.70876	0.70885	0.00022
17.64	0.70850	0.70884	0.00022
17.59	0.70860	0.70884	0.00022
17.55	0.70886	0.70886	0.00021
17.50	0.70930	0.70885	0.00021
17.46	0.70878	0.70880	0.00019

19.89	0.70870	0.70892	0.00016
19.85	0.70900	0.70892	0.00016
19.81	0.70946	0.70892	0.00016
19.77	0.70872	0.70889	0.00013
19.72	0.70890	0.70884	0.00018
19.68	0.70912	0.70884	0.00018
19.64	0.70866	0.70886	0.00019
19.59	0.70876	0.70885	0.00020
19.55	0.70913	0.70890	0.00021
19.51	0.70875	0.70888	0.00020
19.47	0.70868	0.70886	0.00021
19.42	0.70900	0.70891	0.00022
19.38	0.70919	0.70890	0.00022
19.34	0.70824	0.70890	0.00022
19.30	0.70891	0.70899	0.00017
19.25	0.70925	0.70898	0.00017
19.21	0.70856	0.70895	0.00016
19.17	0.70923	0.70896	0.00015
19.13	0.70896	0.70892	0.00014
19.08	0.70859	0.70891	0.00014
19.04	0.70917	0.70898	0.00014
19.00	0.70885	0.70889	0.00019
18.95	0.70924	0.70889	0.00019
18.91	0.70917	0.70882	0.00018
18.87	0.70881	0.70881	0.00017
18.83	0.70893	0.70878	0.00018
18.78	0.70860	0.70879	0.00019
18.74	0.70892	0.70875	0.00022
18.70	0.70884	0.70872	0.00022
18.66	0.70924	0.70864	0.00025
18.61	0.70828	0.70862	0.00023
18.57	0.70888	0.70862	0.00023
18.53	0.70857	0.70862	0.00024
18.48	0.70903	0.70865	0.00024
18.44	0.70847	0.70859	0.00023
18.40	0.70906	0.70861	0.00023
18.36	0.70819	0.70859	0.00021
18.31	0.70861	0.70862	0.00019
18.27	0.70811	0.70864	0.00019
18.23	0.70905	0.70875	0.00018
18.19	0.70824	0.70873	0.00017
18.14	0.70893	0.70878	0.00013
18.10	0.70883	0.70874	0.00014
18.06	0.70840	0.70877	0.00017
18.02	0.70873	0.70884	0.00016
17.97	0.70879	0.70880	0.00020
17.93	0.70856	0.70878	0.00020
17.89	0.70881	0.70882	0.00019
17.84	0.70921	0.70880	0.00020
17.80	0.70880	0.70872	0.00019

17.42	0.70846	0.70886	0.00022	17.76	0.70877	0.70876	0.00021
17.37	0.70934	0.70891	0.00020	17.72	0.70848	0.70877	0.00021
17.33	0.70928	0.70884	0.00018	17.67	0.70919	0.70884	0.00021
17.28	0.70859	0.70881	0.00016	17.63	0.70912	0.70885	0.00022
17.24	0.70867	0.70884	0.00015	17.59	0.70827	0.70880	0.00021
17.20	0.70849	0.70882	0.00017	17.55	0.70864	0.70890	0.00019
17.15	0.70885	0.70882	0.00017	17.50	0.70893	0.70887	0.00020
17.11	0.70872	0.70884	0.00017	17.46	0.70861	0.70890	0.00021
17.07	0.70884	0.70886	0.00017	17.42	0.70843	0.70892	0.00020
17.02	0.70940	0.70887	0.00017	17.37	0.70915	0.70897	0.00017
16.98	0.70888	0.70882	0.00012	17.33	0.70890	0.70891	0.00019
16.93	0.70868	0.70880	0.00012	17.29	0.70921	0.70887	0.00021
16.89	0.70894	0.70885	0.00014	17.25	0.70921	0.70882	0.00020
16.85	0.70894	0.70888	0.00016	17.20	0.70867	0.70883	0.00020
16.80	0.70844	0.70885	0.00017	17.16	0.70921	0.70880	0.00021
16.76	0.70855	0.70885	0.00017	17.12	0.70842	0.70879	0.00020
16.71	0.70900	0.70890	0.00015	17.08	0.70919	0.70882	0.00019
16.67	0.70898	0.70890	0.00016	17.03	0.70880	0.70878	0.00017
16.63	0.70888	0.70889	0.00016	16.99	0.70894	0.70877	0.00017
16.58	0.70890	0.70888	0.00016	16.95	0.70856	0.70870	0.00019
16.54	0.70872	0.70887	0.00016	16.91	0.70842	0.70871	0.00018
16.50	0.70920	0.70891	0.00017	16.86	0.70878	0.70876	0.00017
16.45	0.70920	0.70886	0.00016	16.82	0.70925	0.70879	0.00019
16.41	0.70862	0.70878	0.00016	16.78	0.70845	0.70872	0.00018
16.36	0.70844	0.70885	0.00019	16.73	0.70912	0.70873	0.00017
16.32	0.70904	0.70884	0.00020	16.69	0.70873	0.70871	0.00015
16.28	0.70905	0.70882	0.00019	16.65	0.70873	0.70869	0.00015
16.23	0.70887	0.70873	0.00022	16.61	0.70869	0.70868	0.00015
16.19	0.70877	0.70871	0.00022	16.56	0.70831	0.70872	0.00016
16.14	0.70874	0.70872	0.00022	16.52	0.70867	0.70873	0.00015
16.10	0.70918	0.70869	0.00023	16.48	0.70883	0.70876	0.00015
16.06	0.70865	0.70870	0.00024	16.44	0.70918	0.70876	0.00015
16.01	0.70846	0.70876	0.00026	16.39	0.70846	0.70872	0.00012
15.97	0.70932	0.70879	0.00025	16.35	0.70863	0.70873	0.00011
15.92	0.70833	0.70875	0.00023	16.31	0.70883	0.70872	0.00012
15.88	0.70878	0.70878	0.00021	16.27	0.70855	0.70874	0.00012
15.84	0.70819	0.70878	0.00021	16.22	0.70868	0.70877	0.00012
15.79	0.70873	0.70880	0.00019	16.18	0.70903	0.70872	0.00018
15.75	0.70885	0.70884	0.00021	16.14	0.70847	0.70869	0.00017
15.71	0.70845	0.70886	0.00021	16.09	0.70890	0.70873	0.00016
15.66	0.70928	0.70892	0.00019	16.05	0.70885	0.70871	0.00016
15.62	0.70926	0.70886	0.00018	16.01	0.70877	0.70870	0.00016
15.57	0.70868	0.70882	0.00016	15.97	0.70857	0.70868	0.00016
15.53	0.70896	0.70887	0.00016	15.92	0.70857	0.70871	0.00016
15.49	0.70868	0.70894	0.00024	15.88	0.70898	0.70872	0.00016
15.44	0.70877	0.70895	0.00023	15.84	0.70892	0.70868	0.00015
15.40	0.70833	0.70898	0.00023	15.80	0.70812	0.70872	0.00018
15.35	0.70920	0.70903	0.00019	15.75	0.70870	0.70877	0.00013
15.31	0.70901	0.70898	0.00019	15.71	0.70891	0.70872	0.00017
15.27	0.70903	0.70903	0.00021	15.67	0.70873	0.70870	0.00016

15.22	0.70870	0.70907	0.00023	15.62	0.70874	0.70871	0.00017
15.18	0.70889	0.70906	0.00023	15.58	0.70855	0.70870	0.00017
15.14	0.70909	0.70910	0.00023	15.54	0.70890	0.70876	0.00018
15.09	0.70973	0.70912	0.00023	15.50	0.70865	0.70876	0.00018
15.05	0.70873	0.70904	0.00019	15.45	0.70860	0.70879	0.00018
15.00	0.70908	0.70907	0.00018	15.41	0.70925	0.70880	0.00018
14.96	0.70880	0.70906	0.00018	15.37	0.70863	0.70878	0.00016
14.92	0.70875	0.70911	0.00018	15.33	0.70824	0.70882	0.00016
14.87	0.70947	0.70917	0.00017	15.28	0.70874	0.70885	0.00012
14.83	0.70942	0.70909	0.00018	15.24	0.70885	0.70881	0.00016
14.78	0.70867	0.70902	0.00018	15.20	0.70863	0.70879	0.00016
14.74	0.70925	0.70903	0.00017	15.16	0.70911	0.70876	0.00018
14.70	0.70931	0.70900	0.00017	15.11	0.70895	0.70868	0.00018
14.65	0.70890	0.70897	0.00015	15.07	0.70890	0.70862	0.00018
14.61	0.70902	0.70896	0.00016	15.03	0.70875	0.70861	0.00018
14.56	0.70897	0.70893	0.00016	14.98	0.70900	0.70861	0.00017
14.52	0.70937	0.70895	0.00017	14.94	0.70907	0.70857	0.00015
14.48	0.70934	0.70889	0.00014	14.90	0.70852	0.70852	0.00010
14.43	0.70869	0.70883	0.00011	14.86	0.70835	0.70851	0.00010
14.39	0.70871	0.70882	0.00011	14.81	0.70857	0.70853	0.00010
14.35	0.70871	0.70885	0.00012	14.77	0.70839	0.70857	0.00013
14.30	0.70901	0.70887	0.00011	14.73	0.70829	0.70858	0.00013
14.26	0.70898	0.70886	0.00011	14.69	0.70839	0.70861	0.00011
14.21	0.70879	0.70884	0.00011	14.64	0.70879	0.70865	0.00011
14.17	0.70870	0.70881	0.00012	14.60	0.70872	0.70870	0.00016
14.13	0.70917	0.70884	0.00013	14.56	0.70857	0.70866	0.00019
14.08	0.70885	0.70882	0.00010	14.51	0.70857	0.70865	0.00019
14.04	0.70871	0.70879	0.00011	14.47	0.70850	0.70861	0.00022
13.99	0.70859	0.70880	0.00011	14.43	0.70852	0.70863	0.00022
13.95	0.70901	0.70880	0.00011	14.39	0.70898	0.70866	0.00022
13.91	0.70893	0.70881	0.00011	14.34	0.70846	0.70865	0.00021
13.86	0.70886	0.70878	0.00011	14.30	0.70862	0.70869	0.00022
13.82	0.70877	0.70876	0.00011	14.26	0.70881	0.70871	0.00022
13.78	0.70853	0.70871	0.00014	14.22	0.70929	0.70868	0.00022
13.73	0.70901	0.70873	0.00014	14.17	0.70823	0.70864	0.00018
13.69	0.70891	0.70874	0.00015	14.13	0.70854	0.70871	0.00016
13.64	0.70863	0.70873	0.00015	14.09	0.70813	0.70868	0.00019
13.60	0.70876	0.70873	0.00015	14.05	0.70868	0.70875	0.00014
13.56	0.70864	0.70873	0.00015	14.00	0.70887	0.70874	0.00015
13.51	0.70902	0.70879	0.00018	13.96	0.70884	0.70871	0.00014
13.47	0.70863	0.70879	0.00018	13.92	0.70892	0.70868	0.00014
13.42	0.70867	0.70884	0.00019	13.87	0.70884	0.70867	0.00014
13.38	0.70829	0.70887	0.00018	13.83	0.70851	0.70870	0.00016
13.34	0.70870	0.70890	0.00015	13.79	0.70885	0.70873	0.00015
13.29	0.70916	0.70887	0.00017	13.75	0.70892	0.70873	0.00015
13.25	0.70882	0.70884	0.00016	13.70	0.70823	0.70869	0.00015
13.20	0.70856	0.70892	0.00023	13.66	0.70885	0.70877	0.00012
13.16	0.70882	0.70899	0.00022	13.62	0.70854	0.70874	0.00013
13.12	0.70925	0.70896	0.00024	13.58	0.70862	0.70874	0.00013
13.07	0.70899	0.70890	0.00023	13.53	0.70856	0.70875	0.00012

13.03	0.70909	0.70886	0.00024
12.99	0.70897	0.70879	0.00025
12.94	0.70859	0.70881	0.00026
12.90	0.70845	0.70881	0.00026
12.85	0.70880	0.70887	0.00025
12.81	0.70964	0.70888	0.00025
12.77	0.70926	0.70880	0.00019
12.72	0.70851	0.70878	0.00017
12.68	0.70867	0.70884	0.00016
12.63	0.70863	0.70888	0.00017
12.59	0.70836	0.70887	0.00017
12.55	0.70919	0.70889	0.00014
12.50	0.70861	0.70881	0.00016
12.46	0.70896	0.70883	0.00015
12.42	0.70896	0.70882	0.00015
12.37	0.70885	0.70876	0.00017
12.33	0.70909	0.70874	0.00017
12.28	0.70904	0.70873	0.00017
12.24	0.70905	0.70868	0.00016
12.20	0.70863	0.70867	0.00015
12.15	0.70853	0.70867	0.00015
12.11	0.70839	0.70868	0.00015
12.06	0.70878	0.70872	0.00013
12.02	0.70889	0.70869	0.00014
11.98	0.70837	0.70860	0.00020
11.93	0.70859	0.70865	0.00020
11.89	0.70904	0.70868	0.00020
11.84	0.70853	0.70870	0.00022
11.80	0.70896	0.70867	0.00023
11.76	0.70866	0.70867	0.00023
11.71	0.70860	0.70863	0.00024
11.67	0.70882	0.70868	0.00026
11.63	0.70845	0.70868	0.00026
11.58	0.70796	0.70877	0.00028
11.54	0.70894	0.70889	0.00023
11.49	0.70888	0.70884	0.00024
11.45	0.70918	0.70881	0.00025
11.41	0.70830	0.70879	0.00024
11.36	0.70889	0.70889	0.00024
11.32	0.70828	0.70888	0.00024
11.27	0.70911	0.70888	0.00023
11.23	0.70876	0.70886	0.00023
11.19	0.70937	0.70887	0.00023
11.14	0.70919	0.70889	0.00025
11.10	0.70847	0.70884	0.00024
11.06	0.70855	0.70888	0.00023
11.01	0.70895	0.70896	0.00023
10.97	0.70937	0.70889	0.00027
10.92	0.70871	0.70883	0.00025
10.88	0.70835	0.70882	0.00026

13.49	0.70880	0.70873	0.00014
13.45	0.70907	0.70872	0.00014
13.40	0.70887	0.70869	0.00012
13.36	0.70883	0.70868	0.00012
13.32	0.70856	0.70870	0.00014
13.28	0.70900	0.70868	0.00016
13.23	0.70855	0.70870	0.00018
13.19	0.70854	0.70875	0.00019
13.15	0.70873	0.70879	0.00019
13.11	0.70835	0.70879	0.00019
13.06	0.70866	0.70881	0.00017
13.02	0.70880	0.70882	0.00017
12.98	0.70875	0.70879	0.00018
12.94	0.70908	0.70881	0.00019
12.89	0.70832	0.70877	0.00018
12.85	0.70918	0.70875	0.00020
12.81	0.70906	0.70871	0.00018
12.76	0.70898	0.70872	0.00019
12.72	0.70876	0.70872	0.00019
12.68	0.70852	0.70871	0.00019
12.64	0.70873	0.70874	0.00018
12.59	0.70846	0.70875	0.00018
12.55	0.70901	0.70877	0.00017
12.51	0.70870	0.70871	0.00017
12.47	0.70814	0.70869	0.00018
12.42	0.70875	0.70874	0.00014
12.38	0.70918	0.70877	0.00015
12.34	0.70890	0.70878	0.00016
12.29	0.70870	0.70874	0.00016
12.25	0.70879	0.70875	0.00016
12.21	0.70882	0.70872	0.00017
12.17	0.70871	0.70868	0.00018
12.12	0.70842	0.70868	0.00018
12.08	0.70852	0.70872	0.00018
12.04	0.70857	0.70876	0.00017
12.00	0.70909	0.70877	0.00017
11.95	0.70926	0.70875	0.00016
11.91	0.70856	0.70871	0.00011
11.87	0.70876	0.70872	0.00011
11.83	0.70852	0.70874	0.00011
11.78	0.70836	0.70878	0.00011
11.74	0.70877	0.70881	0.00006
11.70	0.70886	0.70887	0.00012
11.65	0.70888	0.70890	0.00013
11.61	0.70870	0.70892	0.00014
11.57	0.70890	0.70894	0.00013
11.53	0.70879	0.70892	0.00014
11.48	0.70871	0.70886	0.00020
11.44	0.70891	0.70888	0.00020
11.40	0.70890	0.70884	0.00021

10.84	0.70885	0.70889	0.00024	11.36	0.70869	0.70884	0.00021
10.79	0.70890	0.70885	0.00025	11.31	0.70934	0.70887	0.00021
10.75	0.70954	0.70883	0.00025	11.27	0.70917	0.70883	0.00019
10.70	0.70868	0.70873	0.00020	11.23	0.70912	0.70879	0.00017
10.66	0.70893	0.70875	0.00020	11.18	0.70884	0.70874	0.00016
10.62	0.70932	0.70872	0.00020	11.14	0.70877	0.70877	0.00018
10.57	0.70821	0.70868	0.00016	11.10	0.70819	0.70879	0.00018
10.53	0.70885	0.70871	0.00013	11.06	0.70892	0.70886	0.00012
10.48	0.70854	0.70870	0.00012	11.01	0.70848	0.70885	0.00012
10.44	0.70903	0.70872	0.00012	10.97	0.70887	0.70882	0.00017
10.40	0.70847	0.70870	0.00010	10.93	0.70898	0.70884	0.00018
10.35	0.70875	0.70874	0.00009	10.89	0.70901	0.70884	0.00018
10.31	0.70853	0.70875	0.00010	10.84	0.70875	0.70882	0.00017
10.27	0.70889	0.70881	0.00011	10.80	0.70865	0.70881	0.00018
10.22	0.70859	0.70881	0.00011	10.76	0.70913	0.70883	0.00018
10.18	0.70891	0.70886	0.00011	10.72	0.70896	0.70880	0.00016
10.13	0.70851	0.70882	0.00013	10.67	0.70888	0.70881	0.00017
10.09	0.70875	0.70888	0.00013	10.63	0.70878	0.70879	0.00017
10.05	0.70878	0.70889	0.00012	10.59	0.70818	0.70875	0.00019
10.00	0.70878	0.70893	0.00013	10.54	0.70914	0.70875	0.00018
9.96	0.70891	0.70892	0.00014	10.50	0.70888	0.70876	0.00018
9.91	0.70889	0.70892	0.00014	10.46	0.70890	0.70869	0.00021
9.87	0.70912	0.70892	0.00014	10.42	0.70857	0.70869	0.00021
9.83	0.70885	0.70885	0.00017	10.37	0.70888	0.70869	0.00021
9.78	0.70909	0.70890	0.00019	10.33	0.70884	0.70862	0.00022
9.74	0.70849	0.70891	0.00020	10.29	0.70908	0.70854	0.00024
9.70	0.70915	0.70892	0.00019	10.25	0.70863	0.70855	0.00024
9.65	0.70890	0.70887	0.00019	10.20	0.70838	0.70862	0.00029
9.61	0.70914	0.70890	0.00020	10.16	0.70825	0.70870	0.00031
9.56	0.70868	0.70886	0.00020	10.12	0.70916	0.70877	0.00029
9.52	0.70891	0.70888	0.00019	10.07	0.70826	0.70875	0.00028
9.48	0.70892	0.70889	0.00019	10.03	0.70885	0.70875	0.00028
9.43	0.70839	0.70889	0.00019	9.99	0.70855	0.70872	0.00029
9.39	0.70935	0.70895	0.00016	9.95	0.70820	0.70875	0.00029
9.34	0.70917	0.70894	0.00015	9.90	0.70809	0.70884	0.00026
9.30	0.70863	0.70893	0.00014	9.86	0.70911	0.70893	0.00020
9.26	0.70858	0.70896	0.00012	9.82	0.70933	0.70886	0.00023
9.21	0.70921	0.70905	0.00013	9.78	0.70920	0.70878	0.00021
9.17	0.70879	0.70902	0.00012	9.73	0.70896	0.70881	0.00023
9.12	0.70890	0.70904	0.00011	9.69	0.70900	0.70878	0.00023
9.08	0.70895	0.70904	0.00011	9.65	0.70825	0.70874	0.00023
9.04	0.70896	0.70906	0.00011	9.61	0.70854	0.70875	0.00022
8.99	0.70902	0.70906	0.00011	9.56	0.70886	0.70876	0.00022
8.95	0.70923	0.70904	0.00012	9.52	0.70907	0.70877	0.00022
8.91	0.70906	0.70901	0.00012	9.48	0.70902	0.70870	0.00023
8.86	0.70889	0.70897	0.00014	9.43	0.70835	0.70867	0.00022
8.82	0.70946	0.70903	0.00016	9.39	0.70858	0.70871	0.00020
8.77	0.70893	0.70899	0.00014	9.35	0.70944	0.70870	0.00021
8.73	0.70901	0.70898	0.00014	9.31	0.70869	0.70860	0.00013
8.69	0.70890	0.70898	0.00014	9.26	0.70858	0.70862	0.00015

8.64	0.70913	0.70901	0.00015
8.60	0.70896	0.70900	0.00015
8.55	0.70878	0.70898	0.00016
8.51	0.70900	0.70898	0.00016
8.47	0.70866	0.70896	0.00016
8.42	0.70945	0.70903	0.00016
8.38	0.70912	0.70897	0.00013
8.34	0.70877	0.70898	0.00013
8.29	0.70899	0.70900	0.00012
8.25	0.70925	0.70903	0.00014
8.20	0.70904	0.70903	0.00014
8.16	0.70873	0.70900	0.00015
8.12	0.70878	0.70905	0.00014
8.07	0.70884	0.70908	0.00013
8.03	0.70931	0.70912	0.00012
7.98	0.70891	0.70913	0.00013
7.94	0.70916	0.70915	0.00012
7.90	0.70896	0.70910	0.00015
7.85	0.70929	0.70907	0.00018
7.81	0.70925	0.70906	0.00017
7.76	0.70874	0.70908	0.00018
7.72	0.70924	0.70911	0.00017
7.68	0.70909	0.70912	0.00017
7.63	0.70928	0.70907	0.00020
7.59	0.70940	0.70903	0.00020
7.55	0.70907	0.70896	0.00019
7.50	0.70870	0.70897	0.00020
7.46	0.70864	0.70901	0.00019
7.41	0.70918	0.70900	0.00020
7.37	0.70943	0.70896	0.00020
7.33	0.70908	0.70893	0.00017
7.28	0.70931	0.70892	0.00017
7.24	0.70859	0.70889	0.00014
7.19	0.70891	0.70894	0.00013
7.15	0.70866	0.70896	0.00014
7.11	0.70922	0.70902	0.00013
7.06	0.70906	0.70900	0.00012
7.02	0.70854	0.70899	0.00012
6.98	0.70884	0.70900	0.00011
6.93	0.70910	0.70898	0.00013
6.89	0.70895	0.70898	0.00013
6.84	0.70900	0.70891	0.00019
6.80	0.70913	0.70893	0.00020
6.76	0.70912	0.70892	0.00019
6.71	0.70923	0.70891	0.00019
6.67	0.70906	0.70890	0.00018
6.62	0.70895	0.70886	0.00018
6.58	0.70859	0.70896	0.00029
6.54	0.70862	0.70898	0.00028
6.49	0.70910	0.70904	0.00027

9.22	0.70836	0.70866	0.00016
9.18	0.70861	0.70872	0.00016
9.14	0.70901	0.70872	0.00016
9.09	0.70833	0.70871	0.00015
9.05	0.70880	0.70876	0.00012
9.01	0.70866	0.70876	0.00012
8.96	0.70848	0.70879	0.00013
8.92	0.70847	0.70883	0.00011
8.88	0.70894	0.70885	0.00008
8.84	0.70891	0.70888	0.00011
8.79	0.70900	0.70886	0.00012
8.75	0.70862	0.70882	0.00012
8.71	0.70892	0.70884	0.00011
8.67	0.70881	0.70883	0.00011
8.62	0.70875	0.70885	0.00011
8.58	0.70898	0.70885	0.00011
8.54	0.70889	0.70887	0.00012
8.50	0.70870	0.70885	0.00013
8.45	0.70923	0.70889	0.00013
8.41	0.70867	0.70885	0.00011
8.37	0.70866	0.70882	0.00014
8.32	0.70882	0.70881	0.00015
8.28	0.70881	0.70872	0.00023
8.24	0.70897	0.70878	0.00027
8.20	0.70877	0.70881	0.00029
8.15	0.70915	0.70883	0.00029
8.11	0.70873	0.70878	0.00028
8.07	0.70909	0.70880	0.00028
8.03	0.70881	0.70877	0.00027
7.98	0.70836	0.70880	0.00028
7.94	0.70854	0.70889	0.00028
7.90	0.70794	0.70893	0.00026
7.86	0.70942	0.70906	0.00015
7.81	0.70930	0.70898	0.00015
7.77	0.70893	0.70892	0.00014
7.73	0.70866	0.70893	0.00015
7.68	0.70891	0.70891	0.00017
7.64	0.70885	0.70894	0.00018
7.60	0.70909	0.70889	0.00021
7.56	0.70927	0.70887	0.00021
7.51	0.70896	0.70886	0.00020
7.47	0.70920	0.70884	0.00020
7.43	0.70860	0.70881	0.00018
7.39	0.70868	0.70886	0.00019
7.34	0.70912	0.70884	0.00020
7.30	0.70846	0.70880	0.00020
7.26	0.70918	0.70888	0.00021
7.21	0.70836	0.70884	0.00020
7.17	0.70884	0.70890	0.00017
7.13	0.70919	0.70892	0.00017

6.45	0.70831	0.70905	0.00027
6.41	0.70916	0.70917	0.00023
6.36	0.70910	0.70908	0.00029
6.32	0.70895	0.70912	0.00030
6.27	0.70913	0.70914	0.00030
6.23	0.70867	0.70913	0.00030
6.19	0.70995	0.70922	0.00030
6.14	0.70878	0.70912	0.00026
6.10	0.70926	0.70908	0.00030
6.05	0.70915	0.70899	0.00032
6.01	0.70953	0.70898	0.00032
5.97	0.70829	0.70894	0.00030
5.92	0.70952	0.70904	0.00027
5.88	0.70908	0.70898	0.00024
5.83	0.70903	0.70903	0.00027
5.79	0.70964	0.70899	0.00028
5.75	0.70892	0.70896	0.00026
5.70	0.70832	0.70894	0.00027
5.66	0.70840	0.70908	0.00028
5.62	0.70904	0.70914	0.00024
5.57	0.70914	0.70912	0.00024
5.53	0.70926	0.70909	0.00025
5.48	0.70896	0.70909	0.00025
5.44	0.70955	0.70914	0.00026
5.40	0.70863	0.70907	0.00025
5.35	0.70940	0.70913	0.00023
5.31	0.70865	0.70908	0.00023
5.26	0.70980	0.70907	0.00023
5.22	0.70895	0.70899	0.00017
5.18	0.70885	0.70902	0.00018
5.13	0.70882	0.70902	0.00018
5.09	0.70931	0.70904	0.00018
5.05	0.70944	0.70902	0.00017
5.00	0.70884	0.70899	0.00014
4.96	0.70926	0.70900	0.00014
4.91	0.70886	0.70898	0.00013
4.87	0.70860	0.70900	0.00012
4.83	0.70896	0.70902	0.00010
4.78	0.70929	0.70899	0.00013
4.74	0.70878	0.70896	0.00011
4.69	0.70904	0.70895	0.00013
4.65	0.70916	0.70895	0.00013
4.61	0.70909	0.70896	0.00014
4.56	0.70902	0.70891	0.00016
4.52	0.70906	0.70898	0.00023
4.47	0.70905	0.70903	0.00026
4.43	0.70876	0.70902	0.00026
4.39	0.70862	0.70902	0.00026
4.34	0.70908	0.70902	0.00026
4.30	0.70862	0.70899	0.00026

7.09	0.70879	0.70890	0.00016
7.04	0.70887	0.70894	0.00016
7.00	0.70913	0.70888	0.00020
6.96	0.70843	0.70895	0.00027
6.92	0.70871	0.70903	0.00025
6.87	0.70934	0.70904	0.00024
6.83	0.70875	0.70897	0.00025
6.79	0.70892	0.70893	0.00028
6.75	0.70911	0.70885	0.00032
6.70	0.70899	0.70879	0.00032
6.66	0.70911	0.70885	0.00035
6.62	0.70834	0.70883	0.00035
6.57	0.70977	0.70886	0.00033
6.53	0.70928	0.70880	0.00028
6.49	0.70883	0.70876	0.00026
6.45	0.70856	0.70874	0.00026
6.40	0.70836	0.70878	0.00026
6.36	0.70810	0.70884	0.00024
6.32	0.70857	0.70890	0.00018
6.28	0.70953	0.70891	0.00018
6.23	0.70891	0.70881	0.00012
6.19	0.70866	0.70880	0.00012
6.15	0.70916	0.70888	0.00017
6.10	0.70887	0.70882	0.00016
6.06	0.70870	0.70885	0.00017
6.02	0.70895	0.70891	0.00019
5.98	0.70897	0.70887	0.00021
5.93	0.70871	0.70889	0.00022
5.89	0.70860	0.70890	0.00022
5.85	0.70857	0.70894	0.00021
5.81	0.70878	0.70895	0.00020
5.76	0.70943	0.70897	0.00019
5.72	0.70863	0.70886	0.00020
5.68	0.70913	0.70889	0.00020
5.64	0.70934	0.70891	0.00022
5.59	0.70852	0.70889	0.00020
5.55	0.70918	0.70893	0.00018
5.51	0.70882	0.70887	0.00019
5.46	0.70900	0.70887	0.00019
5.42	0.70871	0.70886	0.00019
5.38	0.70893	0.70890	0.00019
5.34	0.70830	0.70889	0.00019
5.29	0.70897	0.70893	0.00014
5.25	0.70937	0.70894	0.00014
5.21	0.70913	0.70886	0.00011
5.17	0.70892	0.70883	0.00010
5.12	0.70857	0.70878	0.00012
5.08	0.70879	0.70880	0.00011
5.04	0.70892	0.70887	0.00018
4.99	0.70905	0.70886	0.00018

4.26	0.70909	0.70902	0.00025
4.21	0.70927	0.70903	0.00025
4.17	0.70855	0.70899	0.00025
4.12	0.70972	0.70907	0.00023
4.08	0.70959	0.70905	0.00021
4.04	0.70890	0.70897	0.00017
3.99	0.70875	0.70896	0.00017
3.95	0.70859	0.70897	0.00017
3.90	0.70880	0.70907	0.00018
3.86	0.70894	0.70915	0.00020
3.82	0.70915	0.70914	0.00020
3.77	0.70892	0.70911	0.00021
3.73	0.70932	0.70906	0.00024
3.69	0.70948	0.70906	0.00025
3.64	0.70881	0.70907	0.00025
3.60	0.70883	0.70908	0.00024
3.55	0.70887	0.70905	0.00026
3.51	0.70953	0.70912	0.00027
3.47	0.70960	0.70909	0.00026
3.42	0.70892	0.70900	0.00024
3.38	0.70876	0.70897	0.00025
3.33	0.70849	0.70895	0.00026
3.29	0.70934	0.70897	0.00025
3.25	0.70949	0.70891	0.00024
3.20	0.70893	0.70882	0.00021
3.16	0.70858	0.70879	0.00021
3.11	0.70958	0.70885	0.00021
3.07	0.70915	0.70878	0.00014
3.03	0.70871	0.70877	0.00014
2.98	0.70864	0.70881	0.00015
2.94	0.70857	0.70881	0.00015
2.90	0.70874	0.70885	0.00014
2.85	0.70873	0.70888	0.00015
2.81	0.70855	0.70890	0.00014
2.76	0.70863	0.70893	0.00012
2.72	0.70918	0.70897	0.00010
2.68	0.70892	0.70898	0.00011
2.63	0.70908	0.70900	0.00011
2.59	0.70905	0.70899	0.00011
2.54	0.70861	0.70899	0.00011
2.50	0.70902	0.70905	0.00008
2.46	0.70906	0.70900	0.00014
2.41	0.70889	0.70899	0.00014
2.37	0.70887	0.70895	0.00017
2.33	0.70903	0.70903	0.00022
2.28	0.70927	0.70902	0.00022
2.24	0.70907	0.70901	0.00022
2.19	0.70907	0.70903	0.00023
2.15	0.70902	0.70901	0.00023
2.11	0.70924	0.70902	0.00023
4.95	0.70888	0.70882	0.00018
4.91	0.70874	0.70885	0.00019
4.87	0.70898	0.70893	0.00023
4.82	0.70865	0.70891	0.00023
4.78	0.70876	0.70886	0.00027
4.74	0.70848	0.70890	0.00028
4.70	0.70874	0.70900	0.00028
4.65	0.70953	0.70906	0.00028
4.61	0.70884	0.70904	0.00027
4.57	0.70864	0.70898	0.00031
4.53	0.70917	0.70899	0.00031
4.48	0.70950	0.70902	0.00032
4.44	0.70875	0.70894	0.00030
4.40	0.70818	0.70890	0.00032
4.35	0.70919	0.70893	0.00029
4.31	0.70944	0.70896	0.00031
4.27	0.70932	0.70887	0.00030
4.23	0.70934	0.70884	0.00029
4.18	0.70822	0.70880	0.00027
4.14	0.70881	0.70887	0.00023
4.10	0.70940	0.70883	0.00025
4.06	0.70872	0.70871	0.00024
4.01	0.70835	0.70874	0.00025
3.97	0.70855	0.70878	0.00023
3.93	0.70949	0.70879	0.00023
3.88	0.70853	0.70872	0.00017
3.84	0.70903	0.70874	0.00016
3.80	0.70890	0.70874	0.00017
3.76	0.70887	0.70868	0.00019
3.71	0.70847	0.70870	0.00020
3.67	0.70821	0.70872	0.00020
3.63	0.70901	0.70876	0.00016
3.59	0.70874	0.70875	0.00016
3.54	0.70862	0.70878	0.00017
3.50	0.70885	0.70880	0.00016
3.46	0.70866	0.70875	0.00019
3.42	0.70910	0.70879	0.00020
3.37	0.70827	0.70880	0.00020
3.33	0.70910	0.70885	0.00017
3.29	0.70861	0.70879	0.00017
3.24	0.70864	0.70885	0.00018
3.20	0.70895	0.70886	0.00017
3.16	0.70897	0.70881	0.00019
3.12	0.70884	0.70883	0.00020
3.07	0.70834	0.70883	0.00020
3.03	0.70912	0.70889	0.00017
2.99	0.70914	0.70890	0.00018
2.95	0.70880	0.70886	0.00017
2.90	0.70854	0.70887	0.00017
2.86	0.70920	0.70895	0.00018

2.06	0.70849	0.70900	0.00022
2.02	0.70898	0.70901	0.00022
1.97	0.70849	0.70898	0.00023
1.93	0.70968	0.70901	0.00021
1.89	0.70892	0.70893	0.00014
1.84	0.70918	0.70895	0.00015
1.80	0.70925	0.70895	0.00015
1.75	0.70888	0.70891	0.00014
1.71	0.70913	0.70886	0.00017
1.67	0.70903	0.70887	0.00017
1.62	0.70852	0.70883	0.00017
1.58	0.70869	0.70886	0.00015
1.54	0.70882	0.70882	0.00019
1.49	0.70890	0.70882	0.00019
1.45	0.70911	0.70879	0.00020
1.40	0.70921	0.70882	0.00022
1.36	0.70882	0.70883	0.00023
1.32	0.70842	0.70876	0.00027
1.27	0.70913	0.70871	0.00031
1.23	0.70865	0.70860	0.00033
1.18	0.70885	0.70862	0.00033
1.14	0.70826	0.70869	0.00038
1.10	0.70882	0.70863	0.00043
1.05	0.70863	0.70859	0.00043
1.01	0.70938	0.70865	0.00045
0.97	0.70935	0.70869	0.00049
0.92	0.70810	0.70859	0.00047
0.88	0.70797	0.70871	0.00047
0.83	0.70800	0.70871	0.00047
0.79	0.70885	0.70882	0.00045
0.75	0.70958	0.70879	0.00045
0.70	0.70766	0.70870	0.00042
0.66	0.70835	0.70885	0.00036
0.61	0.70922	0.70887	0.00035
0.57	0.70986	0.70884	0.00034
0.53	0.70834	0.70874	0.00026
0.48	0.70924	0.70881	0.00025
0.44	0.70800	0.70878	0.00023
0.39	0.70914	0.70886	0.00017
0.35	0.70852	0.70883	0.00016
0.31	0.70864	0.70887	0.00015
0.26	0.70922	0.70891	0.00015
0.22	0.70853	0.70885	0.00013
0.18	0.70892	0.70893	0.00008
0.13	0.70882	0.70894	0.00010
0.09	0.70911	0.70900	0.00010
0.04	0.70888		

2.82	0.70872	0.70891	0.00017
2.77	0.70843	0.70892	0.00016
2.73	0.70916	0.70891	0.00017
2.69	0.70883	0.70891	0.00017
2.65	0.70895	0.70893	0.00017
2.60	0.70917	0.70884	0.00024
2.56	0.70883	0.70885	0.00024
2.52	0.70885	0.70884	0.00024
2.48	0.70936	0.70884	0.00024
2.43	0.70881	0.70879	0.00021
2.39	0.70879	0.70880	0.00022
2.35	0.70837	0.70887	0.00025
2.31	0.70917	0.70888	0.00024
2.26	0.70896	0.70881	0.00024
2.22	0.70811	0.70872	0.00028
2.18	0.70923	0.70874	0.00026
2.13	0.70873	0.70871	0.00024
2.09	0.70892	0.70870	0.00024
2.05	0.70884	0.70867	0.00025
2.01	0.70893	0.70866	0.00025
1.96	0.70942	0.70862	0.00024
1.92	0.70849	0.70850	0.00016
1.88	0.70843	0.70851	0.00016
1.84	0.70813	0.70851	0.00016
1.79	0.70828	0.70860	0.00016
1.75	0.70895	0.70870	0.00018
1.71	0.70856	0.70861	0.00021
1.62	0.70873	0.70871	0.00023
1.58	0.70859	0.70868	0.00023
1.54	0.70830	0.70869	0.00023
1.49	0.70861	0.70878	0.00024
1.45	0.70845	0.70879	0.00023
1.41	0.70896	0.70882	0.00022
1.37	0.70918	0.70879	0.00022
1.32	0.70810	0.70876	0.00021
1.28	0.70905	0.70882	0.00015
1.24	0.70911	0.70883	0.00016
1.20	0.70846	0.70884	0.00017
1.15	0.70866	0.70882	0.00020
1.11	0.70920	0.70890	0.00023
1.07	0.70871	0.70879	0.00027
1.02	0.70883	0.70879	0.00027
0.98	0.70859	0.70870	0.00032
0.94	0.70891	0.70876	0.00033
0.90	0.70870	0.70876	0.00034
0.85	0.70911	0.70874	0.00034
0.81	0.70927	0.70864	0.00035
0.77	0.70826	0.70860	0.00033
0.73	0.70944	0.70866	0.00032
0.68	0.70806	0.70864	0.00030

0.64	0.70875	0.70869	0.00027
0.60	0.70790	0.70875	0.00030
0.55	0.70917	0.70883	0.00024
0.51	0.70894	0.70881	0.00023
0.47	0.70853	0.70880	0.00023
0.43	0.70808	0.70880	0.00023
0.38	0.70888	0.70888	0.00017
0.34	0.70882	0.70888	0.00018
0.30	0.70924	0.70889	0.00019
0.26	0.70859	0.70884	0.00018
0.21	0.70934	0.70889	0.00019
0.17	0.70868	0.70877	0.00010
0.13	0.70896	0.70880	0.00012
0.09	0.70885	0.70872	0.00011
0.04	0.70859		

ARB 119.2.1 (M2)

Distance from cervix (mm)	$^{87}Sr/^{86}Sr$	10 point mov. average	2 SE on mov. average
41.67	0.70801	0.70866	0.00023
41.62	0.70914	0.70876	0.00019
41.56	0.70813	0.70869	0.00017
41.51	0.70900	0.70876	0.00012
41.46	0.70867	0.70875	0.00011
41.40	0.70858	0.70876	0.00011
41.35	0.70854	0.70878	0.00011
41.29	0.70891	0.70876	0.00012
41.24	0.70875	0.70877	0.00013
41.19	0.70890	0.70880	0.00014
41.13	0.70899	0.70881	0.00015
41.08	0.70847	0.70878	0.00015
41.03	0.70885	0.70879	0.00013
40.97	0.70881	0.70879	0.00013
40.92	0.70885	0.70876	0.00014
40.86	0.70868	0.70876	0.00014
40.81	0.70838	0.70877	0.00014
40.76	0.70904	0.70881	0.00011
40.70	0.70904	0.70878	0.00010
40.65	0.70899	0.70875	0.00008
40.60	0.70864	0.70876	0.00010
40.54	0.70863	0.70876	0.00010
40.49	0.70883	0.70879	0.00010
40.43	0.70852	0.70879	0.00010
40.38	0.70880	0.70884	0.00009
40.33	0.70885	0.70882	0.00010
40.27	0.70873	0.70881	0.00011
40.22	0.70879	0.70882	0.00011
40.17	0.70869	0.70883	0.00011
40.11	0.70912	0.70881	0.00012
40.06	0.70868	0.70875	0.00011
40.01	0.70887	0.70876	0.00011
39.95	0.70886	0.70873	0.00011
39.90	0.70902	0.70875	0.00013
39.84	0.70858	0.70871	0.00012
39.79	0.70869	0.70873	0.00011
39.74	0.70891	0.70871	0.00013
39.68	0.70884	0.70868	0.00012
39.63	0.70854	0.70872	0.00015
39.58	0.70851	0.70871	0.00016
39.52	0.70875	0.70877	0.00017
39.47	0.70863	0.70878	0.00017
39.41	0.70906	0.70882	0.00018
39.36	0.70855	0.70879	0.00017
39.31	0.70882	0.70882	0.00016
39.25	0.70844	0.70882	0.00016
39.20	0.70869	0.70888	0.00014

ARB 119.3.1 (M3)

Distance from cervix (mm)	$^{87}Sr/^{86}Sr$	10 point mov. average	2 SE on mov. average
46.18	0.70929	0.70912	0.00026
46.14	0.70882	0.70914	0.00027
46.09	0.70854	0.70918	0.00026
46.05	0.70940	0.70929	0.00023
46.00	0.70926	0.70934	0.00026
45.96	0.70952	0.70943	0.00031
45.91	0.70843	0.70933	0.00036
45.87	0.70895	0.70936	0.00033
45.82	0.70951	0.70940	0.00032
45.78	0.70952	0.70938	0.00032
45.73	0.70948	0.70938	0.00032
45.69	0.70918	0.70938	0.00032
45.65	0.70963	0.70939	0.00032
45.60	0.70989	0.70938	0.00032
45.56	0.71021	0.70938	0.00032
45.51	0.70849	0.70932	0.00027
45.47	0.70874	0.70938	0.00020
45.42	0.70937	0.70940	0.00018
45.38	0.70928	0.70933	0.00023
45.33	0.70954	0.70937	0.00024
45.29	0.70946	0.70936	0.00023
45.24	0.70925	0.70937	0.00024
45.20	0.70958	0.70939	0.00024
45.16	0.70992	0.70935	0.00023
45.11	0.70956	0.70928	0.00020
45.07	0.70910	0.70922	0.00020
45.02	0.70892	0.70932	0.00026
44.98	0.70870	0.70941	0.00027
44.93	0.70967	0.70945	0.00023
44.89	0.70943	0.70947	0.00024
44.84	0.70962	0.70953	0.00026
44.80	0.70939	0.70951	0.00026
44.75	0.70923	0.70948	0.00028
44.71	0.70916	0.70952	0.00027
44.67	0.70895	0.70949	0.00030
44.62	0.71011	0.70959	0.00028
44.58	0.70983	0.70957	0.00027
44.53	0.70909	0.70953	0.00027
44.49	0.70988	0.70961	0.00025
44.44	0.71000	0.70959	0.00025
44.40	0.70946	0.70963	0.00028
44.35	0.70905	0.70973	0.00032
44.31	0.70967	0.70979	0.00028
44.26	0.70887	0.70982	0.00028
44.22	0.70995	0.70985	0.00025
44.18	0.70995	0.70976	0.00028
44.13	0.70942	0.70971	0.00028

39.15	0.70918	0.70890	0.00013	44.09	0.70982	0.70974	0.00028
39.09	0.70845	0.70890	0.00013	44.04	0.70972	0.70971	0.00028
39.04	0.70914	0.70892	0.00009	44.00	0.71037	0.70967	0.00029
38.99	0.70882	0.70888	0.00009	43.95	0.71044	0.70961	0.00024
38.93	0.70903	0.70886	0.00010	43.91	0.70972	0.70947	0.00018
38.88	0.70878	0.70880	0.00013	43.86	0.70992	0.70935	0.00025
38.82	0.70884	0.70879	0.00013	43.82	0.70915	0.70928	0.00022
38.77	0.70885	0.70875	0.00014	43.77	0.70914	0.70922	0.00026
38.72	0.70898	0.70874	0.00014	43.73	0.70945	0.70923	0.00026
38.66	0.70896	0.70875	0.00015	43.69	0.70966	0.70918	0.00026
38.61	0.70911	0.70875	0.00015	43.64	0.70949	0.70911	0.00024
38.56	0.70873	0.70874	0.00014	43.60	0.70939	0.70907	0.00022
38.50	0.70868	0.70872	0.00014	43.55	0.70973	0.70904	0.00021
38.45	0.70860	0.70874	0.00015	43.51	0.70909	0.70904	0.00021
38.39	0.70843	0.70873	0.00015	43.46	0.70851	0.70905	0.00021
38.34	0.70872	0.70876	0.00014	43.42	0.70917	0.70911	0.00017
38.29	0.70843	0.70875	0.00014	43.37	0.70861	0.70911	0.00017
38.23	0.70877	0.70881	0.00013	43.33	0.70922	0.70921	0.00016
38.18	0.70904	0.70882	0.00013	43.28	0.70893	0.70916	0.00019
38.13	0.70893	0.70879	0.00012	43.24	0.70899	0.70924	0.00021
38.07	0.70904	0.70884	0.00016	43.20	0.70904	0.70928	0.00021
38.02	0.70854	0.70879	0.00016	43.15	0.70910	0.70931	0.00020
37.96	0.70887	0.70885	0.00016	43.11	0.70971	0.70929	0.00021
37.91	0.70848	0.70887	0.00017	43.06	0.70917	0.70924	0.00019
37.86	0.70874	0.70894	0.00015	43.02	0.70917	0.70921	0.00021
37.80	0.70868	0.70895	0.00014	42.97	0.70914	0.70922	0.00021
37.75	0.70898	0.70896	0.00014	42.93	0.70962	0.70934	0.00029
37.70	0.70891	0.70894	0.00014	42.88	0.70871	0.70932	0.00029
37.64	0.70877	0.70886	0.00023	42.84	0.70971	0.70935	0.00026
37.59	0.70933	0.70882	0.00025	42.80	0.70946	0.70931	0.00025
37.54	0.70860	0.70878	0.00022	42.75	0.70930	0.70928	0.00025
37.48	0.70915	0.70877	0.00022	42.71	0.70890	0.70924	0.00026
37.43	0.70904	0.70880	0.00025	42.66	0.70920	0.70924	0.00026
37.37	0.70916	0.70879	0.00025	42.62	0.70885	0.70925	0.00026
37.32	0.70890	0.70878	0.00025	42.57	0.70935	0.70936	0.00028
37.27	0.70878	0.70874	0.00025	42.53	0.71025	0.70942	0.00031
37.21	0.70879	0.70872	0.00026	42.48	0.70947	0.70926	0.00028
37.16	0.70805	0.70867	0.00027	42.44	0.70900	0.70928	0.00029
37.11	0.70839	0.70876	0.00024	42.39	0.70930	0.70934	0.00029
37.05	0.70891	0.70880	0.00022	42.35	0.70916	0.70936	0.00029
37.00	0.70853	0.70878	0.00022	42.31	0.70896	0.70934	0.00030
36.94	0.70946	0.70875	0.00024	42.26	0.70890	0.70936	0.00029
36.89	0.70897	0.70875	0.00023	42.22	0.70921	0.70947	0.00029
36.84	0.70907	0.70876	0.00024	42.17	0.70998	0.70944	0.00031
36.78	0.70842	0.70872	0.00023	42.13	0.70999	0.70941	0.00030
36.73	0.70860	0.70878	0.00022	42.08	0.70864	0.70933	0.00027
36.68	0.70827	0.70879	0.00022	42.04	0.70967	0.70941	0.00022
36.62	0.70896	0.70886	0.00019	41.99	0.70956	0.70941	0.00022
36.57	0.70880	0.70885	0.00019	41.95	0.70951	0.70938	0.00022
36.51	0.70865	0.70883	0.00020	41.90	0.70895	0.70931	0.00024

36.46	0.70830	0.70881	0.00020	41.86	0.70918	0.70936	0.00022
36.41	0.70941	0.70887	0.00017	41.82	0.70997	0.70938	0.00022
36.35	0.70908	0.70879	0.00013	41.77	0.70891	0.70927	0.00020
36.30	0.70870	0.70872	0.00013	41.73	0.70973	0.70931	0.00019
36.25	0.70906	0.70871	0.00013	41.68	0.70920	0.70920	0.00021
36.19	0.70870	0.70869	0.00011	41.64	0.70947	0.70923	0.00022
36.14	0.70892	0.70869	0.00011	41.59	0.70963	0.70921	0.00021
36.09	0.70884	0.70869	0.00011	41.55	0.70925	0.70912	0.00021
36.03	0.70860	0.70871	0.00012	41.50	0.70885	0.70915	0.00023
35.98	0.70854	0.70873	0.00012	41.46	0.70939	0.70918	0.00022
35.92	0.70890	0.70877	0.00012	41.41	0.70943	0.70909	0.00026
35.87	0.70854	0.70871	0.00014	41.37	0.70883	0.70903	0.00025
35.82	0.70841	0.70877	0.00016	41.33	0.70935	0.70900	0.00027
35.76	0.70861	0.70877	0.00016	41.28	0.70861	0.70896	0.00026
35.71	0.70885	0.70883	0.00018	41.24	0.70949	0.70898	0.00025
35.66	0.70873	0.70884	0.00018	41.19	0.70924	0.70899	0.00026
35.60	0.70892	0.70887	0.00018	41.15	0.70874	0.70902	0.00027
35.55	0.70899	0.70886	0.00018	41.10	0.70960	0.70911	0.00029
35.49	0.70883	0.70884	0.00018	41.06	0.70916	0.70911	0.00029
35.44	0.70888	0.70886	0.00018	41.01	0.70844	0.70911	0.00029
35.39	0.70833	0.70882	0.00020	40.97	0.70889	0.70919	0.00025
35.33	0.70911	0.70885	0.00017	40.92	0.70851	0.70917	0.00027
35.28	0.70841	0.70883	0.00016	40.88	0.70893	0.70926	0.00022
35.23	0.70922	0.70887	0.00014	40.84	0.70881	0.70934	0.00023
35.17	0.70898	0.70880	0.00013	40.79	0.70961	0.70939	0.00019
35.12	0.70900	0.70879	0.00012	40.75	0.70949	0.70939	0.00019
35.07	0.70882	0.70871	0.00016	40.70	0.70963	0.70937	0.00019
35.01	0.70879	0.70871	0.00016	40.66	0.70962	0.70925	0.00025
34.96	0.70906	0.70876	0.00020	40.61	0.70918	0.70927	0.00027
34.90	0.70849	0.70872	0.00018	40.57	0.70926	0.70925	0.00027
34.85	0.70863	0.70874	0.00018	40.52	0.70869	0.70926	0.00027
34.80	0.70894	0.70874	0.00018	40.48	0.70942	0.70931	0.00025
34.74	0.70878	0.70871	0.00017	40.43	0.70970	0.70929	0.00024
34.69	0.70850	0.70873	0.00018	40.39	0.70929	0.70926	0.00023
34.64	0.70886	0.70875	0.00018	40.35	0.70961	0.70924	0.00023
34.58	0.70820	0.70874	0.00018	40.30	0.70925	0.70919	0.00022
34.53	0.70884	0.70883	0.00014	40.26	0.70847	0.70917	0.00022
34.47	0.70927	0.70879	0.00015	40.21	0.70981	0.70927	0.00016
34.42	0.70872	0.70874	0.00011	40.17	0.70898	0.70919	0.00010
34.37	0.70865	0.70879	0.00014	40.12	0.70942	0.70920	0.00010
34.31	0.70866	0.70879	0.00014	40.08	0.70914	0.70925	0.00019
34.26	0.70861	0.70880	0.00014	40.03	0.70926	0.70925	0.00019
34.21	0.70901	0.70882	0.00013	39.99	0.70932	0.70924	0.00019
34.15	0.70872	0.70880	0.00012	39.95	0.70916	0.70925	0.00020
34.10	0.70875	0.70880	0.00012	39.90	0.70906	0.70925	0.00020
34.04	0.70904	0.70883	0.00013	39.86	0.70906	0.70928	0.00020
33.99	0.70847	0.70884	0.00014	39.81	0.70946	0.70936	0.00022
33.94	0.70881	0.70888	0.00012	39.77	0.70907	0.70932	0.00022
33.88	0.70918	0.70889	0.00012	39.72	0.70901	0.70933	0.00022
33.83	0.70867	0.70885	0.00010	39.68	0.71000	0.70933	0.00022

33.78	0.70876	0.70886	0.00009	39.63	0.70904	0.70926	0.00016
33.72	0.70880	0.70886	0.00009	39.59	0.70918	0.70922	0.00020
33.67	0.70875	0.70883	0.00012	39.54	0.70950	0.70932	0.00027
33.62	0.70877	0.70885	0.00012	39.50	0.70911	0.70926	0.00028
33.56	0.70903	0.70885	0.00012	39.46	0.70937	0.70927	0.00028
33.51	0.70920	0.70883	0.00011	39.41	0.70981	0.70928	0.00028
33.45	0.70885	0.70874	0.00011	39.37	0.70907	0.70922	0.00026
33.40	0.70884	0.70876	0.00012	39.32	0.70918	0.70922	0.00025
33.35	0.70878	0.70876	0.00013	39.28	0.70900	0.70929	0.00028
33.29	0.70886	0.70879	0.00013	39.23	0.70933	0.70928	0.00029
33.24	0.70873	0.70876	0.00014	39.19	0.70864	0.70930	0.00029
33.19	0.70850	0.70879	0.00015	39.14	0.71014	0.70938	0.00025
33.13	0.70894	0.70885	0.00014	39.10	0.70890	0.70929	0.00019
33.08	0.70882	0.70884	0.00014	39.05	0.70921	0.70931	0.00018
33.02	0.70874	0.70882	0.00014	39.01	0.70953	0.70935	0.00018
32.97	0.70836	0.70880	0.00015	38.97	0.70919	0.70929	0.00020
32.92	0.70900	0.70885	0.00012	38.92	0.70912	0.70921	0.00027
32.86	0.70891	0.70884	0.00012	38.88	0.70987	0.70919	0.00028
32.81	0.70899	0.70887	0.00013	38.83	0.70889	0.70920	0.00029
32.76	0.70860	0.70886	0.00013	38.79	0.70948	0.70922	0.00028
32.70	0.70903	0.70888	0.00012	38.74	0.70951	0.70918	0.00027
32.65	0.70907	0.70885	0.00012	38.70	0.70921	0.70912	0.00027
32.59	0.70888	0.70881	0.00011	38.65	0.70912	0.70913	0.00027
32.54	0.70866	0.70879	0.00011	38.61	0.70959	0.70914	0.00027
32.49	0.70855	0.70878	0.00012	38.56	0.70894	0.70910	0.00025
32.43	0.70883	0.70882	0.00010	38.52	0.70839	0.70912	0.00025
32.38	0.70891	0.70878	0.00013	38.48	0.70888	0.70920	0.00019
32.33	0.70919	0.70878	0.00013	38.43	0.70996	0.70922	0.00018
32.27	0.70887	0.70874	0.00009	38.39	0.70913	0.70916	0.00007
32.22	0.70879	0.70873	0.00009	38.34	0.70908	0.70918	0.00009
32.17	0.70875	0.70878	0.00013	38.30	0.70893	0.70912	0.00018
32.11	0.70866	0.70876	0.00014	38.25	0.70933	0.70916	0.00018
32.06	0.70870	0.70880	0.00014	38.21	0.70924	0.70922	0.00023
32.00	0.70859	0.70884	0.00015	38.16	0.70917	0.70918	0.00024
31.95	0.70886	0.70885	0.00015	38.12	0.70908	0.70922	0.00025
31.90	0.70846	0.70889	0.00017	38.07	0.70917	0.70916	0.00029
31.84	0.70894	0.70890	0.00015	38.03	0.70915	0.70911	0.00030
31.79	0.70882	0.70889	0.00016	37.99	0.70929	0.70914	0.00031
31.74	0.70875	0.70889	0.00016	37.94	0.70941	0.70916	0.00032
31.68	0.70923	0.70890	0.00015	37.90	0.70842	0.70913	0.00031
31.63	0.70863	0.70885	0.00014	37.85	0.70937	0.70920	0.00027
31.57	0.70903	0.70887	0.00013	37.81	0.70989	0.70921	0.00027
31.52	0.70908	0.70883	0.00013	37.76	0.70885	0.70914	0.00023
31.47	0.70868	0.70880	0.00012	37.72	0.70952	0.70917	0.00022
31.41	0.70927	0.70884	0.00013	37.67	0.70850	0.70905	0.00027
31.36	0.70861	0.70877	0.00009	37.63	0.70865	0.70916	0.00025
31.31	0.70882	0.70879	0.00009	37.58	0.70947	0.70914	0.00027
31.25	0.70876	0.70878	0.00009	37.54	0.70949	0.70909	0.00026
31.20	0.70889	0.70879	0.00009	37.50	0.70910	0.70910	0.00028
31.15	0.70869	0.70879	0.00009	37.45	0.70918	0.70911	0.00028

31.09	0.70885	0.70877	0.00009
31.04	0.70862	0.70875	0.00010
30.98	0.70879	0.70877	0.00010
30.93	0.70904	0.70878	0.00010
30.88	0.70859	0.70872	0.00009
30.82	0.70883	0.70869	0.00013
30.77	0.70875	0.70869	0.00013
30.72	0.70889	0.70871	0.00014
30.66	0.70881	0.70873	0.00016
30.61	0.70858	0.70874	0.00016
30.55	0.70858	0.70875	0.00016
30.50	0.70888	0.70877	0.00015
30.45	0.70881	0.70876	0.00015
30.39	0.70854	0.70870	0.00018
30.34	0.70824	0.70869	0.00019
30.29	0.70886	0.70878	0.00019
30.23	0.70889	0.70880	0.00019
30.18	0.70914	0.70880	0.00019
30.12	0.70889	0.70876	0.00018
30.07	0.70864	0.70871	0.00019
30.02	0.70878	0.70869	0.00020
29.96	0.70879	0.70866	0.00020
29.91	0.70824	0.70868	0.00021
29.86	0.70841	0.70876	0.00020
29.80	0.70919	0.70882	0.00019
29.75	0.70907	0.70881	0.00018
29.70	0.70884	0.70879	0.00017
29.64	0.70876	0.70875	0.00019
29.59	0.70843	0.70871	0.00020
29.53	0.70834	0.70878	0.00020
29.48	0.70854	0.70873	0.00025
29.43	0.70895	0.70873	0.00026
29.37	0.70910	0.70869	0.00025
29.32	0.70898	0.70863	0.00024
29.27	0.70907	0.70868	0.00028
29.21	0.70894	0.70868	0.00028
29.16	0.70839	0.70868	0.00028
29.10	0.70837	0.70872	0.00027
29.05	0.70907	0.70874	0.00026
29.00	0.70794	0.70866	0.00027
28.94	0.70847	0.70876	0.00023
28.89	0.70853	0.70879	0.00022
28.84	0.70856	0.70881	0.00021
28.78	0.70943	0.70881	0.00021
28.73	0.70909	0.70880	0.00020
28.67	0.70892	0.70882	0.00021
28.62	0.70879	0.70885	0.00023
28.57	0.70863	0.70891	0.00025
28.51	0.70820	0.70896	0.00025
28.46	0.70899	0.70901	0.00019
37.41	0.70941	0.70920	0.00034
37.36	0.70920	0.70915	0.00034
37.32	0.70920	0.70919	0.00036
37.27	0.70829	0.70924	0.00037
37.23	0.70957	0.70937	0.00031
37.18	0.70847	0.70934	0.00031
37.14	0.70895	0.70948	0.00025
37.10	0.70965	0.70953	0.00022
37.05	0.70918	0.70950	0.00022
37.01	0.71011	0.70954	0.00021
36.96	0.70889	0.70947	0.00017
36.92	0.70962	0.70953	0.00010
36.87	0.70963	0.70964	0.00025
36.83	0.70966	0.70962	0.00025
36.78	0.70926	0.70961	0.00025
36.74	0.70983	0.70967	0.00025
36.69	0.70945	0.70967	0.00025
36.65	0.70935	0.70967	0.00025
36.61	0.70957	0.70969	0.00024
36.56	0.70943	0.70964	0.00027
36.52	0.70954	0.70962	0.00028
36.47	0.71067	0.70967	0.00028
36.43	0.70948	0.70951	0.00019
36.38	0.70951	0.70949	0.00020
36.34	0.70989	0.70959	0.00029
36.29	0.70977	0.70954	0.00029
36.25	0.70943	0.70950	0.00028
36.20	0.70961	0.70953	0.00029
36.16	0.70903	0.70959	0.00032
36.12	0.70930	0.70959	0.00032
36.07	0.70997	0.70959	0.00032
36.03	0.70915	0.70953	0.00031
35.98	0.70923	0.70959	0.00030
35.94	0.71055	0.70964	0.00029
35.89	0.70939	0.70952	0.00021
35.85	0.70938	0.70949	0.00023
35.80	0.70969	0.70947	0.00023
35.76	0.71023	0.70948	0.00024
35.71	0.70903	0.70942	0.00018
35.67	0.70923	0.70946	0.00016
35.63	0.70940	0.70950	0.00015
35.58	0.70975	0.70952	0.00015
35.54	0.70971	0.70946	0.00016
35.49	0.70939	0.70945	0.00016
35.45	0.70911	0.70947	0.00016
35.40	0.70917	0.70944	0.00020
35.36	0.70983	0.70944	0.00020
35.31	0.70959	0.70942	0.00019
35.27	0.70940	0.70936	0.00021
35.22	0.70961	0.70932	0.00022

28.41	0.70873	0.70899	0.00020
28.35	0.70880	0.70902	0.00019
28.30	0.70854	0.70901	0.00019
28.25	0.70935	0.70903	0.00018
28.19	0.70928	0.70896	0.00017
28.14	0.70920	0.70897	0.00017
28.08	0.70942	0.70896	0.00017
28.03	0.70906	0.70888	0.00014
27.98	0.70873	0.70883	0.00014
27.92	0.70882	0.70888	0.00015
27.87	0.70897	0.70886	0.00016
27.82	0.70877	0.70889	0.00018
27.76	0.70866	0.70887	0.00019
27.71	0.70873	0.70893	0.00020
27.65	0.70930	0.70900	0.00022
27.60	0.70910	0.70892	0.00022
27.55	0.70869	0.70890	0.00022
27.49	0.70858	0.70895	0.00022
27.44	0.70918	0.70903	0.00022
27.39	0.70865	0.70902	0.00022
27.33	0.70928	0.70908	0.00021
27.28	0.70855	0.70903	0.00021
27.23	0.70925	0.70911	0.00019
27.17	0.70943	0.70914	0.00020
27.12	0.70853	0.70909	0.00019
27.06	0.70884	0.70916	0.00015
27.01	0.70924	0.70922	0.00015
26.96	0.70940	0.70923	0.00015
26.90	0.70904	0.70916	0.00019
26.85	0.70922	0.70912	0.00021
26.80	0.70881	0.70913	0.00021
26.74	0.70938	0.70914	0.00020
26.69	0.70950	0.70914	0.00021
26.63	0.70897	0.70914	0.00020
26.58	0.70918	0.70911	0.00022
26.53	0.70951	0.70915	0.00023
26.47	0.70934	0.70912	0.00022
26.42	0.70861	0.70912	0.00022
26.37	0.70871	0.70916	0.00019
26.31	0.70928	0.70918	0.00017
26.26	0.70891	0.70918	0.00017
26.20	0.70941	0.70916	0.00019
26.15	0.70945	0.70916	0.00019
26.10	0.70873	0.70913	0.00018
26.04	0.70953	0.70920	0.00017
25.99	0.70922	0.70914	0.00017
25.94	0.70937	0.70913	0.00017
25.88	0.70903	0.70907	0.00017
25.83	0.70887	0.70905	0.00017
25.78	0.70928	0.70913	0.00020

35.18	0.70966	0.70926	0.00022
35.14	0.70912	0.70926	0.00023
35.09	0.70966	0.70934	0.00025
35.05	0.70957	0.70934	0.00025
35.00	0.70880	0.70935	0.00026
34.96	0.70913	0.70941	0.00023
34.91	0.70968	0.70942	0.00023
34.87	0.70894	0.70938	0.00022
34.82	0.70902	0.70943	0.00020
34.78	0.70899	0.70946	0.00018
34.73	0.70973	0.70946	0.00018
34.69	0.70987	0.70939	0.00019
34.65	0.70966	0.70932	0.00015
34.60	0.70971	0.70928	0.00014
34.56	0.70938	0.70920	0.00010
34.51	0.70920	0.70921	0.00011
34.47	0.70927	0.70923	0.00011
34.42	0.70948	0.70923	0.00011
34.38	0.70930	0.70922	0.00011
34.33	0.70895	0.70931	0.00022
34.29	0.70905	0.70939	0.00022
34.25	0.70921	0.70944	0.00021
34.20	0.70919	0.70942	0.00022
34.16	0.70901	0.70949	0.00023
34.11	0.70943	0.70960	0.00023
34.07	0.70935	0.70964	0.00023
34.02	0.70928	0.70957	0.00030
33.98	0.70947	0.70954	0.00032
33.93	0.71015	0.70952	0.00032
33.89	0.70979	0.70943	0.00029
33.84	0.70952	0.70939	0.00028
33.80	0.70902	0.70939	0.00028
33.76	0.70992	0.70943	0.00027
33.71	0.71010	0.70936	0.00025
33.67	0.70977	0.70927	0.00019
33.62	0.70865	0.70923	0.00015
33.58	0.70905	0.70930	0.00009
33.53	0.70924	0.70934	0.00007
33.49	0.70923	0.70933	0.00008
33.44	0.70946	0.70937	0.00009
33.40	0.70952	0.70929	0.00017
33.35	0.70935	0.70931	0.00019
33.31	0.70928	0.70932	0.00019
33.27	0.70917	0.70930	0.00019
33.22	0.70934	0.70932	0.00019
33.18	0.70934	0.70933	0.00019
33.13	0.70948	0.70936	0.00020
33.09	0.70917	0.70936	0.00020
33.04	0.70961	0.70942	0.00021
33.00	0.70864	0.70939	0.00021

25.72	0.70870	0.70911	0.00020	32.95	0.70973	0.70943	0.00016
25.67	0.70941	0.70915	0.00018	32.91	0.70942	0.70938	0.00014
25.61	0.70913	0.70909	0.00018	32.86	0.70913	0.70938	0.00014
25.56	0.70950	0.70906	0.00019	32.82	0.70932	0.70942	0.00013
25.51	0.70886	0.70899	0.00016	32.78	0.70942	0.70940	0.00014
25.45	0.70911	0.70905	0.00018	32.73	0.70963	0.70938	0.00015
25.40	0.70878	0.70907	0.00019	32.69	0.70952	0.70940	0.00016
25.35	0.70887	0.70909	0.00018	32.64	0.70974	0.70935	0.00017
25.29	0.70962	0.70907	0.00020	32.60	0.70939	0.70937	0.00019
25.24	0.70912	0.70903	0.00017	32.55	0.70896	0.70942	0.00022
25.18	0.70909	0.70903	0.00017	32.51	0.70929	0.70946	0.00020
25.13	0.70880	0.70901	0.00017	32.46	0.70941	0.70954	0.00024
25.08	0.70883	0.70906	0.00017	32.42	0.70947	0.70956	0.00024
25.02	0.70884	0.70904	0.00019	32.37	0.70920	0.70959	0.00025
24.97	0.70942	0.70906	0.00019	32.33	0.70921	0.70956	0.00027
24.92	0.70933	0.70893	0.00024	32.29	0.70979	0.70957	0.00027
24.86	0.70901	0.70891	0.00022	32.24	0.70905	0.70955	0.00026
24.81	0.70860	0.70890	0.00024	32.20	0.70991	0.70960	0.00024
24.75	0.70931	0.70895	0.00023	32.15	0.70995	0.70952	0.00024
24.70	0.70911	0.70891	0.00021	32.11	0.70928	0.70943	0.00024
24.65	0.70887	0.70891	0.00021	32.06	0.71018	0.70946	0.00024
24.59	0.70930	0.70899	0.00025	32.02	0.70954	0.70946	0.00024
24.54	0.70862	0.70897	0.00024	31.97	0.70980	0.70949	0.00025
24.49	0.70900	0.70895	0.00025	31.93	0.70892	0.70941	0.00026
24.43	0.70818	0.70889	0.00027	31.88	0.70927	0.70952	0.00026
24.38	0.70913	0.70899	0.00021	31.84	0.70957	0.70955	0.00025
24.27	0.70907	0.70898	0.00021	31.80	0.70953	0.70956	0.00025
24.22	0.70893	0.70899	0.00021	31.75	0.70911	0.70956	0.00025
24.16	0.70915	0.70903	0.00022	31.71	0.70909	0.70959	0.00024
24.11	0.70955	0.70900	0.00022	31.66	0.70955	0.70963	0.00021
24.06	0.70907	0.70890	0.00021	31.62	0.71023	0.70967	0.00022
24.00	0.70847	0.70886	0.00021	31.57	0.70983	0.70962	0.00019
23.95	0.70849	0.70889	0.00019	31.53	0.70904	0.70958	0.00018
23.90	0.70903	0.70895	0.00018	31.48	0.71001	0.70962	0.00015
23.84	0.70880	0.70892	0.00018	31.44	0.70951	0.70961	0.00014
23.79	0.70922	0.70892	0.00018	31.39	0.70973	0.70964	0.00014
23.73	0.70921	0.70890	0.00017	31.35	0.70952	0.70965	0.00015
23.68	0.70933	0.70888	0.00016	31.31	0.70936	0.70972	0.00018
23.63	0.70887	0.70882	0.00013	31.26	0.70949	0.70976	0.00017
23.57	0.70850	0.70879	0.00013	31.22	0.70994	0.70973	0.00021
23.52	0.70862	0.70886	0.00015	31.17	0.70978	0.70966	0.00021
23.47	0.70878	0.70888	0.00014	31.13	0.70944	0.70961	0.00023
23.41	0.70916	0.70891	0.00014	31.08	0.70940	0.70962	0.00023
23.36	0.70874	0.70893	0.00017	31.04	0.70998	0.70960	0.00024
23.31	0.70875	0.70898	0.00017	30.99	0.70973	0.70952	0.00023
23.25	0.70905	0.70900	0.00016	30.95	0.70988	0.70950	0.00023
23.20	0.70898	0.70897	0.00017	30.91	0.71023	0.70946	0.00021
23.14	0.70875	0.70894	0.00018	30.86	0.70976	0.70942	0.00016
23.09	0.70856	0.70902	0.00021	30.82	0.70910	0.70940	0.00014
23.04	0.70926	0.70906	0.00019	30.77	0.70934	0.70940	0.00015

22.98	0.70877	0.70904	0.00018
22.93	0.70903	0.70907	0.00017
22.88	0.70940	0.70911	0.00019
22.82	0.70924	0.70905	0.00019
22.77	0.70895	0.70906	0.00019
22.71	0.70872	0.70905	0.00020
22.66	0.70870	0.70904	0.00021
22.61	0.70955	0.70910	0.00020
22.55	0.70893	0.70907	0.00017
22.50	0.70909	0.70908	0.00017
22.45	0.70903	0.70906	0.00018
22.39	0.70951	0.70910	0.00019
22.34	0.70880	0.70904	0.00016
22.28	0.70931	0.70908	0.00016
22.23	0.70888	0.70904	0.00015
22.18	0.70861	0.70907	0.00015
22.12	0.70928	0.70916	0.00013
22.07	0.70928	0.70914	0.00013
22.02	0.70903	0.70914	0.00012
21.96	0.70889	0.70914	0.00013
21.91	0.70937	0.70917	0.00011
21.86	0.70893	0.70915	0.00010
21.80	0.70918	0.70918	0.00009
21.75	0.70894	0.70916	0.00010
21.69	0.70919	0.70915	0.00011
21.64	0.70948	0.70915	0.00011
21.59	0.70912	0.70911	0.00008
21.53	0.70927	0.70913	0.00008
21.48	0.70903	0.70910	0.00008
21.43	0.70918	0.70908	0.00010
21.37	0.70924	0.70911	0.00012
21.32	0.70918	0.70905	0.00015
21.26	0.70903	0.70907	0.00016
21.21	0.70884	0.70909	0.00017
21.16	0.70919	0.70912	0.00016
21.10	0.70908	0.70912	0.00016
21.05	0.70925	0.70908	0.00017
21.00	0.70899	0.70909	0.00018
20.94	0.70884	0.70913	0.00019
20.89	0.70947	0.70911	0.00020
20.84	0.70863	0.70909	0.00019
20.78	0.70934	0.70917	0.00017
20.73	0.70930	0.70916	0.00017
20.67	0.70907	0.70917	0.00017
20.62	0.70918	0.70920	0.00018
20.57	0.70875	0.70920	0.00018
20.51	0.70927	0.70923	0.00015
20.46	0.70942	0.70920	0.00015
20.41	0.70867	0.70915	0.00016
20.35	0.70921	0.70921	0.00012

30.73	0.70923	0.70936	0.00018
30.68	0.70952	0.70942	0.00020
30.64	0.70921	0.70940	0.00019
30.59	0.70919	0.70940	0.00019
30.55	0.70957	0.70947	0.00021
30.50	0.70946	0.70946	0.00021
30.46	0.70983	0.70942	0.00023
30.42	0.70959	0.70943	0.00024
30.37	0.70906	0.70944	0.00025
30.33	0.70892	0.70955	0.00027
30.28	0.70982	0.70968	0.00026
30.24	0.70935	0.70966	0.00026
30.19	0.70921	0.70971	0.00025
30.15	0.70988	0.70976	0.00022
30.10	0.70953	0.70975	0.00022
30.06	0.70896	0.70976	0.00022
30.01	0.70994	0.70983	0.00013
29.97	0.70975	0.70976	0.00017
29.93	0.71014	0.70977	0.00016
29.88	0.71021	0.70971	0.00015
29.84	0.70967	0.70968	0.00011
29.79	0.70984	0.70969	0.00011
29.75	0.70972	0.70968	0.00011
29.70	0.70976	0.70969	0.00011
29.66	0.70964	0.70970	0.00011
29.61	0.70961	0.70974	0.00013
29.57	0.70930	0.70972	0.00014
29.52	0.70978	0.70972	0.00015
29.48	0.70956	0.70969	0.00015
29.44	0.70992	0.70969	0.00015
29.39	0.70975	0.70969	0.00015
29.35	0.70977	0.70969	0.00015
29.30	0.70981	0.70963	0.00018
29.26	0.70983	0.70961	0.00017
29.21	0.71003	0.70962	0.00018
29.17	0.70950	0.70953	0.00017
29.12	0.70923	0.70954	0.00018
29.08	0.70955	0.70962	0.00018
29.03	0.70956	0.70966	0.00019
28.99	0.70986	0.70961	0.00023
28.95	0.70977	0.70956	0.00022
28.90	0.70916	0.70959	0.00024
28.86	0.70965	0.70960	0.00023
28.81	0.70987	0.70964	0.00025
28.77	0.70914	0.70961	0.00025
28.72	0.70962	0.70969	0.00023
28.68	0.71002	0.70968	0.00024
28.63	0.70992	0.70966	0.00023
28.59	0.70905	0.70965	0.00022
28.54	0.70944	0.70970	0.00018

20.30	0.70947	0.70918	0.00013	28.50	0.71002	0.70970	0.00018
20.24	0.70929	0.70918	0.00013	28.46	0.70921	0.70970	0.00019
20.19	0.70940	0.70917	0.00012	28.41	0.71009	0.70970	0.00019
20.14	0.70937	0.70915	0.00011	28.37	0.70953	0.70962	0.00019
20.08	0.70914	0.70916	0.00012	28.32	0.70996	0.70962	0.00019
20.03	0.70907	0.70911	0.00015	28.28	0.70954	0.70965	0.00022
19.98	0.70901	0.70911	0.00015	28.23	0.70983	0.70966	0.00022
19.92	0.70889	0.70914	0.00015	28.19	0.70981	0.70961	0.00023
19.87	0.70928	0.70910	0.00019	28.14	0.70957	0.70963	0.00024
19.81	0.70892	0.70908	0.00019	28.10	0.70942	0.70967	0.00024
19.76	0.70941	0.70906	0.00019	28.06	0.71009	0.70961	0.00029
19.71	0.70918	0.70903	0.00018	28.01	0.70916	0.70961	0.00029
19.65	0.70925	0.70903	0.00018	27.97	0.70927	0.70961	0.00029
19.60	0.70943	0.70903	0.00018	27.92	0.70955	0.70969	0.00029
19.55	0.70866	0.70900	0.00016	27.88	0.71030	0.70971	0.00029
19.49	0.70913	0.70900	0.00015	27.83	0.70956	0.70970	0.00028
19.44	0.70923	0.70898	0.00015	27.79	0.70937	0.70971	0.00028
19.39	0.70855	0.70895	0.00014	27.74	0.70999	0.70971	0.00028
19.33	0.70905	0.70900	0.00011	27.70	0.70994	0.70960	0.00031
19.28	0.70876	0.70898	0.00011	27.65	0.70890	0.70952	0.00032
19.22	0.70911	0.70900	0.00010	27.61	0.71007	0.70963	0.00030
19.17	0.70918	0.70898	0.00010	27.57	0.70911	0.70957	0.00028
19.12	0.70922	0.70894	0.00009	27.52	0.71009	0.70962	0.00028
19.06	0.70906	0.70890	0.00007	27.48	0.70977	0.70957	0.00027
19.01	0.70873	0.70891	0.00008	27.43	0.71020	0.70954	0.00029
18.96	0.70892	0.70894	0.00007	27.39	0.70965	0.70942	0.00024
18.90	0.70891	0.70894	0.00007	27.34	0.70935	0.70938	0.00026
18.85	0.70905	0.70896	0.00008	27.30	0.70892	0.70939	0.00030
18.79	0.70888	0.70897	0.00008	27.25	0.70914	0.70954	0.00028
18.74	0.70888	0.70899	0.00008	27.21	0.71001	0.70974	0.00024
18.69	0.70893	0.70904	0.00011	27.16	0.70948	0.70948	0.00026
18.63	0.70878	0.70909	0.00013	26.41	0.70964	0.70933	0.00019
18.58	0.70883	0.70911	0.00012	26.36	0.70935	0.70930	0.00017
18.53	0.70918	0.70913	0.00011	26.32	0.70925	0.70930	0.00017
18.47	0.70903	0.70911	0.00011	26.27	0.70974	0.70937	0.00021
18.42	0.70898	0.70910	0.00011	26.23	0.70919	0.70931	0.00019
18.36	0.70911	0.70909	0.00012	26.18	0.70926	0.70934	0.00019
18.31	0.70907	0.70913	0.00014	26.14	0.70967	0.70933	0.00020
18.26	0.70913	0.70915	0.00014	26.10	0.70897	0.70928	0.00018
18.20	0.70935	0.70913	0.00015	26.05	0.70934	0.70930	0.00017
18.15	0.70948	0.70910	0.00014	26.01	0.70885	0.70931	0.00017
18.10	0.70895	0.70906	0.00011	25.96	0.70939	0.70940	0.00015
18.04	0.70902	0.70906	0.00012	25.92	0.70935	0.70940	0.00015
17.99	0.70900	0.70908	0.00012	25.87	0.70988	0.70940	0.00015
17.94	0.70896	0.70910	0.00013	25.83	0.70917	0.70935	0.00011
17.88	0.70886	0.70913	0.00012	25.78	0.70949	0.70933	0.00013
17.83	0.70943	0.70913	0.00012	25.74	0.70916	0.70932	0.00012
17.77	0.70931	0.70908	0.00010	25.69	0.70915	0.70935	0.00013
17.72	0.70893	0.70903	0.00011	25.65	0.70921	0.70938	0.00012
17.67	0.70903	0.70903	0.00011	25.61	0.70947	0.70944	0.00014

17.61	0.70915	0.70906	0.00012	25.56	0.70967	0.70949	0.00017
17.56	0.70887	0.70906	0.00012	25.52	0.70947	0.70949	0.00017
17.51	0.70925	0.70911	0.00013	25.47	0.70932	0.70953	0.00018
17.45	0.70924	0.70909	0.00013	25.43	0.70940	0.70955	0.00018
17.40	0.70919	0.70909	0.00013	25.38	0.70898	0.70956	0.00018
17.34	0.70889	0.70907	0.00013	25.34	0.70934	0.70963	0.00012
17.29	0.70896	0.70911	0.00013	25.29	0.70951	0.70968	0.00010
17.24	0.70876	0.70918	0.00016	25.25	0.70946	0.70965	0.00014
17.18	0.70902	0.70918	0.00015	25.21	0.70981	0.70966	0.00013
17.13	0.70932	0.70917	0.00016	25.16	0.70992	0.70961	0.00014
17.08	0.70908	0.70915	0.00016	25.12	0.70967	0.70959	0.00013
17.02	0.70942	0.70911	0.00017	25.07	0.70987	0.70960	0.00013
16.97	0.70902	0.70908	0.00016	25.03	0.70957	0.70951	0.00016
16.92	0.70927	0.70907	0.00016	24.98	0.70949	0.70947	0.00017
16.86	0.70898	0.70902	0.00016	24.94	0.70970	0.70944	0.00018
16.81	0.70932	0.70903	0.00016	24.89	0.70976	0.70942	0.00017
16.75	0.70959	0.70902	0.00016	24.85	0.70922	0.70943	0.00019
16.70	0.70881	0.70896	0.00009	24.80	0.70960	0.70949	0.00019
16.65	0.70892	0.70900	0.00010	24.76	0.70929	0.70949	0.00019
16.59	0.70905	0.70903	0.00011	24.72	0.70972	0.70947	0.00021
16.54	0.70876	0.70904	0.00011	24.67	0.70976	0.70944	0.00020
16.49	0.70906	0.70905	0.00010	24.63	0.70900	0.70940	0.00019
16.43	0.70892	0.70906	0.00011	24.58	0.70920	0.70946	0.00017
16.38	0.70883	0.70909	0.00011	24.54	0.70919	0.70947	0.00016
16.32	0.70904	0.70913	0.00009	24.49	0.70945	0.70949	0.00015
16.27	0.70924	0.70915	0.00009	24.45	0.70992	0.70952	0.00016
16.22	0.70894	0.70914	0.00009	24.40	0.70975	0.70943	0.00016
16.16	0.70922	0.70915	0.00008	24.36	0.70959	0.70942	0.00015
16.11	0.70928	0.70913	0.00009	24.31	0.70909	0.70940	0.00014
16.06	0.70909	0.70913	0.00009	24.27	0.70945	0.70941	0.00013
16.00	0.70886	0.70911	0.00010	24.23	0.70937	0.70946	0.00017
15.95	0.70919	0.70907	0.00016	24.18	0.70956	0.70946	0.00017
15.89	0.70924	0.70912	0.00020	24.14	0.70929	0.70943	0.00017
15.84	0.70916	0.70909	0.00020	24.09	0.70941	0.70946	0.00017
15.79	0.70925	0.70906	0.00020	24.05	0.70976	0.70945	0.00017
15.73	0.70916	0.70905	0.00020	24.00	0.70905	0.70945	0.00017
15.68	0.70909	0.70904	0.00020	23.96	0.70966	0.70954	0.00018
15.63	0.70893	0.70906	0.00020	23.91	0.70935	0.70949	0.00020
15.57	0.70931	0.70910	0.00020	23.87	0.70921	0.70947	0.00021
15.52	0.70892	0.70911	0.00021	23.82	0.70992	0.70949	0.00020
15.47	0.70845	0.70914	0.00021	23.78	0.70938	0.70947	0.00018
15.41	0.70965	0.70923	0.00014	23.74	0.70928	0.70951	0.00019
15.36	0.70895	0.70912	0.00017	23.69	0.70959	0.70953	0.00019
15.30	0.70892	0.70911	0.00017	23.65	0.70926	0.70949	0.00020
15.25	0.70911	0.70916	0.00017	23.60	0.70977	0.70948	0.00020
15.20	0.70909	0.70913	0.00019	23.56	0.71002	0.70939	0.00022
15.14	0.70929	0.70915	0.00019	23.51	0.70909	0.70931	0.00017
15.09	0.70927	0.70915	0.00019	23.47	0.70921	0.70936	0.00017
15.04	0.70942	0.70917	0.00020	23.42	0.70942	0.70938	0.00017
14.98	0.70928	0.70914	0.00020	23.38	0.70971	0.70939	0.00017

14.93	0.70933	0.70921	0.00026
14.87	0.70851	0.70918	0.00026
14.82	0.70893	0.70920	0.00023
14.77	0.70939	0.70923	0.00022
14.71	0.70882	0.70920	0.00022
14.66	0.70923	0.70919	0.00023
14.61	0.70931	0.70918	0.00023
14.55	0.70950	0.70910	0.00026
14.44	0.70987	0.70900	0.00023
14.39	0.70906	0.70894	0.00015
14.34	0.70872	0.70896	0.00016
14.28	0.70921	0.70897	0.00015
14.23	0.70911	0.70902	0.00020
14.18	0.70873	0.70901	0.00020
14.12	0.70911	0.70906	0.00020
14.07	0.70858	0.70906	0.00020
14.02	0.70892	0.70912	0.00017
13.96	0.70873	0.70916	0.00016
13.91	0.70924	0.70921	0.00014
13.85	0.70925	0.70922	0.00014
13.80	0.70885	0.70918	0.00015
13.75	0.70967	0.70924	0.00014
13.69	0.70901	0.70919	0.00011
13.64	0.70927	0.70916	0.00014
13.59	0.70909	0.70918	0.00015
13.53	0.70916	0.70919	0.00015
13.48	0.70929	0.70918	0.00015
13.42	0.70928	0.70918	0.00015
13.37	0.70928	0.70918	0.00015
13.32	0.70886	0.70916	0.00015
13.26	0.70944	0.70918	0.00014
13.21	0.70925	0.70911	0.00016
13.16	0.70870	0.70907	0.00016
13.10	0.70945	0.70913	0.00015
13.05	0.70922	0.70907	0.00014
13.00	0.70902	0.70910	0.00017
12.94	0.70928	0.70906	0.00019
12.89	0.70926	0.70899	0.00021
12.83	0.70909	0.70897	0.00020
12.78	0.70912	0.70898	0.00021
12.73	0.70870	0.70898	0.00021
12.67	0.70881	0.70903	0.00020
12.62	0.70937	0.70905	0.00020
12.57	0.70882	0.70902	0.00019
12.51	0.70953	0.70903	0.00019
12.46	0.70867	0.70899	0.00015
12.40	0.70850	0.70901	0.00014
12.35	0.70907	0.70907	0.00008
12.30	0.70923	0.70906	0.00008
12.24	0.70911	0.70904	0.00007

23.33	0.70978	0.70943	0.00022
23.29	0.70944	0.70939	0.00020
23.25	0.70921	0.70936	0.00021
23.20	0.70914	0.70940	0.00022
23.16	0.70889	0.70938	0.00023
23.11	0.70925	0.70941	0.00021
23.07	0.70953	0.70946	0.00021
23.02	0.70948	0.70950	0.00023
22.98	0.70952	0.70952	0.00023
22.93	0.71011	0.70949	0.00024
22.89	0.70937	0.70944	0.00020
22.84	0.70906	0.70948	0.00021
22.80	0.70965	0.70956	0.00020
22.76	0.70895	0.70952	0.00021
22.71	0.70924	0.70960	0.00017
22.67	0.70973	0.70965	0.00015
22.62	0.70993	0.70967	0.00017
22.58	0.70963	0.70963	0.00016
22.53	0.70925	0.70963	0.00016
22.49	0.70960	0.70966	0.00013
22.44	0.70976	0.70965	0.00014
22.40	0.70987	0.70962	0.00014
22.36	0.70921	0.70956	0.00015
22.31	0.70978	0.70962	0.00014
22.27	0.70975	0.70965	0.00016
22.22	0.70995	0.70961	0.00016
22.18	0.70955	0.70956	0.00015
22.13	0.70962	0.70959	0.00016
22.09	0.70956	0.70956	0.00017
22.04	0.70941	0.70969	0.00031
22.00	0.70955	0.70971	0.00030
21.95	0.70922	0.70978	0.00032
21.91	0.70983	0.70977	0.00032
21.87	0.71003	0.70976	0.00032
21.82	0.70943	0.70971	0.00032
21.78	0.70936	0.70974	0.00032
21.73	0.70985	0.70980	0.00031
21.69	0.70934	0.70978	0.00031
21.64	0.71085	0.70976	0.00032
21.60	0.70958	0.70960	0.00022
21.55	0.71027	0.70963	0.00023
21.51	0.70917	0.70957	0.00018
21.46	0.70967	0.70956	0.00019
21.42	0.70953	0.70960	0.00021
21.38	0.70981	0.70960	0.00021
21.33	0.70991	0.70963	0.00023
21.29	0.70969	0.70958	0.00023
21.24	0.70913	0.70957	0.00022
21.20	0.70925	0.70963	0.00020
21.15	0.70988	0.70971	0.00020

12.19	0.70923	0.70901	0.00009	21.11	0.70966	0.70972	0.00020
12.14	0.70898	0.70899	0.00008	21.06	0.70911	0.70970	0.00021
12.08	0.70908	0.70901	0.00008	21.02	0.71004	0.70979	0.00017
12.03	0.70886	0.70905	0.00012	20.97	0.70952	0.70971	0.00019
11.97	0.70917	0.70909	0.00012	20.93	0.71008	0.70975	0.00019
11.92	0.70888	0.70910	0.00013	20.89	0.70940	0.70978	0.00022
11.87	0.70907	0.70911	0.00012	20.84	0.70963	0.70984	0.00021
11.81	0.70902	0.70913	0.00013	20.80	0.70975	0.70993	0.00024
11.76	0.70901	0.70913	0.00013	20.75	0.71007	0.70994	0.00024
11.71	0.70878	0.70915	0.00013	20.71	0.70994	0.70988	0.00025
11.65	0.70907	0.70920	0.00010	20.66	0.70943	0.70987	0.00025
11.60	0.70916	0.70917	0.00012	20.62	0.71000	0.70988	0.00024
11.55	0.70943	0.70919	0.00013	20.57	0.70926	0.70982	0.00026
11.49	0.70929	0.70914	0.00014	20.53	0.70993	0.70983	0.00026
11.44	0.70931	0.70913	0.00014	20.48	0.71037	0.70985	0.00026
11.38	0.70891	0.70912	0.00013	20.44	0.71004	0.70971	0.00029
11.33	0.70932	0.70914	0.00012	20.40	0.71051	0.70966	0.00028
11.28	0.70907	0.70910	0.00012	20.35	0.70988	0.70959	0.00022
11.22	0.70920	0.70912	0.00012	20.31	0.70948	0.70952	0.00022
11.17	0.70922	0.70914	0.00014	20.26	0.70978	0.70950	0.00022
11.12	0.70879	0.70910	0.00015	20.22	0.70959	0.70951	0.00023
11.06	0.70942	0.70915	0.00014	20.17	0.70932	0.70953	0.00023
11.01	0.70884	0.70909	0.00014	20.13	0.70938	0.70955	0.00023
10.95	0.70920	0.70913	0.00014	20.08	0.71016	0.70957	0.00023
10.90	0.70920	0.70913	0.00014	20.04	0.70892	0.70960	0.00026
10.85	0.70916	0.70913	0.00013	19.99	0.70962	0.70962	0.00024
10.79	0.70894	0.70913	0.00013	19.95	0.70975	0.70964	0.00025
10.74	0.70919	0.70918	0.00014	19.91	0.70922	0.70957	0.00028
10.69	0.70942	0.70924	0.00019	19.86	0.70928	0.70962	0.00027
10.63	0.70879	0.70922	0.00018	19.82	0.70987	0.70964	0.00026
10.58	0.70935	0.70922	0.00018	19.77	0.70979	0.70959	0.00026
10.52	0.70882	0.70922	0.00018	19.73	0.70951	0.70964	0.00030
10.47	0.70925	0.70929	0.00016	19.68	0.70964	0.70966	0.00030
10.42	0.70920	0.70930	0.00016	19.64	0.71044	0.70969	0.00030
10.36	0.70918	0.70931	0.00016	19.59	0.70910	0.70965	0.00027
10.31	0.70917	0.70934	0.00016	19.55	0.70985	0.70976	0.00025
10.26	0.70946	0.70937	0.00016	19.50	0.70899	0.70974	0.00025
10.20	0.70978	0.70932	0.00017	19.46	0.70969	0.70977	0.00021
10.15	0.70922	0.70930	0.00015	19.42	0.70951	0.70980	0.00022
10.10	0.70882	0.70936	0.00017	19.37	0.70933	0.70985	0.00021
10.04	0.70935	0.70941	0.00013	19.33	0.71031	0.70987	0.00020
9.99	0.70945	0.70943	0.00013	19.28	0.70975	0.70982	0.00017
9.93	0.70935	0.70938	0.00016	19.24	0.70990	0.70977	0.00021
9.88	0.70931	0.70939	0.00016	19.19	0.71011	0.70973	0.00021
9.83	0.70952	0.70938	0.00017	19.15	0.71013	0.70967	0.00020
9.77	0.70942	0.70937	0.00016	19.10	0.70970	0.70964	0.00018
9.72	0.70900	0.70934	0.00018	19.06	0.70932	0.70965	0.00018
9.67	0.70956	0.70935	0.00017	19.02	0.70995	0.70966	0.00017
9.61	0.70978	0.70931	0.00016	18.97	0.71006	0.70962	0.00016
9.56	0.70934	0.70928	0.00013	18.93	0.70947	0.70954	0.00014

9.50	0.70957	0.70927	0.00013	18.88	0.70986	0.70955	0.00015
9.45	0.70894	0.70930	0.00017	18.84	0.70924	0.70951	0.00014
9.40	0.70948	0.70930	0.00016	18.79	0.70948	0.70955	0.00013
9.34	0.70923	0.70926	0.00016	18.75	0.70946	0.70956	0.00014
9.29	0.70943	0.70924	0.00017	18.70	0.70989	0.70958	0.00015
9.24	0.70903	0.70920	0.00017	18.66	0.70973	0.70950	0.00012
9.18	0.70910	0.70925	0.00017	18.61	0.70943	0.70942	0.00009
9.13	0.70919	0.70925	0.00018	18.57	0.70955	0.70941	0.00012
9.08	0.70948	0.70922	0.00019	18.53	0.70928	0.70928	0.00016
9.02	0.70925	0.70923	0.00019	17.72	0.70971	0.70978	0.00015
8.97	0.70983	0.70928	0.00022	17.68	0.71004	0.70982	0.00015
8.91	0.70901	0.70921	0.00018	17.63	0.70992	0.70974	0.00018
8.86	0.70901	0.70928	0.00021	17.59	0.70983	0.70977	0.00021
8.81	0.70909	0.70930	0.00020	17.55	0.70968	0.70980	0.00022
8.75	0.70904	0.70930	0.00020	17.50	0.71019	0.70980	0.00022
8.70	0.70951	0.70934	0.00019	17.46	0.70948	0.70980	0.00022
8.65	0.70903	0.70928	0.00021	17.41	0.70989	0.70980	0.00022
8.59	0.70893	0.70926	0.00022	17.37	0.70959	0.70977	0.00022
8.54	0.70960	0.70923	0.00024	17.32	0.70952	0.70982	0.00022
8.48	0.70974	0.70916	0.00023	17.28	0.71005	0.70983	0.00022
8.43	0.70910	0.70910	0.00019	17.23	0.70922	0.70982	0.00022
8.38	0.70975	0.70910	0.00019	17.19	0.71022	0.70990	0.00018
8.32	0.70914	0.70907	0.00015	17.14	0.71011	0.70988	0.00017
8.27	0.70919	0.70904	0.00016	17.10	0.70972	0.70983	0.00016
8.22	0.70939	0.70900	0.00016	17.06	0.71017	0.70984	0.00016
8.16	0.70887	0.70896	0.00013	17.01	0.70949	0.70985	0.00017
8.11	0.70885	0.70904	0.00018	16.97	0.70959	0.70982	0.00019
8.05	0.70866	0.70907	0.00018	16.92	0.71010	0.70983	0.00019
8.00	0.70894	0.70915	0.00017	16.88	0.70957	0.70977	0.00019
7.95	0.70914	0.70912	0.00019	16.83	0.70997	0.70975	0.00020
7.89	0.70908	0.70917	0.00022	16.79	0.71010	0.70972	0.00020
7.84	0.70940	0.70921	0.00023	16.74	0.70998	0.70973	0.00020
7.79	0.70886	0.70919	0.00022	16.70	0.70961	0.70967	0.00020
7.73	0.70880	0.70925	0.00022	16.65	0.70985	0.70971	0.00022
7.68	0.70904	0.70927	0.00020	16.61	0.71020	0.70971	0.00022
7.63	0.70962	0.70930	0.00020	16.57	0.70924	0.70959	0.00024
7.57	0.70920	0.70920	0.00022	16.52	0.70968	0.70961	0.00022
7.52	0.70942	0.70920	0.00022	16.48	0.70949	0.70960	0.00022
7.46	0.70864	0.70924	0.00026	16.43	0.70937	0.70971	0.00030
7.41	0.70965	0.70934	0.00023	16.39	0.70970	0.70986	0.00037
7.36	0.70950	0.70920	0.00031	16.34	0.71015	0.70983	0.00038
7.30	0.70916	0.70922	0.00032	16.30	0.70937	0.70970	0.00041
7.25	0.70947	0.70916	0.00035	16.25	0.71004	0.70971	0.00041
7.20	0.70897	0.70919	0.00036	16.21	0.70987	0.70969	0.00040
7.14	0.70935	0.70920	0.00036	16.17	0.70895	0.70971	0.00041
7.09	0.70867	0.70923	0.00037	16.12	0.70947	0.70973	0.00039
7.03	0.70912	0.70928	0.00035	16.08	0.70956	0.70972	0.00040
6.98	0.70992	0.70924	0.00036	16.03	0.71060	0.70972	0.00040
6.93	0.70963	0.70922	0.00035	15.99	0.71087	0.70960	0.00035
6.87	0.70822	0.70914	0.00035	15.94	0.70939	0.70947	0.00021

6.82	0.70972	0.70917	0.00031
6.77	0.70855	0.70914	0.00029
6.71	0.70973	0.70919	0.00026
6.66	0.70911	0.70914	0.00023
6.60	0.70965	0.70915	0.00023
6.55	0.70911	0.70916	0.00024
6.50	0.70880	0.70929	0.00034
6.44	0.70972	0.70936	0.00032
6.39	0.70878	0.70925	0.00035
6.34	0.70856	0.70930	0.00033
6.28	0.70936	0.70936	0.00029
6.23	0.70911	0.70936	0.00031
6.18	0.70925	0.70940	0.00032
6.12	0.70922	0.70942	0.00035
6.07	0.70970	0.70945	0.00037
6.01	0.71038	0.70940	0.00041
5.96	0.70950	0.70916	0.00025
5.91	0.70860	0.70904	0.00025
5.85	0.70935	0.70920	0.00008
5.80	0.70917	0.70903	0.00012
5.37	0.70908	0.70929	0.00028
5.32	0.70882	0.70932	0.00028
5.26	0.70937	0.70939	0.00026
5.21	0.70935	0.70943	0.00027
5.16	0.70975	0.70945	0.00027
5.10	0.70869	0.70939	0.00027
5.05	0.70966	0.70939	0.00027
4.99	0.70980	0.70935	0.00026
4.94	0.70868	0.70929	0.00024
4.89	0.70974	0.70939	0.00021
4.83	0.70938	0.70936	0.00020
4.78	0.70950	0.70928	0.00026
4.73	0.70977	0.70924	0.00026
4.67	0.70954	0.70923	0.00025
4.62	0.70911	0.70919	0.00024
4.56	0.70868	0.70922	0.00025
4.51	0.70927	0.70926	0.00022
4.46	0.70924	0.70923	0.00023
4.40	0.70969	0.70921	0.00023
4.35	0.70946	0.70917	0.00021
4.30	0.70852	0.70920	0.00024
4.24	0.70909	0.70930	0.00019
4.19	0.70970	0.70931	0.00018
4.13	0.70916	0.70921	0.00020
4.08	0.70943	0.70921	0.00020
4.03	0.70908	0.70920	0.00019
3.97	0.70893	0.70919	0.00020
3.92	0.70903	0.70920	0.00019
3.87	0.70932	0.70926	0.00020
3.81	0.70977	0.70926	0.00020

15.90	0.70890	0.70946	0.00021
15.85	0.70945	0.70959	0.00022
15.81	0.70982	0.70966	0.00024
15.76	0.71009	0.70963	0.00024
15.72	0.70917	0.70951	0.00026
15.68	0.70932	0.70957	0.00025
15.63	0.70954	0.70956	0.00026
15.59	0.70939	0.70955	0.00026
15.54	0.70966	0.70957	0.00026
15.50	0.70927	0.70961	0.00028
15.45	0.71021	0.70955	0.00033
15.41	0.71010	0.70944	0.00031
15.36	0.70955	0.70936	0.00027
15.32	0.70887	0.70937	0.00027
15.27	0.70979	0.70944	0.00025
15.23	0.70919	0.70945	0.00026
15.19	0.70945	0.70952	0.00026
15.14	0.70959	0.70957	0.00028
15.10	0.71008	0.70954	0.00028
15.05	0.70866	0.70949	0.00026
15.01	0.70909	0.70960	0.00018
14.96	0.70936	0.70965	0.00014
14.92	0.70965	0.70966	0.00013
14.87	0.70954	0.70959	0.00020
14.83	0.70992	0.70962	0.00020
14.78	0.70986	0.70953	0.00022
14.74	0.70994	0.70953	0.00022
14.70	0.70926	0.70951	0.00020
14.65	0.70966	0.70951	0.00020
14.61	0.70973	0.70949	0.00020
14.56	0.70958	0.70947	0.00019
14.52	0.70949	0.70945	0.00019
14.47	0.70894	0.70940	0.00022
14.43	0.70978	0.70944	0.00020
14.38	0.70904	0.70950	0.00027
14.34	0.70992	0.70958	0.00026
14.29	0.70966	0.70956	0.00025
14.25	0.70930	0.70961	0.00027
14.21	0.70951	0.70962	0.00027
14.16	0.70949	0.70963	0.00028
14.12	0.70942	0.70965	0.00030
14.07	0.70892	0.70968	0.00032
14.03	0.70935	0.70981	0.00026
13.98	0.71044	0.70990	0.00024
13.94	0.70981	0.70977	0.00017
13.89	0.70972	0.70975	0.00021
13.85	0.71010	0.70977	0.00029
13.80	0.70945	0.70945	0.00027
13.00	0.71023	0.70957	0.00026
12.96	0.70942	0.70951	0.00021

3.76	0.70953	0.70914	0.00021	12.91	0.70897	0.70953	0.00021
3.71	0.70912	0.70906	0.00021	12.87	0.70912	0.70959	0.00017
3.65	0.70870	0.70903	0.00021	12.83	0.70955	0.70958	0.00019
3.60	0.70917	0.70909	0.00021	12.78	0.70954	0.70955	0.00020
3.54	0.70931	0.70912	0.00022	12.74	0.70979	0.70957	0.00020
3.49	0.70898	0.70905	0.00024	12.69	0.71001	0.70954	0.00020
3.44	0.70907	0.70911	0.00026	12.65	0.70983	0.70947	0.00017
3.38	0.70962	0.70913	0.00026	12.60	0.70919	0.70943	0.00016
3.33	0.70938	0.70909	0.00024	12.56	0.70970	0.70942	0.00016
3.28	0.70855	0.70911	0.00025	12.51	0.70957	0.70939	0.00015
3.22	0.70876	0.70919	0.00023	12.47	0.70963	0.70935	0.00015
3.17	0.70880	0.70923	0.00021	12.42	0.70896	0.70940	0.00021
3.11	0.70930	0.70930	0.00019	12.38	0.70930	0.70941	0.00019
3.06	0.70944	0.70927	0.00020	12.34	0.70971	0.70943	0.00019
3.01	0.70859	0.70918	0.00023	12.29	0.70953	0.70941	0.00018
2.95	0.70963	0.70923	0.00020	12.25	0.70927	0.70937	0.00019
2.90	0.70923	0.70915	0.00018	12.20	0.70943	0.70943	0.00021
2.85	0.70922	0.70916	0.00019	12.16	0.70914	0.70943	0.00021
2.79	0.70955	0.70918	0.00020	12.11	0.70932	0.70944	0.00020
2.74	0.70941	0.70916	0.00018	12.07	0.70918	0.70947	0.00020
2.68	0.70912	0.70915	0.00018	12.02	0.71012	0.70952	0.00019
2.63	0.70947	0.70920	0.00020	11.98	0.70914	0.70943	0.00014
2.58	0.70901	0.70917	0.00019	11.93	0.70947	0.70946	0.00013
2.52	0.70859	0.70923	0.00020	11.89	0.70947	0.70951	0.00016
2.47	0.70903	0.70931	0.00015	11.85	0.70919	0.70951	0.00016
2.42	0.70888	0.70938	0.00015	11.80	0.70985	0.70955	0.00015
2.36	0.70928	0.70942	0.00011	11.76	0.70943	0.70951	0.00013
2.31	0.70946	0.70940	0.00012	11.71	0.70923	0.70956	0.00016
2.26	0.70929	0.70936	0.00013	11.67	0.70963	0.70962	0.00015
2.20	0.70935	0.70935	0.00014	11.62	0.70961	0.70963	0.00015
2.15	0.70964	0.70931	0.00016	11.58	0.70923	0.70962	0.00016
2.09	0.70918	0.70928	0.00014	11.53	0.70949	0.70963	0.00014
2.04	0.70959	0.70934	0.00017	11.49	0.70994	0.70961	0.00016
1.99	0.70941	0.70928	0.00016	11.44	0.70950	0.70950	0.00021
1.93	0.70968	0.70928	0.00016	11.40	0.70963	0.70951	0.00022
1.88	0.70927	0.70917	0.00018	11.36	0.70939	0.70949	0.00022
1.83	0.70911	0.70916	0.00018	11.31	0.71000	0.70949	0.00022
1.77	0.70913	0.70918	0.00018	11.27	0.70974	0.70944	0.00018
1.72	0.70910	0.70919	0.00018	11.22	0.70982	0.70942	0.00018
1.66	0.70903	0.70925	0.00021	11.18	0.70947	0.70943	0.00019
1.61	0.70928	0.70918	0.00027	11.13	0.70937	0.70942	0.00019
1.56	0.70976	0.70919	0.00027	11.09	0.70925	0.70945	0.00019
1.50	0.70905	0.70909	0.00025	11.04	0.70878	0.70945	0.00019
1.45	0.70935	0.70911	0.00025	11.00	0.70967	0.70956	0.00014
1.40	0.70864	0.70906	0.00025	10.95	0.70938	0.70954	0.00014
1.34	0.70917	0.70916	0.00025	10.91	0.70940	0.70958	0.00015
1.29	0.70930	0.70919	0.00025	10.87	0.70949	0.70958	0.00015
1.24	0.70923	0.70918	0.00025	10.82	0.70958	0.70963	0.00016
1.18	0.70970	0.70916	0.00025	10.78	0.70990	0.70960	0.00018
1.13	0.70836	0.70913	0.00023	10.73	0.70932	0.70953	0.00017

1.07	0.70933	0.70917	0.00018
1.02	0.70883	0.70909	0.00021
0.97	0.70922	0.70913	0.00020
0.91	0.70887	0.70914	0.00020
0.86	0.70960	0.70919	0.00020
0.81	0.70945	0.70915	0.00018
0.75	0.70918	0.70907	0.00019
0.70	0.70905	0.70907	0.00019
0.64	0.70938	0.70904	0.00020
0.59	0.70875	0.70900	0.00018
0.54	0.70859	0.70903	0.00018
0.48	0.70925	0.70908	0.00015
0.43	0.70923	0.70906	0.00016
0.38	0.70936	0.70903	0.00017
0.32	0.70921	0.70898	0.00015
0.27	0.70866	0.70893	0.00015
0.21	0.70921	0.70900	0.00013
0.16	0.70873	0.70893	0.00011
0.11	0.70902	0.70903	0.00001
0.05	0.70903		

10.69	0.70971	0.70958	0.00017
10.64	0.70930	0.70957	0.00017
10.60	0.70989	0.70960	0.00016
10.55	0.70941	0.70958	0.00014
10.51	0.70980	0.70961	0.00014
10.46	0.70943	0.70958	0.00014
10.42	0.70994	0.70964	0.00016
10.38	0.70926	0.70959	0.00014
10.33	0.70928	0.70961	0.00013
10.29	0.70977	0.70960	0.00014
10.24	0.70962	0.70956	0.00015
10.20	0.70964	0.70959	0.00017
10.15	0.70964	0.70964	0.00020
10.11	0.70978	0.70962	0.00020
10.06	0.70947	0.70962	0.00020
10.02	0.71000	0.70962	0.00020
9.98	0.70950	0.70961	0.00019
9.93	0.70945	0.70965	0.00020
9.89	0.70918	0.70968	0.00019
9.84	0.70932	0.70974	0.00016
9.80	0.70997	0.70982	0.00014
9.75	0.71009	0.70977	0.00015
9.71	0.70944	0.70972	0.00014
9.66	0.70982	0.70976	0.00013
9.62	0.70948	0.70974	0.00013
9.57	0.70984	0.70971	0.00016
9.53	0.70992	0.70972	0.00016
9.49	0.70975	0.70968	0.00016
9.44	0.70977	0.70968	0.00016
9.40	0.71011	0.70972	0.00019
9.35	0.70947	0.70971	0.00018
9.31	0.70958	0.70972	0.00018
9.26	0.70987	0.70975	0.00017
9.22	0.70964	0.70975	0.00018
9.17	0.70918	0.70978	0.00018
9.13	0.70986	0.70986	0.00012
9.08	0.70959	0.70984	0.00013
9.04	0.70969	0.70978	0.00020
9.00	0.71021	0.70981	0.00020
8.95	0.70999	0.70977	0.00018
8.91	0.70960	0.70975	0.00018
8.86	0.70985	0.70981	0.00019
8.82	0.70992	0.70983	0.00020
8.77	0.70996	0.70977	0.00023
8.73	0.70991	0.70973	0.00022
8.68	0.70966	0.70970	0.00022
8.64	0.70905	0.70971	0.00022
8.59	0.71000	0.70977	0.00017
8.55	0.70979	0.70970	0.00018
8.51	0.70981	0.70970	0.00018

8.46	0.71018	0.70971	0.00018
8.42	0.71006	0.70962	0.00016
8.37	0.70927	0.70957	0.00013
8.33	0.70961	0.70956	0.00014
8.28	0.70961	0.70956	0.00014
8.24	0.70975	0.70955	0.00014
8.19	0.70963	0.70959	0.00018
8.15	0.70933	0.70961	0.00019
8.10	0.70970	0.70959	0.00021
8.06	0.70993	0.70958	0.00021
8.02	0.70933	0.70959	0.00021
7.97	0.70958	0.70963	0.00021
7.93	0.70917	0.70967	0.00022
7.88	0.70961	0.70971	0.00019
7.84	0.70950	0.70975	0.00020
7.79	0.71012	0.70980	0.00020
7.75	0.70989	0.70974	0.00019
7.70	0.70907	0.70973	0.00019
7.66	0.70962	0.70985	0.00016
7.61	0.70998	0.70989	0.00015
7.57	0.70972	0.70987	0.00015
7.53	0.71002	0.70988	0.00015
7.48	0.70961	0.70984	0.00015
7.44	0.70993	0.70982	0.00017
7.39	0.71006	0.70982	0.00016
7.35	0.70946	0.70980	0.00016
7.30	0.70987	0.70989	0.00017
7.26	0.71028	0.70990	0.00017
7.21	0.70995	0.70989	0.00016
7.17	0.70983	0.70992	0.00018
7.13	0.70976	0.70992	0.00017
7.08	0.70966	0.70991	0.00018
7.04	0.70942	0.70991	0.00018
6.99	0.70987	0.70995	0.00015
6.95	0.70992	0.70999	0.00015
6.90	0.71032	0.70998	0.00016
6.86	0.70997	0.70991	0.00015
6.81	0.71022	0.70993	0.00016
6.77	0.71022	0.70987	0.00016
6.72	0.70988	0.70986	0.00015
6.68	0.70963	0.70976	0.00025
6.64	0.70966	0.70970	0.00028
6.59	0.70985	0.70973	0.00029
6.55	0.71020	0.70969	0.00029
6.50	0.70986	0.70967	0.00028
6.46	0.70965	0.70970	0.00029
6.41	0.71016	0.70970	0.00029
6.37	0.70959	0.70966	0.00027
6.32	0.71014	0.70971	0.00028
6.28	0.70884	0.70971	0.00028

6.23	0.70911	0.70982	0.00021
6.19	0.70996	0.70990	0.00014
6.15	0.70945	0.70987	0.00015
6.10	0.70995	0.70991	0.00012
6.06	0.71013	0.70986	0.00015
6.01	0.70973	0.70983	0.00014
5.97	0.70971	0.70986	0.00014
5.92	0.71011	0.70987	0.00014
5.88	0.71013	0.70985	0.00013
5.83	0.70988	0.70984	0.00012
5.79	0.70999	0.70987	0.00014
5.74	0.70958	0.70985	0.00014
5.70	0.70988	0.70987	0.00013
5.66	0.70947	0.70988	0.00013
5.61	0.70985	0.70994	0.00010
5.57	0.71003	0.70992	0.00011
5.52	0.70973	0.70993	0.00012
5.48	0.70994	0.70994	0.00011
5.43	0.71004	0.70994	0.00011
5.39	0.71020	0.70995	0.00012
5.34	0.70974	0.70994	0.00011
5.30	0.70980	0.70996	0.00010
5.25	0.70998	0.71000	0.00010
5.21	0.71010	0.71006	0.00016
5.17	0.70969	0.71003	0.00016
5.12	0.71011	0.71006	0.00015
5.08	0.70978	0.71004	0.00015
5.03	0.70994	0.71008	0.00014
4.99	0.71014	0.71006	0.00015
4.94	0.71014	0.71002	0.00016
4.90	0.70996	0.71000	0.00016
4.85	0.71013	0.70996	0.00018
4.81	0.71061	0.70996	0.00018
4.76	0.70985	0.70992	0.00013
4.72	0.70992	0.70991	0.00013
4.68	0.70995	0.70990	0.00013
4.63	0.71013	0.70992	0.00014
4.59	0.70980	0.70993	0.00015
4.54	0.70975	0.70994	0.00015
4.50	0.70990	0.71001	0.00017
4.45	0.70955	0.70998	0.00019
4.41	0.71014	0.71000	0.00017
4.36	0.71019	0.70999	0.00017
4.32	0.70982	0.70995	0.00016
4.28	0.70975	0.70996	0.00016
4.23	0.71020	0.70997	0.00016
4.19	0.71024	0.70993	0.00015
4.14	0.70985	0.70985	0.00017
4.10	0.71044	0.70985	0.00017
4.05	0.70962	0.70980	0.00011

4.01	0.70979	0.70982	0.00010
3.96	0.70997	0.70981	0.00010
3.92	0.70980	0.70981	0.00010
3.87	0.70990	0.70989	0.00019
3.83	0.70988	0.70990	0.00019
3.79	0.70979	0.70988	0.00020
3.74	0.70940	0.70991	0.00020
3.70	0.70994	0.70999	0.00017
3.65	0.70989	0.70999	0.00017
3.61	0.70978	0.70997	0.00018
3.56	0.70977	0.70998	0.00018
3.52	0.70996	0.70996	0.00019
3.47	0.71063	0.70998	0.00019
3.43	0.70996	0.70990	0.00014
3.38	0.70967	0.70990	0.00014
3.34	0.71014	0.70992	0.00013
3.30	0.71015	0.70986	0.00013
3.25	0.70990	0.70983	0.00011
3.21	0.70976	0.70982	0.00011
3.16	0.70981	0.70986	0.00012
3.12	0.70959	0.70986	0.00012
3.07	0.71015	0.70992	0.00012
2.98	0.70997	0.70997	0.00015
2.94	0.70982	0.71001	0.00017
2.89	0.70959	0.71004	0.00016
2.85	0.70990	0.71012	0.00014
2.81	0.70984	0.71015	0.00013
2.76	0.71009	0.71020	0.00012
2.72	0.70977	0.71024	0.00013
2.67	0.71014	0.71023	0.00014
2.63	0.71038	0.71026	0.00014
2.58	0.71021	0.71022	0.00015
2.54	0.71036	0.71024	0.00015
2.49	0.71016	0.71024	0.00015
2.45	0.71038	0.71019	0.00018
2.40	0.71013	0.71018	0.00018
2.36	0.71039	0.71017	0.00018
2.32	0.71048	0.71013	0.00018
2.27	0.70971	0.71008	0.00016
2.23	0.71038	0.71009	0.00015
2.18	0.71000	0.71008	0.00014
2.14	0.71036	0.71016	0.00021
2.09	0.71039	0.71012	0.00021
2.05	0.70972	0.71009	0.00020
2.00	0.71020	0.71016	0.00019
1.96	0.71003	0.71018	0.00020
1.91	0.71001	0.71027	0.00025
1.87	0.71000	0.71032	0.00024
1.83	0.70983	0.71029	0.00026
1.78	0.71022	0.71032	0.00025

1.74	0.71089	0.71030	0.00026
1.69	0.70990	0.71019	0.00023
1.65	0.71009	0.71021	0.00023
1.60	0.71041	0.71017	0.00025
1.56	0.71041	0.71015	0.00024
1.51	0.71093	0.71012	0.00023
1.47	0.71053	0.71007	0.00016
1.43	0.70974	0.71006	0.00015
1.38	0.71006	0.71011	0.00014
1.34	0.71000	0.71014	0.00015
1.29	0.70986	0.71016	0.00014
1.25	0.71008	0.71020	0.00013
1.20	0.70972	0.71025	0.00015
1.16	0.71016	0.71033	0.00011
1.11	0.71017	0.71039	0.00012
1.07	0.71035	0.71034	0.00018
1.02	0.71043	0.71031	0.00019
0.98	0.71027	0.71029	0.00019
0.94	0.71040	0.71036	0.00022
0.89	0.71019	0.71035	0.00023
0.85	0.71019	0.71034	0.00023
0.80	0.71066	0.71038	0.00023
0.76	0.71052	0.71036	0.00022
0.71	0.71069	0.71041	0.00025
0.67	0.70971	0.71033	0.00026
0.62	0.71005	0.71039	0.00022
0.58	0.71025	0.71040	0.00022
0.53	0.71093	0.71041	0.00021
0.49	0.71027	0.71035	0.00018
0.45	0.71013	0.71031	0.00021
0.40	0.71060	0.71033	0.00021
0.36	0.71048	0.71029	0.00022
0.31	0.71098	0.71027	0.00023
0.27	0.70994	0.71015	0.00013
0.22	0.71022	0.71019	0.00012
0.18	0.71018	0.71018	0.00014
0.13	0.71041	0.71018	0.00017
0.09	0.71024	0.71006	0.00016
0.04	0.70988		
